# Extensive recombination challenges the utility of *Sugarcane mosaic virus* phylogeny and strain typing

**DOI:** 10.1038/s41598-019-56227-y

**Published:** 2019-12-27

**Authors:** Luke Braidwood, Sebastian Y. Müller, David Baulcombe

**Affiliations:** University of Cambridge, Department of Plant Sciences, Cambridge, CB2 3EA United Kingdom

**Keywords:** Phylogenetics, Viral evolution

## Abstract

*Sugarcane mosaic virus* (SCMV) is distributed worldwide and infects three major crops: sugarcane, maize, and sorghum. The impact of SCMV is increased by its interaction with *Maize chlorotic mottle virus* which causes the synergistic maize disease maize lethal necrosis. Here, we characterised maize lethal necrosis-infected maize from multiple sites in East Africa, and found that SCMV was present in all thirty samples. This distribution pattern indicates that SCMV is a major partner virus in the East African maize lethal necrosis outbreak. Consistent with previous studies, our SCMV isolates were highly variable with several statistically supported recombination hot- and cold-spots across the SCMV genome. The recombination events generate conflicting phylogenetic signals from different fragments of the SCMV genome, so it is not appropriate to group SCMV genomes by simple similarity.

## Introduction

**Figure 1:**
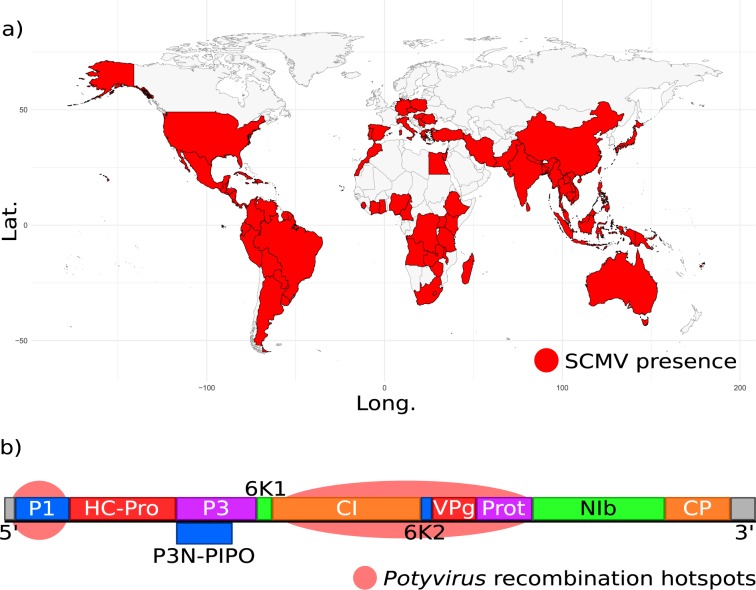
*Sugarcane mosaic virus* is a worldwide crop pathogen (**a**) Distribution of *Sugarcane mosaic virus* (SCMV), using country incidence data from CABI. Map generated using data from the maps package with ggplot2 in R v3.4.1^66^. Lat - latitude; long - longitude. (**b**) SCMV genome structure, showing final protein products (initially transcribed as a polyprotein), and previously reported potyviral recombination hot-spots.

*Sugarcane mosaic virus* (SCMV) is a positive-sense single-stranded RNA virus in the *Potyviridae* family (genus *potyvirus*), the largest and most economically damaging family of plant viruses^1^. SCMV can infect three major crops: sorghum, sugarcane (10–35% yield loss), and maize (20–50% yield loss), and is thought to be one of the top-ten most economically damaging plant viruses^2–4^. It has been reported in 84 countries across the 6 inhabited continents and this cosmopolitan distribution is likely due to worldwide trade in its host crops for hundreds of years (Fig. 1)^5^. Most potyviruses, including SCMV, are spread non-persistently by aphid species^6^ but SCMV can also spread via movement of infected root cane (sugarcane) and through maize seeds and  pollen^7,8^.

The *potyvirus* genus is notable for its size (>150 species) and extensive involvement in synergistic plant viral conditions. Typically, potyviruses enhance the titre of the partner virus in synergistic interactions through a process that is dependent on the multifunctional helper-component protease (HC-Pro)^9,10^. Synergism between potyviruses, including SCMV, and *Maize chlorotic mottle virus* (MCMV) causes maize lethal necrosis (MLN) that can cause total yield loss^11^. SCMV threatens both food security and economic development because maize and sorghum are vital staple foods, while sugarcane is an important cash crop. Despite SCMV being present in East Africa and China for decades, its impact in both regions has been enhanced by the recent arrival of MCMV, and therefore MLN^12–16^. Increased understanding of the variability and evolution of SCMV in these regions may inform future disease control measures.

SCMV has a typical *potyvirus* genome: a roughly 9.5 kb monopartite positive-sense single-stranded RNA molecule (Fig. 1b) which is packaged into around 2,000 helically arranged coat protein (CP) monomers to form flexuous virions 750 nm long and 13 nm wide. The 5′ end of the genome is capped by the 25 kDa Vpg protein, and the 3′ end is poly-adenylated. Translation of the genome produces a single polyprotein which is cleaved by three viral-encoded proteases to generate ten multifunctional proteins^1,17^. An additional protein, P3N-PIPO, is generated due to transcriptional slippage in the P3 gene at a conserved GAAAAAA motif during genome replication^18,19^.

*Potyviridae* evolution features extensive intra-specific recombination^20,21^, which likely occurs when the viral RNA-dependent RNA polymerase (RdRP) switches between viral genome templates^22^ during virus replication. Reported recombination hot-spots are in the P1 region of *Turnip mosaic virus* and in the CI-NIa-protease region of several species (Fig. 1)^23–34^. Predicted recombination breakpoints in the SCMV genome are in CI, NIb, NIa-VPg, and NIa-Pro, and the 6K1-VPg-NIa-Pro-NIb region has been called a recombination hot-spot, although without statistical support^28–34^. Recombination complicates phylogenetic analyses because various genome regions in a single individual may have different evolutionary histories. Accordingly, constructing phylogenies using different sections of SCMV and other potyviral genomes produces conflicting trees^35,36^. Recombination may also impede virus detection because increased genomic variation may lead to false negative results with common techniques such as PCR and antibody ELISA^11,37^.

There are multiple potyviruses present in East Africa which could act as partner viruses to MCMV. Therefore, we decided to survey MLN-infected maize in Kenya and Ethiopia using next-generation sequencing (NGS) to allow identification and analysis of the partner viruses in this region. The only partner virus we detected was SCMV, and these data were then used to look for signals of historical recombination in the SCMV genome. We also assessed the suitability of traditional phylogenetic methods for SCMV genomic data.

## Results and Discussion

### Sequencing of MLN-infected maize reveals SCMV

In August 2014 we collected 23 MLN-symptomatic (mosaic and chlorosis on leaves) maize samples from 13 Kenyan and 4 Ethiopian sites (Table S1) and performed NGS RNA-seq (ArrayExpress accession: E-MTAB-7002). All samples contained both MCMV, characterised previously^38^, and SCMV (Fig. S1). The 23 assembled SCMV sequences ranged from 2,191 bp to 9,632 bp, which is 23% to 100% of the longest previously reported SCMV sequence (available in GenBank: MH093717-MH093739). We aligned and manually trimmed the long (>8000 bp) SCMV contigs to the full SCMV genomes available in GenBank for further analysis.

**Figure 2:**
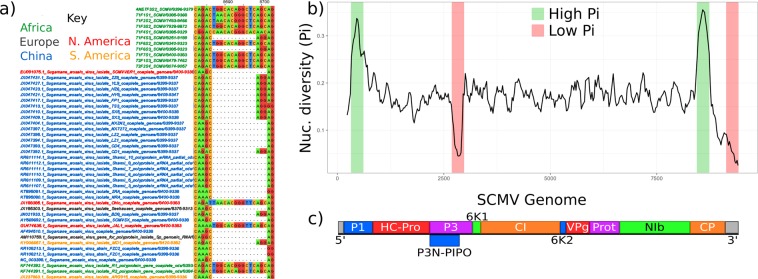
Structural and sequence variation in *Sugarcane mosaic virus* (**a**) Nucleotide alignment of *Sugarcane mosaic virus* (SCMV) genomes, showing insertion/deletion polymorphism in the coat protein gene. (**b**) Nucleotide (nuc.) diversity across the SCMV genome, with a window size of 100 bp and step size of 25 bp. (**c**) SCMV genome structure, showing final protein products, aligned with diversity graph in (**b**).

There were small insertions in the 5′ and 3′ untranslated regions (UTRs) in one (JX047421.1) and three (JX185303.1, EU091075.1, GU474635.1) isolates respectively but most insertion/deletion variation was over a 200 bp region of the CP coding sequence (Figs. 2a and S2, S3). CP indels were not distributed according to the geographic location of isolates. A 15 bp insertion, for example, was present in isolates from Ethiopia (1), Kenya (9), Rwanda (2), USA (1), and Mexico (1) whereas a 3 bp insertion at the same locus is present in Kenya (2), China (10), and Ecuador (1).

We assessed nucleotide polymorphism diversity of multiple sequence alignments using DNAsp5^39^. Diversity was high with 4,289 mutations spread over 2,831 sites and an average of 1121.1 nucleotide differences between sequences. Nucleotide diversity across the genome was 0.17, higher than for most RNA viruses but within the range previously reported for SCMV^30,33^. There were high polymorphism regions in the N-termini of P1 and CP and the most conserved regions were in the central domain of P3 and the 3′ UTR (Fig. 2b,c). P1 is a serine protease with a known hyper-variable region^40^. The variable region in the CP N-terminus (Figs. 2b,c and S2, S3) corresponds to a domain with variable amino acid length and low conservation^41,42^, with episodic positive selection detected by MEME analysis (Fig. S4)^43,44^. This N-terminal domain is surface located, raising the possibility that variation in this region may alter interactions with host or vector proteins^45,46^.

The conserved P3 protein is essential for potyviral replication but it is also the locus of the cryptic fusion protein P3N-PIPO and this overlapping open reading frame is an extra constraint to evolution of the nucleotide sequence^47^. Interestingly, in the P3N-PIPO region, there were also sites with episodic positive selection detected by MEME (Fig. S4). The 3′ UTR of potyviruses contains a poly-A tail to promote genome stability and translation which is completely conserved.

### Evidence for SCMV recombination

**Figure 3:**
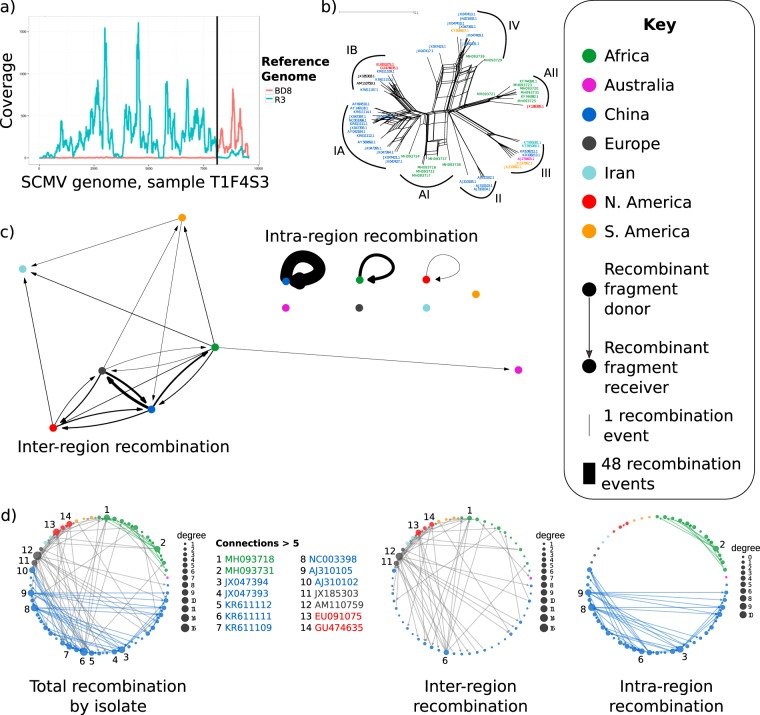
Widespread recombination between the ancestors of geographically separated *Sugarcane mosaic virus* (**a**) Bowtie2 alignment of sample T1F4S3 to two divergent *Sugarcane mosaic virus* (SCMV) reference genomes (JN021933.1 and KF744390.1). Reads are assigned to the reference with the best alignment, and the rapid switch in alignment preference is indicated by the black line. (**b**) Splits network of SCMV genomes, distances calculated with uncorrected P, and network generated by neighbour-net in Splitstree V4.6. The reticulate network indicates conflicting phylogenetic signals within the alignment, suggesting recombination. (**c**) Network showing recombination events within and between geographic regions, predicted by Recombination Detection Programme 4 (RDP4). Inter-region recombination refers to recombination events in which the donor (parent) and receiver (child) isolates are from different regions. Intra-region events refer to those in which the parent and child are from the same region. Wider arrows correspond to more recombination events. (**d**) Network showing RDP4-predicted recombination events between individual isolates, with node size determined by the number of recombination events. In (**b–d**) colour indicates geographic region.

The alignment patterns of several samples suggested recombination, with different regions of the same sample genome showing closest alignment to divergent reference genomes (Fig. 3a). To simplify the analysis whilst retaining maximum diversity, we subsampled the alignment of 116 sequences. We generated a nucleotide identity matrix (Table S2) and grouped sequences with >99% similarity, then kept the longest sequence in each group. This produced a final dataset of 55 SCMV genomes/contigs, including 13 from our NGS libraries.

Splits network analysis can detect and visualize conflicting signals from a phylogenetic dataset^48^. Conflicting signals imply that the relationship between sequences is different depending on the part of the sequence being analysed, and they can be caused by recombination or horizontal gene transfer. Splits networks with reticulate shapes rather than bifurcating tree shapes indicate conflicting phylogenetic signals. Here, we found the splits network derived from our SCMV sequences to be very different from a bifurcating tree indicating conflicting phylogenetic signals and implying recombination (Fig. 3b). Additional independent evidence for SCMV recombination comes from the distribution of multiple indels that do not correlate with geographic or phylogenetic proximity (Figs. 2a and S2, S3).

To estimate the number and location of recombination breakpoints we used Recombination Detection Programme 4 (RDP4) to predict the locations of recombination events in SCMV^49^. RDP uses multiple algorithms to locate sites in an alignment at which phylogenetic signals change rapidly, which is indicative of a recombination event. The recombination scheme suggested multiple recombination events, with many between geographic regions (Fig. 3c,d). There was notable reciprocal exchange of recombinant fragments between European and Chinese isolates (Fig. 3c,d), and between Chinese and African isolates. There was also evidence for intra-region recombination in the regions with more than five isolates (China and Africa). Recombination was more frequent between strains within a region than between strains in different region, as expected. Additionally, there were 28 recombination events with unknown parents (i.e. genome fragments not closely related to any known isolates), demonstrating that more sequencing data will be required to fully describe worldwide recombination patterns.

**Figure 4:**
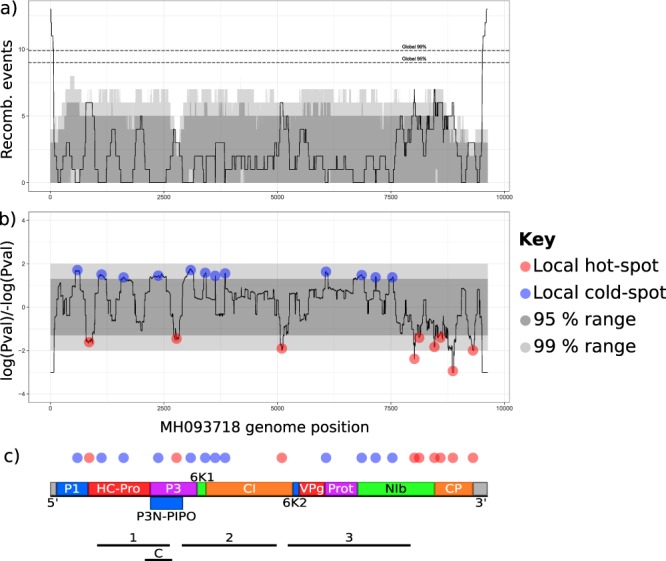
Recombination hot- and cold-spots in the *Sugarcane mosaic virus* genome (**a**) Recombination events across the *Sugarcane mosaic virus* (SCMV) genome in a 200 bp window. (**b**) P-value distribution for recombination frequency across SCMV genome. Grey ribbons show the local 95% and 99% confidence intervals as determined by permutation test. (**c**) SCMV genome structure, showing final protein products and recombination hot- and cold-spots, aligned with graphs in (**a**,**b**). Sections used for statistical analysis of conflicting phylogenetic signals are shown. C = control.

To search for regions of the genome with an over- or under-representation of recombination, we counted the recombination breakpoints in sliding windows across the SCMV genome (Fig. 4a), calculated likelihood ratios, and used permutation testing to identify statistically significant regions (Fig. 4b). The permutation test randomly places recombinant fragments spanning the same number of variable nucleotide positions as each detected recombinant fragment, which controls for sequence variability and generates a density map of where recombination is more likely to be detected in the SCMV alignment.

‘Global hot/cold-spots’ were defined as those with more breakpoints than 95% of the sliding windows across the genome, and ‘local hot/cold-spots’ were those with more breakpoints than 99% of sliding windows at that position. Global recombination hot-spots were present at the 5′ and 3′ genomic termini. We detected nine local recombination hot-spots, and twelve local recombination cold-spots (Fig. 4). The hot-spots were concentrated in the 3′ region encoding NIb and CP, with single hot-spots in *CI*, *P3N-PIPO*, and the *P1/HC-Pro* junction (Fig. 4c). The cold-spots were distributed more uniformly across the first 7500 bp of the genome. These are the first statistically supported recombination hot- and cold-spots reported in SCMV.

Recombination can promote nucleotide diversity by mixing lineages, and we note that the 3′ genomic region encoding CP in SCMV has high nucleotide diversity (Fig. 2), and a concentration of recombination hot-spots (Fig. 4). *Potyvirus* recombination hot-spots have previously been observed in the C-terminal region of *CI*, which we also observed, and in P1, which we did not^23–27^. Recombination is clearly a major force in SCMV evolution (Fig. 3), as in the *Potyviridae* generally^20,50^.

### Is making an SCMV phylogeny useful?

The purpose of a phylogeny is to describe the evolutionary history of biological entities. This exercise has academic value, in tracing the history of life, and practical value, in organising similar biological entities into clades. If a phylogeny does not describe evolutionary history, and does not group biological entities into self-similar clades, it is an inappropriate analysis (due to the methodology or the underlying data) and does not contain useful information. Given the high levels of recombination between our SCMV sequences, we decided to investigate whether further phylogenetic analysis is appropriate.

There are many published SCMV phylogenies, based on CP sequences and whole genomes, which place isolates into two to six strains with variable names, see Gao *et al*. (2011) for a helpful summary^51^. Whole genome phylogenies of SCMV group isolates into four strains (IA, IB, II, III, IV), with around 80% nucleotide similarity between strains^33,51^. African SCMV genomes sequenced in this study form two novel clusters (AI and AII) of sequence identity, decreasing the separation between previously reported strains (Fig. 3b).

Simulations show that phylogenetic analyses are most severely impacted by recombination when breakpoints occur near the centre of alignments, and by recent recombination between diverged taxa^52^. Our recombination analysis shows evidence of recombination between divergent (<80% nucleotide identity) SCMV isolates, in the centre of both genomes and CP sequences (Figs. 3 and S5, Table S3). Therefore, there is no single evolutionary history for phylogenetic analyses to infer. To statistically test for conflicting phylogenetic signals, we constructed phylogenies by maximum likelihood using the whole SCMV genome, and three sections of the alignment (section 1: positions 963–2,764 in the original alignment, section 2: 2,875–5,103, and section 3: 5,181–8,036) without recombination hot-spots (Fig. 4, Supplemental Data d1). We chose alignment sections with a minimum number of recombination events (i.e. containing cold-spots) which were separated with recombination hot-spots (Fig. 4c). Tree incongruence was tested statistically using a Shimodaira-Hasegawa test (SH-test) for each pair of trees. The log likelihood differences were 12,222 between sections 1 and 2, 12,739 between 1 and 3, and 15,692 between 2 and 3 (p < 1e-7 and n = 2 trees for all comparisons), confirming significant differences between the trees, which can be visualised using tanglegrams or identity matrices (Fig. S6).

## Conclusion

Viral studies often present a phylogeny followed by evidence of extensive recombination, showing that the central assumption of the phylogenetic analysis was violated^23,30,31,33^. Multiple evolutionary histories within a genome are valid and averaging these different histories does not produce the true evolutionary history of the genome^52^. Imposing a bifurcating tree structure on a dataset which does not have a single, bifurcating evolutionary history will introduce systematic error. We argue that in organisms with unknown or high recombination rates, such as RNA viruses, recombination analyses should be performed initially, then used to inform the phylogenetic approach taken, as in Ohshima *et al*.^25^. Splits network analysis is appropriate for all alignments, but standard phylogenetic methods may not be, depending on the splits network results. Phylogenetic analyses of whole genomes may be desirable to identify viral strains containing isolates with a broadly similar evolutionary history. However, the presence of sequences from different strains which have entered due to recombination may confound phenotyping and molecular attempts at strain identification in the field.

We have shown that SCMV is in complex with MCMV in MLN-infected maize in East Africa, and that producing SCMV phylogenies does not produce useful classification systems or describe biological truth (Fig. S6)^52^. We conclude that constructing phylogenetic trees is inappropriate for SCMV due to extensive historical recombination between divergent isolates. This may also have implications for studies of other RNA viruses, and phylogenies of other organisms with high recombination rates. There are multiple avenues for progress in this field - for example the statistical framework for assessing splits networks is not well developed, there are no automated approaches for locating viral recombination hot-spots, and visualisation of reticulate recombination networks.

## Methods

### NGS of MLN-infected maize

We collected maize leaf samples from Kenya and Ethiopia in August 2014 (Table S1), storing samples in RNA-later (Ambion) on dry ice. To extract RNA, we used Trizol (Ambion) according to manufacturer’s instructions. We depleted ribosomal RNA with the Ribo-Zero Magnetic Kit (Plant Leaf - Epicentre). To generate indexed stranded libraries, we used Scriptseq V2 RNA-Seq Library Preparation kits and Scriptseq Index PCR primers (Epicentre). Library concentration and quality were confirmed using Qubit (Life Technologies) and a Bioanalyzer High Sensitivity DNA Chip (Agilent Technologies). Beijing Genomics Institute performed 100 bp paired-end sequencing on one lane of a HiSeq 2000 (Illumina).

### NGS quality control

We used a custom python script to demultiplex the libraries allowing one error in index sequences, then trimmed adaptors using Trim galore! (parameters:–phred64–fastQC–illumina–length 30–paired_retain_unpaired input_1.fq input_2.fq)^53,54^. String matching deduplication (deletion of identical reads) was performed using Quality Assessment of Short Read (QUASR) pipeline scripts^55^.

### SCMV consensus sequence generation

To generate SCMV genome sequences, we aligned libraries to a bowtie2 reference containing all SCMV genomes available in NCBI GenBank in March 2016 (parameters: *-D 20 -R 2 -N 1 -L 20 -i S,0,2.50–phred64–maxins 1000–fr*)^56^. Next, we extracted SCMV-aligning reads and performed *de novo* assembly using Trinity (v2.0.2), extracted contigs above 2 kb in length, then inspected and curated (if necessary) SCMV contigs^57^. To generate SCMV consensus sequences, we aligned each library to its respective Trinity contig using bowtie2, generated pileups using samtools, and called sequences using the QUASR script *pileup_consensus.py*, with a threshold of zero or ten % of reads for the calling of ambiguity codes (parameters: *-ambiguity 0*–*10 -dependent -cutoff 25 -lowcoverage 20*)^58^.

### SCMV alignment and diversity analysis

SCMV genomes generated in this study were combined with those in GenBank and aligned using MUSCLE (gap extension cost: 800, other settings default) in MEGA6, with separate alignments for genomes called with and without ambiguous bases^59^. We checked the alignments manually in JALview and refined where necessary^60^. To construct a nucleotide identity matrix we used the dist.alignment function from the R package seqinr. We obtained diversity metrics using the alignment without ambiguous base calls in DnaSP v5^39^.

### SCMV recombination analysis

Recombination analysis was performed with the alignment containing no ambiguous base calls. To generate splits networks, we used SplitsTree4 using default settings - distances calculated by uncorrected P, and network generated by neighbour-net^48^. To generate more specific predictions of recombination, we used RDP4, using the algorithms RDP, GENECONV, MaxChi, BootScan, and SiScan (all default settings), and reviewed all breakpoints manually. Recombination network diagrams were generated by constructing interaction matrices for regions and SCMV isolates. The interaction matrix was converted into a regional recombination network (Fig. 3c) using the ggraph function from the ggraph R package, while the individual interaction networks (Fig. 3d) were constructed using the ggnet2 function of the R package GGally.

### Dendrogram and phylogeny construction

The nucleotide identity dendrogram was constructed from the identity matrix using the heatmap.2 function of the gplots R package. Phylogenies were constructed from the alignment without ambiguous bases using RAxML-HPC2 (8.2.10) on XSEDE, hosted by the CIPRES science gateway (parameters: -T 4 -N autoMRE -n result -s infile.txt -m GTRCAT -q part.txt -c 25 -p 12345 -f a -x 12345–asc-corr lewis)^61^. Phylogenies were compared using the tanglegram function of Dendroscope (3.5.9)^62^.

### Statistical analysis

We used an SH-test to test for tree incongruence^63^ between phylogenies constructed using the three alignment sections. Trees for each section were generated using the methods above and compared using the SH-test as implemented in the R-package phangorn^64^ in a pairwise fashion. To determine whether the SH-test is appropriate for these data we created a negative control dataset from our alignment in which we did not expect tree disagreement. In the negative control dataset, the null hypothesis (of identical tree architectures) should not be rejected. To generate the negative control dataset, we divided a region containing a recombination cold spot (positions 2,224 to 2,586 of the original alignment), which should have had little recombination and therefore have a consistent evolutionary history into two sections (positions 2,224 to 2,400 and 2,401 to 2,586). The log likelihoods of the tree topologies constructed from these sections were −5,895 and −5,735 respectively with a difference of 59.8. Subsequent testing with the SH-test did not reject the null hypothesis of the topologies agreeing (p = 0.21).

## Supplementary information

SupplementaryF Information 

Supplementary Information 2

 Supplementary Information 3

Supplementary Information 4

Supplementary Information 5

## Data Availability

RNA-seq data have been deposited in the ArrayExpress database^65^ at EMBL-EBI (www.ebi.ac.uk/arrayexpress) under accession number E-MTAB-7002. SCMV contigs are available in Genbank under accession numbers MH093717-MH093739.
