# Supplementary material for: Extensive recombination challenges the utility of Sugarcane mosaic virus phylogeny and strain typing
Source: Sci Rep. 2019 Dec 27;9:20067. doi: 10.1038/s41598-019-56227-y (PMC6934591; doi:10.1038/s41598-019-56227-y)
Supplement: Supplementary file 1 — SupplementaryF Information [file 41598_2019_56227_MOESM1_ESM.pdf]

Extensive recombination challenges the utility of *Sugarcane mosaic virus* phylogeny and strain typing

Luke Braidwood[1,*], Sebastian Y. Müller[1], and David Baulcombe[1]

[1] University of Cambridge, Department of Plant Sciences, Cambridge, CB2 3EA, United Kingdom

* braidwoodluke@gmail.com

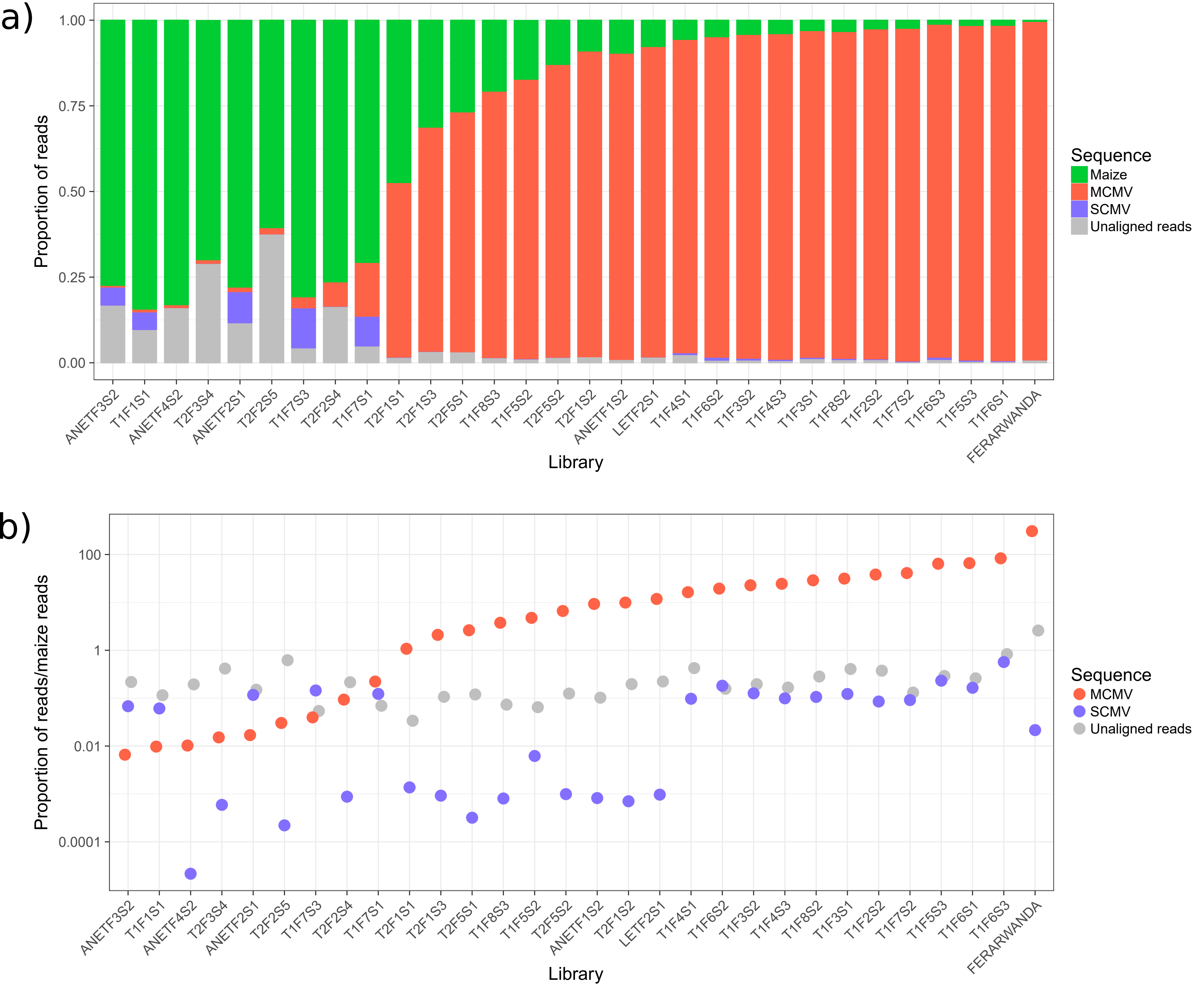

Figure S1. ***Maize chlorotic mottle virus* and *Sugarcane mosaic virus* are present in maize lethal necrosis-infected East African Maize**. a) Proportion of reads from next generation sequencing libraries from Kenya, Ethiopia, and Rwanda aligning to the maize, Maize chlorotic mottle virus, and Sugarcane mosaic virus genomes. b) Virus-aligning and unaligned reads on a logarithmic scale.

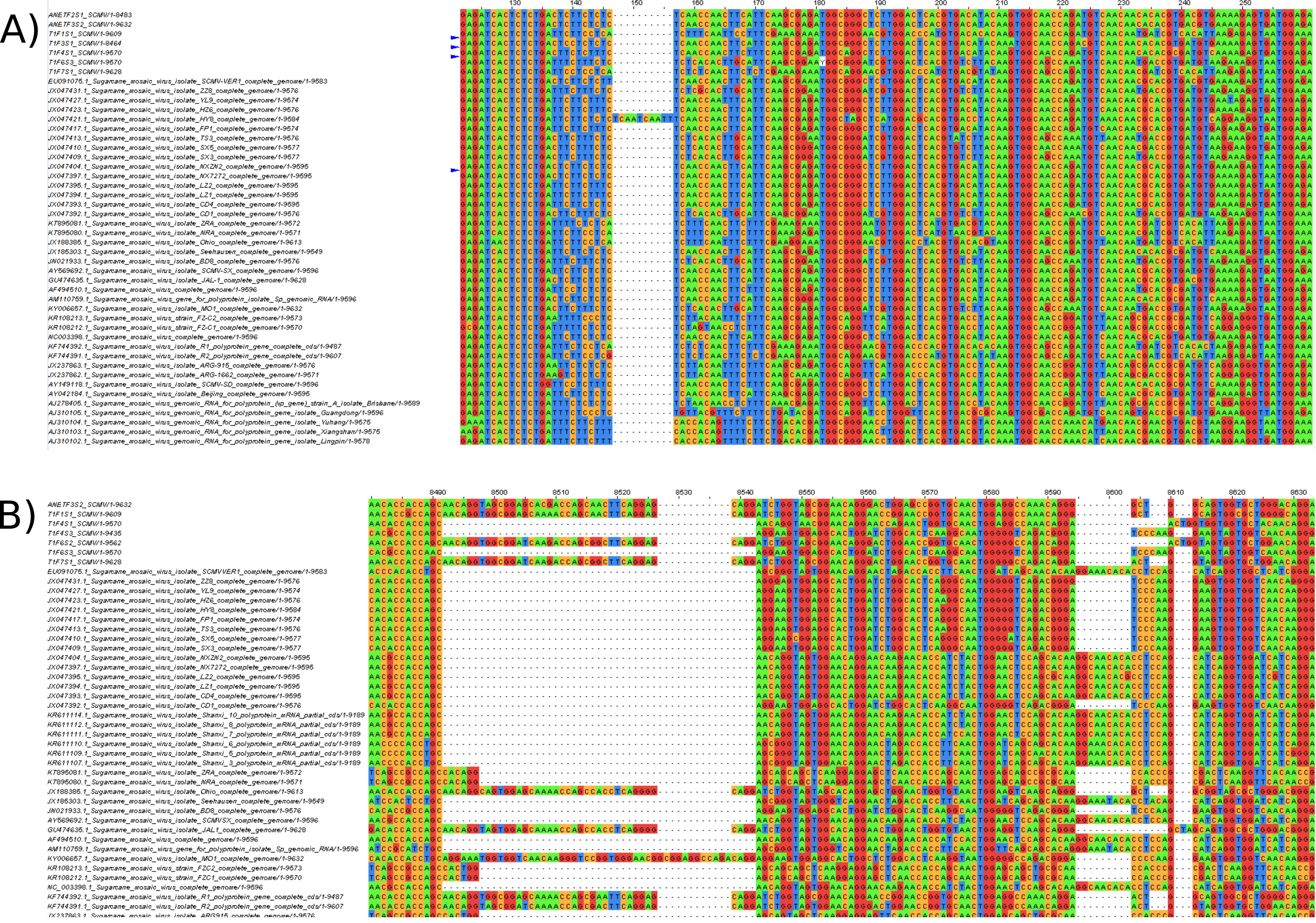

Figure S2. Structural variation in *Sugarcane mosaic virus* genome alignments. a) Structural variation in 5' untranslated region. b) Structural variation in coat protein coding sequence.

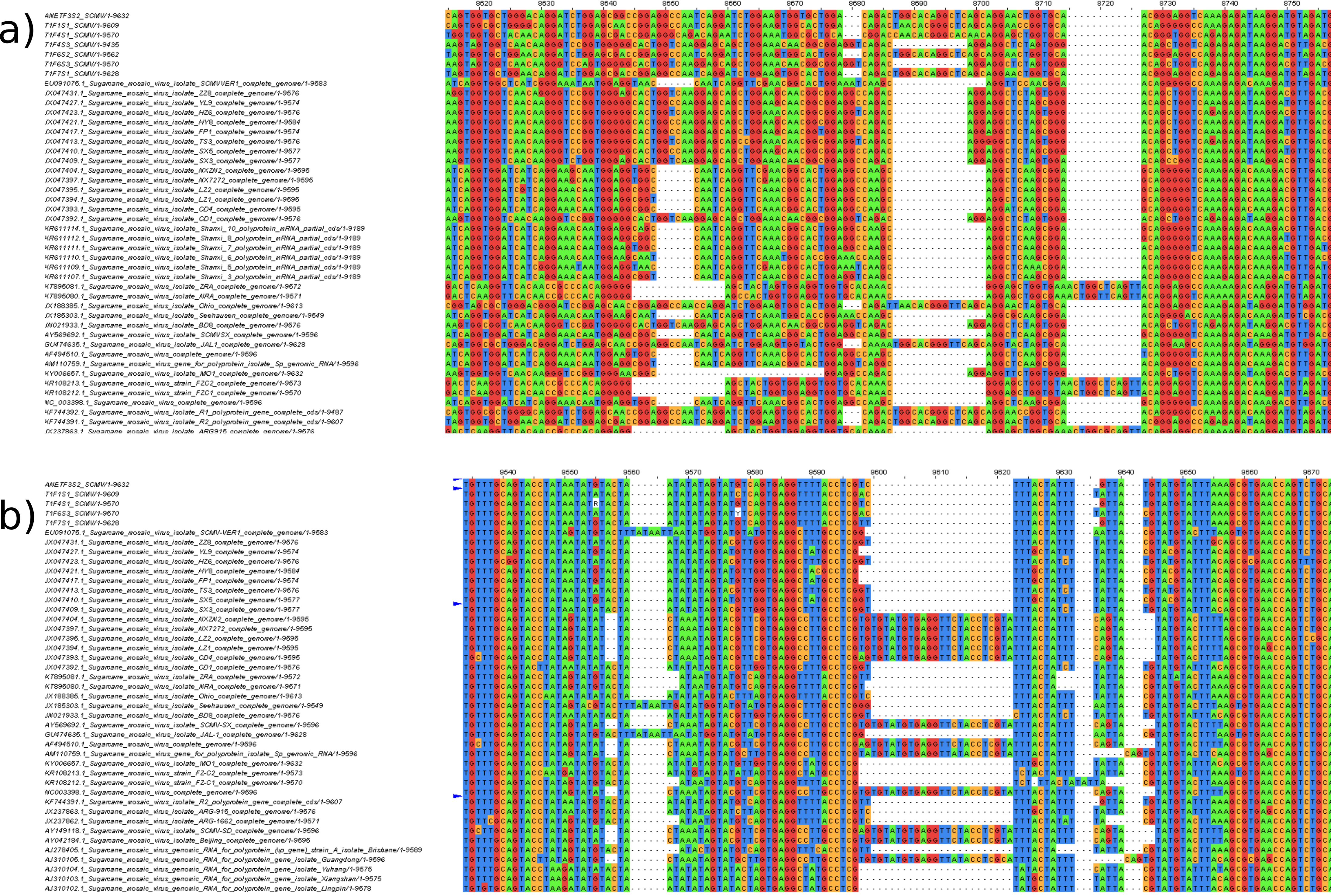

Figure S3. Structural variation in *Sugarcane mosaic virus* genome alignments. a) Structural variation in the coat protein sequence. b) Structural variation in the 3' untranslated region.

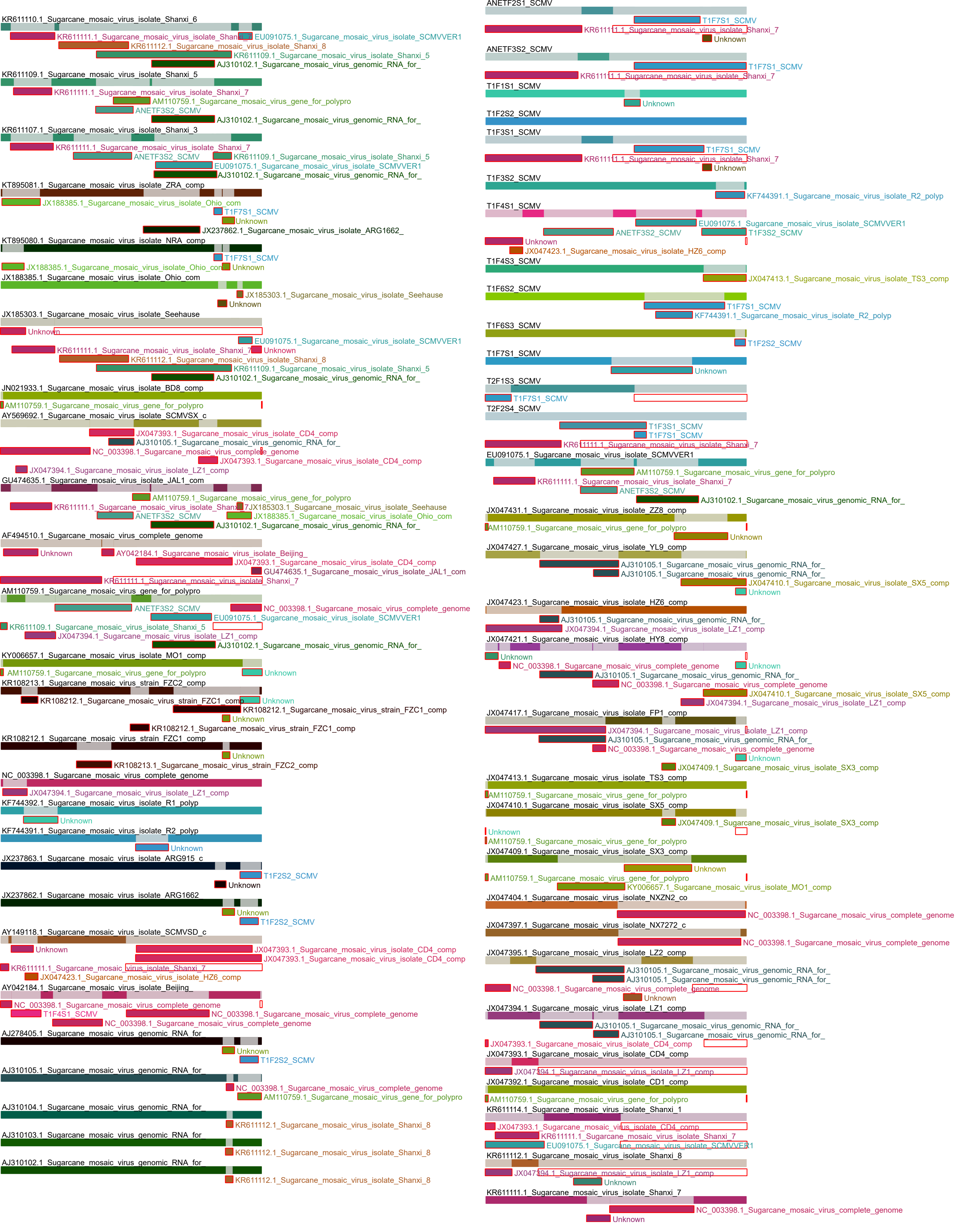

Figure S4. Recombination scheme for *Sugarcane mosaic virus*. Recombination hypothesis as generated by Recombination Detection Programme 4 (RDP4) and manually reviewed.

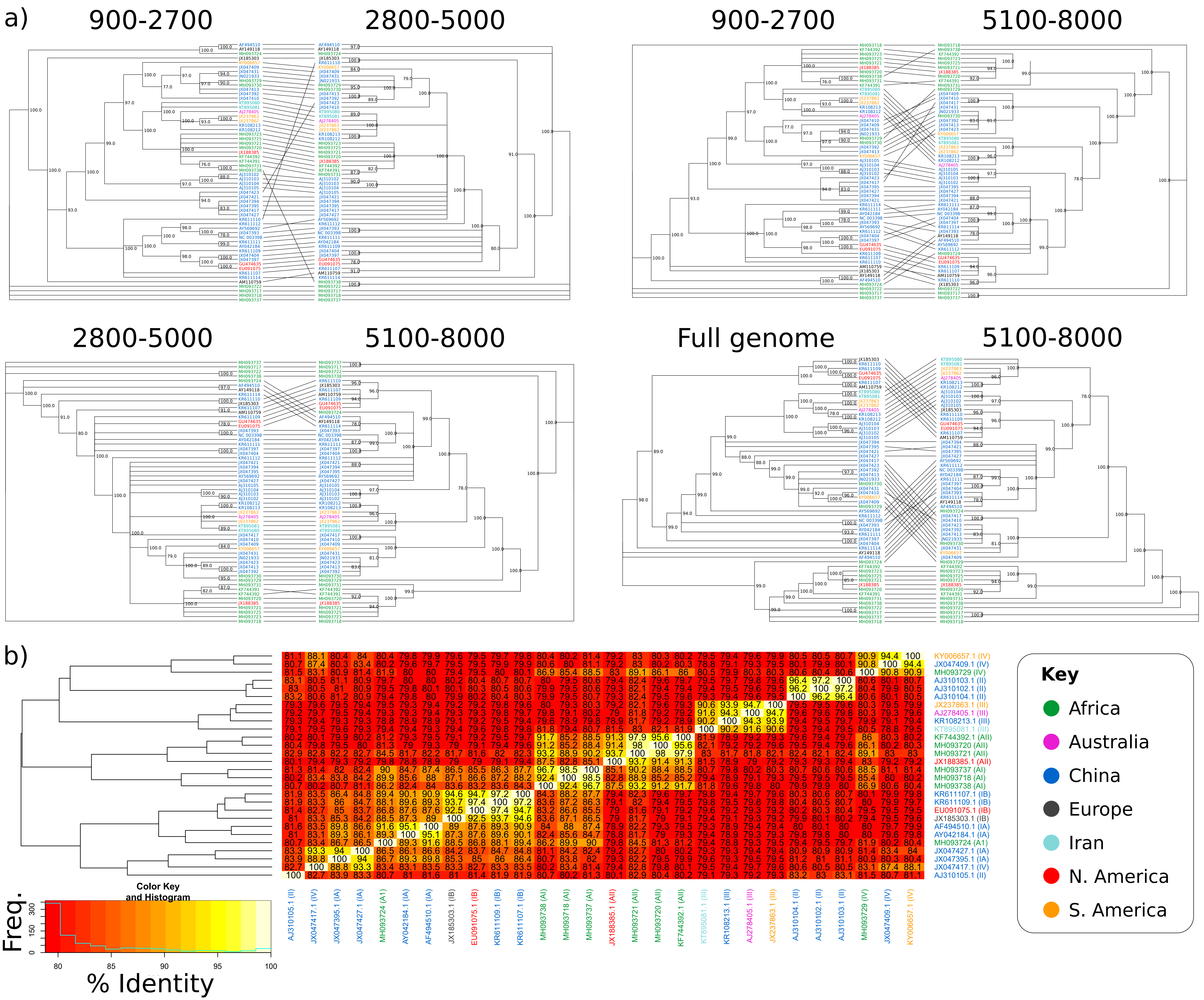

Figure S5. Conflicting phylogenetic signals in the *Sugarcane mosaic virus* genome. a) Tanglegrams showing differences in phylogenetic architecture when *Sugarcane mosaic virus* (SCMV) genomes and SCMV genome fragments are used to infer phylogenetic relationships. b) Identity dendrogram of SCMV genomes with strain numbers indicated in brackets, showing that sequences do not cluster according to previously reported strains. Label colours indicate geographic region.
