# Supplementary material for: Extensive recombination challenges the utility of Sugarcane mosaic virus phylogeny and strain typing
Source: Sci Rep. 2019 Dec 27;9:20067. doi: 10.1038/s41598-019-56227-y (PMC6934591; doi:10.1038/s41598-019-56227-y)
Supplement: Supplementary file 5 — Supplementary Information 5 [file 41598_2019_56227_MOESM5_ESM.docx]

**Extensive recombination challenges the utility of *Sugarcane mosaic virus* phylogeny and strain typing**

**Luke Braidwood^1,^*, Sebastian Y. Müller^1^, and David Baulcombe^1^**

^1^ University of Cambridge, Department of Plant Sciences, Cambridge, CB2 3EA, United Kingdom

* braidwoodluke@gmail.com

**Supplementary data d1.** Fasta alignment of SCMV nucleotide genome sequences used in this study. The text below can be saved as a .fasta/fas/fa file to view in alignment editors.

>MH093717

--------------------CGGGCACACAAAACTCAACACAACACAACAAAACACGACCAAACAAAACCAAGTTACTTTTGCTCAGATTGTAGTGAACGGCTCGGTGGGAAAGGTTCCTCGAGATCACTCTCTGACTCTTCTCTC----------TCAACCAACTTCATTCAAGCGAGATGGCGGGCTCTTGGACTCACGTGACATACAAGTGGCAACCAGATGTCAACAACACACGTGACGTGAAAAGAGTGATGGAGATGTTTGTAGCAAAACATCAACGTTACACTGAGGAACAAAGGCTTGCTCACAACAGCAAGCTGTTAAGGAAGGCTTGTGTTACTAGTGCTGAGTTTACTGAGTCAGCACAGAAACCAAAATGTCATCAAACATGGGTTGAAAAGTGTGACCACAACCCCACAGAGCACTTTGTTTATCAACGTTTC---ACACCCGAGAAGAAAGTGCCAGCCACCAAACCTGAGACAACTTCTGTCACGAAGTTAATCAGGGAAATCCTTGAGATTTCGAAGGGCAGTGGGATAAAAATTGAATTAATTGACAAGCGTGTCAAACGTAAAACTCAATTATCCATAAGGCGACACAATGGTAAAGATTTCTTGCACTGCAAAACCAGGCATGAAAATGGCCTGTTTAAACGCAAGGACGTTGACATTAGTGTCAAGTGGTTGCCCACCATTGAAGCCATTGCAAAATGCTACAGCACTGTGAATGCAGGAGAGTTGCAAAGTCTCAGTAGGGGCAGTAGTGGTCTAACATTCATGCAGAATGATGAATTATTCATCGTGCGTGGAAGAATGCATGGTGAGATTGTTAATAGTTTGCATGAAAATAAGCACGTAATGGAAATCGAACACTATGCTGATCCACAGGCGAATAGTTTCTGGAAAGGCTACACAGATGCGTATGTCGAGAACAGAAACATATCCACTACTCACACAGAGCACACACCAACTATCAATTTAGAAGAGTGTGGCAAGAGAATGGCACTGTTAGAAATCTTATTCCATTCAACTTTTAAAATAACATGCAAAACATGTAATATTGACGATCTTGAATTATCAGATGATGAATTTGGGGCCAAGTTATATAGTAATCTGCAGCGCATTGAAGAAAAACAACGTGAATATCTTGCTAAAGATCAAAAACTTTTACGCATGATTCATTTTGTAAAGGATCGATGTAATCCAAAGTTTTCACATTTACCCTTGCTATGGCAAGTGGCAGAAACAGTGGGGCATTACACTGATAACCAATCGAAGCAGATAATTGATATCAGTGAGGCGCTCATAAAAGTTAATACCTTAACTCCTGATGATGCAGTAAAGGCCAGTGTGGCATTATTGGAAGTAGCACGGTGGTATAAGAACCGGAAGGAATCACTTAAAACGGACACATTGGATTCATTTCGAAACAAGATTTCACCAAAGAGCACGATCAACGCAGCATTAATGTGTGATAACCAGTTAGATAAGAATGCAAATTTCGTGTGGGGAAACAGAGAATACCACGCAAAGCGATTCTTCGCTAACTATTTTGAAGCTGTGGACCCAACTGATGCATATGAAAAACATGTAACACGCTTTAACCCCAATGGACAGCGGAAATTATCAATTGGCAAACTAGTAATCCCATTAGATTTCCAGAAAATCAGAGACTCGTTCGTTGGCCTATCAATAAATAAACAACCGCTGAGCAAAGCTTGCGTAAGTAAAATCGATGGAGGTTACGTGTATCCATGTTGCTGCGTTACAACAGAATTTGGAAAACCAGCATATTCTGAGATAATACCTCCAACGAAAGGACATATCACGATTGGAAATTCAGTGGACCCGAAAATAGTGGATCTACCAAATACAACACCACCGAGTATGTACATTGCAAAAGATGGATACTGTTATATTAACATATTCTTGGCAGCAATGATAAACGTCAACGAGGAATCCGCAAAAGATTACACTAAGTTTCTTAGAGACGAGTTGGTGGAACGGCTTGGTAAATGGCCAAAATTGAAAGATGTGGCCACAGCATGCTATGCTTTGTCAGTGATGTTCCCAGAGATAAAGAATGCCGAATTACCACCAATACTAGTGGATCATGAGAGTAAGTCAATGCACGTCATTGATTCATATGGATCACTCAGCGTTGGCTTTCACATTCTAAAGGCAAGTACTGTTGGACAACTGATAAAATTTCAGTACGAGTCATTGGAAAGTGAGATGCGCGAGTACATAGTGGGTGGCACTTTAACACAGCAAACTTTCAGCACACTTCTTAAGACTCTCACAAAGAACATGTTTAAACCAGATAAAATAAAGCAGATAATTGAGGAAGAGCCATTCTTATTAATGATGGCAATTGCATCCCCAACTGTACTCATCTCGCTGTACAACAACTGCTACATCGAGCAAGCAATGACATATTGGATCGTCAAGAACCAAGGCATCGCAGCGCTTTTTGCGCAGTTGGAGGCACTAGCAAAGAAAACTTCTCAAGCGGAGCTACTAGTTCTTCAAATGCAAATACTTGAGAAAGCTTCGAACCAACTGAGACTTGCAGTCACGGGACTTAATCATGTTGATCCAGCTAAACGACTTTTATGGTCGCACCTGGAAGCTATGACAACACGGTCAGAGATGAATAAGGAACTCATAGCGGAAGGCTATGCACTGTACGACGAGCGCTTATACACTTTAATGGAAAAAAGTTACGTAGATCAATTAAACCAATCATGGGCAGAATTATCATACTGTGGAAAATTTTCAGCAATATGGCGTGTGTTCAGAGTCAGGAAATACTACAAACCATCTTTAACCGTGAGAAAAAGCGTAGATTTAGGCGCTGTGTACAATATATCAGCTACGCATCTAATATCAAATTTAGTGCAGAAAAGTCGAGATCAAGTCAGCTCTACTTTAACCAAACTCCGCAACGGTTTCTATGATAAAATGGAGAGAGCGAGAGTTAGTGCAGTAAGGACAGTATATTGGTTCGTACCCGACATATTTAGACTTATACATATTTGCTTAGTTTTAAGTATATTAACAACTATAGCTAATACAATAATCGCAATTATGAATGATTATAAAAAGTTGAAAAAGCAACAAAGAGAAGACGAATACGAAGCCGAGATTAACGAGGTGCGAAGGATACACGCCAACCTAATGAAGGAGCATAATGATGACTTAACATGTGAACAATTTATTGAGCACATACGACAGACACATCCACGCCTCATTGAGGCAACATTGGATTTAACACATACAGGTGTCATCCATGAGGGCAAATCCAATTTAGAAACAAACCTCGAACAGGCAATGGCAGTGGGAACTTTACTCACTATGATACTCGATCCACAGAAGAGTGATGCAGTTTATAAGGTTCTCAATAAGATGCGAACAGTTATCAGCACAATAGAACAGAATGTACCATTTCCTTCAGTGAACTTCACGAGCATCTTGTCACCTCCTGTAGCTCAGCAAAGTGTAGATGTTGACGAACCGTTGACACTGAGTACCGATAAAAATTTGACTATAGATTTCGACACAAATCAAGATTTGCCAGCGGACACATTTAGCAATGACGTTACGTTCGAGAACTGGTGGGCTAATCAGATAAACAACAACAGAACAGTGCCACACTATCGACTTGGGGGAAAGTTTGTAGAATTCACAAGAGAGAATGCAGCAATGGTTAGCATTGAGCTCGCCCACTCGAACATCGAAAAAGAGTTTCTACTCAGAGGAGCCGTTGGATCAGGAAAATCCACAGGTTTGCCATATCATCTCAGTATGCGTGGAAAAGTGCTATTGATAGAACCTACTCGACCATTAGCTGAGAACGTTTGCAGACAACTGCAAGGTCCTCCATTCAATGTGAGCCCCACTTTACAAATGAGAGGATTGAGCACATTTGGCTGCACTCCTATCACGATAATGACATCTGGCTTCGCATTGCACATGTATGCTAATAACCCCGATAAGATCTCTGAGTATGACTTCATTATCTTTGATGAATGTCACATTATGGAAGCACCTGCAATGGCATTCTATTGTTTGCTTAAGGAGTATGAATATCGAGGCAAGATAATAAAAGTTTCAGCTACACCACCAGGACGAGAATGCGAGTTTTCAACCCAACATCCAGTAGACATACATGTATGTGAAAGCTTGACACAACAGCAATTCATCATGGAATTAGGAACAGGATCAACTGCTGATGCAACCAAATATGGCAATAACATATTAGTGTACGTTGCAAGTTATAATGATGTAGATTCTTTATCCCATGCTCTAACTGAACTTAAATATTCAGTGATTAAAGTCGATGGAAGAACGATGAAGCAAAACACCACAGGAATCGTAACAAATGGAACATCCGGTAAGAAATGCTTCGTTGTGGCCACAAATATTATTGAAAACGGTGTGACGCTAGATGTCGACGTTGTCGTCGACTTTGGACTTAAAGTAACAGCTGAATTAGATGTTGATAACAGGGCGATAATGTATAAACGTGTGAGCATATCTTATGGCGAGCGCATTCAGAGACTCGGAAGAGTCGGAAGGAATAAGCCTGGGACAGTTATCCGCATCGGAAAAACAATGAAAGGCTTACAAGAAATTCCTGCGATGATTGCTACTGAAGCAGCTTTCATGTGTTTTGCATATGGACTAAAAGTTATAACACATAATGTATCAACAACACATCTAGCAAAATGCACTGTCAAACAAGCTAGAACCATGATGCAATTCGAACTATCACCATTTGTGATGGCTGAATTAGTTAAATTCGATGGTTCCATGCATCCACAGATTCATGAAGCGTTAACCAAGTATAAATTGAGGGATTCTGTGATCATGTTAAGACCAAACGCAATACCAAAGGTTAACCTTCACAACTGGTTAACGGCCCGTGATTACAATAGGATAGGCTGCTCACTGGAACTCGAGGATCACGTTAAAATACCATATTATATACGAGGAGTCCCTGACAAGTTGTATGGGAAGTTATATGATATCATCCTTCAATACAGCCCTACAAGTTGTTATGGAAGATTGTCAAGTGCTTGCGTGGGTAAGGTCGCATATACATTGCGTACTGATCCTTGTTCATTACCGAGAACAATAGCTATAATCAACGCACTGATTACTGAAGAGTATGCAAAGAGGGATCACTACAGAAACATGATAGCAAACCCCTCATCATCGCACGCCTTCTCACTTAATGGGCTGGTATCCATGATCGCGTCTCGGTATATGAAAGATCACACGAAGGAGAACATAGACAAACTTGTAAGAGTGCGTGACCAGCTACTTGAGTTCCAAGGCACAGGTATGCAATTTCAAGATCCTTCAGAATTGATGGATATTGGTGCATTAAATACAGTTATCCACCAAGGAATGGACGCCACGGCTGCTTGCATTGGATTACAAGGGCGTTGGAATGCTTCACTCATCCAGCGCGATTTGATGATATCAGCAGGTATCTTCACAGGGGGAATTCTTATGATGTGGTGTCTCTTTACAAAATGGAGCAAGACAGAAGTGTCACATCAAGGAAAGAACAAGCGCAGTAGACAAAAACTCCGATTCAAAGAAGCGAGAGACAACAAATATGCATATGATGTCACAGGATCGGAAGAGTGCCTTGGTGAGAATTTTGGAACAGCTTATACAAAGAAAGGTAAAGGAAAAGGAACTAAAGTTGGACTTGGTGTGAAGCAACACAAATTTCACATGATGTATGGTTTTGATCCTCAAGAGTACAACCTAATTCGATTTGTCGATCCACTCACAGGAGCAACTCTTGATGAGCAAATCCATGCCGACATACGCTTAGTTCAAGAGCATTTCGCTGAAATTCGTGAGGAGGCAGTAGCTAACGACACAATTGAAAAGCAGCATATCTACGGCAATCCTGGACTACAAGCATTTTTCATACAAAATGGGTCAGCAAACGCTCTAAGAGTTGATTTAACGCCACATTCACCTACACGAGTTGTCACAGGTAATAATATAGCAGGATTCCCAGAACATGAGGGTACACTCCGTCAAACTGGAACAGCAATAACCATACCCATTGGTCAAGTCCCAATCGCACGTGAAACAGGAGTTGCACACGAGTCAAAATCCATGATGAATGGGCTGGGTGATTACACGCCAATATCGCAACAGTTATGTTTAGTACAAAATGACTCAGATGGGATAAAGCGGAATGTGTTTTCAATTGGATATGGTTCATATCTCATTTCACCAGCGCACTTATTCAAATATAACAATGGCGAAATAACAATTAGATCATCAAGAGGATTGTATAAAATTCGTAATTCTGTGGATTTAAAACTACATCCGATTACACACAGAGACATGGTTATAATTCAGCTTCCAAAGGATTTCCCACCGTTCCCAATGCGTTTGAAATTCACACAACCATCACGAGATATGCGAGTCTGCTTAGTGGGAGTCAATTTCCAACAGAATTACAGCACTTGCATCGTATCAGAAAGTAGTGTGACAGCACCAAAAGGAAATGGAGACTTTTGGAAACATTGGATATCAACAGTCGACGGTCAATGTGGACTACCATTGGTAGACACTAAGAATAAACACATTGTCGGAATTCATAGTCTTGCATCTACAAGTGGAAACACCAACTTCTTTGTCGCCATGCCTGAGAACTTTAATGAGTACATTAATGAACTTGTGCAAACAAACAAATGGGAAAAGGGATGGCACTACAATCCGAATCTCATATCTTGGTGTGGATTAAACTTAGTCGACTCTGCTCCAAAGGGTCTGTTTAAAACGTCAAAATTGGTTGAAGATTTGGATGCTAGTGTTGAAGAGCAGTGCAAAGTTACTGAAACATGGCTCACAGAGCAATTACAAGATAATTTGCAAGTGGTCGCGAAATGTCCAGGCCAACTCGTAACTAAGCATGTCGTAAAAGGCCAATGCCCGCACTTCCAGTTATATTTGTCAACACATAATGACGCCAAAGAATATTTCGCACCTCTGCTTGGAAAATATGACAAGAGCAGACTCAATAGAGCGGCATTTATTAAAGACATATCAAAGTATGCAAAACCAATTTATATTGGAGAAATAAATTATGATATCTTTGATAGAGCTGTACAACGAGTCATTAATATTCTTAAAAATGTCGGAATGCAACAATGCGTTTATGTCACAGACGAAGAGGAAATTTTCAAATCACTCAACCTAAACGCAGCCGTCGGAGCACTGTACACAGGAAAGAAGAAAGATTACTTTGAAAGTTTTTCAAATGAAGATAAGGAAGAAATCGTGATGAGATCATGTGAACGCATTTACAATGGACAACTTGGTGTGTGGAATGGGTCACTCAAAGCCGAAATCAGACCAATAGAGAAAACCATGTTAAATAAGACTCGAACCTTTACAGCAGCTCCATTGGAAACTTTGCTTGGAGGAAAAGTGTGTGTGGACGATTTCAATAATCAATTTTATTCACACCATTTAGAAGGCCCATGGACTGTTGGGATAACAAAATTCTATGGAGGTTGGAATCGCTTACTTGAGAAGTTGCCGGAAGGATGGATTTACTGCGACGCTGACGGATCCCAATTCGATAGTTCATTAACACCATATCTCATTAATGCAGTGTTGAGTATTCGACTACAGTTCATGGAAGATTGGAACATAGGAGCGCAAATGCTTAAAAACCTTTATACTGAGATTGTTTATACACCAATTGCAACGCCAGATGGATCTATCGTGAAGAAATTCAAAGGAAACAATAGTGGACAACCTTCTACGGTAGTAGACAACACATTGATGGTTATAATAGCTTTCAATTATGCTATGCTATCGAGTGGTATTGAAGAAGAAGAGATTGATAATTGCTGTAGAATGTTTGCGAATGGTGATGATTTACTCCTAGCAGTGCATCCTGATTTCGAGTTCATTCTAGATGGATTTCAAAAACATTTTGGGAATCTTGGGCTGAACTTTGAATTCACATCACGAACACGAAACAAATCCGAACTGTGGTTCATGTCCACAAGAGGCATCAAGTATGAAGGAATTTATATACCAAAGCTTGAGAAAGAAAGAATAGTCGCCATACTTGAGTGGGACAGATCAAACTTGCCTGAACATAGGCTAGAAGCTATATGTGCAGCGATGGTTGAGGCCTGGGGATATTCTGATCTTATTCATGAAATACGAAAATTCTATGCGTGGCTTCTAGAGATGCAACCCTTTGCAAATCTTGCAAAAGAAGGGTTGGCTCCATACATCGCTGAGACAGCACTCCGCAATCTGTATCTTGGAACGGGCATCAAAGAGGAAGAAATTGAAAAATACTTTAAACAATTTGTTAAGGATCTTCCTGGATACATAGAAGATTACAATGAAGATGTAATCCATCAGTCAGGAACTGTTGATGCAGGTGCACAAGGCGGCAGTGGAAACCAAGGAACAACACCACCAGCAACA---------------------------------------------------GGTGGTGGCAC----AGGAGCA--------------------------------------------------------------------------------------------------------------------------------------------------------------------------------------------------------------------------------------------------------------------------------------------------------------------------------------------------------------------------------------------------------------------------------------------------------------------------------------------------------------------------------------------------------------------------------------------------------------------------------------------------------------------------------------------------------------------------------------------------------------------------------------------------------------------------------------------------------------------------------------------------------------------------------------------------------------------------------------------------------------------------------------------------------------------------------------------------------------------------------------------------------------------------------------------------------------------------

>MH093718

------------------------ACAACAAAACTCAACACAACACAACAAAACACAACCAAACAAAACCAAGTTACTTTTGCTCAGATTGTAGTGAACGGCTCGGTGGGAAAGGTTCCTCGAGATCACTCTCTGACTCTTCTCTC----------TCAACCAACTTCATTCAAGCGAGATGGCGGGCTCTTGGACTCACGTGACATACAAGTGGCAACCAGATGTCAACAACACACGTGACGTGAAAAGAGTGATGGAGATGTTTGTAGCGAAACATCAACGTTACACTGAGGAACAAAGGCTTGCTCACAACAGCAAGCTGTTAAGGAAGGCTTGTGTTACTAGTGCTGAGTTTACTGAGTCAGCACAGAAACCAAAATGTCATCAAACATGGGTTGAAAAGTGTGACCACAACCCCACAGAGCACTTTGTTTATCAACGTTTC---ACACCTGAGAAGAAAGTGCCAGCCACCAAACCTGAGACAACTTCTGTCACGAAGTTAATCAGGGATATCCTTGAGATTTCGAAGGGCAGTGGGATAAAAATTGAATTAATTGACAAGCGTGTCAAACGTAAAACTCAATTATCCATAAGACGACACAATGGTAAAGATTTCTTGCACTGCAAAACCAGGCATGAAAATGGCCTGTTTAAACGCAAGGACGTTGACATTAGTGTCAAGTGGTTGTCCACCATTGAAGCCATTGCAAAATGCTACAGCACTGTGAATGCAGAGGAATTGCAAAGTCTCAGTAGAGGCAGTAGTGGTCTAACATTCATGCAGAATGATGAATTATTCATCGTGCGTGGACGGATGCATGGTGAGATTGTCAATAGTTTGCATGAGAATAAGCACGTAATGGAAATCGAACACTATGCTGATCCACAGGCGAACAGTTTCTGGAAAGGCTACACAGATGCGTATGTCGAGAACAGAAACATATCCACTACTCACACAGAGCACACACCAACCATCAATTTAGAAGAGTGTGGCAAGAGAATGGCACTGTTAGAAATCCTATTCCATTCAACTTTTAAAATAACATGCAAGACATGTAATATTGATGATCTTGAATTATCAGATGATGAATTTGGGGCCAAGTTATATAGTAATCTGCAGCGCATTGAAGAAAAACAACGTGAATATCTTGCTAAAGATCAAAAACTTTTACGCATGATTCATTTTGTAAAGGATCGATGTAATCCAAAGTTTTCACATTTACCCTTGCTATGGCAAGTGGCAGAAACAGTGGGGCATTACACTGATAACCAATCGAAGCAGATAATTGATATCAGTGAGGCGCTCATAAAAGTTAATACCTTAACTCCTGATGATGCAGTAAAGGCCAGTGTGGCATTATTGGAAGTAGCACGGTGGTATAAGAATCGGAAGGAATCACTTAAAACGGACACATTGGATTCATTTCGAAACAAGATTTCACCAAAGAGCACGATCAACGCAGCATTAATGTGTGATAACCAGTTAGATAAGAATGCAAATTTCGTGTGGGGAAACAGAGAATACCACGCAAAGCGATTCTTCGCTAACTATTTTGAAGCTGTGGACCCAACTGATGCATATGAAAAACATGTAACACGCTTCAACCCTAATGGACAGCGGAAATTATCAATTGGCAAACTAGTAATCCCATTAGATTTCCAGAAAATCAGAGACTCGTTCGTTGGCCTATCAATAAATAAACAATCACTGAGCAAAGCTTGCGTGAGTAAAATCGATGGAGGTTACGTGTATCCATGTTGCTGCGTTACAACAGAATTTGGAAAACCAGCATATTCTGAGATAATACCTCCAACGAAAGGACATATCACGATTGGAAATTCAGTGGACCCGAAAATAGTGGATCTACCAAATACAACACCACCGAGTATGTACATTGCAAAAGATGGATACTGTTATATTAACATATTCTTGGCAGCAATGATAAACGTCAACGAGGAATCCGCAAAAGATTACACTAAGTTTCTTAGAGACGAGTTGGTGGAACGGCTTGGTAAATGGCCAAAATTGAAAGATGTGGCCACAGCATGCTATGCTTTGTCAGTGATGTTCCCAGAGATAAAGAATGCCGAGTTACCACCAATACTAGTGGATCATGAGAGTAAGTCAATGCATGTCATTGATTCATATGGATCACTCAGCGTTGGCTTTCACATTCTAAAGGCAAGTACTGTTGGACAACTGATAAAATTTCAGTATGAGTCATTGGAAAGTGAGATGCGCGAGTACATAGTGGGTGGCACTTTGACACAGCAAACTTTCAGCACACTTCTTAAGACTCTCACAAAGAACATGTTTAAACCAGACAAAATAAAGCAGATAATTGAGGAAGAGCCATTCTTATTAATGATGGCAATTGCATCCCCAACTGTACTCATCTCGCTGTACAACAACTGCTACATCGAGCAAGCGATGACATATTGGATCGTCAAGAACCAAGGCATCGCAGCGCTTTTTGCGCAGTTGGAGGCACTAGCAAAGAAAACTTCTCAAGCGGAGCTACTAGTTCTTCAAATGCAAATACTTGAGAAAGCTTCGAACCAACTGAGACTTGCAGTCACGGGACTTAATCATGTTGATCCAGCTAAACGACTTTTATGGTCTCACCTGGAAGCTATGACAACACGGTCAGAGATGAATAAGGAACTCATAGCGGAAGGCTATGCACTGTACGACGAGCGCTTATACACTTTAATGGAAAAAAGTTACGTAGATCAATTAAACCAATCATGGGCAGAATTATCATACTGTGGAAAATTTTCAGCAATATGGCGTGTGTTCAGAGTCAGGAAATACTACAAACCATCTTTAACCGTGAGAAAAAGCGTAGATTTAGGCGCTGTGTACAATATATCAGCTACGCATCTAATATCAAATTTAGTGCAGAAAAGTCGAGATCAAGTCAGCTCTACTTTAACCAAACTCCGCAACGGTTTCTATGATAAAATGGAGAGAGCGAGAGTTAGTGCAGTAAGGACAGTATATTGGTTCGTACCCGACATATTTAGACTAATACATATTTGCTTAGTTTTAAGTATATTAACAACTATAGCCAATACAATAATCGCAACTATGAATGATTATAAAAAGTTGAAAAAGCAACAAAGAGAAGACGAATACGAAGCCGAGATTAACGAGGTGCGAAGGATACACGCCAACCTAATGAAGGAGCATAATGATGACTTAACATGTGAACAATTTATTGAGCACATACGACAGACACATCCACGCCTCATTGAGGCAACATTGGATTTAACACATACAGGTGTCATCCATGAGGGCAAATCCAATTTAGAAACAAACCTCGAACAGGCAATGGCAGTGGGAACTTTACTCACTATGATACTCGATCCACAGAAGAGTGATGCAGTTTATAAGGTTCTCAATAAGATGCGAACAGTTATCAGCACAATAGAACAGAATGTACCATTTCCCTCAGTGAATTTCACGAGCATCTTGTCACCTCCTGTAACTCAGCAAAGTGTAGATGTTGACGAACCGTTGACACTGAGTACCGATAAGAATTTGACTATAGATTTCGACACAAATCAAGATTTGCCAGCGGACACATTTAGCAATGACGTTACGTTCGAGAACTGGTGGGCTAATCAGATAAACAACAACAGAACAGTGCCACACTATCGACTTGGGGGAAAGTTTGTAGAATTCACAAGAGAGAATGCAGCAATGGTTAGCATTGAGCTCGCCCATTCGAACATCGAAAAAGAGTTTCTACTCAGAGGAGCCGTTGGGTCAGGAAAATCCACAGGTTTGCCATATCATCTCAGTATGCGTGGAAAAGTGTTATTGATAGAACCTACTCGACCATTAGCTGAGAACGTTTGCAGACAACTGCAAGGTCCTCCATTTAATGTGAGCCCCACTTTACAAATGAGAGGATTGAGCACATTTGGCTGCACTCCTATCACGATAATGACATCTGGCTTCGCATTGCACATGTATGCTAATAACCCCGATAAGATCTCTGAGTATGACTTCATTATCTTTGATGAATGTCACATTATGGAAGCACCTGCAATGGCATTCTATTGTTTGCTTAAGGAGTATGAATATCGAGGCAAGATAATAAAAGTTTCAGCTACACCACCAGGACGAGAATGCGAGTTTTCAACCCAACATCCAGTAGATATACATGTATGTGAAAGCTTGACACAACAGCAATTCATCATGGAATTAGGAACAGGATCAACTGCTGACGCAACCAAATATGGCAATAACATATTAGTGTACGTTGCAAGTTATAATGATGTAGATTCTTTATCCCATGCTCTAACTGAACTTAAATATTCAGTGATTAAAGTCGATGGAAGAACGATGAAGCAGAACACCACAGGAATCGTAACAAATGGAACATCCGGTAAGAAATGCTTCGTTGTGGCCACAAATATTATTGAAAACGGTGTAACGCTAGATGTCGACGTTGTCGTCGACTTTGGACTTAAAGTAACAGCTGAATTAGATGTTGATAACAGGGCAATAATGTATAAACGTGTGAGCATATCTTATGGCGAGCGCATTCAGAGACTCGGAAGAGTCGGAAGGAATAAGCCTGGGACAGTTATCCGCATCGGAAAAACAATGAAAGGCTTACAAGAAATTCCTGCGATGATTGCTACTGAAGCAGCTTTCATGTGTTTTGCATATGGACTGAAAGTTATAACACATAATGTATCAACAACACATCTAGCAAAATGCACTGTCAAACAAGCTAGAACTATGATGCAATTCGAACTATCACCATTTGTAATGGCTGAATTAGTTAAATTCGATGGTTCCATGCATCCACAGATTCATGAAGCGTTAACCAAGTATAAATTGAGGGATTCTGTGATCATGTTAAGACCAAACGCAATACCAAAGGTTAACCTTCACAACTGGTTAACGGCCCGTGATTACAATAGGATAGGCTGCTCACTGGAACTTGAGGATCACGTTAAAATACCATATTATATACGAGGAGTTCCTGACAAGTTGTATGGGAAGTTATATGATATAATCCTTCAATACAGCCCTACAAGTTGTTATGGAAGATTGTCAAGTGCTTGCGTGGGTAAGGTCGCATATACATTGCGCACTGATCCTTGTTCATTACCGAGAACAATAGCTATAATCAACGCACTGATTACTGAAGAGTATGCAAAGAGGGATCACTACAGAAACATGATAGCAAACCCCTCATCATCGCACGCCTTCTCACTTAATGGGCTGGTATCCATGATCGCGTCTCGGTACATGAAAGATCACACGAAGGAGAACATAGATAAGCTTGTAAGAGTGCGTGACCAGCTACTTGAGTTCCAAGGCACAGGTATGCAATTTCAAGATCCTTCAGAATTGATGGATATTGGTGCATTAAATACAGTTATCCACCAAGGAATGGACGCCACGGCTGCTTGCATTGGATTACAAGGGCGTTGGAATGCTTCACTCATCCAGCGCGATTTGATGATATCAGCAGGGATCTTCACAGGAGGAATTCTTATGATGTGGTGTCTCTTTACAAAATGGAGCAAGACAGAAGTATCACATCAAGGAAAGAACAAACGCAGTAGACAAAAACTCCGATTCAAAGAAGCGAGAGACAACAAATATGCATATGATGTCACAGGATCGGAAGAGTGCCTTGGTGAAAATTTTGGAACAGCCTATACAAAGAAAGGTAAAGGAAAAGGAACTAAAGTTGGACTCGGTGTGAAGCAACACAAATTTCATATGATGTATGGTTTTGATCCTCAAGAGTACAACCTAATTCGATTTGTCGATCCACTCACAGGAGCAACTCTTGATGAGCAAATCCATGCCGACATACGCTTAGTTCAAGAGCATTTCGCTGAAATTCGTGAGGAGGCAGTAGCTAATGACACAATTGAAAAGCAGCATATCTACGGCAATCCTGGACTACAAGCATTTTTCATACAAAATGGTTCAGCAAACGCTCTGAGAGTTGATTTAACGCCGCATTCACCTACACGAGTTGTCACGGGTAATAATATAGCAGGATTCCCAGAGCACGAGGGTACACTTCGTCAAACTGGAACAGCAATAACCATACCCATTGGTCAAGTCCCAATCGCACGTGAAACAGGAGTTGCACACGAGTCAAAATCCATGATGAATGGGCTGGGTGACTACACGCCAATATCGCAACAGTTATGTTTAGTACAAAATGACTCAGATGGGGTAAAGCGGAATGTATTTTCAATTGGATATGGTTCATATCTCATTTCACCAGCGCACTTATTCAAATATAACAATGGCGAAATAACAATTAGATCATCAAGAGGATTGTATAAAATTCGTAATTCTGTGGATTTAAAACTACACCCAATTGCACACAGAGACATGGTCATAATTCAGCTTCCCAAGGATTTCCCACCGTTCCCAATGCGTTTAAAATTCACACAACCATCACGAGATATGAGAGTCTGCTTAGTGGGAGTCAATTTCCAACAGAATTATAGCACTTGCATCGTATCAGAAAGTAGTGTGACAGCACCAAAAGGAAATGGAGACTTTTGGAAACATTGGATATCAACAGTCGACGGTCAATGTGGACTACCATTGGTAGACACTAAGAATAAACACATTGTCGGAATTCATAGTCTTGCATCTACAAGTGGAAACACCAACTTCTTTGTCGCTATGCCTGAGAACTTTAACAATTACATCAATGAACTTGTGCAAGCGAATAAATGGGAAAAGGGATGGCACTATAATCCGAATCTCATATCTTGGTGTGGATTGAACTTAGTCGACTCCGCTCCAAAGGGTTTGTTTAAAACGTCAAAATTAGTTGAAGATTTGGATGCTAGTGTTGAAGAGCAGTGCAAAGTTACTGAAACATGGCTCACAGAGCAATTACAAGATAACTTGCAAGTGGTCGCAAAATGCCCAGGCCAACTCGTTACTAAGCATGTCGTCAAAGGCCAATGCCCACATTTCCAGTTATATTTGTCAACACATAATGATGCCAAAGAATATTTCGCACCCCTGCTTGGAAAATATGATAAGAGTAGACTCAATAGAGCGGCATTTATCAAAGACATATCAAAGTATGCAAAACCAATTTATATTGGAGAAATCAATTATGATATCTTTGATAGAGCTGTACAACGAGTCATTAATATTCTTAAAAATGTTGGAATGCAACAATGCGTTTATGTTACAGACGAAGAAGAAATTTTTAAATCACTCAACTTAAACGCAGCTGTCGGAGCACTGTACACAGGAAAGAAGAAAGATTACTTTGAAAGTTTTTCAAATGAAGACAAGGAAGAAATCGTGATGAGATCATGTGAACGCATTTACAATGGACAACTTGGTGTATGGAATGGGTCACTCAAAGCCGAGATCAGACCAATAGAGAAAACCATGTTAAACAAGACTCGAACCTTTACAGCAGCTCCATTGGAAACTTTGCTTGGAGGAAAAGTGTGTGTGGACGATTTTAATAATCAATTTTATTCGCACCATTTAGAAGGCCCATGGACTGTTGGGATAACAAAATTCTATGGAGGTTGGAATCGCTTACTTGAGAAGTTGCCGGAAGGATGGGTTTACTGTGACGCTGACGGATCCCAATTCGACAGTTCATTAACACCATATCTTATTAATGCAGTGTTGAATATTCGATTACAGTTCATGGAAGATTGGAACATAGGAGCACAAATGCTTAAGAATCTTTATACTGAGATTGTTTATACACCAATTGCAACGCCAGATGGATCCATCGTGAAGAAATTCAAAGGAAACAATAGCGGACAACCTTCTACAGTAGTTGATAATACATTGATGGTTATAATAGCTTTCAACTATGCCATGCTATCGAGCGGTATTAGGGAAGAAGAGATTGATAACTGTTGTAGGATGTTTGCAAATGGTGATGACTTGCTCCTAGCAGTACATCCTGATTTTGAATACATTCTAAACGGATTTCAAGATCACTTCGGAAATCTTGGATTGAATTTTGAGTTTACATCACGAACACGGGATAAATCCGAACTATGGTTTATGTCTACAAGAGGAATCAAATGTGAAGGAATCTACATACCGAAACTCGAGAAAGAAAGAATAGTCGCAATACTCGAATGGGATCGATCAAATCTACCTGAGCATAGGTTAGAAGCCATTTGTGCAGCTATGGTTGAAGCATGGGGCTACTCAGATCTTGTTCACGAAATTCGGAAATTTTATGCGTGGCTTCTAGAAATGCAACCTTTCGCAAATCTAGCAAAAGAAGGCATGGCGCCATACATAGCAGAAACAGCGCTCCGTAACCTTTATCTTGGAACGGGTATTAAAGAAGAAGAAATCGAGAAATATTTTAAGCAATTTGTTAAGGATCTTCCTGGATACATAGAAGATTACAATGAAGAGGTCATTCACCAATCGGGAACAGTTGATGCAGGTGCACAAGGCGGCAGCGGAAGCCAAGGAACAACACCACCAGCAACAGGTAGCGGAGCACGACCAGCAACTTCAGGAG------------CAGGATCTGGTAGCGGAAC----AGGGACTGGAGCCGGTGCAACTGGAGGCCAAACAGGG---------GCT---G---GCAGTGGTGCTGGGACAGGATCTGGAGCGGCCGGAGGCCAATCAGGATCTGGAAGTGGTGCTGGACAGACTGGCACAGGCTC---AGCAGGAACTGGTGCA------------ACGGGAGGTCAAAGAGATAAGGATGTAGATGCAGGTACAACAGGAAAAATTTCTGTACCAAAGCTCAAGGCCATGTCAAAGAAAATGCGCTTACCGAAAGCAAAAGGAAAAGATGTCTTGCATCTGGACTTCTTGCTTACATACAAGCCACAGCAACAAGACATATCAAACACAAGAGCAACTAAGGAAGAGTTTGATAGATGGTACGATGCCATAAAGAAAGAATACGAGATTGATGACACACAAATGACAGTCGTCATGAGCGGTCTTATGGTGTGGTGCATTGAGAATGGTTGCTCACCAAACATAAACGGAAATTGGACAATGATGGATGGAGATGAACAAAGAGTCTTTCCACTCAAACCAGTCATTGAAAACGCATCTCCAACTTTCCGACAAATTATGCATCATTTTAGTGATGCAGCTGAAGCGTACATAGAGTACAGAAACTCTACTGAGCGATACATGCCAAGATACGGACTTCAGCGCAATCTCACCGACTATAGCTTAGCACGGTATGCATTTGATTTCTATGAAATGACTTCACGCACACCTGCTAGAGCTAAAGAAGCCCACATGCAG---ATGAAAGCCGCAGCAGTTCGTGGTTCAAACACACGACTGTTCGGTCTGGACGGAAATGTCGGCGAGACTCAGGAGAATACAGAGAGACACACAGCTGGCGACGTTAGTCGCAATATGCACTCTCTGTTGGGAGTGCAGCAGCACCACTAGTCTCCTGGAAACCCTGTTTGCAGTACCTATAATATGTACTA------ATATATAGTATGTCAGTGAGGTTTTACCTC-----------------------GTCTTTACTA-TTTGTTATGTATGTATTTAAAGCGTGAACCAGTCTGCAGCATACAGGGTTGGACCCAGTGTGTTCTGGTGTAGCGTGTACTAGCGTCGAGCCATGAGATGGACTGCACTGGGTGTGGCTTTGCCACTTGTGTTGCGAGTCTCCTGGTAAGAGACAAAAAAAAAAAAAAA-

>MH093720

-----------------------------------CAACACAACACAACAAAACACAACCAAGCAAATCCAATTTACTTGCGCTCAGATTGTAGTGAACGGCTCGAACGAAACGGTTCTTCGAGATCACTCTCTGATTCTTCCTCA----------TCTTTCAATTCCTTTCGAAAGAAATGGCGGGAACGTGGACCCATGTGACACACAAGTGGCAACCAGATGTCAACAATGATCGTCACATTAAGAGAGTAATGGAAATGTTCGCAGCAAAACATCAACATTACTCAGAAGAACAGCGACTTGCTCACAATATGAAATTATTGAGGAAGGCAAGTGTTGTGAGCGTTGAGCCTGCAAAACCAAAGCAGAAGCAGGCAACTCAACAGATGTGGGTTGAGAAATGTGATCACAATCCTGTTGATCACTTAGTATATCCACGACTTGAAAGACCTATCAACAAAGTG---GAAACGAGTATCAAAAGTGCATCTGTAAGCAAGCTAACCAGAGAGATCTTGGAGATCTCGAAGGTAAGCGGCCTTAAAGTTGAACTAATTGATAAGCGGAAGAGATTTAAAACGCAGTTGTCAATCAAAAAGTTCAATGGAAAGAATTTCCTCCACTGCAAAACAAATCATGAAAACAATCTATTCAAGAGGAGGGACATAGCCATCGGACATAAATGGCTTCCGACAATTGAAGCTATTGCTCGATGCTACAGCACAATGAACCGAGAGGAGCTGCAGAGTCTTTATAGGGGAAGCAGCGGTCTCACATTCATCCAAAATAATGAATTATTCATTGTTAGAGGGAGGATGAATGGCGAACTTGTCAATAGCTTGCACGAAACAAACCGAGTTATGGATATTGAACACTATGCAGATCCCCAAGCTAATGACTTTTGGAGGGGATACACAGATGCTTACGTGGATAATCGTAGTATTTCTACCACCCACACAGAGCATACCCCGACAATCAATCTAGAAGAGTGTGGAAAACGAATGGCTCTACTTGAGGTATTATTCCACTCCACATTCAAGATCACATGTAAAACATGCAACATTGATGATCTTGAATTATCAGATGATGAATTTGGAGCTAAACTCTATAAAAATCTGCAACGCATCGAGGAGAAGCAACGAGAGTACCTTGCTAAGGATCAAAAGTTATCCAGAATGATACAATTTATCAAAGAGAGATGCAACCCAAAATTCTCACATTTACCAATGTTGTGGCAAGTTGCAGAAACAATAGGACACTACACTGATAATCAGTCTAAGCAAATAATGGATATTAGCGAAGCGCTCATCAAAGTTAACACTTTAACTCCTGATGATGCAATGAAAGCGAGCGCAGCATTACTCGAAGTGTCGCGATGGTATAAGAATCGTAAGGAATCACTCAAAACCGACTCATTGGAATCTTTTAGAAATAAAATATCACCAAAGAGTACAATAAATGCAGCTTTAATGTGCGACAATCAATTGGACAAAAATGCAAATTTTGTATGGGGTAATAGGGAATACCATGCCAAACGATTCTTTGCAAACTATTTTGAAGCAGTGGATCCCACAGACGCATATGAAAAGCACGTCACACGGTTTAACCCTAATGGTCAACGAAAGTTATCAATAGGAAAGTTAGTTATTCCACTAGACTTTCAAAAGATTAGAGAATCATTTGTTGGACTTCCGATTAACAGACAACCGCTAGACAAATGTTGCGTTAGTAAGATCGAAGGAGGATATATATACCCATGCTGCTGCGTCACAACAGAATTTGGTAAACCAGCATACTCCGAGATAATACCTCCGACAAAAGGCCACATAACAATAGGCAATTCAATTGATCCAAAGATTGTGGACCTGCCAAATACAACACCACCCAGCATGTACATTGCTAAGGACGGGTACTGCTATATCAATATCTTTCTAGCAGCCATGATCAACGTCAATGAAGAATCTGCCAAGGATTACACGAAATTTTTGAGAGATGAACTAGTTGAACGTCTCGGAAAGTGGCCAAGGCTTAAAGACGTAGCAACAGCGTGTTATGCATTGTCTGTAATGTTTCCAGAAATTAAGAATGCTGAGCTACCTCCAATTTTAGTTGATCATGAAAATAAATCAATGCATGTCATTGATTCATATGGTTCACTAAGCGTTGGATTTCACATATTGAAAGCAAGCACGATTGGTCAATTAATCAAATTTCAATATGAATCTATGGATAGTGAGATGCGCGAATACATAGTAGGAGGAACTCTCACGCAACAGACATTCAACACACTTCTTAAGATGCTCACAAAGAACATGTTCAAACCAGAGCGCATCAAACAGATAATTGAGGAGGAACCCTTCTTGCTCATGATGGCAATTGCATCTCCAACGGTATTAATAGCACTATATAATAATTGTTATATCGAGCAAGCTATGACATACTGGATCGTGAAGAATCAAGGAGTTGCAGCCATATTCGCACAACTCGAAGCATTAGCCAAGAAAACATCCCAGGCTGAATTACTAGTTCTACAAATGCAGATACTTGAAAAAGCATCTAGTCAACTAAGATTAGCAGTTTCAGGACTTAGCCATGTCGACCCAGCAAAGCGACTTTTGTGGTCACACCTCGAAGCAATGACAACACGGTCAGAAATGAACAAGGAATTGATAGCTGAGGGATATGCACTATACGACGAGCGTCTTTATACCCTGATGGAAAAAAGTTACGTAGATCAATTAAACCAGTCATGGGCAGAATTATCATACTGTGGAAAATTTTCAGCAATATGGCGTGTGTTCAAAGTCAGGAAGTATTACAAACCGTCTTTAACCGTGAGAAAAAGCGTAGATTTAGGCGCTGTATACAATATATCAGCTACGCATCTAATATCAGATTTAGTGCGGAAAAGTCAAGATCAAGTCAGCTCTATTTTAACCAAACTCCGCAACGGTTTCTATGATAAATTAGAGAAAGCTAGAATACGTACTATAAAAACGGTTTACTGGTTTATACCTGATATATTTAGACTTATGCATATATTCATAGTTTTGAGTTTATTAACTACCGTAGCTAACACTATCATAGTAACTATGAATGATTACAAGAAATTGAAGAAACAACAAAGAGAAGATGAATATGAAGCGGAAATTAACGAAGTTCGCAAAATCCATTCCACCTTGATGGAAAAGCGGAAGGACAATTTGACGTGTGAACAATTTGTTGAATTTATGCGTCAAAATCATCCGCGGTTAGTTGAAGCAACACTGGACTTAACCCACACAGGTGTCATACATGAAGGAAAATCTAATCTCGAAACCAATTTGGAACAGGCAATGGCAATTGGAACCTTGATAACCATGATACTTGATCCACAGAAAAGCGATGCAGTCTACAAGGTGTTGAATAAAATGCGGACAGTAATTAGTACAATTGAACAGAACGTTCCATTCCCTTCAGTGAACTTCTCCAACATCTTAACACCTCCAGTGACACAACAGAGCGTAGATGTTGACGAACCATTGACACTTAGCACTGAGAAAAATTTAACAATAGACTTTGACACGAATCAAGATTTACCTGCCGATACATTCAGTAATGATGTGACATTTGAAGATTGGTGGTCAAATCAATTAAGCAACAACAGAACAGTGCCACATTACCGACTTGGGGGAAAGTTTGTTGAATTCACACGAGAAAACGCAGCCCACACGAGCATCGAACTTGCACACTCAAACATTGAGAAGGAGTTCTTGCTTAGAGGAGCAGTCGGATCAGGAAAATCCACTGGGTTGCCATACCATCTTAGCATGCGTGGAAAAGTGCTTTTGTTAGAGCCTACAAGGCCGCTAGCTGAGAACGTATGTAGGCAATTACAAGGACCACCATTTAATGTAAGTCCAACTCTCCAAATGCGTGGACTGAGTTCTTTTGGGTGTACTCCAATCACGATCATGACATCAGGTTTTGCATTGCACATGTACGCAAACAATCCAGATAAAATATCTGAATACGATTTTATAATCTTCGATGAGTGTCATATAATGGAAGCACCAGCAATGGCCTTTTATTGCTTACTCAAAGAATACGAATACCGAGGGAAAATCATCAAGGTATCAGCCACGCCTCCAGGAAGGGAGTGCGAATTCACAACACAACATCCAGTAGACATCCATGTTTGTGAAAATCTAACTCAGCAACAATTTGTTATGGAACTCGGAACCGGTTCAACCGCAGATGCTACGAAGTACGGAAATAATATCTTAGTTTATGTAGCAAGTTATAATGACGTCGATTCATTGTCGCATGCATTAGTTGAACTCAAATATTCCGTCATTAAAGTAGATGGCCGAACAATGAAGCAAAACACAACAGGAATAATAACAAACGGCACCTCACAAAAGAAGTGTTTTGTTGTCGCAACAAATATAATTGAAAATGGCGTCACACTAGATATCGATGTTGTTGTTGACTTCGGACTGAAAGTCTCAGCCGATTTGGATGTTGACAATAGGGCAATATTGTATAAACGCGTGAGTATATCATATGGTGAACGCATACAACGACTGGGTCGTGTTGGAAGAAATAAACCTGGCACAGTTATTCGTATTGGAAAAACCATGAAAGGTTTGCAGGAGATTCCAGCAATGATCGCAACAGAAGCAGCCTTCATGTGTTTCGCATATGGTCTTAAAGTCATCACCCATAATGTTTCAACAACCCATCTTGCAAAGTGCACAGTTAAACAAGCAAGAACCATGATGCAATTTGAATTATCACCATTTGTCATGGCTGAGCTAGTTAAGTTTGATGGTTCAATGCATCCGCAAATACATGAGGCTTTAGTAAAATACAAACTTAGAGATTCTGTCATAATGCTCAGACCGAATGCAATTCCAAAAGTTAATTTGCACAATTGGCTTACAGCCCGGGATTATAATAGAATAGGTTGCTCATTGGAACTTGAAGATCATGTTAAAATTCCGTACTATATTAGGGGAGTTCCTGATAAGCTGTATGGAAAGCTATATGATATTATTTTGCAGTATAGTCCAACTAGTTGTTATGGTAGACTATCAAGTGCATGTGCAGGTAAAGTAGCATACACTTTGCGAACTGATCCGTGTTCACTTCCAAGAACAATAGCAATAATTAATGCTTTAATCACGGAGGAATATGCGAAGAGAGATCACTACCGAAACATGATTTCAAACCCCTCTTCATCACATGCATTCTCACTCAATGGGTTGGTTTCCATGATCGCCACTAGATATATGAAGGACCACACAAAAGAGAATATTGATAAACTCATTAGAGTACGTGATCAATTACTTGAGTTTCAAGGTACTGGAATGCAATTTCAAGATCCATCAGAACTCATGGAAATTGGGGCTCTCAACACAGTCATTCATCAAGGAATGGATGCGACAGCAGCTTGCATTGGTTTGCAAGGACGATGGAATGCCTCACTTATACAACGTGATCTTCTAATTGCAGGAGGAGTGTTCATCGGAGGCATTCTAATGATGTGGAGCTTATTTACTAAATGGAGTAACACAGATGTCTCACATCAAGGAAAGAACAAGCGCAGCAGACAGAAGCTCCGATTCAAAGAAGCAAGAGACAACAAATATGCATATGATGTTACAGGATCGGAGGAATGCCTTGGTGAGAATTTCGGAACAGCCTACACGAAGAAAGGTAAAGGGAAGGGAACAAAAGTTGGACTCGGTGTTAAACAACATAAATTTCATATGATGTATGGTTTTGATCCTCAAGAGTACAATTTAATTCGATTTGTCGATCCACTCACAGGAGCAACTCTTGATGAGCAAATTCATGCCGACATACGATTGATTCAAGAGCATTTCGCTGAAATTCGTGAGGAGGCAGTAGCTAACGACACAATTGAAAGGCAGCAGATCTACGGCAATCCTGGACTACAAGCATTTTTCATACAGAATGGGTCAGCAAATGCTCTGAGAGTTGATTTAACACCACATTCACCTACACGGGTTGTCACAGGCAATAACATAGCAGGATTCCCAGAATACGAAGGTACACTCCGTCAAACTGGAACAGCTATAGCCATACCCATTGGTCAAGTCCCAATTGCAAATGAAGCAGGAGTCGCACACGAATCAAAATCTATGATGAATGGATTGGGTGATTACACGCCAATATCGCAACAATTGTGTTTAGTGCAAAATGACTCAGATGGAGTAAAACGGAATGTATTTTCAATTGGTTATGGCTCATATCTCATTTCACCAGCGCACTTATTTAAATATAACAATGGCGAAATTACAATTAGATCATCAAGAGGATTGTACAAAATTCGCAACTCTGTGGATTTAAAATTACATCCGATTGCACACAGAGACATGGTCATAATCCAACTCCCAAAGGATTTCCCGCCGTTCCCAATGCGTTTGAAATTCACACAACCATCACGAGAGATGCGAGTTTGCTTAGTGGGAGTTAATTTCCAACAGAACTATAGCACTTGCATCGTATCGGAAAGCAGCGTAACAGCACCAAAGGGGAATGGAGATTTTTGGAAGCATTGGATATCAACAGTCGACGGTCAATGTGGACTACCATTGGTAGATACTAAGAACAAACACATTGTTGGAATTCATAGTCTTGCATCTACAAGTGGAAACACCAATTTCTTTGTTGCCATGCCTGAGAACTTTAATGAATACATTAATGGACTTGTGCAAACAAACAAATGGGAAAAGGGATGGCACTATAATCCGAATCTCATATCTTGGTGTGGATTAAACTTAGTCGACTCTGCTCCAAAGGGTTTATTTAAAACGTCAAAATTAGTTGAAGATCTGGATGCTAGTGTTGAAGAGCAGTGCAAAGTTACTGAAACATGGCTCACAGAGCAATTACAAGACAATTTGCAAGTGGTCGCGAAATGCCCAGGCCAACTCGTCACTAAGCATGTCGTCAAAGGCCAATGCCCGCACTTTCAGTTATATTTATCAACACATAATGATGCCAAAGAATATTTCGCACCCCTGCTTGGAAAATATGATAAGAGCAGACTCAATAGAGCGGCATTTATCAAAGACATATCAAAGTATGCAAAACCAATTTATATTGGAGAAATCAATTATGATATCTTTGATAGAGCTGTACAACGAGTTATTAATATCCTTAAAAATGTTGGAATGCAACAATGCGTTTATGTCACAGATGAAGAAGAGATTTTTAAATCACTTAACCTAAACGCAGCTGTCGGAGCACTGTACACAGGAAAGAAGAAAGATTACTTTGAAAGTTTTTCAAATGAAGACAAGGAAGAAATCGTGATGAGATCATGTGAACGCATTTATAATGGACAACTTGGCGTGTGGAATGGGTCACTCAAAGCTGAAATCAGACCAATAGAGAAAACCATGTTAAATAAGACTCGAACTTTTACAGCAGCTCCATTGGAGACTTTGCTTGGAGGAAAAGTGTGCGTGGACGATTTTAATAATCAATTTTATTCACACCATTTAGAAGGCCCATGGACTGTTGGGATAACAAAATTTTATGGAGGTTGGAATCGCTTACTCGAGAAGTTGCCAGAAGGATGGGTTTACTGCGACGCCGACGGATCCCAATTCGATAGTTCATTAACACCATATCTCATTAATGCAGTGTTGAATATTCGATTACAGTTCATGGAAGATTGGAATATAGGAGCGCAAATGCTCAAAAACCTTTATACTGAGATTGTTTATACACCAATTGCAACGCCAGATGGATCTATCGTGAAGAAATTCAAAGGAAACAATAGTGGACAACCTTCTACAGTAGTTGATAATACATTGATGGTTATAATAGCTTTCAACTATGCAATGCTATCGAGCGGTATTAAAGAAGAAGAGATTGATAACTGCTGTAGGATGTTTGCAAATGGTGATGACCTGCTCCTAGCAGTACATCCTGATTTTGAATACATTCTAGACGGATTTCAAGACCACTTCGGAAATCTCGGATTGAATTTTGAGTTTACATCACGAACACGAGATAAATCCGAACTGTGGTTTATGTCTACAAGAGGAATCAAACGTGAAGGAATCTACATACCTAAACTCGAGAAAGAAAGAATAGTCGCAATACTCGAATGGGATCGATCAAATCTACCTGAGCATAGATTGGAAGCCATTTGCGCAGCTATGGTTGAAGCATGGGGCTACTCAGATCTTGTTCACGAAATTCGGAAATTTTATGCGTGGCTTCTAGAAATGCAACCTTTCGCAAATCTAGCAAAAGAAGGCATGGCACCATACATAGCAGAAACAGCGCTTCGTAACCTTTATCTTGGAACGGGCATTAAAGAAGAAGAAATCGAGAAATATTTTAAGCAATTTGTTAAGGATCTTCCTGGATACATAGAAGATTACAACGAAGAGGTCATCCACCAATCGGGAACAGTTGATGCAGGTGCACAAGGCGGCAGCGGAAATCAAGGAACAACACCGCCAGCAACAGGTGGCGGAGCAAAACCAGCAACTTCAGGAG------------CAGGATCTGGTAGCGGAAC----AGGAACCGGAACCGGTGCAACTGGAGGCCAAACAGGG---------GCT---G---GCAGTGGCGCTGGGGCAGGATCTGGAGCAACCGGAGGCCAATCAGGATCTGGAAGTGGCACTGGACAGACTAACACGGGCTC---AGCAGGAACCGGTGCA------------ACAGGGGGCCAAAGAGATAAGGATGTAGATGCAGGTACAACAGGACAAATGTCTGTACCAAAGCTCAAGGCCATGTCAAAGAAAATGCGCTTACCGAAAGCAAAAGGTAAAGATGTCTTGCATCTGGACTTCTTGCTTACATACAAGCCACAGCAGCAAGACATATCAAACACAAGAGCAACTAAGGAAGAGTTTGATAGATGGTACGATGCCATAAAGAAAGAATACGATATTGATGACACACAAATGACAATTATCATGAGCGGTCTTATGGTGTGGTGCATTGAGAACGGTTGCTCACCAAACATAAACGGAAATTGGACAATGATGGATGGAGATGAACAAAGAGTCTTTCCACTCAAACCAGTTATTGAAAACGCATCTCCAACTTTCCGACAAATTATGCATCATTTTAGTGATGCAGCTGAAGCGTACATAGAGTACAGAAACTCTACTGAGCGATACATGCCAAGATACGGACTTCAGCGCAATCTCACCGACTATAGCTTAGCACGGTATGCATTTGATTTCTATGAAATGACTTCACGCACACCTGCTAGAGCTAAAGAAGCCCACATGCAG---ATGAAAGCCGCAGCAGTTCGTGGTTCAAACACACGACTGTTCGGTCTGGACGGAAATGTCGGCGAGACCCAGGAGAATACAGAGAGACACACAGCTGGCGATGTTAGTCGCAATATGCACTCTCTGTTGGGAGTGCAGCAGCACCACTAGTCTCCTGGAAACCCTGTTTGCAGTACCTATAATATATACTA------ATATATAGTATCTCAGTGAGGTTTTACCTC-----------------------GACTTTACTATTTTATTATGTATGTATTTAAAGCGTGAACCAGTCTGCAGCATACAGGGTTGGACCCAGTGTGTTCTGGTGTAGCGTGTACTAGCGTCGAGCCATGAGATGGACTGCACTGTGTGTGGCTTTGCCACTTGTGTTGCGAGTCTCCTGGTAAGAGACAA--------------

>MH093721

--------------------------------------------------------------------------------------------------------------------------------------------------------------------------------------------------------------------------------------------------------------------------------------------------------------------------------------------------------------------------------------------------------------------------------------------------------------------------------------------------------------------------------------------------------------------------------------------------------------------------------------------------------------------------------------------------------------------------------------------------------------------------------------------------------------------------------------------------------------------------------------------------------------------------------------------------------------------------------------ACCACCCACACAGAGCACACTCCGACAATCAATCTAGAAGAGTGTGGAAAACGAATGGCTCTACTTGAGATATTATTCCACTCCACATTCAAGATTACATGTAAAACATGTAACATGGATGATCTTGAATTATCAGACGATGAATTTGGAGCTAAACTCTACAAAAATCTGCAACGCATCGAAGAGAAGCAACGAGAGTACCTTGCCAAGGATCAAAAGTTATCCAGAATGATACAATTTATCAAAGAAAGGTGTAACCCAAAATTCTCACATTTACCAATGTTGTGGCAAGTTGCAGAAACAATAGGACACTATACTGATAATCAGTCAAAGCAAATAATGGATATCAGCGAAGCGCTCATCAAAGTTAATACTTTAACTCCTGATGATGCTATGAAAGCGAGCGCAGCATTACTCGAAGTGTCGCGATGGTATAAGAATCGCAAGGAGTCGCTCAAAACCGACTCATTGGAATCTTTTAGAAACAAAATATCACCAAAGAGTACAATAAATGCAGCTCTAATGTGTGACAATCAATTGGATAAAAATGCAAATTTTGTATGGGGTAATAGGGAGTATCACGCCAAACGATTTTTCGCAAACTACTTTGAAGCAGTGGATCCCACAGATGCATATGAAAAGCACGTCACACGGTTTAACCCTAATGGTCAACGGAAGCTATCAATAGGAAAGTTAGTAATCCCACTAGACTTTCAAAAGATTAGAGAATCATTCGTTGGACTTCCGATCAATAGACAACCGCTAGACAAATGTTGCGTTAGTAAGATCGAAGGAGGATATATATACCCATGTTGCTGCGTCACAACAGAATTTGGTAAACCAGCATACTCTGAGATAATACCTCCGACAAAAGGCCACATAACAATAGGCAATTCAATTGATCCAAAGATTGTGGACCTGCCAAACACAACACCCCCCAGCATGTACATTGCTAAGGACGGGTACTGTTATATCAATATCTTTCTAGCAGCCATGATCAACGTCAATGAAGAATCTGCCAAAGATTACACGAAATTCTTGAGAGATGAACTAGTTGAGCGTCTTGGAAAGTGGCCAAGGCTTAAAGACGTAGCAACAGCGTGTTATGCATTATCTGTAATGTTTCCAGAGATTAAGAATGCTGAGCTACCTCCAATTTTAGTTGATCATGAAAATAAGTCAATGCACGTCATCGACTCATATGGTTCACTAAGCGTCGGATTTCACATATTGAAAGCAAGCACGATTGGTCAATTAATTAAATTTCAATATGAATCTATGGATAGTGAAATGCGCGAATACATAGTAGGAGGAACTCTTACGCAACAGACATTCAACACACTTCTTAAGATGCTCACGAAAAACATGTTTAAACCAGAGCGTATTAAGCAGATAATTGAGGAGGAACCCTTCTTGCTTATGATGGCAATCGCATCTCCAACGGTATTAATAGCACTATATAATAATTGTTACATTGAGCAAGCTATGACATACTGGATCGTGAAGAATCAAGGAGTTGCAGCCATATTCGCACAACTCGAAGCATTAGCCAAGAAAACATCCCAGGCTGAATTACTAGTTCTACAAATGCAGATACTTGAAAAAGCATCTAGTCAACTAAGATTGGCAGTTTCAGGACTTAGCCACGTCGACCCAGCAAAGCGACTTTTGTGGTCACACCTCGAAGCAATGACAACACGGTCAGAAATGAACAAGGAGTTGATAGCTGAGGGATATGCACTATACGACGAGCGTCTATATACCCTGATGGAAAAAAGTTACGTAGATCAATTAAACCAGTCATGGGCAGAATTATCATACTGTGGAAAATTTTCAGCAATATGGCGTGTGTTCAAAGTCAGGAAGTATTACAAACCGTCTTTAACCGTGAGAAAAAGCGTAGATTTAGGCGCTGTATACAATATATCAGCTACGCATCTAATATCAGATTTAGTGCGGAAAAGTCAAGATCAAGTCAGCTCTATTTTAACCAAACTCCGCAACGGTTTCTATAATAAATTAGAGAAAGCTAGAATACGTACTATAAAAACGGTTTATTGGTTTATACCTGATATATTTAGACTCATGCATATATTCATAGTTTTGAGTTTATTAACTACCATAGCTAACACTATCATAGTAACTATGAATGACTACAAGAAATTGAAGAAACAACAAAGAGAAGACGAATATGAAGCAGAAATTAACGAAGTTCGCAAAATCCATTCTACCTTGATGGAAGAGCGGAAGGATAATTTGACATGTGAACAATTTGTTGAGTATATGCGCCAAAATCACCCGCGGTTAGTTGAAGCAACACTGGACTTAACCCACACAGGTGTCATACATGAAGGAAAATCCAATCTCGAAACCAATTTGGAACAGGCAATGGCAGTTGGAACTTTGATAACCATGATACTTGATCCACAGAAAAGCGACGCTGTCTACAAGGTGTTGAACAAAATGCGGACAGTAATTAGTACAATTGAACAAAACGTCCCATTCCCTTCAGTGAACTTTTCCAACATCTTGACACCTCCAGTGACACAACAGAGTGTAGATGTTGACGAGCCATTGACACTTAGCACTGAGAAAAATTTAACAATAGACTTCGACACGAATCAAGATTTACCTGCCGATACATTCAGTAATGATGTGACATTTGAAGATTGGTGGTCAAATCAATTAAGCAACAACAGAACAGTACCACACTACCGACTTGGGGGAAAGTTTGTTGAATTCACACGAGAAAACGCAGCCCACACGAGCATCGAACTTGCACACTCAAACATCGAGAGGGAGTTCTTGCTTAGAGGAGCAGTCGGCTCGGGAAAATCTACTGGGTTACCATACCATCTTAGCATGCGCGGAAAAGTGCTTTTGCTAGAGCCTACAAGGCCACTAGCCGAGAACGTGTGTAGGCAATTACAAGGACCACCATTTAATGTAAGTCCAACTCTCCAAATGCGTGGACTGAGTTCTTTCGGGTGCACTCCAATCACGATCATGACATCAGGTTTTGCATTGCACATGTACGCAAACAATCCAGATAAAATATCTGAATACGATTTTATAATCTTCGATGAATGCCATATAATGGAAGCACCAGCAATGGCCTTTTATTGCTTACTCAAAGAATACGAATACCGAGGAAAAATCATCAAGGTATCAGCCACGCCTCCAGGAAGGGAATGCGAATTCACAACACAACATCCAGTRGACATCCATGTTTGTGAAAATCTAACTCAGCAACAATTTGTTATGGAACTCGGAACCGGTTCAACCGCAGATGCCACGAAGTACGGAAATAATATCTTAGTTTATGTAGCAAGTTATAATGACGTCGATTCATTGTCGCATGCATTAGTTGAACTCAAATTTTCCGTCATTAAAGTGGATGGCCGAACAATGAAGCAAAACACAACAGGAATCATCACAAACGGCACCTCACAAAAGAAGTGCTTTGTTGTCGCAACGAATATAATTGAAAATGGCGTCACACTAGATATTGATGTTGTTGTTGACTTYGGGCTGAAAGTCTCAGCCGATTTGGATGTTGACAACAGGGCAATATTGTATAAACGCGTGAGTATATCATATGGTGAACGCATACAACGACTGGGTCGTGTTGGAAGAAATAAACCCGGCACAGTTATTCGTATTGGAAAAACCATGAAAGGTTTGCAGGAAATCCCAGCAATGATCGCAACAGAAGCAGCCTTCATGTGTTTCGCATATGGTCTTAAAGTCATTACCCATAATGTTTCAACAACCCATCTTGCAAAGTGCACAGTCAAACAAGCAAGAACCATGATGCAATTTGAATTATCACCATTTGTTATGGCTGAGCTCGTTAAGTTTGATGGTTCAATGCATCCACAAATACATGAGGCTTTAGTAAAATACAAACTTAGAGATTCTGTCATAATGCTCAGACCGAATGCAATTCCAAAAGTTAATTTACACAATTGGCTCACAGCCCGGGATTATAATAGAATAGGTTGCTCATTGGAACTTGAAGATCACGTTAAAATTCCGTACTATATTAGGGGAGTTCCTGACAAGCTGTATGGAAAGCTATATGATATTATTTTACAGTATAGTCCAACTAGTTGTTATGGTAGACTATCAAGTGCGTGTGCAGGTAAAGTAGCATACACTTTGCGAACTGATCCGTGTTCACTTCCAAGAACAATAGCAATAATTAATGCTTTAATCACGGAGGAATATGCGAAGAGAGATCACTACCGAAACATGATTTCAAACCCCTCCTCATCACATGCATTCTCACTCAATGGGTTGGTATCCATGATCGCAACTAGATATATGAAGGATCATACAAAAGAGAATATTGACAAACTCATCAGAGTACGTGATCAATTACTTGAGTTTCAAGGTACCGGAATGCAATTTCAAGATCCGTTAGAACTCATGGAAATTGGGGCTCTCAATACAGTCATTCACCAAGGAATGGATGCAACTGCAGCTTGTATCGGATTACAAGGACGATGGAATGCTTCGCTCATACAACGTGATCTCCTAATTGCAGGAGGAGTTTTTATCGGAGGCATTTTAATGATGTGGAGCCTATTCACTAAATGGAGCAACACAAGTGTCTCACATCAAGGAAAGAACAAACGCAGTAGACAAAAACTCCGATTCAAAGAAGCAAGAGACAACAAATATGCATATGATGTCACAGGATCGGAAGAGTGCCTTGGTGAGAATTTCGGAACAGCCTATACAAAGAAAGGTAAAGGAAAAGGAACTAAAGTTGGACTCGGTGTGAAGCAACACAAATTTCACATGATGTATGGTTTTGACCCTCAAGAGTACAACCTAATCCGATTTGTCGATCCACTCACAGGAGCAACTCTTGATGAGCAAATCCATGCCGACATACGCTTAATTCAAGAGCATTTCGCTGAGATTCGTGAGGAGGCAGTAGCTAACGACACAATTGAAAGGCAGCAGATCTACGGCAATCCTGGACTACAAGCATTTTTCATACAAAATGGGTCAGCAAACGCTCTGAGAGTTGATTTAACGCCACATTCACCTACACGGGTTGTCACAGGCAATAACATAGCAGGATTCCCAGAATATGAAGGTACACTCCGTCAAACTGGAACAGCTATAACCATACCCATTGGTCAAGTCCCAATTGCAAATGAAGCAGGAGTCGCACACGAATCAAAATCTATGATGAATGGATTGGGTGATTACACGCCAATATCGCAACAATTGTGTTTAGTGCAAAATGACTCAGATGGAGTAAAACGAAATGTATTTTCAATTGGATATGGTTCATATCTCATTTCACCAGCGCACTTATTCAAATATAACAATGGCGAAATAACAATTAGATCATCAAGAGGATTGTATAAAATTCGTAATTCTGTGGATTTAAAACTACACCCGATTGCACAAAGAGACATGGTCATAATTCAACTTCCAAAGGATTTCCCACCGTTCCCAATGCGTTTGAAATTCACACAACCATCACGAGATATGCGAGTCTGCTTAGTGGGAGTCAATTTCCAACAGAATTATAGCACTTGCATCGTATCAGAAAGTAGTGTGACAGCACCAAAAGGAAATGGAGATTTTTGGAAACATTGGATATCAACAGTCGACGGTCAATGTGGACTACCATTGGTAGATACTAAGAATAAACACATTGTCGGAATTCATAGTCTTGCATCTACAAGTGGAAATACCAACTTCTTTGTTGCTATGCCTGAGAACTTTAATGAATACATTAATGGACTTGTGCAAACAAACAAATGGGAAAAGGGATGGCACTATAATCCGAACCTCATATCTTGGTGTGGACTAAACTTAGTAGACTCTGCTCCAAAGGGTTTGTTTAAAACGTCAAAATTAGTTGAAGATTTGGATGCTAGTGTTGAAGAGCAGTGCAAAGTTACTGAAACATGGCTCACAGAGCAATTACAAGATAATTTGCAAGTGGTCGCGAAATGCCCAGGCCAACTCGTCACTAAGCATGTCGTCAAAGGCCAATGCCCACACTTCCAGTTATATTTGTCAACACATAATGATGCCAAAGAATATTTCGCACCCCTGCTTGGAAAATATGATAAGAGCAGACTCAATAGAGCGGCATTCATCAAAGACATATCAAAATATGCAAAACCAATTTATATTGGGGAAATCAATTATGATATCTTTGATAGAGCTGTACAACGAGTCATTAATATTCTTAAGAATGTTGGAATGCAACAATGCGTTTACGTCACAGATGAAGAAGAAATTTTTAAATCACTTAACCTAAACGCAGCTGTCGGAGCACTGTACACAGGAAAGAAGAAAGATTACTTTGAAAGTTTTTCAAATGAAGACAAGGAAGAAATCGTGATGAGATCATGTGAACGCATATACAATGGACAACTCGGCGTGTGGAATGGGTCACTCAAAGCCGAAATCAGACCAATAGAGAAAACCATGTTAAATAAGACTCGAACCTTTACAGCAGCTCCATTGGAAACTTTGCTTGGAGGAAAAGTGTGTGTGGACGATTTTAATAATCAATTTTATTCACACCATTTAGAAGGCCCATGGACTGTTGGGATAACAAAATTTTATGGAGGTTGGAATCGCTTACTTGAGAAGTTGCCAGAAGGATGGGTTTACTGCGACGCTGACGGATCCCAATTCGATAGTTCATTAACACCATATCTCATTAATGCAGTGTTGAATATTCGATTACAGTTCATGGAAGATTGGAACATAGGAGCGCAAATGCTTAAAAACCTTTATACTGAGATTGTTTATACACCAATTGCAACGCCAGATGGATCCATTGTGAAGAAATTCAAAGGAAACAATAGTGGACAACCTTCTACAGTAGTTGATAATACATTGATGGTTATAATAGCTTTCAACTATGCTATGCTATCGAGCGGTATTAAAGAAGAAGAAATTGATAACTGCTGTAGGATGTTTGCAAATGGTGACGACCTGCTCCTAGCAGTGCATCCCAATTTTGAATACATTCTAAACGGATTCCAAGATCACTTCGGAAATCTCGGATTAAATTTTGAGTTTACATCACGAATGCGAGATAAATCCGAACTGTGGTTTATGTCTACAAGAGGAATCAAATGTGAAGGAATCTACATACCTAAACTCGAGAAAGAAAGAATAGTCGCGATACTCGAATGGGATCGATCAAATTTACCTGAGCATAGATTGGAAGCCATTTGTGCAGCCATGGTTGAAGCATGGGGCTACTCAGATCTTGTTCATGAAATTCGGAAATTTTATGCGTGGCTTCTAGAAATGCAACCTTTCGCAAATCTAGCAAAAGAAGGCATGGCGCCATACATAGCAGAAACAGCGCTCCGTAACCTTTATCTTGGAACGGGCATTAAAGAAGAAGAAATCGAGAAATATTTCAAGCAATTTGTTAAGGATCTTCCTGGATACATAGAAGATTACAACGAAGAGGTCATTCACCAATCGGGAACAGTTGATGCAGGTGCACAAGGCGGCAGCGGGAATCAAGGAACAACACCACCAGCAACAGGTGGCGGAGCAAAACCAGCGGCTTCAGGAA------------CAGGATCTGGTAGCGGAAC----AGGAACCGGAACTGGTGCAACTGGAGGCCAAACAGGA---------GCT---G---GCAGTGGTGCTGGAGCAGGATCTGGAGCAACCGGAGGCCAATCAGGATCTGGAAGTGGCACTGGACAGACTAACACGGGCTC---AGCAGGAACCGGTGCA------------ACAGGAGGTCAAAGAGATAAGGATGTAGATGCAGGTACAACAGGACAAATGTCTGTACCAAAGCTCAAGGCCATGTCAAAGAAAATGCGCTTACCGAAAGCAAAAGGAAAAGATGTCTTGCATCTGGACTTCTTGCTTACATACAAGCCACAGCAGCAAGACATATCAAACACAAGAGCAACTAAGGAAGAGTTTGATAGATGGTACGATGCCATAAAGAAAGAATACGAGATTGATGACACACAAATGACAATTGTCATGAGCGGTCTTATGGTGTGGTGCATTGAGAACGGTTGCTCGCCAAACATAAACGGAAATTGGACAATGATGGATGGAGATGAACAAAGAGTTTTTCCACTCAAACCAGTCATTGAAAACGCATCTCCAACTTTCCGACAAATTATGCATCATTTTAGTGATGCAGCTGAAGCGTACATAGAGTACAGAAACTCTACTGAGCGATACATGCCAAGATACGGACTTCAGCGCAATCTCACCGACTATAGCTTAGCACGGTATGCATTTGATTTCTATGAAATGACTTCACGCACACCTGCTAGAGCTAAAGAAGCCCACATGCAG---ATGAAAGCCGCAGCAGTTCGTGGTTCAAACACACGACTGTTCGGTCTGGACGGAAATGTCGGCGAGACCCAGGAGAATACAGAGAGACACACAGCTGGCGATGTTAGTCGCAATATGCACTCTCTGTTGGGAGTGCAGCAGCACCACTAGTCTCCTGGAAACCCTGTTTGCAGTACCTATAATATATACTA------ATATATAGTATCTCAGTGAGGTTTTACCTC-----------------------GACTTTACTATTTTATTACGTATGTATTTAAAGCGTGAACCAGTCTGCAGCATACAGGGTTGGACCCAGTGTGTTCTGGTGTAGCGTGTACTAGCGTCGAGCCATGAGATGGACTGCACTGGGTGTGGCTTTGCCACTTGTGTTGCGAGTCTCCTGGTAAGAGACAA--------------

>MH093722

-----------------------------------CAACACAACACAACAAAACACAACCAAACAAAACCAAGTTACTTTTGCTCAGATTGTAGTGAACGGCTCGGTGGGAAAGGTTCCTCGAGATCACTCTCTGACTCCTCTCTC----------TCAACCAACTTCATTCAAGCGAGATGGCGGGCTCTTGGACTCACGTGACATACAAATGGCAACCAGACGTCAACAACACACGTGATGTGAAAAGAGTGATGGAGATGTTTGTAGCGAAACATCAACGTTACACTGAGGAACAAAGGCTTGCTCACAACAGCAAGCTATTAAGGAAGGCTAGTGTTACTAGTGCTGAGTTTACTGAATCAGCACAGAGACCAAAATGTCATCAGACATGGGTTGAAAAGTACGACCACAACCCCACAAAGCACTTTGTTTATCAACGTTTC---ACACCTGAGAAGAAAGTGCCAGCCACCAAACCTGAGACAACTTCTGTCACGAAGTTAATCAGGGATATCCTTGAGATTTCGAAGGGCAGTGGGATAAAAATCGAATTAATTGACAAGCGTGTCAGACGTAAAACTCAATTATCCATAAGGCGACACAATGGTAAAGATTTCCTGCACTGCAAAACCAGGCATGAAAATGGCCTGTTTAAACGCAAGGACGTTGACATTAGTGTCAAGTGGTTGCCCACCATTAAAGCCATTGCAAAATGCTACAGTACTGTGAATGCAGAAGAATTGCAAAGTCTCAGTAGAGGCAGTAGTGGTCTAACATTCATGCAGAATGATGAATTATTCATCGTGCGTGGAAGGATGCATGGTAAGATTGTCAATAGTTTGCATGAGAATAAGCACGTAATGGAAATCGAACACTATGCTGATCCACAGGCAAACAGTTTCTGGAAAGGCTACACAGATGCATATGTCGAGAACAGAAACATATCCACTACTCACACAGAGCACACACCAACTATCAATTTAGAAGAGTGTGGCAAGAGAATGGCACTGTTAGAAATCCTATTCCATTCAACTTTTAAAATAACATGCAAAACATGTAATATTGACGACCTTGAATTATCAGATGATGAATTTGGGGCCAAGTTGTATAGTAATCTGCAGCGCATTGAAGAAAAACAACGTGAATATCTTGCTAAAGATCAAAAACTTTTACGCATGATTCATTTTGTAAAGGATCGATGTAATCCAAAGTTTTCACATTTACCCTTGCTATGGCAAGTGGCAGAAACAGTGGGGCATTACACTGATAACCAATCGAAGCAGATAATTGATATCAGTGAAGCGCTCATAAAAGTTAATACCTTAACTCCTGATGATGCAGTAAAGGCCAGTGTGGCATTATTGGAAGTAGCACGGTGGTATAAGAATCGGAAGGAATCACTTAAAACGGACACATTGGATTCATTTCGAAACAAGATTTCACCAAAGAGCACGATCAACGCAGCATTAATGTGTGATAACCAGTTAGATAAGAATGCAAATTTTGTGTGGGGAAACAGAGAATACCATGCAAAGCGATTCTTCGCCAACTATTTTGAAGCTGTGGACCCAACTGATGCATATGAAAAACATGTAACACGCTTCAACCCCAATGGACAGCGGAAATTATCAATTGGCAAACTAGTAATCCCATTAGATTTCCAGAAAATCAGAGACTCGTTCGTTGGCCTATCAATAAATAAACAACCACTGAGCAAAGCTTGCGTAAGTAAAATCGATGGAGGTTACGTGTATCCATGTTGCTGCGTTACAACAGAATTTGGAAAACCAGCATATTCTGAGATAATACCTCCAACGAAAGGACATATCACGATTGGAAATTCAGTGGACCCGAAAATAGTGGATTTACCAAATACAACACCACCGAGTATGTACATTGCAAAAGATGGATACTGTTATATTAACATATTCTTGGCAGCAATGATAAACGTCAACGAGGAATCCGCAAAAGATTACACTAAGTTTCTTAGAGACGAGTTGGTGGAACGGCTTGGTAAATGGCCAAAATTGAAAGATGTGGCCACAGCATGCTATGCTTTGTCAGTGATGTTCCCAGAGATAAAGAATGCCGAATTACCACCAATACTAGTGGATCATGAGAGTAAGTCAATGCACGTCATTGATTCATATGGATCACTCAGCGTTGGCTTTCACATTCTAAAGGCAAGTACTGTTGGACAACTGATAAAATTTCAGTATGAGTCATTGGAAAGTGAGATGCGCGAGTACATAGTGGGTGGCACTTTAACACAGCAAACTTTCAGCACACTTCTTAAGACTCTCACAAAGAACATGTTTAAGCCAGATAAAGTAAAGCAGATAATTGAGGAAGAGCCATTCTTATTAATGATGGCAATTGCATCCCCAACTGTACTCATCTCGCTGTACAATAACTGCTACATCGAGCAAGCAATGACATATTGGATCGTCAAGAACCAAGGCATCGCAGCGCTTTTTGCGCAGTTGGAGGCACTAGCAAAGAAAACTTCTCAAGCGGAGCTACTAGTTCTTCAAATGCAAATACTTGAGAAAGCTTCGAACCAACTGAGACTTGCAGTCACGGGACTTAATCATGTTGATCCAGCTAAACGACTTTTATGGTCTCACCTGGAAGCTATGACAACACGGTCAGAAATGAATAAGGAACTCATAGCGGAAGGCTATGCACTGTACGACGAGCGCTTATACACTTTAATGGAAAAAAGTTACGTAGATCAATTAAACCAATCATGGGCAGAATTATCATACTGTGGAAAATTTTCAGCAATATGGCGTGTGTTCAGAGTCAGGAAATACTACAAACCATCTTTAACCGTGAGAAAAAGCGTAGATTTAGGCGCTGTGTACAATATATCAGCTACGCATCTAATATCAAATTTAGTGCAGAAAAGTCGAGATCAAGTCAGCTCTACTTTAACCAAACTCCGCAACGGTTTCTATGATAGAATGGAGAGAGCGAGAGTTAGTGCAGTAAGGACAGTATATTGGTTCGTACCCGATATATTTAGACTAATACATATTTGCTTAGTTTTAAGTATATTAACAACTATAGCTAATACAATAATCGCGATTATGAATGATTATAAAAAGTTGAAAAAGCAACAAAGAGAAGACGAATACGAAGCCGAGATTAACGAGGTACGAAGGATACACGCCAACCTAATGAAGGAGCATAATGATGACTTAACATGTGAACAATTTATTGAGCACATACGACAGACACATCCACGCCTCATTGAGGCAACATTGGATTTAACACATACAGGTGTCATCCATGAGGGCAAATCCAATTTAGAAACAAACCTCGAACAGGCAATGGCAGTGGGAACTTTACTCACTATGATACTCGATCCACAGAAGAGTGACGCAGTTTATAAGGTTCTCAATAAGATGCGAACAGTTATCAGCACAATAGAACAGAATGTACCATTTCCCTCAGTGAACTTCACGAGCATCTTGTCACCTCCTGTAACTCAGCAAAGTGTAGATGTTGACGAACCGTTAACACTGAGCACCGATAAGAATTTGACTATAGATTTCGACACAAATCAAGATTTGCCAGCGGACACATTTAGCAATGACGTTACGTTCGAGAACTGGTGGGCTAATCAGATAAACAACAACAGAACAGTGCCACACTATCGACTTGGGGGAAAGTTTGTAGAATTCACAAGAGAGAATGCAGCAATGGTTAGCATTGAGCTCGCCCATTCGAACATCGAAAAAGAGTTTCTACTCAGAGGAGCCGTTGGGTCAGGAAAATCCACAGGCTTGCCATACCATCTCAGTATGCGTGGAAAAGTGCTATTGATAGAACCCACTCGACCATTAGCTGAGAACGTTTGCAGACAACTGCAAGGTCCTCCATTTAATGTGAGCCCCACTTTACAAATGAGAGGATTGAGCACATTTGGCTGCACTCCTATCACGATAATGACATCTGGCTTCGCATTGCACATGTATGCTAATAACCCCGATAAGATCTCTGAGTATGACTTCATTATCTTTGATGAATGTCACATTATGGAAGCACCTGCAATGGCATTCTATTGTTTGCTTAAGGAGTATGAATATCGAGGCAAGATAATAAAAGTTTCAGCTACACCACCAGGACGAGAATGCGAGTTTTCAACCCAACATCCAGTAGATATACATGTATGTGAAAGCTTGACACAACAGCAATTCATCATGGAATTAGGAACAGGATCAACTGCTGATGCAACCAAATATGGCAATAACATATTAGTGTACGTTGCAAGTTATAATGATGTAGATTCTTTATCCCATGCTCTAACTGAACTTAAATATTCAGTGATTAAAGTCGATGGAAGAACTATGAAGCAGAACACCACAGGAATCGTAACAAATGGAACATCCAGTAAGAAATGCTTCGTTGTGGCCACAAATATTATTGAAAACGGTGTAACGCTAGATGTCGACGTTGTCGTCGACTTTGGACTTAAAGTAACAGCTGAATTAGATGTTGATAACAGGGCGATAATGTATAAACGTGTGAGCATATCTTATGGCGAGCGCATTCAGAGACTCGGAAGAGTCGGAAGGAATAAGCCTGGGACAGTTATCCGCATCGGAAAAACAATGAAAGGCTTACAAGAAATTCCTGCGATGATTGCTACTGAAGCAGCTTTCATGTGTTTTGCATATGGACTGAAAGTTATAACACATAATGTATCAACAACACATCTAGCAAAATGCACTGTCAAACAAGCTAGAACCATGATGCAATTCGAACTATCACCATTTGTAATGGCTGAATTAGTTAAATTCGATGGTTCCATGCATCCACAGATTCATGAAGTGTTAACCAAGTATAAATTGAGGGATTCTGTGATCATGTTAAGACCAAACGCAATACCAAAGGTTAACCTTCACAACTGGTTAACGGCCCGAGATTACAATAGGATTGGCTGCTCACTGGAACTCGAGGATCACGTTAAGATACCATATTATATACGAGGAGTTCCTGACAAGTTGTATGGGAAGTTATATGATATCATCCTTCAATACAGCCCTACAAGTTGTTATGGAAGATTGTCAAGTGCTTGCGTAGGTAAGGTCGCATATACATTGCGCACTGATCCTTGTTCATTACCGAGAACAATAGCTATAATCAACGCACTGATTACTGAAGAGTATGCAAAGAGGGACCACTACAGAAACATGATAGCAAACCCCTCATCATCACACGCCTTCTCACTTAATGGACTAGTATCCATGATCGCGTCTCGGTACATGAAAGATCACACGAAGGAGAACATAGATAAACTTGTAAGAGTGCGCGACCAGCTACTTGAGTTCCAAGGCACAGGCATGCAATTTCAAGATCCTTCAGAATTGATGGATATTGGAGCATTAAATACAGTTATCCACCAAGGAATGGACGCCACGGCCGCTTGCATCGGATTACAAGGGCGTTGGAATGCTTCACTCATCCAGCGCGATTTGATGATATCAGCAGGGATCTTCACAGGAGGAATTCTTATGATGTGGTGTCTCTTTACAAAATGGAGCAAGACAGAAGTGTCACATCAAGGAAAGAACAAACGCAGTAGACAAAAACTCCGATTCAAAGAAGCAAGAGACAACAAATATGCATATGATGTCACAGGATCGGAAGAGTGCCTTGGTGAGAATTTCGGTACAGCCTATACAAAGAAAGGTAAAGGAAAAGGAACTAAAGTTGGACTCGGTGTGAAGCAACACAAATTTCATATGATGTATGGTTTTGACCCTCAAGAGTACAACCTAATTCGATTTGTTGATCCACTCACAGGAGCAACTCTTGATGAGCAAATCCATGCCGACATACGCTTAGTTCAAGAGCATTTCGCTGAAATTCGTGAGGAGGCAGTAGCTAACGACACAATTGAAAAGCAGCATATCTACGGCAATCCTGGACTACAAGCATTTTTCATACAAAATGGTTCAGCAAATGCTCTGAGAGTCGATTTGACGCCACATTCACCTACACGAGTTGTCACAGGTAATAATATAGCAGGATTCCCAGAACACGAGGGTACACTTCGTCAAACTGGAACAGCAATAACCATACCCATTGGTCAAGTCCCAATCGCACGTGAAGCAGGAGTTGCACACGAGTCAAAGTCCATGATGAATGGGCTGGGTGACTACACGCCAATATCGCAGCAGTTATGTTTAGTGCAAAATGACTCAGATGGGGTAAAGCGGAATGTGTTTTCAATTGGATATGGTTCATATCTCATTTCACCAGCGCACTTATTCAAATATAACAATGGTGAAATAACAATTAGATCATCAAGAGGATTGTATAAAATTCGTAATTCTGTGGATTTAAAACTACATCCAATTGCACACAGAGACATGGTCATAATTCAACTTCCAAAGGATTTCCCACCGTTCCCAATGCGTTTGAAATTCACACAACCATCACGAGATATGCGAGTTTGCTTAGTGGGAGTCAATTTCCAACAGAATTATAGCACTTGCATCGTATCAGAAAGTAGTGTGACAGCTCCAAAAGGAAATGGAGACTTTTGGAAACATTGGATATCAACAGTCGACGGTCAATGTGGACTGCCATTAGTAGACACTAAGAATAAACACATTGTCGGAATCCATAGTCTTGCATCTACAAGCGGAAATACCAACTTCTTCGTCGCTATGCCTGGGAACTTTAATGAATATATTAATGGACTTGTGCAAACAAACAAATGGGAAAAGGGGTGGCACTATAATCCGAATCTTATATCTTGGTGTGGACTAAACTTAGTCGACTCTGCTCCAAAGGGTTTGTTTAAAACGTCAAAATTAGTTGAAGATTTGGATGCTAGTGTTGAAGAGCAGTGCAAAGTTACTGAAACATGGCTCACAGAGCAATTACAAGATAACTTGCAAGTGGTCGCGAAATGCCCAGGCCAACTCGTCACTAAGCATGTCGTCAAAGGCCAATGCCCACACTTCCAGTTATATTTGTCAACACATAATGATGCCAAAGAATATTTCGCACCCCTGCTCGGAAAATATGATAAGAGCAGACTCAACAGAGCGGCATTTATCAAAGACATATCAAAGTATGCAAAACCAATTTATATTGGAGAAATCAATTATGATATCTTTGATAGAGCTGTACAACGAGTCATTAATATCCTTAAAAATGTTGGAATGCAACAATGCGTTTATGTCACAGATGAAGAAGAAATTTTTAAATCACTTAACCTAAACGCAGCTGTCGGAGCACTGTACACAGGAAAGAAGAAAGATTATTTTGAAAGTTTTTCAAATGAAGACAAGGAAGAAATCGTGATGAGGTCATGTGAACGCATTTACAATGGACAACTTGGTGTGTGGAATGGGTCACTTAAAGCCGAAATCAGACCAATAGAGAAAACCATGTTAAATAAGACTCGAACTTTTACAGCAGCTCCATTGGAAACTTTGCTTGGAGGAAAAGTGTGTGTGGACGATTTTAATAATCAATTTTATTCACACCATTTAGAAGGCCCATGGACTGTTGGGATAACAAAATTCTATGGAGGTTGGAATCGCTTACTTGAGAAGTTGCCGGAAGGATGGGTTTACTGCGACGCTGACGGATCTCAATTCGATAGTTCATTAACACCTTATCTTATTAATGCAGTGTTGAATATTCGATTACAGTTTATGGAAGATTGGAACATAGGAGCGCAAATGCTTAAAAACCTTTATACTGAAATTGTTTATACACCAATTGCAACACCAGACGGATCTATCGTGAAGAAATTCAAAGGAAATAATAGTGGACAACCTTCTACAGTAGTTGATAATACATTGATGGTTATAATAGCTTTCAACTATGCCATGCTATCGAGCGGTATTAGAGAAGAAGAGATTGATAACTGCTGTAGGATGTTTGCAAATGGTGACGACCTGCTCCTAGCAGTACATCCTGATTTTGAATACATTCTAAACGGGTTTCAAGATCACTTCGGAAATCTTGGATTGAATTTTGAGTTTACATCACGAACACGAGATAAATCCGAACTATGGTTTATGTCTACAAGAGGAATCAAATGTGAAGGAGTCTACATACCTAAACTCGAGAAAGAGAGAATAGTCGCCATACTTGAGTGGGACCGATCGAACTTGCCCGAACATAGGCTAGAAGCTATATGTGCAGCGATGGTTGAGGCTTGGGGATATTCTGATCTTATTCATGAAATACGAAAATTCTATGCGTGGCTTCTAGAAATGCAACCCTTTGCAAATCTTGCAAAAGAAGGGTTGGCTCCATATATTGCTGAGACAGCACTCCGCAATCTGTATTTTGGAACGGGCATCAAAGAAGAAGAAATTGAAAAATACTTTAAACAATTTGTTAAGGATCTTCCTGGATACATAGAAGATTACAATGAAGATGTAATCCATCAGTCGGGAACTGTTGATGCAGGTGCACAAGGCGGCGGTGGAAACCAAGGAACAACACCACCAGCAACA---------------------------------------------------GGTGGTGGCCC----AGG------------------------------------------------------------------------------------------------------------------------------------------------------------------------------------------------------------------------------------------------------------------------------------------------------------------------------------------------------------------------------------------------------------------------------------------------------------------------------------------------------------------------------------------------------------------------------------------------------------------------------------------------------------------------------------------------------------------------------------------------------------------------------------------------------------------------------------------------------------------------------------------------------------------------------------------------------------------------------------------------------------------------------------------------------------------------------------------------------------------------------------------------------------------------------------------------------------------------------

>MH093723

-------------------------------------------------------------------------------------------------------------------------------------------------------------------------------------------------------------------------------------------------------------------------------------------------------------------------------------------------------------------------------------------------------------------------------------------------------------------------------------------------------------------------AACCAGGGAGATTCTCGAAATCTCGAAGGCTAGTGGCTTGAAGGTTGAATTGATTGATAAGCGCAAAAGATCTAAAACACAGTTGTCAATCAAAAGGTTCAATGGCAAGGACTTTCTCCATTGCAAAACAAAACACGAAAATAATTTGTTTAAGAGGAAGGACATAGCTATCGGACACAAATGGCTTCCAACAATCGAAGCCATTGCTCGCTGTTATAGCACAATGAACCAAGAAGAATTGCAAAGCCTTTATAGAGGCAGCAGTGGTCTCACATTCATTCAAAATGATGAATTATTCATTGTTAGAGGAAGAATGAATGGTGAACTAGTTAATAGTCTATGTGAAACAAACCGGATTTTGGATATTGAACACTATGCAGATCCCCAGGCTAATGACTTTTGGAGGGGATACACAGATGCTTACGTAGATAATCGTAGCATTTCTACCACTCATACAGAGCATACCCCAACAATCAATCTAGTAGAGTGTGGAAAACGAATGGCTCTACTTGAGATATTATTTCATTCCACATTCAAGATCACATGTAAAACATGTAACATTGATGATCTTGAATTATCAGATGATGAATTTGGAGCTAAACTCTACAAAAATCTGCAACGCATCGAAGAGAAGCAACGAGAGTATCTTGCCAAGGATCAAAAGTTATCCAGAATGATACAATTTATCAAAGAAAGATGCAATCCAAAATTCTCACATTTACCAATGTTGTGGCAAGTTGCAGAAACAATAGGACACTACACTGATAATCAGTCTAAGCAAATAATGGATATCAGTGAAGCACTCATCAAAGTTAATACTTTAACTCCTGATGATGCAATGAAAGCGAGTGCAGCATTACTCGAAGTGTCACGGTGGTATAAGAATCGTAAGGAATCACTCAAAACTGACTCATTGGAATCTTTTAGAAATAAAATATCACCAAAGAGTACAATAAATGCAGCTTTGATGTGTGACAATCAATTGGATAAAAATGCAAATTTTGTATGGGGTAATAGGGAATACCACGCTAAACGATTCTTCGCAAATTATTTTGAAGCAGTGGATCCTACAGATGCATATGAAAAGCACGTCACACGGTTTAACCCTAATGGTCAACGGAAGCTATCAATAGGAAAGTTAGTTATCCCACTAGACTTTCAAAAGATTAGAGAATCATTCGTTGGACTTCCGATTAATAGACAGCCGCTAGATAAATGTTGCGTTAGTAAGATCGAAGGGGGATATATATACCCATGTTGCTGCGTCACAACAGAGTTTGGCAAACCAGCATACTCTGAGATAATACCTCCAACAAAAGGCCACATAACAATAGGCAATTCAATTGATCCAAAGATTGTGGACCTGCCAAATACAACACCACCCAGCATGTACATTGCTAAGGACGGGTACTGTTATATCAACATCTTTCTAGCAGCCATGATCAACGTCAATGAAGAATCTGCCAAGGATTACACGAAATTTTTGAGAGATGAGCTAGTTGAGCGTCTCGGAAAGTGGCCAAAGCTTAAAGATGTAGCAACAGCATGTTATGCGTTATCTGTAATGTTTCCAGAAATTAAGAATGCCGAGTTACCTCCAATTTTAGTTGATCATGAGAATAAAACAATGCACGTCATCGATTCATATGGTTCACTAAGCGTCGGATTTCACATATTAAAAGCAAGCACAATTGGTCAATTAATCAAATTCCAATATGAGTCTATGGATAGTGAAATGCGCGAATACATAGTGGGAGGAACTCTCACGCAACAGACATTCAACACACTCCTTAAGATGCTTACGAAGAACATGTTCAAACCAGAGCGCATCAAACAGATAATTGAGGAGGAACCCTTCTTGCTCATGATGGCAATCGCATCTCCAACGGTATTAATAGCACTATATAATAATTGTTATATTGAGCAAGCTATGACATACTGGATCGTGAAGAATCAAGGAGTTGCAGCTATATTCGCACAACTCGAAGCATTAGCTAAGAAAACATCCCAGGCTGAATTACTAGTTCTACAAATGCAGATACTTGAAAAAGCATCTAGTCAACTAAGATTAGCAGTCTCAGGACTTAGCCATGTCGACCCAGCAAAGCGACTTTTGTGGTCACACCTCGAAGCAATGACAACACGGTCAGAAATGAACAAGGAGTTGATAGCCGAGGGATATGCACTATATGACGAGCGTCTATATACCCTGATGGAAAAAAGTTACGTAGATCAATTAAACCAGTCATGGGCAGATTTATCATACTGTGGAAAATTTTCAGCAATATGGCGTGTGTTCAAAGTCAGGAAGTATTACAAACCGTCTTTAACCGTGAGAAAAAGCGTAGATTTAGGCGCTGTGTACAATATATCAGCTACGCATCTAATATCAGATTTAGTGCGGAAAAGTCAAGATCAAGTCAGCTCTATTTTAACCAAACTCCGCAACGGTTTCTATGATAAATTAGAGAAAGCTAGAATACGTACTATAAAAACGGTTTATTGGTTTATACCTGATATATTTAGACTTATGCATATATTCATAGTTTTGAGTTTATTAACCACCATAGCTAACACTATCATAGTAACTATGAATGATTACAAGAAATTGAAGAAACAACAAAGAGAAGACGAATATGAAGCAGAAGTTAACGAAGTTCGCAAAATCCATTCTACCTTGATGGAGGAGCGGAAGGACAATTTGACGTGTGAACAATTTGTTGAATATATGCGCCAAAATCATCCGCGGTTAATTGAAGCAACACTGGACTTAACCCATACAGGTGTCATACACGAAGGAAAATCCAATCTCGAAACCAATTTGGAACAGGCAATGGCAGTTGGAACCTTGATAACCATGATACTTGATCCACAGAAAAGCGACGCTGTTTACAAGGTGTTGAATAAAATGCGGACAGTAATTAGTACAATTGAACAGAATGTCCCATTCCCTTCAGTGAATTTCTCCAGCATCTTAACACCTCCAGTGACACAACAGAGTGTGGACGTTGATGAGCCATTAACACTTAGCACTGATAAAAATTTGACAATAGATTTTGACACGAATCAAGATTTACCTGCTGATACATTCAGCAATGATGTGACATTTGAAGATTGGTGGTCAAATCAATTAAGCAACAACAGAACAGTGCCACACTACCGACTTGGGGGAAAGTTTGTCGAATTCACACGAGAAAACGCAGCCCACACGAGCATCGAACTTGCACACTCAAACATTGAGAAGGAGTTCTTGCTTAGAGGAGCAGTTGGCTCGGGAAAATCTACTGGATTACCATACCATCTTAGCATGCGTGGAAAAGTGCTTTTGTTAGAACCTACAAGGCCGCTAGCTGAGAACGTGTGTAGGCAATTACAAGGACCACCATTTAACGTAAGTCCAACTCTCCAAATGCGTGGACTGAGTTCTTTCGGGTGTACTCCAATCACAATCATGACGTCAGGTTTCGCATTGCACATGTACGCAAACAATCCAGATAAAATATCTGAATACGACTTTATAATCTTCGATGAATGTCACATAATGGAAGCACCAGCAATGGCCTTTTATTGCTTACTCAAAGAGTATGAATACCGAGGGAAAATTATCAAGGTATCAGCTACGCCTCCAGGAAGGGAGTGTGAATTCACTACACAACATCCAGTTGACATCCATGTTTGTGAGAATCTAACTCAGCAACAATTTGTCATGGAACTCGGGACTGGTTCGACTGCAGATGCTACGAAGTACGGAAATAACATCTTAGTTTATGTAGCAAGCTATAATGACGTTGATTCATTGTCACAAGCATTAGTCGAACTAAAATTTTCCGTCATTAAAGTGGATGGCCGAACAATGAAACAAAACACAACAGGAATCACCACAAACGGAACCTCACAAAAGAAGTGTTTTGTTGTTGCAACAAATATAATTGAAAACGGCGTCACACTAGATATCGATGTTGTTGTCGACTTCGGACTCAAGGTCTCAGCTGACTTGGACGTTGACAATAGGGCAATATTGTATAAACGTGTGAGCATATCATATGGTGAACGCATACAACGACTAGGTCGTGTCGGAAGAAACAAACCTGGCACAGTTATTCGCATCGGAAAAACAATGAAAGGTTTGCAGGAAATTCCAGCAATGATCGCAACAGAAGCAGCCTTCATGTGTTTCGCATATGGCCTTAAAGTCATCACCCATAATGTTTCAACGACCCACCTTGCAAAGTGCACAGTTAAACAAGCAAGGACCATGATGCAATTTGAGTTATCACCGTTTGTCATGGCTGAACTTGTTAAGTTTGATGGTTCAATGCATCCACAAATACATGAGGCTTTAGTTAAATACAAACTTAGAGATTCCGTCATAATGCTCAGACCAAATGCACTTCCAAAAGTTAATTTACACAATTGGCTTACAGCCCGAGATTATAATAGAATAGGTTGCTCATTGGAACTTGAAGATCACGTTAAAATTCCGTACTATATTAGGGGAGTTCCTGATAAGTTGTATGGAAAAATATATGATATTATTCTACAGTATAGTCCAACTAGTTGTTATGGTAGACTATCAAGTGCGTGTGCGGGTAAAGTAGCATACACTCTGCGAACTGATCCGTGTTCACTTCCAAGAACAATAGCAATAATTAATGCTTTAATCACGGAAGAATATGCGAAGAGAGATCACTACCGAAACATGATTTCAAACCCCTCCTCATCACATGCATTCTCACTCAATGGGTTGGTCTCCATGATCGCAACTAGATATATGAAAGATCATACAAAAGAGAACATTGACAAACTCATTAGAGTGCGTGATCAATTACTTGAGTTTCAAGGTACTGGAATGCAATTTCAAGATCCGTCAGAACTCATGGAAATTGGGGCTCTCAATACAGTCATTCACCAAGGAATGGATGCAACTGCGGCTTGTATCGGATTACAAGGACGATGGAATGCTTCGCTCATACAACGTGATCTCTTAATTGCAGGAGGAGTTTTCATCGGAGGCATTTTGATGATGTGGAGCCTATTCACTAAATGGAGCAACACAAATGTCTCACATCAAGGAAAGAACAAACGCAGTAGACAAAAACTCCGATTCAAAGAAGCAAGAGATAATAAATATGCATATGATGTTACAGGATCGGAAGAGTGTCTTGGTGAGAATTTCGGAACAGCTTATACAAAGAAGGGTAAAGGAAAAGGAACTAAGGTTGGACTCGGTGTGAAGCAGCACAAATTTCATATGATGTACGGTTTTGATCCTCAAGAGTACAACCTAATTCGATTTGTTGATCCACTCACAGGAGCAACTCTCGATGAACAAATTCATGCCGATATGCGCTTAATTCAAGAGCATTTCGCTGAAATTCGTGAGGAAGCAGTAGCTAATGATACAATTGAAAGGCAGCAGATATACGGCAATCCTGGACTACAAGCATTTTTCATACAAAACGGATCAGCAAACGCTCTGAGAGTTGATTTAACGCCACACTCACCTACACGAGTTGTTACAGGCAACAACATAGCAGGATTCCCAGAATATGAAGGCACACTCCGTCAAACTGGAACAGCTATAACCATACCCATTGGTCAAATCCCAATTGCAAATGAAGCAGGAGTTGCACATGAATCAAAATCTATGATGAATGGACTGGGTGATTACACGCCAATATCGCAACAATTGTGTTTAGTGCAAAATGACTCAGATGGAGTAAAACGAAATGTATTTTCAATTGGATATGGCTCATATCTCATTTCACCAGCACACTTATTCAAATATAACAATGGTGAAATTACAATTAGATCATCAAGAGGATTGTATAAAATTCGTAATTCTGTGGATTTAAAATTACATCCAATTGCACACAGAGACATGGTCATAATCCAACTCCCAAAGGATTTCCCACCGTTCCCAATGCGTTTGAAATTTACACAACCATCACGAGAAATGCGAGTTTGCTTAGTAGGAATCAATTTCCAACAGAACTATAGCACTTGCATCGTATCAGAAAGCAGCGTAACAGCACCAAAGGGGAATGGAGATTTTTGGAAGCATTGGATATCAACAGTCGACGGTCAATGTGGATTACCATTGGTAGATACTAAGAACAAACACATTGTTGGAATCCATAGTCTTGCATCCACAAGTGGGAACACCAATTTCTTTGTTGCCATGCCTGAGAACTTTAATGAATACATCAATGGACTTGTGCAAACAAACAAATGGGAGAAGGGATGGCACTATAATCCGAATCTCATATCTTGGTGTGGATTAAACTTAGTCGACTCTGCTCCAAAGGGCTTGTTTAAAACGTCAAAATTGGTCGAAGATTTGGATGCTAGTGTTGAAGAGCAGTGCAAAGTTACTGAAACATGGCTCACAGAGCAATTACAAGATAATTTGCAAGTGGTCGCGAAATGTCCAGGTCAACTCGTCACTAAGCATGTTGTCAAAGGCCAATGCCCGCATTTCCAGTTATACTTGTCAACACATAACGATGCCAAAGAATATTTCGCACCCCTACTTGGAAAATATGATAAGAGTAGACTCAATAGAGCGGCATTTATCAAAGACATATCAAAGTATGCAAAACCAATTTATATTGGAGAAATCAATTATGACATCTTTGATAGAGCCGTACAACGAGTCGTTAATATTCTTAAAAATGTTGGAATGCAACAATGCGTTTATGTTACAGATGAAGAAGAAATTTTCAAATCACTTAATCTAAACGCAGCTGTCGGAGCACTCTACACAGGAAAGAAGAAAGATTACTTTGAAAGTTTTTCAAATGAAGACAAGGAAGAAATCGTGATGAGATCATGTGAACGCATTTACAATGGACAACTTGGCGTGTGGAATGGGTCACTCAAAGCTGAAATCAGGCCAATAGAGAAAACCATGTTAAATAAAACTCGAACTTTTACAGCAGCTCCATTGGAAACTTTGCTTGGAGGAAAAGTGTGCGTGGATGATTTTAATAATCAATTTTATTCACACCATTTGGAAGGCCCGTGGACTGTTGGGATAACAAAATTCTATGGAGGTTGGAATCGCTTACTTGAGAAGTTGCCAGAAGGATGGGTTTACTGCGATGCTGACGGATCCCAATTCGATAGTTCATTAACACCATATCTCATTAATGCAGTGTTGAATATTCGACTACAGTTCATGGAAGATTGGAACATAGGAGCGCGAATGCTTAAAAACCTTTATACTGAGATTGTTTACACACCAATTGCAACGCCAGATGGATCTATCGTGAAGAAATTCAAAGGAAACAATAGTGGACAACCTTCTACAGTAGTTGATAATACATTGATGGTCATAATAGCTTTCAACTATGCTATGCTATCGAGCGGTATTAGAGAAGAAGAGATTGATAACTGCTGTAGGATGTTTGCAAATGGTGATGACCTGCTCCTAGCAGTGCATCCCGATTTTGAATACATCTTAAACGGTTTTCAAAATCACTTCGGAAATCTCGGATTAAATTTTGAGTTTACATCACGAACACGAGATAAATCCGAACTGTGGTTCATGTCTACAAGAGGAATCAAATGTGAAGGAATCTACATACCTAAACTTGAGAAAGAAAGAATAGTCGCAATACTTGAGTGGGATCGATCAAATCTACCTGAGCATAGATTGGAAGCCATTTGTGCAGCTATGGTTGAAGCATGGGGCTACTCAGATCTTGTTCATGAAATTCGGAAATTTTATGCGTGGCTTCTAGAAATGCAACCTTTCGCAAATCTAGCAAAAGAAGGCAAGGCGCCATACATAGCAGAAACAGCGCTCCGTAACCTTTATCTTGGAACGGGCATTAAAGAAGAAGAAATCGAGAAATATTTTAAACAATTTGTTAAGGATCTTCCTGGATACATAGAGGATTACAATGAAGAGGTTATCCACCAATCGGGAACAGTTGATGCAGGTGCACAAGGAGGTAGCGGAAACCAAGGAACAACACCACCAGCAACA---------------------------------------------------GGTAGCGGAAC----AGGAACCAGAACTGGTGCAACTGGAGGCCAAACAGGA---------GCT---G---GTAGTGGTGCTGGAACAGGATCTGGAGCGACCGGAGGCCAATCAGGATCTGGAAGTGGCACTGGACAGACTGGCACAGGCTC---AGCAGGAACTGGTGCA------------ACGGGAGGCCAGAGAGATAAGGATGTAGATGCAGGTACAACAGGAAAAATTTCTGTACCAAAGCTCAAGGCCATGTCAAAGAAAATGCGCTTACCGAAAGCAAAAGGAAAAGATGTCTTGCATCTGGATTTCTTGCTTACATACAAGCCACAGCAGCAAGACATATCAAACACAAGAGCAACTAAGGAGGAGTTTGATAGATGGTACGATGCCATAAAGAAAGAATACGAGATTGATGACACACAAATGACAGTTGTCATGAGCGGTCTTATGGTGTGGTGCATTGAGAACGGTTGCTCACCAAACATAAACGGAAATTGGACAATGATGGATGGAGATGAACAAAGAGTCTTTCCACTCAAACCAGTCATTGAAAACGCATCTCCAACTTTCCGACAGATTATGCATCATTTTAGTGATGCAGCTGAAGCGTACATAGAGTACAGAAACTCTACTGAGCGATACATGCCAAGATACGGACTTCAGCGCAATCTCACCGACTATAGCTTAGCACGGTATGCATTTGATTTCTATGAAATGACTTCACGCACACCTGCTAGAGCTAAAGAAGCCCACATGCAG---ATGAAAGCCGCAGCAGTTCGTGGTTCAAACACACGACTGTTCGGTCTGGACGGAAATGTCGGCGAGACCCAGGAGAATACAGAGAGACACACCGCTGGCGACGTTAGTCGCAATATGCACTCTCTGTTGGGAGTGCAGCAGCACCACTAGTCTCCTGGAAACCCTGTTTGCAGTACCTATAATATGTACTA------ATATATAGTATGTCAGTGAGGTTTTACCTC-----------------------GTTTTTACTA-TTTGTTATGTATGTATTTAAAGCGTGAACCAGTCTGCAGCATACAGGGTTGGACCCAGTGTGTTCTGGTGTAGCGTGTACTAGCGTCGAGCCATGAGATGGACTGCACTGGGTGTGGCTTTGCCACTTGTGTTGCGAGTCTCCTGGTAAGAGACAA--------------

>MH093724

-----------------------------------CAACACAACACAACAAAACATAGCCAAACAAAACCAAGTTACTTTTGCTCAGATTGTAGTGAACGGCTCGGCAGGAAAGGTTCCTCGAGATCACTCTCTGACTTCTCTTTC----------TCAACCAACTTCTTTCAAGCGAGATGGCAGGCTCTTGGACTCACGTGACATACAAGTGGCAACCAGATGTCAACAACACACGCGATGTCAAAAGAGTGATGGAGATGTTTGCAGCAAAACATCAACATTACACTGAGGAGCAAAGGCTTGCTCACAACAGCAAGCTATTGAGAAGGGCTCATGTAACTAGTGCTGAGTTAATTGAACCAGCACAGAAACCGAAATGTCACCAGACGTGGGTTGAAAAGTGCGACCACAACCCCACAGAGCACTTCATTTATCAACGTTTC---ACACCTGAAAAGAAAGTGCTTAACACCAAGCCTGAGACAACCTCCGTCACGAAATTGATCAGGGATGTCCTTGAAATTTCGAAGGGTAGTGGAATCAAAATTGAGTTAATTGACAAGCGCGTCAAACGAAAGACTCAACTATCCATAAGGCGATACAATGGCAAGGATTTCTTGCATTGTAAGACCAGGCATGAGAATGGCTTGTTCAAACGCAAGGATATCGATGTTAGCCTCAAATGGTTGCCCACCATTGAAGCTATTGCGAAATGCTACAGCACGGTGAATGCAGAAGAACTACAAAGTCTCAATAGAGGCAGCAGTGGTCTCACATTCATACAGAACGGTGAATTGTTTATCGTGCGTGGACGGATGCATGGTGAGATAGTTAATAGTCTGCACGAAAGCAAGCATGTGATGGAAATAGAACACTATGCTGATCCACAAGCGAATAGTTTCTGGAAAGGCTATACAGATGCGTATGTTGAGAACAGAAACATATCCATCACTCATACAGAGCACACACCAACTATTAATTTAGAGGAGTGTGGTAAGAGGATGGCACTGTTAGAAATCCTATTCCATTCAACTTTCAAAATTACATGCAAAACGTGCAATATTGATGATCTGGAATTATCAGATGATGAATTTGGGGCCAAGTTATACAGCAATCTGCAGCGTATTGAAGAAAAGCAACGCGAGTATCTCGCTAAAGATCAAAAACTTCTACGCATGATTCACTTTGTCAAAGATCGGTGTAACCCAAAATTTTCACATTTGCCTTTACTATGGCAAGTGGCTGAAACAGTAGGACATTACACTGACAACCAATCGAAGCAGATAATTGATATCAGTGAGGCGCTCATCAAAGTTAATACTTTAACTCCTGATGATGCAGTGAAGGCCAGTGTAGCATTATTGGAAGTAGCACGGTGGTATAAGAATCGGAAGGAATCACTTAAAACGGACACATTGGATTCATTTCGAAACAAGATCTCACCAAAGAGCACAATCAATGCAGCATTAATGTGTGATAACCAGTTAGATAAGAATGCAAATTTCGTGTGGGGAAACAGAGAATACCATGCAAAGCGATTCTTTGCTAATTATTTTGAAGCTGTGGACCCAACTGATGCGTATGAAAAACACGTGACACGCTTCAATCCCAACGGACAGCGAAAATTATCAATTGGCAAGCTAGTAATCCCACTAGATTTCCAGAAGATCAGAGACTCGTTCGTTGGCCTATCAATAAATAAACAACCACTGAGCAAAGCTTGCGTAAGTAAAATTGACGGAGGTTATGTATATCCATGTTGTTGTGTAACAACGGAATTTGGAAAACCAGCATATTCTGAGATAATACCTCCAACGAAAGGACACATCACGATTGGAAATTCAGTGGACCCAAAAATAGTGGACTTACCGAATACAACACCACCGAGTATGTACATTGCAAAAGATGGATATTGTTACATTAACATATTCTTGGCAGCAATGATAAATGTCAATGAGGAGTCCGCAAAGGATTACACTAAGTTTCTTAGAGATGAGTTGGTGGAACGGCTTGGTAAATGGCCAAAACTGAAAGATGTGGCCACAGCATGCTATGCTTTATCAGTGATGTTTCCAGAGATAAAGAATGCTGAATTACCACCAATACTAGTTGATCATGAGAGTAAGTCGATGCACGTCATTGATTCATATGGATCACTCAGTGTTGGATTTCACATTCTAAAGGCAAGTACTGTTGGACAACTAATAAAATTTCAGTATGAGTCATTAGAAAGCGAGATGCGCGAGTACATAGTGGGTGGCACTTTAACACAGCAAACTTTCAGTACACTCCTTAAGACTCTTACAAAGAACATGTTTAAGCCAGATAAAATAAAGCAGGTAATTGAAGAAGAGCCATTCTTATTAATGATGGCAATTGCATCCCCAACTGTACTCATCTCGCTGTACAACAACTGCTACATCGAGCAAGCAATGACATATTGGATCGTCAAGAACCAAGGCATCGCAGCACTCTTTGCGCAGTTGGAGGCACTAGCGAAGAAAACTTCTCAAGCGGAACTATTAGTTCTTCAAATGCAAATACTTGAGAAAGCTTCAAACCAACTGAGACTTGCAGTCACGGGGCTTAATCATGTTGATCCAGCAAAACGACTTCTATGGTCTCACCTGGAAGCTATGACAACACGGTCAGAGATGAACAAGGAACTCATAGCGGAAGGTTATGCACTGTACGACGAGCGCTTATACACTTTAATGGAAAAAAGTTACGTAGATCAATTAAACCAATCATGGGCAGAATTATCATACTGTGGAAAATTTTCAGCAATATGGCGTGTGTTCAGAGTCAGGAAATACTACAAACCGTCCTTAACCGTGAGAAAAAGCGTAGATTTAGGCGCTGTGTACAATATATCAGCTACGCATCTAATATCAAATTTAGTGCAGAAAAGTCGAGATCAAGTCAGCTCTACTTTAACCAAACTCCGCAACGGTTTCTATGATAAAATGGAGAGAGCGAGAGTTGGTGCAGTAAGGACAATATATTGGTTCGTACCTGATATATTTAGACTAATTCATATTTTCTTAGTTTTAAGTATATTAACAACTATAGCTAATACAATAGTCGCAACTATGAATGATTATAAAAAGTTGAAAAAGCAACAAAGAGAAGACGAATACGAAGCCGAGATTAATGAGGTACGAAGGATACACGCAAACCTGATGAAGGAGCATAATGATAATTTAACATGTGATCAATTTATTGAATACATACGACAGACACATCCACGCCTCATTGAGGCAACACTGGACTTAACACATACAGGTGTCATCCATGAGGGTAAATCCAATTTGGAAACAAACCTTGAACAGGCAATGGCAGTGGGAACTTTACTCACTATGATACTCGATCCACAGAAGAGTGACGCAGTTTATAAGGTTCTTAATAAGATGCGTACAGTGATCAGTACAATAGAACAGAATGTGCCATTTCCCTCAGTAAACTTCACGAGCATCTTGACACCTCCTGTAACTCAGCAAAGTGTAGATGTTGACGAACCGTTAACACTGAGTACTGATAAGAATTTGACTATAGATTTCGACACAAATCAAGATTTGCCAGCGGACACATTCAGCAATGACGTTACATTCGAGAACTGGTGGGCCAATCAGATAAACAACAACAGAACAGTGCCACACTACCGACTCGGGGGAAAGTTTGTGGAATTCACAAGAGAAAATGCAGCAATGGTTAGCATTGAGCTTGCCCATTCGAACATCGAAAAAGAGTTTCTACTCAGAGGAGCTGTTGGGTCAGGAAAATCCACAGGTTTGCCGTATCATCTTAGTATGCGTGGAAAAGTGCTACTAATAGAACCCACTCGGCCATTAGCTGAGAATGTTTGTAGGCAATTGCAAGGTCCTCCATTTAATGTGAGCCCCACTTTACAAATGAGAGGATTAAGCACATTTGGCTGCACTCCTATCACGATAATGACATCTGGCTTCGCATTGCACATGTATGCTAATAACCCCGATAAGATCTCTGAATACGACTTCATCATCTTTGATGAATGTCACATTATGGAAGCACCTGCAATGGCATTCTATTGTTTGCTTAAGGAGTATGAATACCGAGGCAAGATAATAAAAGTTTCAGCCACACCACCAGGACGAGAGTGTGAATTTTCAACTCAACATCCAGTAGATATACATGTATGTGAGAGCTTGACACAACAGCAATTTGTCATGGAACTAGGAACAGGATCAACTGCTGACGCAACCAAATACGGTAATAACATATTAGTGTACGTCGCGAGTTATAATGACGTAGATTCTTTGTCCCATGCTCTAACTGAACTTAAATATTCAGTGATTAAAGTCGACGGAAGAACAATGAAGCAGAACACCACAGGAATTGTAACAAATGGAACATCCAGTAAGAAGTGCTTCGTTGTGGCTACAAACATTATTGAAAACGGTGTAACGCTAGATGTCGATGTTGTTGTTGACTTTGGGCTTAAAGTAACAGCTGAATTAGATGTTGATAACAGGGCGGTAATGTATAAACGTGTGAGCATATCTTATGGCGAGCGCATTCAGAGACTTGGAAGAGTCGGAAGGAATAAGCCTGGAACAGTTATCCGCATCGGGAAAACAATGAAAGGCTTACAAGAAATTCCGGCAATGATTGCCACTGAAGCAGCTTTCATGTGTTTTGCATATGGACTGAAGGTTATAACACACAATGTTTCAACAACACATCTAGCAAAATGCACTGTCAAACAAGCTAGAACCATGATGCAATTTGAGCTATCACCATTTGTAATGGCTGAGTTGGTTAAATTTGACGGTTCTATGCACCCACAGATTCACGAAGCGTTAACCAAGTATAAATTGAGAGATTCTGTGATCATGTTGAGACCGAATGCAATACCAAAGGTTAATCTTCACAACTGGCTGACGGCTCGTGATTATAACAGAATAGGCTGCTCATTAGAACTTGAAGACCACGTTAAAATACCATATTATGTACGGGGAGTTCCTGACAAGTTGTATGGGAAGTTATATGATATTATCCTTCAATATAGCCCTACAAGTTGCTATGGAAGACTATCAAGTGCTTGCGCAGGCAAGGTTGCATATACATTGCGCACTGATCCTTGTTCATTACCAAGAACAATAGCTATAATCAACGCACTAATTACTGAAGAGTACGCAAAGAGGGATCATTATAGAAACATGATAGCGAACCCCTCATCATCGCACGCCTTTTCACTCAATGGACTAGTATCCATGATTGCTTCTCGGTACATGAAAGATCACACGAAGGAAAATATTGAAAAACTCGTAAGAGTGCGCGATCAACTACTTGAATTCCAAGGCACAGGGATGCAGTTTCAAGATCCTTCGGAAATAATGGACATCGGTGCATTAAACACAGTTATTCACCAAGGAATGGATGCCACGGCTGCTTGCATTGGATTACAAGGGCGTTGGAATGCTTCACTCATTCAACGTGATTTGATGATATCAGCAGGGGTTTTCGCAGGGGGCATTCTTATGATGTGGTGTCTTTTCACAAAATGGAGTAAGACAGAAGTATCACACCAAGGAAAGAACAAGCGAAGTCGGCAGAAACTACGATTCAAGGAGGCTCGTGATAACAAATACGCCTATGATGTAACAGGGTCAGAAGAAGTTCTTGGTGAGAATTTCGGAACTGCTTATGTTAAGAAAGGAAAAGGAAAAGGAACAAAAGTTGGTCTTGGAGTCAAACAACACAAATTCCACATGATGTATGGTTTTGACCCACAAGAATACAACTTAATCCGTTTCGTTGATCCTTTAACAGGTGCAACACTAGACGAGCAAATTCATGCAGATATACGTCTAGTGCAAGAACACTTTAGTGTCATTAGAGATGAAGCAGTGGCAAATGACACAATTGAAAGACAACATATTTACAGCAATCCTGGACTACAAGCGTTCTTCATACAAAATGGATCGGCAAATGCACTGAGAGTTGACTTAACACCGCACGCACCACTGCGTGTCGTGACAAATAACAATATAGCAGGTTTCCCAGAATATGAAGGCACACTCCGACAAACAGGAACGGCCTTACAAATACCTGTTAATCAAGTTCCAGCTGCGAATGAAACAGGAGTGGCGCATGAGTCAAAATCAATGATGGCAGGGCTAGGTGATTACACTCCCATATCACAGCAATTATGCTTAATCCAAAATGACTCTGACGGAATTAAAAGGAATGTCTATTCAATCGGATATGGGTCATATCTCATTGCACCAGCACATTTATTTAAATATAACAATGGTGAGGTGACGATCAAATCATCACGAGGCTTGTATAAAATCAGAAACTCAGTCGAAATTAAGTTACACCCCATTGCACATAGAGATATGGTTATAATTCAACTTCCAAAGGATTTTCCACCATTCCCAATGCGCCTTAAATTTTCCAAACCATCTAGAGAGTCAAGAGTGTGCTTGGTTGGAGTGAATTTCCAACAAAACTACAGCACATGCATTGTGTCGGAGAGTAGCGTTACAGCACCAAAAGGCAACGGAGATTTCTGGAAACACTGGATATCCACAGTGGACGGACAATGTGGCCTCCCATTAGTAGACGTCAAGAGCAAACACATAGTTGGAATACACAGCCTTGCATCAACTAGTGGAAACACTAACTTCTTCGTCGCCATGCCTGAAGATTTCAACGATTACATTCACAATCTTGTGCAAACCAACAAGTGGGAAAAAGGATGGCATTATAACCCGAATCTCATTTCGTGGTGTGGTCTCAATCTAGTTGATTCAGCTCCAAAGGGTCTCTTCAAAACTTCGAAATTAGTGGAAGACCTTGATATGAGTGTCGAAGAGCAGTGTAAGGTTACAGAGACATGGCTCACAGAATGCATTCAGGATAATTTACAGGTTGTTGCAAAATGCCCAGGTCAACTTGTCACCAAGCACGTCGTTAAAGGCCCATGCCCACACTTTCAGCTATATCTGTCAACACATGATGAAGCCAAAGCATACTTTGCACCATTGCTTGGAAAATACGATAAGAGCAGATTAAACAGAGCAGCTTTTATCAAAGACATTTCAAAATATGCAAAACCAATCTACATCGGAGAAATCAATTACGATATCTTCGAAAAGGCTATACAACGTGTGATTAAAATTCTTAGAGATGTGGGAATGCAACAATGCACGTATGTCACGGACGAGGATGAAATATTCCAATCACTTAACCTCAATGCCGCAGTTGGTGCCTTATACACAGGAAAGAAGAAAGATTATTTCAAGGATTTCTCAAACGAGGACAAATCAGAAATCATTATGAGATCCTGTGAGCGTATCTACAACGGACAACTCGGTGTGTGGAATGGTTCACTCAAAGCTGAAATAAGGTCCATTGAGAAAACTATGTTAAACAAGACTCGGACTTTCACAGCAGCACCATTAGAAACTCTACTTGGTGGCAAAGTCTGCGTCGACGATTTCAATAATCAATTCTACTCACACCACTTAGAAGGGCCTTGGACGGTCGGAATCACAAAATTTTATGGTGGGTGGAACCGTTTGTTGGAAAAATTGCCAGATGGTTGGATATACTGCGATGCCGATGGATCACAGTTTGACAGCTCTTTAACACCATACCTTATTAACGCTGTACTACATATTCGGCTACAATTCATGGAGGAATGGAACTTAGGAGCACAAATGTTGAGAAACTTATACACTGAAATTGTTTACACGCCAATTGCAACACCGGATGGATCTGTGATTAAGAAATTCAAAGGAAACAATAGCGGGCAACCATCAACAGTTGTTGACAATACACTTATGGTTATATTAGCATTTAATTATGCAATGTTATCGAGCGGTGTCAGAGAAGATGAAATAGACAACTGCTGCCGAATGTTCGCTAATGGAGACGATCTATTATTGGCAGTTCATCCGGACTTTGAACATATACTAGATGGATTTCAAGATCACTTTGGAAATCTGGGTCTCAACTTTGAATTTACATCACGAACAAAAGACAAATCAGAATTGTGGTTTATGTCCACACGAGGTATTAAATGTGAAGGAATCTATATACCTAAACTCGAGAAAGAAAGAATAGTCGCAATACTTGAATGGGATCGATCAAATCTACCTGAGCATAGATTGGAAGCCATTTGTGCAGCTATGGTTGAAGCATGGGGCTACTCAGATCTTGTTCATGAAATTCGAAAATTTTATGCGTGGCTTCTAGAAATGCAACCTTTCGCAAACCTAGCAAAAGAAGGCAAGGCACCATACATAGCAGAAACAGCGCTCCGTAACCTTTATCTTGGAACGGGTATTAAAGAAGAAGAAATCGAAAAATACTTTAAACAATTTATTAAGGATCTTCCTGGATACATAGAGGATTACAATGAAGAGGTCATCCACCAATCGGGAACAGTTGATGCAGGTGCACAAGGAGGCGGCGGTAACCAAGGAACAACACCACCAGCAACA---------------------------------------------------GGTAACGGAAC----AGGAACCAGAACTGGTGCAACTGGAGGCCAAACAGGA---------ACT---G---GTGGTGGTGCTACAACAGGATCTGGAGCGACCGGAGGGCAGACAGAATCTGGAAATGGCGCTGCACGGACCAACACGGGCAC---AACAGGAGCTGGTGCA------------ACGGGTGGCCAGAGAGATAAGGATGTAGATGCAGGTACAACAGGAAAAATTTCTGTACCAAAGCTCAAGGCCATGTCAAAGAAAATGCGCTTACCGAAAGCAAAAGGAAAAGATGTCTTGCATCTGGACTTCTTGCTTACATACAAGCCACAGCAGCAAGACATATCAAACACAAGAGCAACTAAGGAGGAGTTTGATAGATGGTACGATGCCATAAAGAAAGAATACGAGATTGATGACACACAAATGACAGTTGTCATGAGCGGTCTTATGGTGTGGTGCATTGAGAATGGTTGCTCACCAAACATAAACGGAAATTGGACAATGATGGATGGAGATGAACAAAGAGTCTTTCCACTCAAACCAGTCATTGAAAACGCATCTCCAACTTTTCGACAAATTATGCATCATTTTAGTGATGCAGCTGAAGCGTACATAGAGTACAGAAACTCTACTGAGCGATACATGCCAAGATACGGACTTCAGCGCAATCTCACCGACTATAGCTTAGCACGGTATGCATTTGATTTCTATGAAATGACTTCACGCACACCTGCTAGAGCTAAAGAAGCCCACATGCAG---ATGAAAGCCGCAGCAGTTCGTGGTTCAAACACACGATTGTTCGGTCTGGACGGAAATGTCGGCGAGACCCAGGAGAATACAGAGAGACACACAGCTGGCGACGTTAGTCGCAATATGCACTCTCTGTTGGGAGTGCAGCAGCACCACTAGTCTCCTGGAAACCCTGTTTGCAGTACCTATAATATGTACTA------ATATATAGTATGTCAGTGAGGTTTTACCTC-----------------------GTCTTTACTA-TTTGTTATGTATGTATTTAAAGCGTGAACCAGTCTGCAGCATACAGGGTTGGACCCAGTGTGTTCTGGTGTAGCGTGTACTAGCGTCGAGCCATGAGATGGACTGCACTGGGTGTGGCTTTGCCACTTGTGTTGCGAGTCTCCTGGTAAGAGACAAA-------------

>MH093725

-----------------------------------------------------------------------------------------------------------------------------------------------------------------------------------ATGGCGGGAACGTGGACCCATGTGACATACAAGTGGCAGCCAGATGTCAACAATGATCGTCACATTAAGAGAGTAATGGAAATGTTTGCAGCAAAACATCAACATTACTCAGAAGAACAGCGACTTGCTCACAATATGAAATTATTGAGGAAGACAAGTGTTGTGAGTGTTGAGCCTGCAAAACCGAAACAGAAGCAGGCAACTCAACAAATGTGGGTTGAGAAATGTGATCACAATCCTGTTGATCACTTAGTATATTCACGATTTGAAAGACCTATCAACAAAGTG---GATACGAGTATCAAAAGTGCATCTGTAAGCAAGCTAACCAGAGAGATTTTAGAGATCTCGAAGGTTAGCGGCCTTAAAGTTGAACTAATTGATAAGCGTAAAAGAGCTAAAACACAGTTATCAATCAAAAGGTTCAATGGCAAGGACTTTCTCCATTGCAAAACAAACCATGAAAACAATTTGTTTAAGAGGAGGGACATAGCCATCGGACACAAATGGCTTCCAACAATCGAAGCCATTGCTCGCTGCTATAGTACAATGAACCAAGAGGAATTGCAGAGCCTTTATAGAGGCAGCAGTGGTCTCACATTCATTCAAAATGATGAACTGTTCGTTGTTAGAGGGAGAATGAATGGTGAACTAGTCAATAGCCTATGTGAAACAGACCGGGTTATGGATATTGAACACTACGCAGATCCCCAAGCTAATGACTTTTGGAGAGGATATACAAATGCTTACGTGGATAATCGTAGCATTTCTACCACCCATACAGAGCACACTCCGACAATCAATCTAGAAGAGTGTGGAAAACGAATGGCTCTACTTGAGATATTATTCCACTCCACATTCAAGATTACATGTAAAACATGTAACATGGATGATCTTGAATTATCAGATGATGAATTTGGAGCTAAACTCTACAAAAATCTGCAACGCATCGAAGAGAAGCAACGAGAGTACCTTGCCAAGGATCAAAAGTTATCCAGAATGATACAATTTATCAAAGAAAGATGCAACCCAAAATTCTCACATTTACCAATGTTGTGGCAAGTTGCAGAAACAATAGGACACTATACTGATAATCAGTCAAAGCAAATAATGGATATCAGCGAAGCGCTCATCAAAGTTAATACTTTAACTCCTGATGATGCTATGAAAGCAAGCGCAGCATTACTCGAAGTGTCGCGGTGGTATAAGAATCGTAAGGAATCACTCAAAACTGACTCATTGGAATCTTTTAGAAATAAAATATCACCAAAGAGTACAATAAATGCAGCTTTAATGTGCGACAATCAATTGGATAAAAATGCAAATTTTGTATGGGGTAATAGGGAATATCACGCCAAACGATTCTTTGCAAACTATTTTGAAGCAGTGGATCCCACAGACGCATATGAAAAGCACGTTACACGGTTCAACCCTAATGGTCAACGAAAGTTATCGATAGGAAAGTTAGTTATTCCACTAGACTTTCAAAAGATTAGAGAATCATTTGTTGGACTTCCGATCAATAGACAACCGCTAGACAAATGTTGTGTTAGTAAGATCGAAGGAGGATATATATACCCGTGTTGCTGCGTCACAACAGAATTTGGTAAACCAGCATACTCTGAGATAATACCTCCAACAAAAGGCCACATAACAATAGGCAACTCAATTGATCCAAAGATTGTGGACCTACCAAACACAACACCACCCAGCATGTACATTGCTAAGGACGGGTACTGTTATATCAATATCTTTCTAGCAGCCATGATCAACGTCAATGAAGAATCTGCCAAGGATTATACGAAATTCTTGAGAGATGAACTAGTTGAGCGTCTTGGAAAGTGGCCAAGGCTTAAAGACGTAGCAACAGCGTGTTATGCATTATCTGTAATGTTTCCAGAAATTAAGAATGCTGAGCTACCTCCAATCTTAGTTGACCATGAAAATAAATCTATGCACGTCATCGACTCATATGGTTCACTAAGCGTTGGATTTCACATATTGAAAGCAAGCACGATTGGTCAATTAATTAAATTTCAATATGAATCTATGGATAGCGAAATGCGCGAATACATAGTAGGAGGAACTCTTACGCAACAGACATTCAATACACTTCTTAAGATGCTCACGAAAAACATGTTCAAACCAGAGCGTATTAAGCAGATAATTGAGGAGGAACCCTTCTTGCTTATGATGGCAATCGCATCTCCAACGGTATTGATAGCACTGTATAATAATTGTTACATTGAGCAAGCTATGACATACTGGATCGTGAAGAATCAAGGAGTTGCAGCCATATTCGCACAACTCGAAGCATTAGCCAAGAAAACATCCCAGGCTGAATTACTAGTTCTACAAATGCAGATACTTGAAAAAGCATCTAGTCAACTAAGATTAGCAGTTTCAGGACTTAGCCATGTCGACCCAGCAAAGCGACTTTTGTGGTCACACCTCGAAGCAATGACAACACGGTCAGAAATGAACAAGGAGTTGATAGCTGAGGGATATGCACTATACGACGAGCGTCTATATACCCTGATGGAAAAAAGTTACGTAGATCAATTAAACCAGTCATGGGCAGAATTATCATACTGTGGAAAATTTTCAGCAATATGGCATGTGTTCAAAGTCAGGAAGTATTACAAACCGTCTTTAACCGTGAGAAAAAGCGTAGATTTAGGCGCTGTATACAATATATCAGCTACGCATCTAATATCAGATTTAGTGCGGAAAAGTCAAGATCAAGTCAGCTCTATTTTAACCAAACTCCGCAACGGTTTCTATGATAAATTAGAGAAAGCTAGAATACGCACTATAAAAACGGTTTATTGGTTTATACCCGATATATTTAGACTCATGCATATATTCATAGTTTTGAGTTTATTAACTACCATAGCGAACACTATCATAGTAACTATGAACGACTACAAGAAATTGAAGAAACAACAAAGAGAAGACGAATATGAAGCAGAAATTAACGAAGTTCGCAAAATCCATTCTACCTTGATGGAAGAGCGGAAGGACAATTTGACATGTGAACAATTTGTTGAATATATGCGCCAAAACCATCCGCGGTTAGTTGAAGCAACACTGGACTTAACCCACACAGGTGTCATACATGAAGGAAAATCCAATCTCGAAACCAATTTGGAACAAGCAATGGCAGTTGGCACCTTGATAACCATGATACTTGATCCACAGAAAAGCGATGCTGTCTATAAGGTGTTGAACAAAATGCGGACAGTAATTAGTACAATTGAACAAAACGTCCCATTCCCTTCAATAAACTTTTCCAACATCTTGACACCTCCAGTGACACAACAGAGTGTAGATGTTGATGAGCCATTGACACTTAGCACTGAGAAAAATTTAACAATAGACTTTGACACGAATCAAGATTTACCTGCCGATACATTCAGTAATGATGTGACATTTGAAGATTGGTGGTCAAATCAATTAAGCAACAATAGAACAGTGCCACACTACCGACTTGGGGGAAAGTTTGTTGAATTCACACGAGAAAACGCAGCCCACACGAGCATCGAACTTGCACACTCAAACATTGAGAAAGAGTTCTTACTTAGAGGAGCAGTCGGCTCGGGAAAATCTACTGGGTTACCATACCATCTTAGCATGCGTGGAAAAGTGCTTTTACTAGAGCCTACAAGGCCACTAGCCGAGAACGTGTGTAGGCAATTACAAGGACCACCATTTAATGTAAGTCCAACTCTTCAAATGCGTGGACTGAGTTCTTTCGGGTGTACTCCAATCACGATCATGACATCAGGTTTTGCATTGCACATGTACGCAAATAATCCAGATAAAATATCTGAATACGATTTTATAATCTTCGATGAATGTCATATAATGGAAGCACCAGCAATGGCCTTTTATTGCTTACTCAAAGAATACGAATACCGAGGAAAAATCATCAAGGTATCAGCCACGCCTCCAGGAAGGGAGTGCGAATTCACAACACAACATCCAGTAGACATCCATGTTTGTGAAAATCTAACTCAGCAACAATTTGTTATGGAACTCGGAACCGGTTCAACCGCAGATGCTACGAAGTACGGAAATAATATCTTAGTTTATGTAGCAAGTTATAATGACGTCGATTCATTGTCGCATGCATTAGTTGAACTCAAATATTCCGTTATTAAAGTGGATGGCCGAACAATGAAGCAAAACACAACAGGAATCACCACAAACGGTACCTCACAAAAGAAGTGTTTTGTTGTCGCAACAAATATAATTGAAAATGGCGTCACACTAGATATCGATGTTGTTGTTGACTTTGGACTGAAAGTATCAGCCGATTTGGATGTTGACAACAGGGCAATATTGTATAAACGCGTGAGTATATCATATGGTGAACGCATACAACGACTGGGTCGTGTTGGAAGAAATAAACCTGGCACAGTTATTCGTATTGGAAAAACCATGAAAGGTTTGCAAGAAATTCCAGCAATGATCGCAACAGAAGCAGCTTTCATGTGTTTCGCATATGGCCTCAAAGTCATCACCCATAATGTTTCAACGACCCATCTTGCAAAGTGCACAGTTAAACAAGCAAGAACCATGATGCAATTTGAATTATCACCATTTGTCATGGCTGAGCTCGTTAAGTTTGATGGTTCAATGCATCCACAAATACATGAGGCTTTAGTTAAATACAAGCTTAGAGATTCTGTCATAATGCTTAGGCCGAATGCAATTCCAAAAGTTAATTTACACAATTGGCTTACAGCCCGGGATTATAATAGAATAGGTTGCTCATTAGAACTTGAAGATCATGTTAAAATTCCGTACTATATTAGGGGAGTTCCCGATAAGTTGTATGGAAAGCTATATGATATTATTTTACAGTATAGTCCAACTAGTTGTTATGGTAGACTATCAAGTGCGTGTGCAGGTAAAGTAGCATACACTTTACGAACAGATCCGTGTTCACTTCCAAGAACAATAGCAATAATCAATGCTTTAATCACGGAGGAATATGCGAAGAGAGATCACTACCGAAACATGATTTCAAACCCTTCTTCATCACATGCATTCTCACTCAATGGGTTGGTATCTATGATCGCAACTAGATATATGAAGGATCATACAAAAGAGAATATTGACAAACTCATCAGAGTACGTGATCAATTACTTGAGTTTCAAGGCACCGGAATGCAATTTCAAGATCCGTCAGAACTCATGGAAATTGGGGCTCTCAATACAGTCATTCACCAAGGAATGGATGCAACTGCAGCTTGTATCGGATTACAAGGACGATGGAATGCTTCGCTCATACAACGTGACCTCTTAATCGCAGGAGGAGTTTTTATCGGAGGCATTTTGATGATGTGGAGCTTATTCACTAAATGGAGCAACACAAATGTCTCACATCAAGGAAAGAACAAACGTAGTAGACAAAAACTCCGATTCAAAGAAGCAAGAGATAACAAATATGCATATGATGTCACAGGATCAGAAGAGTGCCTTGGCGAGAATTTCGGAACAGCCTATACAAAGAAAGGTAAAGGAAAAGGAACTAAAGTTGGACTCGGTGTGAAACAACACAAATTTCATATGATGTACGGTTTTGATCCTCAAGAGTACAACCTAATTCGATTTGTCGATCCACTCACAGGAGCAACTCTTGATGAGCAAATTCATGCCGACATACGCTTAATTCAAGAGCATTTCGCTGAAATTCGTGAGGAGGCAGTAGCTAACGACACAATTGAAAGACAGCAGATCTACGGCAATCCTGGACTACAAGCATTTTTCATACAAAATGGGTCAGCAAACGCCCTGAGAGTTGATTTAACGCCACATTCACCTACACGAGTTGTCACAGGTAATAATATAGCAGGATTCCCAGAATATGAAGGCACACTTCGTCAGACTGGAACAGCTATAACCATACCCATTGGTCAAGTCCCGATCGCGAATGAATCAGGAGTTGCACACGAATCAAAATCTATGATGAATGGATTGGGTGATTACACGCCAATATCGCAACAATTGTGTTTAGTGCAAAATGACTCAGATGGAGTAAAACGGAATGTATTTTCAATCGGATATGGCTCGTATCTCATTTCACCAGCGCACTTATTTAAATATAACAATGGCGAAATTACAATTAGATCATCAAGAGGATTGTATAAAATTCGCAACTCTGTGGATTTAAAATTACATCCGATTGCACACAGAGACATGGTCATAATCCAACTCCCAAAGGATTTCCCGCCGTTCCCAATGCGTTTGAAGTTCACACAACCATCACGAGAGATGCGAGTTTGCTTAGTGGGAGTCAATTTCCAACAGAATTATAGCACTTGTATCGTATCGGAAAGCAGCGTAACAGCACCAAAGGGGAATGGAGATTTTTGGAAGCATTGGATATCAACAGTCGACGGTCAATGTGGACTACCATTGGTAGATACTAAGAATAAACACATTGTTGGAATTCATAGTCTTGCATCCACAAGTGGAAACACCAATTTCTTTGTTGCCATGCCTGAGAACTTTAATGAATACATTAATGGACTTGTGCAAACAAACAAATGGGAAAAGGGATGGCACTATAATCCGAATCTCATATCTTGGTGTGGATTAAACTTAGTCGACTCTGCTCCAAAGGGTTTATTTAAAACGTCAAAATTAGTTGAAGATTTGGATGCTAGTGTTGAAGAGCAGTGCAAAGTTACTGAAACATGGCTCACAGAGCAATTACAAGATAATTTGCAAGTGGTTGCGAAATGCCCAGGCCAACTCGTCACTAAGCATGTCGTCAAAGGCCAATGCCCACACTTCCAGTTATATTTGTCAACACATAATGATGCCAAAGAATATTTCGCACCCCTGCTTGGAAAATATGATAAGAGCAGACTCAATAGAGCGGCATTTATCAAAGACATATCAAAGTATGCAAAACCAATTTATATTGGAGAAATCAATTATGATATCTTTGATAGAGCTGTACAACGAGTCATTAACATTCTTAAAAATGTTGGAATGCAACAATGCGTTTATGTCACAGATGAAGAAGAGATTTTTAAATCACTTAACCTAAACGCAGCTGTCGGAGCACTGTACACAGGAAAGAAGAAAGATTACTTTGAAAGCTTTTCAAATGAAGACAAGGAAGAAATCGTGATGAGATCATGTGAACGCATTTACAATGGACAACTTGGCGTGTGGAATGGGTCACTCAAAGCTGAAATCAGGCCAATAGAGAAAACCATGTTAAATAAGACTCGAACCTTTACAGCAGCTCCATTGGAAACTTTGCTTGGAGGAAAAGTGTGTGTGGACGATTTTAATAATCAATTTTATTCACACCATTTAGAAGGCCCATGGACTGTTGGGATAACAAAATTCTATGGAGGTTGGAACCGCTTACTTGAGAAGTTACCGGAAGGATGGATTTACTGCGACGCTGACGGATCCCAATTCGATAGTTCATTAACACCATATCTCATTAATGCAGTGTTGAATATTCGATTACAGTTCATGGAAGATTGGAACATAGGAGCGCAAATGCTTAAAAACCTTTATACTGAAATTGTTTATACACCAATTGCAACGCCAGATGGATCCATCGTGAAGAAATTCAAAGGGAACAATAGTGGACAACCTTCTACAGTAGTTGATAATACATTGATGGTTATAATAGCTTTCAACTATGCTATGCTATCGAGCGGTATTAGAGAAGAAGAGATTGATAACTGCTGTAGGATGTTTGCAAATGGTGATGACCTGCTCCTAGCAGTGCATCCTGATTTTGAATACATTCTAAACGGATTTCAAGACCACTTCGGAAATCTCGGATTGAATTTTGAGTTTACATCACGAACACGAGATAAATCCGAATTGTGGTTCATGTCTACAAGAGGAATCAAATGTGAAGGAATCTACATACCTAAGCTCGAGAAAGAAAGAATAGTCGCAATACTCGAATGGGACCGATCGAACTTGCCTGAGCATCGTCTTGAAGCTATCTGTGCAGCCATGGTTGAAGCATGGGGTTACCCAGATTTAGTTCATGAAATTCGAAAGTTTTACGCGTGGCTTCTTGAAATGCAACCTTTCGCGAATCTGGCAAAGGAAGGCATGGCGCCATACATAGCAGAAACAGCACTCCGCAACCTCTACCTTGGAACAGGCATCAAAGAAGAAGAAATTGAAAAATATTTTAGACAGTTTGTCAAGGATCTACCTGGATACGTAGAAGATTACAACGAAGAAGTTATTCATCAATCTGGTCAAGTTGACGCAGGGAGACAGGGCGGTAGCGGCGCTCAAGGAGGCACGCCACCAGCAGGA---------------------------------------------------AGTGGAGGCAC----TGGATCTGGCACTCAAGGCAATGGGGGTCAGACGGGA---------TCCCAAG---GAAGTAGTGGTCAACAAGGGTCCGGTGGGGGCACTGGTCAAGGAGCAGCTGGAAACAACGGCGG------------AGGTCAGACAGGAGGCTCTAGTGGG------------ACAGCTGGTCAGAGAGATAAGGACGTTGACGCAGGCTCGGCTGGAAAGATATCCGTACCAAAGCTTAAAGCCATGTCAAAGAAAATGCGCTTGCCAAAGGCAAAAGGAAAAGATGTCTTGCATTTGGACTTTTTGTTGACATACAAGCCACAACAGCAGGACATATCGAACACAAGAGCAACTAAGGAAGAGTTCGATAGATGGTACGACGCCATAAAGAAGGAGTACGAGATCGATGATACACAAATGACAGTCGTCATGAGTGGTCTGATGGTCTGGTGCATCGAAAATGGTTGCTCACCAAACATAAACGGAAATTGGACGATGATGGATGGGGATGAACAAAGAGTTTTCCCACTAAAACCAGTTATCGAAAACGCATCTCCAACTTTTCGACAAGTTATGCATCATTTCAGTGATGCAGCTGAAGCGTATATAGAATACAGAAATTCTACTGAGCGATACATGCCAAGATATGGACTTCAGCGAAATCTCACCGACTATAGCTTAGCGCGGTATGCTTTTGATTTCTACGAAATGACTTCACGCACACCAGCTAGAGCTAAGGAAGCCCACATGCAG---ATGAAAGCCGCAGCAGTTCGTGGTTCAAACACACGACTGTTCGGTTTGGACGGAAATGTCGGCGAGACTCAGGAGAATACAGAGAGACACACAGCTGGCGACGTTAGTCGCAATATGCACTCTCTGTTGGGAGTGCAGCAACACCACTAGTCTCCTGGAAACCCTGTTTGCAGTACCTATAATATATACTA------ATATATAGTACGTTGGTGAGGCTTTGCCTC-----------------------GGTTCTACTATCTTATTATGTATGTATTTACAGCGTGAACCAGTCTGCAGCATGCAGGGTTGGACCCAGCGTGTTCTGGTGTAGCGTGTACTAGCGTCGAGCCATGAGACGGACTGCACTGGGTGTGGCTATGCCACTTGTGTTGCGAGTTTCCTGGTAAGAGACAAAAAAA---------

>MH093729

--------------------------------------------------------------------------------TTCTCAGATTGTAGTGAACGGCTCGCAAGAAACGGTTCTTCGAGATCACTCTCTGATTTCTTTCTC----------TCTCACACTTGCATTCAAGCGGAATGGCGGGATCGTGGACTCACGTGTCTTACAAGTGGCAGCCAAATGTCAACAATGACCGTGATGTAAGAAAGGTAATGGAAATGTTTGCAGCAAAACATCAACATTATACAGAGGAGCAGCGACTTGCACATAACAGCAAGTTGCTAAGGAAAGCGTATGTTATGGACGTTGAACCAGTGAAGCCAGCACCGGAGCCTATAAGGCGTAAAGTGTGGGTGGAAAAATTCGACCACAACCCAACCGAAGACCTGGTGTATCCGCGCCTT---GTCACGGTTAAAAAGGCAGCAGAAATGAAGCCCGTGAACACCTCTATAAATAAACTTATAAGGGATGTTCTGGACATATCAAAAGGGAGCAGCCTTAAACTTGAGCTAATTGGTAAACGCCAGAAGTGCAGAACACAACTAGCTATTAAGAAATACAACAATAAGGACTACCTCCATTGCAGGACACGTCATGAAGACAACATGTTCAAGAGGAGGGACGTGGCAATCGGCATTGAATGGATCCCAACCATTGAAGCGATTGCTCGATGCTACAGCACAGTAAACAAACAGGAGATGCAAAGTCTCTACAAAGGCAGTAGTGGCTTAACATTCATGCAAAATGATGAATTATTCATTGTTAGAGGCAGAATGAATGGCGAGCTAGTTAATAGCCTGGAAGAGAACAAGAACGTGCTCGACATCGATCACTACGCTGATCCACAAGCGAACGACTTTTGGAAAGGGTACACTGATGCATACGTTGCGAATCGCAGTATCTCCACTACACATACCGTGCATACTCCAACAATCAACTTGGTAGAGTGTGGAAAGAGAATGGCATTACTTGAAATTTTATTCCACTCAACTTTTAAGATTACATGCAAGACATGCAATATTGATGACTTGGAGTTATCAGACGATGAATTTGGAGCAAAACTTTTCAAGAATCTACAACGTATCGAAGAACAGCAACGCGAATATCTCGCTAAAGATCAAAAGCTGCGTCGCATGATTCAATTCATCAAAGAAAGGTGTAATCCAAAGTTCTCTCATCTACCATTGCTTTGGCAAGTTGCAGAAACAATAGGACATTATACGGACAATCAGTCAAAGCAAATAATTGATATCAGTGAAGCACTCATCAAAGTGAACACATTAACACCTGATGATGCAGTCAAGGCGAGCGTAGCACTGCTTGAGGTGGCACGATGGTATAAAAATAGGAAAGAATCACTTAAAACAGACACATTAGATTCGTTCCGAAACAAGATATCCCCAAAGAGTACGATAAACACAGCATTGATGTGTGATAATCAACTAGACAAGAATGCAAATTTTGTCTGGGGAAACAGGGAGTATCATGCAAAGCGCTTCTTCTCAAATTACTTTGAAGCAGTAGATCCTACTGACGCATACGAGAAGCACGTAACCCGTTTCAATCCAAATGGTCAACGGAAATTATCAATTGGTAAACTAGTAATTCCGTTAGATTTCCAGAAAATAAGAGACTCATTCGTTGGTTTGGCAATAAACAAACAGCCACTTACAAAGGCTTGTGTGAGCAAAATCGATGGTGGTTATGTATACCCATGCTGTTGTGTCACAACAGAATTTGGAAAACCAGCATACTCTGAGATAATACCACCGACAAAGGGCCATATAACAATTGGAAATTCTGTAGACCCAAAGATCGTAGACCTGCCAAATACAACACCACCAAGTATGTACATTGCCAAAGACGGATATTGTTACATCAACATTTTCTTAGCAGCAATGATAAATGTCAATGAAGACTCAGCAAAGGACTACACAAAATTCCTCAGGGATGAATTAGTTGAGCGACTTGGAAAGTGGCCGAAACTTAAAGATGTTGCAACAGCGTGCTATGCGTTATCAGTTATGTTTCCAGAGATTAAGAACGCTGAATTGCCTCCAATACTCGTTGACCACGAAAGCAAGTCAATGCACGTGATTGATTCATACGGCTCATTGAGCGTTGGATTCCACATCCTTAAAGCAAGCACTGTTGGCCAGCTCATAAAATTTCAATACGAGTCAATGGACAGTGAGATGCGCGAGTATATAGTGGGAGGTACTTTAACGACCCAAACATTTAACAAACTCCTTACATCTTTAGCTAAAAATATGTTCAAACCAGATCAAATTAAGCAGATGATTGAGGAGGAACCCTTCCTACTAATGATGGCAATCGCGTCACCAACTATGCTTATAGCGCTATACAATAATTGCTATATAGAGCAGGCTATGACGTATTGGATCGTTAAAAACCAAGGAGTGGCAGCAATATTCGCACAACTGGAAGCACTAGCAAAGAAGACCTCACAAGCTGAATTGTTAGTCCAACAAATGCAAATACTTGAAAAGGCGTCTAATCAATTGAGGCTTGCAGTCACGGGTTTAAACCATGTCGATCCAGCCAAGCGTCTGCTATGGTCGCATCTAGAAGCAATGACAACACGATCAGAGATGAACAAGGAATTAATTGCTGAAGGCTATGCCCTGTATGATGAGCGCCTGTATGCACTTATGGAAAAAAGTTACGTAGATCAATTAAACCAATCATGGGCAGAGTTATCATTCTGTGGAAAATTTTCAGCAATATGGCGTGTGTTCAAAGTCAAGAAATATTACAAGCCGTCTTTAACCGTGAGAAAAAGCGTAGATTTAGGCGCTGTGTACAATATATCAGCTACGCATCTAATATCAGATTTAGCGCAGAGAAGTCGCGATCGAGCCAGCTCTATTTTAACCAAACTCCGCAACGGTTTTTATGATAAGTTAGAGAAGGCTAGAACTCGAGCGATTAAAACTGTTTACTGGTTCATACCTGACATATTTAGACTTATGCATATTTTCATAGTTCTTAGTTTATTAACAACTGTGGCAAACACTATCATTGTGACTATGAATGATTACAAAAAGCTGAAAAAGCAACAAAGAGAGGATGAGTATGAAGCTGAAATTAACGAGGTTCGAAAAATTCACGCTAATCTGATGAAAGAGCATAATGACAATCTAACATGTGAACAGTTTATCGAACATATGCGCCAGACGCATCCACGATTAATTGAAGCTACATTGGAGTTGACACACACAGGCGTCATTCATGAAGGAAAGAGCAACCTAGAAACCAATCTCGAGCAAGCTATGGCAGTGGGCACTTTACTCACAATGATGTTAGACCCACAGAAGAGTGATGCAGTATACAAAGTGTTGAATAAGATGAGAACTGTAATTAGCACATTCGAGCAGAATGTCCCATTTCCTTCAATCAACTTTACAAATATTTTAACACCATCAGTGGCGCAACAAAGTGTAGATGTTGATGAACCTCTAACTTTAAGTACTGACAAGAATCTAACAATAGATTTTGATACTAATCAAGATTTACCAGCGGACACGTTTAGCAATGATGTCACTTTCGAAGATTGGTGGGCAAATCAAATAAGCAACAATAGAACAGTTCCACATTATCGGCTTGGTGGCAAATTTGTAGAATTCACAAGAGAAAATGCAGCACATGTTAGCATTGAGCTCGCACACTCAAATCTTGAGAAGGAGTTTCTACTCAGAGGAGCTGTTGGCTCAGGAAAATCCACTGGTCTTCCATATCATCTTAGTATGCGTGGTAAGGTGCTTTTAATAGAACCAACAAGACCGCTAGCCGAGAATGTCTGCAGACAGCTTCAAGGACCACCATTCAATGTCAGCCCGACACTACAAATGCGTGGTTTAAGCTCTTTTGGTTGCACGCCAATTACGATAATGACATCAGGTTTCGCACTGCACATGTATGCAAATAATCCGGATAAGATCTCTGATTACGATTTTATAATATTTGATGAGTGTCATATCATGGAAGCGCCAGCCATGGCGTTCTACTGTTTGTTGAAAGAATATGAATATAGAGGCAAAATCATTAAGGTGTCCGCAACACCACCAGGACGAGAGTGTGAGTTCTCTACTCAGCATCCAGTCGATATCCATGTGTGCGAAAATCTCACTCAGCAACAATTCGTTAGAGAGCTTGGTTCCGGATCAAACGTTGACGCAACAAAGTATGGAAACAACATACTTGTGTATGTTGCAAGCTACAACGACGTCGACTCATTAGCGCATGCTTTAACTGAATTACACTATTCAGTCATAAAGGTTGATGGGAGAACAATGAAACAGAACACTACAGGGATAGTGACAAATGGCACCTCTCAAAAGAAGTGTTTTGTCGTTGCTACGAATATCATTGAGAATGGTGTCACATTAGACGTTGACGTTGTGGTCGACTTCGGACTCAAAGTAACAGCTGAATTAGATGTTGACAACAGAGCTATTTTATACAAGCGTGTTAGCATTTCATATGGTGAGCGAATTCAAAGGCTAGGGCGTGTTGGAAGAAACAAACCCGGAACAGTCGTTCGAATTGGAAAAACCATGAAGGGTTTGCAAGAAATCCCAGCCATGATAGCGACAGAAGCAGCATTCATGTGCTTTGCATATGGACTAAAGGTCATAACACACAATGTTTCAACAACACACTTAGCAAAGTGCACAGTCAAGCAAGCGCGAACAATGATGCAATTTGAGCTTTCACCATTTGTAATGGCAGAACTAGTCAAATTTGATGGCTCAATGCACCCACAAATCCATGAAGCACTTGTGAAATATAAGCTTAGAGATTCTGTAATAATGCTCAGACCTAACGCAATTCCCAAAGTTAATTTTCACAACTGGCTGACAGCACGTGATTACAACAGGATAGGTTGTTCACTAGAGCTAGAGGATCATGTCAAAATTCCATATTACATACGAGGCGTCCCTGACAAGTTATACGGGAAATTGTATGATATCATTTTACAATATAGTCCAACCAGTTGTTACGGAAGACTATCAAGTGCTTGTGCTGGGAAGGTAGCATACACCTTGCGAACGGACCCTTGTTCGCTACCACGAACAATAGCAATAATCAATGCATTAATCACAGAGGAGTATGCTAAGAGAGATCATTACCGTAATATGATAGCAAATCCTTCGTCTTCGCATGCATTCTCACTAAATGGATTAGTCTCCATGATTGCTTCAAGATACATGAAAGACCACACAAAAGAGAATATAGACAAACTTATTAAGGTGCGGGATCAACTACTCGAATTTCAAGGCATGGGTATGCAATTTCAAGATCCATCAGAACTAATGGATATTGGCGCCTTGAACACAGTTATACATCAAGGAATGGATGCAACAGCTGCATGCATTGGTCTCCAAGGACGATGGAATGCATCACTCATACAACGTGATCTCATGATTGCAGGAGGAGTATTCATTGGAGGAATATTAATGATGTGGAGCTTGTTCACTAAGTGGGGAACGACCAATGTATCACACCAAGGAAAGAACAAGCGTAGCCGACAAAAATTGAAGTTCAAGCAAGCTAGAGACACTAAATACGCATATGATGTGACAGGATCGGAAGAAACTCTTGGTGAAAACTTTGGAACAGCTTATACAAAGAAGGGCAAAGGAAAAGGAACCAAAGTTGGTCTTGGGGTGAAGCAGCATAAATTTCACATGATGTATGGTTTTGATCCTCAAGAGTACAATCTAATTCGTTTTGTCGACCCTCTTACAGGTGCAACATTAGATGAACAAATCCATGCTGATATTCGCTTAGTGCAAGAACACTTTGACATAATCCGAGAAGAGGCAGTCGCAAATGACACAATTGAGCGACAACACATATACGGAAATCCTGGATTACAAGCATTTTTCATACAGAATGGGTCAGCAAATGCTTTAAGAGTTGATTTAACACCACATTCACCTACACGGGTTGTCACAGGCAATAACATAGCAGGATTCCCAGAATATGAAGGTACACTCCGTCAAACTGGAACAGCTATAACCATACCCATTGGTCAAGTCCCAATTGCAAATGAAGCAGGAGTCGCACACGAATCAAAATCCATGATGAATGGATTGGGTGATTACACGCCAATATCACAACAATTGTGTTTAGTGCAAAATGACTCAGATGGAGTAAAACGAAATGTGTTTTCAATCGGATATGGCTCATATCTCATTTCACCAGCGCACTTATTTAAATATAACAATGGCGAAATTACAATTAGATCATCAAGAGGATTGTATAAAATTCGCAACTCTGTGGATTTAAAATTACATCCGATTGCACACAGAGACATGGTCATAATCCAACTTCCAAAGGATTTCCCACCGTTCCCAATGCGTTTGAAATTTACACAACCATCACGAGAGATGCGAGTCTGCTTAGTAGGAGTCAATTTCCAACAGAACTATAGCACTTGCATTGTATCAGAAAGCAGCGTAACAGCACCAAAGGGGAATGGAGATTTTTGGAAGCATTGGATTTCAACAGTCGACGGTCAATGTGGACTACCATTGGTAGATACTAAGAACAAACACATTGTTGGAATCCATAGTCTTGCATCTACAAGTGGGAACACCAATTTCTTTGTTGCCATGCCTGAGAACTTTAATGAATACATTAATGGACTTGTGCAAACAAACAAATGGGAAAAGGGATGGCACTACAATCCGAATCTCATATCTTGGTGTGGATTAAACTTAGTCGATTCTGCTCCAAAGGGTTTGTTTAAAACGTCAAAATTAGTTGAAGATTTGGATGCTAGTGTTGAAGAGCAGTGCAAGGTTACTGAAACATGGCTCACAGAGAAATTACAAGATAATTTGCAAGTGGTCGCGAAATGCCCAGGCCAACTCGTCACTAAGCATGTTGTCAAAGGTCAATGCCCACACTTCCAGTTATATTTGTCAACACATAATGATGCCAAAGAATATTTCGCACCCCTGCTTGGAAAATATGATAAGAGCAGACTCAATAGAGCGGCATTTATTAAAGACATATCAAAGTATGCAAAACCAATTTATATTGGAGAAATCAATTATGATATCTTTGATAGAGCTGTACAACGAGTCATTAATATTCTTAGAAATGTTGGAATGCAACAATGCGTGTATGTTACAGACGAAGAAGAAATTTTTAAATCACTCAACCTAAACGCAGCTGTCGGAGCACTGTACACAGGAAAGAAGAAAGATTACTTTGAAAGTTTTTCAAATGAAGACAAAGAAGAAATCGTGATGAGATCATGTGAACGCATTTACAATGGACAACTTGGTGTGTGGAATGGGTCACTCAAAGCCGAAATCAGACCAATAGAGAAAACCATGTTAAATAAGACTCGAACCTTTACAGCAGCTCCATTGGAAACTTTGCTTGGAGGAAAAGTGTGTGTGGACGATTTTAATAATCAATTTTATTCGCACCATTTAGAAGGCCCATGGACTGTTGGAATAACAAAATTCTATGGAGGTTGGAATCGCTTACTTGAGAAGCTGCCGGAAGGATGGATTTACTGTGATGCTGACGGATCCCAATTCGATAGTTCGTTAACACCATATCTTATTAATGCAGTGTTGAATATTCGATTACAGTTCATGGAGGATTGGAACATAGGAGCGCAAATGCTTAAAAACCTTTATACTGAGATTGTTTATACACCAATTGCAACACCAGATGGATCTATCGTGAAGAAATTCAAAGGAAATAATAGTGGACAACCTTCTACAGTAGTTGATAATACATTGATGGTTATAATAGCTTTCAACTATGCTATGCTATCGAGCGGTATTAGAGAAGAAGAGATCGATAACTGCTGTAGGATGTTTGCAAATGGTGATGACCTGCTCCTAGCAGTACATCCTGATTTTGAATACATTCTAAACGGATTTCAAGATCACTTCGGAAATCTCGGATTGAATTTTGAGTTTACATCACGAACACGAGATAAATCCGAGCTATGGTTTATGTCTACAAGAGGAATCAAATGTGAAGGAATCTACATACCTAAACTCGAGAAAGAAAGAATAGTCGCAATACTCGAATGGGATCGATCAAATCTACCTGAGCATAGATTGGAAGCCATTTGTGCAGCTATGGTTGAAGCATGGGGCTACTCAGATCTTGTCCACGAAATTCGGAAATTTTATGCGTGGCTTCTAGAAATGCAACCTTTCGCAAATCTAGCAAAAGAAGGAATGGCGCCATACATAGCAGAAACAGCGCTCCGTAACCTTTATCTTGGAACGGGCATTAAAGAAGAAGAAATCGAGATATATTTCAAGCAATTTGTTAAGGATCTTCCCGGATACATAGAAGATTACAATGAAGAAGTCATTCACCAATCGGGAACAGTTGATGCAGGTGCACAAGGCGGCAGCGGAAGCCAAGGAACAACACCACCAGCAACAGGTGGCGGATCAAGACCAGCGGCTTCAGGAG------------CAGGATCTGGTAGCGGAAC----AGGGACTGGAACCGGTGCAACTGGGGGCCAGACAGGA---------ACT---G---GTAGTGGTGCTGGAACAGGATCTGGAGCGACCGGAGGCCAATCAGGATCTGGAAGTGGCACTGGACAGACTGGCACAGGCTC---AGCAGGAACTGGTGCA------------ACGGGAGGCCAGAGAGATAAGGATGTAGATGCAGGTACAACAGGAAAAATTTCTGTACCAAAGCTCAAGGCCATGTCAAAGAAAATGCGCTTACCGAAAGCAAAAGGAAAAGATGTCTTGCATCTGGACTTCTTGCTTACATACAAGCCACAGCAGCAAGACATATCAAACACAAGAGCAACTAAGGAAGAGTTCGATAGATGGTACGACGCCATAAAGAAAGAATACGAGATCGATGATACACAAATGACAGTCGTCATGAGTGGTCTGATGGTCTGGTGCATTGAAAATGGTTGCTCACCAAACATAAACGGAAATTGGACGATGATGGATGGGGATGAACAAAGAGTTTTCCCACTAAAACCAGTTATCGAAAACGCATCTCCAACTTTTCGACAAGTTATGCATCATTTCAGTGATGCAGCTGAAGCGTATATAGAATACAGAAATTCTACTGAGCGATACATGCCAAGATATGGACTTCAGCGAAATCTCACCGACTATAGCTTAGCGCGGTATGCTTTTGATTTCTACGAAATGACTTCACGCACACCAGCTAGAGCTAAGGAAGCCCACATGCAG---ATGAAAGCCGCAGCAGTTCGTGGTTCAAACACACGACTGTTCGGTTTGGACGGAAATGTCGGCGAGACTCAGGAGAATACAGAGAGACACACAGCTGGCGACGTTAGTCGCAATATGCACTCTCTGTTGGGAGTGCAGCAACACCATTAGTCTCCTGGAAACCCTGTTTGCAGTACCTATAATATATACTA------ATATATAGTACGTTGGTGAGGCTTTGCCTC-----------------------GGTTTTACTATCTTATTATGTATGTATTTACAGCGTGAACCAGTCTGCAGCATGCAGGGTTGGACCCAGCGTGTTCTGGTGTAGCGTGTACTAGCGTCGAGCCATGAGACGGACTGCACTGGGTGTGGCTATGCCACTTGTGTTGCGAGTTTCCTGGTAAGAGAC----------------

>MH093730

-----------------------------------CAACAACACACAACAAAACACAACCAAACAACACCAAGTTTTCTTTGCTCAGATTGTAGTGAACGGCTCGCAAGAAACGGTTCTTCGAGATCACTCTCTGATTTCTTTCTC----------TCTCACACTTGCATTCAAGCGGAATGGCGGGATCGTGGACTCACGTGTCTTACAAGTGGCAGCCAAATGTCAACAATGACCGTGATGTAAGAAAGGTAATGGAGATGTTTGCAGCAAAACATCAACATTATACAGAGGAGCAGCGACTTGCACATAACAGCAAGTTGCTAAGGAAGGCGTATGTTGTGGACGTTGAACCAGTGAAGCCAGCACCGGAGCCTATAAGGCGTAAAGTGTGGGTGGAAAAATTCGACCACAACCCAACCGAAGACCTGGTGTATCCGCGCCTT---GTCACGGTTAAAAAGGCAGCAGAAATGAAGCCCGTGAACACCTCTATAAATAAACTTATAAGGGATGTTCTGGACATATCAAAAGGGAGCAGCCTTAAACTTGAGCTAATTGGTAAACGCCAGAAGTGCAGAACACAACTAGCTATTAAGAAATACAACAATAAGGACTACCTCCATTGCAGGACACGTCATGAAGACAACATGTTCAAGAGGAGGGACGTGGCAATCGGCATTGAATGGATCCCAACCATTGAAGCGATTGCTCGATGCTACAGCACAGTAAACAAACAGGAGATGCAAAGTCTCTACAAAGGCAGTAGTGGCTTAACATTCATGCAAAATGATGAATTATTCATTGTTAGAGGCAGAATGAATGGCGAGTTAGTTAACAGCCTGGAAGAGAACAAGAACGTGCTCGACATCGATCACTACGCTGATCCACAAGCGAACGACTTTTGGAAAGGGTATACTGATGCGTACGTTGCGAATCGCAGTATCTCCACTACACATACCGTGCATACTCCAACAATCAACTTGGTAGAGTGTGGAAAGAGAATGGCATTACTTGAAATTTTATTCCACTCAACTTTTAAGATTACATGCAAGACATGCAATATCGATGACTTGGAGTTATCAGACGATGAATTTGGAGCAAAACTTTTCAAGAATCTACAACGTATCGAGGAACAGCAACGCGAATATCTCGCTAAAGATCAAAAGCTGCGTCGCATGATTCAATTCATCAAAGAAAGGTGTAATCCAAAGTTCTCTCATCTACCATTGCTTTGGCAAGTTGCAGAAACAATAGGACATTATACGGACAATCAGTCAAAGCAAATAATTGATATCAGTGAAGCACTCATCAAAGTGAACACATTAACACCTGATGATGCAGTCAAGGCGAGCGTAGCACTGCTTGAGGTGGCACGATGGTATAAAAATAGGAAAGAATCACTTAAAACAGACACATTAGATTCGTTCCGAAACAAGATATCCCCAAAGAGCACGATAAACACAGCATTGATGTGTGATAATCAACTAGACAAGAATGCAAATTTTGTCTGGGGAAACAGGGAGTATCATGCAAAGCGCTTCTTCTCAAATTACTTTGAAGCAGTAGATCCTACTGACGCATACGAGAAGCACGTAACTCGTTTCAATCCAAATGGTCAACGGAAATTATCAATTGGTAAACTAGTAATTCCGTTAGATTTCCAGAAAATAAGAGACTCATTCGTTGGTTTGGCAATAAACAAACAGCCACTTACAAAGGCTTGTGTGAGCAAAATCGATGGTGGTTATGTATACCCATGCTGTTGTGTCACAACAGAATTTGGAAAACCAGCATACTCTGAGATAATACCACCAACAAAGGGCCATATAACAATTGGAAATTCTGTAGACCCAAAGATCGTAGACCTGCCAAATACAACACCACCAAGTATGTACATTGCCAAAGACGGATATTGTTACATCAACATTTTCTTAGCAGCAATGATAAATGTCAATGAAGACTCAGCAAAGGACTACACAAAATTCCTCAGGGATGAATTAGTCGAGCGACTTGGAAAGTGGCCGAAACTTAAAGATGTTGCAACAGCGTGCTATGCGTTATCAGTTATGTTTCCAGAGATTAAGAACGCTGAATTGCCTCCAATACTCGTTGACCACGAAAGCAAGTCAATGCACGTGATTGATTCATACGGCTCATTGAGCGTTGGATTCCACATCCTTAAAGCAAGCACTGTTGGCCAGCTCATAAAATTTCAATACGAGTCAATGGATAGTGAGATGCGCGAGTATATAGTGGGAGGTACTTTAACGACCCAAACATTTAACAAACTCCTTACATCTTTAGCTAAAAATATGTTCAAACCAGATCAAATTAAGCAGATGATTGAGGAGGAACCCTTCCTACTAATGATGGCAATCGCGTCACCAACTATGCTTATAGCGCTATACAATAATTGCTATATAGAGCAGGCTATGACGTATTGGATCGTTAAAAACCAAGGAGTGGCAGCGATATTCGCACAACTGGAAGCACTAGCAAAGAAGACCTCACAAGCTGAATTGCTAGTCCAACAAATGCAAATACTTGAAAAGGCGTCTAATCAATTGAGGCTTGCAGTCACGGGTTTAAACCATGTCGATCCAGCCAAGCGTCTGCTATGGTCGCATCTAGAAGCAATGACAACACGATCAGAGATGAACAAGGAATTAATTGCTGAAGGCTATGCCCTGTATGATGAGCGCCTGTATGCACTTATGGAAAAAAGTTACGTAGATCAATTAAACCAATCATGGGCAGAGTTATCATTCTGTGGAAAATTTTCAGCAATATGGCGTGTGTTCAAAGTCAAGAAATATTACAAGCCGTCTTTAACCGTGAGAAAAAGCGTAGATTTAGGCGCTGTGTACAATATATCAGCTACGCATCTAATATCAGATTTAGCGCAGAGAAGTCGCGATCGAGCCAGCTCTATTTTAACCAAACTCCGCAACGGTTTTTATGATAAGTTAGAGAAGGCTAGAACTCGAGCAATTAAAACTGTTTATTGGTTCATACCTGACATATTTAGACTTATGCATATTTTCATAGTTCTTAGTTTATTAACAACTGTGGCAAACACTATTATTGTGACTATGAATGATTACAAAAAGCTGAAAAAGCAACAAAGAGAGGATGAGTATGAAGCTGAAATTAACGAGGTTCGAAAAATTCACGCTATTCTGATGAAAGAGCATAATGACAATCTAACATGTGAACAGTTTATCGAACATATGCGCCAGACGCATCCACGATTAATTGAAGCCACATTGGAGTTGACACACACAGGCGTCATTCATGAAGGAAAGAGCAACCTAGAAACCAATCTCGAGCAAGCTATGGCAGTGGGCACTTTACTCACAATGATGTTAGACCCACAGAAGAGTGATGCAGTATACAAAGTGTTGAATAAGATGAGAACTGTAATTAGCACATTTGAGCAGAATGTCCCATTTCCTTCAATCAACTTTACAAATATTTTAACACCATCAGTGGCGCAACAAAGTGTAGATGTTGATGAACCTCTAACTTTAAGTACTGATAAGAATCTAACAATAGATTTTGATACTAATCAAGATTTACCAGCGGACACGTTTAGCAATGATGTTACTTTCGAAGATTGGTGGGCAAATCAAATAAGCAACAATAGAACAGTTCCACATTATCGGCTTGGTGGCAAATTTGTAGAATTCACAAGAGAAAATGCAGCACATGTTAGCATTGAGCTCGCACACTCAAATCTTGAGAAGGAGTTTCTACTCAGAGGAGCTGTTGGCTCAGGAAAATCCACTGGTCTTCCATATCATCTTAGTATGCGTGGTAAGGTGCTTTTAATAGAACCAACAAGACCACTAGCCGAGAATGTCTGCAGACAGCTTCAAGGACCACCATTCAATGTCAGCCCGACACTACAAATGCGTGGTTTAAGCTCTTTTGGTTGCACGCCAATTACGATAATGACATCAGGTTTTGCACTGCACATGTATGCAAATAATCCGGATAAGATCTCTGATTACGATTTTATAATATTTGATGAGTGTCATATCATGGAAGCGCCAGCCATGGCGTTCTACTGTTTGTTAAAAGAATATGAATATAGAGGCAAAATCATTAAGGTGTCCGCAACACCACCAGGACGAGAGTGTGAGTTCTCTACTCAGCATCCAGTCGATATCCATGTGTGCGAAAATCTCACTCAGCAACAATTCGTTAGAGAGCTTGGTTCCGGATCAAACGTTGACGCAACAAAGTATGGAAACAACATACTTGTGTATGTTGCAAGCTACAACGACGTCGACTCATTAGCGCATGCTTTAACTGAATTACACTATTCAGTCATAAAGGTTGATGGGAGAACAATGAAACAGAACACTACAGGGATAGTGACAAATGGCACCTCTCAAAAGAAGTGTTTTGTCGTTGCTACGAATATCATTGAAAATGGTGTCACATTAGACGTTGATGTTGTGGTCGACTTCGGACTCAAAGTAACAGCTGAATTAGATGTTGACAACAGAGCCATTTTATACAAACGTGTTAGCATTTCATATGGTGAGCGAATTCAAAGGCTAGGGCGTGTTGGAAGAAACAAACCCGGAACAGTCGTTCGAATTGGAAAAACCATGAAGGGTTTGCAAGAAATCCCAGCCATGATAGCGACAGAAGCAGCATTCATGTGCTTTGCATATGGACTAAAGGTCATAACACACAATGTTTCAACAACACACTTAGCAAAGTGCACAGTCAAGCAAGCGCGAACAATGATGCAATTTGAGCTTTCACCATTTGTAATGGCAGAACTAGTCAAATTTGATGGCTCAATGCACCCACAAATCCATGAAGCACTAGTGAAATATAAGCTTAGAGATTCTGTAATAATGCTCAGACCTAACGCAATTCCCAAAGTTAATTTTCACAACTGGCTGACAGCACGTGATTACAACAGGATAGGTTGTTCACTAGAGCTAGAGGATCATGTCAAAATTCCATATTACATACGAGGCGTTCCTGACAAGTTATACGGGAAATTGTATGATATCATTTTACAATATAGTCCAACCAGTTGTTACGGAAGACTATCAAGTGCTTGTGCTGGGAAGGTAGCATACACCTTGCGAACGGACCCTTGTTCGCTACCACGAACAATAGCAATAATCAATGCATTAATCACAGAGGAGTATGCTAAGAGAGATCATTACCGTAATATGATAGCAAATCCTTCGTCTTCGCATGCATTCTCACTAAATGGATTAGTCTCCATGATTGCTTCAAGATATATGAAAGACCACACAAAAGAGAATATAGACAAACTTATTAAGGTGCGGGACCAACTACTCGAATTTCAAGGCATGGGTATGCAATTTCAAGATCCATCAGAACTAATGGATATTGGCGCCTTGAACACAGTTATACATCAAGGAATGAATGCAACAGCTGCATGCATTGGTCTCCAAGGACGATGGAATGCATCACTCATACAACGTGATCTCATGATTGCAGGAGGAGTATTCATTGGAGGAATATTAATGATGTGGAGCTTGTTCACTAAGTGGGGAACGACCAATGTATCACACCAAGGAAAGAACAAGCGTAGCCGACAAAAATTGAAGTTCAAGCAAGCTAGAGACACTAAATACGCATATGATGTGACAGGATCGGAAGAAACTCTTGGTGAAAACTTTGGAACAGCTTATACAAAGAAGGGCAAAGGAAAAGGAACCAAAGTTGGTCTTGGGGTGAAGCAGCATAAATTTCACATGATGTATGGTTTTGATCCTCAAGAGTACAACCTAATTCGTTTTGTCGACCCTCTTACAGGTGCAACATTAGATGAACAAATCCATGCTGATATTCGCTTAGTGCAAGAACACTTTGACATAATCCGAGAAGAGGCAGTCGCAAATGACACAATTGAGCGACAACACATATACGGAAATCCTGGTCTCCAAGCATTCTTCATACAGAATGGATCAGCTAATGCATTAAGAGTTGATTTAACACCACATTCACCTTTGCGTGTTGTGACGAACAACAACATAGCAGGATTTCCAGAATATGAGGGCACATTACGACAAACTGGCACAGCTCTCACTGTACCTGTGAATCAAGTACCAGCAGCAAATGAGACAGGAGTTGCCCACGAATCCAAATCTATGATGGCTGGATTGGGCGATTACACACCTATTTCTCAGCAGCTCTGTCTCATACAAAACGATTCTGAAGGAGTCAAACGTAATGTGTATGCAATTGGATATGGATCATATTTAATATCACCGGCGCATCTTTTCAAGTATAACAATGGTGAAATCACAATTAAATCCTCAAGGGGGCTGTATAAAATTAGAAATTCAGTCGATGTCAAATTGCACCCGATTGCACAGAGGGACATGGTCATAATTCAACTTCCAAAAGACTTCCCACCGTTCCCAATGCGACTTAAGTTCTCAACTCCGTCAAGAGATGTGCGTGTGTGCTTAGTTGGAATCAACTTTCAACAGAATCATACCACGTGCATAATATCCGAAAGCAGTGTGACAGCACCCAAAGGAAATGGTGATTTCTGGAAACATTGGATTTCAACTGTTGATGGGCAATGTGGGCTACCGTTAGTCGACACTAAGAATAAACACATTGTCGGAATTCACAGCCTGGCCTCAACAAGTGGAAATACGAACTTTTTCGTTGCAATGCCTGAGAACTTCAATGAATATATATCTAATCTCGTGCAAACGAATAAGTGGGAAAAGGGATGGCATTACAACCCAAATCTTATTTCATGGTGTGGTCTAAACCTAGTTGATTCAGCACCTAAAGGATTATTTAAAACATCAAAACTTGTTGAAGATTTGGATATGAGCGTTGAAGAACAATGCAAGGTGACAGAGACATGGTTGACGGAACACATCCAGGATAATCTACAGGTCGTTGCAAAATGTCCAGGCCAACTTGTAACAAAGCATGTCGTTAAAGGCCCATGTCCGCATTTTCAACTGTATTTATCCACACATGATGAAGCAAAGTTGTACTTTTCACCTTTGCTTGGAAAGTATGACAAGAGTAGGTTGAACAGGGCAGCATTTATCAAAGATCTTTCAAAGTACGCAAAGCCAATTTATATTGGAGAGATCAATTATGAAATCTTTGATAAGGCAGTTGATCGAGTCATAAGCATCCTCAGAAGTGTAGGAATGCTACAGTGTACATACGTGACGGACGAAGAAGAAATTTTCAATTCGTTAAATATGAACGCAGCCGTAGGTGCACTCTACACAGGAAAGAAGAAAGACTATTTCAAAGATTTCTCGAACGATGACAAAGTCGAAATCATCATGCGTTCATGTGAGCGCATCTACAATGGACAATTGGGTGTATGGAACGGTTCACTCAAAGCTGAAATACGACCAATAGAGAAAACCATACTAAACAAGACACGCACTTTCACAGCAGCGCCATTAGAAACTCTACTTGGTGGGAAAGTATGTGTGGACGATTTCAACAATCAATTTTATTCACATCATCTTGAAGGCCCATGGACCGTAGGAATCACAAAGTTTTATGGAGGATGGAACCGACTTTTGGAGAAATTGCCAGAAGGATGGATTTATTGCGATGCAGATGGATCCCAGTTTGACAGCTCGTTAACTCCATATCTTATTAATGCTGTATTGCACATTCGCTTACATTTCATGGAAGAATGGGAGTTGGGAGCTCAGATGTTGCGAAATTTATACACAGAGATTGTTTATACGCCAATCGCAACGCCTGATGGGTCTGTCATCAAGAAATTCAAAGGAAATAATAGTGGGCAACCATCTACAGTCGTTGACAACACGCTTATGGTTATTATAGCATTTAATTACGCAATGTTATCAAGTGGCATTCCTGAAGACAAAATTGACGACTGCTGTAGAATGTTTGCAAACGGTGACGACTTACTCTTGGCAGTGCATCCGGATTACGAATATATATTGGACGGATTTCAAAATCATTTTGGAAACCTTGGCCTTAACTTTGAGTTCACATCGAGGACAAAGGACAAATCAGAGTTATGGTTCATGTCAACACAAGGAGTCAAGTGTGAAGGTATCTACATACCAAAACTCGAAAGGGAAAGAATAGTCGCAATCCTTGAATGGGACCGATCGAACTTGCCTGAGCATCGTCTTGAAGCTATCTGTGCAGCCATGGTTGAAGCATGGGGTTACCCAGATTTAGTTCATGAAATTCGAAAGTTTTACGCGTGGCTTCTTGAAATGCAACCCTTCGCGAACCTGGCAAAGGAAGGCATGGCGCCATACATAGCAGAAACAGCACTCCGCAACCTCTACCTTGGAACAGGCATCAAAGAAGAAGAAATTGAAAAATATTTTAGACAGTTTGCCAAGGATCTTCCTGGATACGTAGAAGATTACAATGAAGAAGTTATTCATCAATCTGGTCAAGTTGACGCAGGGAGACAGGGCGGTAGCGGCACTCAAGGAGGCACGCCACCAACAGGA---------------------------------------------------AGTGGAGGCAC----TGGATCTGGCACTCAAGGCAATGGGGGTCAGACGGGA---------TCCCAAG---GAAGTAGTGGTCAACAAGGGTCCAGTGGGGGCACTGGTCAAGGAGCAGCTGGAAACAACGGCGG------------AGGTCAGACAGGAGGCTCTAGTGGG------------ACAGCTGGTCAGAGAGATAAGGACGTTGACGCAGGCTCGGCTGGAAAGGTATCCGTACCAAAGCTTAAAGCCATGTCAAAGAAAATGCGCTTGCCAAAGGCAAAAGGAAAAGATGTCTTGCATTTGGACTTTTTGTTGACATACAAGCCACAACAGCAGGACATATCGAACACAAGAGCAACTAAGGAAGAGTTCGATAGATGGTACGACGCCATAAAGAAGGAGTACGAGATCGATGATACACAAATGACAGTCGTCATGAGTGGTCTGATGGTCTGGTGCATCGAAAATGGTTGCTCACCAAACATAAACGGAAATTGGACGATGATGGATGGGGATGAACAAAGAGTTTTTCCACTAAAACCAGTTATTGAAAACGCATCTCCAACTTTTCGACAAGTTATGCATCATTTCAGTGATGCAGCTGAAGCGTATATAGAATACAGAAATTCTACTGAGCGATACATGCCAAGATATGGACTTCAGCGAAATCTCACCGACTATAGCTTAGCGCGGTATGCTTTTGATTTCTACGAAATGACTTCACGCACACCAGCTAGAGCTAAGGAAGCCCACATGCAG---ATGAAAGCCGCAGCAGTTCGTGGTTCAAACACACGACTGTTCGGTCTGGACGGAAATGTCGGCGAGACCCAGGAGAATACAGAGAGACACACAGCTGGCGATGTTAGTCGCAATATGCACTCTCTGTTGGGAGTGCAGCAGCACCACTAGTCTCCTGGAAACCCTGTTTGCAGTACCTATAATATATACTA------ATATATAGTATTTCAGTGAGGTTTTACCTC-----------------------GACTTTACTATTTTATTACGTATGTATTTAAAGCGTGAACCAGTCTGCAGCATACAGGGTTGGACCCAGTGTGTTCTGGTGTAGCGTGTACTAGCGTCGAGCCATGAGATGGACTGCACTGGGTGTGGCTTTGCCACTTGTGTTGCGAGTCTCCTGGTAAGAGACAAAAAAAA--------

>MH093731

--------------------AAAAACAACAAAACTCAACACAACACAACAAAACACAACCAAGCAAATCCAATTTACTTGCGCTCAGATTGTAGTGAACGGCTCGAACGAAACGGTTCTTTGAGATCACTCTCTGATTCCTCCTCA----------TCTCTCAACTTCTCTCGAAAGAAATGGCAGGAACGTGGACCCATGTGACGTATAAGTGGCAGCCAGATGTCAACAACGATCGTCACATTAAGAGAGTAATGGAAATGTTTGCAGCAAAACATCAACATTACTCGGAAGAACAGCGACTTGCTCACAATATGAAATTATTGAGGAAGACAAGTGTTGTGAGCGTTGAGCCTGCAAAACCAAAACAGAAGCAGGCAACTCAACAGATGTGGGTTGAGAAATGTGATCACAATCCTGTTGATCACTTAGTATATCCACGATTTGAAAGATCTATCAACAAAGTG---GATACGAATATCAAAAGTGCATCTGTAAGCAAGCTAACCAGAGAGATTTTAGAAATCTCAAAGGCAAGCGGCCTTAAAGTTGAACTAATTGATAAGCGTAAAAGATCTAAAACACAGTTATCAATCAAAAGGTTCAATGGCAAGGACTTTCTCCACTGCAAAACAAACCACGAAAACAATTTGTTTAAGAGGAAGGACATAGCTATTGGACACAAATGGCTTCCAACAATCGAAGCTATTGCTCGCTGTTATAGTACAATGAACCAAAAGGAATTGCAAAGCCTTTATAAAGGCAGCAGTGGTCTCACATTCATTCAAAATGATGAACTGTTCGTTGTTAGAGGGAGAATGAATGGTGAACTAGTCAATAGCCTATGTGAAACAGACCGGGTTACGGATATTGAACACTATGCAGATCCCCAAGCTAATGATTTTTGGAGAGGATACACAAATGCTTATGTGGATAATCGTAGTATTTCTACCACCCACACAGAGCACACCCCGACAATCAATCTAGAAGAGTGTGGAAAACGAATGGCTCTACTTGAGATATTATTCCACTCCACATTCAAGATTACATGTAAAACATGCAACATGGATGATCTTGAATTATCAGATGATGAGTTCGGAGCTAAACTCTACAAAAATCTGCAACGCATCGAAGAGAAGCAACGAGAGTACCTTGCCAAGGATCAAAAGTTATCCAGAATGATACAATTTATCAAAGAAAGATGCAACCCAAAATTCTCACATTTACCAATGTTGTGGCAAGTTGCAGAAACAATAGGACACTATACTGATAATCAGTCAAAGCAAATAATGGATATCAGCGAAGCGCTCATCAAAGTTAATACTTTAACTCCTGATGACGCTATGAAAGCAAGCGCAGCATTACTTGAAGTGTCACGATGGTATAAGAATCGTAAGGAGTCGCTCAAAACCGACTCATTGGAATCTTTTAGAAATAAAATATCACCAAAGAGTACAATAAATGCAGCTCTAATGTGTGACAATCAATTGGATAAAAATGCAAATTTTGTATGGGGTAATAGGGAATATCACGCTAAACGATTTTTCGCAAACTACTTTGAAGCAGTGGACCCCACAGATGCATATGAAAAGCACGTCACACGGTTTAACCCTAATGGTCAACGGAAGCTATCAATAGGAAAGTTAGTTATCCCACTAGACTTTCAAAAGATTAGAGAATCATTCGTTGGACTTCCGATCAATAGACAACCGCTAGACAAATGTTGCGTTAGTAAGATCGAAGGGGGATATATATACCCATGTTGCTGCGTCACAACAGAATTTGGTAAACCAGCATACTCTGAGATAATACCTCCGACAAAAGGCCACATAACAATAGGCAATTCGATTGATCCAAAGATTGTGGACTTGCCAAACACAACACCACCCAGCATGTACATTGCTAAGGATGGGTACTGTTATATCAACATCTTTCTAGCAGCCATGATCAACGTTAATGAAGAATCTGCCAAGGATTACACGAAATTCTTGAGAGATGAACTAGTCGAGCGTCTTGGAAAGTGGCCAAGGCTTAAAGACGTAGCAACAGCGTGTTATGCATTATCTGTAATGTTTCCAGAAATTAAGAATGCTGAGCTACCTCCAATTTTAGTTGATCATGAAAATAAATCAATGCACGTCATCGACTCATATGGTTCACTAAGCGTTGGATTTCACATATTGAAAGCAAGCACGATTGGTCAATTAATTAAATTTCAATATGAATCTATGGATAGTGAAATGCGCGAATACATAGTAGGAGGAACTCTTACGCAACAGACATTCAACACACTTCTTAAGATGCTCACGAAAAACATGTTCAAACCAGAGCGCATTAAGCAGATAATTGAGGAGGAACCCTTCTTGCTTATGATGGCAATCGCATCTCCAACGGTATTAATAGCACTATATAATAATTGTTACATTGAGCAAGCTATGACATACTGGATCGTGAAGAATCAAGGAGTTGCAGCCATATTCGCACAACTCGAAGCATTAGCCAAGAAAACATCCCAGGCTGAATTGCTAGTTCTACAAATGCAGATACTTGAAAAAGCATCTAGTCAACTAAGATTAGCAGTTTCAGGACTTAGCCATGTCGACCCAGCAAAGCGACTTTTGTGGTCACACCTCGAAGCAATGACAACACGGTCAGAAATGAACAAGGAGTTGATAGCTGAGGGATATGCACTATACGACGAGCGTCTATATACCCTGATGGAAAAAAGTTACGTAGATCAATTAAACCAGTCATGGGCAGAATTATCATACTGTGGAAAATTTTCAGCAATATGGCGTGTGTTCAAAGTCAGGAAGTATTACAAACCGTCTTTAACCGTGAGAAAAAGCGTAGATTTAGGCGCTGTATACAATATATCAGCTACGCATCTAATATCAGATTTAGTGCGGAAAAGTCAAGATCAAGTCAGCTCTATTTTAACCAAACTCCGCAACGGTTTCTATGATAAATTAGAGAAAGCTAGAATACGTACTATTAAAACGGTTTATTGGTTTATACCCGATATATTTAGACTCATGCATATATTCATAGTTTTGAGTTTATTAACCACCATAGCTAACACTATCATAGTAACTATGAATGACTACAAGAAATTGAAGAAACAACAAAGAGAAGACGAATATGAAGCAGAAATTAACGAAGTTCGCAAAATCCATTCTACCTTGATGGAAGAGCGGAAGGACAATTTGACATGTGAACAATTTGTTGAATATATGCGCCAAAACCATCCGCGGTTAGTTGAAGCAACACTGGACTTAACCCACACAGGTGTCATACATGAAGGAAAATCCAATCTCGAAACCAATTTGGAACAGGCAATGGCAGTTGGGACCTTGATAACCATGATACTTGATCCACAGAAAAGCGATGCTGTCTACAAGGTGTTGAATAAAATGCGGACAGTAATTAGTACAATCGAACAAAATGTCCCATTCCCTTCAGTGAACTTCTCTAACATCTTAACACCTCCAGTGACACAACAGAGTGTGGATGTTGATGAGCCATTAACACTTAGCACTGACAAAAATTTGACAATAGATTTCGACACGAATCAAGATTTACCTGCCGATACATTCAGCAATGATGTGACATTTGAAGATTGGTGGTCAAATCAGCTAAGCAACAACAGAACAGTGCCACACTACCGACTTGGGGGAAAGTTCATCGAATTCACACGGGAAAACGCAGCCCACACGAGTATTGAACTTGCGCACTCAAACATTGAGAGGGAATTCTTGCTTAGAGGAGCAGTCGGCTCGGGAAAATCTACTGGGTTACCATACCATCTTAGCATGCGCGGAAAAGTACTTCTGCTAGAACCTACAAGGCCGCTAGCCGAGAACGTGTGTAGGCAATTACAAGGACCACCATTTAATGTAAGTCCAACTCTCCAAATGCGTGGACTGAGTTCTTTCGGGTGTACTCCGATCACGATCATGACATCAGGTTTTGCATTACACATGTACGCAAACAATCCAGATAAAATATCTGAATACGATTTCATAATCTTCGATGAATGTCATATAATGGAAGCACCAGCAATGGCCTTTTATTGCTTACTCAAAGAATATGAATATCGAGGGAAAATCATTAAGGTATCAGCCACGCCTCCAGGAAGGGAATGCGAATTCACAACACAACATCCAGTAGACATCCATGTTTGTGAAAATCTAACTCAGCAACAGTTCGTTATGGAACTCGGGACCGGTTCAACCGCAGATGCTACGAAGTACGGAAATAACATCCTAGTTTATGTAGCAAGTTATAATGACGTCGATTCACTGTCGCATGCATTAGTTGAACTCAAATTTTCCGTTATTAAAGTGGATGGCCGAACAATGAAGCAAAACACAACAGGAATCATCACAAACGGTACCTCACAAAAGAAGTGTTTTGTTGTTGCAACAAATATAATTGAAAATGGCGTCACACTAGATATCGATGTTGTTGTTGACTTTGGACTGAAAGTCTCAGCTGATTTGGATGTTGACAACAGGGCAATATTGTATAAACGCGTGAGTATATCATATGGTGAACGCATACAACGACTGGGTCGTGTTGGAAGAAATAAACCTGGCACAGTTATTCGTATTGGAAAAACCATGAAAGGTTTGCAGGAAATTCCAGCAATGATCGCAACAGAAGCAGCCTTCATGTGTTTCGCATATGGTCTTAAAGTCATCACCCATAATGTTTCAACGACCCATCTTGCAAAGTGCACAGTTAAACAAGCAAGAACCATGATGCAATTTGAATTGTCACCATTTGTCATGGCTGAGCTCGTTAAGTTTGATGGTTCAATGCATCCACAAATTCATGAGGCTTTAGTAAAATACAAACTTAGAGATTCTGTCATAATGCTCAGACCGAATGCACTTCCAAAAGTTAATTTACATAATTGGCTTACAGCCCGGGATTATAATAGAATAGGTTGTTCATTGGAACTTGAAGACTACGTTAAAATTCCGTACTATATTAGGGGAGTTCCTGATAAGCTGTATGGAAAGCTATATGATATTATTTTACAGTATGGTCCAACTAGTTGTTACGGTAGACTATCAAGTGCGTGTGCAGGTAAAGTAGCATACACTTTGCGAACTGATCCGTGTTCACTTCCAAGAACAATAGCAATAATTAATGCTTTAATCACGGAGGAATATGCGAAGAGAGATCACTACCGAAACATGATTTCAAACCCCTCCTCATCACATGCATTCTCACTCAATGGGTTGGTATCCATGATTGCAACCAGATACATGAAGGATCATACAAAAGAGAACATTGACAAACTCATCAGAGTACGTGATCAATTGCTCGAGTTTCAGGGCACCGGAATGCAATTTCAAGATCCGTCAGAACTCATGGAGATTGGGGCTCTTAATACAGTCATTCACCAAGGAATGGATGCAACTGCAGCTTGCATCGGATTACAAGGACGATGGAACGCTTCGCTCATACAACGCGATCTCCTAATTGCAGGAGGAGTTTTTATCGGAGGCATTTTGATGATGTGGAGCTTATTCACTAAATGGAGCAACACAAATGTCTCACATCAAGGAAAGAACAAACGCAGTAGGCAAAAACTCCGATTTAAAGAAGCAAGAGACAACAAATATGCATATGATGTTACAGGATCGGAAGAGTGCCTTGGTGAGAATTTCGGAACAGCCTACACAAAGAAAGGTAAAGGAAAAGGAACTAAAGTTGGACTCGGTGTGAAACAACACAAATTTCATATGATGTATGGTTTTGATCCTCAAGAGTACAACCTAATTCGATTTGTTGATCCACTCACAGGAGCAACTCTTGATGAGCAAATCCATGCCGACATACGCTTAATTCAAGAGCATTTCGCTGAGATTCGTGAGGAGGCAGTAGTTAACGACACAATTGAAAGGCAGCAAATTTACGGCAATCCTGGATTACAAGCATTTTTCATACAGAATGGGTCAGCAAATGCTTTAAGAGTTGATTTAACACCACATTCACCTACACGGGTTGTCACAGGCAATAACATAGCAGGATTCCCAGAATATGAAGGTACACTCCGTCAAACTGGAACAGCTATAACCATACCCATTGGTCAAGTCCCAATTGCAAATGAAGCAGGAGTCGCACACGAATCAAAATCCATGATGAATGGATTGGGTGATTACACGCCAATATCACAACAATTGTGTTTAGTGCAAAATGACTCAGATGGAGTAAAACGAAATGTGTTTTCAATCGGATATGGCTCATATCTCATTTCACCAGCGCACTTATTTAAATATAACAATGGCGAAATTACAATTAGATCATCAAGAGGATTGTATAAAATTCGCAACTCTGTGGATTTAAAATTACATCCGATTGCACACAGAGACATGGTCATAATCCAACTTCCAAAGGATTTCCCACCGTTCCCAATGCGTTTGAAATTTACACAACCATCACGAGAGATGCGAGTCTGCTTAGTAGGAGTCAATTTCCAACAGAACTATAGCACTTGCATTGTATCAGAAAGCAGCGTAACAGCACCAAAGGGGAATGGAGATTTTTGGAAGCATTGGATTTCAACAGTCGACGGTCAATGTGGACTACCATTGGTAGATACTAAGAACAAACACATTGTTGGAATCCATAGTCTTGCATCTACAAGTGGGAACACCAATTTCTTTGTTGCCATGCCTGAGAACTTTAATGAATACATTAATGGACTTGTGCAAACAAACAAATGGGAAAAGGGATGGCACTACAATCCGAATCTCATATCTTGGTGTGGATTAAACTTAGTCGATTCTGCTCCAAAGGGTTTGTTTAAAACGTCAAAATTAGTTGAAGATTTGGATGCTAGTGTTGAAGAGCAGTGCAAGGTTACTGAAACATGGCTCACAGAGAAATTACAAGATAATTTGCAAGTGGTCGCGAAATGCCCAGGCCAACTCGTCACTAAGCATGTTGTCAAAGGTCAATGCCCACACTTCCAGTTATATTTGTCAACACATAATGATGCCAAAGAATATTTCGCACCCCTGCTTGGAAAATATGATAAGAGCAGACTCAATAGAGCGGCATTTATTAAAGACATATCAAAGTATGCAAAACCAATTTATATTGGAGAAATCAATTATGATATCTTTGATAGAGCTGTACAACGAGTCATTAATATTCTTAGAAATGTTGGAATGCAACAATGCGTGTATGTTACAGACGAAGAAGAAATTTTTAAATCACTCAACCTAAACGCAGCTGTCGGAGCACTGTACACAGGAAAGAAGAAAGATTACTTTGAAAGTTTTTCAAATGAAGACAAAGAAGAAATCGTGATGAGATCATGTGAACGCATTTACAATGGACAACTTGGTGTGTGGAATGGGTCACTCAAAGCCGAAATCAGACCAATAGAGAAAACCATGTTAAATAAGACTCGAACCTTTACAGCAGCTCCATTGGAAACTTTGCTTGGAGGAAAAGTGTGTGTGGACGATTTTAATAATCAATTTTATTCGCACCATTTAGAAGGCCCATGGACTGTTGGAATAACAAAATTCTATGGAGGTTGGAATCGCTTACTTGAGAAGCTGCCGGAAGGATGGATTTACTGTGATGCTGACGGATCCCAATTCGATAGTTCGTTAACACCATATCTTATTAATGCAGTGTTGAATATTCGATTACAGTTCATGGAGGATTGGAACATAGGAGCGCAAATGCTTAAAAACCTTTATACTGAGATTGTTTATACACCAATTGCAACACCAGATGGATCTATCGTGAAGAAATTCAAAGGAAATAATAGTGGACAACCTTCTACAGTAGTTGATAATACATTGATGGTTATAATAGCTTTCAACTATGCTATGCTATCGAGCGGTATTAGAGAAGAAGAGATCGATAACTGCTGTAGGATGTTTGCAAATGGTGATGACCTGCTCCTAGCAGTACATCCTGATTTTGAATACATTCTAAACGGATTTCAAGATCACTTCGGAAATCTCGGATTGAATTTTGAGTTTACATCACGAACACGAGATAAATCCGAGCTATGGTTTATGTCTACAAGAGGAATCAAATGTGAAGGAATCTACATACCTAAACTCGAGAAAGAAAGAATAGTCGCAATACTCGAATGGGATCGATCAAATCTACCTGAGCATAGATTGGAAGCCATTTGTGCAGCTATGGTTGAAGCATGGGGCTACTCAGATCTTGTCCACGAAATTCGGAAATTTTATGCGTGGCTTCTAGAAATGCAACCTTTCGCAAATCTAGCAAAAGAAGGAATGGCGCCATACATAGCAGAAACAGCGCTCCGTAACCTTTATCTTGGAACGGGCATTAAAGAAGAAGAAATCGAGATATATTTCAAGCAATTTGTTAAGGATCTTCCCGGATACATAGAAGATTACAATGAAGAAGTCATTCACCAATCGGGAACAGTTGATGCAGGTGCACAAGGCGGCAGCGGAAGCCAAGGAACAACACCACCAGCAACAGGTGGCGGATCAAGACCAGCGGCTTCAGGAG------------CAGGATCTGGTAGCGGAAC----AGGGACTGGAACCGGTGCAACTGGGGGCCAGACAGGA---------ACT---G---GTAGTGGTGCTGGAACAGGATCTGGAGCGACCGGAGGCCAATCAGGATCTGGAAGTGGCACTGGACAGACTGGCACAGGCTC---AGCAGGAACTGGTGCA------------ACGGGAGGCCAGAGAGATAAGGATGTAGATGCAGGTACAACAGGAAAAATTTCTGTACCAAAGCTCAAGGCCATGTCAAAGAAAATGCGCTTACCGAAAGCAAAAGGAAAAGATGTCTTGCATCTGGACTTCTTGCTTACATACAAGCCACAGCAGCAAGACATATCAAACACAAGAGCAACTAAGGAAGAGTTTGATAGATGGTACGATGCCATAAAGAAAGAATACGAGATTGATGACACACAAATGACAGTTGTCATGAGCGGTCTTATGGTGTGGTGCATTGAGAACGGTTGCTCACCAAACATAAACGGAAATTGGACAATGATGGATGGAGATGAACAAAGAGTTTTTCCACTCAAACCAGTCATTGAAAACGCATCCCCAACTTTCCGACAAATTATGCATCATTTTAGTGATGCAGCTGAAGCGTACATAGAGTACAGAAACTCTACTGAGCGATACATGCCAAGATACGGACTTCAGCGCAATCTCACCGACTATAGCTTAGCACGGTATGCATTTGATTTCTATGAAATGACTTCACGCACACCTGCTAGAGCTAAAGAAGCCCACATGCAG---ATGAAAGCCGCAGCAGTTCGTGGTTCAAACACACGACTGTTCGGTCTGGACGGAAATGTCGGCGAGACCCAGGAGAATACAGAGAGACACACAGCTGGCGACGTTAGTCGCAATATGCACTCTCTGTTGGGAGTGCAGCAGCACCACTAGTCTCCTGGAAACCCTGTTTGCAGTACCTATAATATGTACTA------ATATATAGTATGTCAGTGAGGTTTTACCTC-----------------------GTTTTTACTA-TTTGTTATGTATGTATTTAAAGCGTGAACCAGTCTGCAGCATACAGGGTTGGACCCAGTGTGTTCTGGTGTAGCGTGTACTAGCGTCGAGCCATGAGATGGACTGCACTGGGTGTGGCTTTGCCACTTGTGTTGCGAGTCTCCTGGTAAGAGACGGTATGT---------

>MH093737

----------------------------------------------------------------------------------------------------------------------------------------------------------------------------------------------------------------------------------------------------------------------------------------------------------------------------------------------------------------------------------------------------------------------------------------------------------------------------------------------------------------------------------------------------------------------------------------------------------------------------------------------------------------------------------------------------------------------------------------------------------------------------------------------------------------------------------------------------------------------------------------------------------------------------------------------------------------------------------------------------------------------------------------------------------------------------------------------------------------------------------------------------------------------------------------------------------------------------------------------------------------------------------------------------------------------------------------------------------------------------------------------------------------------------------------------------------------------------------------------------------------------------------------------------------------------------------------------------------------------------------------------------------------------------------------------------------------------------------------------------------------------------------------------------------------------------------------------------------------------------------------------------------------------------------------------------------------------------------------------------------------------------------CAACGAGGAATCCGCAAAAGATTACACTAAGTTTCTTAGAGACGAGTTGGTGGAACGGCTTGGCAAATGGCCAAAATTGAAAGATGTGGCCACAGCATGCTATGCTTTGTCAGTGATGTTCCCAGAGATAAAGAATGCCGAATTACCACCAATACTAGTGGATCATGAGAGTAAGTCAATGCACGTCATTGATTCATATGGATCACTCAGCGTTGGCTTTCACATTCTAAAGGCAAGTACTGTTGGACAACTGATAAAATTTCAGTATGAGTCATTGGAAAGTGAGATGCGCGAGTACATAGTGGGTGGCACTTTGACACAACAAACTTTCAGCACACTTCTTAAGACTCTCACAAAGAACATGTTTAAACCAGATAAAATAAAGCAGATAATTGAGGAAGAGCCATTCTTATTAATGATGGCAATTGCATCCCCAACTGTACTCATCTCGCTGTACAACAACTGCTACATCGAGCAAGCAATGACATATTGGATCGTCAAGAACCAAGGCATCGCAGCGCTTTTTGCGCAGTTGGAGGCACTAGCAAAGAAAACTTCTCAAGCGGAGCTACTAGTTCTTCAAATGCAAATACTTGAGAAAGCTTCGAACCAACTGAGACTTGCAGTCACGGGACTTAATCATGTTGATCCAGCTAAACGACTTTTATGGTCTCACCTGGAAGCTATGACAACACGGTCAGAGATGAATAAGGAACTCATAGCGGAAGGCTATGCACTGTACGACGAGCGCTTATACACTTTAATGGAAAAAAGTTACGTAGATCAATTAAACCAATCATGGGCAGAATTATCATATTGTGGAAAATTTTCAGCAATATGGCGTGTGTTCAGAGTCAGGAAATACTACAAACCATCTTTAACCGTGAGAAAAAGCGTAGATTTAGGCGCTGTGTACAATATATCAGCTACGCATCTAATATCAAATTTAGTGCAGAAAAGTCGAGATCAAGTCAGCTCTACTTTAACCAAACTCCGCAACGGTTTCTATGATAAAATGGAGAGAGCGAGAGTTAGTGCAGTAAGGACAGTATATTGGTTCGTACCCGACATATTTAGACTAATACATATTTGCTTAGTTTTAAGTATATTAACAACTATAGCTAATACAATAATCGCAATTATGAATGATTATAAAAAGTTGAAAAAGCAACAAAGAGAAGACGAATACGAAGCCGAGATTAACGAGGTGCGAAGGATACACGCCAACTTAATGAAGGAGCATAATGATGACTTAACATGCAAACAATTTATTGAGCACATACGACAGACACATCCGCGCCTCATTGAGGCAACATTGGATTTAACACATACAGGTGTCATCCATGAGGGCAAATCCAATTTAGAAACAAACCTCGAACAGGCAATGGCAGTGGGAACTTTACTCACTATGATACTCGATCCACAGAAGAGTGATGCAGTTTATAAGGTTCTCAATAAGATGCGAACAGTTATCAGCACAATAGAACAGAATGTACCATTTCCCTCAGTGAACTTCACGAGTATCTTGTCACCTCCTGTAACTCAGCAAAGTGTAGATGTTGACGAACCGTTGACACTGAGTACCGATAAGAATTTGACTATAGATTTCGACACAAATCAAGATTTGCCAGCGGACACATTTAGCAATGACGTTACGTTCGAGAACTGGTGGGCTAATCAGATAAACAACAACAGAACAGTGCCACACTATCGACTTGGGGGAAAGTTTGTAGAATTCACAAGAGAGAATGCAGCAATGGTTAGCATTGAGCTCGCCCATTCGAACATCGAAAAAGAGTTTCTACTCAGAGGAGCCGTTGGGTCAGGAAAATCCACAGGTTTGCCATATCATCTCAGTATGCGTGGAAAAGTGCTATTGATAGAACCTACGCGACCATTAGCTGAGAACGTTTGCAGACAACTGCAAGGTCCTCCATTTAATGTGAGCCCCACTTTACAAATGAGAGGATTGAGCACATTTGGCTGCACTCCTATCACGATAATGACATCTGGCTTCGCATTGCACATGTATGCTAATAACCCCGATAAGATCTCTGAGTATGACTTCATTATCTTTGATGAATGTCACATTATGGAAGCACCTGCAATGGCATTCTATTGTTTGCTTAAGGAGTATGAATATCGAGGCAAGATAATAAAAGTTTCAGCTACACCACCAGGACGAGAATGCGAGTTTTCAACCCAACATCCAGTAGATATACATGTATGTGAAAGCTTGACACAACAGCAATTCATCATGGAATTAGGAACAGGATCAACTGCTGATGCAACCAAATATGGCAATAACATATTAGTGTACGTTGCAAGTTATAATGATGTAGATTCTTTATCCCATGCTCTAACTGAACTTAAATATTCAGTGATTAAAGTCGATGGAAGAACGATGAAGCAGAACACCACAGGAATCGTAACAAATGGAACATCCAGTAAGAAATGCTTCGTTGTGGCCACAAATATTATTGAAAATGGTGTAACGCTAGACGTCGACGTTGTCGTCGACTTTGGACTTAAAGTAACAGCTGAATTAGATGTTGATAACAGGGCGATAATGTATAAACGTGTGAGCATATCTTATGGCGAGCGCATTCAGAGACTCGGAAGAGTCGGAAGGAACAAGCCTGGGACAGTTATCCGCATCGGAAAAACAATGAAAGGCTTACAAGAAATTCCTGCGATGATTGCTACTGAAGCAGCTTTCATGTGTTTTGCATATGGACTGAAAGTTATAACACATAATGTATCAACAACACATCTAGCAAAATGCACTGTCAAACAAGCTAGAACCATGATGCAATTCGAACTATCACCATTTGTAATGGCTGAATTAGTTAAATTCGATGGTTCCATGCATCCACAGATTCATGAAGCGTTAACTAAGTATAAATTAAGGGATTCTGTGATCATGTTAAGACCAAACGCAATACCAAAGGTTAACCTTCACAACTGGTTAACGGCCCGTGATTACAATAGGATAGGCTGCTCACTGGAACTCGAGGATCACGTTAAAATACCATATTATATACGAGGAGTTCCTGACAAATTGTATGGGAAGTTATATGATATCATCCTTCAATACAGCCCTACAAGTTGTTATGGAAGATTGTCAAGTGCTTGTGTGGGTAAGGTCGCATATACATTGCGCACTGATCCTTGTTCATTACCGAGAACAATAGCTATAATCAACGCACTGATTACTGAAGAATATGCAAAGAGAGATCATTACAGAAACATGATAGCAAACCCCTCATCATCGCACGCCTTCTCACTTAATGGGCTGGTATCCATGATCGCGTCTCGGTACATGAAAGATCACACGAAGGAGAACATAGATAAACTTGTAAGAGTGCGTGACCAGCTACTTGAGTTCCAAGGCACAGGTATGCAATTTCAAGATCCTTCAGAATTGATGGATATTGGTGCATTAAATACAGTTATCCACCAAGGAATGGACGCCACGGCTGCTTGCATTGGATTACAAGGGCGTTGGAATGCTTCACTCATCCAGCGCGATTTGATGATATCAGCAGGGATCTTCACAGGAGGAATTCTTATGATGTGGTGTCTCTTTACAAAATGGAGCAAGACAGAAGTGTCACATCAAGGAAAGAACAAACGCAGTAGACAAAAACTCCGATTCAAAGAAGCAAGAGACAACAAATATGCATATGATGTCACAGGATCGGAAGAGTGCCTTGGTGAAAATTTTGGAACAGCCTATACAAAGAAAGGTAAAGGAAAAGGAACTAAAGTTGGACTCGGTGTGAAGCAACACAAATTTCATATGATGTATGGTTTTGATCCTCAAGAGTACAACCTAATTCGATTTGTCGATCCACTCACAGGAGCAACTCTTGATGAGCAAATCCATGCCGACATACGCTTAGTTCAAGAGCATTTCGCTGAAATTCGTGAGGAGGCAGTAGCTAATGACACAATTGAAAAGCAGCATATCTACGGCAATCCTGGACTACAAGCATTTTTCATACAAAATGGTTCAGCAAACGCTCTGAGAGTTGATTTAACGCCACATTCACCTACACGAGTTGTCACAGGTAATAATATAGCAGGATTCCCAGAACACGAGGGTACACTTCGTCAAACTGGAACAGCAATAACCATACCCATTGGTCAAGTCCCAATCGCACGTGAAACAGGAGTTGCACACGAGTCAAAATCCATGATGAATGGGCTGGGTGACTACACACCAATATCGCAACAGTTATGTTTAGTACAAAATGACTCAGATGGGGTAAAGCGGAATGTGTTTTCAATTGGATATGGTTCATATCTCATTTCACCAGCGCACTTATTCAAATATAACAATGGCGAAATAACAATTAGATCATCAAGAGGATTGTATAAAATTCGTAATTCTGTGGATTTAAAACTACATCCGATTGCACACAGAGACATGGTCATAATTCAGCTTCCAAAGGATTTCCCACCGTTCCCAATGCGTTTGAAATTCACACAACCATCACGAGATATGCGAGTCTGCTTAGTGGGAGTCAATTTCCAACAGAACTACAGCACTTGCATCGTATCAGAAAGTAGTGTGACAGCACCAAAAGGAAATGGAGACTTTTGGAAACATTGGATATCAACAGTCGACGGTCAATGTGGATTACCATTGGTAGATACTAAGAATAAACACATTGTCGGAATTCATAGTCTTGCATCTACAAGTGGAAACACCAACTTCTTTGTCGCCATGCCTGAGAGCTTTAATGAATACATTAATGAACTTGTGCAAACAAACAAATGGGAAAAGGGATGGCACTACAATCCGAATCTCATATCTTGGTGTGGATTAAACTTAGTTGACTCCGCTCCAAAGGGTCTGTTTAAAACGTCAAAATTGGTTGAAGATTTGGATGCTAGTGTCGAAGAGCAGTGCAAAGTTACTGAAACATGGCTCACAGAGCAATTACAAGATAATTTGCAAGTGGTCGCGAAATGTCCAGGCCAACTCGTAACTAAGCATGTCGTAAAAGGCCAATGCCCGCACTTCCAATTATATTTGTCAACACATAATGATGCCAAAGAATATTTCGCACCTCTGCTTGGAAAATATGACAAGAGCAGACTCAATAGAGCGGCATTTATCAAAGACATATCAAAGTATGCAAAGCCAATTTATATTGGAGAAATAAACTATGATATCTTTGATAGAGCTGTACAACGAGTCATTAATATTCTTAAAAATGTCGGAATGCAACAATGCGTTTATGTTACAGACGAAGAAGAAATTTTTAAATCACTCAACCTAAACGCAGCTGTCGGAGCACTGTACACAGGAAAGAAGAAAGATTACTTTGAAAGTTTTTCAAATGAAGACAAGGAAGAAATCGTGATGAGATCATGTGAACGCATTTACAATGGACAACTTGGTGTGTGGAATGGTTCACTCAAAGCCGAAATCAGACCAATAGAGAAAACCATGTTAAATAAGACTCGAACCTTTACAGCAGCCCCATTGGAAACTTTGCTTGGAGGAAAAGTGTGTGTGGACGATTTTAATAATCAATTTTATTCGCACCATTTAGAAGGCCCATGGACTGTTGGGATAACAAAATTCTATGGAGGTTGGAATCGCTTACTTGAGAAGTTGCCAGAAGGATGGGTTTACTGCGACGCCGACGGATCTCAATTCGATAGTTCATTAACACCATATCTTATTAATGCAGTGCTGAATATTCGATTACAGTTCATGGAAGATTGGAACATAGGAGCGCAAATGCTTAAAAACCTTTATACCGAGATTGTTTATACACCAATTGCAACACCAGATGGATCTATCGTGAAGAAATTCAAAGGAAACAATAGTGGACAACCTTCTACAGTAGTTGATAATACATTGATGGTTATAATAGCTTTCAACTATGCTATGCTATCGAGTGGTATTAGAGAAGAAGAGATTGATAACTGCTGTAGGATGTTTGCAAATGGTGATGACCTGCTCCTAGCAGTACATCCTGATTTTGAATACATTCTAAACGGATTTCAAGATCACTTCGGAAATCTCGGATTGAATTTTGAGTTTACATCACGAACACGAGATAAATCCGAACTATGGTTCATGTCTACGAGAGGAATCAAATGTGAAGGAATCTACATACCTAAACTCGAGAAAGAAAGAATAGTCGCAATACTCGAATGGGATCGATCAAATCTACCTGAGCATAGATTGGAAGCCATTTGTGCAGCTATGGTTGAAGCATGGGGCTACTCAGATCTTGTTCACGAAATTCGGAAATTTTATGCGTGGCTTCTAGAAATGCAACCTTTCGCAAACCTAGCAAAAGAAGGCATGGCACCATACATAGCAGAAACAGCGCTCCGTAACCTTTATCTTGGGACGGGTATTAAAGAAGAAGAAATCGAGAAATATTTTAAGCAATTTGTTAAGGATCTTCCTGGATACATAGAAGATTACAATGAAGAAGTCATTCACCAATCGGGAACAGTTGATGCAGGTGCACAAGGTGGCAGCGGAAGCCAAGGAACAACACCACCAGCAACAGGTAGCGGAGCACGGACAGCTACTTCAGGAG------------CAGGATCTGGTAGCGGAAC----AGGGACTGGAACCGGTGCAACTGGAGGCCAAACAGGA---------GCT---G---GCAGTGGTGCTGGAACAGGATCTGGAGCGACCGGAGGTCAATCAGGATCTGGAAGTGGCGCTGGACAGACTGGCACAGGCTC---AGCAGGAACTGGTGCA------------ACGGGAGGCCAGAGAGATAAGGACGTAGATGCAGGTACAACAGGAAAAATTTCTGTACCAAAGCTCAAGGCCATGTCAAAGAAAATGCGCTTACCGAAAGCAAAAGGAAAAGATGTCTTGCATCTGGACTTCTTGCTTACATACAAGCCACAGCAGCAAGACATATCAAACACAAGAGCAACTAAGGAAGAGTTTGATAGATGGTACGATGCCATAAAGAAGGAATACGAGATTGATGACACACAAATGACAGTTGTCATGAGCGGTCTTATGGTGTGGTGCATTGAGAACGGTTGCTCACCAAACATAAACGGAAATTGGACAATGATGGATGGAGATGAACAAAGAGTCTTTCCACTCAAACCAGTCATCGAAAACGCATCTCCAACTTTCCGACAAATTATGCATCATTTTAGTGATGCAGCTGAAGCGTACATAGAGTACAGAAACTCTACTGAGCGATACATGCCAAGATACGGACTTCAGCGCAATCTCACCGACTATAGCTTAGCACGGTATGCATTTGATTTCTATGAAATGACTTCACGCACACCTGCTAGAGCTAAAGAAGCCCACATGCAG---ATGAAAGCCGCAGCAGTTCGTGGTTCAAACACACGACTGTTCGGTCTGGACGGAAATGTTGGCGAGACCCAGGAGAATACAGAGAGACACACAGCTGGCGACGTTAGTCGCAATATGCACTCTCTGTTGGGAGTGCAGCAGCACCACTAGTTTCCTGGAAACCCTGTTTGCAGTACCTATAATATGTATTA------ATATATAGTATGCCAGTGAGGTTTTACCTC-----------------------GTCTTCACTA-TTTGTTATGTATGTATTTAAAGCGTGAACCAGTCTGCAGCATACAGGGTTGGACCCAGTGTGTTCTGGTGTAGCGTGTACTAGCGTCGAGCCATGAGATGGACTGCACTGGGTGTGGCTTTGCCACTTGTGTT-CG----------------------------------

>MH093738

--------------------------------------------------------------------------------------------------------------------------------------------------------------------------------------------------------------------------------------------------------------------------------------------------------------------------------------------------------------------GCAAAACCAAAACAGAAGCAGGCAACTCAACAGATGTGGGTTGAGAAATGTGATCACAATCCTGTTGATCACTTAGTATATCCACGATTTGAAAGATCTATCAACAAAGTG---GATACGAATATCAAAAGTGCATCTGTAAGCAAGCTAACCAGAGAGATTTTAGAAATCTCAAAGGCAAGCGGCCTTAAAGTTGAACTAATTGATAAGCGTAAAAGATCTAAAACACAGTTATCAATCAAAAGGTTCAATGGCAAGGACTTTCTCCACTGCAAAACAAACCACGAAAACAATTTGTTTAAGAGGAAGGACATAGCTATTGGACACAAATGGCTTCCAACAATCGAAGCTATTGCTCGCTGTTATAGTACAATGAACCAAAAGGAATTGCAAAGCCTTTATAAAGGCAGCAGTGGTCTCACATTCATTCAAAATGATGAACTGTTCGTTGTTAGAGGGAGAATGAATGGTGAACTAGTCAATAGCCTATGTGAAACAGACCGGGTTACGGATATTGAACACTATGCAGATCCCCAAGCTAATGATTTTTGGAGAGGATACACAAATGCTTATGTGGATAATCGTAGTATTTCTACCACCCACACAGAGCACACCCCGACAATCAATCTAGAAGAGTGTGGAAAACGAATGGCTCTACTTGAGATATTATTCCACTCCACATTCAAGATTACATGTAAAACATGCAACATGGATGATCTTGAATTATCAGATGATGAGTTCGGAGCTAAACTCTACAAAAATCTGCAACGCATCGAAGAGAAGCAACGAGAGTACCTTGCCAAGGATCAAAAGTTATCCAGAATGATACAATTTATCAAAGAAAGATGCAACCCAAAATTCTCACATTTACCAATGTTGTGGCAAGTTGCAGAAACAATAGGACACTATACTGATAATCAGTCAAAGCAAATAATGGATATCAGCGAAGCGCTCATCAAAGTTAATACTTTAACTCCTGATGACGCTATGAAAGCAAGCGCAGCATTACTTGAAGTGTCACGATGGTATAAGAATCGTAAGGAGTCGCTCAAAACCGACTCATTGGAATCTTTTAGAAATAAAATATCACCAAAGAGTACAATAAATGCAGCTCTAATGTGTGACAATCAATTGGATAAAAATGCAAATTTTGTATGGGGTAATAGGGAATATCACGCTAAACGATTTTTCGCAAACTACTTTGAAGCAGTGGACCCCACAGATGCATATGAAAAGCACGTCACACGGTTTAACCCTAATGGTCAACGGAAGCTATCAATAGGAAAGTTAGTTATCCCACTAGACTTTCAAAAGATTAGAGAATCATTCGTTGGACTTCCGATCAATAGACAACCGCTAGACAAATGTTGCGTTAGTAAGATCGAAGGGGGATATATATACCCATGTTGCTGCGTCACAACAGAATTTGGTAAACCAGCATACTCTGAGATAATACCTCCGACAAAAGGCCACATAACAATAGGCAATTCGATTGATCCAAAGATTGTGGACTTGCCAAACACAACACCACCCAGCATGTACATTGCTAAGGATGGGTACTGTTATATCAACATCTTTCTAGCAGCCATGATCAACGTTAATGAAGAATCTGCCAAGGATTACACGAAATTCTTGAGAGATGAACTAGTCGAGCGTCTTGGAAAGTGGCCAAGGCTTAAAGACGTAGCAACAGCGTGTTATGCATTATCTGTAATGTTTCCAGAAATTAAGAATGCTGAGCTACCTCCAATTTTAGTTGATCATGAAAATAAATCAATGCACGTCATCGACTCATATGGTTCACTAAGCGTTGGATTTCACATATTGAAAGCAAGCACGATTGGTCAATTAATTAAATTTCAATATGAATCTATGGATAGTGAAATGCGCGAATACATAGTAGGAGGAACTCTTACGCAACAGACATTCAACACACTTCTTAAGATGCTCACGAAAAACATGTTCAAACCAGAGCGCATTAAGCAGATAATTGAGGAGGAACCCTTCTTGCTTATGATGGCAATCGCATCTCCAACGGTATTAATAGCACTATATAATAATTGTTACATTGAGCAAGCTATGACATACTGGATCGTGAAGAATCAAGGAGTTGCAGCCATATTCGCACAACTCGAAGCATTAGCCAAGAAAACATCCCAGGCTGAATTGCTAGTTCTACAAATGCAGATACTTGAAAAAGCATCTAGTCAACTAAGATTAGCAGTTTCAGGACTTAGCCATGTCGACCCAGCAAAGCGACTTTTGTGGTCACACCTCGAAGCAATGACAACACGGTCAGAAATGAACAAGGAGTTGATAGCTGAGGGATATGCACTATACGACGAGCGTCTATATACCCTGATGGAAAAAAGTTACGTAGATCAATTAAACCAGTCATGGGCAGAATTATCATACTGTGGAAAATTTTCAGCAATATGGCGTGTGTTCAGAGTCAGGAAATACTACAAACCGTCTTTAACCGTGAGAAAAAGCGTAGATTTAGGCGCTGTGTACAATATATCAGCTACGCATCTAATATCAAATTTAGTGCAGAAAAGTCGAGATCAAGTCAGCTCTACTTTAACCAAACTCCGCAACGGTTTCTATGATAGAATGGAGAGAGCGAGAGTTAGTGCAGTAAGGACAGTATATTGGTTCGTACCCGATATATTTAGACTAATACATATTTGCTTAGTTTTAAGTATATTAACAACTATAGCTAATACAATAATCGCGATTATGAATGATTATAAAAAGTTGAAAAAGCAACAAAGAGAAGACGAATACGAAGCCGAGATTAACGAGGTACGAAGGATACACGCCAACCTAATGAAGGAGCATAATGATGACTTAACATGTGAACAATTTATTGAGCACATACGACAGACACATCCACGCCTCATTGAGGCAACATTGGATTTAACACATACAGGTGTCATCCATGAGGGCAAATCCAATTTAGAAACAAACCTCGAACAGGCAATGGCAGTGGGAACTTTACTCACTATGATACTCGATCCACAGAAGAGTGACGCAGTTTATAAGGTTCTCAATAAGATGCGAACAGTTATCAGCACAATAGAACAGAATGTACCATTTCCCTCAGTGAACTTCACGAGCATCTTGTCACCTCCTGTAACTCAGCAAAGTGTAGATGTTGACGAACCGTTAACACTGAGCACCGATAAGAATTTGACTATAGATTTCGACACAAATCAAGATTTGCCAGCGGACACATTTAGCAATGACGTTACGTTCGAGAACTGGTGGGCTAATCAGATAAACAACAACAGAACAGTGCCACACTATCGACTTGGGGGAAAGTTTGTAGAATTCACAAGAGAGAATGCAGCAATGGTTAGCATTGAGCTCGCCCATTCGAACATCGAAAAAGAGTTTCTACTCAGAGGAGCCGTTGGGTCAGGAAAATCCACAGGCTTGCCATACCATCTCAGTATGCGTGGAAAAGTGCTATTGATAGAACCCACTCGACCATTAGCTGAGAACGTTTGCAGACAACTGCAAGGTCCTCCATTTAATGTGAGCCCCACTTTACAAATGAGAGGATTGAGCACATTTGGCTGCACTCCTATCACGATAATGACATCTGGCTTCGCATTGCACATGTATGCTAATAACCCCGATAAGATCTCTGAGTATGACTTCATTATCTTTGATGAATGTCACATTATGGAAGCACCTGCAATGGCATTCTATTGTTTGCTTAAGGAGTATGAATATCGAGGCAAGATAATAAAAGTTTCAGCTACACCACCAGGACGAGAATGCGAGTTTTCAACCCAACATCCAGTAGATATACATGTATGTGAAAGCTTGACACAACAGCAATTCATCATGGAATTAGGAACAGGATCAACTGCTGATGCAACCAAATATGGCAATAACATATTAGTGTACGTTGCAAGTTATAATGATGTAGATTCTTTATCCCATGCTCTAACTGAACTTAAATATTCAGTGATTAAAGTCGATGGAAGAACTATGAAGCAGAACACCACAGGAATCGTAACAAATGGAACATCCAGTAAGAAATGCTTCGTTGTGGCCACAAATATTATTGAAAACGGTGTAACGCTAGATGTCGACGTTGTCGTCGACTTTGGACTTAAAGTAACAGCTGAATTAGATGTTGATAACAGGGCGATAATGTATAAACGTGTGAGCATATCTTATGGCGAGCGCATTCAGAGACTCGGAAGAGTCGGAAGGAATAAGCCTGGGACAGTTATCCGCATCGGAAAAACAATGAAAGGCTTACAAGAAATTCCTGCGATGATTGCTACTGAAGCAGCTTTCATGTGTTTTGCATATGGACTGAAAGTTATAACACATAATGTATCAACAACACATCTAGCAAAATGCACTGTCAAACAAGCTAGAACCATGATGCAATTCGAACTATCACCATTTGTAATGGCTGAATTAGTTAAATTCGATGGTTCCATGCATCCACAGATTCATGAAGTGTTAACCAAGTATAAATTGAGGGATTCTGTGATCATGTTAAGACCAAACGCAATACCAAAGGTTAACCTTCACAACTGGTTAACGGCCCGAGATTACAATAGGATTGGCTGCTCACTGGAACTCGAGGATCACGTTAAGATACCATATTATATACGAGGAGTTCCTGACAAGTTGTATGGGAAGTTATATGATATCATCCTTCAATACAGCCCTACAAGTTGTTATGGAAGATTGTCAAGTGCTTGCGTAGGTAAGGTCGCATATACATTGCGCACTGATCCTTGTTCATTACCGAGAACAATAGCTATAATCAACGCACTGATTACTGAAGAGTATGCAAAGAGGGACCACTACAGAAACATGATAGCAAACCCCTCATCATCACACGCCTTCTCACTTAATGGACTAGTATCCATGATCGCGTCTCGGTACATGAAAGATCACACGAAGGAGAACATAGATAAACTTGTAAGAGTGCGCGACCAGCTACTTGAGTTCCAAGGCACAGGCATGCAATTTCAAGATCCTTCAGAATTGATGGATATTGGAGCATTAAATACAGTTATCCACCAAGGAATGGACGCCACGGCCGCTTGCATCGGATTACAAGGGCGTTGGAATGCTTCACTCATCCAGCGCGATTTGATGATATCAGCAGGGATCTTCACAGGAGGAATTCTTATGATGTGGTGTCTCTTTACAAAATGGAGCAAGACAGAAGTGTCACATCAAGGAAAGAACAAACGCAGTAGACAAAAACTCCGATTCAAAGAAGCAAGAGACAACAAATATGCATATGATGTCACAGGATCGGAAGAGTGCCTTGGTGAGAATTTCGGTACAGCCTATACAAAGAAAGGTAAAGGAAAAGGAACTAAAGTTGGACTCGGTGTGAAACAACACAAATTTCATATGATGTATGGTTTTGATCCTCAAGAGTACAACCTAATTCGATTTGTTGATCCACTCACAGGAGCAACTCTTGATGAGCAAATCCATGCCGACATACGCTTAATTCAAGAGCATTTCGCTGAGATTCGTGAGGAGGCAGTAGTTAACGACACAATTGAAAGGCAGCAAATTTACGGCAATCCTGGATTACAAGCATTTTTCATACAGAATGGGTCAGCAAATGCTTTAAGAGTTGATTTAACACCACATTCACCTACACGGGTTGTCACAGGCAATAACATAGCAGGATTCCCAGAATATGAAGGTACACTCCGTCAAACTGGAACAGCTATAACCATACCCATTGGTCAAGTCCCAATTGCAAATGAAGCAGGAGTCGCACACGAATCAAAATCCATGATGAATGGATTGGGTGATTACACGCCAATATCACAACAATTGTGTTTAGTGCAAAATGACTCAGATGGAGTAAAACGAAATGTGTTTTCAATCGGATATGGCTCATATCTCATTTCACCAGCGCACTTATTTAAATATAACAATGGCGAAATTACAATTAGATCATCAAGAGGATTGTATAAAATTCGCAACTCTGTGGATTTAAAATTACATCCGATTGCACACAGAGACATGGTCATAATCCAACTTCCAAAGGATTTCCCACCGTTCCCAATGCGTTTGAAATTTACACAACCATCACGAGAGATGCGAGTCTGCTTAGTAGGAGTCAATTTCCAACAGAACTATAGCACTTGCATTGTATCAGAAAGCAGCGTAACAGCACCAAAGGGGAATGGAGATTTTTGGAAGCATTGGATTTCAACAGTCGACGGTCAATGTGGACTACCATTGGTAGATACTAAGAACAAACACATTGTTGGAATCCATAGTCTTGCATCTACAAGTGGGAACACCAATTTCTTTGTTGCCATGCCTGAGAACTTTAATGAATACATTAATGGACTTGTGCAAACAAACAAATGGGAAAAGGGATGGCACTACAATCCGAATCTCATATCTTGGTGTGGATTAAACTTAGTCGATTCTGCTCCAAAGGGTTTGTTTAAAACGTCAAAATTAGTTGAAGATTTGGATGCTAGTGTTGAAGAGCAGTGCAAGGTTACTGAAACATGGCTCACAGAGAAATTACAAGATAATTTGCAAGTGGTCGCGAAATGCCCAGGCCAACTCGTCACTAAGCATGTTGTCAAAGGTCAATGCCCACACTTCCAGTTATATTTGTCAACACATAATGATGCCAAAGAATATTTCGCACCCCTGCTTGGAAAATATGATAAGAGCAGACTCAATAGAGCGGCATTTATCAAAGACATATCAAAGTATGCAAAACCAATTTATATTGGAGAAATCAATTATGATATCTTTGATAGAGCTGTACAACGAGTCATTAATATCCTTAAAAATGTTGGAATGCAACAATGCGTTTATGTCACAGACGAAGAAGAAATTTTTAAATCACTCAACCTAAACGCAGCTGTCGGAGCACTGTACACAGGAAAGAAGAAAGATTACTTTGAAAGTTTTTCAAATGAAGACAAAGAAGAAATCGTGATGAGATCATGTGAACGCATTTACAATGGACAACTTGGTGTGTGGAATGGGTCACTCAAAGCCGAAATCAGACCAATAGAGAAAACCATGTTAAATAAGACTCGAACTTTTACAGCAGCTCCATTGGAAACTTTGCTTGGAGGAAAAGTGTGTGTGGACGATTTTAATAATCAATTTTATTCACACCATTTAGAAGGCCCATGGACTGTTGGGATAACAAAATTCTATGGAGGTTGGAATCGCTTACTTGAGAAGTTGCCGGAAGGATGGGTTTACTGCGACGCTGACGGATCTCAATTCGATAGTTCATTAACACCTTATCTTATTAATGCAGTGTTGAATATTCGATTACAGTTCATGGAGGATTGGAACATAGGAGCGCAAATGCTTAAAAACCTTTATACTGAAATTGTTTATACACCAATTGCAACACCAGACGGATCTATCGTGAAGAAATTCAAAGGAAATAATAGTGGACAACCTTCTACAGTAGTTGATAATACATTGATGGTTATAATAGCTTTCAACTATGCCATGCTATCGAGCGGTATTAGAGAAGAAGAGATTGATAACTGCTGTAGGATGTTTGCAAATGGTGATGACCTGCTCCTAGCAGTACATCCTGATTTTGAATACATTCTAAACGGGTTTCAAGATCACTTCGGAAATCTTGGATTGAATTTTGAGTTTACATCACGAACACGAGATAAATCCGAACTATGGTTTATGTCTACAAGAGGAATCAAATGTGAAGGAATCTACATACCTAAACTCGAGAAAGAAAGAATAGTCGCAATACTCGAATGGGATCGATCAAATCTACCTGAGCATAGATTGGAAGCCATTTGTGCAGCTATGGTTGAAGCATGGGGCTACTCAGATCTTGTCCACGAAATTCGGAAATTTTATGCGTGGCTTCTAGAAATGCAACCTTTCGCAAATCTAGCAAAAGAAGGAATGGCGCCATACATAGCAGAAACAGCGCTCCGTAACCTTTATCTTGGAACGGGCATTAAAGAAGAAGAAATCGAGATATATTTCAAGCAATTTGTTAAGGATCTTCCCGGATACATAGAAGATTACAATGAAGAAGTCATTCACCAATCGGGAACAGTTGATGCAGGTGCACAAGGCGGCAGCGGAAGCCAAGGAACAACACCACCAGCAACAGGTGGCGGATCAAGACCAGCGGCTTCAGGAG------------CAGGATCTGGTAGCGGAAC----AGGGACTGGAACCGGTGCAACTGGGGGCCAGACAGGA---------ACT---G---GTAGTGGTGCTGGAACAGGATCTGGAGCGACCGGAGGCCAATCAGGATCTGGAAGTGGCACTGGACAGACTGGCACAGGCTC---AGCAGGAACTGGTGCA------------ACAGGAGGCCAGAGAGATAAGGATGTAGATGCAGGTACAACAGGAAAAATTTCTGTACCAAAGCTCAAGGCCATGTCAAAGAAAATGCGCTTACCGAAAGCAAAAGGAAAAGATGTCTTGCATCTGGACTTCTTGCTTACATACAAGCCACAGCAGCAAGACATATCAAACACAAGAGCAACTAAGGAAGAGTTTGATAGATGGTACGATGCCATAAAGAAAGAATACGAGATTGATGACACACAAATGACAGTTGTCATGAGCGGTCTTATGGTGTGGTGCATTGAGAACGGTTGCTCACCAAACATAAACGGAAATTGGACAATGATGGATGGAGATGAACAAAGAGTYTTTCCACTCAAACCAGTCATTGAAAACGCATCCCCAACTTTCCGACAAATTATGCATCATTTTAGTGATGCAGCTGAAGCGTACATAGAGTACAGAAACTCTACTGAGCGATACATGCCAAGATACGGACTTCAGCGCAATCTCACCGACTATAGCTTAGCACGGTATGCATTTGATTTCTATGAAATGACTTCACGCACACCTGCTAGAGCTAAAGAAGCCCACATGCAG---ATGAAAGCCGCAGCAGTTCGTGGTTCAAACACACGACTGTTCGGTCTGGACGGAAATGTCGGCGAGACCCAGGAGAATACAGAGAGACACACAGCTGGCGACGTTAGTCGCAATATGCACTCTCTGTTGGGAGTGCAGCAGCACCACTAGTCTCCTGGAAACCCTGTTTGCAGTACCTATAATATATACTA------ATATATAGTATSTCAGTGAGGTTTTACCTC-----------------------GTTTTTACTA-TTTGTTATGTATGTATTTAAAGCGTGAACCAGTCTGCAGCATACAGGGTTGGACCCAGTGTGTTCTGGTGTAGCGTGTACTAGCGTCGAGCCATGAGATGGACTGCACTGGGTGTGGCTTTGCCACTTGTGTTGCGAGTCTCCTGGTAAGAGA-----------------

>EU091075

--------------------AAAAACAACAAAACTCAACACAACACAACAAAACACAACCAAACAAAACCAAGTTACTTTTGCTCAGATTGTAGTGAACGGCTCGGCGGGAAAGGTTCCTCGAGATCACTCTCTGACTCTTCTCTT----------TCAACCAACTTCATTCAAGCGAAATGGCGGGCTCTTGGACTCACGTGACATACAAGTGGCAACCAGATGTCAACAACGCACGTGACGTGAAAAGAGTAATGGAGATGTTTGCAGCAAAACATCAACGTTACACTGAGGAGCAGAGGCTTGCTCACAACAGCAAGCTATTAAGGAAGGCTTGTGTTACTAGTGCTGAGTTTGCTGAATCAGCACAGAAACCAAAATGTCATCAGATATGGGTTGAAAAGTGCGACCACAACCCCACGGAGCACTTTGTTTATCAACGCTTC---ACACCTGAGAAGAAAGTGCTAGCCATCAAACCTGAAACAACTTCTGTCACGAAGTTAATCAGGGATGTCCTAGAAATTTCGAAGAGCAGTGGGATAAAAATTGAGTTAATTGATAAGCGTGTCAAACGTAAAACTCAATTATCCATAAGGCAACACAATGGCAAAGATTTCTTGCACTACAAAACTAGGCATGAAAATGGTCTGTTTAAACGCAAGGACGTTGACATTAGTGTTAAGTGGTTGCCCATCATTGAAGCCATTGCAAAATGCTACAGCACGGTGAATGCAGAAGAACTGCAAAGTCTCAATAGAGGCAGTAGTGGTCTAACATTCATGCAAAACGATGAATTATTCATCGTGCGTGGAAGAATGCATGGTGAGATTGTCAATAGTTTACATGAGAATAAGCACGTAATGGAAATTGAACACTATGCTGATCCACAAGCAAACAGTTTTTGGAAAGGATACACAGATGCGTATGTCGAGAACAGAAACATATCTACTACTCACACAGAGCACACACCAACTATTAATTTAGAAGAATGTGGTAAGAGAATGGCACTGTTAGAAATCCTATTCCATTCAACTTTTAAGATAACATGTAAAACGTGTAATATTGATGACCTTGAATTATCGGATGATGAATTTGGGGCCAAGTTATATAGTAATCTGCAGCGCATTGAAGAAAAACAGCGTGAATATCTTGCTAAAGATCAAAAACTTTTACGCATGCTTCACTTTATAAAGGACCGATGCAATCCAAAATTTTCACATTTGCCCTTGCTATGGCAAGTGGCAGAAACAGTAGGACATTACACTGATAATCAATCGAAGCAGATAATTGATATCAGTGAGGCGCTCATTAAAGTCAATACCTTAACCCCTGATGATGCAGTAAAGGCCAGTGTAGCATTATTGGAAGTAGCACGATGGTATAAAAATCGGAAGGAATCACTCAAAACAGACACATTGGACTCATTCCGAAACAAGATTTCACCAAAAAGCACAATTAACGCAGCATTGATGTGTGACAACCAGTTAGATAAGAATGCAAATTTCGTATGGGGAAACAGAGAATACCATGCAAAGCGATTCTTCGCTAATTATTTTGAAGCTGTAGATCCAACTGATGCATATGAAAAACACGTTACACGCTTTAATCCCAATGGACAGCGTAAGTTATCAATTGGCAAATTGGTAATTCCACTGGACTTCCAGAAAATAAGAGACTCGTTTGTCGGCCTATCGATAAATAAACAACCACTAAGCAAAGCTTGTGTGAGCAAGATTGATGGGGGTTACGTATATCCATGTTGCTGCGTCACAACGGAATTCGGAAAACCAGCATACTCTGAAATAATACCTCCAACAAAAGGACATATTACGATTGGAAATTCAGTGAACCCAAAGATAGTGGATTTACCGAACACAACACCACCGAGCATGTACATTGCAAAAGATGGATATTGTTATATTAACATATTCTTAGCAGCAATGATAAATGTTAATGAGGAATCCGCAAAAGACTACACTAAGTTCCTTAGAGATGAGCTTGTAGAACGGCTTGGCAAATGGCCTAAACTAAAAGATGTAGCAACAGCATGTTACGCTCTATCAGTGATGTTCCCAGAAATCAAGAATGCTGAATTACCACCAATATTAGTTGATCATGAGAGCAAGTCAATGCATGTCATCGATTCATATGGCTCACTTAGTGTTGGATTTCACATTCTAAAGGCAAGTACTGTGGGACAACTGATAAAATTCCAGTATGAATCATTAGAAAGCGAGATGCGTGAATACATAGTGGGGGGCACTTTGACACAGCACACTTTCAGCACACTTCTCAAGACTCTCACAAAGAACATGTTTAAGCCAGGTAAGATTAGACAGATAATAGAGGAAGAGCCTTTTCTACTAATGATGGCGATTGCATCCCCAACCGTACTTATCTCACTGTATAACAACTGCTACATCGAACAGGCAATGACATATTGGATTGTTAAGAACCAAGGAGTTGCAGCAATTTTTGCACAGTTGGGGGCTTTGGCGAAGAAAACCTCTCAGGCAGAACTATTAGTTCTGCAAATGCAAATACTTGAAAAAGCTTCAAGCCAACTAAGACTTGCAGTCACCGGACTTAATCATGTTGATCCAGCGAAACGACTTTTGTGGTCGCACCTAGAAGCCATGACAACACGATCGGAAATGAACAAGGAACTCATAGCGGAAGGTTATGCACTGTACGACGAGCGTCTATATACTCTAATGGAAAAAACTTATGTAGATCAATTAAACCAATCGTGGGCAGAACTATCATACTGTGGAAAATTTTCAGCAATATGGCGTGTGTTCAGAGTCAGGAAATATTACAAGCCATCTTTAACCGTGAGAAAAAGCGTAGATTTAGGCGCTGTTTACAATATATCAGCTACGCATCTAATATCAGATTTAGTGCAGAGAAGTCGCGATCAAGTCAGCTCTACTTTAACCAAACTCCGCAACGGTTTCTATAACAGAGTGGAGAAAGCGAGGGTTAGTGCAGTGAGGACAGTGTATTGGTTTATACCTGATATATTCAGATTAGTTCATATCTTTATAGTTCTAAGCTTATTAACTACTATAGCTAATACGATAGTCACAACTATGAATGACTACAAAAAGTTAAAGAAACAACAAAGAGAGGATGAGTATGAAGCTGAAATTAATGAGGTGCGGAAAATACACGCCACGTTGATGAAAGAGCACAACGATAATCTGACATGTGATCAATTCATTGAATACATACGGCAAACACACCCACGCCTCATCGAAGCAACTTTAGATTTAACACATACAGGTGTTATTCACGAAGGTAAATCCAACCTAGAAACAAATCTCGAACAGGCGATGGCAGTGGGAACTTTATTAACTATGATACTCGACCCACAAAAGAGTGATGCAGTATACAAAGTTCTCAATAAGATGCGAACAGTGATTAGCACAATTGAGCAGAATGTACCATTTCCAGCAGTTAATTTTACAAGCATCTTAACACCTCCTGTGACACAGCAAAGCGTGGATGTTGATGAACCATTGACATTGAGTACTGACAAGAATCTGACTATAGATTTTGATACAAACCAAGATTTGCCAGCAGATACATTTAGCAATGACGTTACATTCGAGAACTGGTGGGCCAACCAGATAAACAACAACAGAACAGTGCCACACTATCGACTTGGGGGAAAGTTTGTAGAATTCACAAGAGAGAATGCAGCAATGGTTAGCATTGAGCTCGCTCACTCGAACATCGAAAAAGAATTTCTACTTAGAGGAGCTGTTGGTTCGGGAAAATCCACAGGTTTGCCATATCATCTCAGTATGCGTGGAAAAGTGCTGTTGATAGAACCCACTCGACCGTTGGCTGAGAACGTTTGCAGGCAACTGCAAGGTCCCCCATTCAACGTGAGTCCCACTTTACAAATGAGAGGGTTGAGCACGTTTGGCTGCACTCCTATCACGATAATGACGTCTGGCTTCGCATTACACATGTATGCTAATAACCCTGACAAGATTTCTGAATACGATTTCATTATCTTTGATGAATGTCACATCATGGAAGCACCTGCAATGGCATTTTATTGTTTGCTTAAGGAGTATGAATACCGAGGCAAGATAATAAAAGTTTCAGCTACACCACCAGGACGAGAATGCGAATTTTCAACCCAACATCCAGTAGATATACATGTATGTGAGAGCTTGACACAACAGCAATTCGTTATGGAACTAGGAACAGGATCGACTGCTGATGCAACCAAATATGGCAATAACATATTGGTGTACGTTGCAAGTTACAATGACGTAGATTCTTTATCCCATGCTCTAACTGAACTTAAGTATTCAGTGATTCAAGTCGACGGAAGAACGATGAAGCAGAAATCCACAGGGATCGTTACAAATGGAACATCCAGTAGGAAATGCTTCGTTGTGGCCACGAATATTATTGAAAACGGTGTAACGCTAGATGTCGATGTCGTCGTCGACTTTGGACTTAAAGTAACAGCTGAATTAGATGTTGATAACAGGGCAATAATGTATAAACGTGTGAGCATATCTTATGGCGAGCGCATTCAGAGACTTGGAAGAGTTGGAAGGAATAAGCCTGGGACAGTAATCCGCATCGGGAAAACAATGAAAGGTTTACAAGAAATTCCAGCGATGATTGCAACTGAAGCGGCTTTCATGTGTTTCGCATATGGACTGAAAGTTATAACACATAATGTATCAACAACACATCTGGCAAAATGCACTGTCAAACAAGCTAGAACCATGATGCAATTTGAACTGTCACCATTTGTAATGGCTGATTTAGTTAAATTTGACGGCTCTATGCACCCACAGATTCATGAAGCGTTAACCAAGTACAAATTGAGAGATTCTGTGATCATGTTAAGACCAAATGCAATACCAAAGGTTAATCTTCATAACTGGCTGACGGCTCGTGACTACAACAGGATAGGCTGCTCATTGGAACTCGAAGACCACGTTAAAATACCATATTATATACGGGGAGTTCCTGACAAGCTGTACGGGAAGTTGTATGATATCATCCTTCAATATAGCCCTACAAGTTGCTATGGAAGACTATCAAGTGCTTGTGTAGGCAAGGTTGCATATACATTGCGCACTGATCCTTGTTCGTTACCAAGAACAATAGCTATAATCAACGCACTGATTACTGAAGAATACGCAAAGAGGGATCATTACAGAAACATGATAGCGAATCCTTCATCATCACACGCTTTCTCACTTAATGGACTAGTATCCATGATCGCTTCTCGGTACATGAAAGACCACACGAAGGAAAATATTGAAAAACTCGTAAGAGTACGCGATCAACTACTTGAGTTCCAAGGCACAGGCATGCAGTTTCAAGATCCTTCAGAATTAATGGATATCGGTGCATTAAACACAGTTATTCACCAAGGAATGGACGCCATGGCTGCTTGTATTGGATTGCAAGGGCGTTGGAATGCTTCACTTATTCAGCGCGACTTGATGATATCAGCAGGGGTCTTCACGGGAGGAATTCTTATGATGTGGTGTCTTTTTACAAAATGGATCAAAACAGAAGTGTCACACCAAGGGAAGAACAAGCGTAGTCGGCAAAAACTACGATTTAAAGAGGCTCGTGATAACAAATACGCATACGACGTAACAGGATCAAAGGATGCGATCGAAGAAATTTTTGGATCCGCATATACTAAGAAAGGTAAAGGTAAAGGGACAAAGGTTGGTTTGGGAGTCAAGCAACACAAATTCCACATGATGTATGGCTTTGATCCTCAAGAATACAACCTCATTCGTTTTGTCGATCCACTCACAGGAGCCACATTGGATGAACAGATCCATGCCGATATACGTTTAGTTCAAGAACATTTCGATGTTCTTCGGGAGGAAGCAGTGGCAAACGACACGATTGAGAGACAACATATATATAGCAGTCCCGGTTTGCAAGCATTTTTCATACAGAATGGATCAGCAAATGCATTAAGAGTTGATCTAACGCCGCACTCACCCTTACGTGTTGTCACAAACAACAATATAGCAGGTTTCCCAGAATACGAAGGCACTCTTCGTCAAACTGGAACTGCCATCACTGTACCTGTAAACCAAGTTCCAGTAGCCAATGAAACAGGAGTGGCACACGAATCTAAATCAATGATGATTGGACTAGGTGATTACACACCGATTTCACAGCAATTGTGCTTAGTACAAAATGATTCTGACGGGGTGAAAAGAAACGTATTCTCAATTGGTTATGGGTCGTACCTTATATCACCAGCGCATTTGTTCAAGTATAATAATGGTGAAATTACGATTAAGTCATCGAGGGGTTTGTATAAGATAAGAAATTCAGTAGAACTCAAATTACACCCTATTGCACACAGAGATATGGTTATAATTCAACTCCCTAAAGATTTCCCACCATTTCCAATGCGCCTTAAATTTTCAACACCAACACGGGAATCACGGGTGTGCTTGGTTGGAGTAAATTTTCAGCAAAATTACAGTACCTGCATTGTATCAGAGAGCAGCGTGACAGCACCAAAAGGAAATGGTGATTTTTGGAAACACTGGATCTCTACAGTGGATGGACAATGTGGTCTTCCATTAGTAGATGTTAAAAGCAAGCACATAGTCGGAATACATAGTCTTGCATCAACGAGTGGAAACACAAATTTCTTCGTCGCCATACCGGAGAACTTTAATGAGTACATCAGCAATCTTGTGCAAACAAACAAGTGGGAAAAAGGATGGCACTACAATCCAAATCTGATATCTTGGTGTGGTTTAAATTTAGTTGACTCAGCACCTAAAGGCTTATTCAAGACATCCAAACTAGTTGAAGACTTAGACACGAGTGTTGAAGAACAATGCAAGGTGACAGAAACATGGCTCACAGAATGTATCCAGGACAATCTACAAGTTGTGGCGAAATGCCCAGGTCAACTCGTAACTAAACACGTTGTCAAAGGTCCGTGTCCACACTTTCAATTATATTTATCAACACATGACGAAGCCAAAACATACTTTGCCCCCCTACTTGGAAAGTATGATAAGAGCAGATTGAACAGAGCAGCATTCATCAAGGATATATCAAAGTATGCGAGGCCGATTTACGTAGGTGAAATTAACTACGATATCTTTGAAAAGGCAATCGAGCGAGTGATCAAGATTCTTAAAAATGTCGGCATGCAGCAATGCGTTTATGTCACGGATGAAGAAGAAATTTTCAATTCACTCAATCTTAATGCAGCCGTCGGTGCCTTATACACAGGCAAGAAGAAGGATTATTTCAAGGATTATTCAAATGAGGATAAAGCTGAAATCATCATGCGATCCTGTGAGCGGATCTTCAATGGACAACTTGGCATCTGGAATGGGTCACTCAAAGCCGAAATACGCCCAATTGAGAAAACCATGTTGAATAAAACACGCACTTTCACAGCAGCACCATTGGAAACTCTACTTGGTGGAAAGGTTTGCGTGGACGATTTTAATAATCAGTTTTACTCTCATCACCTTGAAGGCCCATGGACAGTTGGAATCACAAAATTTTATGGAGGATGGAATCGCCTACTCGAGAAGTTACCAGAAGGCTGGATTTATTGCGACGCCGATGGTTCACAATTTGATAGCTCATTAACGCCATACCTTATCAACGCTGTTTTACATATTCGGCTGCAATTTATGGAAGAATGGGCACTAGGGGCACAAATGTTGCAAAATCTGTACACCGAAATCGTATACACACCAATTGCGACGCCAGACGGGTCAGTCATTAAGAAATTCAAAGGAAACAACAGTGGCCAGCCCTCTACAGTTGTTGATAACACACTCATGGTCATATTAGCATTCAATTACGCAATGTTGTCGAGTGGTATCAGAGAAGATGAAATAGATAACTGCTGCCGAATGTTCGCCAATGGAGACGATCTATTGTTGGCAGTGCACCCGGACTTTGAACATGTATTGGATGGATTCCAAAATCACTTTGGAAATTTGGGTCTCAATTTTGAGTTCACATCACGAACAAAAGACAAATCAGAACTGTGGTTCATGTCCACACGAGGCATCAAATGTGAAGGCATTTATATACCAAAACTTGAGAAAGAAAGGATAGTTGCCATACTCGAATGGGATCGATCGAACCTACCTGAGCACCGTCTCGAAGCTATATGCGCAGCCATGGTAGAAGCATGGGGATATCCAGACCTTGTTCAAGAAATACGAAAATTCTATGCGTGGCTTCTCGAAATGCAACCATTTGCGAATCTAGCGAAAGAGGGCTTAGCACCATATATAGCAGAAACCGCACTTAGAAATTTGTACTTGGGCACAGGAATCAAGGAAGAGGAGATTGAAAAATACTTTAAACAGTTTGCAAAAGATCTCCCTGGCTATATAGAAGATTATAATGAAGATGTTTTCCATCAATCCGGTTCTGTGGATGCAGGAGCTCAAGGAGGAACCAGTGGCTCAGGAACACCCACACCTGCAGCG---------------------------------------------------GGTAGTGGAAC----AGGAACTAGACCACCTTCAACTGGATCAGCAACACAAGGAAACACACCTCCAG---CATCAGGTGGCTCATCGGGAAATAATGGAGGT---AACCAATCAGGTTC---GAACGGCACTGG------------AAATCA---AGCAGGTTCCAACGGA------------ACAGGGGGCCAAAGAGACAAAGATGTTGATGCTGGTTCAACAGGAAAAATATCAGTGCCAAAGCTTAAGGCAATGTCAAAGAAAATGCGCCTGCCAAAAGCAAAAGGAAAAGATGTTCTACACTTAGATTTCCTTTTAACATATAAACCACAACAACAAGACATATCAAACACTAGAGCAACCAAGGAAGAGTTTGATAGATGGTATGACGCCATAAAGAAGGAGTATGAAATTGATGATACACAAATGACAGTTGTCATGAGTGGTCTCATGGTATGGTGCATTGAAAATGGTTGCTCACCAAACATAAACGGAAATTGGACGATGATGGACGGAGATGAACAAAGAGTGTTTCCATTGAAACCAGTTATTGAAAATGCATCTCCAACTTTCCGACAAATTATGCATCACTTCAGTGATGCAGCTGAAGCGTATATAGAATACCGAAACTCTACAGAGCGATATATGCCAAGATACGGACTTCAGCGAAATCTCACCGACTATAGCTTAGCACGGTATGCTTTTGATTTCTATGAAATGACTTCACGCACACCAGCTAGAGCTAAGGAAGCCCACATGCAG---ATGAAGGCCGCAGCAGTTCGTGGTTCCAACACACGCCTGTTCGGTCTGGACGGAAATGTCGGCGAGACTCAGGAGAATACAGAGAGACACACAGCTGGCGACGTTAGTCGCAACATGCACTCTCTGTTGGGAGTGCAGCATCACCACTAGTCTCCTGGAAACCCTGTTTGCAGTACCTATAGTATGTACTTTATAATTAATATGGTATGTATGTGAGGCTTTGCCTC------------------------GGTTTACTATTTAATTACGTATGTACTTTAAGTGTGAACCAGTCTGCAGGATACAGGGTTGGACCCAGTGTCTTCTGGTGTAGCACGTACTAGCGTCGAGCCACGTCACGGACAGCATTGGGGATGGCTTTGCCATGGGTGCTGCGAGTGTCTTGGTAAGAGACC---------------

>JX047431

---------------------AAAACAACAAAACTCAACACAACACAACAAAACACAACCAAACAAAACCAAGTTTTCTTTGCTCAGATTGTAGTGAACGGCTCGCAAGAAACGGTTCTTCGAGATCACTCTCTGATTTCTTTCTC----------TCTCGCACTTGCATTCAAGCGGAATGGCGGGATCGTGGACTCACGTGTCTTACAAGTGGCAACCAAATGTCAACAATGACCGTGATGTAAGAAAGGTAATGGAAATGTTTGCAGCAAAACATCAACATTACACAGAGGAGCAGCGACTTGCACATAACAGCAAGTTGCTAAGGAAGGCGTATGTTGTGGACGTTAAACCAGTGAAGCCAGCACCGGAGCCTATAAGGCGTAAAGTGTGGGTGGAAAAATTTGATCACAACCCAACCAAAGACCTGGTGTATCCGCGTCTT---GTCACGGTTAAAAAGGCAGCAGAAATGAAGCCCGTGAACACCTCTATAAATAAACTTATAAGGGATGTTCTGGACATATCAAAAGGGAGCAGCCTTAAACTTGAGCTGATTGGTAAACGCCAGAAGTGCAAAACACAACTAGCTATTAAGAAATACAACAATAAGGACTACCTCCATTGCAGGACACGTCATGAAGACAACATGTTCAAGAGGAAGGACGTGGCAATCGGCATTGAATGGATCCCAACCATTGAAGCGATTGCTCGATGCTACAGCACAGTAAACAAACAGGAGATGCAAAGTCTCTACAAAGGCAGTAGTGGCTTAACATTCATGCAAAATGATGAATTATTCATTGTTAGAGGCAGAATGAATGGCGAGTTAGTTAATAGCCTGGAAGAGAACAGGAACGTGCTCGACATCGATCACTACGCTGATCCACAAGCGAACGACTTTTGGAAAGGGTACACTGATGCATACGTTGCGAATCGCAGTATCTCCACTACACATACCGTGCATACTCCAACAATCAACTTGGTAGAGTGTGGAAAGAGAATGGCATTACTTGAAATTTTATTCCATTCAACTTTTAAGATTACATGTAAGACATGCAATATTGATGACTTGGAGTTATCAGACGATGAATTTGGTGCAAAACTTTTCAAGAATCTGCAACGTATCGAGGAACAGCAACGCGAATATCTCGCTAAAGATCAAAAGCTGCGTCGCATGATTCAATTCATCAAAGAAAGGTGTAATCCAAAGTTCTCTCATCTACCATTGCTTTGGCAAGTTGCAGAAACAATAGGACATTATACGGACAACCAGTCAAAGCAAATAATTGATATCAGTGAAGCACTCATCAAAGTTAACACATTAACACCTGATGATGCAGTCAAGGCGAGTGTAGCACTGCTTGAGGTGGCACGATGGTATAAAAATAGGAAAGAATCACTTAAAACAGACACATTAGATTCGTTTCGAAACAAGATATCCCCAAAGAGCACGATAAACACAGCATTGATGTGTGACAATCAACTAGACAAGAATGCAAATTTTGTCTGGGGAAACAGGGAATATCATGCAAAGCGCTTCTTCTCAAATTACTTTGAAGCAGTAGATCCTACTGACGCATACGAGAAGCACGTAACCCGTTTCAATCCAAATGGTCAACGGAAATTATCAATTGGGAAACTAGTAATTCCGTTAGATTTCCAGAAAATAAGAGACTCATTCGTCGGTTTGGCAATAAACAAACAGCCACTTACAAAGGCTTGTGTGAGCAAAATCGATGGTGGTTATGTATACCCATGCTGTTGTGTCACAACAGAATTTGGAAAACCAGCATACTCTGAGATAATACCACCAACAAAGGGCCATATAACAATTGGAAATTCTGTAGACCCAAAGATCGTAGACCTGCCAAATACAACACCACCAAGTATGTACATTGCCAAAGATGGATATTGTTACATCAACATTTTCTTAGCAGCAATGATAAATGTCAATGAAGACTCAGCAAAGGACTACACAAAATTCCTCAGGGATGAATTAGTCGAGCGACTTGGAAAGTGGCCGAAACTTAAAGATGTTGCAACAGCGTGCTATGCGTTATCAGTTATGTTTCCAGAGATTAAGAACGCTGAATTGCCTCCAATACTCGTTGACCACGAAAGCAAGTCAATGCACGTGATCGATTCATACGGCTCATTGAGCGTTGGGTTCCACATCCTTAAAGCAAGTACTGTTGGCCAGCTCATAAAATTTCAATACGAATCAATGGATAGTGAGATGCGCGAATACATAGTGGGAGGTACTTTAACGACCCAAACATTTAACAAACTCCTCACATCTTTAGCTAAAAATATGTTCAAACCAGATCAAATTAAGCAGATGATTGAGGAGGAACCCTTCCTACTAATGATGGCAATCGCGTCACCAACTATGCTCATAGCGCTATACAATAATTGCTATATAGAGCAGGCTATGACGTATTGGATCGTTAAAAACCAAGGAGTGGCAGCGATATTCGCACAACTGGAAGCACTAGCAAAGAAGACCTCACAAGCTGAATTGCTAGTCCAACAAATGCAAATACTTGAAAAGGCGTCTAATCAATTGAGGCTTGCAGTCACGGGTTTAAATCATGTCGATCCAGCCAAGCGTCTGCTATGGTCGCATCTAGAAGCAATGACAACACGATCAGAGATGAACAAGGAATTAATTGCTGAAGGCTATGCCCTGTATGATGAGCGCCTGTATGCACTTATGGAAAAAAGTTACGTAGATCAATTAAACCAATCATGGGCAGAGTTATCATTCTGTGGAAAATTTTCAGCAATATGGCGTGTGTTCAAAGTCAAGAAATATTACAAGCCGTCTTTAACCGTGAGAAAAAGCGTAGATTTAGGCGCTGTGTACAATATATCAGCTACGCATCTAATATCAGATTTAGCGCAGAGAAGTCGCGATCGAGCCAGCTCTATTTTAACCAAACTCCGCAACGGTTTTTATGATAAGTTAGAGAAGACTAGAACTCGAGTAATTAAAACTGTTTATTGGTTCATACCTGACATATTTAGACTTATGCATATTTTCATAGTTCTTAGTTTATTAACAACTGTGGCAAACACTATTATTGTGACTATGAATGATTACAGAAAGCTTAAAAAGCAACAAAGAGAGGATGAGTATGAAGCTGAAATCAACGAGGTTCGAAAAATTCACGCTAATCTAATGAAAGAGCATAATGACAATCTAACATGTGAACAGTTTATCGAACATATGCGCCAGACGCATCCACGATTAATTGAAGCCACATTGGAGTTGACACACACAGGCGTCATTCATGAAGGAAAGAGCAACCTAGAAACCAATCTCGAGCAAGCTATGGCAGTGGGCACCTTACTCACAATGATGTTAGACCCACAGAAGAGTGATGCAGTATACAAAGTGTTGAATAAGATGAGAACTGTAATTAGCACATTTGAGCAGAATGTCCCATTTCCTTCAATCAACTTTACAAATATTTTAACACCATCAGTGGCGCAACAAAGTGTAGATGTTGATGAACCTCTAACTTTAAGTACTGATAAGAATCTAACAATAGATTTTGATACTAATCAAGATTTACCAGCGGACACGTTTAGCAATGATGTTACTTTCGAAGATTGGTGGGCAAATCAAATAAGCAACAATAGAACAGTTCCACATTATCGGCTTGGTGGCAAATTTGTAGAATTCACAAGAGAAAATGCAGCACATGTTAGCATTGAGCTTGCACACTCAAACCTTGAGAAGGAGTTTCTACTCAGAGGAGCTGTTGGCTCAGGAAAATCCACTGGTCTTCCATATCATCTTAGTATGCGTGGTAAGGTGCTTTTAATAGAACCAACAAGACCACTAGCCGAGAATGTCTGCAGACAGCTTCAAGGACCACCATTCAATGTCAGCCCGACACTACAAATGCGTGGTTTAAGCTCTTTTGGTTGTACGCCAATTACGATAATGACATCAGGTTTCGCACTGCACATGTATGCAAATAATCCGGATAAGATCTCTGACTACGATTTTATAATATTTGATGAGTGTCATATCATGGAAGCGCCAGCTATGGCGTTCTACTGTTTGCTGAAAGAATATGAATACAGAGGCAAGATCATTAAGGTGTCCGCAACACCACCAGGACGAGAGTGTGAGTTCTCTACTCAGCATCCAGTTGATATCCATGTATGCGAAAATCTCACTCAGCAACAATTCGTTAGAGAGCTTGGTTCCGGATCAAACGTTGACGCAACAAAGTATGGAAACAACATACTTGTATATGTCGCAAGCTACAACGACGTCGACTCATTAGCGCATGCTTTAACTGAATTACATTATTCAGTCATAAAGGTTGATGGGAGAACAATGAAACAGAACACCACAGGGATAGTGACAAATGGCACCTCTCAAAAGAAGTGTTTTGTCGTTGCTACGAATATTATTGAGAATGGTGTCACATTGGATGTTGATGTTGTGGTCGACTTCGGACTCAAAGTAACAGCTGAATTAGATGTTGACAACAGAGCTATTCTATACAAGCGAGTTAGCATTTCATATGGTGAGCGAATTCAAAGGCTAGGGCGTGTTGGAAGAAATAAACCCGGAACAGTCGTTCGAATTGGAAAAACCATGAAGGGTTTGCAAGAAATCCCAGCCATGATAGCGACAGAAGCGGCATTCATGTGCTTTGCATATGGACTAAAGGTCATAACACACAATGTATCAACAACACACTTAGCAAAGTGCACAGTCAAGCAAGCGCGAACAATGATGCAATTTGAACTTTCACCATTTGTGATGGCAGAACTAGTCAAATTTGATGGCTCAATGCACCCACAAATCCACGAAGCACTTGTGAAATATAAGCTTAGAGATTCTGTAATAATGCTCAGACCTAACGCAATTCCCAAAGTTAATTTTCACAACTGGCTAACAGCACGTGATTACAACAGGATGGGTTGTTCATTAGAGCTAGAGGATCATGTCAAAATTCCATATTACATACGAGGTGTCCCTGACAAGTTATATGGAAAGTTATATGATATCATTTTACAATATAGTCCAACCAGTTGTTATGGGAGACTATCAAGTGCTTGTGCTGGGAAGGTAGCATACACCTTACGAACCGACCCTTGTTCGCTACCACGAACAATAGCAATAATTAATGCATTAATCACAGAGGAGCATGCTAAGAGAGATCATTATCGTAATATGATAGCAAATCCTTCGTCTTCGCATGCATTCTCACTAAATGGATTAGTCTCCATGATTGCTTCAAGATATATGAAAGACCACACAAAAGAGAATATAGACAAACTTATTAAGGTGCGTGATCAACTACTCGAATTTCAAGGCACGGGTATGCAATTTCAAGATCCATCAGAACTAATGGACATTGGCGCCTTGAACACAGTTATACATCAAGGAATGGATGCAACAGCTGCATGCATTGGTCTCCAAGGGCGATGGAACGCATCACTCATACAACGTGATCTCATGATTGCAGGAGGAGTATTCATTGGAGGAATATTAATGATGTGGAGCTTGTTCACTAAGTGGGGAACAACCAATGTATCACACCAAGGAAAGAACAAGCGTAGCCGACAAAAATTGAAGTTCAAGCAAGCTAGAGACACTAAATACGCATATGATGTGACAGGATCGGAAGAAACTCTTGGTGAAAACTTTGGAACAGCTTATACAAAGAAGGGCAAAGGGAAAGGTACCAAAGTTGGTCTTGGGGTGAAGCAGCATAAATTTCACATGATGTATGGTTTTGATCCTCAAGAGTACAACCTGATTCGCTTTGTTGACCCTCTTACAGGTGCAACATTAGATGAACAAATCCATGCTGATATTCGCTTAGTGCAAGAGCACTTTGACATAATCCGAGAAGAGGCAGTCGCAAACGACACAATTGAGCGGCAACACATATACGGAAATCCTGGTCTCCAAGCATTCTTTATACAGAATGGATCAGCTAATGCATTAAGAGTTGATTTAACACCACATTCACCTTTGCGTGTTGTAACGAATAACAACATAGCAGGATTTCCAGAATATGAGGGCACATTACGACAAACTGGTACAGCTCTCACTGTACCTGTGAATCAAGTACCAGTAGCAAATGAGACAGGAGTTGCCCACGAATCCAAATCTATGATGTCTGGATTAGGCGATTACACACCTATTTCTCAGCAGCTCTGTCTCGTACAAAATGATTCTGAAGGAGTCAAACGTAATGTGTATGCAATTGGGTATGGATCATATTTAATATCACCGGCGCATCTCTTCAAGTATAACAATGGTGAAATTACAATTAAATCCTCAAGAGGGCTGTATAAAATTAGAAATTCAGTCGATGTCAAATTGCACCCGATTGCACAGAGGGATATGGTCATAATTCAACTTCCAAAAGACTTCCCACCGTTCCCAATGCGACTTAAGTTCTCAACTCCGTCAAGAGATGTGCGTGTGTGCTTAGTTGGAATCAATTTTCAACAGAATCATACCACGTGCATAATATCCGAAAGCAGTGTGACAGCACCCAAAGGAAATGGTGATTTCTGGAAACATTGGATTTCAACTGTTGATGGGCAATGTGGGCTACCGTTAGTTGACGTTAAGAATAAACACATTGTCGGAATTCACAGCCTGGCTTCAACGAGTGGAAATACGAACTTTTTCGTTGCAATGCCTGAGAACTTCAATGAGTATATATCTAATCTCGTGCAAACAAATAAGTGGGAAAAGGGATGGCATTACAACCCAAATCTTATTTCATGGTGTGGTCTAAACCTAGTTGATTCAGCACCTAAAGGATTGTTCAAAACATCAAAACTTGTTGAAGATTTGGATATGAGCGTTGAAGAACAATGCAAGGTGACAGGGACATGGTTGACGGAACACATCCAGGATAATCTACAGGTCGTTGCAAAGTGTCCAGGCCAACTTGTAACAAAGCATGTCGTTAAAGGCCCATGTCCGCATTTTCAACTGTATTTATCCACACATGATGAGGCAAAGTTGTACTTTTCACCTTTGCTTGGAAAGTATGACAAGAGTAGGTTGAACAGGGCAGCATTTATCAAAGATCTCTCAAAGTACGCAAAGCCGATTTATATTGGAGAGATCAATTATGAAATCTTTGATAAGGCAGTTGAACGAGTTATAAGCATCCTCAGAAGTGTAGGAATGCTACAGTGTACATACGTGACGGACGAGGAAGAAATTTTCAATTCGTTAAACATGAACGCAGCCGTAGGTGCACTCTACACAGGAAAGAAGAAAGACTATTTCAAAGATTTCTCGAACGATGACAAAGCTGAAATCGTTATGCGTTCATGTGAGCGCATCTACAATGGACAATTGGGTGTATGGAACGGCTCACTCAAAGCTGAAATACGACCAATAGAGAAAACCATACTAAACAAGACACGCACTTTCACAGCAGCGCCATTAGAAACTCTACTTGGTGGGAAAGTATGTGTGGACGATTTTAACAACCAATTCTATTCACACCATCTTGAAGGCCCATGGACCGTAGGAATCACAAAGTTTTATGGAGGATGGAACCGACTTTTGGAGAAATTGCCAGAAGGATGGATTTATTGCGATGCAGACGGATCCCAGTTTGACAGCTCATTAACTCCATATCTTATTAATGCTGTATTGCACATTCGCTTACATTTCATGGAAGAATGGGAATTGGGAGCTCAAATGTTGCGAAACTTATACACAGAGATTGTTTACACGCCAATCGCAACACCTGATGGATCTGTCATCAAGAAGTTTAAAGGAAACAATAGTGGGCAACCATCTACAGTCGTTGACAACACGCTCATGGTTATTATAGCATTTAACTATGCGATGTTATCAAGCGGCATTCCTGAAGACAAAATTGACGACTGCTGTAGAATGTTTGCAAACGGCGACGACTTACTCTTGGCAGTGCATCCGGATTACGAATACATATTGGACGGATTTCAAAATCATTTCGGAAACCTTGGCCTCAATTTCGAGTTCACATCAAGGACAAAGGACAAATCAGAGTTATGGTTCATGTCAACACAAGGAGTCAAGTGTGAAGGTATCTACATACCAAAACTCGAAAGGGAAAGAATAGTCGCAATCCTTGAATGGGACCGATCGAACTTGCCTGAGCATCGTCTTGAAGCTATTTGTGCAGCAATGGTTGAGGCATGGGGTTATCCAGACTTGGTGCAAGAAATACGGAAATTTTATGCATGGTTGCTTGATATGCAACCATTTGCAAATTTAGCAAAGGAAGGCATGGCACCATACATAGCAGAAACAGCACTCCGCAACCTTTACCTTGGAACAGGCATCAAAGAAGAAGAAATTGAAAAATATTTTAAGCAGTTTATCAAGGATCTTCCTGGATACGTAGAAGATTACAATGAAGAAGTTATTCATCAATCTGGCCAAGTTGATGCAGGGAGACAAGGTGGTAGCGGCACTCAAGGAAACACACCACCAGCAGGG---------------------------------------------------AGTGGAGGCAC----TGGATCTGGCACTCAGGGCAATGGGGGTCAGACGGGG---------TCCCAAG---GAGGTGGTGGTCAACAGGGGTCCGGTGGGAGCACTGGTCAAGGAGCAGCTGGAAGCAACGGCGG------------AGGCCAGACAGGAGGCTCTAGTGGG------------ACAGCTGGTCAAAGAGATAAGGACGTTGACGCAGGCTCGGCTGGAAAGATATCCGTACCAAAGCTTAAAGCCATGTCAAAGAAAATGCGCTTGCCAAAGGCAAAAGGAAAAGATGTCTTGCATCTGGACTTTCTGTTAACATACAAACCACAACAGCAAGACATATCGAACACAAGAGCAACCAAGGAAGAGTTCGATAGATGGTATGATGCCATAAAGAGGGAATATGAGATCGATGATACACAGATGACAGTCGTCATGAGCGGTCTCATGGTATGGTGCATCGAAAACGGTTGCTCACCAAACATAAACGGAAATTGGACGATGATGGATGGGGATGAACAAAGAGTTTTCCCACTAAAACCAGTTATTGAAAACGCATCTCCAACTTTTCGACAAGTTATGCATCATTTCAGTGATGCAGCTGAAGCGTATATAGAATACAGAAATTCTACTGAGCGATACATGCCAAGATACGGACTTCAGCGAAATCTCACCGACTATAGCTTAGCGCGGTATGCTTTTGATTTCTATGAAATGACTTCACGCACACCAGCTAGAGCTAAGGAAGCCCACATGCAG---ATGAAAGCCGCAGCAGTTCGTGGTTCAAACACACGACTGTTCGGCTTGGACGGAAATGTCGGCGAGACTCAGGAGAATACAGAGAGACACACAGCTGGCGATGTTAGTCGCAATATGCACTCTCTGTTGGGAGTGCAGCAACACCACTAGTCTCCTGGAAACCTTGTTTGCAGTACCTATAATATATACTA------ATATATAGTACGTTGGTGAGGCTTTGCCTC-----------------------GGTTTTACTATTTTATTACGTATGTATTTGCAGCGTGAACCAGTCTGCAGCATGCAGGGTTGGACCCAGCGTGTTCTGGTGTAGCGTGTACTAGCGTCGAGCCATGAGACGGACTGCACTGGGTGTGGCTATGCCACTTGTGTTGCGAGTCTCCTGGTAAGAGAC----------------

>JX047427

---------------------AAAACAACAAAACTCAACACAACACAACAAAACACAACCAAGCAAAATCAAGTTTCCTTTGCTCAGATTGTAGTGAACGGCTCGGTAGGAAAGGTTCCTCGAGATCACTCTCTGATTCTTCTTTC----------TCAACCAATTTCATTCAAGCGAGATGGCGGGCTCTTGGACTCACGTGACATACAAGTGGCAACCAGATGTCAACAACGCACGTGATGTGAATAGAGTGATGGAGATGTTTGCAGCAAAACATCAACGTTACACTGAGGAGCAAAGGCTTGCTCACAACAGCAAGCTATTAAGGAAGGCTTGTGTCACTAGTGCTGAGTTTATTGAACCAGCACAGAAACCAAAATGTCGCCAGACATGGGTTGAAAAGTGCGACCACAACCCCACAGAGCACTTTGTTTATCAACGCTTC---ACACCTGAGAAGAGAGTGCTTAACACCAAACCTGAGACAACCTCTGTTACGAAATTAATCAGGGATGTCCTTGAGATTTCGAAGGGCAGTGGGATTAAAATTGAGTTGATCGATAAGCGTATTAAACGTAAGACTCAATTATCCATAAGGCAATACAATGGCAAAAACTTCTTGCACTGCAAAACCAGGCATGAAAATGGCCTGTTTAAACGCAAGGACATTGACATTAATGTCAAGTGGTTGCCCACCATTGAAGCCATTGCAAAATGCTACAGCACGGTGAATGCGGAAGAACTGCAAAGTCTCACTAAAGGCAGTAGTGGTCTTACATTCATGCAAAATGATGAATTGTTCATCGTGCGTGGAAGGATGCATGGTGAGATTGTCAATAGTTTACACGAAAATAAGCACGTTATGGAAATTGAACACTATGCTGACCCACAAGCAAACAGTTTCTGGAAAGGTTACACAGACGCGTATGTCGAAAACAGAAACATATCTACCACTCATACAGAGCACACACCAACTATTAATTTAGAGGAGTGTGGTAAGAGAATGGCACTGTTAGAAATTCTATTCCACTCAACTTTTAAGATCACATGCAAAACGTGCAATATTGATGATCTGGAATTATCAGATGATGAATTTGGGGCCAAGTTATATAGCAATCTACAGCGTATTGAAGAAAAGCAACGTGAATATCTCGCTAAAGATCAAAAACTTCTACGCATGATACACTTTGTAAAGGATCGGTGTAACCCAAAATTTTCACATTTGCCTTTACTATGGCAAGTGGCGGAAACAGTAGGGCATTACACCGACAACCAATCAAAGCAGATAATTGACATCAGTGAGGCGCTTACCAAAGTTAATACTTTAACTCCTGATGATGCAGTGAAGGCCAGTGTAGCATTACTAGAAGTAGCACGGTGGTATAAAAATCGAAAGGAATCACTTAAAACGGACACACTGGATTCATTTCGAAACAAGATTTCTCCAAAGAGCACGATCAACGCAGCATTAATGTGTGATAACCAGTTAGATAAGAATGCAAATTTCGTATGGGGAAACAGAGAATACCATGCAAAGCGATTCTTCGCTAATTATTTTGAAGCCGTGGACCCAACTGATGCATATGAAAAACACGTAACACGCTTCAACCCCAATGGACAGCGGAAATTATCGATTGGCAAACTAGTAATTCCACTAGATTTCCAGAAGATCAGAGATTCGTTCGTTGGCCTATCAATAAATAAACAACCACTGAGCAAAGCTTGCGTAAGCAAAATTGATGGAGGCTACGTATATCCATGTTGCTGCGTTACAACGGAGTTTGGAAAACCAGCATATTCTGAGATAATACCTCCAACGAAAGGACATATCACGATTGGAAACTCAGTGGACCCAAAAATAGTGGATTTACCGAATACTACACCACCAAGTATGTACATTGCAAAAGATGGATACTGTTACATTAACATATTCTTGGCAGCAATGATAAACGTCAATGAGGAATCCGCAAAGGATTACACTAAGTTTCTTAGAGACGAGTTGGTGGAACGGCTTGGTAAATGGCCAAAATTGAAAGATGTAGCTACAGCATGTTATGCGCTATCTGTGATGTTTCCTGAAATCAAGAACGCTGAGTTACCACCGATATTAGTTGACCATGAGAGCAAATCGATGCACGTAATAGATTCGTATGGTTCGCTGAGTGTGGGGTTTCACATACTCAAAGCAAGCACCATTGGACAGTTGATAAAATTCCAATATGAATCGATGGAAAGCGAAATGCGGGAATACATAGTAGGAGGCACTTTAACACAACAAACTTTCAACACTCTACTTAAAACTCTCACAAAGAACATGTTCAAACCTGAGAAAATTAAGCAGATAATTGAGGAAGAGCCTTTCTTACTGATGATGGCAATCGCATCACCAACTGTGCTCATATCTTTATACAATAATTGTTACATAGAGCAAGCTATGACATACTGGATTGTTAAAAATCAAGGTGTTGCAGCAATATTCGCACAGCTCGAAGCATTAGCCAAAAAGACGTCTCAAGCTGAATTATTAGTTTTACAGATGCAAATACTTGAAAAGGCGTCTAATCAGCTGAGGCTTGCAGTGACAGGACTAAACCATGTTGACCCCGCTAAGCGTCTTTTATGGTCACATCTTGAAGCTATGACAACGCGATCGGAGATGAACAAGGAACTGATAGCAGAAGGCTATGCTTTATATGACGAGCGCTTGTACACTTTGATGGAAAAAAGTTACGTAGATCAATTAAACCAGTCATGGGCAGAATTATCATACTGTGGAAAATTTTCAGCAATATGGCGTGTGTTCAAAGTCAGGAAATATTACAAGCCGTCTTTAACCGTGAGAAAAAGCGTAGATTTAGGCGCTGTATACAATATATCAGCTACGCATCTAATATCAGGTTTAGTGCAGAAAAGTCGCGATCAAGTCAGCTCTACTTTAACCAAACTCCGCAACGGTTTTTATGATAAATTAGAGAAGACTAGAGTGCGTGCAATCAAAACAGTATATTGGTTTATACCCGATATATTTAGACTTGTTCACATTTTTATAGTGCTAAGTTTGTTAACCACCATAGCTAACACGATCGTAATAACTATGAATGACTATAAAAAGTTGAAAAAGCAACAAAGAGAGGATGCGTATGAGGCTGAGATAAACGAAGTTCGCAAGATTCATGCCGCCTTGATGAAGGAGCATAACGACAATTTAACATGCGAACAGTTCATTGAACATATGCGTCAAACACATCCACGATTAGTTGAAGCCACATTGGAGTTGACCCACACAGGTGTGATTCATGAAGGAAAGTCTAATCTAGAAACCAACCTTGAGCAAGCTATGGCTGTGGGAACACTACTCACGATGATACTTGATCCTCAGAAGAGTGACGCTGTGTACGAAGTTCTAAACAAAATGCGTACAGTGATTAGTACATTTGAACAGAATGTCTCATTCCCTTCAATTAACTTCACTAACATCTTGACACCACCCGTAACACAGCAGAGTGTGGACGTCGATGAGCCTCTTACTTTGAGCACTGACAAGAACTTAACGATAGATTTTGATACAAATCAAGATTTACCTGCAGACACTTTCAGTAACGATGTTACATTCGAGGATTGGTGGGCAAATCAGATAAGCAATAATAGAACAGTTCCACACTATCGTCTCGGTGGAAAATTTGTTGAATTTACAAGGGAAAACGCAGCACTTGTTAGTATTGAATTAGCTCACTCGAACATTGAAAAGGAATTTCTTTTAAGGGGTGCGGTTGGCTCAGGAAAGTCCACAGGGCTGCCATACCATCTTAGTGCGCGCGGAAAAGTACTTCTAATCGAACCAACAAGACCACTTGCCGAGAATGTATGCAGACAGCTACAAGGACCACCATTCAATGTTAGTCCGACACTTCAAATGCGTGGGTTGAGCTCTTTTGGCTGCACGCCAATCACGATCATGACCTCTGGTTTTGCATTACATATGTACGCAAACAATCCGGACAAGATATCTGATTACGACTTCATCATATTTGATGAATGTCATATCATGGAAGCACCAGCCATGGCATTTTATTGTTTGCTGAAGGAATATGAATATCGAGGCAAAATCATTAAAGTCTCCGCAACACCACCTGGAAGAGAATGTGAATTTACCACACAACATCCAGTTGACATTCACGTCTGCGAGAACCTCACACAACAGCAATTTGTAAGGGAACTTGGCACGGGTTCAAATATCGACGCAACGAAATATGGAAATAACATACTTGTTTACGTTGCTAGCTACAACGACGTCGATTCGCTATCACACGCGCTAACTGAATTACATTATTCAGTTATCAAAGTTGACGGTAGAACTATGAAGCAGAACACCACAGGCATTGTAACTAATGGCACATCACAAAAGAAGTGTTTTGTCGTTGCAACGAACATAATCGAGAATGGTATTACATTGGATGTTGATGTCGTAGTGGACTTTGGACTAAAAGTCACAGCTGAGCTGGATGTTGACAATAGAGCAATTCTATACAAACGTGTCAGCATCTCGTATGGTGAGCGTATTCAAAGACTCGGTCGTGTTGGAAGGAATAAACCCGGAACAGTGGTCCGCATTGGGAAAACTATGAAAGGTTTGCAGGAAATCCCAGCGATGATAGCAACGGAAGCTGCTTTTATGTGTTTTGCATACGGACTCAAAGTTATAACACACAATGTATCTACAACACTCTTAGCTAAATGCACGGTAAAACAAGCAAGAACAATGATGCAATTTGAACTATCACCATTTGTAATGGCAGAATTAGTTAAATTCGATGGTTCAATGCATCCACAAATTCATGAAGCACTCGTAAAATACAAACTTAGAGATTCTGTGATAATGCTAAGACCAAATGCTATCCCGAAGGTCAACTTTCACAACTGGCTGACTGCACGTGATTATAACAGGATGGGCTGTACAGTAGAACTTGAAGATCATGTGAAAATACCGTACTATATACGAGGGATTCCTGATAAACTATATGGAAAATTGTACGATATCATTTTACAATACAGCCCAACTAGTTGTTATGGAAGACTATCAAGTGCCTGTGCGGGAAAAGTAGCATATACCCTGCGCACTGATCCTTGTTCGTTACCACGCACGATAGCTATTATTAATGCCTTAATTACTGAAGAGTATGCAAAGAGGGACCATTACAGAAATATGATAGCAAACCCCTCATCATCGCACGCCTTTTCACTCAATGGGCTGGTATCCATGATCGCTTCTCGGTATATGAAAGACCACACGAAGGAAAACATTGATAAGCTTGTAAGAGTGCGCGACCAACTACTTGAGTTCCAAGGCACAGGTATGCAATTTCAAGATCCTTCAGAATTGATGGACATTGGTGCATTAAACACAGTTATTCACCAAGGAATGGACGCCACGGCTGCTTGTATTGGATTACAAGGGCGCTGGAATGCTTCGCTCATTCAACGCGACTTGATGATATCAGCAGGGGTCTTCACAGGAGGAATTCTCATGATGTGGTATCTTTTCACAAAATGGAGCAAGACAGAAGTGTCACACCAAGGAAAGAACAAACGCAGCCGGCAAAAACTACGATTCAAAGAGGCACGTGATAATAAATATGCCTATGACGTAACAGGATCAGAGGAAGTTCTTGGTGAGAATTTTGGAGCCGCATACACTAAGAAAGGAAAAGGAAAGGGGACAAAAGTTGGCCTTGGAGTCAAACAGCACAAATTCCACATGATGTACGGGTTTGACCCACAAGAATATAACTTAATCCGCTTCGTGGATCCTTTAACAGGCGCGACACTAGACGAGCAAATTCATGCAGATATAAGTTTAGTGCAAGAGCACTTTAGCGTCATTAGAGATGAAGCAGTGGCAAATGACACAATTGAAAGACAACACATTTACAGCAATCCTGGACTACAAGCGTTCTTCATACAAAATGGATCTGCAAATGCACTGAGAGTTGATTTAACACCACATACACCACTGCGTGTCGTAACCAATAACAATATAGCAGGTTTCCCAGAATATGAAGGTACACTTCGACAAACAGGGACAGCTTTACAGATACCCGTGAATCAAGTTCCAGCTGCGAATGAAGCGGGGGTAGCACATGAGTCAAAATCGATGATGGCAGGGTTAGGCGACTACACCCCAATATCACAGCAGTTGTGTTTAGTCCAGAATGACTCAGATGGAATCAAAAGGAATGTGTACTCAATTGGATATGGATCATATCTCATTTCACCGGCACACTTATTTAAATATAACAATGGCGAAATAACAATTAAATCTTCAAGAGGTTTGTATAAGATCAGAAATTCAGTAGAACTCAAGTTGCATCCTATTGCACATAGAGATATGGTCATTATTCAACTTCCGAAAGATTTTCCGCCGTTCCCAATGCGTCTCAAGTTTTCTAAACCATCTAGAGAGTCAAGAGTGTGCTTAGTTGGAGTGAACTTCCAACAAAACTACAGCACATGCATTGCATCGGAGAGTAGTGTCACAGCACCAAAAGGCAATGGAGATTTCTGGAAACACTGGATATCCACAGTGGACGGACAATGCGGCCTCCCATTAGTAGATGTCAAGAGCAAGCACATAGTTGGAATACACAGCCTTGCATCAACTAGTGGAAACACTAACTTTTTCGTCGCCATGCCTGAAGATTTCAATGACTACATTCACAATCTTGTGCAAACCAACAAGTGGGAAAAAGGATGGCATTACAACCCAAATCTCATTTCATGGTGTGGTCTCAACCTAGTTGATTCAGCTCCAAAGGGTCTCTTCAAAACTTCGAAATTAGTGGAAGACCTTGACATGAGTGTTGAAGAGCAGTGCAAAGTTACAGAGACATGGCTCACAGAATGCATTCAGGACAATTTACAGGTTGTCGCAAAATGCCCAGGCCAACTTGTCACCAAGCACGTTGTCAAAGGCCCATGCCCACACTTTCAGCTATATCTGTCAACACATGATGAAGCCAAAGCATACTTTGCACCACTACTCGGAAAGTACGATAAGAGCAGATTGAACAGAGCAGCTTTTATCAAAGACATTTCAAAATATGCAAAACCAATCTACATTGGAGAAATCAATTATGATGTTTTTGAAAAGGCTATAGAACGTGTGATTAAAATCCTTAGAGATGTGGGAATGCAACAATGCGCGTATGTCACGGATGAGGATGAAATATTCCAGTCACTCAACCTCAACGCCGCAGTTGGTGCCTTATACACAGGAAAGAAGAAAGACTATTTCAAGGATTTCTCAAATGAAGACAAAGCCGAAATCATCATGCGATCATGTGAGCGCATCTATAATGGACAATTGGGTGTATGGAACGGTTCACTCAAAGCTGAAATACGACCAATAGAGAAAACCATACTAAACAAGACACGCACTTTCACAGCAGCGCCATTAGAAACTCTACTTGGTGGGAAAGTATGTGTGGACGATTTTAACAATCAATTCTATTCACACCATCTTGAAGGCCCGTGGACCGTAGGAATCACAAAGTTTTACGGAGGATGGAACCGACTTTTGGAGAAATTGCCAGAAGGATGGATTTATTGCGATGCAGATGGATCCCAGTTTGACAGCTCACTAACTCCATATCTTATTAATGCTGTGCTGCATATTCGTTTACATTTCATGGAAGAATGGGAGTTGGGAGCCCAAATGTTGCGAAATTTATACACAGAGATTGTTTACACGCCAATCGCAACACCTGATGGATCTGTCATCAAGAAGTTTAAAGGAAATAATAGTGGGCAACCATCTACAGTCGATGACAACACGCTCATGGTTATTATAGCATTTAATTATGCAATGTTATCAAGTGGCATTCCTGAAGACAAAATTGACGACTGCTGTAGAATGTTTGCAAACGGTGACGACTTACTTTTGGCAGTACATCCGGATTACGAATATATATTGGACGGATTTCAAAATCACTTTGGAAACCTCGGCCTTAATTTCGAGTTCACATCGAGGACAAAGGACAAGTCAGAGTTATGGTTCATGTCAACACAAGGAGTCAAGTGTGAAGGTATCTACATACCAAAACTCGAAAGGGAAAGAATAGTCGCAATCCTTGAATGGGACCGATCGAACTTGCCTGAGCATCGTCTTGAAGCTATCTGTGCAGCCATGGTTGAAGCATGGGGTTACTCAGATTTAGTTCATGAAATTCGAAAGTTTTACGCGTGGCTTCTCGAAATGCAACCCTTCGCGAACCTGGCAAAGGAAGGCATGGCGCCATACATAGCAGAAACAGCACTCCGCAACCTTTACCTTGGAACAGGCATCAAAGAAGAAGAAATTGAAAAATATTTTAAGCAGTTTGTCAAGGATCTTCCTGGATACGTAGAAGATTACAATGAAGAAGTTATTCATCAATCTGGCCATGTTGATGCAGGGAGACAAGGTGGTAGCGGTGCTCAAGGGGGCACACCACCAGCAGGA---------------------------------------------------AGTGGAGGCAC----TGGATCTGGCACTCAGGGCAATGGGGGTCAGACGGGA---------TCCCAAG---GAAGTGGTGGTCAACAAGGGTCCGGTGGGGGCACTGGCCAAGGAGCAGCTGGAAGCAACGGCGG------------AGGCCAGACAGGAGGCTCTAGCGGG------------ACAGCTGGTCAAAGAGATAAGGATGTTGACGCAGGCTCGGCTGGAAAGATATCCGTACCAAAGCTTAAAGCCATGTCAAAGAAAATGCGCTTGCCAAAGGCAAAAGGAAAAGATGTCTTGCATCTGGACTTTTTGTTGACATACAAGCCACAACAGCAAGACATATCGAACACAAGAGCAACTAAGGAAGAGTTCGATAGATGGTACGACGCCATAAAGAAGGAGTACGAGATCGATGATACACAAATGACAGTTGTCATGAGTGGTCTGATGGTCTGGTGCATCGAGAATGGTTGCTCACCAAATATAAACGGGAATTGGACGATGATGGATGGAGAAGAACAACGAGTTTTTCCATTAAAACCAGTCATTGAAAACGCATCTCCAACCTTCCGACAAATAATGCACCACTTTAGTGATGCAGCTGAAGCGTACATAGAGTATAGAAACTCTACAGAGCGATATATGCCAAGATACGGACTTCAGCGAAATCTCACCGACTATAGTCTAGCACGGTATGCATTTGATTTCTATGAAATGACCTCACGCACACCAGCTAGAGCTAAGGAAGCCCACATGCAG---ATGAAAGCCGCAGCAGTTCGTGGTTCAAACACACGACTGTTCGGTCTGGACGGAAATGTCGGCGAGACTCAGGAGAATACAGAGAGACACACAGCTGGCGACGTTAGTCGCAACATGCACTCTCTGTTGGGAGTGCAGCAGCACCACTAGTCTCCTGGAAACCCTGTTTGCAGTACCTATAATATGTACTA------ATATATAGTATGTTGGTGAGGCTATGCCTC-------------------------GTTTGCTATTTTATTACGTACGTATTTACAGCGTGAACCAGTCTGCAGGACACAGGGTTGGACCCAGTGTCTTCTGGTGTAGCGTGTACTAGCGTCAAGCCACGTGACGGACAGCACTGGGTGTGGCTTTGCCATTGGTGCTGCGAGTCTCTTGGTGAGAGAC----------------

>JX047423

---------------------AAAACAACAAAACTCAACACAACACAACAAAACACAACCAAGCAAAACCAAGTTTCCTTTGCTCAGATTGTAGTGAACGGCTCGGTAGGAAAGGTTCCTCGAGATCACTCTCTGATTCTTCTTTC----------TCAACCAACTTCATTCAAGCGAGATGGCGGGCTCTTGGACTCACGTGACATACAAGTGGCAACCAGATGTCAACAACGCACGTGATGTGAAAAGAGTGATGGAGATGTTTGCAGCAAAACATCAACGTTACACTGAGGAGCAAAGGCTTGCTCACAACAGCAAGCTATTAAGGAAGGCTTGTGTCACTGGTGCTGAGTTTATTGAACCAGCACAGAAACCAAAATGTCGCCAGACATGGGTTGAAAAGTGCGACCACAACCCCACAGAGCACTTTGTTTATCAACGCTTC---ACACCTGAGAAGAAAGTGCTTAACACCAAACCTGAGACAACTTCTGTTACGAAATTAATCAGGGATGTCCTTGAGATTTCGAAGGGCAGTGGGATTAAAATTGAGTTGATCGATAAGCGTATTAAACGTAAGACTCAATTATCCATAAGGCAATACAATGGCAAATACTTCTTACACTGCAAAACCAGGCATGAAAATGGCCTGTTTAAACGCAAGGACATTGACATTAATGTCAAGTGGTTGCCCACCATTGAAGCCATTGCAAAATGCTACAGCACGGTGAATGCGGAAGAACTGCAAAGTCTCAGTAAAGGCAGTAGTGGTCTTACATTTATGCAAAACGATGAATTGTTCATCGTGCGTGGAAGGATGCATGGTGAGATTGTCAATAGTTTACACGAAAATAAGCACGTTATGGAAATTGAACACTATGCTGACCCACAAGCAAACAGTTTCTGGAAAGGTTACACAGACGCGTATGTTGAAAACAGAAACATATCTACCACTCATACAGAGCACACACCAACTATTAATTTAGAGGAGTGTGGTAAGAGAATGGCACTGTTAGAAATTCTATTCCACTCAACTTTTAAAATCACATGCAAAACGTGCAATATTGATGATCTGGAATTATCAGATGATGAATTTGGGGCCAAGTTATATAGCAATCTGCAGCGTATTGAAGAAAAGCAACGTGAATATCTCGCTAAAGATCAAAAACTTCTACGCATGATAAACTTCGTAAAGGATCGGTGTAACCCAAAATTCTCACATTTGCCTTTACTATGGCAAGTGGCGGAAACAGTAGGGCATTACACCGACAACCAATCAAAGCAGATAATTGACATCAGTGAGGCGCTTATCAAAGTTAATACTTTAACTCCTGATGATGCAGTGAAGGCCAGTGTAGCATTACTAGAAGTAGCACGGTGGTATAAAAATCGAAAGGAGTCACTTAAAACGGACACACTGGATTCGTTTCGAAACAAGATTTCTCCAAAGAGCACGATCAACGCAGCATTAATGTGTGATAACCAGTTAGATAAGAATGCAAATTTCGTATGGGGAAACAGAGAATACCATGCAAAGCGATTCTTCGCTAATTATTTTGAAGCCGTGGACCCAACTGATGCATATGAAAAACACGTAACACGTTTCAACCCCAATGGACAGCGGAAATTATCGATTGGCAAGCTAGTAATTCCACTAGATTTCCAGAAGATCAGAGATTCGTTCGTTGGCCTATCAATAAATAAACAACCACTGAGCAAAGCTTGCGTAAGCAAAATTGATGGAGGCTACGTATATCCATGTTGCTGCGTTACAACGGAGTTTGGAAAACCAGCATATTCTGAGATAATACCTCCAACGAAAGGACATATCACGATTGGAAACTCAGTGGACCCAAAAATAGTGGATTTACCGAATACTACACCACCAAGTATGTACATTGCAAAAGATGGATACTGTTACATTAACATATTCTTGGCAGCAATGATAAACGTCAATGAGGAATCCGCAAAAGATTACACTAAGTTTCTTAGGGACGAGTTGGTGGAACGTCTTGGTAAATGGCCAAAATTGAAAGATGTAGCTACAGCATGTTATGCGCTATCTGTGATGTTTCCTGAAATCAAGAACGCTGAGTTACCACCGATATTAGTTGACCATGAGAGCAAATCGATGCACGTAATAGATTCGTATGGTTCGCTGAGTGTGGGGTTTCACATCCTCAAAGCAAGCACCATTGGACAGTTGATAAAATTCCAATATGAATCGATGGAAAGCGAAATGCGAGAATACATAGTAGGAGGCACTTTAACACAACAAACTTTCAACACTCTACTTAAAACTCTCACAAAGAACATGTTCAAACCTGAGAAAATTAAGCAGATAATTGAGGAAGAGCCTTTCTTACTGATGATGGCAATCGCATCACCAACTGTGCTCATATCTTTATACAACAATTGTTACATAGAGCAAGCTATGACATACTGGATTGTTAAAAATCAAGGTGTTGCAGCAATATTCGCACAGCTCGAAGCATTAGCCAAAAAGACGTCTCAAGCTGAATTATTAGTTTTACAGATGCAAATACTTGAAAAGGCGTCTAATCAGTTGAGACTTGCAGTGACAGGACTAAACCATGTTGACCCCGCTAAGCGTCTTTTGTGGTCACACCTTGAAGCTATGACAACGCGATCGGAGATGAACAAGGAACTGATAGCAGAAGGCTATGCTTTATATGACGAGCGCTTGTACACTTTGATGGAAAAAAGTTACGTAGATCAATTAAACCAGTCATGGGCAGAATTATCATACTGTGGAAAATTTTCAGCAATATGGCGTGTGTTCAAAGTCAGGAAATATTACAAGCCGTCTTTAACCGTGAGAAAAAGCGTAGATTTAGGCGCTGTGTACAATATATCAGCTACGCATCTAATATCAGATTTAGCGCAGAGAAGTCGCGATCGAGCCAGCTCTATTTTAACCAAACTCCGCAACGGTTTTTATGATAAGTTAGAGAAGGCTAGAACTCGAGCAATTAAAACTGTTTATTGGTTCATACCTGACATATTTAGACTTATGCATATTTTCATAGTTCTTAGTTTATTAACAACTGTTGCAAACACTATTATTGTGACTATGAATGATTACAAAAAGCTGAAAAAGCAACAAAGAGAGGATGAGTATGAAGCTGGAATTAACGAGGTTCGAAAAATTCACGCTAATCTGATGAAAGAGCATAATGACAATCTAACATGTGAACAGTTTATCGAACATATGCGCCAGACGCATCCACGATTAATTGAAGCCACATTGGAGTTGACACACACAGGCGTCATTCATGAAGGAAAGAGCAACCTAGAAACCAATCTCGAGCAAGCTATGGCAGTGGGCACTTTACTCACAATGATGTTAGACCCACAGAAGAGCGATGCAGTATACAAAGTGTTGAATAAGATGAGAACTGTAATTAGCACATTTGAGCAGAATGTCCCATTTCCTTCAATCAACTTTACAAATATTTTAACACCATCAGTGGCGCAACAAAGTGTAGATGTTGATGAACCTCTAACTTTAAGTACTGACAAGAACCTAACAATAGATTTTGATACTAATCAAGATTTACCAGCGGACACGTTTAGCAATGATGTTACTTTCGAAGATTGGTGGGCAAATCAAATAAGCAACAATAGAACAGTCCCACATTATCGGCTTGGTGGAAAATTTGTAGAATTCACAAGAGAAAATGCAGCACATGTTAGCATTGAGCTCGCACACTCAAATCTTGAGAAGGAGTTTCTACTTAGAGGAGCTGTTGGCTCAGGAAAATCCACTGGTCTTCCATATCATCTTAGTATGCGTGGTAAGGTGCTTTTAATAGAACCAACAAGACCACTAGCCGAGAATGTCTGCAGACAGCTTCAAGGACCACCATTCAATGTCAGCCCGACACTACAAATGCGTGGTTTAAGCTCTTTTGGTTGCACGCCAATTACGATAATGACATCAGGTTTCGCACTGCACATGTATGCAAATAATCCGGATAAGATCTCTGACTACGATTTTATAATATTTGATGAGTGTCGTATCATGGAAGCACCAGCCATGGCGTTCTACTGTTTGTTGAAAGAATATGAATATAGAGGCAAAATCATTAAGGTGTCCGCAACACCACCAGGACGAGAGTGTGAGTTCTCTACTCAGCATCCAGTTGATATCCATGTGTGCGAAAATCTCACTCAGCAACAATTCGTTAGAGAGCTTGGTTCCGGATCAAACGTTGACGCAACAAAGTATGGAAACAACATACTTGTGTATGTTGCAAGCTACAACGACGTCGACTCATTAGCGCATGCTTTAACTGAATTACACTATTCAGTCATAAAGGTTGATGGGAGAACAATGAAACAGAACACTACAGGGATAGTGACAAATGGCACCTCTCAAAAGAAGTGTTTTGTCGTTGCTACGAATATCATTGAGAATGGTGTCACATTAGACGTTGACGTTGTGGTCGACTTCGGACTCAAAGTAACAGCTGAATTAGATGTTGACAACAGAGCTGTTCTATACAAGCGTGTTAGCATTTCATATGGTGAGCGAATTCAAAGGCTAGGGCGTGTTGGAAGAAACAAACCCGGAACAGTCGTTCGAATTGGAAAAACCATGAAGGGTTTGCAAGAAATCCCAGCCATGATAGCGACAGAAGCAGCATTCATGTGCTTTGCATATGGACTAAAGGTTATAACACACAATGTTTCAACAACACACTTAGCAAAGTGCACAGTCAAGCAAGCGCGAACAATGATGCAATTTGAGCTTTCACCATTTGTAATGGCAGAACTAGTCAAATTTGATGGCTCAATGCACCCACAAATCCATGAAGCACTTGTGAAATATAAGCTTAGAGATTCTGTAATAATGCTCAGACCTAACGCAATTCCCAAAGTTAATTTTCATAACTGGCTGACAGCACGTGATTACAACAGGATGGGTTGTTCACTAGAGCTAGAGGATCATGTCAAAATTCCATATTACATACGAGGCGTCCCTGACAAGTTATACGGGAAATTGTATGATATCATTTTACAATATAGTCCAACCAGTTGTTACGGAAGACTATCAAGTGCTTGTGCTGGGAAGGTAGCATACACCTTGCGAACGGACCCTTGTTCGCTACCACGAACAATAGCAATAATCAATGCATTAATCACAGAGGAGTATGCTAAGAGAGATCATTACCGTAATATGATAGCAAATCCTTCGTCTTCGCATGCATTCTCACTAAATGGATTAGTCTCCATGATTGCTTCAAGATACATGAAAGACCACACAAAAGAGAATATAGACAAACTTATTAAGGTGCGGGATCAACTACTCGAATTTCAAGGCATGGGTATGCAATTTCAAGATCCATCAGAACTAATGGACATTGGCGCCTTGAACACAGTTATACATCAAGGAATGGATGCAACAGCTGCATGCATTGGTCTCCAAGGACGATGGAATGCATCACTCATACAACGTGATCTCATGATTGCAGGAGGAGTATTCATTGGAGGAATATTAATGATGTGGAGCTTGTTCACTAAGTGGGGAACGACCAATGTATCACACCAAGGAAAGAACAAGCGTAGCCGACAAAAATTGAAGTTCAAGCAAGCTAGAGACACTAAATACGCATATGATGTGACAGGATCGGAAGAAACTCTTGGTGAAAACTTTGGAACAGCTTATACAAAGAAGGGCAAAGGAAAAGGAACCAAAGTTGGTCTTGGGGTGAAGCAGCATAGATTTCACATGATGTATGGTTTTGATCCTCAAGAGTACAACCTAATTCGTTTTGTCGACCCTCTTACAGGTGCAACATTAGATGAACAAATCCATGCTGATATTCGCTTAGTGCAAGAACACTTTGACATAATCCGAGAAGAGGCAGTCGCAAACGACACAATTGAGCGACAACACATATACGGAAATCCTGGTCTCCAAGCATTCTTCATACAGAATGGATCAGCTAATGCATTAAGAGTTGATTTAACACCACATTCACCTTTGCGTGTTATGACGAACAACAACATAGCAGGATTTCCAGAATATGAGGGCACATTACGACAAACTGGCACAGCTCTCACTGTACCTGTGAATCAAGTACCAGCAGCAAATGAGACAGGAGTTGCCCACGAATCCAAATCTATGATGGCTGGATTGGGCGATTACACACCTATTTCTCAGCAGCTCTGTCTCGTACAAAATGATTCTGAAGGAGTCAAACGTAATGTGTATGCAATTGGATATGGATCATATTTAATATCACCGGCGCATCTTTTCAAGTATAACAATGGTGAAATCACAATTAAATCCTCAAGAGGGCTGTATAAAATTAGAAATTCAGTCGATGTCAAATTGCACCCGATTGCACAGAGGGACATGGTCATAATTCAACTTCCAAAAGACTTCCCACCGTTCCCAATGCGACTTAAGTTCTCAACTCCGTCAAGAGATGTGCGTGTGTGCTTAGTTGGAATCAACTTTCAACAGAATCATACCACGTGCATAATATCCGAAAGCAGTGTGACAGCACCCAAAGGAAATGGTGATTTCTGGAAACATTGGATTTCAACTGTTGATGGGCAATGTGGGCTACCGTTAGTTGACGTTAAGAATAAACACATTGTCGGAATTCACAGCCTGGCCTCAACAAGTGGAAACACGAACTTTTTCGTTGCAATGCCTGAGAACTTCAATGAATATATATCTAATCTCGTGCAAACGAATAAGTGGGAAAAGGGATGGCATTACAACCCAAATCTTATTTCATGGTGTGGTCTAAACCTAGTTGATTCAGCACCTAAAGGATTGTTTAAAACATCAAAACTTGTTGAAGATTTGGATATGAGCGTTGAAGAACAATGCAAGGTGACAGAGACATGGTTGACGGAACACATCCAGGATAATCTACAGGTCGTTGCAAAGTGTCCAGGCCAACTTGTAACAAAGCATGTCGTTAAAGGCCCATGTCCGCATTTTCAACTGTATTTATCCACACATGATGAGGCAAAATTGTACTTTTCACCTTTGCTTGGAAAGTATGACAAGAGTAGGTTGAACAGGGCAGCATTTATCAAAGATCTTTCAAAGTACGCAAAGCCGATTTATATTGGAGAGATCAATTATGAAATCTTTGATAAGGCAGTTGATCGAGTTATAAGCATCCTCAGAAGTGTAGGAATGCTACAGTGTACATACGTGACGGACGAAGAAGAAATTTTCAATTCGTTAAATATGAACGCAGCCGTAGGTGCACTCTACACAGGAAAGAAGAAGGACTATTTCAAAGATTTCTCGAACGATGACAAAGCCGAAATCATCATGCGTTCATGTGAGCGCATCTACAATGGACAATTAGGTGTATGGAACGGTTCACTCAAAGCTGAAATACGACCAATAGAGAAAACCATACTAAACAAGACACGCACTTTCACAGCAGCGCCATTAGAAACTCTACTTGGTGGGAAAGTGTGTGTGGACGATTTCAACAATCAATTTTATTCACATCATCTTGAAGGCCCATGGACCGTAGGAATCACAAAGTTTTATGGAGGATGGAACCGACTTTTGGAGAAATTGCCAGAAGGATGGATTTATTGCGATGCAGATGGATCCCAGTTTGACAGCTCACTAACTCCATATCTTATTAATGCTGTATTGCACATTCGCTTACATTTCATGGAAGAATGGGAGTTGGGAGCTCAGATGTTGCGAAATTTATACACAGAGATTGTTTATACGCCAATCGCAACGCCTGATGGGTCTGTCATCAAGAAATTCAAAGGAAATAATAGTGGGCAACCATCTACAGTCGTTGACAACACGCTTATGGTTATTATAGCATTTAATTACGCAATGTTATCAAGTGGCATTCCTGAAGACAAAATTGACGACTGCTGTAGAATGTTTGCAAACGGTGACGACTTACTCTTGGCAGTGCATCCGGATTACGAATATATACTGGACGGATTTCAAAATCATTTTGGAAACCTTGGCCTTAACTTTGAGTTCACATCGAGGACAAAGGACAAATCAGAGTTATGGTTTATGTCAACACAAGGAGTCAAGTGTGAAGGTATCTACATACCAAAACTCGAAAGGGAAAGAATAGTCGCAATCCTTGAATGGGACCGATCGAACTTGCCTGAGCATCGTCTTGAAGCTATCTGTGCAGCCATGGTTGAAGCATGGGGTTACCCAGATTTAGTTCATGAAATTCGTAAGTTTTACGCGTGGCTTCTTGAAATGCAACCCTTCGCGAACCTGGCAAAGGAAGGCATGGCGCCATACATAGCAGAAACAGCACTCCGCAACCTCTACCTTGGAACAGGCATCAAAGAAGAAGAAATTGAAAAATATTTTAGACAGTTTGTCAAGGATCTTCCTGGATACGTAGAAGACTACAATGAAGAAGTTATTCATCAATCTGGTCAAGTTGACGCAGGGAGACAGGGCGGTAGCGGCGCTCAAGGAGGCACACCACCAGCAGGA---------------------------------------------------AGTGGAGGCAC----TGGATCTGGCACTCAAGGCAATGGGGGTCAGACGGGA---------TCCCAAG---GAAGTGGTGGTCAACAAGGGTCCGGTGGGGGCACTGGTCAAGGAGCAGCTGGAAACAACGGCGG------------AGGTCAGACAGGAGGCTCTAGTGGG------------ACAGCTGGTCAGAGAGATAAGGACGTTGACGCAGGCTCGGCTGGAAAGATATCCGTGCCAAAGCTTAAAGCCATGTCAAAGAAAATGCGCTTGCCAAAGGCAAAAGGAAAAGACGTCTTGCATTTGGACTTTTTGTTGACATATAAGCCACAACAGCAGGACATATCGAACACAAGAGCAACTAAGGAAGAGTTCGATAGATGGTACGACGCCATAAAGAAGGAGTACGAGATCGATGATACACAAATGACAGTTGTCATGAGTGGTCTGATGGTCTGGTGCATCGAAAATGGTTGCTCACCAAACATAAACGGAAATTGGACGATGATGGATGGGGATGAACAAAGAGTTTTCCCACTAAAACCAGTTATTGAAAACGCATCTCCAACTTTTCGACAAGTTATGCATCATTTCAGTGATGCAGCTGAAGCGTATATAGAATACAGAAATTCTACTGAGCGATACATGCCAAGATATGGACTTCAGCGAAATCTCACCGACTATAGCTTAGCGCGGTATGCTTTTGATTTTTATGAAATGACTTCACGCACACCAGCTAGAGCTAAGGAAGCCCACATGCAG---ATGAAAGCCGCAGCAGTTCGTGGTTCAAACACACGACTGTTCGGCTTGGACGGAAATGTCGGCGAGACTCAGGAGAATACAGAGAGACACACAGCTGGCGACGTTAGTCGCAATATGCACTTTCTGTTGGGAGTGCAGCAACACCACTAGTCTCCTGGAAACCCTGTTTGCGGTACCTATAATATATACTA------ATATATAGTACGTTGGTGAGGCTTTGCCTC-----------------------GGTTTTACTATCTTATTATGTATGTATTTACAGCGCGAACCAGTTTGCAGCATGCAGGGTTGGACCCAGTGTGTTCTGGTGTAGCGTGTACTAGCGTCGAGCCATGAGACGGACTGCACTGGGTGTGGCTGTGCCACTTGTGTTGCGAGTTTCCTGGTAAGAGAC----------------

>JX047421

---------------------AAAACAACAAAACTCAACACAACACAACAAAACACAACCAAGCAAAACCAAGTTACCTTCGCTCAGATTGTAGTGAACGGCTCGGTGAGAAAGGTTCCCCGAGATCACTCTCTGATTCTTCTCTCTCAATCAATTTCAACCAACTTCATTCAAGCGAGATGGCTAGCTCATGGACGCACGTGACCTACAAGTGGCAACCAGATGTAAACAACGCACGTGATGTCAGGAAGGTAATGGAGATGTTTGCAGCAAAACATCAACGTTACACTGAGGAGCAGAGGCTTGCCCATAATAGCAAGCTTCTGAGGAGAGCATATGTTACTAGTGCTGAGTTTGTTGAACCAGCACAGAAACCCAAGTGTCACCAGACATGGGTTGAGAAATGCAACCACAACCCCACAGAGCACTTCGTTTATCAACGCTTC---ACACCTGAGAAGAAAGTGCTTAATACCAAACCTAAAACAACTTCTGTCACGAAGTTAATTAGGGATGTCCTTGAAATTTCGAAGGGCAGTGGGATAAAAATTGAGTTAATTGACAAGCGTATCAAACGCAAGACTCAATTATCCGTAAGGCAACACAATGGCAAAGATTTCCTGCACTGTAAAACCAAGCATGAAAATGGCCTGTTTAAACGCAGGGACATTGACATTAGTGTCAAGTGGTTGCCCACCATTGAAGCCATTGCAAAATGCTACAGCACGGTGAATGCAGAAGAACTGCAAAGTCTCAGTAGAGGCAGTAGTGGTCTAACATTCATGCAAAACGATGAATTATTCATCGTGCGTGGAAGGATGCATGGTGAGATTGTCAATAGTTTACATGAGAATAAGCACGTAATGGAAATCGAACACTATGCTGATCCACAAGCAAACAGTTTCTGGAAAGGCTACACAGATGCGTATGTCGAGAACAGAAACATATCTACCACTCACACAGAGCACACACCAACTATTAATTTAGAAGAGTGTGGTAAGAGAATGGCACTGTTAGAAATCTTATTCCATTCAACTTTTAAAATAACATGCAAAACGTGCAATATCGATGACCTTGAATTATCAGATGATGAATTTGGGGCCAAGTTATACAGTAATCTGCAGCGCATTGAAGAAAAGCAACGTGAATATCTTGCCAAAGATCAGAAACTCTCACGCATGATTCACTTTGTGAAGGATCGATGTAATCCAAAGTTTTCACATTTGCCTTTACTATGGCAAGTGGCGGAAACAGTAGGGCATTACACCGACAACCAATCAAAGCAGATAATTGACATCAGTGAGGCGCTTATCAAAGTTAATACTTTAACTCCTGATGATGCAGTGAAGGCCAGTGTAGCATTACTAGAAGTAGCACGGTGGTATAAAAATCGAAAGGAGTCACTTAAAACGGACACACTGGATTCATTTCGAAACAAGATTTCTCCAAAGAGCACGATCAACGCAGCATTAATGTGTGATAACCAGTTAGATAAGAATGCAAATTTCGTATGGGGAAACAGAGAATACCATGCAAAGCGATTCTTCGCTAATTATTTTGAAGCCGTGGACCCAACTGATGCATATGAAAAACACGTAACACGTTTCAACCCCAATGGACAGCGGAAATTATCGATTGGCAAGCTAGTAATTCCACTAGATTTCCAGAAGATCAGAGATTCGTTCGTTGGCTTATCAATAAATAAACAACCACTGAGCAAAGCTTGCGTAAGCAAAATTGATGGAGGCTACGTATATCCATGTTGCTGCGTTACAACGGAGTTTGGAAAACCAGCATATTCTGAGATAATACCTCCAACGAAAGGACATATCACGATTGGAAACTCAGTGGACCCAAAAATAGTGGATTTACCGAATACTACACCACCAAGTATGTACATTGCAAAAGATGGATACTGTTACATTAACATATTCTTGGCAGCAATGATAAACGTCAATGAGGAATCCGCAAAAGATTACACTAAGTTTCTTAGGGACGAGTTGGTGGAACGTCTTGGTAAATGGCCAAAATTGAAAGATGTAGCTACAGCATGTTATGCGCTATCTGTGATGTTTCCTGAAATCAAGAACGCTGAGTTACCACCGATATTAGTTGACCACGAGAGCAAATCGATGCACGTAATAGATTCGTATGGTTCGCTGAGTGTGGGGTTTCACATCCTCAAAGCAAGCACCATTGGACAGTTGATAAAATTCCAATATGAATCGATGGAAAGCGAAATGCGAGAATACATAGTAGGAGGCACTTTAACACAACAAACTTTCAATACTCTACTTAAAACTCTCACAAAGAACATGTTCAAACCTGAGAAAATTAAGCAGATAATTGAGGAAGAGCCTTTCCTACTGATGATGGCAATCGCATCACCAACTGTGCTCATATCTTTATACAACAATTGTTACATAGAGCAAGCTATGACATACTGGATTGTTAAAAATCAAGGTGTTGCAGCAATATTCGCACAGCTCGAAGCATTAGCCAAAAAGACGTCTCAAGCTGAATTATTAGTTTTACAGATGCAAATACTTGAAAAGGCGTCTAATCAGTTGAGACTTGCAGTGACAGGACTAAACCATGTTGACCCCGCTAAGCGTCTTTTGTGGTCACACCTTGAAGCTATGACAACGCGATCGGAGATGAACAAGGAACTGATAGCAGAAGGCTATGCTTTATATGACGAGCGCTTGTACACTTTGATGGAAAAAAGTTACGTAGATCAATTAAACCAGTCATGGGCAGAATTATCATACTGTGGAAAATTTTCAGCAATATGGCGTGTGTTCAAAGTCAGGAAATATTACAAGCCGTCTTTAACCGTGAGAAAAAGCGTAGATTTAGGCGCTGTATACAATATATCAGCTACGCATCTAATATCAGGTTTAGTGCAGAAAAGTCGCGATCAAGTCAGCTCTACTTTAACCAAACTCCGCAACGGTTTTTATGATAAATTAGAGAAGACTAGAGTGCGTGCAATCAAAACAGTATATTGGTTTATACCCGATATATTTAGACTTGCTCACATTTTTATAGTGCTAAGTTTGTTAACCACCATAGCTAACACGATCGTAATAACTATGAATGACTATAAAAAGTTGAAAAAGCAACAAAGAGAGGATGCGTATGAGGCTGAGATAAACGAAGTTCGCAAGATTCATGCCGCCTTGATGAAGGAGCATAACGACAATTTAACATGCGAACAGTTCATTGAACATATGCGTCAAACACATCCACGATTAGTTGAAGCCACATTGGAGTTGACCCACACAGGTGTGATTCATGAAGGAAAGTCTAATCTAGAAACCAACCTTGAGCAAGCTATGGCTGTGGGAACACTACTCACGATGATACTTGATCCTCAGAAGAGTGACGCTGTGTACAAAGTTCTAAACAAGATGCGAACAGTGATTAGTACATTTGAACAGAATGTCTCATTCCCTTCAATTAACTTCACTAACATCTTGACACCACCCGTAACACAGCAGAGTGTGGACGTCGATGAGCCTCTTACTTTGAGCACTGACAAGGACTTAACGATAGATTTTGATACAAATCAAGATTTACCTGCAGACACTTTCAGTAACGATGTTACATTCGAGGATTGGTGGGCAAATCAGATAAGCAATAATAGAACAGTTCCACACTATCGTCTCGGTGGTAAGTTTGTAGAATTTACAAGGGAAAACGCAGCACTTGTTAGTATTGAATTAGCTCGCTCGAACATTGAAAAGGAATTTCTTTTAAGGGGTGCGGTTGGCTCAGGAAAGTCCACAGGGCTGCCATACCATCTTAGTGCGCGTGGAAAAGTACTTCTAATCGAACCAACAAGACCACTTGCCGAGAATGTATGCAGACAGCTACAAGGGCCACCATTCAATGTTAGCCCGACACTTCAAATGCGTGGGTTGAGCTCTTTTGGCTGCACGCCAATCACGATCATGACCTCTGGTTTTGCATTACATATGTACGCAAACAATCCGGACAAGATATCTAATTACGACTTCATCATATTTGATGAATGTCATATCATGGAAGCACCAGCTATGGCATTTTATTGTTTGCTGAAGGAATATGAATATCGAGGCAAAATCATTAAAGTCTCCGCAACACCACCTGGAAGAGAATGTGAATTTACCACACAACATCCAGTTGACATTCACGTCTGCGAGAACCTCACACAACAGCAATTTGTAAGGGAACTTGGCACGGGTTCAAATGTCGACGCAACGAAATATGGAAATAACATACTTGTTTACGTTGCTAGCTACAATGACGTAGATTCGCTATCACACGCGCTAACTAAATTACATTATTCAGTTATCAAAGTTGACGGTAGAACTATGAAGCAGAACACCACAGGCATTGTAACTAATGGCACATCACAAAAGAAGTGTTTTGTCGTTGCAACGAACATAATCGAGAATGGTGTTACATTGGATGTTGATGTCGTAGTGGACTTTGGGCTAAAAGTCACAGCTGAGCTGGATGTTGACAATAGAGCAATTCTATACAAACGTGTCAGCATCTCGTATGGTGAGCGTATTCAAAGACTTGGTCGTGTTGGAAGGAACAAACCCGGAACAGTGGTCCGCATTGGGAAAACTATGAAAGGTCTGCAGGAAATCCCAGCGATGATAGCAACGGAAGCTGCTTTTATGTGTTTTGCATACGGACTCAAAGTTATAACACACAATGTATCTACAACACACTTAGCTAAATGCACGGTAAAACAAGCAAGAACAATGATGCAATTTGAACTATCACCATTTGTAATGGCAGAATTAGTTAAATTCGATGGTTCAATGCATCCACAAATTCATGAAGCACTCGTAAAATACAAACTTAGAGATTCTGTGATAATGCTAAGACCAAATGCTATCCCGAAGGTCAACTTTCACAACTGGCTGACTGCACGTGATTATAACAGGATGGGCTGTACAGTAGAACTTGAAGATCATGTGAAAATACCGTACTATATACGAGGGATTCCTGATAAACTATATGGAAAATTGTACGATATCATTTTACAATACAGCCCAACTAGTTGTTATGGAAGACTATCAAGTGCCTGTGCGGGAAAAGTAGCATACACCCTGCGCACTGATCCTTGTTCGTTACCACGCACGATAGCTATCATTAATGCCTTAATTACTGAAGAGTATGCAAAGAGGGACCATTACAGAAATATGATAGCAAACCCCTCATCATCGCACGCCTTTTCACTCAATGGGCTGGTATCCATGATCGCTTCTCGGTATATGAAAGACCACACGAAGGAAAACATTGACAAGCTTGTAAGAGTGCGCGACCAACTACTTGAGTTTCAAGGCACAGGTATGCAATTTCAAGATCCTTCAGAATTGATGGACATTGGTGCATTAAACACAGTTATTCACCAAGGAATGGACGCCACGGCTGCTTGTATTGGATTGCAAGGGCGCTGGAATGCTTCGCTCATTCAACGCGATTTGATGATATCAGCAGGGGTCTTCACAGGAGGAATTCTCATGATGTGGTATCTTTTCACAAAATGGAGCAAGACAGAAGTGTCACACCAAGGAAAGAACAAACGCAGCCGGCAAAAACTACGATTCAAAGAGGCACGTGATAATAAATATGCCTATGACGTAACAGGATCAGAGGAAATTCTTGGTGAGAATTTTGGAACCGCATACACTAAGAAAGGAAAAGGAAAGGGGACAAAAGTTGGCCTTGGAGTCAAACAGCACAAATTCCACATGATGTACGGGTTTGATCCACAAGAATATAACTTAATCCGCTTCGTGGATCCTTTAACAGGTGCGACACTAGACGAGCAAATTCATGCAGATATACGTTTAGTGCAAGAGCACTTTAGCGTCATTAGAGATGAAGCAGTGGCAAACGACACAATTGAAAGACAACACATTTACAGCAATCCTGGACTACAAGCGTTCTTCATACAAAATGGATCTGCAAATGCACTGAGAGTTGATTTAACACCACACACACCACTGCGTGTCGTAACCAATAACAATGTAGCAGGTTTCCCAGAATATGAAGGTACACTTCGACAAACAGGGACAGCTTTACAGATACCCGTGAATCAAGTTCCAGCTGCGAATGAAGCGGGGGTAGCACATGAGTCGAAATCGATGATGGCAGGGTTAGGCGATTACACCCCAATATCACAGCAGTTGTGTTTAGTCCAGAATGACTCAGATGGAATCAAAAGGAATGTGTACTCAATTGGATATGGATCATATCTCATTTCACCGGCACATTTATTTAAATATAACAATGGCGAAATAACAATTAAATCTTCAAGAGGTTTGTATAAGATCAGAAATTCAGTAGAACTCAAGTTGCATCCCATTGCACATAGAGATATGGTCATTATTCAACTTCCGAAAGATTTTCCGCCGTTCCCAATGCGTCTCAAGTTTTCTAAACCATCTAGAGAGTCAAGAGTGTGCTTAGTTGGAGTGAACTTCCAACAAAACTACAGCACATGCATTGTATCGGAGAGTAGTGTCACAGCACCAAAAGGCAATGGAGATTTCTGGAAACACTGGATATCCACAGTGGACGGACAATGCGGCCTCCCATTAGTAGATGTCAAGAGCAAGCACATAGTTGGAATACACAGCCTTGCATCAACTAGTGGAAGCACTAACTTTTTCGTCGCCATGCCTGAAGATTTCAATGACTACATTCACAATCTTGTGCAAACCAACAAGTGGGAAAAAGGATGGCATTACAACCCAAATCTCATTTCATGGTGTGGTCTCAATCTAGTTGATTCAGCTCCAAAGGGTCTCTTCAAAACTTCGAAATTAGTGGAAGACCTTGACATGAGTGTTGAAGAGCAGTGCAAGGCTACAGAGACATGGCTCACAGAATGCATTCAGGACAATTTACAGGTTGTCGCAAAATGCCCAGGCCAACTTGTCACCAAGCACGTTGTCAAAGGCCCATGCCCACACTTTCAGCTATATCTGTCAACACATGATGAAGCCAAAGCATACTTTGCACCACTACTCGGAAAGTACGATAAGAGCAGATTGAACAGAGCAGCTTTTATCAAAGACATTTCAAAATATGCAAAACCAATCTACATTGGAGAAATCAATTACGATGTCTTTGAAAAGGCTATAGAACGTGTGATTAAAATCCTTAGAGATGTGGGAATGCAACAATGCACGTATGTCACGGATGAGGATGAAATATTCCAGTCACTCAATCTCAACGCCGCAGTTGGTGCCTTATACACAGGAAAGAAGAAAGACTATTTCAAGGATTTCTCAAATGAAGACAAATCAGAAATCATTATGAGATCCTGTGAGCGTATCTACAACGGACAACTTGGTGTGTGGAATGGTTCACTCAAAGCTGAAATAAGGCCCATAGAGAAAACTATGTTAAACAAGACTCGAACTTTCACAGCAGCACCATTAGAAACTCTACTTGGTGGCAAAGTCTGCGTCGATGATTTCAACAATCAGTTCTACTCACACCACTTAGAGGGTCCTTGGACAGTTGGAATTACAAAGTTCTATGGTGGGTGGAATCGTTTATTAGAAAAATTACCAGACGGTTGGATTTACTGCGACGCTGATGGATCACAGTTTGACAGCTCTTTGACACCATACCTTATCAACGCTGTATTACATATTCGATTACAATTCATGGAGGAATGGAACTTAGGAGAACAAATGTTGCGAAACTTGTACACCGAAATCGTATACACACCAATTGCAACACCAGATGGATCTGTAATTAAGAAATTCAAAGGAAATAACAGTGGGCAGCCGTCAACAGTTGTAGACAACACACTCATGGTGATATTAGCGTTTAATTATGCAATGTTGTCGAGTGGCGTTAAAGAGGAAGAAATAGATAACTGTTGCCGAATGTTCGCCAATGGTGATGATCTGTTGCTTGCAGTGCACCCAGACTTCGAACATATATTGGATGGATTTCAAAATCACTTTGGAAACTTGGGTCTCAACTTTGAGTTCACATCACGAACAAGAAACAAGTCAGAGTTATGGTTCATGTCCACACGAGGTATCAAATGCGAAGGCGTCTATATACCGAAGCTTGAGAAAGAAAGAATAGTTGCCATACTCGAGTGGGATCGGTCAAACTTACCTGAGCATCGTCTTGAAGCTATCTGTGCAGCCATGGTTGAAGCATGGGGTTACTCAGATTTAGTTCATGAAATTCGAAAGTTTTACGCGTGGCTTCTCGAAATGCAACCCTTCGCGAACCTGGCAAAGGAAGGCATGGCGCCATACATAGCAGAAACAGCACTTCGCAACCTTTACCTTGGAACAGGCATCAAAGAAGAAGAAATTGAAAAATATTTTAAGCAGTTTGTCAAGGATCTTCCTGGATACGTAGAAGATTACAATGAAGAAGTTATTCATCAATCTGGCCATGTTGATGCAGGGAGACAAGGCGGTAGCGGTGCTCAAGGAGGCACACCACCAGCAGGA---------------------------------------------------AGTGGAGGCAC----TGGATCTGGCACTCAGGGCAATGGGGGTCAGACGGGA---------TCCCAAG---GAAGTGGTGGTCAACAAGGGTCCGGTGGGGGCACTGGCCAAGGAGCAGCTGGAAGCAACGGCGG------------AGGCCAGACAGGAGGCTCTAGCGGG------------ACAGCTGGTCAAAGAGATAAGGATGTTGACGCAGGCTCGGCTGGAAAGATATCCGTACCAAAGCTTAAAGCCATGTCAAAGAAAATGCGCTTGCCAAAGGCAAAAGGAAAAGATGTCTTGCATCTGGACTTTTTGTTGACATACAAGCCACAACAGCAAGACATATCGAACACAAGAGCAACTAAGGAAGAGTTCGATAGATGGTACGACGCCATAAAGAAGGAGTACGAGATCGATGATACACAAATGACAGTTGTCATGAGTGGTCTGATGGTCTGGTGCATCGAGAATGGTTGCTCACCAAATATAAACGGGAATTGGACGATGATGGATGGAGAAGAACAACGAGTTTTTCCATTAAAACCAGTCATTGAAAACGCATCTCCAACCTTCCGACAAATAATGCACCACTTTAGTGATGCAGCTGAAGCGTACATAGAGTATAGAAACTCTACAGAGCGATATATGCCAAGATACGGACTTCAGCGAAATCTCACCGACTATAGCCTAGCACGGTATGCATTTGATTTCTATGAAATGACCTCACGCACACCAGCTAGAGCTAAGGAAGCCCACATGCAG---ATGAAAGCCGCAGCAGTTCGTGGTTCAAACACACGACTGTTCGGTCTGGACGGAAATGTCGGCGAGACTCAGGAGAATACAGAGAGACACACAGCTGGTGACGTTAGTCGCAACATGCACTCTCTGTTGGGAGTGCAGCAGCACCACTAGTCTCCTGGAAACCCTGTTTGCAGTACCTATAATATGTACTA------ATATATAGTATGTTGGTGAGGCTACGCCTC-------------------------GTTTGCTATTTTATTACGTACGTATTTACAGCGTGAACCAGTCTGCAGGACACAGGGTTGGACCCAGTGTCTTCTGGTGTAGCGTGTACTAGCGTCGAGCCTCGTGACGGACAGCACTGGGTGTGGCTTTGCCATTGGTGCTGCGAGTCTCTTGGTGAGAGAC----------------

>JX047417

---------------------AAAACAACAAAACTCAACACAACACAACAAAACACAACCAAGCAAAACCAAGTTTCCTTTGCTCAGATTGTAGTGAACGGCTCGGTAGGAAAGGTTCCTCGAGATCACTCTCTGATTCTTCTTTC----------TCAACCAACTTCATTCAAGCGAGATGGCGGGCTCTTGGACTCACGTGACATACAAGTGGCAACCAGATGTCAACAACGCACGTGATGTGAGAAGAGTGATGGAGATGTTTGCAGCAAAACATCAACGTTACACTGAGGAGCAAAGGCTTGCTCACAACAGCAAGCTATTAAGGAAGGCTTGTGTCACTAGTGCTGAGTTTATTGAACCAGCACAGAAACCAAAATGTCGTCAGACATGGGTTGAAAAGTGCGACCACAACCCCACAGAGCACTTTGTTTATCAACGCTTC---ACACCTGAGAAGAAAGTGCTTAACACCAAACCTGAGACAACCTCTGTTACGAAATTAATCAGGGATGTCCTTGAGATTTCGAAGGGCAGTGGGATTAAAATTGAGTTGATCGATAAGCGTATTAAACGTAAGACTCAATTATCCATAAGGCAATACAATGGCAAAAACTTCTTGCACTGCAAAACCAGGCATGAAAATGGCCTGTTTAAACGCAAGGACATTGATATTAATGTCAAGTGGTTGCCCACCATTGAAGCCATTGCAAAATGCTACAGCACGGTGAATGCGGAAGAACTGCAAAGTCTCAATAAAGGCAGTAGTGGTCTTACATTCATGCAAAACGATGAATTGTTCATCGTGCGTGGAAGGATGCATGGTGAGATTGTCAATAGTTTACACGAAAATAAGCACGTTATGGAAATTGAACACTATGCTGACCCACAAGCAAACAGTTTCTGGAAAGGTTACACAGACGCGTATGTCGAAAACAGAAACATATCTACCACTCACACAGAGCACACACCAACTATTAATTTAGAGGAGTGTGGTAAGAGAATGGCACTGTTAGAAATTCTATTCCACTCAACTTTTAAGATCACATGCAAAACGTGCAATATTGATGATCTGGAATTATCAGATGATGAATTTGGGGCCAAGTTATATAGCAATCTGCAGCGTATTGAAGAAAAGCAACGTGAATATCTCGCTAAAGATCAAAAACTTCTACGCATGATACACTTTGTAAAGGATCGGTGTAACCCAAAATTTTCACATTTGCCTTTATTATGGCAAGTGGCGGAAACAGTAGGGCATTACACCGACAACCAATCAAAGCAGATAATTGACATCAGTGAGGCGCTTATCAAAGTTAATACTTTAACTCCTGATGATGCAGTGAAGGCCAGTGTAGCATTACTAGAAGTAGCACGGTGGTATAAAAACCGAAAGGAATCACTCAAAACGGACACACTGGATTCATTTCGAAGCAAGATTTCTCCAAAGAGCACGATCAACGCAGCATTAATGTGTGATAACCAGTTAGACAAGAATGCAAATTTCGTATGGGGAAACAGAGAATACCATGCAAAGCGATTCTTCGCTAATTATTTTGAAGCCGTGGACCCAACTGATGCATATGAAAAACACGTAACACGCTTCAACCCTAATGGACAGCGGAAATTATCGATTGGTAAGCTAGTAATTCCACTAGACTTCCAGAAGATCAGAGATTCGTTCGTTGGCCTATCAATAAATAAACAACCACTGAGCAAAGCTTGCGTAAGCAAAATTGATGGAGGCTACGTATATCCATGTTGCTGCGTTACAACGGAGTTTGGAAAACCAGCATATTCTGAGATAATACCTCCAACGAAAGGACATATCACGATTGGAAACTCAGTGGACCCAAAAATAGTGGATTTACCGAATACTACACCACCAAGTATGTACATTGCAAAAGATGGATACTGTTACATTAACATATTCTTGGCAGCAATGATAAACGTCAATGAGGAATCCGCAAAAGATTACACTAAGTTTCTTAGAGATGAGTTGGTGGAACGGCTTGGTAAATGGCCAAAATTGAAAGATGTAGCTACAGCATGTTATGCGCTATCTGTGATGTTTCCTGAAATCAAGAACGCTGAGTTACCACCGATATTAGTTGACCATGAGAGCAAATCAATGCACGTAATAGATTCGTATGGTTCGCTGAGTGTGGGGTTTCACATCCTCAAAGCAAGCACCATTGGACAGTTGATAAAATTCCAGTATGAATCGACGGAAAGCGAAATGCGAGAATACATAGTAGGAGGCACTTTAACACAACAAACTTTCAACACTCTACTTAAAACTCTCACAAAGAACATGTTCAAACCTGAGAAAATTAAGCAGATAATTGAGGAAGAGCCTTTCTTACTGATGATGGCAATTGCATCACCAACTGTGCTCATATCTTTATACAATAATTGTTACATAGAGCAAGCTATGACATACTGGATTGTTAAAAATCAAGGTGTTGCAGCAATATTCGCACAGCTCGAAGCATTAGCCAAAAAGACGTCTCAAGCTGAATTATTAGTTTTACAGATGCAAATACTTGAAAAGGCGTCTAATCAGCTGAGGCTTGCAGTGACAGGACTAAACCATGTTGACCCCGCTAAGCGTCTTTTATGGTCACATCTTGAAGCTATGACAACGCGATCGGAGATGAACAAGGAACTGATAGCAGAAGGCTATGCTTTATATGACGAGCGCTTGTACACTTTGATGGAAAAAAGTTACGTAGATCAATTAAACCAGTCATGGGCAGAATTATCATACTGTGGAAAATTTTCAGCAATATGGCGTGTGTTCAAAGTCAGGAAATATTACAAGCCGTCTTTGACCGTGAGAAAAAGCGTAGATTTAGGCGCTGTATACAATATATCAGCTACGCATCTAATATCAGGTTTAGTGCAGAAAAGTCGCGATCAAGTCAGCTCTACTTTAACCAAACTCCGCAACGGTTTTTATGATAAATTAGAGAAGACTAGAGTGCGTGCAATCAAAACAGTATATTGGTTTATACCCGATATATTTAGACTTGTTCACATTTTTATAGTGCTAAGTTTGTTAACCACCATAGCTAACACGATCGTAATAACTATGAATGACTATAAAAAGTTGAAAAAGCAACAAAGAGAGGATGCGTATGAGGCTGAGATAAACGAGGTTCGCAAGATTCATGCCGCCTTGATGAAGGAGCATAACGACAATTTAACATGCGAACAGTTCATTGAACATATGCGTCAAACACATCCACGATTAGTTGAAGCCACATTGGAGTTGACCCACACAGGTGTGATTCATGAAGGAAAGTCTAATCTAGAAACCAACCTTGAGCAAGCTATGGCTGTGGGAACACTACTCACGATGATACTTGATCCTCAGAAGAGTGACGCTGTGTACAAAGTTCTAAACAAAATGCGAACAGTGATTAGTACATTTGAACAGAATGTCTCATTCCCTTCAATTAACTTCACCAACATCTTGACACCACCCGTAACACAGCAGAGTGTGGACGTCGATGAGCCTCTTACTTTGAGCACTGACAAAAACTTAACGATAGATTTTGATACAAATCAAGATTTACCTGCAGACACTTTCAGTAACGATGTTACATTCGAGGATTGGTGGGCAAATCAGATAAGCAATAATAGAACAGTTCCACACTATCGTCTCGGTGGTAAGTTTGTAGAATTTACAAGGGAAAACGCAGCACTTGTTAGTATTGAATTAGCTCACTCGAACATTGAAAAGGAATTTCTTTTAAGGGGTGCGGTTGGCTCAGGAAAGTCCACAGGGCTGCCATACCATCTTAGTGCGCGTGGAAAAGTACTCCTAATCGAACCAACAAGACCACTTGCCGAGAATGTATGCAGACAGCTACAAGGGCCACCATTCAATGTTAGCCCGACACTTCAAATGCGTGGGTTGAGCTCTTTTGGTTGCACGCCAATCACGATCATGACCTCTGGTTTTGCATTACATATGTACGCAAACAATCCGGATAAGATATCTGATTACGACTTCATCATATTTGATGAATGTCATATCATGGAAGCACCAGCTATGGCATTTTATTGTTTGCTGAAGGAATATGAATATCGAGGCAAAATCATTAAAGTCTCCGCAACACCACCTGGAAGAGAATGTGAATTTACCACACAACATCCAGTTGACATTCACGTCTGCGAGAACCTCACACAACAGCAATTTGTAAGGGAACTTGGCACGGGTTCAAATGTCGACGCAACGAAATATGGAAATAACATACTTGTTTACGTTGCTAGCTACAATGACGTCGATTCGCTATCACACGCGCTAACTGAATTACATTATTCAGTTATCAAAGTCGACGGTAGAACTATGAAGCAGAACACCACAGGCATTGTAACTAATGGCACATCACAAAAGAAGTGTTTTGTCGTTGCAACGAACATAATCGAGAATGGTGTTACATTGGATGTCGATGTCGTAGTGGACTTTGGACTAAAGGTTACAGCTGAACTAGATGTTGACAACAGAGCTATACTGTACAAGCGCGTTAGCATTTCATATGGTGAGCGAATTCAAAGGCTAGGGCGTGTTGGAAGGAATAAACCCGGAACAGTCGTTCGAATTGGAAAAACCATGAAGGGTTTGCAAGAGATCCCAGCCATGATAGCGACAGAAGCAGCATTCATGTGCTTCGCATACGGGTTAAAAGTTATAACGCACAATGTTTCAACAACACACTTAGCAAAGTGCACAGTAAAACAAGCGCGAACAATGATGCAATTTGAGCTCTCGCCATTTGTGATGGCAGAACTAGTCAAATTTGATGGCTCAATGCATCCGCAAATCCACGAAGCACTCGTGAAATATAAGCTCAGGGATTCTGTAATAATGCTCAGACCTAATGCAATTCCCAAAGTTAATTTTCACAACTGGCTGACAGCACGTGACTACAACAGAATGGGTTGCTCACTAGAGCTAGAGGATCATGTCAAGATTCCATATTACATACGAGGCGTCCCTGACAAGTTATACGGGAAGTTGTATGATATCATTCTACAGTACAGTCCAACCAGTTGCTATGGAAGATTATCAAGTGCCTGTGCTGGGAAAATAGCATACACCTTGCGAACCGATCCTTGTTCGCTACCACGAACAATAGCAATAATCAATGCATTAATCACAGAAGAATATGCGAAGAGGGATCATTACCGCAACATGATAGCAAATCCTTCGTCCTCGCATGCATTCTCACTAAATGGATTAGTCTCTATGATTGCTTCAAGATATATGAAAGACCACACAAAGGAGAATATAGACAAACTTATTAAAGTGCGAGACCAATTACTCGAATTCCAAGGCATGGGTATGCAATTTCAAGATCCATCAGAACTAATGGATATTGGTGCCTTGAACACAGTTATACATCAAGGAATGGATGCAACAGCAGCATGTATTGGACTCCAAGGACGATGGAATGCATCGCTCATACAACGCGATCTCATGATTGCAGGAGGAGTATTCATTGGGGGTATCCTGATGATGTGGAGCTTATTCACTAAGTGGGGAACAACCAATGTATCACACCAAGGAAAGAACAAGCGTAGACGACAAAAATTGAAATTCAAGCAAGCCAGAGACACTAAATACGCATATGATGTGACAGGATCGGAAGAGGCTCTTGGTGAGAACTTTGGAACAGCTTATATAAAGAAGGGCAAAGGAAAAGGAACCAAAGTTGGCCTTGGGGTAAAGCAGCACAAATTTCACATGATGTATGGTTTTGATCCTCAAGAGTATAACCTGATCCGTTTTGTCGACCCTCTTACAGGTGCAACATTAGATGAACAAATCCATGCTGATATTCGCTTAGTGCAAGAACACTTTGACATAATCCGAGAAGAGGCAGTTGCAAACGACACAATTGAACGACAACACATATACGGAAATCCTGGGCTCCAAGCATTCTTTATACAGAATGGATCAGCTAATGCATTAAGAGTTGATTTAACACCACATTCACCTTTGCGTGTTGTGACGAACAACAACATAGCAGGGTTTCCAGAGTATGAGGGCACATTACGACAAACTGGCACAGCTCTCACTGTACCTGTAAATCAAGTACCAGCAGCAAACGAGACAGGAGTCGCCCATGAATCCAAATCCATGATGTCTGGACTGGGCGATTATACACCTATTTCTCAGCAGCTCTGTCTCGTACAAAATGATTCTGAAGGAGTTAAACGTAATGTTTACGCAATTGGATACGGATCATATTTAATATCACCGGCGCATCTTTTCAAGTATAATAATGGTGAAATCACAATTAAATCCTCAAGAGGGCTGTATAAAATTAGAAATTCAGTCGATGTCAAATTGCACCCGATTGCACAGAGAGATATGGTCATAATTCAACTTCCAAAAGATTTCCCACCGTTCCCAATGCGACTTAAGTTCTCAACTCCGTCAAGAGATGTGCGTGTATGCTTAGTTGGAATCAATTTTCAACAGAATCACACCACGTGCATAATATCCGAAAGCAGTGTGACAGCACCCAAAGGAAATGGTGACTTCTGGAAACATTGGATTTCAACAGTTGATGGGCAATGTGGACTACCACTGGTTGATGTTAAGAATAAGCACATTGTTGGAATCCACAGCCTAGCCTCAACAAGTGGAAACACAAACTTCTTCGTCGCAATGCCCGAAAACTTCAATGAATATATATCTAATCTTGTGCAAACGAATAAGTGGGAAAGGGGATGGCACTACAACCCAAATCTCATTTCATGGTGTGGTTTGAACCTAGTCGATTCAGCACCCAGAGGATTATTCAAAACGTCAAAGCTTGTTGAAGATCTAGATATGAGTGTTGAAGAACAATGCAAGGTGACAGAAACATGGTTGACGGAACACATCCAAGATAACCTGCAGGTCGTCGCAAAGTGTCCAGGCCAACTCGTAACAAAGCATGTTGTCAAAGGCCCATGTCCACACTTCCAACTATATTTATCTACACACGATGAAGCAAAGTCGTATTTTTCACCATTGCTTGGAAAGTATGACAAGAGTAGGTTGAACAGGGCAGCATTTATCAAAGATCTCTCAAAGTATGCAAAGCCGATTTATATTGGAGAGATCAATTATGAAATCTTTGATAAAGCAGTTAAACGGGTTATAAGCATCCTCAGAAGTGTAGGAATGCTACAGTGTACGTACGTGACGGACGAAGAAGAAATTTTCAATTCGTTAAACATGAACGCAGCCGTAGGTGCACTCTACACAGGAAAGAAGAAAGACCTTTTCAAAGATTTCTCGAACGATGACAAAGCCGAAATCATCATGCGATCATGTGAGCGCATCTACAATGGACAATTGGGTGTATGGAACGGTTCACTCAAAGCTGAAATACGACCAATAGAGAAAATCATACTAAACAAGACACGCACTTTCACAGCAGCGCCATTAGAAACTCTACTTGGTGGGAAAGTATGTGTGGACGATTTTAACAATCAATTCTATTCACACCATCTTGAAGGCCCGTGGACCGTAGGAATCACAAAGTTTTACGGAGGATGGAACCGACTTTTGGAGAAATTGCCAGAAGGATGGATTTATTGCGATGCAGATGGATCCCAGTTTGACAGCTCACTAACTCCATATCTTATTAATGCTGTGCTGCATATTCGTTTACATTTCATGGAAGAATGGGAGTTGGGAGCCCAAATGTTGCGAAATTTATACACAGAGATTGTTTACACGCCAATCGCAACACCTGATGGATCTATCATCAAGAAGTTTAAAGGAAATAATAGTGGGCAACCATCTACAGTCGTTGACAACACGCTCATGGTTATTATAGCATTTAATTATGCAATGTTATCAAGTGGCATTCCTGAAGACAAAATTGACGACTGCTGTAGAATGTTTGCAAACGGTGACGACTTACTTTTGGCAGTGCATCCGGATTACGAATATATATTGGACGGATTTCAAAATCACTTTGGAAACCTCGGCCTTAATTTCGAGTTCACATCGAGGACAAAGGACAAGTCAGAGTTATGGTTCATGTCAACACAAGGAGTCAAGTGTGAAGGTATCTACATACCAAAACTCGAAAGGGAAAGAATAGTCGCAATCCTTGAATGGGACCGATCGAACTTGCCTGAGCATCGTCTTGAAGCTATCTGTGCAGCCATGGTTGAAGCATGGGGTTACTCAGATTTAGTTCATGAAATTCGAAAGTTTTACGCGTGGCTTCTCGAAATGCAACCCTTCGCGAACCTGGCAAAGGAAGGCATGGCGCCATACATAGCAGAAACAGCACTCCGCAACCTTTACCTTGGAACAGGCATCAAAGAAGAAGAAATTGAAAAATATTTTAAGCAGTTTGTCAAGGATCTTCCTGGATACGTAGAAGATTACAATGAAGAAGTTATTCATCAATCTGGCCATGTTGATGCAGGGAGACAAGGCGGTAGCGGTGCTCAAGGAGGCACACCACCAGCAGGA---------------------------------------------------AGTGGAGGCAC----TGGATCTGGCACTCAGGGCAATGGGGGTCAGACGGGA---------TCCCAAG---GAAGTGGTGGTCAACAAGGGTCCGGTGGGGGCACTGGCCAAGGAGCAGCTGGAAGCAACGGTGG------------AGGCCAGACAGGAGGCTCTAGCGGG------------ACAGCTGGTCAAAGAGATAAGGATGTTGACGCAGGCTCGGCTGGAAAGATATCCGTACCAAAGCTTAAAGCCATGTCAAAGAAAATGCGCTTGCCAAAGGCAAAAGGAAAAGATGTCTTGCATCTGGACTTTTTGTTGACATACAAGCCACAACAGCAAGACATATCGAACACAAGAGCAACTAAGGAAGAGTTCGATAGATGGTACGACGCCATAAAGAAGGAGTACGAGATCGATGATACACAAATGACAGTTGTCATGAGTGGTCTGATGGTCTGGTGCATCGAGAATGGTTGCTCACCAAATATAAACGGGAATTGGACGATGATGGATGGAGAAGAACAACGAGTTTTTCCATTAAAACCAGTCATTGAAAACGCATCTCCAACCTTCCGACAAATAATGCACCACTTTAGTGATGCAGCTGAAGCGTACATAGAGTATAGAAACTCTACAGAGCGATATATGCCAAGATACGGACTTCAGCGAAATCTCACCGACTGTAGCCTAGCACGGTATGCATTTGATTTCTATGAAATGACCTCACGCACACCAGCTAGAGCTAAGGAAGCCCACATGCAG---ATGAAAGCCGCAGCAGTTCGTGGTTCAAACACACGACTGTTCGGTCTGGACGGAAATGTCGGCGAGACTCAGGAGAATACAGAGAGACACACAGCTGGTGACGTTAGTCGCAACATGCACTCTCTGTTGGGAGTGCAGCAGCACCACTAGTCTCCTGGAAACCCTGTTTGCAGTACCTATAATATGTACTA------ATATATAGTATGTTGGTGAGGCTATGCCTC-------------------------GTTTGCTATTTTATTACGTACGTATTTACAGCGTGAACCAGTCTGCAGGACACAGGGTTGGACCCAGTGTCTTCTGGTGTAGCGTGTACTAGCGTCGAGCCACGTGACGGACAGCACTGGGTGTGGCTTTACCATTGGTGCTGCGAGTCTCTTGGTGAGAGAC----------------

>JX047413

---------------------AAAACAACAAAACTCAACAACACACAACAAAACACAACCAAACAAAACCAAGTTTTCTTTGCTCAGATTGTAGTGAACGGCTCGCAAGAAACGGTTCTTCGAGATCACTCTCTGACTTCTTTCTC----------TCTCACACTTGCATTCAAGCGGAATGGCGGGATCGTGGACTCACGTATCTTACAAGTGGCAGCCAAATGTTAACAATGACCGTGATGTAAGAAAGGTAATGGAAATGTTTGCAGCAAAACATCAACATTACACAGAGGAGCAGCGACTTGCACATAACAGCAAGTTGCTAAGGAAGGCGTATGTTGTGGACGTTGAACCAGTGAAGCCAGCACCGGAGCCTATAAGGCGTAAAGTGTGGGTGGAAAAATTCGACCACAACCCAACCGAAGACCTGGTGTATCCGCGCCTT---GTCACGTTCAAAAAGGCAACAGAAATGAAGCCCGTGAACACCTCTATAAATAAACTTATAAGGGATGTTCTGGACATATCAAAAGGAAGCAGCCTTAAACTTGAGCTGATTGGTAAACGCCAGAAGTGCAAAACACAATTAGCTATTAAGAAATACAACAATAAGGACTACCTCCATTGCAGGACACGTCATGAAGACAACATGTTCAAGAGGAGGGACGTGGCAATCGGCATTGAATGGATCCCAACCATTGAAGCGATTGCTCGATGCTACAGCACAGTAAACAAACAGGAGATGCAAAGTCTCTACAAAGGCAGTAGTGGCTTAACATTCATGCAAAATGATGAATTATTCATTGTTAGAGGCAGAATGAATGGCGAGTTAGTTAATAGCCTGGAAGAGAACAGGAACGTGCTCGACATCGATCACTACGCTGATCCACAAGCGAACGACTTTTGGAAAGGGTACACTGATGCATACGTTGCGAATCGCAGTATCTCCACTACACATACCGTGCATACTCCAACAATCAACTTGGTAGAGTGTGGAAAGAGAATGGCATTACTTGAAATTCTATTCCACTCAACTTTTAAGATTACATGCAAGACATGCAATATTGATGACTTGGAGTTATCAGACGATGAATTTGGAGCAAAACTTTTCAAGAATCTACAACGTATCGAGGAACGGCAACGCGAATATCTCGCTAAAGATCAAAAGCTGCGTCGCATGATTCAATTCATCAAAGAAAGGTGTAATCCAAAGTTTTCTCATCTACCATTGCTTTGGCAAGTTGCAGAAACAATAGGACATTATACAGACAATCAATCAAAGCAAATAATTGATATCAGTGAAGCACTCATCAAAGTGAACACATTAACACCTGATGATGCAGTCAAGGCGAGCGTAGCACTGCTTGAGGTGGCACGATGGTATAAAAATAGGAAAGAATCACTTAAAACAGACACATTAGATTCGTTCCGAAACAAGATATCCCCGAAGAGCACGATAAACACAGCATTGATGTGTGACAATCAACTAGACAAGAATGCAAATTTTGTCTGGGGAAATAGGGAGTATCATGCAAAGCGTTTCTTCTCAAATTACTTTGAAGCAGTAGATCCTACTGACGCATACGAGAAGCACGTAACCCGTTTCAATCCAAATGGTCAACGGAAATTATCAATTGGTAAACTAGTAATTCCGTTAGACTTCCAGAAAATAAGAGACTCATTCGTTGGTTTGGCAATAAACAAACAGCCACTCACAAAGGCTTGTGTGAGCAAAATCGATGGTGGTTATGTATACCCATGCTGTTGTGTCACAACAGAATTTGGAAAACCAGCATACTCTGAGATAATACCACCAACAAAGGGCCATATAACAATTGGAAATTCTGTAGACCCAAAAATCGTAGACCTGCCAAACACAACACCACCAAGTATGTACATTGCCAAAGACGGATATTGTTACATCAACATTTTCTTAGCAGCAATGATAAATGTCAATGAAGACTCAGCAAAGGACTACACAAAATTCCTCAGGGATGAATTAGTTGAGCGACTTGGAAAGTGGCCGAAACTTAAAGATGTTGCAACAGCGTGCTATGCGTTATCAGTTATGTTTCCAGAGATTAAGAACGCTGAATTGCCTCCAATACTCGTTGACCACGAAAGCAAGTCAATGCACGTGATCGATTCATACGGCTCATTGAGCGTTGGATTCCACATCCTTAAAGCAAGCACTGTTGGCCAGCTCATAAAATTTCAATACGAGTCAATGGATAGTGAGATGCGCGAGTATATAGTGGGAGGTACTTTAACGACCCAAACATTTAACAAACTCCTTACATCTTTAGCTAAAAATATGTTCAAACCAGATCAAATTAAGCGGATGATTGAGGAGGAACCCTTCCTTCTAATGATGGCAATCGCGTCACCAACTATGCTCATAGCGCTATACAATAATTGCTATATAGAGCAGGCTATGACGTATTGGATCGTTAAAAACCAAGGAGTGGCAGCGATATTTGCACAACTGGAAGCACTAGCAAAGAAGACCTCACAAGCTGAATTGCTAGTCCAACAAATGCAAATACTTGAAAAGGCGTCTAATCAATTGAGGCTTGCAGTCACGGGTTTAAATCATGTCGATCCAGCTAAGCGTCTGCTATGGTCGCATCTAGAAGCAATGACAACACGATCAGAGATGAACAAGGAATTAATTGCTGGAGGCTATGCCCTGTATGATGAGCGCCTTTATGCACTTATGGAAAAAAGTTACGTAGATCAATTAAACCAATCATGGGCAGAGTTATCATTCTGTGGAAAATTTTCAGCAATATGGCGTGTGTTCAAAGTCAAGAAATATTACAAGCCGTCTTTAACCGTGAGAAAAAGCGTAGATTTAGGCGCTGTATACAATATATCAGCTACGCATCTAATATCGGATTTAGCGCAGAGAAGTCGCGATCGAGCCAGCTCTATTTTAACCAAACTCCGCAACGGTTTTTATGATAAGTTAGAGAAGGCAAGAACTCGAGCAATTAAAACTGTTTATTGGTTCATACCTGACATATTTAGACTTATGCATATTTTCATAGTTCTTAGTTTATTAACAACTGTGGCAAACACTATTATTGTGACTATGAATGATTACAAAAAGCTGAAAAAGCAACAAAGAGAGGATGAGTATGAAGCTGAAATTAACGAGGTTCGAAAAATTCACGCTAATCTGATGAAAGAGCATAATGACAATCTAACATGTGAGCAGTTTATCGAACATATGCGCCAGATGCATCCACGATTGATTGAAGCCACATTGGAGTTGACACACACAGGCGTCATTCATGAAGGAAAGAGCAACCTAGAAACCAATCTCGAGCAAGCTATGGCAGTGGGCACTTTACTCACAATGATGTTAGACCCACAGAAGAGTGATGCAGTATACAAAGTGTTGAATAAGATGAGAACTGTAATTAGCACATTTGAGCAGAATGTCCCATTTCCTTCAATCAACTTTACAAATATTTTAACACCATCAGTGGCGCAACAAAGTGTAGATGTTGATGAACCTCTAACTTTAAGTACTGACAAGAATCTAACAATAGATTTTGATACTAATCAAGATTTACCAGCGGACACGTTTAGCAATGATGTTACTTTCGAAGATTGGTGGGCAAATCAAATAAGCAACAATAGAACAGTTCCACATTATCGGCTTGGTGGAAAATTTGTAGAATTCACAAGAGAAAATGCAGCACATGTTAGCATTGAGCTCGCACACTCAAATCTTGAGAAGGAGTTTCTACTCAGAGGAGCTGTTGGCTCAGGAAAATCCACTGGTCTTCCATATCATCTTAGTATGCGTGGTAAGGTGCTTTTAATAGAACCAACAAGACCACTAGCCGAGAATGTCTGCAGACAGCTTCAAGGACCACCATTCAATGTCAGCCCGACACTACAAATGCGTGGTTTAAGCTCTTTTGGTTGCACGCCAATTACGATAATGACATCAGGTTTCGCACTACACATGTATGCAAATAATCCGGATAAGATCTCTGACTACGATTTTATAATATTTGATGAGTGTCATATCATGGAAGCGCCAGCCATGGCGTTCTACTGTTTGTTGAAAGAATATGAATATAGAGGCAAAATCATTAAGGTGTCCGCAACACCACCAGGACGAGAGTGTGAGTTCTCTACTCAGCATCCAGTTGATATCCATGTGTGCGAAAATCTCACTCAGCAACAATTCGTTAGAGAGCTTGGTTCCGGATCAAACGTTGACGCAACAAAGTATGGAAACAACATACTTGTGTATGTTGCAAGCTACAACGACGTCGACTCATTAGCGCATGCTCTAACTGAATTACACTATTCAGTCATAAAGGTTGATGGGAGAACAATGAAACAGAACACTACAGGGATAGTGACAAATGGCACCTCTCAAAAGAAGTGTTTTGTCGTTGCTACGAATATCATTGAGAATGGTGTCACATTAGACGTTGATGTTGTGGTCGACTTCGGACTCAAAGTAACAGCTGAATTAGATGTTGACAACAGAGCTATTCTATACAAGCGTGTTAGCATTTCATATGGTGCGCGAATTCAAAGGCTAGGGCGTGTTGGAAGAAACAAACCCGGAACAGTCGTTCGAATTGGAAAAACCATGAAGGGTTTGCAAGAAATCCCAGCCATGATAGCGACAGAAGCAGCATTCATGTGCTTTGCATATGGACTAAAGGTCATAACACACAATGTTTCAACAACACACTTAGCAAAGTGCACAGTCAAGCAAGCGCGAACAATGATGCAATTTGAGCTTTCACCATTCGTAATGGCAGAACTAGTCAAATTTGATGGCTCAATGCACCCACAAATCCATGAAGCACTTGTGAAATATAAGCTTAGAGATTCTGTAATAATGCTCAGACCTAACGCAATTCCCAAAGTTAATTTTCATAACTGGCTGACAGCACGTGATTACAACAGGATGGGTTGTTCACTAGAGCTAGAGGATCATGTCAAAATTCCATATTACATACGAGGCGTCCCTGACAAGTTATACGGGAAATTGTATGATATCATTTTACAATATAGTCCAACCAGTTGTTACGGAAGACTATCAAGTGCTTGTGCTGGGAAGGTAGCATACACCTTGCGAACGGACCCTTGTTCGCTACCACGAACAATAGCAATAATCAATGCATTAATCACAGAGGAGTATGCTAAGAGAGATCATTACCGTAATATGATAGCAAATCCTTCGTCTTCGCATGCATTCTCACTAAATGGATTAGTCTCCATGATTGCTTCAAGATACATGAAGGACCACACAAAAGAGAATATAGACAAACTTATTAAGGTGCGGGATCAACTACTCGAATTTCAAGGCACTGGTATGCAATTTCAAGATCCATCAGAACTAATGGACATTGGCGCCTTGAACACAGTTATACATCAAGGAATGGATGCAACAGCTGCATGCATTGGTCTCCAAGGACGATGGAATGCATCACTCATACAACGTGATCTCATGATTGCAGGAGGAGTATTCATTGGAGGAATATTAATGATGTGGAGCTTGTTCACTAAGTGGGGAACGACCAATGTATCACACCAAGGAAAGAACAAGCGTAGCCGACAAAAATTGAAGTTCAAGCAAGCTAGAGACACTAAATACGCATATGATGTGACAGGATCGGAAGAAACTCTTGGTGAAAACTTTGGAACAGCTTATACAAAGAAGGGCAAAGGAAAAGGAACCAAAGTTGGTCTTGGGGTGAAGCAGCATAAATTTCACATGATGTATGGTTTTGATCCTCAAGAGTACAACCTAATTCGTTTTGTCGACCCTCTTACAGGTGCAACATTGGATGAACAAATCCATGCTGATATTCGCTTAGTGCAAGAACACTTTGACATAATCCGAGAGGAGGCAGTCGCAAACGACACAATCGAGCGACAACACATATACGGAAATCCTGGTCTCCAAGCATTCTTCATACAGAATGGATCAGCTAATGCATTAAGAGTTGATTTAACACCACATTCACCTTTGCGTGTTGTGACGAACAACAACATAGCAGGATTTCCAGAATATGAGGACACATTACGACAAACTGGCACAGCTCTCACTGTACCTGTGAATCAAGTACCAGCAGCAAATGAGACAGGAGTCGCCCACGAATCCAAATCTATGATGGCTGGATTGGGCGATTACACACCTATCTCTCAGCAGCTCTGTCTCGTACAAAACGATTCTGAAGGAGTCAAACGTAATGTGTATGCAATTGGATATGGATCATATTTAATATCGCCGGCGCACCTTTTCAAGTATAACAATGGTGAAATCACAATTAAATCCTCAAGAGGGCTGTATAAAATTAGAAATTCAGTCGATGTCAAGTTGCACCCGATTGCACAGAGGGACATGGTCATAATTCAACTTCCAAAAGACTTCCCGCCGTTCCCAATGCGACTTAAGTTCTCAACTCCGTCAAGAGATGTGCGTGTGTGCTTAGTTGGAATCAACTTTCAACAGAATCATACCACGTGCATAATATCCGAGAGCAGTGTGACAGCACCCAAAGGAAATGGTGATTTCTGGAAACATTGGATTTCAACTGTTGATGGGCAATGTGGGCTACCGTTAGTTGACGTTAAGAATAAACACATTGTCGGAATTCACAGCCTGGCCTCAACAAGTGGAAATACGAACTTTTTCGTTGCAATGCCTGAGAACTTCAATGAATATATATCTAATCTCGTGCAAACGAATAAGTGGGAAAAGGGATGGCATTATAACCCAAATCTTACTTCATGGTGTGGTCTAAACCTGGTTGATTCAGCACCTAAAGGATTGTTTAAAACATCAAAACTTGTTGAAGATTTGGATATGAGCGTTGAAGAACAATGCAAGGTGACAGAGACATGGTTGACGGAACACATCCAGGATAATCTACAGGTCGTTGCAAAGTGTCCAGGCCAACTTGTAACAAAGCATGTTGTTAAAGGCCCATGTCCGCATTTTCAACTGTATTTATCCACACATGATGAGGCAAAGTTGTACTTTTCACCTTTGCTTGGAAAGTATGACAAGAGTAGGTTGAACAGGGCAGCATTTATCAAAGATCTTTCAAAGTACGCAAAGCCGATTTATATTGGAGAGATCAATTATGAAATCTTTGATAAGGCAGTTGATCGAGTCATAAGCATCCTCAGAAGTGTAGGAATGCTACAGTGTACATACGTGACGGACGAAGAAGAAATTTTCAATTCATTAAATATGAACGCAGCCGTAGGTGCACTCTACACAGGAAAGAAGAAAGACTATTTCAAAGATTTCTCGAACGATGACAAAGCCGAAATCATCATGCGTTCATGTGAGCGCATCTATAATGGACAATTGGGCGTATGGAACGGTTCACTCAAAGCTGAAATACGACCAATAGAGAAAACCATACTAAACAAGACACGCACTTTCACAGCAGCGCCATTAGAAACTCTACTTGGTGGGAAAGTATGTGTGGACGATTTCAACAATCAATTTTATTCACATCATCTTGAAGGCCCATGGACCGTAGGAATCACAAAGTTTTATGGAGGATGGAACCGACTTTTGGAGAAATTGCCAGAAGGATGGATTTATTGCGATGCAGATGGATCCCAGTTTGACAGCTCACTAACTCCATATCTTATTAATGCTGTATTGCACATTCGCTTACATTTCATGGAAGAATGGGAGTTGGGAGCTCAGATGTTGCGAAATTTATACACAGAGATCGTTTATACGCCAATCGCAACGCCTGATGGGTCTGTCATCAAGAAATTCAAAGGAAATAATAGTGGGCAACCATCTACAGTCGTTGACAACACGCTTATGGTTATTATAGCATTTAATTACGCAATGTTATCAAGTGGCATTCCTGAAGACAAAATTGACGATTGCTGTAGAATGTTTGCAAACGGTGACGACTTACTCTTGGCAGTGCATCCGGATTACGAATATATATTGGACGGATTTCAAAATCATTTTGGAAACCTCGGCCTTAACTTTGAGTTTACATCGAGGACAAAGGACAAATCAGAATTATGGTTCATGTCAACACAAGGAGTCAAGTGTGAAGGTATCTACATACCAAAACTCGAAAGGGAAAGAATAGTCGCAATTCTTGAATGGGACCGATCGAACTTGCCTGAGCATCGTCTTGAAGCTATCTGTGCAGCCATGGTTGAAGCATGGGGTTACCCAGATTTAGTTCATGAAATTCGAAAGTTTTACGCGTGGCTTCTTGAAATGCAACCCTTCGCGAACCTGGCAAAGGAAGGCATGGCGCCATACATAGCAGAAACAGCACTCCGCAACCTCTACCTTGGAACAGGCATCAAAGAGGAAGAAATTGAAAAATATTTTAAGCAGTTTGTCAAGGATCTTCCTGGATACATAGAAGATTACAATGAAGAAGTTATTCATCAATCTGGTCAAGTTGACGCAGGGAGACAGGGCGGTAGCGGCACTCAAGGAGGCACACCACCAGCAGGA---------------------------------------------------AGTGGAGGCAC----TGGATCTGGCACTCAAGGCAATGGGGGTCAGACGGGA---------TCCCAAG---GAAGTGGTGGTCAACAAGGGTCCGGTGGGGGCACTGGTCAAGGAGCAGCCGGAAACAACGGCGG------------AGGTCAGACAGGGGGCTCTAGTGGG------------ACAGCTGGTCAGAGAGATAAGGACGTTGACGCAGGCTCGGCTGGAAAGATATCCGTACCAAAGCTTAAAGCCATGTCAAAGAAAATGCGCTTGCCAAAGGCAAAAGGAAAAGACGTCTTGCATTTGGACTTTTTGTTGACATACAAGCCACAACAGCAGGACATATCGAACACAAGAGCAACTAAGGAAGAGTTCGATAGATGGTACGACGCCATAAAGAAGGAGTACGAGATCGATGATACACAAATGACAGTCGTCATGAGTGGTCTGATGGTCTGGTGCATCGAAAATGGTTGCTCACCAAATATAAACGGAAATTGGACGATGATGGATGGGGATGAACAAAGAGTTTTCCCACTAAAACCAGTTATTGAAAACGCATCTCCAACTTTTCGACAAGTTATGCATCATTTCAGTGATGCAGCTGAAGCGTATATAGAATACAGAAATTCTACTGAGCGATACATGCCAAGATATGGACTTCAGCGAAATCTCACCGACTATAGCTTAGCGCGGTATGCTTTTGATTTCTATGAAATGACTTCACGCACACCAGCTAGAGCTAAGGAAGCCCACATGCAG---ATGAAAGCCGCAGCAGTTCGTGGTTCAAACACACGACTGTTCGGCTTGGACGGAAATGTCGGCGAGACTCAGGAGAATACAGAGAGACACACAGCTGGCGACGTTAGTCGCAATATGCACTCTCTGTTGGGAGTGCAGCAACACCACTAGTCTCCTGGAAACCCTGTTTGCAGTACCTATAATATATACTA------ATATATAGTACGTTGGTGAGGCTTTGCCTC-----------------------GGTTTTACTATCTTATTATGTATGTATTTACAGCGTGAACCAGTCTGCAGCATGCAGGGTTGGACCCAGCGTGTTCTGGTGTAGCGTGTACTAGCGTCGAGCCATGAGACGGACTGCACTGGGTGTGGCTATGCCACTTGTGTTGCGAGTTTCCTGGTAAGAGAC----------------

>JX047410

--------------------AAAAACAACAAAACTCAACAACACACAACAAAACACGACCAAACAAAACCAAGTTTTCTTTGCTCAGATTGTAGTGAACGGCTCGCAAGAAACGGTTCTTCGAGATCACTCTCTGACTTCTTTCTC----------TCTCACACTTGCATTCAAGCGGGATGGCGGGATCGTGGACTCACGTGTCTTACAAGTGGCAACCAAATGTCAACAACGACCGTGATGTAAGGAAGGTGATGGAGATGTTTGCAGCAAAACATCAACATTACACGGAGGAGCAGCGACTTGCACATAACAGCAAGTTGCTCAGGAAGGCGTACGTTGTGGACGTTGAACCAATGAAGCCAGCACCAGAACCCATAAGGCGTAAGGTGTGGGTGGAAAAATTTGACCATAACCCAACCGAAGACCTGGTGTATCCGCGCCTT---GTCACGGTTAAAAAGGCAGCAGAAATGAAGCCCGTGAACACCTCTATAAATAAACTCATAAGGGATGTTCTGAATATATCAAAAGGGAGTAGCCTTAAACTTGAGTTGATTGGCAAACGCCAGAAGTGCAAAACACAACTGGCTATAAAGAAATACAACAATAAGGACTACCTCCATTGCAGGACACGTCATGAGGGCAACATGTTCAAGAGGAGGGATGTGGCAATCGGCATTGAGTGGGTCCCAACCATTGAAGCGATTGCTCGATGCTACAGTACAGTAAACAAACAGGAGATGCAAAGTCTCTACAAAGGCAGTAGTGGCTTGACATTCATGCAAAATGATGAACTATTCATTGTTAGAGGCAGAATGAATGGCGAATTAGTTAATAGTCTGGAAGAGAACAGAAATGTGCTCGACATCGATCATTACGCTGATCCACAAGCGAACGACTTTTGGAGAGGGTACACTGATGCATATGTTGCGAATCGAAATATTTCCACTACACACACCGAGCACACTCCAACAATCAATTTGGAAGAGTGTGGAAAGAGAATGGCATTACTTGAAATCTTATTTCACTCAACTTTTAAGATTACATGCAAGACATGCAATATTGATGATTTGGAGTTATCAGACGATGAATTTGGAGCAAAACTTTTCAAGAATTTACAACGCATCGAAGAACAGCAACGCGAATACCTCGCTAAAGACCAAAAACTTCGTCGCATGATTCAATTCATCAAGGAGAGGTGTAATCCAAAGTTCTCTCATCTACCATTGCTTTGGCAAGTTGCAGAAACAATAGGACATTACACGGACAACCAGTCGAAGCAAATAATTGATATCAGTGAAGCACTCATCAAAGTGTACACATTAACACCTGATGATGCAGTCAAAGCGAGCGTAGCACTGCTTGAGGTGGCACGATGGTACAAAAATAGGAAAGAATCACTTAAAACAGACACATTAGATTCATTCCGAAACAAGATATCCCCAAAGAGCACGATAAACACAGCATTGATGTGTGATAATCAACTGGACAAGAATGCAAATTTTGTCTGGGGAAACAGAGAATACCATGCAAAGCGCTTCTTCTCAAATTACTTTGAAGCAGTAGATCCTACTGACGCATATGAGAAGCATGTAACCCGTTTCAATCCAAATGGTCAACGGAAATTATCAATTGGCAAACTAGTAATTCCGTTAGATTTTCAGAAAATAAGAGACTCATTCGTTGGTTTGTCAATTAACAAACAGCCACTTACAAAGGCTTGTGTTAGCAAAATCGATGGTGGTTATGTATACCCATGCTGTTGTGTTACAACAGAGTTTGGAAAACCAGCTTACTCTGAGATAATACCACCAACAAAGGGTCATATAACAATTGGAAACTCTGTAGATCCAAAGATCGTAGACCTGCCAAACACAACACCACCAAGTATGTACATTGCCAAAGACGGATACTGTTACATTAACATTTTCTTGGCAGCAATGATAAATGTCAATGAGGACTCAGCAAAGGACTACACAAAATTCCTCAGGGATGAATTAGTTGAGCGACTTGGAAAGTGGCCGAAACTTAAAGACGTTGCAACAGCGTGCTATGCGTTATCAGTTATGTTTCCAGAGATTAAGAACGCTGAATTACCTCCAATACTCGTTGACCACGAAAGCAAGTCAATGCACGTGATCGATTCATACGGCTCATTGAGCGTTGGATTCCACATCCTTAAAGCAAGCACCGTTGGCCAGCTCATAAAATTTCAATACGAGTCAATGGATAGTGAGATGCGCGGGTATATAGTGGGAGGTACTTTAACGACCCAAACATTTAACAAACTCCTTACATCTTTAGCTAAAAATATGTTCAAACCAGATCAAATTAAGCAGATGATTGAGGAGGAACCCTTCCTACTAATGATGGCAATCGCATCACCAACTATGCTCATAGCGCTGTATAATAATTGCTATATAGAGCAGGCTATGACGTATTGGATCGTTAAAAATCAAGGAGTGGCAGCGATATTCGCACAACTGGAAGCATTAGCAAAGAAAACCTCACAAGCCGAATTGCTGGTCCAACAAATGCAAATACTCGAAAAGGCGTCTAACCAGTTGAGGCTTGCAGTCACGGGTTTAAATCATGTCGATCCAGCCAAGCGTCTGCTATGGTCGCATCTAGAAGCAATGACAACACGATCAGAGATGAATAAGGAATTAATTGCTGAAGGCTATGCCCTGTATGATGAGCGCTTGTACGCACTTATGGAAAAAAGTTACGTAGATCAATTAAACCAATCATGGGCAGAGTTATCATATTGGGAAAAATTTTCAGCAATATGGCGTGTGTTCAAAGTCAAGAAATATTACAAGCCGTCTTTAACCGTGAGAAAAAGCGTAGATTTAGGCGCTGTATACAATATATCAGCTACGCATCTAATATCAGATTTAGCGCAGAGAAGTCGCGATCGAGTCAGCTCTACTTTAACCAAACTCCGCAACGGTTTTTATGATAAGTTAGAGAAGGCTAGAACTCGAGCAATTAAAACTGTTTATTGGTTCATACCTGACATATTTAGACTTATGCACATTTTTATAGTTCTTAGTTTACTAACGACTGTGGCTAACTCTATTATTGTAACTATGAATGATTACAAGAAGTTGAAAAAGCAACAAAGAGAGGATGAGTATGAAGCTGAGATTAACGAAGTTCGGAAAATTCATGCTAATCTGATGAAAGAGAACAATGACAATTTAACATGTGAACAGTTTGTCGAACATATGCGTCAGACGCATCCACGATTGATTGAAGCCACATTGGAATTGACACACACAGGCGTCATTCATGAAGGAAAGAGCAACCTAGAAACCAACCTCGAACAAGCTATGGCAGTGGGCACTTTACTCACAATGATGTTAGACCCACAAAAGAGTGACGCAGTATACAAAGTGTTGAATAAGATGAGAACTGTAATTAGTACATTTGAGCAGAATGTCCCGTTTCCTTCAATCAACTTTACAAATATTTTAACACCATCAGTGGCGCAACAAAGTGTGGATGTTGATGAACCTCTAACTTTAAGTACTGACAAGAATCTAACAATAGATTTTGATACTAATCAAGATTTACCAGCGGATACGTTTAGCAATGATGTCACTTTCGAAGATTGGTGGGCAAATCAAATAAGCAACAATAGAACAGTTCCACATTATCGACTTGGTGGCAAATTTGTAGAATTTACGAGAGAAAATGCAGCGCATGTCAGCATTGAGCTCGCACATTCAAATCTCGAGAAGGAATTCCTACTCAGAGGGGCTGTCGGCTCAGGAAAATCCACTGGTCTTCCATATCATCTTAGTATGCGTGGCAAAGTACTTTTAATAGAACCAACAAGACCACTTGCCGAGAATGTCTGCAGACAGCTTCAAGGACCACCATTCAATGTTAGCCCGACGCTACAAATGCGTGGTTTAAGCTCTTTTGGTTGCACGCCAATTACAATAATGACATCAGGTTTCGCGCTGCACATGTATGCAAACAATCCGGACAAGATCTCTGACTATGATTTTATAATATTTGATGAGTGTCATATCATGGAAGCGCCAGCCATGGCGTTCTACTGTTTGTTGAAAGAATATGAATACAGAGGCAAGATCATTAAGGTGTCCGCAACACCACCAGGACGAGAGTGCGAATTCTCTACTCAGCATCCAGTTGATATCCATGTGTGCGAAAATCTCACCCAGCAGCAATTCGTCAGAGAGCTCGGCTCCGGATCAAACGTTGACGCAACAAAGTATGGAAACAACATACTTGTGTATGTTGCCAGCTACAACGATGTTGACTCATTAGCGCATGCTTTAACTGAACTACATTATTCAGTTATAAAGGTCGATGGGAGAACAATGAAACAGAACACCACAGGAATTGTAACAAATGGCACCTCTCAAAAGAAGTGTTTTGTTGTTGCTACGAATATCATTGAGAACGGTGTCACATTGGATGTGGATGTCGTAGTTGATTTCGGACTTAAGGTTACAGCTGAACTAGATGTTGACAACAGAGCTATACTGTACAAGCGCGTTAGCATTTCATATGGTGAGCGAATTCAAAGGCTAGGGCGTGTTGGAAGGAATAAACCCGGAACAGTCGTTCGAATTGGAAAAACCATGAAGGGTTTGCAAGAGATCCCAGCCATGATAGCGACAGAAGCAGCATTCATGTGCTTCGCATACGGGTTAAAAGTTATAACGCACAATGTTTCAACAACACACTTAGCAAAGTGCACAGTAAAACAAGCGCGAACAATGATGCAATTTGAGCTCTCGCCATTTGTGATGGCAGAACTAGTCAAATTTGATGGCTCAATGCATCCGCAAATCCACGAAGCACTCGTGAAATATAAGCTTAGGGATTCTGTAATAATGCTCAGACCTAATGCAATTCCCAAAGTTAATTTTCACAATTGGCTGACAGCACGTGACTACAACAGAATGGGTTGCTCACTAGAGCTAGAGGATCATGTCAAGATTCCATATTACATACGAGGCGTCCCTGACAAGTTGTACGGGAAGTTGTATGATACCATTCTACAGTACAGTCCAACCAGTTGCTATGGAAGATTATCAAGTGCCTGTGCTGGGAAAATAGCATACACCTTGCGAACCGATCCTTGTTCGCTACCACGAACAATAGCAATAATCAATGCATTAATCACAGAAGAATATGCGAAGAGGGATCATTACCGCAACATGATAGCAAATCCTTCGTCCTCGCATGCATTCTCACTAAATGGATTAGTCTCTATGATTGCTTCAAGATATATGAAAGACCACACAAAGGAGAATATAGACAAACTTATTAAAGTGCGAGACCAATTACTCGAATTTCAAGGCATGGGTATGCAATTTCAAGACCCATCAGAACTAATGGATATTGGTGCCTTGAACACAGTTATACATCAAGGAATGGATGCAATAGCAGCATGTATTGGACTCCAAGGACGATGGAATGCATCGCTCATACAACGCGATCTCATGATTGCAGGAGGAGTATTCATTGGAGGTATCCTGATGATGTGGAGCTTATTCACTAAGTGGGGAACAACCAATGTATCACACCAAGGAAAGAACAAGCGTAGTCGACAAAAATTGAAATTCAAGCAAGCCAGAGACACTAAATACGCATATGATGTGACAGGATCGGAAGAGGCTCTTGGTGAGAACTTTGGAACAGCTTATACAAAGAAGGGCAAAGGAAAAGGAACCAAAGTTGGCCTTGGGGTAAAGCAGCACAAATTTCACATGATGTATGGTTTTGATCCTCAAGAGTATAACCTGATCCGTTTTGTCGACCCTCTCACAGGTGCAACATTAGATGAACAAATCCATGCTGATATTCGCTTAGTGCAAGAACATTTTGACATAATCCGAGAAGAGGCAATTGCAAACGACACAATTGAACGACAACACGTATACGGAAATCCTGGGCTCCAAGCATTCTTTATACAGAATGGATCAGCTAATGCATTAAGAGTTGATTTAACACCACATTCACCTTTGCGTGTTGTGACGAACAACAACGTAGCAGGGTTTCCAGGGTATGAGGGCACATTACGACAAACTGGCACAGCTCTCACTGTACCTGTAAATCAAGTACCAGCAGCAAACGAGACAGGAGTCGCCCATGAATCCAAATCCATGATGTCTGGACTGGGCGATTATACACCTATTTCTCAGCAGCTCTGTCTCGTACAAAATGATTCTGGAGGAGTTAAACGTAATGTTTACGCAATTGGATACGGATCATATTTAATATCACCGGCGCATCTTTTCAAGTATAATAATGGTGAAATCACAATTAAATCCTCAAGAGGGCTGTATAAAATTAGAAATTCAGTCGATGTCAAATTGCACCCGATTGCACAGAGAGATATGGTCATAATTCAACTTCCAAAAGATTTCCCACCGTTCCCAATGCGACTTAAGTTCTCAACTCCGTCAAGAGATGTGCGTGTATGCTTAGTTGGAATCAATTTTCAACAGAATCACACCACGTGCATAATATCCGAAAGCAGTGTGACAGCACCCAAAGGAAATGGTGACTTCTGGAAACATTGGATTTCAACAGTTGATGGGCAATGTGGACTACCACTGGTTGATGTTAAGAATAAGCACATTGTTGGAATCCACAGCCTAGCCTCAACAAGTGGAAACACAAACTTCTTCGTCGCAATGCCCGAAAACTTCAATGAATATATATCTAATCTTGTGCAAACGAATAAATGGGAAAGGGGATGGCACTACAACCCAAATCTCATTTCATGGTGTGGTTTGAACCTAGTCGATTCAGCACCTAGAGGATTATTCAAAACGTCAAAGCTTGTTGAAGATCTAGATATGAGTGTTGAAGAACAATGCAAGGTGACAGAAACATGGTTGACGGAACACATCCAAGATAACCTGCAGGTCGTCGCAAAGTGTCCAGGCCAACTCGTAACAAAGCATGTTGTCAAAGGCCCATGTCCACACTTCCAACTATATTTATCTACACACGATGAAGCAAAGTCGTATTTTTCACCATTGCTTGGAAAGTATGACAAGAGTAGGTTGAACAGGGCAGCATTTATCAAAGATCTCTCAAAGTATGCAAAGCCGATTTATATTGGAGAGATCAATTATGAAATCTTTGATAAAGCAGTTGAACGGGTTATAAGCATCCTTAGAAGTGTAGGAATGCTACAGTGTACGTACGTGACGGACGAAGAAGAAATTTTCAATTCGTTAAACATGAACGCAGCCGTAGGTGCACTCTACACAGGAAAGAAGAAAGACTTTTTCAAAGATTTCTCGAACGATGACAAAGCCGAAATCATCATGCGATCATGTGAGCGCATCTACAATGGACAATTGGGTGTATGGAACGGTTCACTCAAAGCTGAAATACGACCAATAGAGAAAACCATACTAAACAAGACACGCACTTTCACAGCAGCGCCATTAGAAACTCTACTTGGTGGGAAAGTATGTGTGGACGATTTTAACAATCAATTCTATTCACACCATCTTGAAGGCCCGTGGACCGTAGGAATCACAAAGTTTTACGGAGGATGGAACCGACTTTTGGAGAAATTGCCAGAAGGATGGATTTATTGCGATGCAGATGGATCCCAGTTTGACAGCTCACTAACTCCATATCTTATTAATGCTGTGCTGCATATTCGTTTACATTTCATGGAAGAATGGGAGTTGGGAGCCCAAATGTTGCGAAATTTATACACAGAGATTGTTTACACGCCAATCGCAACACCTGATGGATCTGTCATCAAGAAGTTTAAAGGAAATAATAGTGGGCAACCATCTACAGTCGTTGACAACACGCTCATGGTTATTATAGCATTTAATTATGCAATGTTATCAAGTGGCATTCCTGAAGACAAAATTGACGACTGCTGTAGAATGTTTGCAAACGGTGACGACTTACTTTTGGCAGTGCATCCGGATTACGAATATATATTGGACGGATTCCAAAATCACTTTGGAAACCTCGGCCTTAATTTCGAGTTCACATCGAGGACAAAGGACAAGTCAGAGTTATGGTTCATGTCAACACAAGGAGTCAAGTGTGAAGGTATCTACATACCAAAACTCGAAAGGGAAAGAATAGTCGCAATCCTTGAATGGGACCGATCGAACTTGCCTGAGCATCGTCTTGAAGCTATCTGTGCAGCCATGGTTGAAGCATGGGGTTACTCAGATTTAATTCATGAAATTCGAAAGTTTTACGCGTGGCTTCTCGAAATGCAACCCTTCGCGAACCTGGCAAAGGAAGGCATGGCGCCATACATAGCAGAAACAGCACTCCGCAACCTTTACCTTGGAACAGGCATCAAAGAAGAAGAAATTGAAAAATATTTTAAGCAGTTTGTCAAGGATCTTCCTGGATACGTAGAAGATTACAATGAAGAAGTTATTCATCAATCTGGCCATGTTGATGCAGGGAGACAAGGCGGTAGCGGTGCTCAAGGAGGCACACCACCAGCAGGA---------------------------------------------------AGCGGAGGCAC----TGGATCTGGCACTCAGGGCAATGGGGATCAGACGGGA---------TCCCAAG---GAAGTGGTGGTCAACAAGGGTCCGGTGGGGGCACTGGCCAAGGAGCAGCTGGAAGCAACGGCGG------------AGGCCAGACAGGAGGCTCTAGCGGG------------ACAGCTGGTCAAAGAGATAAGGATGTTGACGCAGGCTCGGCTGGAAAGATATCCGTACCAAAGCTTAAAGCCATGTCAAAGAAAATGCGCTTGCCAAAGGCAAAAGGAAAAGATGTCTTGCATCTGGACTTTTTGTTGACATACAAGCCACAACAGCAAGACATATCGAACACAAGAGCAACTAAGGAAGAGTTCGATAGATGGTACGACGCCATAAAGAAGGAGTACGAGATCGATGATACACAAATGACAGTTGTCATGAGTGGTCTGATGGTCTGGTGCATCGAGAATGGTTGCTCACCAAATATAAACGGGAATTGGACGATGATGGATGGAGAAGAACAACGAGTTTTTCCATTAAAACCAGTCATTGAAAACGCATCTCCAACTTTCCGACAAATAATGCACCACTTTAGTGATGCAGCTGAAGCGTACATAGAGTATAGAAACTCTACAGAGCGATATATGCCAAGATACGGACTTCAGCGAAATCTCACCGACTATAGCCTAGCACGGTATGCATTTGATTTCTATGAAATGACCTCACGCACACCAGCTAGAGCTAAGGAAGCCCACATGCAG---ATGAAAGCCGCAGCAGTTCGTGGTTCAAACACACGACTGTTCGGTCTGGACGGAAATGTCGGCGAGACTCAGGAGAATACAGAGAGACACACAGCTGGTGATGTTAGTCGCAACATGCACTCTCTGTTGGGAGTGCAGCAGCACCACTAGTCTCCTGGAAACCCTGTTTGCAGTACCTATAATATGTACTA------ATATATAGTATGTTGGTGAGGCTATGCCTC-----------------------GGTTTTGCTATTTTATTACGTACGTATTTACAGCGTGAACCAGTCTGCAGGACACAGGGTTGGACCCAGTGTCTTCTGGTGTAGCGTGTACTAGCGTCGAGCCACGTGACGGACAGCACTGGGTGTGGCTTTGCCATTGGTGCTGCGAGTCTCTTGGTGAGAGAC----------------

>JX047409

--------------------AAAAACAACAAAACTCAACAACACACAACAAAACACAACCAAACAAAACCAAGTTTTCTTTGTTCAGATTGTAGTGAACGGCTCGCAAGAAACGGTTCTTCGAGATCACTCTCTGACTTCTTTCTC----------TCTCACACTTGCATTCAAGCGGGATGGCGGGATCGTGGACTCACGTGTCTTACAAGTGGCAGCCAAATGTCAACAATGACCGTGATGTAAGAAAGGTAATGGAAATGTTTGCAGCAAAACATCAACATTACACGGAGGAGCAGCGACTTGCACATAACAGCAAGTTACTAAGGAAGGCGTATGTTGTGGACGTTGAACCAATGAAGCCAGCACCGGAGCCTATAAGGCGTAAGGTGTGGGTGGAAAAATTCGATCACAACCCAACCGACGACCTGGTGTATCCGCGCCTT---GTCACGGTTAAAAAGGCAGCAGAAACGAAGCCCGTGAACACCTCTATAAATAAACTTATAAGGGATGTTCTGGACATATCAAAAGGGAGCGGCCTTAAACTTGAGCTGATTGGTAAACGCCAGAAGTGCAAAACACAACTGGCTATTAAGAGATACAACAATAAGGACTACCTCCATTGCAGGACACGTCATGAAGATAACATGTTCAAGAGGAGGGATGTGGCAATCGGCATTGAATGGATCCCAACCATTGAAGCGATTGCTCGATGCTACAGCACAGTAAACAAACAGGAGATGCAAAGTCTCTACAAAGGCAGTAGTGGCTTAACATTCATGCAAAATGATGAATTGTTCATTGTTAGAGGCAGAATGAATGGCGAATTAGTTAATAGCCTGGAGGAGAACAGAAATGTACTCGATATCGACCACTACGCTGATCCACAAGCGAACGACTTTTGGAAAGGGTACACTGATGCATACGTTGCGAATCGCAATATCTCCACTACACATACCGTGCATACTCCAACAATCAACTTGGTAGAGTGTGGAAAGAGAATGGCATTACTTGAAATTCTATTCCACTCAACTTTTAAGATTACATGCAAAACATGCAATATTGATGACTTGGAGTTATCAGATGATGAATTTGGAGCAAAACTTTTCAAGAATCTACAACGCATCGAGGAACAGCAACGTGAATATCTCGCTAAAGATCAAAAGCTGCGTCGCATGATTCAATTCATTAAAGAAAGATGTAATCCAAAGTTCTCTCATCTGCCATTGCTTTGGCAAGTTGCAGAAACAATAGGACATTATACGGACAACCAGTCAAAGCAAATAATTGATATCAGTGAAGCACTCATCAAAGTGAACACGTTAACACCTGATGATGCAGTCAAGGCGAGCGTGGCACTGCTTGAGGTGGCACGATGGTATAAAAATAGGAAAGAATCACTTAAAACAGACACATTAGATTCGTTCCGAAACAAGATATCCCCAAAGAGCACGATAAACACAGCATTGATGTGTGACAATCAACTAGATAAGAATGCAAATTTTGTCTGGGGAAACAGGGAGTATCATGCAAAGCGTTTCTTCTCAAATTACTTTGAAGCAGTAGATCCTACTGACGCATATGAGAAGCACGTAACCCGTTTCAATCCAAATGGTCAACGGAAATTATCAATTGGCAAACTAGTAATTCCGTTAGATTTCCAGAAAATAAGAGACTCATTCGTTGGTTTGGCAATTAACAAACAGCCACTTACAAAGGCTTGTGTTAGTAAAATCGATGGTGGTTATGTATACCCATGCTGTTGTGTCACAACAGAATTTGGAAAACCAGCATACTCTGAGATAATACCACCAACAAAGGGCCATATAACAATTGGAAATTCTGTAGATCCAAAGATCGTAGACCTGCCAAATACAACACCACCAAGTATGTACATTGCCAAAGACGGATATTGTTACATCAACATTTTCTTGGCAGCAATGATAAATGTCAATGAAGATTCAGCAAAGGACTACACAAAATTCCTCAGGGATGAATTAGTTGAGCGACTTGGAAAGTGGCCGAAACTCAAAGACGTTGCAACAGCGTGTTATGCGTTATCAGTTATGTTTCCAGAGATTAAGAACGCTGAATTGCCTCCAATACTCGTTGATCATGAAAGCAAGTCAATGCACGTGATCGATTCATACGGCTCATTGAGCGTTGGATTCCACATCCTTAAAGCAGGCACTGTTGGCCAGCTCATAAAATTTCAATACGAGTCAATGGATAGTGAGATGCGCGAATATATAGTGGGAGGTACTTTGACGACCCAAACATTTAACAAACTCCTTACATCTTTAGCTAAAAATATGTTCAAACCAGATCAAATTAAGCAGATGATTGAGGAGGAACCCTTCCTACTAATGATGGCAATCGCGTCACCAACTATGCTCATAGCGCTATACAATAATTGCTATATAGAGCAGGCTATGACGTATTGGATCGTTAAGAACCAAGGAGTGGCAGCAATATTCGCACAACTGGAAGCACTAGCAAAGAAGACCTCACAAGCCGAATTGCTAGTCCAACAAATGCAAATACTTGAAAAAGCGTCTAGTCAACTGAGACTTGCAGTCACGGGCTTAAATCATGTCGATCCAGCCAAGCGTCTGCTATGGTCACATCTAGAAGCAATGACAACACGATCAGAAATGAATAAGGAATTAATTGCTGAAGGCTATGCCCTGTACGATGAACGCTTGTACGCACTTATGGAAAAAAGTTACGTAGATCAATTAAACCAATCATGGGCAGAGTTATCATTCTGTGGAAAATTTTCAGCAATATGGCGTGTGTTCAAAGTCAAGAAATATTACAAGCCATCTTTAACCGTGAGAGAAAGCGCAGATTTAGGCGCTGTGTACAATATATCAGCTACGCATCTAATATCAGATTTAGTGCAGAGAAGTCGCGATCGAGTCAGCTCTACTTTAACCAAACTCCGCAACGGTTTTTATGATAAGTTAGAGAAGGCTAGAACTCGAGCAATCAAAACTGTTTATTGGTTTATACCTGACATATTTAGACTTATGCATATTTTCATAGTTCTTAGTTTATTAACAACTGTGGCTAACACTATTATTGTGACTATGAATGACTACAAAAAGTTGAAAAAGCAACAAAGAGAAGATGAATATGAAGCTGAAATTAACGAGGTTCGAAAAATTCATGCCAATTTGATGAAAGAGCACAATGACAATTTAACATGTGAACAGTTCATCGAACATATGCGTCAGACGCATCCACGATTAATTGAAGCCACATTGGAGTTGACACACACAGGCGTCATTCATGAAGGAAAGAGCAACCTAGAAACCAATCTCGAGCAGGCTATGGCAGTGGGCACTTTACTCACAATGATTTTAGACCCACAAAAGAGTGACGCAGTATACAAAGTGTTGAATAAGATGAGAACTGTAATTAGTACATTTGAGCAGAATGTCCCATTTCCTTCAATCAACTTTACAAATATTTTAACACCATCAGTGGCGCAACAAAGTGTAGATGTTGATGAACCCCTAACTTTAAGCACTGACAAGAATCTAACAATAGATTTTGATACTAATCAAGATTTACCAGCAGATACGTTTAGCAATGATGTCACCTTCGAAGATTGGTGGGCAAATCAAATAAGCAACAATAGAACAGTTCCACACTATCGACTTGGTGGCAAGTTCGTAGAATTCACAAGAGAAAATGCAGCGCATGTCAGCATTGAACTCGCACACTCAAATCTTGAGAAGGAATTTCTACTCAGAGGAGCTGTTGGCTCAGGAAAATCCACTGGCCTTCCATATCATCTAAGTATGCGTGGCAAGGTACTTTTGATAGAACCAACAAGACCACTTGCCGAGAATGTCTGCAGACAGCTTCAAGGACCACCATTCAATGTCAGCCCGACGCTACGAATGCGTGGTTTAAGCTCTTTTGGTTGCACGCCAATCACAATAATGACATCAGGTTTCGCGCTGCACATGTATGCAAATAACCCGGATAAGATCTCTGATTATGATTTCATAATATTTGATGAGTGTCATATCATGGAAGCGCCAGCCATGGCGTTCTACTGCTTGTTGAAAGAATATGAATATAGAGGCAAAATCATTAAGGTGTCCGCAACACCACCAGGGCGAGAGTGCGAATTCTCTACTCAGCATCCAGTTGACATCCATGTGTGCGAAAATCTCACTCAGCAACAATTTGTTAAAGAGCTCGGTTCCGGATCGAACGTTGACGCAACAAAATATGGAAACAACATACTTGTGTATGTTGCAAGCTACAACGACGTCGACTCATTAGCGCATGCTTTAACTGAATTACATTATTCAGTCATAAAGGTTGATGGGAGAACAATGAAGCAGAACACCACAGGGATAGTAACAAATGGCACCTCTCAAAAGAAGTGTTTTGTCGTTGCTACGAATATCATTGAGAATGGTGTCACATTAGACGTCGATGTTGTAGTTGATTTCGGACTCAAAGTAACAGCTGAGTTAGATGTTGACAACAGAGCTATACTGTACAAACGCGTTAGCATTTCATACGGCGAGCGGATTCAAAGGCTAGGACGCGTTGGAAGGAACAAACCCGGAACAGTCGTTCGAATTGGAAAAACCATGAAGGGTTTGCAAGAAATCCCAGCCATGATAGCGACAGAAGCAGCATTCATGTGCTTTGCGTGTGGACTAAAGGTTATAACACACAATGTTTCAACAACACACTTAGCAAAGTGCACAGTCAAACAAGCGCGAACAATGATGCAATTTGAGCTCTCACCATTTGTGATGGCAGAACTAGTCAAATTTGATGGCTCAATGCATCCACAAATCCACGAAGCACTTGTGAAATATAAGCTTAGGGATTCTGTAATAATGCTCAGACCTAACGCAATTCCCAAAGTTAATTTTCACAACTGGCTGACAGCACGTGACTACAACAGGATGGGTTGTTCATTAGAACTAGAGGATCATGTCAAGATTCCATATTACATACGAGGCATCCCTGACAAGTTGTATGGAAAGTTGTATGATATCATTCTACAATATAGTCCAACCAGTTGTTATGGAAGATTATCAAGTGCTTGTGCTGGGAAGGTAGCATACACCTTGCGAACTGATCCTTGTTCGCTACCACGAACAATAGCAATAATCAATGCATTAATTACAGAAGAATATGCTAAGAGAGATCATTATCGAAATATGGTAGCAAACCCTTCGTCATCACATGCATTCTCTCTTAATGGATTGGTCTCCATGATTGCTTCAAGGTACATGAAAGATCACACAAAAGAGAATATAGATAAACTCATCAAGGTGCGAGATCAACTACTCGAATTTCAGGGCACGGGCATGCAGTTTCAAGACCCAGCAGAACTTATGGATATTGGCGCCTTGAACACAGTTATACATCAAGGAATGGATGCAACAGCCGCATGCATCGGACTTCAAGGAAGATGGAACGGCTCACTTATACAACGTGATCTTATGATAGCTGGAGGCGTGTTTATTGGAGGTATCTTAATGATGTGGAGTCTATTCACTAAGTGGGGCACAACCAATGTATCACACCAAGGAAAGAATAAACGTAGCCGACAAAAATTAAGATTCAAGCAAGCTAGAGATACTAAATATGCATACGATGTGACAGGATCGGAAGAAGTTCCTGGCGAAAACTTTGGAACAGCCTATACAAAGAAGGGCAAAGGAAAAGGAACTAAAGTTGGTCTTGGAGTGAAGCAGCACAAATTTCACATGATGTATGGTTTCGATCCCCAAGAATACAATCTGATCCGTTTTGTCGACCCTCTTACAGGTGCAACATTAGATGAACAAATCCATGCTGATATTCGCTTAGTGCAAGAACATTTTGACATAATCCGAGAGGAGGCAGTTGCAAACGACACAATTGAACGGCAACACATATATGGAAATCCCGGGCTCCAAGCATTCTTCATTCAGAATGGATCAGCCAATGCATTAAGAGTCGATTTAACACCACATTCACCTTTGCGTGTTGTGACAAACAACAACATAGCAGGGTTTCCAGAATACGAGGGTACATTACGACAAACTGGCACAGCTCTCACTGTACCTGTAAATCAAGTACCAGCAGCAAATGAGACAGGAGTTGCCCACGAGTCCAAATCTATGATGTCTGGATTGGGCGATTATACACCTATTTCTCAGCAGCTCTGCCTCGTACAAAATGACTCTGAAGGAGTTAAACGTAATGTGTATGCAATTGGATACGGATCATATTTGATATCACCGGCGCATCTTTTCAAGTACAACAATGGTGAAATCACAATTAAATCCTCAAGAGGGCTGTATAAAATTAGAAATTCAGTCGATGTCAAATTACACCCGATTGCACAGAGGGATATGGTTATAATTCAACTCCCAAAAGACTTCCCACCGTTCCCAATGCGACTTAAATTCTCAACTCCATCAAGGGATGTACGCGTATGCTTAGTCGGAATCAACTTTCAACAGAATCACACCACGTGCATAATATCCGAAAGCAGTGTAACAGCACCCAAAGGAGATGGTGATTTTTGGAAACATTGGATTTCAACGGTTGATGGACAATGTGGATTACCACTGGTCGATGTTAAGAATAAACACATTGTTGGAATCCACAGCCTGGCCTCAACGAGTGGAAACACAAACTTCTTCGTTGCAATGCCCGAAAACTTCAATGAATATATATCTAATCTCGTGCAAACGAATAAGTGGGAAAGGGGATGGCACTACAACCCAAATCTCATTTCATGGTGTGGTTTAAACCTAGTCGATTCAGCACCTAAAGGATTATTCAAAACGTCAAAGCTTGTTGAAGATCTAGATATGAGTGTTGAAGAGCAATGCAAGGTGACAGAAACATGGTTGACGGAACACATCCAAGATAACCTGCAGGTTGTCGCAAAGTGTCCAGGCCAACTCGTAACAAAACATGTTGTTAAAGGCCCATGTCCACACTTCCAACTATATTTATCTACACACGATGAAGCAAAGTTGTATTTTTCACCATTACTCGGAAAGTATGACAAGAGTAGGTTGAACAGAGCAGCATTTATCAAAGATCTCTCAAAGTACGCAAAACCTATTTACATTGGAGAGATCAATTACGAAATCTTTGATAAAGCAGTTGAGCGAGTTATAAGCATCCTCAGAAGTGTAGGAATGCAACAGTGCATGTACGTGACGGATGAGGAGGAAATTTTCAACTCATTAAACATGAACGCAGCTGTAGGTGCACTCTACACAGGAAAGAAGAAAGACTATTTCAAAGATTTCTCAAATGAGGAGAGAGCTGAAATCGTCATGCGATCATGTGAGCGCATCTACAACGGACAATTAGGTGTATGGAATGGGTCACTCAAAGCTGAAATACGACCTATAGAGAAAACCATACTGAACAAGACACGTACTTTCACAGCAGCACCACTAGAAACTCTACTTGGTGGAAAAGTATGTGTGGACGATTTCAATAATCAATTCTACTCACATCATCTTGAAGGCCCGTGGACCGTAGGAATCACAAAATTTTATGGAGGATGGAACCGACTTTTGGAGAAGTTGCCAGAGGGGTGGATTTACTGTGATGCAGATGGATCTCAGTTCGACAGCTCACTAACTCCATATCTTATTAATGCTGTACTGCACATTCGTTTACATTTCATGGAAGAATGGGAGTTGGGAGCCCAAATGTTGCGAAATTTATACACAGAGATTGTTTACACGCCAATCGCAACACCTGATGGGTCTGTCATCAAGAAGTTTAAAGGAAATAATAGTGGGCAACCATCTACAGTCGTTGACAACACGCTCATAGTTATTATAGCATTTAATTATGCAATGTTATCAAGTGGCATTCCTGAAGACAAAATTGACGACTGCTGTAGAATGTTTGCAAACGGTGACGACTTACTCTTGGCAGTGCATCCAGATTACGAATATATATTGGACGGATTTCAAAATCATTTTGGAAACCTTGGCCTTAACTTCGAGTTCACATCGAGGACAAAGGACAAATCAGAGTTATGGTTCATGTCAACACAAGGAGTTAAGTGTGAAGGTATCTACATACCAAAACTCGAAAGGGAAAGAATAGTCGCAATCCTTGAATGGGATCGATCAAACTTGCCTGAGCACCGTCTTGAAGCTATCTGTGCAGCCATGGTTGAAGCATGGGGTTACTCAGATTTAGTTCATGAAATTCGAAAGTTTTACGCGTGGCTTCTTGAAATGCAACCCTTCGCGAACCTGGCAAAGGAAGGCATGGCGCCATACATAGCAGAAACAGCACTCCGCAACCTCTACCTTGGAACAGGCATCAAAGAAGAAGAAATTGAAAAATATTTTAAGCAGTTTGTCAAGGATCTTCCTGGATACGTAGAAGATTACAATGAAGACGTTATTCATCAATCTGGTCAAGTTGACGCAGGGAGACAAGGCGGTAGCGGCACTCAAGGAGGCACACCACCAGCAGGA---------------------------------------------------AGTGGAGGCAC----TGGATCTGGCACTCAGGGCAATGGGGGTCAGACGGGA---------TCCCAAG---GAAGTGGTGGTCAACAAGGGTCTGGTGGGAGCACTGGTCAAGGAGCAGCTGGAAACAACGGCGG------------AGGCCAGACAGGAGGCTCTAGTGGA------------ACAGCCGGTCAAAGAGATAAGGACGTTGACGCAGGCTCAGCTGGAAAGATATCCGTACCAAAGCTTAAAGCTATGTCAAAGAAAATGCGCTTGCCAAAGGCAAAAGGAAAAGACGTCTTGCATTTGGACTTTTTGTTGACATATAAGCCACAACAGCAAGACATATCGAATACAAGAGCAACTAAGGAAGAGTTCGATAGATGGTACGACGCCATAAAGAAGGAGTACGAGATCGATGATACACAAATGACAGTTGTCATGAGTGGTCTGATGGTCTGGTGCATCGAAAATGGTTGCTCACCAAACATAAACGGAAATTGGACGATGATGGATGGGGATGAACAGAGAGTTTTCCCACTAAAACCAGTTATTGAAAACGCGTCTCCAACTTTCCGACAAGTTATGCATCATTTCAGTGATGCAGCTGAAGCGTATATAGAATACAGAAATTCTACTGAGCGATACATGCCAAGATACGGACTTCAGCGAAATCTCACCGACTATAGCTTAGCGCGGTATGCTTTTGATTTCTATGAAATGACTTCACGCACACCAGCTAGAGCTAAGGAAGCCCACATGCAG---ATGAAAGCCGCAGCAGTTCGTGGTTCAAACACACGACTGTTCGGCTTGGACGGAAATGTCGGCGAGACTCAGGAGAATACAGAGAGACACACAGCTGGCGACGTTAGTCGCAATATGCACTCTCTGTTGGGAGTGCAGCAACACCACTAGTCTCCTGGAAACCCTGTTTGCAGTACCTATAATATATACTA------ATATATAGTACGTTGGTGAGGCTTTGCCTC-----------------------GGTTTTACTATCTTATTATGTATGTATTTACAGCGTGAACCAGTCTGCAGCATGCAGGGTTGGACCCAGCGTGTTCTGGTGTAGCGTGTACTAGCGTCGAGCCATGAGACGGACTGCACTGGGTGTGGCTATGCCACTTGTGTTGCGAGTTTCCTGGTAAGAGAC----------------

>JX047404

---------------------AAAACAACAAAACTCAACACAACACAACAAAACACAACCAAACAAAACCAAGTTACATTTGCTCAGATTGTAGTGAACGGCTCGATTGGAAAGGTTCCTCGAGATCACTCTCTGACTCTTCTCTC----------TCAACCAACTTCATTCAAGCGAGATGGCGGGCTCTTGGACTCACGTGACATACAAGTGGCAACCAGATGTCAACAACGCACGTGATGTGAAAAGAGTAATGGAGATGTTTGCAGCGAAACATCAACGTTACACTGAGGAGCAGAGGCTTGCTCACAACAGCAAACTATTAAGGAAGGTTTACGTTACTAGTGCTGAGTTTACTGAATCAGCACAGAAACCGAAATGTCATCAAACATGGGTTGAAAAGTGCGACCACAACCCCACGGAGCACTTTGTTTATCAACGCTTC---ACATCTGAGAAGAAAGTGCCAACTACCAAACCTGAGACAACTTCTGTCACGAAGTTAATCAGGGATGTCCTTGAGATTTCGAAGGGCAGTGGGATAAAAATTGAGTTAATTGACAAGCGTGTCAAACGTAAAACTCAATTATCCATAAGGCAACACAATGGTAAAGATTTCTTGCACTGCAAAACCAAGCATGAAAATGGCTTGTTCAAACGCAAGGACATTGACATTAGTGTCAAGTGGTTGCCCACCATTGAAGCCATTGCAAAATGCTATAGCACGGTGAATGCAGAAGAACTGCAAAGTCTCAATAGGGGCAGTAGTGGTCTAACATTCATGCAAAACGATGAATTATTTATTGTGCGTGGAAGGATGCATGGTGAGATTGTCAATAGTTTACATGAGATTAAGCACGTAATGGAAATTGAACACTATGCTGATCCACAAGCAAACAGTTTCTGGAAAGGCTACACAGATGCATATGTTGAGAACAGAAACATATCTACCACTCACACAGAGCACACACCAACCATCAATTTGGAAGAGTGTGGCAAGAGAATGGCACTGTTAGAAATCCTATTCCATTCAACTTTTAAAATAACATGCAAAACGTGCAATATTGATGACCTTGAATTATCAGATGATGAATTTGGGGCTAAGCTATATAGTAATCTGCAGCGCATTGAAGAAAAACAACGTGAATATCTTGCTAAAGATCAAAAACTTTCACGCATGATTCACTTTGTAAAGGACCGATGTAATCCAAAATTTTCACATTTGCCTTTGCTATGGCAAGTGGCAGAAACAGTAGGACATTACACCGATAACCAATCGAAGCAGATAATTGATATCAGTGAGGCACTCATCAAAGTTAATACCTTAACTCCTGATGATGCAGTAAAGGCCAGTGTAGCATTGTTGGAAGTAGCACGATGGTATAAAAATCGGAAGGAATCACTCAAAACAGACACACTGGACTCATTCCGAAACAAGATTTCACCAAAGAGCACGATTAACGCAGCATTGATGTGTGACAACCAGTTAGATAAGAATGCAAATTTCGTATGGGGAAACAGAGAATACCATGCGAAGCGATTCTTCGCTAATTACTTTGAAGCTGTAGACCCGACTGATGCATATGAAAAACACGTCACACGCTTCAATCCCAATGGACAGCGGAAGTTATCAATTGGCAAATTAGTAATTCCGCTAGATTTCCAGAAAATTAGAGACTCGTTTGTTGGCTTATCGATAAATAAACAACCACTGAGCAAAGCTTGTGTAAGCAAAATTGATGGAGGCTACGTATATCCATGTTGCTGCGTCACAACGGAATTCGGAAAACCAGCATATTCTGAGATAATACCTCCAACAAAAGGACATATCACGATTGGAAACTCAGTGGACCCAAAAATAGTGGATTTACCGAACACAACACCACCGAGTATGTACATTGCGAAAGATGGATATTGTTATATTAACATATTCTTAGCAGCAATGATAAATGTTAATGAGGAATCCGCGAAAGATTACACTAAGTTCCTCAGAGATGAGCTTGTAGAACGGCTTGGTAAATGGCCTAAGCTAAAAGATGTGGCAACAGCATGTTACGCTTTATCTGTGATGTTCCCAGAAATTAAGAATGCCGAATTACCGCCAATATTAGTTGATCATGAGAGCAAGTCAATGCATGTCATTGATTCATATGGTTCACTTAGTGTTGGATTTCACATTCTAAAGGCAAGCACTGTGGGACAATTGATAAAATTTCAGTATGAGTCATTAGAAAGCGAGATGCGTGAATACATAGTAGGAGGCACCTTGACACAACAAACTTTTAGCACACTTCTCAAGACTCTTATAAAGAACATGTTTAAGCCAGATAAGATTAAACAGATAATAGAGGAAGAACCTTTCCTACTAATGATGGCGATTGCATCCCCAGCCGTACTTATCTCACTGTACAACAACTGCTACATCGAGCAGGCAATGACATATTGGATTGTCAAGAACCAAGGAGTTGCGGCAATTTTTGCACAATTGGAGGCTTTGGCGAAGAAAACCTCTCAGGCAGAATTATTAGTTCTGCAAATGCAAATACTTGAAAAAGCTTCAAACCAACTAAGACTTGCAGTCACTGGACTTAATCATGTTGATCCAGCGAAACGACTTTTGTGGTCTCACCTAGAGGCCATGACAACACGATCGGAAATGAACAAGGAACTTATAGCGGAAGGTTATGCACTGTACGACGAGCGTCTATATACTCTAATGGAAAAAAGTTATGTAGATCAATTAAACCAATCATGGGCAGAACTATCATACTGTGGAAGATTTTCAGCAATATGGCGTGTGTTCAGAGTCAGGAAATATTACAAGCCATCTTTAACCGTGAGAAAAAGCGTAGATTTAGGCGCTGTTTACAATATATCAGCTACGCATCTAATATCAAATTTAGTGCAGAAAAGTCGCGATCAAGTCAGCTCTACTTTAACCAAACTCCGCAACGGTTTCTATGACAAGATGGAGAAAGCGAGAGTTAGTGCAGTGAGGACGGTGTATTGGTTCATACCTGACATATTCAGACTAGTTCATATCTTTATAATTTTAAGCTTATTAACTACTATAGCTAATACGATAGTTACAACTATGAATGACTATAAAAAGTTAAAGAAACAACAAAGAGAAGACGAATATGAAGCTGAAATTAATGAGGTGCGAAAGATACACGCCACGTTGATGAAAGAGCACAACGATAATCTAACATGCGATCAATTCATTGAATATATACGGCAAACACACCCACGTCTCATCGAAGCAACTTTGGATTTGACACATACAGGTGTTATTCATGAAGGCAAATCCAACCTAGAGACAAATCTCGAACAGGCGATGGCAGTGGGAACTTTATTAACTATGATACTCGACCCACAAAAGAGTGATGCAGTATATAAAGTTCTCAATAAGATGCGGACAGTGATTAGCACAATTGAGCAGAATGTGCCATTTCCAGCAGTTAATTTCACAAGTATTTTAACACCTCCCGTGACACAGCAAAGCGTAGATGTTGATGAACCATTGACATTGAGTACTGACAAGAATCTGACTATAGATTTTGATACAAATCAAGATTTACCAGCAGACACATTTAGCAATGACGTTACTTTTGAGGACTGGTGGGCTAATCAGATAAATAACAATAGGACAGTGCCACACTACCGACTTGGAGGAAAGTTTGTAGAATTCACAAGAGAGAATGCAGCAATGGTTAGCATTGAGCTTGCTCATTCGAACATTGAAAGAGAATTTCTACTTAGAGGAGCTGTCGGGTCAGGGAAATCCACAGGTTTACCATATCACCTCAGCATGCGCGGAAAAGTATTATTGATAGAGCCTACTCGCCCATTAGCTGAGAACGTCTGTAGGCAACTGCAAGGTCCTCCATTTAATGTGAGCCCCACTTTACAAATGAGAGGATTGAGCACATTTGGCTGCACCCCTATCACAATAATGACATCTGGTTTTGCATTGCATATGTACGCTAATAACCCTGATAAGATCTCTGAGTATGACTTCATCATCTTTGACGAATGTCACATCATGGAAGCACCTGCAATGGCATTCTATTGTTTACTTAAGGAGTATGAATATCGAGGCAAGATAATTAAAGTTTCAGCAACACCACCAGGACGAGAATGCGAGTTCTCAACTCAACATCCAGTGGATATACATGTATGCGAAAGTTTGACACAACAACAATTCGTCATGGAGTTAGGAACGGGATCAACCGCTGACGCAACTAAGTACGGCAATAACATATTAGTATATGTTGCAAGTTACAATGATGTAGACTCTCTCTCCCATGCTCTGACTGAACTCAAATATTCAGTAATCAAAGTTGATGGAAGAACAATGAAGCAGAACACCACAGGAATTGTAACAAATGGAACATCCAGTAAGAAATGCTTCGTTGTAGCCACAAACATCATTGAAAACGGTGTAACGCTGGATGTTGACGTCGTAGTCGACTTTGGACTTAAAGTGACAGCTGAACTGGATGTTGATAACAGAGCAATAATGTATAAACGTGTGAGCATATCTTATGGCGAACGTATTCAGAGACTCGGAAGAGTTGGAAGGAACAAGCCTGGAACAGTCATCCGCATCGGGAAAACAATGAAAGGTTTACAAGAAATTCCAGCGATGATCGCCACTGAAGCAGCTTTCATGTGTTTCGCATATGGACTGAAGGTTATAACGCACAATGTATCTACAACACACCTAGCAAAATGCACTGTCAAACAAGCCAGAACTATGATGCAATTTGAATTATCACCATTTGTAATGGCTGAATTAGTCAAATTTGATGGTTCTATGCATCCACAGATTCATGAAGCGTTAACTAAGTACAAGTTGAGAGATTCTGTGATCATGTTAAGACCAAACGCAATACCAAAGGTTAATCTTCGCAACTGGTTAACGGCCCGTGATTACAATAGAATAGGCTGCTCATTGGAACTCGAGGATCATGTTAAAATACCATATTACATACGAGGAGTCCCTGACAAGTTGTACGGGAAGTTGTATGAAATTATCCTTCAATACAGCCCTACAAGTTGCTATGGAAGACTATCAAGCGCTTGTGCAGGTAAGGTAGCATATACACTGCGCACTGATCCTTGTTCGTTACCAAGAACAATAGCTATAATCAACGCATTAATCACCGAAGAGTATGCAAAGAGGGATCATTACAGAAACATGATAGCGAACCCTTCGTCATCGCACGCCTTTTCACTTAATGGGCTGGTATCCATGATCGCTTCTCGATACATGAAAGATCACACGAAGGAAAACATTGATAAACTTGTAAGAGTGCGCGACCAGCTACTTGAATTCCAAGGCACAGGTATGCAATTTCAAGATCCTTCAGAATTGATGGACATTGGTGCTTTAAACACAGTTATTCACCAAGGAATGGACGCTACGGCTGCTTGTATTGGACTGCAAGGGCGCTGGAACGCTTCGCTTATTCAACGCGATTTGATGATATCAGCAGGGGTCTTCACAGGAGGAATTCTCATGATGTGGTATCTTTTCACAAAATGGAGCAAGACAGAAGTGTTACACCAAGGAAAGAACAAGCGCAGTCGGCAAAAGCTACGATTCAAAGAGGCTCGTGATAATAAATATGCCTACGATGTAGTAGGATCAGAGGAAGTTCTTGGTGAGAATTTCGGAACTGCATACACTAAGAAAGGAAAAGGAAAGGGGACAAAAGTTGGCCTTGGAGTCAAACAGCACAAATTTCACATGATGTACGGGTTTGATCCACAAGAATACAACTTAATCCGCTTCGTGGATCCTTTAACAGGTGCGACACTAGACGAGCAAATCCATGCAGATATACAGTTAGTGCAAGAGCACTTCAGCGTCATCAGAGATGAAGCAGTAGCAAACGACACAATTGAAAGACAGCACATTTACGCTAATCCTGGATTACAAGCTTTCTTCATACAAAATGGATCGGCAAATGCACTGAGAGTTGATTTGACGCCGCATTCACCCTTGCGTGCCGTGACCAATAACAATATAGCAGGCTTCCCAGAGTATGAGGGCACACTTCGGCAAACAGGAACAGCTCTACAAATACCCGTGAATCAAGTTCCAGCTGCGAACGAAGCGGGGGTGGCACATGAATCGAAATCGATGATGGCAGGGCTAGGTGACTACACTCCAATATCACAGCAATTGTGCCTAGTTCAAAATGACTCAGATGGAATCAAAAGGAATGTATATTCAATTGGATATGGATCATATCTCATTGCGCCAGCACATTTATTTAAGTATAATAATGGCGAAATAACGATCAAATCATCACGAGGCCTATACAAAATCAGAAATTCAGTCGAAATAAAGTTGCATCCCATTGCGCATAGAGACATGGTTATAATTCAACTTCCAAAAGATTTTCCACCATTCCCAATGCGTCTTAAGTTTTCAAAACCATCTAGAGAGTCAAGGGTGTGCTTAGTCGGAGTGAATTTTCAGCAGAACTATAGCACATGTATCGTATCGGAGAGTAGCGTCACAGCACCAAAAGGAAACGGAGATTTCTGGAAACACTGGATATCCACAGTAGACGGACAATGCGGTCTCCCATTAGTAGATGTCAAGAGTAAGCACATAGTTGGAATACACAGTCTTGCATCAACTAGTGGAAATACTAACTTCTTTGTCGCCATGCCTGAAGACTTCAATGATTACATCAATAATCTTGTGCAAACCAACAAGTGGGAAAAAGGATGGCATTACAACCCAAATCTCATTTCATGGTGTGGTCTCAACCTAGTTGACTCAGCTCCAAAGGGTCTTTTCAAAACTTCAAAATTAGTAGAAGACCTTGACATGAGCGTTGAAGAACAATGTGAGGTTACAGAGACATGGCTCACGGAGTGTATTCAGGATAATTTACAGGTTGTTGCAAAATGTCCAGGCCAACTTGTCACCAAGCACGTTGTCAAAGGCCCATGCCCACACTTTCAGCTATATCTGTCAACACATGATGAAGCTAAAGCATACTTTGCACCATTACTCGGAAAATACGACAAGAGCAGATTAAACAGAGCAGCTTTTACCAAAGACATTTCAAAATATGCAAAACCAATCTACATTGGAGAAATCAATTACGAAGTCTTTGAAAAGGCTATACAACGTGTAATTAAAATTCTTAGAGACGTGGGAATGCAGCAATGCACGTATGTAACGGACGAGGGTGAAATATTCCAGTCACTTAACCTCAACGCTGCAGTTGGCGCCTTATACACAGGAAAGAAGAAAGATTATTTCAAGGATTTTTCAAATGAGGACAAATCAGAAATTATCATGAGATCCTGTGAGCGTTTATACAACGGACACCTTGGCGTGTGGAATGGTTCACTCAAAGCTGAAATAAGGCCCATAGAGAAAACAATGTTAAATAAGACTCGGACTTTTACAGCAGCACCATTAGAAACTTTACTTGGTGGCAAGGTCTGTGTTGATGATTTCAACAACCAATTCTACTCGCACCACTTAGAAGGTCCTTGGACAGTTGGAATAACAAAGTTTTATGGTGGGTGGAACCGTTTGTTGGAAAAATTGCCAGATGGTTGGATATACTGCGACGCCGATGGATCACAGTTCGACAGCTCTTTGACACCATATCTCATCAACGCCGTATTACACATGCGATTACAATTCATGGAAGAATGGAACTTAGGAGAACAAATGTTGCGAAACTTGTACACTGAAATCGTGTACACACCAATTGCAACACCAGATGGATCTGTAATCAAGAAATTTAAAGGAAATAACAGCGGGCAGCCGTCAACAGTTGTAGACAACACACTCATGGTGATATTAGCATTTAATTATGCAATGTTATCAAGTGGTGTTAAAGAGGAAGAAATAGACAATTGCTGCCGAATGTTCGCCAATGGTGATGATTTGCTACTTGCAGTGCATCCAGATTTCGAACACATACTGGACGGATTTCAAAATCACTTTGGGAACCTAGGTCTCAACTTTGAGTTTACATCACGAACAAGAGACAAGTCAGAGTTATGGTTCATGTCCACACGAGGTATCAAATGCGAAGGCGTCTATATACCAAAACTTGAGAAAGAAAGAATAGTTGCCATACTCGAGTGGGATCGGTCAAACTTACCTGAGCACCGTCTCGAAGCCATTTGTGCAGCCATGGTAGAGGCATGGGGATACCCAGACCTTGTCCAAGAGATACGGAAATTTTATGCGTGGCTTCTCGAAATGCAACCATTTGCAAATTTAGCTAAAGAGGGCCTAGCACCATATATCGCTGAAACTGCACTCAGAAATCTGTATTTGGGCACAGGGATCAAGGAAGAAGAGATTGAGAAATATTTCAAACAGTTTGCAAAAGATCTCCCTGGTTATATAGAAGATTACAACGAAGATGTTTTCCATCAATCCGGAACTGTGGATGCAGGAGCCCAAGGAGGGGGAAGTGGATCTGGAACAACGCCACCAGCAACA---------------------------------------------------GGTAGTGGAAC----AGGAACAAGAACACCATCTACTGGAACTCCAGCACAAGGCAACACACCTCCAG---CATCAGGTGGATCATCAGGAAGCAATGGAGGT---GGCCAATCAGGTTC---GAACGGCACTGG------------AGGCCA---AGCAGGCTCAAGCGGA------------GCAGGGGGTCAAAGAGACAAAGACGTTGACGCTGGCTCAACAGGGAAGATATCAGTGCCAAAGCTTAAGGCAATGTCGAAGAAAATGCGCCTGCCAAAGGCGAAAGGAAAAGACGTTTTACACTTAGATTTCCTGTTAACATATAAACCACAACAGCAAGATATATCAAACACTAGAGCAACTAAGGAAGAGTTTGATAGATGGTACGATGCCATAAAGAAGGAGTACGAAATTGATGACACACAAATGACAGTTGTCATGAGTGGTCTCATGGTATGGTGCATCGAAAATGGTTGCTCACCAAACATAAACGGAAATTGGACGATGATGGACGGAGACGAACAAAGGGTTTTTCCATTAAAGCCAGTCATTGAGAACGCATCTCCAACTTTCCGACAGATAATGCATCATTTTAGTGATGCAGCTGAAGCGTATATAGAGTACCGAAACTCCACAGAGCGATACATGCCAAGATACGGTCTTCAGCGAAATCTCACCGACTATAGCTTAGCGCGGTATGCTTTCGATTTCTATGAAATGACTTCGCGCACACCAGCTAGAGCTAAGGAAGCCCACATGCAG---ATGAAAGCCGCAGCAGTTCGTGGTTCAAACACACGTCTGTTCGGTCTGGACGGAAATGTCGGCGAGACTCAGGAGAATACAGAGAGACACACAGCTGGCGACGTTAGTCGCAACATGCACTCTCTGTTGGGAGTGCAGCAGCACCACTAGTCTCCTGGAAACCCTGTTTGCAGTACCTATAGTA--TATTA------CTAAATAGTACGTTCGTGAGGCCTTGCCTCGTGTGTATGTGAGGTTCTACCTCGTATTTACTATTTCAGTA--TATGTACTTTTAGCGTGAACCAGTCTGCAGGACACAGGGTTGGACCCAGTGTCTTCTGGTGTAGCGTGTACTAGCGTCGAGCCAATGGACGGACGGCACTGGGAGTGGTTTTACCATTGGTGCTGCGAGTCTCTTGGTGAGAGAC----------------

>JX047397

---------------------AAAACAACAAAACTCAACACAACACAACAAAACACAACCAAACAAAACCAAGTTACATTTGCTCAGATTGTAGTGAACGGCTCGATTGGAAAGGTTCCTCGAGATCACTCTCTGACTCTTCTCTC----------TCAACCAACTTCATTCAAGCGAGATGGCGGGCTCTTGGACTCACGTGACATACAAGTGGCAACCAGATGTCAACAACGCACGTGATGTGAAAAGAGTAATGGAGATGTTTGCAGCGAAACATCAACGTTACACTGAGGAGCAGAGGCTTGCTCACAACAGCAAACTATTAAGGAAGGTTTACGTTACTAGTGCTGAGTTTACTGAATCAGCACAGAAACCGAAATGTCATCAAACATGGGTTGAAAAGTGCGACCACAACCCCACGGAGCACTTTGTTTATCAACGCTTC---ACATCTGAGAAGAAAGTGCCAACTACCAAACCTGAGACAACTTCTGTCACGAAGTTAATCAGGGATGTCCTTGAGATTTCGAAGGGCAGTGGGATAAAAATTGAGTTAATTGACAAGCGTGTCAAACGTAAAACTCAATTATCCATAAGGCAACACAATGGTAAAGATTTCTTGCACTGCAAAACCAAGCATGAAAATGGCTTGTTCAAACGCAAGGACATTGACATTAGTGTCAAGTGGTTGCCCACCATTGAAGCCATTGCAAAATGCTATAGCACGGTGAATGCAGAAGAACTGCAAAGTCTCAATAGGGGCAGTAGTGGTCTAACATTCATGCAAAACGATGAATTATTTATTGTGCGTGGAAGGATGCATGGTGAGATTGTCAATAGTTTACATGAGATTAAGCACGTAATGGAAATTGAACACTATGCTGATCCACAAGCAAACAGTTTCTGGAAAGGCTACACAGATGCATATGTTGAGAACAGAAACATATCTACCACTCACACAGAGCACACACCAACCATCAATTTGGAAGAGTGTGGCAAGAGAATGGCACTGTTAGAAATCCTATTCCATTCAACTTTTAAAATAACATGCAAAACGTGCAATATTGATGACCTTGAATTATCAGATGATGAATTTGGGGCTAAGCTATATAGTAATCTGCAGCGCATTGAAGAAAAACAACGTGAATATCTTGCTAAAGATCAAAAACTTTCACGCATGATTCACTTTGTAAAGGACCGATGTAATCCAAAATTTTCACATTTGCCTTTGCTATGGCAAGTGGCAGAAACAGTAGGACATTACACCGATAACCAATCGAAGCAGATAATTGATATCAGTGAGGCACTCATCAAAGTTAATACCTTAACTCCTGATGATGCAGTAAAGGCCAGTGTAGCATTGTTGGAAGTAGCACGATGGTATAAAAATCGGAAGGAATCACTCAAAACAGACACACTGGACTCATTCCGAAACAAGATTTCACCAAAGAGCACGATTAACGCAGCATTGATGTGTGACAACCAGTTAGATAAGAATGCAAATTTCGTATGGGGAAACAGAGAATACCATGCGAAGCGATTCTTCGCTAATTACTTTGAAGCTGTAGACCCGACTGATGCATATGAAAAACACGTCACACGCTTCAATCCCAATGGACAGCGGAAGTTATCAATTGGCAAATTAGTAATTCCGCTAGATTTCCAGAAAATTAGAGACTCGTTTGTTGGCTTATCGATAAATAAACAACCACTGAGCAAAGCTTGTGTAAGCAAAATTGATGGAGGCTACGTATATCCATGTTGCTGCGTCACAACGGAATTCGGAAAACCAGCATATTCTGAGATAATACCTCCAACAAAAGGACATATCACGATTGGAAACTCAGTGGACCCAAAAATAGTGGATTTACCGAACACAACACCACCGAGTATGTACATTGCGAAAGATGGATATTGTTATATTAACATATTCTTAGCAGCAATGATAAATGTTAATGAGGAATCCGCGAAAGATTACACTAAGTTCCTCAGAGATGAGCTTGTAGAACGGCTTGGTAAATGGCCTAAGCTAAAAGATGTGGCAACAGCATGTTACGCTTTATCTGTGATGTTCCCAGAAATTAAGAATGCCGAATTACCGCCAATATTAGTTGATCATGAGAGCAAGTCAATGCATGTCATTGATTCATATGGTTCACTTAGTGTTGGATTTCACATTCTAAAGGCAAGCACTGTGGGACAATTGATAAAATTTCAGTATGAGTCATTAGAAAGCGAGATGCGTGAATACATAGTAGGAGGCACCTTGACACAACAAACTTTTAGCACACTTCTCAAGACTCTTATAAAGAACATGTTTAAGCCAGATAAGATTAAACAGATAATAGAGGAAGAACCTTTCCTACTAATGATGGCGATTGCATCCCCAGCCGTACTTATCTCACTGTACAACAACTGCTACATCGAGCAGGCAATGACATATTGGATTGTCAAGAACCAAGGAGTTGCGGCAATTTTTGCACAATTGGAGGCTTTGGCGAAGAAAACCTCTCAGGCAGAATTATTAGTTCTGCAAATGCAAATACTTGAAAAAGCTTCAAACCAACTAAGACTTGCAGTCACTGGACTTAATCATGTTGATCCAGCGAAACGACTTTTGTGGTCTCACCTAGAGGCCATGACAACACGATCGGAAATGAACAAGGAACTTATAGCGGAAGGTTATGCACTGTACGACGAGCGTCTATATACTCTAATGGAAAAAAGTTATGTAGATCAATTAAACCAATCATGGGCAGAACTATCATACTGTGGAAGATTTTCAGCAATATGGCGTGTGTTCAGAGTCAGGAAATATTACAAGCCATCTTTAACCGTGAGAAAAAGCGTAGATTTAGGCGCTGTTTACAATATATCAGCTACGCATCTAATATCAAATTTAGTGCAGAAAAGTCGCGATCAAGTCAGCTCTACTTTAACCAAACTCCGCAACGGTTTCTATGACAAGATGGAGAAAGCGAGAGTTAGTGCAGTGAGGACGGTGTATTGGTTCATACCTGACATATTCAGACTAGTTCATATCTTTATAATTTTAAGCTTATTAACTACTATAGCTAATACGATAGTTACAACTATGAATGACTATAAAAAGTTAAAGAAACAACAAAGAGAAGACGAATATGAAGCTGAAATTAATGAGGTGCGAAAGATACACGCCACGTTGATGAAAGAGCACAACGATAATCTAACATGCGATCAATTCATTGAATATATACGGCAAACACACCCACGTCTCATCGAAGCAACTTTGGATTTGACACATACAGGTGTTATTCATGAAGGCAAATCCAACCTAGAGACAAATCTCGAACAGGCGATGGCAGTGGGAACTTTATTAACTATGATACTCGACCCACAAAAGAGTGATGCAGTATATAAAGTTCTCAATAAGATGCGGACAGTGATTAGCACAATTGAGCAGAATGTGCCATTTCCAGCAGTTAATTTCACAAGTATTTTAACACCTCCCGTGACACAGCAAAGCGTAGATGTTGATGAACCATTGACATTGAGTACTGACAAGAATCTGACTATAGATTTTGATACAAATCAAGATTTACCAGCAGACACATTTAGCAATGACGTTACTTTTGAGGACTGGTGGGCTAATCAGATAAATAACAATAGGACAGTGCCACACTACCGACTTGGAGGAAAGTTTGTAGAATTCACAAGAGAGAATGCAGCAATGGTTAGCATTGAGCTTGCTCATTCGAACATTGAAAGAGAATTTCTACTTAGAGGAGCTGTCGGGTCAGGGAAATCCACAGGTTTACCATATCACCTCAGCATGCGCGGAAAAGTATTATTGATAGAGCCTACTCGCCCATTAGCTGAGAACGTCTGTAGGCAACTGCAAGGTCCTCCATTTAATGTGAGCCCCACTTTACAAATGAGAGGATTGAGCACATTTGGCTGCACCCCTATCACAATAATGACATCTGGTTTTGCATTGCATATGTACGCTAATAACCCTGATAAGATCTCTGAGTATGACTTCATCATCTTTGACGAATGTCACATCATGGAAGCACCTGCAATGGCATTCTATTGTTTACTTAAGGAGTATGAATATCGAGGCAAGATAATTAAAGTTTCAGCAACACCACCAGGACGAGAATGCGAGTTCTCAACTCAACATCCAGTGGATATACATGTATGCGAAAGTTTGACACAACAACAATTCGTCATGGAGTTAGGAACGGGATCAACCGCTGACGCAACTAAGTACGGCAATAACATATTAGTATATGTTGCAAGTTACAATGATGTAGACTCTCTCTCCCATGCTCTGACTGAACTCAAATATTCAGTAATCAAAGTTGATGGAAGAACAATGAAGCAGAACACCACAGGAATTGTAACAAATGGAACATCCAGTAAGAAATGCTTCGTTGTAGCCACAAACATCATTGAAAACGGTGTAACGCTGGATGTTGACGTCGTAGTCGACTTTGGACTTAAAGTGACAGCTGAACTGGATGTTGATAACAGAGCAATAATGTATAAACGTGTGAGCATATCTTATGGCGAACGTATTCAGAGACTCGGAAGAGTTGGAAGGAACAAGCCTGGAACAGTCATCCGCATCGGGAAAACAATGAAAGGTTTACAAGAAATTCCAGCGATGATCGCCACTGAAGCAGCTTTCATGTGTTTCGCATATGGACTGAAGGTTATAACGCACAATGTATCTACAACACACCTAGCAAAATGCACTGTCAAACAAGCCAGAACTATGATGCAATTTGAATTATCACCATTTGTAATGGCTGAATTAGTCAAATTTGATGGTTCTATGCATCCACAGATTCATGAAGCGTTAACTAAGTACAAGTTGAGAGATTCTGTGATCATGTTAAGACCAAACGCAATACCAAAGGTTAATCTTCGCAACTGGTTAACGGCCCGTGATTACAATAGAATAGGCTGCTCATTGGAACTCGAGGATCATGTTAAAATACCATATTACATACGAGGAGTCCCTGACAAGTTGTACGGGAAGTTGTATGAAATTATCCTTCAATACAGCCCTACAAGTTGCTATGGAAGACTATCAAGCGCTTGTGCAGGTAAGGTAGCATATACACTGCGCACTGATCCTTGTTCGTTACCAAGAACAATAGCTATAATCAACGCATTAATCACCGAAGAGTATGCAAAGAGGGATCATTACAGAAACATGATAGCGAACCCTTCGTCATCGCACGCCTTTTCACTTAATGGGCTGGTATCCATGATCGCTTCTCGATACATGAAAGATCACACGAAGGAAAACATTGATAAACTTGTAAGAGTGCGCGACCAGCTACTTGAATTCCAAGGCACAGGTATGCAATTTCAAGATCCTTCAGAATTGATGGACATTGGTGCTTTAAACACAGTTATTCACCAAGGAATGGACGCTACGGCTGCTTGTATTGGACTGCAAGGGCGCTGGAACGCTTCGCTTATTCAACGCGATTTGATGATATCAGCAGGGGTCTTCACAGGAGGAATTCTCATGATGTGGTATCTTTTCACAAAATGGAGCAAGACAGAAGTGTTACACCAAGGAAAGAACAAGCGCAGTCGGCAAAAGCTACGATTCAAAGAGGCTCGTGATAATAAATATGCCTACGATGTAGTAGGATCAGAGGAAGTTCTTGGTGAGAATTTCGGAACTGCATACACTAAGAAAGGAAAAGGAAAGGGGACAAAAGTTGGCCTTGGAGTCAAACAGCACAAATTTCACATGATGTACGGGTTTGATCCACAAGAATACAACTTAATCCGCTTCGTGGATCCTTTAACAGGTGCGACACTAGACGAGCAAATCCATGCAGATATACAGTTAGTGCAAGAGCACTTCAGCGTCATCAGAGATGAAGCAGTAGCAAACGACACAATTGAAAGACAGCACATTTACGCTAATCCTGGATTACAAGCTTTCTTCATACAAAATGGATCGGCAAATGCACTGAGAGTTGATTTGACGCCGCATTCACCCTTGCGTGCCGTGACCAATAACAATATAGCAGGCTTCCCAGAGTATGAGGGCACACTTCGGCAAACAGGAACAGCTCTACAAATACCCGTGAATCAAGTTCCAGCTGCGAACGAAGCGGGGGTGGCACATGAATCGAAATCGATGATGGCAGGGCTAGGTGACTACACTCCAATATCACAGCAATTGTGCCTAGTTCAAAATGACTCAGATGGAATCAAAAGGAATGTATATTCAATTGGATATGGATCATATCTCATTGCGCCAGCACATTTATTTAAGTATAATAATGGCGAAATAACGATCAAATCATCACGAGGCCTATACAAAATCAGAAATTCAGTCGAAATAAAGTTGCATCCCATTGCGCATAGAGACATGGTTATAATTCAACTTCCAAAAGATTTTCCACCATTCCCAATGCGTCTTAAGTTTTCAAAACCATCTAGAGAGTCAAGGGTGTGCTTAGTCGGAGTGAATTTTCAGCAGAACTATAGCACATGTATCGTATCGGAGAGTAGCGTCACAGCACCAAAAGGAAACGGAGATTTCTGGAAACACTGGATATCCACAGTAGACGGACAATGCGGTCTCCCATTAGTAGATGTCAAGAGTAAGCACATAGTTGGAATACACAGTCTTGCATCAACTAGTGGAAATACTAACTTCTTTGTCGCCATGCCTGAAGACTTCAATGATTACATCAATAATCTTGTGCAAACCAACAAGTGGGAAAAAGGATGGCATTACAACCCAAATCTCATTTCATGGTGTGGTCTCAACCTAGTTGACTCAGCTCCAAAGGGTCTTTTCAAAACTTCAAAATTAGTAGAAGACCTTGACATGAGCGTTGAAGAACAATGTGAGGTTACAGAGACATGGCTCACGGAGTGTATTCAGGATAATTTACAGGTTGTTGCAAAATGTCCAGGCCAACTTGTCACCAAGCACGTTGTCAAAGGCCCATGCCCACACTTTCAGCTATATCTGTCAACACATGATGAAGCTAAAGCATACTTTGCACCATTACTCGGAAAATACGACAAGAGCAGATTAAACAGAGCAGCTTTTACCAAAGACATTTCAAAATATGCAAAACCAATCTACATTGGAGAAATCAATTACGAAGTCTTTGAAAAGGCTATACAACGTGTAATTAAAATTCTTAGAGACGTGGGAATGCAGCAATGCACGTATGTAACGGACGAGGGTGAAATATTCCAGTCACTTAACCTCAACGCTGCAGTTGGCGCCTTATACACAGGAAAGAAGAAAGATTATTTCAAGGATTTTTCAAATGAGGACAAATCAGAAATTATCATGAGATCCTGTGAGCGTTTATACAACGGACACCTTGGCGTGTGGAATGGTTCACTCAAAGCTGAAATAAGGCCCATAGAGAAAACAATGTTAAATAAGACTCGGACTTTTACAGCAGCACCATTAGAAACTTTACTTGGTGGCAAGGTCTGTGTTGATGATTTCAACAACCAATTCTACTCGCACCACTTAGAAGGTCCTTGGACAGTTGGAATAACAAAGTTTTATGGTGGGTGGAACCGTTTGTTGGAAAAATTGCCAGATGGTTGGATATACTGCGACGCCGATGGATCACAGTTCGACAGCTCTTTGACACCATATCTCATCAACGCCGTATTACACATGCGATTACAATTCATGGAAGAATGGAACTTAGGAGAACAAATGTTGCGAAACTTGTACACTGAAATCGTGTACACACCAATTGCAACACCAGATGGATCTGTAATCAAGAAATTTAAAGGAAATAACAGCGGGCAGCCGTCAACAGTTGTAGACAACACACTCATGGTGATATTAGCATTTAATTATGCAATGTTATCAAGTGGTGTTAAAGAGGAAGAAATAGACAATTGCTGCCGAATGTTCGCCAATGGTGATGATTTGCTACTTGCAGTGCATCCAGATTTCGAACACATACTGGACGGATTTCAAAATCACTTTGGGAACCTAGGTCTCAACTTTGAGTTTACATCACGAACAAGAGACAAGTCAGAGTTATGGTTCATGTCCACACGAGGTATCAAATGCGAAGGCGTCTATATACCAAAACTTGAGAAAGAAAGAATAGTTGCCATACTCGAGTGGGATCGGTCAAACTTACCTGAGCACCGTCTCGAAGCCATTTGTGCAGCCATGGTAGAGGCATGGGGATACCCAGACCTTGTCCAAGAGATACGGAAATTTTATGCGTGGCTTCTCGAAATGCAACCATTTGCAAATTTAGCTAAAGAGGGCCTAGCACCATATATCGCTGAAACTGCACTCAGAAATCTGTATTTGGGCACAGGGATCAAGGAAGAAGAGATTGAGAAATATTTCAAACAGTTTGCAAAAGATCTCCCTGGTTATATAGAAGATTACAACGAAGATGTTTTCCATCAATCCGGAACTGTGGATGCAGGAGCCCAAGGAGGGGGAAGTGGATCTGGAACAACGCCACCAGCAACA---------------------------------------------------GGTAGTGGAAC----AGGAACAAGAACACCATCTACTGGAACTCCAGCACAAGGCAACACACCTCCAG---CATCAGGTGGATCATCAGGAAGCAATGGAGGT---GGCCAATCAGGTTC---GAACGGCACTGG------------AGGCCA---AGCAGGCTCAAGCGGA------------GCAGGGGGTCAAAGAGACAAAGACGTTGACGCTGGCTCAACAGGGAAGATATCAGTGCCAAAGCTTAAGGCAATGTCGAAGAAAATGCGCCTGCCAAAGGCGAAAGGAAAAGACGTTTTACACTTAGATTTCCTGTTAACATATAAACCACAACAGCAAGATATATCAAACACTAGAGCAACTAAGGAAGAGTTTGATAGATGGTACGATGCCATAAAGAAGGAGTACGAAATTGATGACACACAAATGACAGTTGTCATGAGTGGTCTCATGGTATGGTGCATCGAAAATGGTTGCTCACCAAACATAAACGGAAATTGGACGATGATGGACGGAGACGAACAAAGGGTTTTTCCATTAAAGCCAGTCATTGAGAACGCATCTCCAACTTTCCGACAGATAATGCATCATTTTAGTGATGCAGCTGAAGCGTATATAGAGTACCGAAACTCCACAGAGCGATACATGCCAAGATACGGTCTTCAGCGAAATCTCACCGACTATAGCTTAGCGCGGTATGCTTTCGATTTCTATGAAATGACTTCGCGCACACCAGCTAGAGCTAAGGAAGCCCACATGCAG---ATGAAAGCCGCAGCAGTTCGTGGTTCAAACACACGTCTGTTCGGTCTGGACGGAAATGTCGGCGAGACTCAGGAGAATACAGAGAGACACACAGCTGGCGACGTTAGTCGCAACATGCACTCTCTGTTGGGAGTGCAGCAGCACCACTAGTCTCCTGGAAACCCTGTTTGCAGTACCTATAGTA--TATTA------CTAAATAGTACGTTCGTGAGGCCTTGCCTCGTGTGTATGTGAGGTTCTACCTCGTATTTACTATTTCAGTA--TATGTACTTTTAGCGTGAACCAGTCTGCAGGACACAGGGTTGGACCCAGTGTCTTCTGGTGTAGCGTGTACTAGCGTCGAGCCAATGGACGGACGGCACTGGGAGTGGTTTTACCATTGGTGCTGCGAGTCTCTTGGTGAGAGAC----------------

>JX047395

---------------------AAAACAACAAAACTCAACACAACACAACAAAACACAACCAAGCAAAACCAAGTTACCTTTGCTCAGATTGTAGTGAACGGCTCAGTAGGAAAGGTTCCTCGAGATCACTCTCTGATTCTTCTTTC----------TCAACCAACTTCATTCAAGCGAGATGGCGGGCTCTTGGACTCACGTGACATACAAGTGGCAACCAGATGTCAACAACGCACGTGATGTGAAAAGAGTAATGGAGATGTTTGCAGCAAAACATCAACGTTACACTGAGGAGCAAAGGCTTGCTCACAATAGCAAGCTATTAAGGAAGGCTTGTGTCACTAGTGCTGAGTTTATTGAACCAGCACAGAAACCAAAATGTCGCCAGACATGGGTTGAAAAGTGCGACCACAACCCCACAGAGCACTTTGTTTATCAACGCCTC---ACACCTGAGAAGAAAGTGCTTAACACCAAACCTAAGTCAACTTCTATCACGAAGTTAATCAGGGATGTCCTTGAGATTTCGAAGGGCAGCGGGATTAAAATTGAATTAATTGACAAGCGTGCCAAACGCAAAACTCAATTATCCATAAGGCAACACAAAGGTAAAGATTTCTTGCACTGCAAAACCAAGCATGAAAATGGCCTGTTTAAACGCAAGGACATTGACATCAATGTCAAGTGGTTGCACACCATTGAAGCCATTGCAAAATGCTACAGCACGGTGAATGCAAAAGAACTGCAAAGTCTCACTAGAGGCAGTAGTGGTCTAACATTCATGCAAAACGATGAATTATTCATCGTGCGTGGAAGGATGCATGGTGAGATTATCAATAGTTTACATGAGAATAAACACGTAATGGAAATCGAGCACTATGCTGATCCACAAGCAAACAGTTTCCGGAAGGGCTACACAGACGCGTATGTCGAAAACAGAAACATATCTACCACTCACACAGAGCACACACCAACTATTAATTTAGAGGAGTGTGGTAAGAGGATGGCACTGTTAGAAATTCTATTCCACTCAACTTTTAAGATCACATGCAAAACGTGCAATATTGATGATCTGGAATTATCAGATGATGAATTTGGGGCCAAGTTATATAGCAATCTGCAGCGTATTGAAGAAAAGCAACGTGAATATCTCGCTAAAGATCAAAAACTTCTACGCATGATACACTCTGTAAAGGATCGGTGTAACCCAAAATTTTCACATTTGCCTCTACTATGGCAAGTGGCGGAAACAGTAGGGCATTACACCGACAACCAATCAAAGCAGATAATTGACATCAGTGAGGCGCTTATCAAAGTTAATACTTTAACTCCTGATGATGCAGTGAAGGCCAGTGTAGCATTATTAGAAGTAGCACGGTGGTATAAAAATCGAAAGGAATCACTCAAAACGGACACACTGGATTCATTTCGAAACAAGATTTCTCCAAAGAGCACGATCAACGCAGCACTAATGTGTGATAACCAGTTAGATAAGAATGCAAATTTCGTATGGGGAAACAGAGAATATCATGCAAAGCGGTTCTTCGCTAATTATTTTGAAGCCGTGGACCCAACTGACGCATATGAAAAACACGTAACACGCTTCAACCCAAATGGACAGCGGAAATTATCGATTGGCAAGCTAGTAATTCCACTAGATTTCCAGAAGATCAGAGATTCGTTCGTTGGCCTATCGATAAATAAACAACCACTGAGCAAAGCTTGCGTAAGCAAAATTGATGGAGGCTACGTATATCCATGTTGCTGCGTTACAACGGAGTTTGGAAAACCAGCATATTCTGAGATAATACCTCCAACGAAAGGACATATCACGATTGGAAACTCAGTGGACCCAAAAATAGTGGATTTACCGAATACTACACCACCAAGTATGTACATTGCAAAAGATGGATACTGTTACATTAACATATTCTTGGCAGCAATGATAAACGTTAATGAAGAATCCGCAAAGGATTACACTAAGTTTCTTAGAGATGAGCTGGTGGAACGGCTTGGTAAATGGCCAAAATTGAAAGATGTGGCTACAGCATGTTATGCGCTATCTGTGATGTTTCCTGAAATCAAGAACGCTGAGTTACCACCGATATTAGTTGACCATGAGAGCAAATCGATGCACGTAATAGATTCGTATGGTTCGCTGAGTGTGGGGTTTCACATCCTCAAAGCAAGCACCATTGGACAGTTGATAAAATTCCAATATGAATCGATGGGAAGCGAAATGCGAGAATATATAGTAGGAGGCACTTTAACACAACAAACTTCCAACACTCTACTTAAAACTCTCACAAAGAACATGTTCAAGCCTGAGAAAATTAAGCAGATAATTGAGGAAGAGCCTTTCTTACTAATGATGGCAATCGCATCACCAACTGTACTCATATCTTTATACAATAATTGTTACATAGAGCAAGCTATGACATATTGGATTGTTAAAAATCAAGGTGTTGCAGCAATATTCGCACAGCTCGAAGCATTAGCCAAAAAGACGTCTCAAGCTGAATTATTAGTTTTACAGATGCAAATACTTGAAAAGGCGTCTAATCAGCTGAGGCTTGCAGTGACAGGACTAAACCATGTTGACCCCGCTAAGCGTCTTTTATGGTCACATCTTGAAGCTATGACAACGCGATCGGAGATGAACAAGGAACTAATAGCAGAAGGCTATGCTTTATATGACGAGCGCTTGTACACTTTGATGGAAAAAAGTTACGTAGATCAATTAAACCAGTCATGGGCAGAATTATCATACTGTGGAAAATTTTCAGCAATATGGCGTGTGTTCAAAGTCAGGAAATATTACAAGCCGTCTTTAACCGTGAGAAAAAGCGTAGATTTAGGCGCTGTATACAATATATCAGCTACGCATCTAATATCAGGTTTAGTGCAGAAAAGTCGCGATCAAGTCAGCTCTACTTTAACCAAACTCCGCAACGGTTTTTATGATAAATTAGAGAAGACTAGAGTGCGTGCAATCAAAACAGTATATTGGTTTGTACCCGATATATTTAGACTTGTTCACATTTTTATAGTGCTAAGTTTGTTAACCACCATAGCTAACACGATCGTAATAACTATGAATGACTATAAAAAGTTGAAAAAGCAACAAAGAGAGGATGCGTATGAAGCTGAGATAAACGAAGTTCGCAAGATTCATGCCGCCTTGATGAAGGAGCATAATGACAATTTAACATGCGAACAGTTCATTGAACATATGCGTCAAACACATCCACGATTAGTTGAAGCTACATTGGAGTTGACCCACACAGGTGTAATTCATGAAGGAAAGTCTAATCTAGAAACCAACCTTGAGCAAGCTATGGCTGTGGGAACACTACTCACGATGATACTTGATCCTCAGAAGAGTGACGCTGTGTACAAAGTTCTAAACAAAATGCGAACAGTGATTAGTACATTTGAACAGAATGTCTCATTCCCTTCAATTAACTTCACTAACATCTTGACACCACCCGTAACACAGCAGAGTGTGGACGTCGATGAGCCTCTTACTTTGAGTACTGACAAGAACTTAACGATAGATTTTGATACAAACCAAGATTTACCTGCAGACACTTTCAGTAACGATGTTACATTCGAGGATTGGTGGGCAAATCAGATAAGCAATAATAGAACAGTTCCACACTATCGTCTCGGTGGCAAGTTTGAAGAATTTACAAGGGAAAATGCAGCACTTGTTAGTATTGAATTAGCTCACTCGAACATTGAAAAGGAATTTCTTTTAAGGGGTGCGGTTGGCTCAGGAAAGTCCACAGGGCTGCCATACCATCTTAGTGCGCGTGGAAAAGTACTTCTAATTGAACCAACAAGACCACTTGCCGAGAATGTATGCAGACAGTTACAAGGGCCACCATTCAATGTTAGTCCGACACCTCAAATGCGTGGGTTGAGCTCTTTTGGCTGCACGCCAATCACGATTATGACCTCTGGTTTTGCATTACATATGTACGCAAACAATCCGGACAAGATATCTGATTACGACTTCATCATATTTGATGAATGTCATATCATGGAAGCACCAGCTATGGCATTTTATTGTTTGCTGAAGGAATATGAATATCGAGGCAAAATCATTAAAGTCTCCGCAACACCACCTGGAAGAGAATGTGAATTTACCACACAACATCCAGTTGACATTCACGTCTGCGAGAACCTCACACAACAGCAATTTGTAAGGGAACTTGGCACGGGTTCAAATGTCGACGCGACGAAATATGGAAATAACATACTTGTTTACGTTGCAAGCTACAATGACGTCGATTCGCTATCACACGCGCTAACTGAATTACATTACTCAGTTATCAAAGTTGACGGTAGGACTATGAAGCAGAACACCACAGGCATTGTAACTAATGGCACGTCACAAAAGAAGTGTTTTGTCGTTGCAACGAACATAATCGAAAATGGTGTTACATTGGATGTTGATGTCGTAGTGGACTTTGGACTGAAAGTCACAGCTGAGCTGGATGTTGACAATAGAGCAATTCTATACAAACGTGTCAGCATCTCGTATGGTGAGCGTATTCAAAGACTCGGTCGTGTTGGAAGGAATAAACCCGGAACAGTGGTCCGCATTGGGAAAACTATGAAAGGTCTGCAGGAAATTCCAGCGATGATAGCAACGGAAGCTGCTTTTATGTGTTTTGCATACGGACTCAAAGTTATAACACACAATGTATCTACAACACACTTAGCTAAATGCACGGTAAAACAAGCAAGAACAATGATGCAATTTGAACTATCACCATTCGTAATGGCAGAATTAGTCAAATTCGATGGTTCAATGCATCCACAAATTCATGAAGCACTCGTAAAATACAAACTTAGAGATTCTGTGATAATGTTAAGACCAAATGCTATTCCGAAGGTCAACTTTCACAACTGGTTGACTGCACGTGATTACAACAGGATGGGCTGTACAGTAGAACTTGAAGATCATGTGAAAATACCATACTATATACGAGGGATTCCTGATAAACTATATGGAAAATTGTACGATATCATTTTACAATACAGCCCAACTAGTTGTTATGGAAGACTATCAAGTGCCTGTGCGGGAAAAGTAGCATATACTCTGCGCACTGATCCTTGTTCGTTACCACGCACAATAGCTATCATTAATGCCTTAATTACTGAAGAGTATGCAAAGAGGGACCATTACAGAAACATGATAGCAAACCCCTCATCATCGCACGCCTTTTCACTTAATGGGCTGGTATCCATGATCGCTTCTCGGTATATGAAAGACCACACGAAGGAAAACATTGACAAGCTTGTAAGAGTGCGCGACCAACTACTTGAGTTCCAAGGCACAGGTATGCAATTTCAAGATCCTTCAGAATTGATGGACATTGGTGCATTAAATACAGTTATTCACCAAGGAATGGACGCCACGGCTGCTTGTATTGGATTGCAAGGGCGCTGGAATGCTTCGCTCATTCAGCGCGACTTGATGATATCAGCAGGAGTCTTCGCAGGAGGAATTCTCATGATGTGGTATCTTTTCACAAAATGGAGCAAGACAGAAGTGTCACACCAAGGAAAGAACAAGCGTAGTCGGCAAAAACTACGATTCAAAGAGGCGCGTGATAACAAATACGCCTATGATGTGACAGGGTCAGAAGAAGTTCTTGGTGAGAATTTCGGAACTGCTTATGTCAAGAAAGGAAAGGGAAAAGGAACAAAAGTTGGTCTTGGAGTCAAACGACACAAATTCCACATGATGTACGGTTTTGACCCACAGGAATACAACTTAATCCGCTTCGTGGATCCTTTAACAGGCGCGACACTAGACGAGCAAATTCATGCAGATATACGTTTAGTGCAAGAGCACTTTAGCGTCATCAGAGATGAAGCAGTGGCAAATGACACAATCGAAAGACAACACATTTACAGCAATCCTGGACTACAAGCGTTCTTCATACAAAATGGATCTGCGAATGCACTGAGAGTTGATTTAACACCACATACACCACTGCGTGTCGTAACCAATAACAATATAGCAGGTTTCCCAGGATATGAAGGTACACTTCGACAAACAGGGACAGCTTTACAGATACCCGTGAATCAAGTTCCAGCTGCGAATGAAGCGGGGGTGGCACATGAGTCAAAATCGATGATGGCAGGATTAGGCGACTACACCCCAATATCACAGCAGTTGTGTCTAGTCCAGAATGACTCAGATGGAATCAAAAGGAATGTGTACTCAATTGGATATGGATCATATCTCATTTCACCGGCACACTTATTTAAATATAACAATGGCGAAATAACAATTAAATCATCAAGAGGTTTGTATAAGATCAGAAATTCAGTAGAACTCAAATTGCATCCCATTGCACATAGAGATATGGTCATTATTCAACTTCCGAAAGATTTTCCGCCGTTCCCAATGCGTCTCAAGTTTTCTAAACCATCTAGAGAGTCAAGAGTGTGCTTAGTTGGAGTGAACTTCCAACAAAACTACAGCACATGCATTGTATCGGAGAGTAGTGTCACAGCACCAAAAGGCAATGGAGATTTCTGGAAACACTGGATATCCGCGGTGGACGGACAATGCGGCCTCCCATTAGTGGATGTCAAGAGCAAGCACATAGTTGGGATACACAGCCTTGCATCAACTAGTGGAAACACTAACTTTTTCGTCGCCATGCCTGAAGACTTCAATGACTACATTCACAATCTTGTGCAAACCAACAAGTGGGAAAAAGGATGGCATTACAACCCAAATCTCATTTCATGGTGTGGTCTCAATCTAGTCGATTCAGCTCCAAAGGGCCTCTTCAAAACTTCGAAATTAGTGGAAGACCTTGACATGAGTGTTGAAGAGCAGTGCAAGGTTACAGAGACATGGCTCACAGAATGCATTCAGGACAATTTACAGGTTGTCGCAAAATGCCCAGGCCAACTTGTCACCAAGCACGTTGTCAAAGGCCCATGCCCACACTTTCAGCTATATCTGTCAACACATGATGAAGCCAAAGCATACTTTGCACCACTACTCGGAAAATACGATAAGAGCAGATTGAACAGAGCAGCTTTTATCAAAGACATTTCAAAATATGCAAAACCAATCTACATTGGAGAAATCAATTATGATGTCTTTGAAAAGGCTATAGAACGTGTGATTAAAATTCTTAGAGATGTGGGAATGCAACAATGCACGTATGTCACGGATGAGGATGAAATATTTCAGTCACTCAACCTCAACGCCGCAGTTGGTGCCTTATACACAGGAAAGAAGAAAGACTATTTTAAGGATTTCTCAAATGAAGACAAATCAGAAATCATTATGAGATCCTGTGAGCGTATCTACAACGGACAACTTGGTGTGTGGAATGGTTCACTCAAAGCTGAAATAAGGCCCATAGAGAAAACTATGTTAAACAAGACTCGAACCTTCACAGCAGCACCATTAGAAACTCTACTTGGTGGCAAAGTCTGCGTCGATGATTTCAACAATCAGTTCTACTCACACCACTTAGAGGGTCCTTGGACAGTTGGAATTACAAAGTTCTATGGTGGGTGGAATCGTTTATTAGAAAAATTACCAGACGGTTGGATATACTGCGACGCCGATGGATCACAGTTTGACAGCTCCTTGACACCATACCTTATCAACGCTGTATTACATATTCGATTACAATTCATGGAGGAATGGAACTTAGGAGAACAAATGTTGCGAAACTTGTACACCGAAATCGTGTACACACCAATTGCAACACCAGATGGATCTGTAATCAAGAAATTTAAAGGAAATAACAGCGGGCAGCCGTCAACAGTTGTAGACAACACACTCATGGTGATATTAGCATTTAATTATGCAATGTTATCAAGTGGTGTTAAAGAGGAAGAAATAGACAATTGCTGCCGAATGTTCGCCAATGGTGATGATTTGCTACTTGCAGTGCATCCGAATTTCGAACACATATTGGACGAATTTCAAAATCACTTCGGAAACCTAGGTCTCAACTTTGAGTTTACATCACGAACAAGAGACAAGTCAGAGTTATGGTTCATGTCCACACGAGGTATCAAATGCGAAGGCGTCTATATACCAAAGCTTGAGAAAGAAAGAATAGTTGCCATACTCGAGTGGGATCGGTCAAACTTACCTGAGCACCGTCTCGAAGCCATTTGTGCAGCCATGGTAGAGGCATGGGGATACTCAGACCTTGTCCAAGAGATACGGAAATTTTATGCGTGGCTTCTCGAAATGCAACCATTTGCAAATTTAGCTAAAGAGGGCCTAGCACCATATATCGCTGAAACTGCACTCAGAAATCTGTATTTGGGCACAGGGATTAAGGAAGAAGAGATTGAGAAATATTTCAAACAGTTTGCCAAAGATCTCCCTGGTTATATAGAAGATTACAACGAAGATGTTTTCCATCAATCCGGAACTGTGGATGCAGGAGCCCAAGGAGGGGGAAGTGGATCTGGAACAACGCCACCAGCAACA---------------------------------------------------GGTAGTGGAAC----AGGAACAAGAACACCATCTACTGGAACTCCAGCGCAAGGCAACACGCCTCCAG---CATCAGGTGGATCGTCAGGAAACAATGGAGGC---GGCCAATCAGGTTC---AAACGGCACTGG------------AGGCCA---AGCAGGCTCAAGCGGA------------GCAGGGGGTCAAAGAGACAAAGACGTTGACGCTGGCTCAACAGGGAAGATATCAGTGCCAAAGCTTAAGGCAATGTCGAAGAAAATGCGCCTGCCAAAGGCGAAAGGAAAAGACGTTTTACACTTAGATTTCCTGTTAACATATAAACCACAACAGCAAGATATATCAAACACTAGAGCAACTAAGGAAGAGTTTGATAGATGGTACGATGCCATAAAGAAGGAGTACGAAATTGATGACACACAAATGACAGTTGTCATGAGTGGTCTCATGGTATGGTGCATCGAAAATGGTTGCTCACCAAACATAAACGGAAATTGGACTATGATGGACGGAGACGAACAAAGGGTTTTTCCATTAAAGCCAGTCATTGAGAACGCATCTCCAACTTTCCGACAGATAATGCATCATTTTAGTGATGCAGCTGAAGCGTATATAGAGTACCGAAACTCTACAGAGCGATACATGCCAAGATACGGTCTTCAGCGAAATCTCACCGACTATAGCTTAGCACGGTATGCTTTCGATTTCTATGAAATGACTTCGCGCACACCAGCTAGAGCTAAGGAAGCCCACATGCAG---ATGAAAGCCGCAGCAGTTCGTGGTTCAAACACACGTCTGTTCGGTCTGGACGGAAATGTCGGCGAGACTCAGGAGAATACAGAGAGACACACAGCTGGCGACGTTAGTCGCAACATGCACTCTCTGTTGGGAGTGCAGCAGCACCACTAGTCTCCTGGAAACCCTGTTTGCAGTACCTATAGTA--TATTA------CTAAATAGTACGTTCGTGAGGCCTTGCCTCGTGTGTATGTGAGGTTCTACCTCGTATTTACTATTTCAGTA--TATGTACTTTTAGCGTGAACCAGTCCGCAGGACACAGGGTTGGACCCAGTGTCTTCTGGTGTAGCGTGTACTAGCGTCGAGCCAATGGACGGACGGCACTGGGAGTGGTTTTACCATTGGTGCTGCGAGTCTCTTGGTGAGAGAC----------------

>JX047394

---------------------AAAACAACAAAACTCAACACAACACAACAAAACACAACCAAGCAAAACCAAGTTTCCTCTGCTCAGATTGTAGTGAACGGCTTGATAGGAAAGGTTCCTCGAGATCACTCTCTGATTCTTCTTTC----------TCAACCAACTTCATTCAAGCGAGATGGCGGGCTCTTGGACTCACGTGACATACAAGTGGCAACCAGATGTCAACAACGCACGTGATGTGAAAAGAGTGATGGAGATGTTTGCAGCAAAACATCAACGTTACACTGAGGAGCAAAGGCTTGCTCACAACAGCAAGCTATTAAGGAAGGCTTGTGTCACTAGTGCTGAGTTTATTGAACCAGCACAGAAACCAAAATGTCGCCAGACATGGGTTGAAAAGTGCGACCACAACCCCACAGAGCACTTTATTTATCAACGCTTC---ACACCTGAGAAGAAAGTGCTTAACACCAAACCTGAGACAACCTCTGTTACGAAATTAATCAGGGATGTCCTTGAGATTTCGAAGGGCAGTGGGATTAAAATTGAGTTGATCGATAAGCGTATTAAACGTAAGACTCAATTATCCATAAGGCAATACAATGGCAAAAACTTCTTGCACTGCAAAACCAGGCATGAAAATGGCCTGTTTAAACGCAAGGACATTGACATTAATGTCAAGTGGTTGCCCACCATTGAAGCCATTGCAAAATGCTACAGCACGGTGAATGCGGAAGAACTGCAAAGTCTCAATAAAGGCAGTAGTGGTCTTACATTCATGCAAAACGATGAATTGTTCATCGTGCGTGGAAGGATGCATGGTGAGATTGTCAATAGTTTACACGAAAATAAGCACGTTATGGAAATTGAACACTATGCTGACCCACAAGCAAACAGTTTCTGGAAAGGTTACACAGACGCGTATGTCGAAAACAGAAACATATCTACCACTCACACAGAGCACACACCAACTATTAATTTAGAGGAGTGTGGTAAGAGAATGGCACTGTTAGAAATTCTATTCCACTCAACTTTTAAGATCACATGCAAAACGTGCAATATTGATGATCTGGAATTATCGGATGATGAATTTGGGGCCAAGTTATATAGCAATCTGCAGCGTATTGAAGAAAAGCAACGTGAATATCTCGCTAAAGATCAAAAACTTCTACGCATGATACACTTTGTAAAGGATCGGTGTAACCCAAAATTTTCACATTTGCCTTTACTATGGCAAGTGGCGGAAACAGTAGGGCATTACACCGACAACCAATCAAAGCAGATAATTGACATCAGTGAGGCGCTTATCAAAGTTAATACTTTAACTCCTGATGATGCAGTGAAGGCCAGTGTAGCATTACTAGAAGTAGCACGGTGGTATAAAAATCGAAAGGAATCACTTAAAACGGACACACTGGATTCATTTCGAAACAAGATTTCTCCAAAGAGCACGATCAACGCAGCATTAATGTGTGATAACCAGTTAGATAAGAATGCAAATTTCGTATGGGGAAACAGAGAATACCATGCAAAGCGATTCTTCGCTAATTATTTTGAAGCCGTGGACCCAACTGATGCATATGAAAAACACGTAACACGCTTCAACCCCAATGGACAGCGGAAATTATCGATTGGCAAGCTAGTAATTCCACTAGATTTCCAGAAGATCAGAGATTCGTTCGTTGGCCTATCAATAAATAAACAACCACTGAGCAAAGCTTGCGTAAGCAAAATTGATGGAGGCTACGTATATCCATGTTGCTGCGTTACAACGGAGTTTGGAAAACCAGCATATTCTGAGATAATACCTCCAACGAAAGGACATATCACGATTGGAAACTCAGTGGACCCAAAAATAGTGGATTTACCGAACACTACACCACCAAGTATGTACATTGCAAAAGATGGATACTGTTACATTAACATATTCTTGGCAGCAATGATAAACGTCAATGAGGAATCCGCAAAAGATTACACTAAGTTTCTTAGAGACGAGTTGGTGGAACGGCTTGGTAAATGGCCAAAATTGAAAGATGTAGCTACAGCATGTTATGCGCTATCTGTGATGTTTCCTGAAATCAAGAACGCTGAGTTACCACCGATATTAGTTGACCATGAGAGCAAATCGATGCACGTAATAGATTCGTATGGTTCGCTGAGTGTGGGGTTTCACATCCTCAAAGCAAGCACCATTGGACAGTTGATAAAATTCCAATATGAATCGATGGAAAGCGAAATGCGAGAATACATAGTAGGAGGCACTTTAACACAACAAACTTTCAACACTCTACTTAAAACTCTCACAAAGAACATGTTCAAACCTGAGAAAATTAAGCAGATAATTGAGGAAGAGCCTTTCTTACTGATGATGGCAATCGCATCACCAACTGTGCTCATATCTTTATACAATAATTGTTACATAGAGCAAGCTATTACATACTGGATTGTTAAAAATCAAGGTGTTGCAGCAATATTCGCACAGCTCGAAGCATTAGCCAAAAAGACGTCTCAAGCTGAATTATTAGTTTTACAGATGCAAATACTTGAAAAGGCGTCTAATCAGCTGAGGCTTGCAGTGACAGGACTAAACCATGTTGACCCCGCTAAGCGTCTTTTATGGTCACATCTTGAAGCTATGACAACGCGATCGGAAATGAACAAGGAACTGATAGCAGAAGGCTATGCTTTATATGACGAGCGCTTGTACACTTTGATGGAAAAAAGTTACGTAGATCAATTAAACCAGTCATGGGCAGAGTTATCATACTGTGGAAAATTTTCAGCAATATGGCGTGTGTTCAAAGTCAGGAAATATTACAAGCCGTCTTTAACCGTGAGAAAAAGCGTAGATTTAGGCGCTGTATACAATATATCAGCTACGCATCTAATATCAGGTTTAGTGCAGAAAAGTCGCGATCAAGTCAGCTCTACTTTAACCAAACTCCGCAACGGTTTTTATGATAAATTAGAGAAGACTAGAGTGCGTGCAATCAAAACAGTATATTGGTTTATACCCGATATATTTAGACTTGTTCACATTTTTATAGTGCTAAGTTTGTTAACCACCATAGCTAACACGATCGTAATAACTATGAATGACTATAAAAAGTTGAAAAAGCAACAAAGAGAGGATGCGTATGAGGCTGAGATAAACGAAGTTCGCAAGATTCATGCCGCCTTGATGAAGGAGCATAACGACAATTTAACATGCGAACAGTTCATTGAACATATGCGTCAAACACATCCACGATTAGTTGAAGCCACATTGGAGTTGACCCACACAGGTGTGATTCATGAAGGAAAGTCTAATCTAGAAACCAACCTTGAGCAAGCTATGGCTGTGGGAACACTACTCACGATGATACTTGATCCTCAGAAGAGTGACGCTGTGTACAAAGTTCTAAACAAAATGCGAACAGTGATTAGTACATTTGAACAGAATGTCTCATTCCCTTCAATTAACTTCACTAACATCTTGACACCACCCGTAACACAGCAGAGTGTGGACGTCGATGAGCCTCTTACTTTGAGCACTGACAAGAACTTAACGATAGATTTTGATACAAATCAAGATTTACCTGCAGACACTTTCAGTAACGATGTTACATTCGAGGATTGGTGGGCAAATCAGATAAGCAATAATAGAACAGTTCCACACTATCGTCTCGGTGGTAAGTTTGTAGAATTTACAAGGGAAAACGCAGCACTTGTTAGTATTGAATTAGCTCACTCGAACATTGAAAAGGAATTTCTTTTAAGGGGTGCGGTTGGCTCAGGAAAGTCCACAGGGCTGCCATACCATCTTAGTGCGCGTGGAAAAGTACTTCTAATCGAACCAACAAGACCACTTGCCGAGAATGTATGCAGACAGCTACAAGGGCCACCATTCAATGTTAGCCCGACACTTCAAATGCGTGGGTTGAGCTCTTTTGGCTGCACGCCAATCACGATCATGACCTCTGGTTTTGCATTACATATGTATGCAAACAATCCGGACAAGATATCTGATTACGACTTCATCATATTTGATGAATGTCATATCATGGAAGCACCAGCTATGGCATTTTATTGTTTGCTGAAGGAATATGAATATCGAGGCAAAATCATTAAAGTCTCCGCAACACCACCTGGAAGAGAATGTGAATTTACCACACAACATCCAGTTGACATTCACGTCTGCGAGAACCTCACACAACAGCAATTTGTAAGGGAACTTGGCACGGGTTCAAATGTCGACGCAACGAAATATGGAAATAACATACTTGTTTACGTTGCTAGCTACAATGACGTCGATTCGCTATCACACGCGCTAACTGAATTACATTATTCAGTTATCAAAGTTGACGGTAGAACTATGAAGCAGAACACCACAGGCATTGTAACTAATGGTACATCACAAAAGAAGTGTTTTGTCGTTGCAACGAACATAATCGAGAATGGTGTTACATTGGATGTTGATGTCGTAGTGGACTTTGGACTAAAAGTCACAGCTGAGCTGGATGTTGACAATAGAGCGATTCTATACAAACGTGTCAGCATCTCGTATGGTGAGCGTATTCAAAGACTCGGTCGTGTTGGAAGGAATAAACCCGGAACAGTGGTCCGCATTGGGAAAACTATGAAAGGTCTGCAGGAAATCCCAGCGATGATAGCAACGGAAGCTGCTTTTATGTGTTTTGCATACGGACTCAAAGTTATAACACACAATGTATCTACAACACACTTAGCTAAATGCACGGTAAAACAAGCAAGAACAATGATGCAATTTGAACTATCACCATTTGTAATGGCAGAATTAGTTAAATTCGATGGTTCGATGCATCCACAAATTCATGAAGCACTCGTAAAATACAAACTTAGAGATTCTGTGATAATGCTAAGACCAAATGCTATCCCGAAGGTCAACTTTCACAACTGGCTGACTGCACGTGATTATAACAGGATGGGCTGTGCAGTAGAACTTGAAGATCATGTGAAAATACCGTACTATATACGAGGGATTCCTGACAAACTATATGGAAAATTGTACGATATCATTTTACAATACAGCCCAACTAGTTGTTATGGAAGACTATCAAGTGCCTGTGCGGGAAAAGTTGCATATACCCTGCGCACTGATCCTTGTTCGTTACCACGCACGATAGCTATCATTAATGCCTTAATTACTGAAGAGTATGCAAAGAGGGACCATTACAGAAACATGATAGCAAACCCCTCATCATCGCACGCCTTTTCACTCAATGGGCTGGTATCCATGATCGCTTCTCGGTATATGAAAGACCACACGAAGGAAAACATTGACAAGCTTGTAAGAGTGCGCGACCAACTACTTGAGTTCCAAGGCACAGGTATGCAATTTCAAGATCCTTCAGAATTGATGGACATTGGTGCATTAAACACAGTTATTCACCAAGGAATGGACGCCACGGCTGCTTGTATTGGATTGCAAGGGCGCTGGAATGCTTCGCTCATTCAACGCGACTTGATGATATCAGCAGGGGTCTTCACAGGAGGAATTCTCATGATGTGGTATCTTTTCACAAAATGGAGCAAGACAGAAGTGTCACACCAAGGAAAGAACAAACGCAGCCGGCAAAAACTACGATTCAAAGAGGCACGTGATAATAAATATGCCTATGACGTAATAGGATCAGAGGAAGTTCTTGGTGAGAATTTTGGAACCGCATACACTAAGAAAGGAAAAGGAAAGGGGACAAAAGTTGGCCTTGGAGTCAAACAGCACGAATTCCACATGATGTACGGGTTTGATCCACAAGAATATAACTTAATCCGCTTCGTGGATCCTCTAACAGGCGCGACACTAGACGAGCAAATTCATGCAGATATACGTTTAGTGCAAGAGCACTTTAGCGTCATTAGAGATGAAGCAGTGGCAAATGACACAATTGAAAGACAACACATTTACAGCAATCCTGGACTACAAGCGTTCTTCATACAAAATGGATCTGCAAATGCACTGAGAGTTGATTTAACACCACATACACCACTGCGTGTCATGACCAATAACAATATAGCAGGTTTCCCGGAATATGAAGGTACACTTCGACAAACAGGGACAGCTTTACAGATACCCGTGAATCAAGTTCCAGCTGCGAATGAAGCGGGGGTAGCACATGAGTCAAAATCGATGATGGCAGGGTTAGGCGACTACACCCCAATATCACAGCAGTTGTGTCTAGTCCAGAATGACTCAGATGGAATCAAAAGGAATGTGTACTCAATTGGATATGGATCATATCTCATTTCGCCGGCACACTTATTTAAATATAACAATGGCGAAATAACAATTAAATCTTCAAGAGGTTTGTATAAGATCAGAAATTCAGTAGAACTCAAGTTGCATCCCATTGCACATAGAGATATGGTCATTATTCAACTTCCGAAAGATTTCCCGCCGTTCCCAATGCGTCTCAAGTTTTCTAAACCATCTAGAGAGTCAAGAGTGTGCTTAGTTGGAGTGAACTTCCAACAAAACTACAGCACATGCATTGTATCGGAGAGTAGTGTCACAGCACCAAAAGGCAATGGAGATTTCTGGAAACACTGGATATCCACAGTGGACGGACAATGCGGCCTCCCATTAGTAGATGTCAAGAGCAAGCACATAGTTGGAATACACAGCCTTGCATCAACTAGTGGAAACACTAACTTTTTCGTCGCCATGCCTGAAGATTTCAATGACTACATTCACAATCTTGTGCAAACCAACAAGTGGGAAAAAGGATGGCATTACAACCCAAATCTCATTTCATGGTGTGGTCTCAATCTAGTTGATTCAGCTCCAAAGGGTCTCTTCAAAACTTCGAAATTAGTGGAAGACCTTGACATGAGTGTTGAAGAGCAGTGCAAGGTTACAGAGACATGGCTCACAGAATGCATTCAGGACAATTTACAGGTTGTCGCAAAATGCCCAGGCCAACTTGTCACCAAGCACGTTGTCAAAGGCCCATGCCCACACTTTCAGCTATATCTATCAACACATGATGAAGCCAAAGCATACTTCGCACCACTACTCGGAAAGTACGATAAGAGCAGATTGAACAGAGCAGCTTTTATCAAAGACATTTCAAAATATGCAAAACCAATCTACATTGGAGAAATCAATTACGATGTCTTTGAAAAGGCTATAGAACGTGTGATTAAAATCCTTAGAGATGTGGGAATGCAACAATGCGCGTATGTCACGGATGAGGATGAAATATTCCAGTCACTCAACCTCAACGCCGCTGTTGGTGCCTTATACACAGGAAAGAAGAAGGACTATTTTAAGGATTTCTCAAATGAAGACAAATCAGAAATCATTATGAGATCCTGTGAGCGTATCTACAACGGACAACTTGGTGTGTGGAATGGTTCACTCAAAGCTGAAATAAGGCCCATAGAGAAAACTATGTTAAACAAGACTCGAACCTTCACAGCAGCACCATTAGAAACTCTACTTGGTGGCAAAGTCTGCGTCGATGATTTCAACAATCAGTTCTACTCACACCACTTAGAGGGTCCTTGGACAGTTGGAATTACAAAGTTCTATGGTGGGTGGAATCGTTTATTAGAAAAATTACCAGACGGTTGGGTATACTGCGACGCTGATGGATCACAGTTTGACAGCTCTTTGACACCATACCTTATCAACGCTGTATTACATATTCGATTACAATTCATGGAGGAATGGAACTTAGGAGAACAAATGTTGCGAAACTTGTACACCGAAATCGTATACACACCAATTGCAACACCAGATGGATCTGTAATTAAGAAATTCAAAGGAAATAACAGTGGGCAGCCGTCAACAGTTGTAGACAACACACTCATGGTGATATTAGCGTTTAATTATGCAATGTTGTTGAGTGGCGTTAAAGAGGAAGAAATAGATAACTGTTGCCGAATGTTCGCCAATGGTGATGATCTGTTGCTTGCAGTGCACCCAGACTTCGAACATATATTGGATGGATTTCAAAATCACTTTGGAAACTTGGGTCTCAACTTTGAGTTCACATCACGAACAAGAAGCAAGTCAGAGTTATGGTTCATGTCCACACGAGGTATCAAATGCGAAGGCGTCTATATACCAAAGCTTGAGAAAGAAAGAATAGTTGCCATACTCGAGTGGGATCGGTCAAACTTACCTGAGTACCGTCTCGAAGCCATTTGTGCAGCCATGGTAGAGGCATGGGGATACCCAGACCTTGTCCAAGAGATACGGAAATTTTATGCGTGGCTTCTCGAAATGCAACCATTTGCAAATTTAGCTAAAGAGGGCCTAGCACCATATATCGCTGAAACTGCACTCAGAAATCTGTATTTGGGCACAGGGATCAAGGAAGAAGAGATTGAGAAATATTTCAAACAGCTTGCCAAAGATCTCTCTGGTTATATAGAAGATTACAATGAAGATGTTTTCCATCAATCCGGAACTGTGGATGCAGGAGCCCAAGGAGGGGGAAGTGGATCTGGAACAACGCCACCAGCAACA---------------------------------------------------GGTAGTGGAAC----AGGAACAAGAACACCATCTACTGGAACTCCAGCACAAGGCAACACACCTCCAG---CATCAGGTGGATCATCAGGAAACAATGGAGGC---GGTCAATCAGGTTC---AAACGGCACTGG------------AGGCCA---AGCAGGCTCAAGCGGA------------GCAGGGGGTCAAAGAGACAAAGACGTTGACGCTGGCTCAACAGGGAAGATATCAGTGCCAAAGCTTAAGGCAATGTCGAAGAAAATGCGCCTGCCAAAGGCGAAAGGAAAAGACGTTTTACACTTAGATTTCCTGTTAACATATAAACCACAACAGCAAGATATATCAAACACTAGAGCAACTAAGGAAGAGTTTGATAGATGGTACGATGCCATAAAGAAGGAGTACGAAATTGATGACACACAAATGACAGTTGTCATGAGTGGTCTCATGGTATGGTGCATCGAAAATGGTTGCTCACCAAACATAAACGGAAATTGGACGATGATGGACGGAGACGAACAAAGGGTTTTTCCATTAAAGCCAGTCATTGAGAACGCATCTCCAACTTTCCGACAGATAATGCATCATTTTAGTGATGCAGCTGAAGCGTATATAGAGTACCGAAACTCTACAGAGCGATACATGCCAAGATATGGTCTTCAGCGAAATCTCACCGACTATAGCTTAGCGCGGTATGCTTTCGATTTCTATGAAATGACTTCGCGCACACCAGCTAGAGCTAAGGAAGCCCACATGCAG---ATGAAAGCCGCAGCAGTTCGTGGTTCAAACACACGTCTGTTCGGTCTGGACGGAAATGTCGGCGAGACTCAGGAGAATACAGAGAGACACACAGCTGGCGACGTTAGTCGCAACATGCACTCTCTGTTGGGAGTGCAGCAGCACCACTAGTCTCCTGGAAACCCTGTTTGCAGTACCTATAGTA--TATTA------CTAAATAGTACGTTCGTGAGGCCTTGCCTCGTGTGTATGTGAGGTTCTACCTCGTATTTACTATTTCAGTA--TATGTACTTTTAGCGTGAGCCAGTCTGCAGGACACAGGGTTGGACCCAGTGTCTTCTGGTGTAGCGTGTACTAGCGTCGAGCCAATGGACGGACGGCACTGGGAGTGGTTTTACCATTGGTGCTGCGAGTCTCTTGGTGAGAGAC----------------

>JX047393

---------------------AAAACAACAAAACTCAACACAACACAACAAAACACAACCAAGCAAATCCAAGTTTCCTTTACTCAGATTGTAGTGAACGGCTCGGTAGGAAAGGTTCCTCGAGATCACTCTCTGATTCTTCTCTC----------TCAACCAACTTCATTCAAGCGAGATGGCGGGCTCTTGGACTCACGTGACATACAAGTGGCAACCAGATGTCAACAACGCACGTGATGTGAAAAGAGTGATGGAGATGTTTGCAGCAAAACATCAACATTACACTGAGGAGCAAAGGCTTGCTCACAACAGCAAGCTATTAAGGAAGGCTTGTGTCACTAACGCTGAGTTTATTGAACCAGCACAGAAACCAAAATGTCACCAGACATGGGTTGAAAAGTGCGACCACAACCCCACAGAGCACTTTGTTTATCAACGCTCT---ACACCTGAGAAGAAAGTGCTTAGCACCAAACCTAAGACAACCTCTGTTACGAAGTTAATCAGGGATGTCCTTGAGATTTCGAAGGGCAGTGGGATAAAAATTGAGTTAATTGGCAAGCGTGTTAAACGTAAGACTCAATTGTCCATAAGGCAACACAATGGCAAAGACTTCTTGCACTGCAAAACCAGGCATGAGAATGGCCTGTTTAAACGCAAGGACATTGACATTAATGTCAAGTGGTTGCCCACCATTGAAGCCATTGCAAAATGCTACAGTACGGTGAATGCGGAAGAACTGCAAAGTCTCAATAGAGGCAGTAGTGGTCTTACATTCATGCAAAACGATGAATTGTTCATCGTGCGTGGAAGGATGCATGGTGAGATTGTCAATAGTTTACACGAAAATAAGCACGTTATGGAAATTGAACACTATGCTGATCCACAAGCAAACAGTTTCTGGAAAGGTTACACAGATGCATATGTCGAGAATAGAAACATATCTACCACTCACACAGAGCACACACCAACTATTAATTTAGAAGAGTGTGGCAAGAGAATGGCACTCTTAGAAATCTTATTCCATTCAACTTTCAAAATAACATGCAAAACGTGCAACATTGATGACCTCGAATTATCAGATGATGAATTTGGGGCCAAGTTATACAGTAATCTGCAACGCATTGCAGAAAGGCAACGTGAATATCTTGCTAAAGACCAAAAACATTTACGCATGATTCACTTTGTGAAGGACCGATGCAATCCAAAATTTTCGCACTTGCCCTTGCTATGGCAAGTAGCAGAAACAGTAGGACATTATACTGATAACCAATCGAAGCAGATAATTGATATCAGTGAGGCGCTCATAAAAGTTAATACCTTAACTCCTGATGATGCAGTAAAGGCCAGTGTAGCACTATTGGAAGTTGCACGATGGTATAAAAATCGGAAGGAATCACTCAAAACAGACACACTAAACTCATTCCGAAACAAAATTTCACCAAAGAGCACGATCAACGCAGCATTGATGTGCGACAATCAATTAGATAAGAATGCTAATTTCGTATGGGGAAACAGAGAATATCATGCAAAGCGATTCTTTGCCAATTATTTTGAAGCTGTAGACCCAACTGATGCGTATGAAAAACATGTCACACGCTTCAACCCCAATGGACAGCGGAAATTATCAATTGGCAAGTTAGTAATTCCACTAGATTTCCAGAAAATTAGAGATTCGTTTGTTGGCTTATCGATAAATAAACAACCATTGAGCAAAGCTTGTGTGAGCAAAATTGATGGAGGCTACGTATATCCATGTTGCTGCGTTACAACGGAATTCGGAAAACCAGCATATTCTGAGATAATACCTCCAACAAAAGGACATATCACGATTGGAAATTCAGTGGACCCAAAAATAGTAGATTTGCCAAACACAACACCACCGAGTATGTATATCGCAAAAGATGGATATTGTTATATTAACATATTCTTAGCAGCAATGATAAACGTCAATGAGGAATCCGCGAAAGACTACACGAAGTTCCTTAGAGACGAGTTGGTGGAACGGCTCGGCAAGTGGCCTAAATTGAAAGATGTAGCAACAGCATGTTACGCTTTATCAGTAATGTTCCCAGAGATCAAGAATGCCGAATTGCCACCAATATTGGTTGATCATGAGAGCAAGTCAATGCATGTCATTGATTCATATGGATCACTCAGTGTTGGATTCCACATTCTAAAGGCAAGTACCGTGGGACAACTGATAAAATTCCAGTACGAATCGTTAGAAAGCGAGATGCGTGAATACATAGTGGGAGGTACCTTGACACAACAAACTTTTAACACACTTCTCAAGACTCTCACAAGGAACATGTTCAAGCCAAATAAAATTAAACAGATAATAGAGGAAGAACCTTTCCTACTAATGATGGCGATTGCATCCCCTACCGTGCTTATCTCACTGTATAACAATTGCTACATCGAACAGGCGATGACATACTGGATTGTCAAGAACCAAGGAGTCGCGGCAATTTTTGCACAGTTGGAGGCTTTGGCAAAGAAAACCTCCCAAGCAGAACTATTAGTTCTGCAAATGCAAATACTCGAAAAAGCTTCAAACCAGCTAAGACTTGCAGTCACCGGACTTAATCATGTCGATCCAGCGAAACGACTCCTGTGGTCTCACCTGGAAGCCATGACAACGCGATCGGAAATGAATAAGGAACTCATAGCGGAAGGTTATGCACTATATGATGAGCGTCTATATACCCTAATGGAAAAAAGTTATGTAGATCAATTAAACCAGTCATGGGCAGAATTATCATACTGTGGAAAATTTTCAGCAATATGGCGTGTGTTCAGAGTCAAGAAATACTACAAGCCATCTTTAACCGTGAGAAAAAGCGTAGATTTAGGCGCTGTTTACAATATATCAGCTACGCATCTAATATCAGATTTAGTGCAGAGAAGTCGAGATCGAGTCAGCTCTACTTTAACCAAACTCCGCAACGGTTTTTATGACAAAATGGAAAAAGCGAGGGTTAGCGCAGTGAGGACTGTATATTGGTTCATACCCGATATATTTAGACTAGTCCACATCTTTATAATTTTAAGTTTATTAACTACTATAGCCAATACAATAGTTACAACCATGAATGATTACAAAAAGTTAAAGAAGCAACAAAGAGAGGACGAGTATGAGGCCGAAATTAATGAGGTGCGGAAAATACATGCCAATTTGATGAAGGAGCACAATGACAATCTGACATGTGATCAGTTTATTGAATACATACGCCAAACACACCCACGCCTCATTGAAGCAACTTTGGACTTGACACATACAGGCGTCATTCACGAGGGTAAATCCAACTTGGAAACAAACCTTGAACAGGCGATGGCAGTGGGAACTTTACTCACTATGATACTCGACCCACAAAAGAGCGATGCAGTTTACAAGGTTCTCAACAAAATGCGGACAGTGATTAACACAATCGAACAGAATGTGCCATTCCCAACAGTCAATTTTACAAGCATCTTAACGCCTCCTGTAACACAACAAAGTGTTGATGTTGATGAACCATTGACACTAAGTACTGATAAGAATTTGACTATAGATTTTGATACAAATCAAGACTTGCCAGCGGACACATTTAGTAATGACGTTACATTTGAGGATTGGTGGGCTAATCAGATTAACAACAACAGAACAGTGCCACACTACCGACTTGGGGGAAAATTTGTAGAATTCACAAGAGAAAATGCAGCAATGGTCAGCATTGAACTCGCTCACTCAAACATTGAAAAAGAATTCTTACTCAGAGGAGCTGTTGGGTCAGGAAAATCCACAGGCTTACCATACCACCTCAGTATGCGTGGAAAAGTGCTATTGATAGAACCCACTCGACCATTAGCTGAAAACGTTTGCAGGCAATTGCAAGGACCTCCATTTAACGTGAGCCCCACTTTACAAATGCGAGGTTTGAGCACATTTGGCTGCACTCCCATCACGATAATGACGTCTGGATTCGCATTGCATATGTATGCTAACAACCCTGACAAGATCTCTGAATACGACTTCATCATTTTTGATGAATGTCATATCATGGAAGCACCTGCAATGGCATTCTATTGTTTGCTTAAGGAGTATGAATACCGAGGAAAGATAATAAAAGTTTCAGCAACACCACCAGGACGAGAATGTGAGTTTTCAACCCAACATCCAGTAGATATACATGTATGTGAGAGCTTGACACAACAGCAATTCGTCATGGAGCTTGGAACAGGGTCAACCGCTGATGCAACCAAGTACGGTAATAACATATTAGTGTACGTTGCAAGTTACAATGACGTAGATTCTTTATCCCATGCTCTAACCGAGCTCAAGTATTCAGTAGTCAAAGTTGATGGAAGAACAATGAAGCAGAACACCACAGGAATTGTAACTAATGGAACATCCAGTAAGAAATGCTTCGTTGTAGCTACAAACATCATTGAGAATGGTGTGACGCTAGACGTTGATGTCGTTGTCGACTTTGGACTTAAAGTAACAGCCGAACTGGATGTTGATAACAGGGCAATAATGTATAAACGTGTGAGCATATCTTATGGCGAGCGTATTCAGAGACTTGGAAGAGTTGGAAGGAACAAGCCCGGAACAGTTATCCGCATCGGGAAAACAATGAAAGGTCTGCAAGAAATTCCAGCGATGATCGCCACTGAAGCGGCTTTCATGTGTTTCGCATATGGACTGAAGGTCATAACGCACAATGTATCAACAACACATTTGGCAAAATGCACCGTTAAACAAGCCAGAACTATGATGCAGTTTGAGCTTTCACCATTTGTAATGGCTGATCTAGTAAAGTTTGATGGTTCTATGCACCCACAAATCCATGAAGCATTAACTAAGTATAAATTACGAGATTCTGTAATTATGCTAAGACCAAACGCAATACCAAAGGTAAATCTTCACAATTGGTTAACGGCTCGTGATTACAACAGAATAGGCTGCTCATTAGACCTTGAAGATCATGTTAAAATACCATATTACATACGAGGAGTTCCTGACAAGTTATACGGGAAGTTGTATGAAATTATCCTTCAATACAGCCCTACAAGTTGCTATGGAAGGCTATCAAGCGCTTGTGCAGGTAAGGTAGCATATACACTGCGCACTGATCCTTGTTCGTTACCAAGAACAATAGCTATAATCAACGCATTAATCACTGAAGAGTATGCAAAGAGGGATCATTACAGAAACATGATAGCGAACCCTTCGTCATCGCACGCCTTTTCACTTAATGGGCTGGTATCCATGATCGCTTCTCGATACATGAAAGATCACACAAAGGAAAACATCGATAAACTTGTAAGAGTGCGCGACCAGCTACTTGAATTCCAAGGCACAGGTATGCAATTTCAAGATCCTTCAGAATTGATGGACATTGGTGCTTTAAACACAGTTATTCACCAAGGAATGGACGCTACGGCTGCTTGTATTGGACTGCAAGGGCGCTGGAATGCTTCGCTCATTCAGCGCGATTTGATGATATCAGCAGGGGTCTTCACAGGAGGAATTCTCATGATGTGGTATCTTTTCACAAAATGGAGCAAGACAGAAGTGTCACACCAAGGAAAGAACAAGCGCAGTCGGCAAAAGCTACGATTCAAAGAGGCTCGTGATAATAAATATGCCTACGATGTAGTAGGATCAGAGGAAGTTCTTGGTGAGAATTTCGGAACTGCATACACTAAGAAAGGAAAAGGAAAGGGGACAAAAGTTGGCCTTGGAGTCAAACAGCACAAATTTCACATGATGTACGGGTTTGATCCACAAGAATACAACTTAATCCGCTTCGTGGATCCTTTAACAGGTGCGACACTAGACGAGCAAATCCATGCAGATATACAGTTAGTGCAAGAGCACTTCAGCGTCATCAGAGATGAAGCAGTAGCAAATGACACAATTGAAAGACAGCACATTTACGCTAATCCTGGATTACAAGCTTTCTTCATACAAAATGGATCGGCAAATGCACTGAGAGTTGATTTGACGCCGCATTCACCCTTGCGTGCCGTGACCAATAACAATATAGCAGGCTTCCCAGAGTATGAAGGTACACTTCGGCAAACAGGAACAGCTCTACAAATACCCGTGAATCAAGTTCCAGCTGCGAACGAAGCGGGGGTGGCACATGAATCGAAATCGATGATGGCAGGGCTAGGTGACTACACTCCAATATCACAGCAATTGTGCCTAGTTCAAAATGACTCAGATGGAATCAAAAGGAATGTATATTCAATTGGATATGGATCATATCTCATTGCGCCAGCACATTTATTTAAGTATAATAATGGCGAAATAACGATAAAATCATCACGAGGCTTATACAAAATCAGAAATTCAGTCGAAATCAAGTTGCATCCCATTGCGCATAGAGACATGGTTATAATTCAACTTCCAAAAGATTTTCCACCATTCCCAATGCGTCTTAAGTTTTCAAAACCATTTAGAGAGTCAAGGGTGTGCTTAGTCGGAGTGAATTTTCAACAGAACTATAGCACATGTATCGTATCGGAGAGTAGCGTCACAGCACCAAAAGGAAACGGAGATTTCTGGAAACACTGGATATCCACAGTAGACGGACAATGCGGTCTCCCATTAGTAGATGTCAAGAGTAAGCACATAGTTGGAATACACAGTCTTGCATCAACTAGTGGAAATACTAACTTCTTTGTCGCCATGCCTGAAGACTTCAATGACTACATCAATAATCTTGTGCAAACCAACAAGTGGGAAAAAGGATGGCATTACAACCCAAATCTCATTTCATGGTGTGGTCTCAACCTAGTTGACTCAGCTCCAAAGGGTCTTTTCAAAACTTCAAAATTAGTAGAAGACCTTGATATGAGCGTTGAAGAACAATGTGAGGTAACAGAGACATGGCTCACGGAGTGTATTCAGGATAATTTACAGGTTGTTGCAAAATGTCCAGGCCAACTTGTCACCAAGCACGTTGTCAAAGGCCCATGCCCACACTTTCAGCTATACCTGTCAACACATGATGAAGCTAAAGCATACTTTGCACCATTACTCGGAAAATACGATAAGAGCAGATTAAACAGAGCAGCTTTTATCAAAGACATTTCAAAATATGCAAAACCAATCTACATTGGAGAAATCAATTACGATGTCTTTGAAAAGGCTATACAACGTGTAATTAAAATTCTTAGAGACGTGAGAATGCAGCAATGCACGTATGTAACGGACGAGGATGAAATATTCCAGTCACTTAACCTCAACGCTGCAGTTGGCGCCTTATACACAGGAAAGAAGAAAGATTATTTCAAGGATTTTTCAAATGAGGACAAATCAGAAATTATCATGAGATCCTGTGAGCGTTTATACAACGGACACCTTGGCGTGTGGAATGGTTCACTCAAAGCTGAAATAAGGCCTATAGAGAAAACAATGTTAAATAAGACTCGGACTTTTACAGCAGCACCATTAGAAACTTTACTTGGTGGTAAGGTTTGTGTCGATGATTTCAACAACCAATTCTACTCGCACCACTTAGAAGGTCCTTGGACAGTTGGAATAACAAAGTTTTATGGTGGGTGGAACCGTTTGTTGGAAAAATTGCCAGATGGTTGGATATACTGCGACGCCGATGGATCACAGTTCGACAGCTCTTTGACACCATATCTCATCAACGCCGTATTACACATTCGATTACAATTCATGGAAGAATGGAACTTAGGAGAACAAATGTTGCGAAACTTGTACACTGAAATCGTGTACACACCAATTGCAACACCAGATGGATCTGTAATCAAGAAATTTAAAGGAAATAACAGCGGGCAGCCGTCAACAGTTGTAGACAACACACTCATGGTGATATTAGCATTTAATTATGCAATGTTATCAAGTGGTGTTAAAGAGGAAGAAATAGACAATTGCTGCCGAATGTTCGCCAATGGTGATGATTTGCTACTTGCAGTGCATCCGGATTTCGAACACATATTGGACGGATTTCAAAATCACTTTGGAAACCTAGGTCTCAACTTTGAGTTTACATCACGAACAAGAGACAAGTCAGAGTTATGGTTCATGTCCACACGAGGTATCAAATGCGAAGGCATCTATATACCAAAGCTTGAGAAAGAAAGAATAGTTGCCATACTCGAGTGGGATCGGTCAAACCTACCTGAGCACCGTCTCGAAGCCATTTGTGCAGCCATGGTAGAGGCATGGGGATACCCAGACCTTGTCCAAGAGATACGGAAATTTTATGCGTGGCTTCTCGAAATGCAACCATTTGCAAATTTAGCAAAAGAGGGCCTAGCACCATATATCGCTGAAACTGCACTCAGAAATCTGTATTTGGGCACAGGGATCAAGGAAGAAGAGATTGAGAAATATTTCAAACAGTTTGCCAAAGATCTCCCTGGTTATATAGAAGATTACAACGAAGATGTTTTCCATCAATCTGGAACTGTGGATGCAGGAGCCCAAGGAGGGGGAAGTGGATCTGGAACAACGCCACCAGCAACA---------------------------------------------------GGTAGTGGAAC----AGGAACAAGAACACCATCTACTGGAACTCCAGCACAAGGCAACACACCTCCAG---CATCAGGTGGATCATCAGGAAACAATGGAGGC---GGCCAATCAGGTTC---AAACGGCACTGG------------AGGCCA---AGCAGGATCAAGCGGA------------GCAGGGGGTCAAAGAGACAAAGACGTTGACGCTGGCTCAACAGGGAAGATATCAGTGCCAAAGCTTAAGGCAATGTCGAAGAAAATGCGCCTGCCAAAGGCGAAAGGAAAAGACGTTTTACATTTAGATTTCCTATTAACATATAAACCACAACAGCAAGATATATCAAACACTAGAGCAACTAAGGAAGAGTTTGATAGATGGTACGATGCCATAAAGAAGGAGTACGAAATTGATGACACACAAATGACAGTTGTCATGAGTGGTCTCATGGTATGGTGCATCGAAAATGGTTGCTCACCAAACATAAACGGAAATTGGACGATGATGGACGGAGACGAACAAAGGGTTTTTCCATTAAAGCCAGTCATTGAGAACGCATCTCCAACTTTCCGACAGATAATGCATCATTTTAGTGATGCAGCTGAAGCGTATATAGAGTACCGAAACTCTACAGAGCGATACATGCCAAGATACGGTCTTCAGCGAAATCTCACCGACTATAGCTTAGCGCGGTATGCTTTCGATTTCTATGAAATGACTTCGCGCACACCAGCTAGAGCTAAGGAAGCCCACATGCAG---ATGAAAGCCGCAGCAGTTCGTGGTTCAAACACACGTCTGTTCGGTCTGGACGGAAATGTCGGCGAGACTCAGGAGAATACAGAGAGACACACAGCTGGCGACGTTAGTCGCAACATGCACTCTCTGTTGGGAGTGCAGCAGCACCACTAGTCTCCTGGAAACCCTGCTTGCAGTACCTATAGTA--TATTA------CTAAATAGTACGTTCGTGAGGCCTTGCCTCGAGTGTATGTGAGGTTCTACCTCGTATTTACTATTTCAGTA--TATGTACTTTTAGCGTGAACCAGTCTGCAGGACACAGGGTTGGACCCAGTGTCTTCTGGTGTAGCGTGTACTAGCGTCGAGCCAATGGACGGACGGCACTGGGAGTGGTTTTACCATTGGTGCTGCGAGTCTCTTGGTGAGAGAC----------------

>JX047392

---------------------AAAACAACAAAACTCAACAACACACAACAAAACACAACCAAACAAAACCAAGTTTTCTTTGCTCAGATTGTAGTGAACGGCTCGCAAGAAACGGTTCTTCGAGATCACTCTCTGACTTCTTTCTC----------TCTCACACTTGCATTCAAGCGGAATGGCGGGATCGTGGACTCACGTGTCTTACAAGTGGCAGCCAAACGTCAACAATGACCGTGATGTAAGAAAGGTAATGGAAATGTTTGCAGCAAAACATCAACATTACACAGAGGAGCAGCGACTTGCACATAACAGCAAGTTGCTAAGGAAGGCGTATGTTGTGGACGTTGAACCAGTGAAGCCAGCACCGGAGCCTATAAGGCGTAAAGTGTGGGTAGAAAAATTCGATCACAACCCAACCGAAGATCTGGTGTATCCGCGCCTT---GTCACGGTTAAAAAGGCAGCAGAAATGAAGCCCGTGAACACCTCTATAAATAAACTTATAAGGGATGTTCTGGACATATCAAAAGGGAGCAGCCTTAAACTTGAGCTGATTGGTAAACGCCAAAAGTGCAAAACACAACTAGCTATTAAGAAATACAACAATAAGGACTACCTCCATTGCAGGACACGTCATGAAGACAACATGTTCAAGAGGAGGGACGTGGCAATCGGCATTGAATGGATCCCAACCATTGAAGCGATTGCTCGATGCTACAGCACAGTAAACAAACAGGAGATGCAAAGTCTCTACAAAGGCAGTAGTGGCTTAACATTCATGCAAAATAATGAATTATTCATTGTTAGAGGCAGAATGAATGGCGAGTTAGTTAATAGCCTGGAAGAGAACAGGAACGTGCTCGACATCGATCACTACGCTGATCCACAAGCGAACGACTTTTGGAAAGGGTACACTGATGCATACGTTGCGAATCGCAGTATCTCCACTACACATACCGTGCATACTCCAACAATCAACTTGGTAGAGTGTGGAAAGAGAATGGCATTACTTGAAATTTTATTCCACTCAACTTTTAAGATTACATGCAAGACATGCAATATTGATGACTTGGAGTTATCAGACGATGAATTTGGAGCAAAACTTTTCAAGAATCTACAACGTATCGAGGAACAGCAACGCGAATATCTCGCTAAAGATCAAAAGCTGCGTCGCATGATTCAATTCATCAAAGAAAGGTGTAATCCAAAGTTCTCTCATCTACCATTGCTTTGGCAAGTTGCAGAAACAATAGGACATTATACGGACAATCAGTCAAAGCAAATAATTGATATCAGTGAAGCACTCATCAAAGTGAACACATTAACACCTGATGATGCAGTCAAGGCGAGCGTAGCACTGCTTGAGGTGGCACGATGGTATAAAAATAGGAAAGAATCACTTAAAACAGACACATTAGATTCGTTCCGAAACAAGATATCCCCAAAGAGCACGATAAACACAGCATTGATGTGTGACAATCAACTAGACAAGAATGCAAATTTTGTCTGGGGAAACAGGGAGTATCATGCAAAGCGCTTCTTCTCAAATTACTTTGAAGCAGTAGATCCTACTGACGCATACGAGAAGCACGTAACCCGTTTCAATCCAAATGGTCAACGGAAATTATCAATTGGTAAACTAGTAATTCCGTTAGATTTCCAGAAAATAAGAGACTCATTCGTTGGTTTGGCAATAAACAAACAGCCACTTACAAAGGCTTGTGTGAGCAAAATCGATGGTGGTTATGTATACCCATGCTGTTGTGTCACAACAGAATTTGGAAAACCAGCATACTCTGAGATAATACCACCAACAAAGGGCCATATAACAATTGGAAATTCTGTAGACCCAAAGATCGTAGACCTGCCAAATACAACACCACCAAGTATGTACATTGCCAAAGACGGATATTGTTACATCAACATTTTCTTAGCAGCAATGATAAATGTCAATGAAGACTCAGCAAAGGACTACACAAAATTCCTCAGGGATGAATTAGTCGAGCGACTTGGAAAGTGGCCGAAACTTAAAGATGTTGCAACAGCGTGCTATGCGTTATCAGTTATGTTTCCAGAGATTAAGAACGCTGAATTGCCTCCAATACTCGTTGACCACGAAAGCAAGTCAATGCACGTGATCGATTCATACGGCTCATTGAGCGTTGGATTCCACATCCTTAAAGCAAGCACTGTTGGCCAGCTCATAAAATTTCAATACGAGTCAATGGATAGTGAGATGCGCGAGTATATAGTGGGAGGTACTTTAACGACCCAAACATTTAACAAACTCCTTACCTCTTTAGCTAAAAATATGTTCAAACCAGATCAAATTAAGCAGATGATTGAGGAGGAACCCTTCCTACTAATGATGGCAATCGCGTCACCAACTATGCTCATAGCGCTATACAATAATTGCTATATAGAGCAGGCCATGACGTATTGGATCGTTAAAAACCAAGGAGTGGCAGCGATATTCGCACAACTGGAAGCACTAGCAAAGAAGACCTCACAAGCTGAATTGCTAGTCCAACAAATGCAAATACTTGAAAAGGCGTCTAATCAATTGAGGCTTGCAGTCACGGGTTTAAATCATGTCGATCCAGCCAAGCGTCTGCTATGGTCGCATCTAGAAGCAATGACAACACGATCAGAGATGAACAAGGAATTAATTGCTGAAGGCTATGCCCTGTATGATGAGCGCCTGTATGCACTTATGGAAAAAAGTTACGTAGATCAATTAAACCAATCATGGGCAGAGTTATCATTCTGTGGAAAATTTTCAGCAATATGGCGTGTGTTCAAAGTCAAGAAATATTACAAGCCGTCTTTAACCGTGAGAAAAAGCGTAGATTTAGGCGCTGTATACAATATATCAGCTACGCATCTAATATCAGATTTAGCGCAGAGAAGTCGCGATCGAGCCAGCTCTATTTTAACCAAACTCCGCAACGGTTTTTATGATAAGTTAGAGAAGGCAAGAACTCGAGCAATTAAAACTGTTTATTGGTTCATACCTGACATATTTAGACTTATGCATATTTTCATAGTTCTTAGTTTATTAACAACTGTGGCAAACACTATTATTGTGACTATGAATGATTACAAAAAGCTGAAAAAGCAACAAAGAGAGGATGAGTATGAAGCTGAAATTAACGAGGTTCGAAAAATTCACGCTAATCTGATGAAAGAGCATAATGACAATCTAACATGTGAGCAGTTTATCGAACATATGCGCCAGATGCATCCACGATTGATTGAAGCCACATTGGAGTTGACACACACAGGCGTCATTCATGAAGGAAAGAGCAACCTAGAAACCAATCTCGAGCAAGCTATGGCAGTGGGCACTTTACTCACAATGATGTTAGACCCACAGAAGAGTGATGCAGTATACAAAGTGTTGAATAAGATGAGAACTGTAATTAGCACATTTGAGCAGAATGTCCCATTTCCTTCAATCAACTTTACAAATATTTTAACACCATCAGTGGCGCAACAAAGTGTAGATGTTGATGAACCTCTAACTTTAAGTACTGACAAGAATCTAACAATAGATTTTGATACTAATCAAGATTTACCAGCGGACACGTTTAGCAATGATGTTACTTTCGAAGATTGGTGGGCAAATCAAATAAGCAACAATAGAACAGTTCCACATTATCGGCTTGGTGGAAAATTTGCAGAATTCACAAGAGAAAATGCAGCACATGTTAGCATTGAGCTCGCACACTCAAATCTTGAGAAGGAGTTTCTACTCAGAGGAGCTGTTGGCTCAGGAAAATCCACTGGTCTTCCATATCATCTTAGTATGCGTGGTAAGGTGCTTTTAATAGAACCAACAAGACCACTAGCCGAGAATGTCTGCAGACAGCTTCAAGGACCACCATTCAATGTCAGCCCGACACTACAAATGCGTGGTTTAAGCTCTTTTGGTTGCACGCCAATTACGATAATGACATCAGGTTTCGCACTACACATGTATGCAAATAATCCGGATAAGATCTCTGACTACGATTTTATAATATTTGATGAGTGTCATATCATGGAAGCGCCAGCCATGGCGTTCTACTGTTTGTTGAAAGAATATGAATATAGAGGCAAAATCATTAAGGTGTCCGCAACACCACCAGGACGAGAGTGTGAGTTCTCTACTCAGCATCCAGTTGATATCCATGTGTGCGAAAATCTCACTCAGCAACAATTCGTTAGAGAGCTTGGTTCCGGATCAAACGTTGACGCAACAAAGTATGGAAACAACATACTTGTGTATGTTGCAAGCTACAACGACGTCGACTCATTAGCGCATGCTTTAACTGAATTACACTATTCAGTCATAAAGGTTGATGGGAGAACAATGAAACAGAACACTACAGGGATAGTGACAAATGGCACCTCTCAAAAGAAGTGTTTTGTCGTTGCTACGAATATCATTGAGAATGGTGTCACATTAGACGTTGATGTTGTGGTCGACTTCGGACTCAAAGTAACAGCTGAATTAGATGTTGACAACAGAGCTATTCTATACAAGCGTGTTAGCATTTCATATGGTGAGCGAATTCAAAGGCTAGGGCGTGTTGGAAGAAACAAACCCGGAACAGTCGTTCGAATTGGAAAAACCATGAAGGGTTTGCAAGAAATCCCAGCCATGATAGCGACAGAAGCAGCATTCATGTGCTTTGCATATGGACTAAAGGTCATAACACACAATGTTTCAACAACACACTTCGCAAAGTGCACAGTCAAGCAAGCGCGAACAATGACGCAATTTGAGCTTTCACCATTCGTAATGGCAGAACTAGTCAAATTTGATGGCTCAATGCACCCACAAATCCATGAAGCACTTGTGAAATATAAGCTTAGAGATTCTGTAATAATGCTCAGACCTAACGCAATTCCCAAAGTTAATTTTCATAACTGGCTGACAGCACGTGATTACAACAGGATGGGTTGTTCACTAGAGCTAGAGGATCATGTCAAAATTCCATATTACATACGAGGCGTCCCTGACAAGTTATACGGGAAATTGTATGATATCATTTTACAATATAGTCCAACCAGTTGTTACGGAAGACTATCAAGTGCTTGTGCTGGGAAGGTAGCATACACCTTGCGAACGGACCCTTGTTCGCTACCACGAACAATAGCAATAATCAATGCATTAATCACAGAGGAGTATGCTAAGAGAGATCATTACCGTAATATGATAGCAAATCCTTCGTCTTCGCATGCATTCTCACTAAATGGATTAGTCTCCATGATTGCTTCAAGATACATTAAGGACCACACAAAAGAGAATATAGACAAACTTATTAAGGTGCGGGATCAACTACTCGAATTTCAAGGCATGGGTATGCAATTTCAAGATCCATCAGAACTAATGGACATTGGCGCCTTGAACACAGTTATACATCAAGGAATGGATGCAACAGCTGCATGCATTGGTCTCCAAGGACGATGGAATGCATCACTCATACAACGTGATCTCATGATTGCAGGAGGAGTATTCATTGGAGGAATATTAATGATGTGGAGCTTGTTCACTAAGTGGGGAACGACCAATGTATCACACCAAGGAAAGAACAAGCGTAGCCGACAAAAATTGAAGTTCAAGCAAGCTAGAGACACTAAATACGCATATGATGTGACAGGATCGGAAGAAACTCTAGGTGAAAACTTTGGAACAGCTTATACAAAGAAGGGCAAAGGAAAAGGAACCAAAGTTGGTCTTGGGGTGAAGCAGCATAAATTTCACATGATGTATGGTTTTGATCCTCAAGAGTACAACCTAATTCGTTTTGTCGACCCTCTTACAGGTGCAACATTAGATGAACAAATCCATGCTGATATTCGCTTAGTGCAAGAACACTTTGACATAATCCGAGAAGAGGCAGTCGCAAACGACACAATTGAGCGACAACACATATACGGAAATCCTGGTCTCCAAGCATTCTTCATACAGAATGGATCAGCTAATGCATTAAGAGTTGATTTAACACCACATTCACCTTTGCGTGTTGTGACGAACAACAACATAGCAGGATTTCCAGAATATGAGGGCACATTACGACAAACTGGCACAGCTCTCACTGTACCTGTGAATCAAGTACCAGCAGCAAATGAGACAGGAGTTGCCCACGAATCCAAATCTATGATGGCTGGATTGGGCGATTACACACCTATTTCTCAGCAGCTCTGTCTCGTACAAAATGATTCTGAAGGAGTCAAACGTAATGTGTATGCAATTGGATATGGATCATATTTAATATCACCGGCGCATCTTTTCAAGTATAACAATGATGAAATCACAATTAAATCCTCAAGAGGGCTGTATAAAATTAGAAATTCAGTCGATGTCAAATTGCACCCGATTGCACAGAGGGACATGGTCATAATTCAACTTCCAAAAGACTTCCCACCGTTCCCAATGCGACTTAAGTTCTCAACTCCGTCAAGAGATGTGCGTGTGTGCTTAGTTGGAATCAACTTTCAACAGAATCATACCACGTGCATAATATCCGAAAGCAGTGTGACAGCACCCAAAGGAAATGGTGATTTCTGGAAACATTGGATTTCAACTGTTGATGGGCAATGTGGGCTACCGTTAGTTGACGTTAAGAATAAACACATTGTCGGAATTCACAGCCTGGCCTCAACAAGTGGAAATACGAACTTTTTCGTTGCAATGCCTGAGAACTTCAATGAATACATATCTAATCTCGTGCAAACGAATAAGTGGGAAAAGGGATGGCATTACAACCCAAATCTTATTTCATGGTGTGGTCTAAACCTAGTTGATTCAGCACCTAAAGGATTGTTTAAAACATCAAAACTTATTGAAGATTTGGATATGAGCGTTGAAGAACAATGCAAGGTGACAGAGACATGGTTGACGGAACACATTCAGGATAATCTACAGGTCGTTGCAAAGTGTCCAGGCCAACTTGTAACAAAGCATGTCGTTAAAGGCCCATGCCCACACTTTCAACTGTATTTATCCACACATGATGAGGCAAGATTGTACTTTTCACCTTTGCTTGGAAAGTATGATAAGAGTAGGTTGAACAGGGCAGCATTTATTAAAGATCTTTCAAAGTACGCAAAGCCGATTTATATTGGAGAGATCAATTATGAAATCTTTGATAAGGCAGTTGATCGAGTTATAAGCATCCTCAGAAGTGTAGGAATGCTACAGTGTACATACGTGACGGACGAAGAAGAAATTTTCAATTCGTTAAATATGAACGCAGCCGTAGGTGCACTCTACACAGGAAAGAAGAAAGACTATTCCAAAGATTTCTCGAACGATGACAAAGCCGAAATCATCATGCGTTCATGTGAGCGCATCTACAATGGACAATTGGGTGTATGGAACGGTTCACTCAAAGCTGAAATACGACCAATAGAGAAAACCATACTAAACAAGACACGCACTTTCACAGCAGCGCCATTAGAAACTCTACTTGGTGGGAAAGTATGTGTGGACGATTTCAACAATCAATTTTATTCACATCATCTTGAAGGCCCATGGACCGTAGGAATCACAAAGTTTTATGGAGGATGGAATCGACTTTTGGAGAAATTGCCAGAAGGATGGATTTATTGCGATGCAGATGGATCCCAGTTTGACAGCTCACTAACTCCATATCTTATTAATGCTGTATTGCACATTCGCTTACATTTCATGGAAGAATGGGAGTTGGGAGCTCAGATGTTGCGAAATTTATACACAGAGATTGTTTATACGCCAATCGCAACGCCTGATGGGTCTGTCATCAAGAAATTCAAAGGAAATAATAGTGGGCAACCATCTACAGTCGTTGACAACACGCTTATGGTTATTATAGCATTTAATTACGCAATGTTATCAAGTGGCATTCCTGAAGACAAAATTGACGACTGCTGTAGAATGTTTGCAAACGGTGACGACTTACTCTTGGCAGTGCATCCGGATTACGAATATATACTGGACGGATTTCAAAATCATTTTGGAAACCTTGGCCTTAACTTTGAGTTCACATCGAGGACAAAGGACAAATCAGAGTTATGGTTTATGTCAACACAAGGAGTCAAGTGTGAAGGTATCTACATACCAAAACTCGAAAGGGAAAGAATAGTCGCAATCCTTGAATGGGACCGATCGAACTTGCCTGAGCATCGTCTTGAAGCTATCTGTGCAGCCATGGTTGAAGCATGGGGTTACCCAGATTTAGTTCATGAAATTCGTAAGTTTTACGCGTGGCTTCTTGAAATGCAACCCTTCGCTAACCTGGCAAAGGAAGGCATGGCGCCATACATAGCAGAAACAGCACTCCGCAACCTCTACCTTGGAACAGGCATCAAAGAAGAAGAAATTGAAAAATATTTTAGACAGTTTGTTAAGGATCTTCCTGGATACGTAGAAGATTACAATGAAGAAGTTATTCATCAATCTGGTCAAGTTGACGCAGGGAGACAGGGCGGTAGCGGCGCTCAAGGAGGCACACCACCAGCAGGA---------------------------------------------------AGTGGAGGCAC----TGGATCTGGCACTCAAGGCAATGGGGGTCAGACGGGA---------TCCCAAG---GAAGTGGTGGTCAACAAGGGTCCGGTGGGGGCACTGGTCAAGGAGCAGCTGGAAACAACGGCGG------------AGGTCAGACAGGAGGCTCTAGTGGG------------ACAGCTGGTCAGAGAGATAAGGACGTTGACGCAGGCTCAGCTGGAAAGATATCCGTGCCAAAGCTTAAAGCCATGTCAAAGAAAATGCGCTTGCCAAAGGCAAAAGGAAAAGACGTCTTGCATTTGGACTTTTTATTGACATATAAGCCACAACAGCAGGACATATCGAACACAAGAGCAACTAAGGAAGAGTTCGATAGATGGTACGACGCCATAAAGAAGGAGTACGAGATCGATGATACACAAATGACAGTCGTCATGAGTGGTCTGATGGTCTGGTGCATCGAAAATGGTTGCTCACCAAACATAAACGGAAATTGGACGATGATGGATGGGGATGAACAAAGAGTTTTCCCACTAAAACCAGTTATTGAAAACGCATCTCCAACTTTTCGACAAGTTATGCATCATTTTAGCGATGCAGCTGAAGCGTATATAGAATACAGAAATTCTACTGAGCGATACATGCCAAGATATGGACTTCAGCGAAATCTCACCGACTATAGCTTAGCGCGGTATGCTTTTGATTTCTATGAAATGACTTCACGCACACCAGCTAGAGCTAAGGAAGCCCACATGCAG---ATGAAAGCCGCAGCAGTTCGTGGTTCAAACACACGACTGTTCGGCTTGGACGGAAATGTCGGCGAGACTCAGGAGAATACAGAGAGACACACAGCTGGCGACGTTAGTCGCAATATGCACTCTCTGTTGGGAGTGCAGCAACACCACTAGTCTCCTGGAAACCCTGTTTGCAGTACTTATAATATATACTA------ATATATAGTACGTTGGTGAGGCTTTGCCTC-----------------------GGTTTTACTATCTTATTATGTATGTATTTACAGCGTGAACCAGTCTGCAGCATGCAGGGTTGGACCCAGCGTGTTCTGGTGTAGCGTGTACTAGCGTCGAGCCATGAGACGGACTGCACTGGGTGTGGCTGTGCCACTTGTGTTGCGAGTTTCCTGGTAAGAGAC----------------

>KR611114

-----------------------------------------------------------------------------------------------------------------------------------------------------------------------------------ATGGCGGGCTCTTGGACTCACGTGACATACAAGTGGCAACCAGATGTCAACAACGCACGTGATGTCAAAAGAGTGATGGAGATGTTTGCAGCAAAACATCAACGTTACACTGAGGAGCAAAGGCTTGCTCACAACAGCAAGCTATTAAGGAAGGCTTGTGTCACTAGTGCTGAGTTTGTTGAACCAGCACAGAAACCAAAATGTCACCAGACATGGGTTGAAAAGTGCGACCACAACCCCACAGAGCACTTCGTTTATCAACGCTTC---ACACCTGAAAAGAAAGTGCTTAACACCAAACCTGAGACAACTTCTGTCACGAAGTTAATCAGGGATGTCCTTGAAATTTCGAAGGGTAGTGGAATAAAAATTGAGTTAATTGACAAGCGCATCAAACGTAAGACTCAATTATCCATAAGGCAACACAATGGCAAAGATTTCTTGCATTGCAAAACCAGGCATGAAAATGGCTTGTTCAAACGCAAGGACATTGACATTAGTGTCAAGTGGTTACCCACCATTGAAGCCATTGCAAAATGCTACAGCACGGTGAATGCAGAAGAACTACAAAGTCTCAATAGAGGCAGTAGTGGTCTTACATTCATGCAAAACGATGAATTGTTCATCGTGCGTGGAAGGATGCATGGTGAGATTGTTAATAGTTTGCACGAAAGTAAGCATGTTATGGAAATTGAACACTATGCTGATCCACAAGCAAACAGTTTCTGGAAAGGTTATACAGATGCATATGTCGGGAACAGAAACATATCCACCACTCACACAGAGCACACACCAACTATTAATTTAGAGGAGTGTGGCAAGAGAATGGCACTCTTAGAAATCTTATTCCATTCAACTTTTAAAATTACATGCAAAACGTGCAATATTGATGATCTGGAATTATCAGATGATGAATTTGGGGCCAAGTTATACAGTAATCTGCAGCGTATTGAAGAAAGGCAACGCGAATATCTTGCTAAAGATCAAAAACTTTTACGCATGATTCACTTTGTGAAGGACCGGTGTAATCGAAAATTTTCACATTTGCCTTTACTATGGCAAGTGGCAGAAACAGTAGGACATTACACTGATAATCAATCGAAGCAGATAATTGATATCAGTGAGGCGCTCATCAAAGTTAATACCTTAACTCCTGATGATGCAGTGAAGGCCAGTGTAGCATTATTGGAAGTAGCACGATGGTATAAAAATCGGAAGGAATCACTTAAAACAGACACATTGGACTCATTCCGAAACAAAATTTCACCAAAGAGCACGATCAACGCAGCATTGATGTGTGATAACCAGTTAGATAAGAATGCTAATTTCGTATGGGGAAACAGAGAATACCATGCAAAGCGATTCTTCGCTAATTATTTTGAAGCTGTAGACCCAACTGATGCATATGAAAAACACGTCACACGCTTCAACCCCAATGGACAGCGGAAATTATCAATTGGCAAATTAGTAATTCCACTAGATTTCCAGAAGATTAGAGACTCGTTCGTTGGCCTATCGATAAATAAACAACCACTGAGCAAAGCTTGTGTAAGCAAAATTGATGGAGGCTACGTATATCCATGTTGCTGCGTTACAACGGAATTTGGAAAACCAGCATATTCTGAGATAATACCTCCAACAAAAGGACATATCACGATTGGAAATTCAGTGGACCCAAAAATAGTGGATTTACCGAACACAACACCACCAAGTATGTACATTGCAAAAGATGGATATTGTTATATTAATATATTCTTAGCAGCAATGATAAATGTCAATGAGGAATCCGCAAAAGATTACACGAAGTTCCTTAGAGATGAGTTGGTGGAACGGCTTGGCAAATGGCCAAAATTGAAAGATGTAGCAACAGCATGTTATGCTTTATCAGTAATGTTCCCAGAAATCAAGAATGCCGAATTACCACCAATATTAGTTGATCATGAGAGTAAGTCAATGCATGTCATTGATTCATATGGATCACTCAGTGTTGGATTCCACATTCTAAAGGCAAGTACTGTTGGACAACTGATAAAATTTCAGTATGAATCATTGGAAAGTGAGATGCGCGAATACATAGTGGGAGGCACTTTGACACAACAAACTTTCAGCACACTTCTTAAGACTCTCACAAAGAACATGTTTAAGCCAGATAAAATAAAGCAGATAATAGAGGAAGAGCCTTTCCTACTAATGATGGCAATTGCATCCCCAACTGTACTTATCTCGCTGTACAACAACTGCTACATCGAGCAAGCAATGACATATTGGATTGTCAAGAACCAAGGAATCGCAGCAATTTTTGCACAGTTGGAGGCATTAGCAAAGAAAACTTCTCAAGCAGAACTACTAGTTCTTCAAATGCAAATACTTGAAAAAGCTTCTTACCAACTGAGACTTGCAGTCACGGGACTTAATCATGTTGATCCAGCAAAACGACTTTTATGGTCTCACCTAGAAGCCATGACAACACGATCGGAGATGAACAAGGAACTCATAGCGGAAGGTTATGCACTGTATGACGAGCGCTTATATACTTTAATGGAAAAAAGTTACGTAGATCAATTAAACCAATCATGGGCAGAATTATCATACTGTGGAAAATTTTCAGCAATATGGCGTGTGTTCAGAGTCAGGAAATACTACAAACCGTCTTTAACCGTGAGAAAAAGCGTAGATTTAGGCGCTGTTTACAATATATCAGCTACGCATCTAATATCAGATTTAGTGCAGAGAAGTCGAGATCAAGTCAGCTCTACTTTAACCAAACTCCGCAACGGTTTCTATGATAAAATGGAGAGAGCGAGAGTTAGTGCAGTAAGGACAATATATTGGTTCATACCTGACATATTTAGACTAATCCATATTTTCATAGTTTTAAGTTTTTTAACAACTATAGCTAATACAATAGTCGCAACTATGAATGATTATAAAAAGTTGAAAAAGCAACAAAGAGAAGACGAATATGAAGCTGAGATTAATGAGGTACGAAAAATACACGCCAACCTGATGAAGGAGCATAATGATAATCTGACATGTGATCAATTTATTGAACACATACGCCAGACACATCCACGCCTCATTGAGGCAACATTGGATTTAACACATACAGGTGTCATCCATGAGGGTAAATCCAATTTGGAAACAAACCTTGAACAGGCGATGGCAGTGGGAACTTTACTCACTATGATACTCGATCCACAAAAGAGTGATGCAGTTTATAAGGTTCTCAATAAGATGCGAACAGTGATTAGCACAATCGAACAGAATGTGCCATTTCCATCAGTCAACTTCACAAGCATCTTGACACCTCCTGTAACACAGCAAAGTGTTGATGTTGACGAACCATTAACACTGAGTACCGATAAGAATTTGACTATAGATTTTGACACAAATCAAGATTTGCCAGCGGATACATTCAGCAATGACGTTACATTCGAGAACTGGTGGGCTAATCAGATAAACAACAACAGAACAGTGCCACACTATCGACTTGGGGGAAAGTTTGTAGAATTCACAAGAGAAAATGCAGCAATGGTTAGCATTGAGCTTGCTCACTCGAACATCGAAAAAGAATTTCTACTCAGAGGAGCTGTTGGATCAGGAAAATCCACAGGTTTGCCATATCATCTCAGTATGCGTGGAAAAGTGCTATTGATAGAACCTACTCGACCATTAGCTGAGAACGTTTGCAGGCAACTGCAAGGTCCTCCATTTAATGTGAGCCCCACTTTACAAATGAGAGGTTTGAGCACATTTGGCTGCACTCCTATCACGATAATGACGTCTGGTTTCGCATTGCACATGTATGCAAATAACCCCGATAAGATCTCTGAATACGACTTCATCATCTTTGATGAATGTCACATTATGGAAGCACCTGCAATGGCATTCTATTGTTTGCTTAAGGAGTATGAATACCGAGGCAAGATAATAAAAGTTTCAGCTACACCACCAGGACGAGAATGCGAGTTTTCAACCCAACATCCAGTAGATATACATGTATGTGAAAGCTTGACACAACAGCAATTCGTCATGGAACTAGGAACAGGATCAACTGCTGATGCAACCAAATATGGCAATAACATATTAGTGTACGTTGCAAGTTATAATGATGTAGATTCTTTATCCCATGCTCTAACTGAACTTAAATATTCAGTAATTAAAGTCGATGGAAGAACAATGAAGCAGAACACCACAGGAATCGTAACAAATGGAACATCCAGTAAGAAATGCTTCGTTGTGGCCACAAACATTATTGAAAACGGTGTAACGCTAGATGTCGATGTCGTCGTCGACTTTGGACTTAAAGTAACAGCTGAATTAGATGTTGATAACAGGGCGATAATGTATAAACGTGTGAGCATATCTTATGGCGAGCGCATTCAGAGACTCGGAAGAGTTGGAAGGAATAAGCCTGGAACAGTTATCCGCATCGGGAAAACAATGAAAGGTTTACAAGAAATTCCAGCGATGATTGCCACTGAAGCAGCTTTCATGTGTTTTGCATATGGACTGAAGGTTATAACACATAATGTATCAACAACACATCTGGCAAAATGCACCGTCAAACAAGCTAGAACCATGATGCAATTTGAGCTATCACCATTTGTAATGGCTGATTTAGTTAAATTTGACGGTTCTATGCACCCACAGATTCATGAAGCATTAACCAAGTATAAATTGAGAGATTCTGTGATCATGTTAAGACCAAATGCAATACCAAAGGTAAATCTTCACAACTGGCTGACGGCTCGTGATTACAACAGGATAGGCTGCTCATTAGACCTTGAAGACCACGTTAAAATACCATATTATATACGGGGAGTTCCTGACAAGCTGTATGGGAAGTTATATGATATTATCCTTCAATACAGCCCTACAAGTTGCTATGGAAGACTATCAAGCGCTTGTGCAGGTAAGGTAGCATATACACTGCGCACTGATCCTTGTTCGTTACCAAGAACAATAGCTATAATCAACGCATTAATCACTGAAGAGTATGCAAAGAGGGATCATTACAGAAACATGATAGCGAACCCTTCGTCATCGCACGCCTTTTCACTTAATGGGCTGGTATCCATGATCGCTTCTCGATACATGAAAGATCACACGAAGGAAAACATTGATAAACTTGTAAGAGTGCGCGACCAGCTACTTGAATTCCAAGGCACAGGTATGCAATTTCAAGATCCTTCAGAATTGATGGACATTGGTGCTTTAAACACAGTTATTCACCAAGGAATGGACGCTACGGCTGCTTGTATTGGACTGCAAGGGCGCTGGAATGCTTCGCTCATTCAACGCGATTTGATGATATCAGCAGGGGTCTTCACAGGAGGAATTCTCATGATGTGGTATCTTTTCACAAAATGGAGCAAGACAGAAGTGTCACACCAAGGAAAGAACAAGCGCAGTCGGCAAAAGCTACGATTCAAAGAGGCTCGTGATAATAAATATGCCTACGATGTAGTAGGATCAGAGGAAGTTCTTGGTGAGAATTTCGGAACTGCATACACTAAGAAAGGAAAAGGAAAGGGGACAAAAGTTGGCCTTGGAGTCAAACAGCACAAATTTCACATGATGTACGGGTTTGATCCACAAGAATACAACTTAATCCGCTTCGTGGATCCTTTAACAGGTGCGACACTAGACGAGCAAATCCATGCAGATATACAGTTAGTGCAAGAGCACTTCAGCGTCATCAGAGATGAAGCAGTAGCAAATGACACAATTGAAAGACAGCACATTTACGCTAATCCTGGATTACAAGCTTTCTTCATACAAAATGGATCGGCAAATGCACTGAGAGTTGATTTGACGCCGCATTCACCCTTGCGTGTCGTGACCAATAACAATATAGCAGGCTTCCCAGAGTATGAAGGTACACTTCGGCAAACAGGAACAGCTCTACAAATACCCGTGAATCAAGTTCCAGCTGCGAACGAAGCGGGGGTGGCACATGAATCGAAATCGATGATGGCAGGGCTAGGTGACTACACTCCAATATCACAGCAATTGTGCCTAGTTCAAAATGACTCAGATGGAATCAAAAGGAATGTATATTCAATTGGATATGGATCATATCTCATTGCGCCAGCACATTTATTTAAGTATAATAATGGCGAAATAACGATCAAATCATCACGAGGCCTATACAAAATCAGAAATTCAGTCGAAATCAAGTTGCATCCCATTGCGCATAGAGACATGGTTATAATTCAACTTCCAAAAGATTTTCCACCATTCCCAATGCGTCTTAAGTTTTCAAAACCATCTAGAGAATCAAGGGTGTGCTTAGTCGGAGTGAATTTTCAACAGAACTATAGCACATGTATCGTATCGGAGAGTAGCGTCACAGCACCAAAAGGAAACGGAGATTTCTGGAAACACTGGATATCCACAGTAGACGGACAATGCGGTCTCCCATTAGTAGATGTCAAGAGTAAGCACATAGTTGGAATACACAGTCTTGCATCAACTAGTGGAAATACTAACTTCTTTGTCGCCATGCCTGAAGACTTCAATGACTACATCAATAATCTTGTGCAAACCAACAAGTGGGAAAAAGGATGGCATTACAACCCAAATCTCATTTCATGGTGTGGTCTCAACCTAGTTGACTCAGCTCCAAAGGGTCTTTTCAAAACTTCAAAATTAGTAGAAGACCTTGATATGAGCGTTGAAGAACAATGTGAGGTAACAGAGACATGGCTCACGGAGTGTATTCAGGATAATTTACAGGTTGTTGCAAAATGTCCAGGCCAACTTGTTACCAAGCACGTTGTCAAAGGCCCATGCCCACACTTTCAGCTATACCTGTCAACACATGATGAAGCTAAAGCATACTTTGCACCATTACTCGGAAAATACGACAAGAGCAGATTAAACAGAGCAGCTTTTATCAAAGACATTTCAAAATATGCAAAACCAATCTACATTGGAGAAATCAATTACGATGTCTTTGAAAAGGCTATACAACGTGTAATTAAAATTCTTAGAGACGTGGGAATGCAGCAATGCACGTATGTAACGGACGAGGATGAAATATTCCAGTCACTTAACCTCAACGCTGCAGTTGGCGCCTTATACACAGGAAAGAAGAAAGATTATTTCAAGGATTTTTCAAATGAGGACAAATCAGAAATTATCATGAGATCCTGTGAGCGTTTATACAACGGACACCTTGGCGTGTGGAATGGTTCACTCAAAGCTGAAATAAGGCCTATAGAGAAAACAATGTTAAATAAGACTCGGACTTTTACAGCAGCACCATTAGAAACTTTACTTGGTGGCAAGGTTTGTGTCGATGATTTCAACAACCAATTCTACTCGCACCACTTAGAAGGTCCTTGGACAGTTGGAATAACAAAGTTTTATGGTGGGTGGAACCGTTTGTTGGAAAAATTGCCAGATGGTTGGATATACTGCGACGCCGATGGATCACAGTTCGACAGCTCTTTGACACCATATCTCATCAACGCCGTATTACACATTCGATTACAATTCATGGAAGAATGGAACTTAGGAGAACAAATGTTGCGAAACTTGTACACTGAAATCGTGTACACACCAATTGCAACACCAGATGGATCTGTAATCAAGAAATTTAAAGGAAATAACAGCGGGCAGCCGTCAACAGTTGTAGACAACACACTCATGGTGATATTAGCATTTAATTATGCAATGTTATCAAGTGGTGTTAAAGAGGAAGAAATAGACAATTGCTGCCGAATGTTCGCCAATGGTGATGATTTGCTACTTGCAGTGCATCCAGATTTCGAACACATATTGGACGGATTTCAAAATCACTTTGGAAACCTAGGTCTCAACTTTGAGTTTACATCACGAACAAGAGACAAGTCAGAGTTATGGTTCATGTCCACACGAGGTATCAAATGCGAAGGCATCTATATACCAAAGCTTGAGAAAGAAAGAATAGTTGCCATACTCGAGTGGGATCGGTCAAACTTACCTGAGCACCGTCTCGAAGCCATTTGTGCAGCCATGGTAGAGGCATGGGGATACCCAGACCTTGTCCAAGAGATACGGAAATTTTATGCGTGGCTTCTGGAAATGGAACCATTTGCAAATTTAGCTAAAGAGGGCCTAGCACCATATATCGCTGAAACTGCACTCAGAAATCTGTATTTGGGCACAGGGATCAAGGAAGAAGAGATTGAGAAATATTTCAAACAGTTTGCCAAAGATCTCTCTGGTTATATAGAAGATTACAACGAAGATGTTTTTTATCAATCCGGAACTGTGGATGCAGGAGCCCAAGGAGGGGGAAGTGGATCTGGAACAACGCCACCAGCAACA---------------------------------------------------GGTAGTGGAAC----AGGAACAAGAACACCATCTACTGGAACTCCAGCACAAGGCAACACACCTCCAG---CATCAGGTGGATCATCAGGAAACAATGGAGGC---AGCCAATCAGGTTC---AAACGGCACTGG------------AGGCCA---AGCAGGCTCAAGCGGA------------GCAGGGGGTCAAAGAGACAAAGACGTTGACGCTGGCTCAACAGGGAAGATATCAGTGCCAAAGCTTAAGGCAATGTCGAAGAAAATGCGCCTGCCAAAGGCGAAAGGAAAAGACGTTTTACATTTAGATTTCCTTTTAACATATAAACCCCAACAGCAAGATATATCAAACACTAGAGCAACTAAGGAAGAGTTTGATAGATGGTACGATGCCATAAAGAAGGAGTACGAAATTGATGACACACAAATGACAGTTGTCATGAGTGGTCTCATGGTATGGTGCATCGAAAATGGTTGCTCACCAAACATAAACGGAAATTGGACGATGATGGACGGAGACGAACAAAGGGTTTTTCCATTAAAGCCAGTCATTGAGAACGCATCTCCAACTTTCCGACAGATAATGCATCATTTTAGTGATGCAGCTGAAGCGTATATAGAGTACCGAAACTCTACAGAGCGATACATGCCAAGATACGGTCTTCAGCGAAATCTCACCGACTATAGCTTAGCGCGGTATGCTTTCGATTTCTATGAAATGACTTCGCGCACACCAGCTAGAGCTAAGGAAGCCCACATGCAG---ATGAAAGCCGCAGCAGTTCGTGGTTCAAACACACGTCTGTTCGGTCTGGACGGAAATGTCGGCGAGACTCAGGAGAATACAGAGAGACACACAGCTGGCGACGTTAGTCGCAACATGCACTCTCTGTTGGGAGTGCAGCAGCACCAC--------------------------------------------------------------------------------------------------------------------------------------------------------------------------------------------------------------------------------------------------------------------------------------------

>KR611112

-----------------------------------------------------------------------------------------------------------------------------------------------------------------------------------ATGGCGGGCTCTTGGACTCACGTGACATACAAGTGGCAACCAGATGTCAACAACGCACGTGATGTGAAAAGAGTGATGGAGATGTTTGCAGCAAAACATCAACATTACACTGAGGAGCAAAGGCTTGCTCACAACAGCAAGCTATTAAGGAAGGCTTGTGTCACTAGTGCTGAGTTTATTGAACCAGCACAGAAACCAAAATGTCACCAGACATGGGTTGAAAAGTGCGACCACAACCCCACAGAGCACTTTGTTTATCAACGCTTT---ACACCTGAGAAGAAAGTGCTTAGCACCAAACCTGAGACAACCTCTGTTACGAAGTTAATCAGGGATGTCCTTGAAATTTCGAAGGGTAGTGGGATAAAAATTGAGTTAATTGGCAAGCGTATCAAACGTAAGACTCAATTATCCATAAGGCAACACAATGGCAAAGATTTCTTGCACTGCAAAACCAGGCATGAAAATGGCCTGTTTAAACGCAAGGACATTGACATTAGTGTCAAGTGGTTGCCCACCATTGAAGCCATTGCAAAATGCTACAGCACGGTGAATGCAGAAGAACTGCAAAGTCTCAATAGAGGCAGTAGTGGTCTTACATTCATGCAAAACGATGAATTGTTCATCGTGCGTGGAAGGATGCATGGTGAGATTGTTAATAGTTTACACGAAAATAAGCATGTTATGGAAATTGAACACTATGCTGATCCACAAGCAAACAGTTTCTGGAAAGGTTATACAGATGCATATGTCGGGAACAGAAACATATCTACCACTCACACAGAGCACACACCAACTATTAATTTAGAGGAGTGTGGCAAGAGAATGGCACTCTTAGAAATCTTATTCCATTCAACTTTCAAAATAACATGCAAAACGTGCAATATTGATGACCTGGAATTATCAGATGATGAATTTGGGGCCAAGTTATACAGTAATCTGCAACGCATTGAAGAAAGGCAACGCGAATATCTTGCTAAAGATCAAAAACTTTTACGCATGATTCACTTTGTGAAGGACCGGTGTAATCGAAAATTTTCACATTTGCCTTTGCTATGGCAAGTGGCAGAAACAGTAGGACATTACACTGATAACCAATCGAAGCAGATAATTGATATCAGTGAGGCGCTCATCAAAGTTAATACCTTAACTCCTGATGATGCAGTGAAGGCCAGTGTAGCATTATTGGAAGTAGCACGATGGTATAAAAATCGGAAGGAATCACTTAAAACAGACACATTGGACTCATTCCGAAACAAAATTTCACCAAAGAGCACGATCAACGCAGCATTGATGTGTGACAATCAGTTAGATAAGAATGCTAATTTCGTATGGGGAAACAGAGAATATCATGCAAAGCGATTCTTCGCTAATTATTTTGAAGCTGTAGACCCAACTGATGCATATGAAAAACACGTCACACGCTTCAACCCCAATGGACAGCGGAAATTATCAATTGGCAAATTAGTAATTCCACTAGATTTCCAGAAGATTAGAGACTCGTTTGTTGGCTTATCGATAAATAAACAACCACTGAGCAAAGCTTGTGTAAGCAAAATTGATGGAGGCTACGTATATCCATGTTGCTGCGTTACAACGGAATTCGGAAAACCAGCATATTCTGAGATAATACCTCCAACAAAAGGACATATCACGATTGGAAATTCAGTGGACCCAAAAATAGTGGATTTACCAAACACAACACCACCAAGTATGTACATTGCAAAAGATGGATATTGTTATATTAATATATTCTTAGCAGCAATGATAAACGTCAATGAGGAATCCGCAAAAGACTACACGAAGTTCCTTAGAGACGAGTTGGTGGAACGGCTCGGCAAGTGGCCTAAATTGAAAGATGTAGCAACAGCATGTTACGCTTTATCAGTAATGTTCCCAGAAATCAAGAATGCCGAATTACCACCAATATTGGTTGATCATGAGAGCAAGTCAATGCATGTCATTGATTCATATGGATCACTCAGTGTTGGATTCCACATTCTAAAGGCAAGTACCGTGGGACAACTGATAAAATTCCAGTACGAATCATTAGAAAGCGAGATGCGTGAATACATAGTGGGAGGTACTTTGACACAACAAACTTTTAACACACTTCTCAAGACTCTCACAAAGAACATGTTCAAGCCAAATAAAATAAAACAGATAATAGAGGAAGAACCTTTCCTACTAATGATGGCGATTGCATCCCCTACCGTGCTTATCTCACTGTATAACAATTGCTACATCGAGCAGGCGATGACATACTGGATTGTCAAGAACCAAGGAGTCGCGGCAATTTTTGCACAGTTGGAGGCTTTGGCAAAGAAAACCTCCCAAGCAGAACTATTAGTTCTGCAAATGCAAATACTTGAAAAAGCTTCTTACCAGCTAAGACTTGCAGTCACCGGACTTAATCATGTCGATCCAGCGAAACGACTTCTGTGGTCTCACCTAGAAGCCATGACAACACGATCGGAAATGAATAAGGAACTCATAGCGGAAGGTTATGCACTATATGATGAGCGTCTATATACCCTAATGGAAAAAAGTTATGTAGATCAATTAAACCAGTCATGGGCAGAATTATCATACTGTGGAAAATTTTCAGCAATATGGCGTGTGTTCAGAGTCAAGAAATACTACAAGCCATCTTTAACCGTGAGAAAAAGCGTAGATTTAGGCGCTGTTTACAATATATCAGCTACGCATCTAATATCAGATTTAGTGCAGAGAAGTCGAGATCGAGTCAGCTCTACTTTAACCAAACTCCGCAACGGTTTCTATGACAAAATGGAAAAAGCGAGGGTTAGCGCAGTGAGGACTGTATATTGGTTCATACCCGACATATTTAGACTAGTCCACATCTTTATAATTCTAAGTTTTTTAACTACTATAGCCAATACAATAGTTACAACCATGAATGACTACAAAAAGTTAAAGAAGCAACAAAGAGAGGACGAGTATGAGGCCGAAATTAATGAGGTGCGGAAAATACATGCCAATTTGATGAAGGAGCACAATGATAATCTGACATGCGATCAGTTTATTGAATACATACGCCAAACACACCCACGCCTCATTGAAGCAACTTTGGATTTGACACATACAGGCGTTATTCACGAGGGTAAATCCAATCTGGAAACCAACCTTGAACAGGCGATGGCAGTGGGAACTTTACTCACTATGATACTCGATCCACAGAAGAGTGATGCAGTTTATAAGGTTCTCAACAAAATGCGAACAGTGATTAGCACAATTGAACAGAATGTGCCATTCCCTTCAATTAACTTCACAAACATCTTGACACCTCCTGTAACACAGCAGAGTGTTGATGTTGATGAACCATTGACATTGAGTACTGACAAGAACCTGACTATAGATTTTGATACAAATCAAGATTTGCCAGCAGATACATTCAGTAATGATGTTACATTCGAGGATTGGTGGGCTAATCAGATAAACAACAACAGAACAGTGCCACACTATCGACTTGGGGGAAAGTTTGTAGAATTCACAAGAGAAAATGCAGCAATGGTTAGCATTGAACTCGCTCACTCGAACATTGAAAAAGAATTTCTATTAAGAGGAGCTGTTGGATCAGGAAAATCCACAGGTTTGCCATACCATCTCAGTATGCGTGGAAAAGTGCTATTAATAGAACCCACACGACCACTAGCTGAGAATGTTTGCAGACAACTGCAAGGTCCTCCATTCAATGTGAGCCCCACACTACAAATGCGAGGTTTAAGCACATTTGGCTGCACTCCTATCACGATAATGACGTCTGGTTTCGCATTACATATGTACGCAAACAACCCTGACAAGATCTCTGAATATGACTTCATCATCTTTGATGAATGTCATATCATGGAAGCACCAGCAATGGCATTTTATTGTTTGCTTAAGGAGTATGAATATCGAGGCAAGATAATTAAAGTTTCAGCAACACCACCAGGAAGAGAATGTGAATTTTCAACCCAACATCCAGTGGATATACATGTATGCGAGAGCTTGACACAACAGCAATTCGTCATGGAACTTGGAACAGGGTCAACTGCTGACGCAACCAAATATGGCAACAACATATTAGTGTACGTTGCAAGTTACAATGACGTAGATTCTTTATCCCACGCTCTAACTGAACTCAAATATTCAGTAATCAAAGTTGACGGAAGAACAATGAAGCAGAACACCACAGGAATTGTAACTAATGGAACATCCAATAAGAAATGCTTCGTTGTAGCCACGAACATCATTGAGAATGGTGTGACATTAGATGTTGATGTCGTAGTCGACTTTGGACTTAAAGTAACAGCTGAACTGGATGTTGATAACAGAGCAATAATGTATAAACGTGTGAGCATATCTTATGGCGAGCGTATTCAAAGACTCGGAAGAGTTGGAAGGAATAAGCCCGGAACAGTTATCCGCATCGGGAAAACAATGAAAGGTTTGCAAGAAATTCCAGCGATGATCGCAACTGAAGCGGCTTTCATGTGTTTTGCATACGGACTGAAAGTTATAACACACAATGTATCAACAACACACTTGGCAAAATGCACCGTTAAACAAGCCAGAACTATGATGCAATTTGAACTATCACCATTTGTAATGGCCGATTTAGTTAAATTTGATGGTTCAATGCACCCACAAATTCATGAAGCATTAACTAAATACAAATTTAGAGATTCTGTGATTATGCTAAGACCAAACGCAATACCAAAGGTAAATTTTCACAACTGGCTGACGGCTCGTGATTACAACAGGATAGGCTGCTCATTAGAACTCGAAGATCATGTTAAAATACCATATTATATACGAGGAGTTCCTGACAAGCTGTATGGGAAGTTGTATGATATCATCCTTCAATACAGCCCTACAAGTTGCTATGGAAGACTATCAAGTGCTTGTGCAGGTAAAGTAGCATATACACTGCGCACTGATCCTTGTTCGTTACCAAGAACAATAGCTATAATCAACGCATTAATTACTGAAGAGTATGCAAAGAGGGATCATTACAGAAACATGATAGCGAACCCTTCATCATCGCACGCCTTTTCACTTAATGGGCTAGTATCCATGATCGCTTCTCGGTACATGAAAGACCACACGAAGGAAAACATTGATAAACTTGTAAGAGTGCGCGACCAACTACTTGAGTTCCAAGGCACAGGTATGCAATTTCAAGATCCTTCAGAATTGATGGACATTGGTGCATTAAACACAGTTATTCACCAAGGAATGGACGCCACGGCTGCTTGTATTGGATTGCAAGGGCGCTGGAATGCTTCGCTCATTCAACGCGATTTGATGATATCAGCAGGGGTCTTCACAGGAGGAATTCTCATGATGTGGTATCTTTTCACAAAATGGAGCAAGACAGAAGTGTCACACCAAGGAAAGAACAAGCGCAGCCGGCAAAAACTACGATTCAAAGAGGCTCGTGATAACAAATATGCCTATGATGTAACAGGATCAGAGGAAGTTCTTGGTGAGAATTTTGGAACTGCATATATTAAGAAAGGAAAAGGAAAAGGGACAAAAGTTGGTCTTGGAGTCAAACAACACAAATTCCACATGATGTATGGGTTTGATCCACAAGAATACAACTTAATCCGTTTCGTGGATCCTCTAACAGGTGCGACACTAGACGAGCAAATCCATGCAGATATACGTCTAGTGCAAGAGCACTTCAGCGTCATTAGAGATGAAGCAGTAGCAAATGACACAATTGAAAGACAACATATTTATAGCAATCCTGGATTACAAGCTTTCTTCATACAAAATGGATCGGCAAATGCACTGAGAGTTGATTTAACGCCGCATTCACCCTTGCGTGTCGTGACCAATAACAATATAGCAGGTTTCCCAGAATATGAAGGCACACTTCGGCAAACAGGAACAGCTTTACAAATACCTGTGAATCAAGTTCCAGCTGCGAATGAAACAGGAGTGGCACATGAATCGAAATCAATGATGGCAGGACTAGGTGATTACACACCAATATCACAGCAATTGTGTTTAATTCAAAATGACTCTGATGGAATCAAAAGGAATGTATATTCAATTGGATATGGATCATATCTCATTGCGCCAGCACATTTATTTAAATATAACAATGGTGAAATAACGATCAAATCATCACGAGGCTTGTACAAGATCAGAAATTCAGTTGAAATCAAGTTACATCCCATTGCACATAGAGATATGGTTATAATTCAACTTCCAAAAGATTTTCCACCATTCCCAATGCGCCTTAAGTTTTCAAAACCATCCAGAGAATCAAGGGTGTGCTTAGTTGGAGTGAATTTTCAACAAAACTACAGCACATGCATTGTATCGGAGAGTAGCGTCACAGCACCAAAAGGAAACGGAGATTTCTGGAAACACTGGATATCCACAGTGGACGGACAATGCGGTCTCCCATTAGTAGATGTCAAGAGCAAGCACATAGTTGGAATACACAGTCTTGCATCAACTAGTGGAAACACTAACTTCTTCGTCGCCATGCCTGAAGATTTCAATGACTACATCAACAATCTTGTGCAAACCAACAAGTGGGAAAAAGGATGGCATTACAACCCAAATCTCATTTCATGGTGTGGTCTCAATCTAGTTGACTCAGCTCCAAAGGGTCTTTTCAAAACTTCAAAATTAGTGGAAGACCTTGACATGAGTGTTGAAGAACAATGCAAGGTTACAGAAACATGGCTCACAGAATGTATTCAGGATAATTTACAGGTTGTCGCAAAATGCCCAGGCCAACTTGTCACCAAGCACGTTGTCAAAGGCCCATGCCCACACTTTCAGCTATATCTGTCAACACATGATGAAGCCAAAGCATACTTTGCACCACTACTCGGAAAATACGATAAGAGCAGATTAAACAGAGCAGCTTTTATCAAAGACATTTCAAAATATGCAAAACCAATCTATATTGGAGAAATCAATTACGATGTCTTTGAAAAGGCTATAGAACGTGTGATTAAAATTCTTAAAGATGTGGGAATGCAGCAATGCACGTATGTCACGGATGAGGATGAAATATTCCAGTCACTCAACCTCAATGCTGCAGTTGGTGCCTTATACACAGGAAAGAAGAAAGATTATTTCAAGGATTTTTCAAATGAGGATAAATCAGAAATCATCATGAGATCCTGTGAGCGTATCTACAACGGACAACTTGGCGTGTGGAATGGTTCACTCAAAGCTGAAATAAGGCCTATAGAGAAAACAATGTTAAATAAGACTCGGACTTTTACAGCAGCACCATTAGAAACTTTACTTGGTGGCAAGGTCTGTGTCGATGATTTCAACAACCAATTCTACTCGCACCACTTAGAAGGTCCTTGGACAGTTGGAATAACAAAGTTTTATGGTGGGTGGAACCGTTTGTTGGAAAAATTGCCAGATGGTTGGATATACTGCGACGCCGATGGATCACAGTTCGATAGCTCTTTGACACCATATCTCATCAACGCCGTATTACACATTCGATTACAATTCATGGAAGAATGGAACTTAGGAGAACAAATGTTGCGAAATTTGTACACTGAAATCGTGTACACACCAATTGCAACACCAGATGGATCTGTAATCAAGAAATTTAAAGGAAATAACAGCGGGCAGCCGTCAACAGTTGTAGACAACACACTCATGGTGATATTAGCATTTAATTATGCAATGTTATCAAGTGGTGTTAAAGAGGAAGAAATAGACAATTGCTGCCGAATGTTCGCCAATGGTGATGATCTGCTACTTGCAGTGCATCCAGATTTCGAACACATATTGGACGGATTTCAAAATCACTTTGGAAACCTAGGTCTCAACTTTGAGTTTACATCACGAACAAGAGACAAGTCAGAGTTATGGTTCATGTCCACACGAGGTATCAAATGCGAAGGCATCTATATACCAAAGCTTGAGAAAGAAAGAATAGTTGCCATACTCGAGTGGGATCGGTCAAACTTACCTGAGCACCGTCTCGAAGCCATTTGTGCAGCCATGGTAGAGGCATGGGGATACCCAGACCTTGTCCAAGAGATACGGAAATTTTATGCGTGGCTTCTGGAAATGGAACCATTTGCAAATTTAGCTAAAGAGGGCCTAGCACCATATATCGCTGAAACTGCACTCAGAAATCTGTATTTGGGCACAGGGATCAAGGAAGAAGAGATTGAGAAATATTTCAAACAGTTTGCCAAAGATCTCTCTGGTTATATAGAAGATTACAATGAAGATGTTTTTTATCAATCCGGAACTGTGGATGCAGGAGCCCAAGGAGGGGGAAGTGGATCTGGAACAACGCCACCAGCAACA---------------------------------------------------GGTAGTGGAAC----AGGAACAAGAACACCATCTACTGGAACTCCAGCACAAGGCAACACACCTCCAG---CATCAGGTGGATCATCAGGAAACAATGGAGGC---GGCCAATCAGGTTC---AAACGGCACTGG------------AGGCCA---AGCAGGCTCAAGCGGA------------GCAGGGGGTCAAAGAGACAAAGATGTTGACGCTGGCTCAACAGGGAAGATATCAGTGCCAAAGCTTAAGGCAATGTCGAAGAAAATGCGCCTGCCAAAGGCGAAAGGAAAAGACGTTTTACACTTAGATTTCCTGTTAACATATAAACCCCAACAGCAAGATATATCAAACACTAGAGCAACTAAGGAAGAGTTTGATAGATGGTACGATGCCATAAAGAAGGAGTACGAAATTGATGACACACAAATGACAGTTGTCATGAGTGGTCTCATGGTATGGTGCATCGAAAATGGTTGCTCACCAAACATAAACGGAAATTGGACGATGATGGACGGAGACGAACAAAGGGTTTTTCCATTAAAGCCAGTCATTGAGAACGCATCTCCAACTTTCCGACAAATAATGCATCATTTTAGTGATGCAGCTGAAGCGTATATAGAGTACCGAAACTCTACAGAGCGATACATGCCAAGATACGGTCTTCAGCGAAATCTCACCGACTATAGCTTAGCGCGGTATGCTTTCGATTTCTATGAAATGACTTCGCGCACACCAGCTAGAGCTAAGGAAGCCCACATGCAG---ATGAAAGCCGCAGCAGTTCGTGGTTCAAACACACGTCTGTTCGGTCTGGACGGAAATGTCGGCGAGACTCAGGAGAATACAGAGAGACACACAGCTGGCGACGTTAGTCGCAACATGCACTCTCTGTTGGGAGTGCAGCAGCACCAC--------------------------------------------------------------------------------------------------------------------------------------------------------------------------------------------------------------------------------------------------------------------------------------------

>KR611111

-----------------------------------------------------------------------------------------------------------------------------------------------------------------------------------ATGGCGGGCTCTTGGACTCACGTGACATACAAGTGGCAACCAGATGTCAACAACGCACGTGATGTGAAAAGAGTGATGGAGATGTTTGCAGCAAAACATCAACATTACACTGAGGAGCAAAGGCTTGCTCACAACAGCAAGCTATTAAGGAAGGCTTGTGTCACTAGTGCTGAGTTTATTGAACCAGCACAGAAACCAAAATGTCACCAGACATGGGTTGAAAAGTGCGACCACAACCCCACAGAGCACTTCGTTTATCAACGCTTC---ACACCTGAAAAGAAAGTGCTTAACACCAAACCTGAGACAACCTCTGTCACGAAGTTAATCAGGGATGTCCTTGAAATTTCGAAGGGTAGTGGAATAAAAATTGAGTTAATTGACAAGCGCATCAAACGTAAGACTCAATTATCCATAAGGCAACACAATGGCAAAGATTTCTTGCATTGCAAAACCAGGCATGAAAATGGCTTGTTCAAACGCAAGGACATTGACATTAGTGTCAAGTGGTTACCCACCATTGAAGCCATTGCAAAATGCTACAGCACGGTGAATGCAGAAGAACTACAAAGTCTCAATAGAGGCAGTAGTGGTCTTACATTCATGCAAAACGATGAATTGTTCATCGTGCGTGGAAGGATGCATGGTGAGATTGTTAATAGTTTGCACGAAAGTAAGCATGTTATGGAAATTGAACACTATGCTGATCCACAAGCAAACAGTTTCTGGAAAGGTTATACAGATGCATATGTCGGGAACAGAAACATATCCACCACTCACACAGAGCACACACCAACTATTAATTTAGAGGAGTGTGGCAAGAGAATGGCACTCTTAGAAATCTTATTCCATTCAACTTTCAAAATTACATGCAAAACGTGCAATATTGATGATCTGGAATTATCAGATGATGAATTTGGGGCCAAGTTATACAGTAATCTGCAGCGTATTGAAGAAAGGCAACGCGAATATCTTGCTAAAGATCAAAAACTTTTACGCATGATTCACTTTGTGAAGGACCGGTGTAATCGAAAATTTTCACATTTGCCTTTACTATGGCAAGTGGCAGAAACAGTAGGACATTACACTGATAACCAATCGAAGCAGATAATTGATATCAGTGAGGCGCTCATCAAAGTTAATACCTTAACTCCTGATGATGCAGTGAAGGCCAGTGTAGCATTATTGGAAGTAGCACGATGGTATAAAAATCGGAAGGAATCACTTAAAACAGACACATTGGACTCATTCCGAAACAAAATTTCACCAAAGAGCACGATCAACGCAGCATTGATGTGTGATAACCAGTTAGATAAGAATGGTAATTTCGTATGGGGAAACAGAGAATATCATGCAAAGCGATTCTTCGCTAATTATTTTGAAGCTGTAGACCCAACTGATGCATATGAAAAACACGTCACACGCTTCAATCCCAATGGACAGCGGAAATTATCAATTGGCAAATTAGTAATTCCACTAGATTTCCAGAAAATTAGAGACTCGTTTGTTGGCTTATCGATAAATAAACAACCACTGAGCAAAGCTTGTGTAAGCAAAATTGATGGAGGCTACGTATATCCATGTTGCTGCGTTACAACGGAATTCGGAAAACCAGCATATTCTGAGATAATACCTCCAACAAAAGGACATATCACGATTGGAAATTCAGTGGACCCAAAAATAGTGGATTTACCGAACACAACACCACCAAGTATGTACATTGCAAAAGATGGATATTGTTATATTAATATATTCTTAGCAGCAATGATAAATGTCAATGAGGAATCCGCAAAAGACTACACGAAGTTCCTTAGAGACGAGTTGGTGGAACGGCTCGGCAAGTGGCCTAAACTGAAAGATGTAGCAACAGCATGTTACGCTTTATCAGTAATGTTCCCAGAAATCAAGAATGCCGAATTACCACCAATATTGGTTGATCATGAGAGCAAGTCAATGCATGTCATTGATTCATATGGATCACTCAGTGTTGGATTCCACATTCTAAAGGCAAGTACCGTGGGACAACTGATAAAATTCCAGTACGAATCATTAGAAAGCGAGATGCGTGAATACATAGTGGGAGGTACCTTGACACAACAAACTTTTAACACACTTCTCAAGACTCTCACAAAGAACATGTTCAAGCCAAATAAAATTAAACAGATAATAGAGGAAGAACCTTTCCTACTAATGATGGCGATTGCATCCCCTACCGTGCTTATCTCACTGTATAACAATTGCTACATCGAGCAGGCGATGACATACTGGATTGTCAAGAACCAAGGAGTCGCGGCAATTTTTGCACAGTTGGAGGCTTTGGCAAAGAAAACCTCCCAAGCAGAACTATTAGTTCTGCAAATGCAAATACTTGAAAAAGCTTCTTACCAGCTAAGACTTGCGGTCACCGGACTTAATCATGTCGATCCAGCGAAACGACTTCTGTGGTCTCACCTAGAAGCCATGACAACACGATCGGAAATGAATAAGGAACTCATAGCGGAAGGTTATGCACTATATGATGAGCGTCTATATACCCTAATGGAAAAAAGTTATGTAGATCAATTAAACCAGTCATGGGCAGAATTATCATACTGTGGAAAATTTTCAGCAATATGGCGTGTGTTCAGAGTCAAGAAATACTACAAGCCATCTTTAACCGTGAGAAAAAGCGTAGATTTAGGCGCTGTTTACAATATATCAGCTACGCATCTAATATCAGATTTAGTGCAGAGAAGTCGAGATCGAGTCAGCTCTACTTTAACCAAACTCCGCAACGGTTTCTATGACAAAATGGAAAAAGCGAGGGTTAGCGCAGTGAGGACTGTATATTGGTTCATACCCGATATATTTAGACTAGTCCACATCTTTATAATTCTAAGTTTTTTAACTACTATAGCCAATACAATAGTTACAACCATGAATGACTACAAAAAGTTAAAGAAGCAACAAAGAGAGGACGAGTATGAGGCCGAAATTAATGAGGTGCGGAAAATACATGCCAATTTGATGAAGGAGCGCAATGATAATCTGACATGCGATCAGTTTATTGAATACATACGCCAAACACACCCACGCCTCATCGAAGCAACTTTGGATTTGACACATACAGGCGTTATTCACGAGGGTAAATCCAACTTGGAAACAAACCTTGAACAGGCGATGGCAGTGGGAACTTTACTCACTATGATACTCGACCCACAAAAGAGCGATGCAGTTTACAAGGTTCTCAACAAAATGCGAACAGTGATTAACACAATCGAACAGAATGTGCCATTCCCAACAGTCAATTTTACAAGCATCTTAACGCCTCCTGTAACACAACAGAGTGTTGATGTTGATGAACCATTGACACTAAGTACTGATAAGAACCTGACTATAGATTTTGATACAAATCAAGACTTGCCAGCGGATACATTTAGTAATGACGTTACATTTGAGGATTGGTGGGCTAATCAGATTAACAACAACAGAACAGTGCCACACTACCGACTTGGGGGAAAATTTGTAGAATTCACAAGAGAAAATGCAGCAATGGTCAGCATTGAACTCGCTCACTCAAACATTGAAAAAGAATTCTTACTCAGAGGAGCTGTTGGATCAGGAAAATCCACAGGTTTACCATATCACCTCAGTATGCGTGGAAAAGTGCTATTGATAGAACCTACTCGACCATTAGCTGAGAATGTTTGTAGACAACTGCAAGGTCCTCCATTTAATGTGAGCCCCACTTTACAAATGAGAGGTTTGAGCACATTTGGCTGCACTCCTATCACAATAATGACGTCTGGTTTCGCATTGCATATGTACGCTAATAACCCTGATAAGATCTCTGAATACGACTTCATCATCTTTGATGAATGTCACATCATGGAAGCACCTGCAATGGCATTCTATTGTTTACTTAAGGAGTATGAATATCGAGGCAAGATAATTAAAGTTTCAGCAACACCACCAGGACGAGAATGCGAGTTCTCAACCCAACATCCAGTGGATATACATGTATGCGAAAGTTTGACACAACAACAATTCGTCATGGAATTAGGAACAGGATCAACCGCTGACGCAACCAAATACGGCAATAACATATTAGTGTATGTTGCAAGTTACAATGATGTAGACTCTTTATCCCACGCTCTAACTGAACTCAAATATTCAGTAATCAAAGTTGATGGAAGAACAATGAAGCAGAACACCACAGGAATTGTAACAAATGGAACATCCAGTAAGAAATGCTTCGTTGTAGCCACAAACATCATTGAAAACGGTGTGACGCTAGATGTTGATGTCGTAGTCGACTTTGGACTTAAAGTAACAGCTGAACTGGATGTTGATAACAGAGCAATAATGTATAAACGTGTGAGCATATCTTATGGCGAGCGTATTCAGAGACTCGGAAGAGTTGGAAGGAACAAGCCTGGAACAGTCATCCGCATCGGGAAAACAATGAAAGGTTTACAAGAAATTCCAGCGATGATCGCCACTGAAGCGGCTTTCATGTGTTTCGCATATGGATTGAAGGTCATAACGCACAATGTATCAACAACACATTTGGCAAAATGCACCGTTAAACAAGCCAGAACTATGATGCAGTTTGAGCTTTCACCATTTGTAATGGCCGATCTAGTAAAGTTTGATGGTTCTATGCACCCACAAATCCATGAAGCATTAACTAAGTATAAATTACGAGATTCTGTAATTATGCTAAGACCAAACGCAATACCAAAGGTAAATCTTCACAATTGGTTAACGGCTCGTGATTACAACAGAATAGGCTGCTCATTAGACCTTGAAGATCATGTTAAAATACCATATTACATACGAGGAGTTCCTGACAAGTTGTACGGGAAGTTGTATGAAATTATCCTTCAATACAGCCCTACAAGTTGCTATGGAAGACTATCAAGCGCTTGTGCAGGTAAGGTAGCATATACACTGCGCACTGATCCTTGTTCGTTACCAAGAACAATAGCTATAATCAACGCATTAATCACTGAAGAGTATGCAAAGAGGGATCATTACAGAAACATGATAGCGAACCCTTCGTCATCGCACGCCTTTTCACTTAATGGGCTGGTATCCATGATCGCTTCTCGATACATGAAAGATCACACGAAGGAAAACATTGATAAACTTGTAAGAGTGCGCGACCAGCTACTTGAATTCCAAGGCACAGGTATGCAATTTCAAGATCCTTCAGAATTGATGGACATTGGTGCTTTAAACACAGTTATTCACCAAGGAATGGACGCTACGGCTGCTTGTATTGGACTGCAAGGGCGCTGGAACGCTTCGCTTATTCAACGCGATTTGATGATATCAGCAGGGGTCTTCACAGGAGGAATTCTCATGATGTGGTATCTTTTCACAAAATGGAGCAAGACAGAAGTGTCACACCAAGGAAAGAACAAGCGCAGTCGGCAAAAGCTACGATTCAAAGAGGCTCGTGATAATAAATATGCCTACGATGTAGTAGGATCAGAGGAAGTTCTTGGTGAGAATTTCGGAACTGCATACACTAAGAAAGGAAAAGGAAAGGGGACAAAAGTTGGCCTTGGAGTCAAACAGCACAAATTTCACATGATGTACGGGTTTGATCCACAAGAATACAACTTAATCCGCTTCGTGGATCCTTTAACAGGTGCGACACTAGACGAGCAAATCCATGCAGATATACAGTTAGTGCAAGAGCACTTCAGCGTCATCAGAGATGAAGCAGTAGCAAATGACACAATTGAAAGACAGCACATTTACGCTAATCCTGGATTACAAGCTTTCTTCATACAAAATGGATCGGCAAATGCACTGAGAGTTGATTTGACGCCGCATTCACCCTTGCGTGTCGTGACCAATAACAATATAGCAGGCTTCCCAGAGTATGAGGGTACACTTCGGCAAACAGGAACAGCTCTACAAATACCCGTGAATCAAGTTCCAGTTGCGAACGAAGCGGGGGTGGCACATGAATCGAAATCGATGATGGCAGGGCTAGGTGACTACACTCCAATATCACAGCAATTGTGCCTAGTTCAAAATGACTCAGATGGAATCAAAAGGAATGTATATTCAATTGGATATGGATCATATCTCATTGCGCCAGCACATTTATTTAAGTATAATAATGGCGAAATAACGATCAAATCATCACGAGGCCTATACAAAATCAGAAATTCAGTCGAAATAAAGTTGCATCCCATTGCGCATAGAGACATGGTTATAATTCAACTTCCAAAAGATTTTCCACCATTCCCAATGCGTCTTAAGTTTTCAAAACCATCTAGAGAATCAAGGGTGTGCTTAGTCGGAGTGAATTTTCAGCAGAACTATAGCACATGTATCGTATCGGAGAGTAGCGTCACAGCACCAAAAGGAAACGGAGATTTCTGGAAACACTGGATATCCACAATTGACGGACAATGCGGTCTCCCATTAGTAGATGTCAAGAGTAAGCACATAGTTGGAATACACAGTCTTGCATCAACTAGTGGAAATACTAACTTCTTTGTCGCCATGCCTGAAGACTTCAATGACTACATCAATAATCTTGTGCAAACCAACAAGTGGGAAAAAGGATGGCATTACAACCCAAATCTCATTTCATGGTGTGGTCTCAACCTAGTTGACTCAGCTCCAAAGGGTCTTTTCAAAACTTCAAAATTAGTAGAAGACCTTGATATGAGCGTTGAAGAACAATGTGAGGTTACAGAGACATGGCTCACGGAGTGTATTCAGGATAATTTACAGGTTGTTGCAAAATGTCCAGGCCAACTTGTCACCAAGCACGTTGTCAAAGGCCCATGCCCACACTTTCAGCTATACCTGTCAACACATGATGAAGCTAAAGCATACTTTGCACCATTACTCGGAAAATACGACAAGAGCAGATTAAACAGAGCAGCTTTTATCAAAGACATTTCAAAATATGCAAAACCAATCTACATTGGAGAAATCAATTACGATGTCTTTGAAAAGGCTATACAACGTGTAATTAAAATTCTTAGAGACGTGGGAATGCAGCAATGCACGTATGTAACGGACGAGGATGAAATATTCCAGTCACTTAACCTCAACGCTGCAGTTGGCGCCTTATACACAGGAAAGAAGAAAGATTATTTCAAGGATTTTTCAAATGAGGACAAATCAGAAATTATCATGAGATCCTGTGAGCGTTTATACAACGGACACCTTGGCGTGTGGAATGGTTCACTCAAAGCTGAAATAAGGCCTATAGAGAAAACAATGTTAAATAAGACTCGGACTTTTACAGCAGCACCATTAGAAACTTTACTTGGTGGCAAGGTCTGTGTTGATGATTTCAACAACCAATTCTACTCGCACCACTTAGAAGGTCCTTGGACAGTTGGAATAACAAAGTTTTATGGTGGGTGGAACCGTTTGTTGGAAAAATTGCCAGATGGTTGGATATACTGCGACGCCGATGGATCACAGTTCGACAGCTCTTTGACACCATATCTCATCAACGCCGTATTACACATTCGATTACAATTCATGGAAGAATGGAACTTAGGAGAACAAATGTTGCGAAACTTGTACACTGAAATCGTGTACACACCAATTGCAACACCAGATGGATCTGTAATCAAGAAATTTAAAGGAAATAACAGCGGGCAGCCGTCAACAGTTGTAGACAACACACTCATGGTGATATTAGCATTTAATTATGCAATGTTATCAAGTGGTGTTAAAGAGGAAGAAATAGACAATTGCTGCCGAATGTTCGCCAATGGTGATGATTTGCTACTTGCAGTGCATCCAGATTTCGAACACATACTGGACGGATTTCAAAATCACTTTGGAAACCTAGGTCTCAACTTTGAGTTTACATCACGAACAAGAGACAAGTCAGAGTTATGGTTCATGTCCACACGAGGTATCAAATGCGAAGGCGTCTATATACCAAAACTTGAGAAAGAAAGAATAGTTGCCATACTCGAGTGGGATCGGTCAAACTTACCTGAGCACCGTCTCGAAGCCATTTGTGCAGCCATGGTAGAGGCATGGGGATACCCAGACCTTGTCCAAGAGATACGGAAATTTTATGCGTGGCTTCTGGAAATGGAACCATTTGCAAATTTAGCTAAAGAGGGCCTAGCACCATATATCGCTGAAACTGCACTCAGAAATCTGTATTTGGGCACAGGGATCAAGGAAGAAGAGATTGAGAAATATTTCAAACAGTTTGCCAAAGATCTCTCTGGTTATATAGAAGATTACAATGAAGATGTTTTTTATCAATCCGGAACTGTGGATGCAGGAGCCCAAGGAGGGGGAAGTGGATCTGGAACAACGCCACCAGCAACA---------------------------------------------------GGTAGTGGAAC----AGGAACAAGAACACCATCCACTGGAACTCCAGCACAAGGCAACACACCTCCAG---CATCAGGTGGATCATCAGGAAACAATGGAAGT---GGCCAATCAGGTTC---AAACGGCACTGG------------AGGCCA---AGCAGGCTCAAGCGGA------------ACAGGGGGTCAAAGAGACAAAGACGTTGATGCTGGCTCAACAGGGAAGATATCAGTGCCAAAGCTTAAGGCAATGTCGAAGAAAATGCGCCTGCCAAAGGCGAAAGGAAAAGACGTTTTACACTTAGATTTCCTGTTAACATATAAACCCCAACAGCAAGATATATCAAACACTAGAGCAACTAAGGAAGAGTTTGATAGATGGTACGATGCCATAAAGAAGGAGTACGAAATTGATGACACACAAATGACAGTTGTCATGAGTGGTCTCATGGTATGGTGCATCGAAAATGGTTGCTCACCAAACATAAACGGAAATTGGACGATGATGGACGGAGACGAACAAAGGGTTTTTCCATTAAAGCCAGTCATTGAGAACGCATCTCCAACTTTCCGACAGATAATGCATCATTTTAGTGATGCAGCTGAAGCGTATATAGAGTACCGAAACTCTACAGAGCGATACATGCCAAGATACGGTCTTCAGCGAAATCTCACCGACTATAGCTTAGCGCGGTATGCTTTCGATTTCTATGAAATGACTTCGCGCACACCAGCTAGAGCTAAGGAAGCCCACATGCAG---ATGAAAGCCGCAGCAGTTCGTGGTTCAAACACACGTCTGTTCGGTCTGGACGGAAATGTCGGCGAGACTCAGGAGAATACAGAGAGACACACAGCTGGCGACGTTAGTCGCAACATGCACTCTCTGTTGGGAGTGCAGCAGCACCAC--------------------------------------------------------------------------------------------------------------------------------------------------------------------------------------------------------------------------------------------------------------------------------------------

>KR611110

-----------------------------------------------------------------------------------------------------------------------------------------------------------------------------------ATGGCGGGCTCTTGGACTCACGTGACATACAAGTGGCAACCAGATGTCAACAACGCACGTGATGTCAAAAGAGTGATGGAGATGTTTGTAGCAAAACATCAACGTTACACTGAGGAGCAAAGGCTTGCTCATAACAGCAAGCTATTAAGGAAGGCTTGTGTCATTAGTGCTGAGTTTACCAAACCAGCACAGAAACCAAAATGTCACCAGACATGGGTTGAAAAGTGCGACCACAACCCCACAGAGCACTTCGTTTATCAACGCTTC---ACACCTGAAAAGAAAGTGCTTAGCACCAAACCTGAGACAACTTCTGTTACGAAGTTAATCAGGGATGTCCTTGAAATTTCGAAGGGTAGTGGAATAAAAATTGAGTTAATTGACAAGCGCATTAAACGTAAGACTCAATTATCCATAAGGCAACACAATGGCAAAGATTTCCTGCATTGCAAAACCAGGCATGAAAATGGCTTGTTCAAACGCAAGGACATTGACATTAGTGTCAAGTGGTTACCAACCATTGAAGCCATTGCAAAATGCTACAGCACGGTGAATGCAGAAGAACTACAAAGTCTCAATAGAGGCAGTAGTGGTCTTACATTCATGCAAAACGATGAATTGTTCATCGTGCGTGGAAGGATGCATGGTGAGATTGTTAATAGTTTGCACGAAAGTAAGCATGTTATGGAAATTGAACACTATGCTGATCCACAAGCAAACAGTTTCTGGAAAGGTTATACAGATGCGTATGTCGAGAACAGAAACATATCCACCACTCACACAGAGCACACACCAACTATTAATTTAGAGGAGTGTGGCAAGAGAATGGCACTCTTAGAAATCTTATTCCATTCAACTTTTAAAATTACATGCAAAACGTGTAATATTGATGATCTGGAATTATCAGATGATGAATTTGGGGCCAAGTTATACAGTAATCTGCAGCGTATTGAAGAAAGGCAACGCGAATATCTTGCTAAAGATCAAAAACTTTTACGCATGATTCACTTTGTGAAGGACCGGTGTAATCGAAAATTTTCACATTTGCCTTTACTATGGCAAGTGGCAGAAACAGTAGGACATTACACTGATAATCAATCGAAGCAGATAATTGATATCAGTGAGGCGCTCATCAAAGTTAATACCTTAACTCCTGATGATGCAGTGAAGGCCAGTGTAGCATTATTGGAAGTAGCACGATGGTATAAAAATCGGAAGGAATCACTTAAAACAGACACATTGGACTCATTCCGAAACAAAATTTCACCAAAGAGCACGATCAACGCAGCATTGATGTGTGATAACCAGTTAGATAAGAATGCTAATTTCGTATGGGGAAACAGAGAATACCATGCAAAGCGATTCTTCGCTAATTATTTTGAAGCTGTAGACCCAACTGATGCATATGAAAAACACGTCACACGCTTTAACCCCAATGGACAGCGGAAATTATCAATTGGCAAATTAGTAATTCCACTAGATTTCCAGAAGATTAGAGACTCGTTCGTTGGCCTATCGATAAATAAACAACCACTGAGCAAAGCTTGCGTAAGCAAAATTGATGGAGGCTACGTATATCCATGTTGCTGCGTTACAACGGAATTTGGAAAACCAGCATATTCTGAGATAATACCTCCAACAAAAGGACATATCACGATTGGAAATTCAGTGGACCCAAAAATAGTGGATTTACCGAACACAACACCACCAAGTATGTACATTGCAAAAGATGGATATTGTTATATTAATATATTCTTAGCAGCAATGATAAACGTCAATGAGGAATCCGCAAAAGATTACACGAAGTTCCTTAGAGACGAGTTGGTGGAACGGCTTGGCAAATGGCCAAAATTGAAAGATGTAGCAACAGCATGTTATGCTTTATCAGTAATGTTCCCAGAAATCAAGAATGCTGAATTACCACCAATATTAGTTGATCATGAGAGTAAGTCAATGCACGTCATCGATTCATACGGATCACTCAGTGTTGGATTTCACATTCTAAAGGCAAGCACCGTTGGACAACTGATAAAATTTCAGTATGAATCATTGGAAAGTGAGATGCGTGAATACATAGTGGGAGGTACTTTGACACAACAAACTTTCAGCACACTTCTTAAGACTCTCACAAAGAACATGTTTAAGCCAAATAAGATAAAGCAGATAATAGAGGAAGAGCCTTTCCTACTAATGATGGCAATTGCATCCCCAACTGTACTTATCTCGCTATACAACAACTGCTACATCGAACAGGCAATGACATATTGGATTGTCAAGAACCAAGGAATCGCAGCAATTTTTGCACAGTTGGAGGCATTAGCAAAGAAAACTTCTCAAGCAGAACTACTAGTTCTTCAAATGCAAATACTTGAAAAAGCTTCTTACCAACTGAGACTTGCAGTTACCGGACTTAATCATGTTGATCCAGCAAAACGACTTTTATGGTCTCACCTAGAAGCCATGACAACACGATCGGAGATGAATAAGGAACTCATAGCGGAAGGTTATGCACTATATGACGAGCGCTTATATACTTTAATGGAAAAAAGTTACGTAGATCAATTAAACCAATCATGGGCAGAATTATCATACTGTGGAAAATTTTCAGCAATATGGCGTGTGTTCAGAGTCAGGAAATACTACAAACCATCTTTAACCGTGAGAAAAAGCGTAGATTTAGGCGCTGTTTACAATATATCAGCTACGCATCTAATATCAGATTTAGTGCAGAGAAGTCGAGATCAAGTCAGCTCTACTTTAACCAAACTCCGCAACGGTTTCTATGATAAAATGGAGAGAGCGAGAGTTAGTGCAGTAAGGACAATATATTGGTTTATACCTGATATATTTAGATTAATCCATATTTTCATAGTTTTAAGTTTTTTAACAACTATAGCTAATACAATAGTCACAACTATGAATGATTATAAAAAGTTGAAAAAGCAACAAAGAGAAGACGAATATGAAGCCGAGATTAATGAGGTACGGAAAATACACGCCAATCTGATGAAGGAGCATAATGATAATCTGACATGCGATCAATTTATTGAACACATACGCCAGACACATCCACGCCTCATCGAAGCAACATTGGATTTAACACATACAGGTGTCATCCATGAGGGTAAATCCAATTTGGAAACCAACCTCGAACAGGCGATGGCAGTGGGAACTTTACTCACTATGATACTCGATCCACAGAAGAGCGATGCAGTTTATAAGGTTCTCAATAAAATGCGAACAGTGATTAGCACAATCGAACAGAATGTGCCATTCCCATCAGTCAACTTCACAAGCATCTTGACACCTCCTGTAACACAGCAGAGTGTTGATGTTGACGAACCATTAACACTGAGTACCGACAAGAATTTGACTATAGATTTTGACACAAATCAAGATTTGCCAGCAGATACATTTAGCAATGACGTTACATTCGAGAACTGGTGGGCTAATCAGATAAACAACAACAGAACAGTGCCACACTATCGACTTGGGGGAAAGTTTGTAGAATTCACAAGAGAAAATGCAGCAATGGTTAGCATTGAACTTGCTCACTCGAACATCGAAAAAGAATTTCTACTCAGAGGAGCTGTTGGATCAGGAAAATCCACAGGTTTGCCATATCATCTCAGTATGCGTGGAAAAGTGCTATTGATAGAACCTACTCGACCGTTAGCTGAGAACGTTTGCAGGCAACTGCAAGGTCCTCCATTTAATGTGAGTCCCACTCTACAAATGAGAGGTTTGAGCACATTTGGCTGCACTCCTATCACGATAATGACGTCTGGTTTCGCATTGCACATGTATGCTAATAACCCCGATAAGATTTCTGAATACGACTTCATCATCTTTGATGAATGTCACATTATGGAAGCACCTGCAATGGCATTTTATTGTTTGCTTAAGGAGTATGAATACCGAGGCAAGATAATAAAAGTTTCAGCTACACCACCAGGACGAGAATGCGAATTTTCAACCCAACATCCAGTAGATATACATGTATGTGAAAGCTTGACACAACAGCAATTCGTCATGGAACTAGGAACAGGATCAACTGCTGACGCAACCAAATATGGCAATAACATATTAGTGTACGTTGCAAGTTATAATGACGTAGATTCTTTATCCCATGCTCTAACTGAACTTAAGTATTCAGTAATCAAAGTCGACGGAAGAACAATGAAGCAGAACACCACAGGAATCGTAACAAATGGAACATCCAGTAAGAAATGCTTCGTTGTGGCCACGAACATTATTGAAAACGGTGTAACGCTAGATGTCGATGTCGTCGTCGACTTTGGACTTAAAGTAACAGCCGAATTAGATGTTGATAACAGGGCGATAATGTATAAACGTGTGAGCATATCTTATGGCGAGCGCATTCAGAGACTCGGAAGAGTTGGAAGGAATAAGCCTGGAACAGTAATCCGCATCGGGAAAACAATGAAAGGTTTACAAGAAATTCCAGCGATGATTGCCACTGAAGCAGCTTTCATGTGTTTTGCATACGGACTGAAGGTCATAACACATAATGTATCAACAACACATCTGGCAAAATGCACTGTCAAACAAGCCAGAACCATGATGCAATTTGAGCTATCACCATTTGTAATGGCTGATTTAGTCAAATTTGACGGTTCTATGCACCCACAGATTCATGAAGCATTAACCAAATATAAATTGAGAGATTCTGTAATCATGTTAAGACCAAATGCAATACCAAAGGTAAATCTTCACAACTGGCTGACGGCTCGTGATTACAACAGAATAGGCTGCTCATTGGAACTCGAAGACCACGTCAAAATACCATATTATATACGGGGAGTTCCTGACAAGTTGTATGGGAAGTTATATGATATTATCCTTCAATATAGCCCTACAAGTTGCTATGGAAGACTATCAAGTGCTTGTGCAGGCAAAGTAGCATATACATTGCGCACTGATCCTTGTTCGTTACCAAGAACAATAGCTATAATCAACGCACTGATTACTGAAGAATACGCAAAGAGGGATCATTACAGAAACATGATAGCGAACCCTTCATCATCGCACGCCTTTTCACTTAATGGGCTAGTATCCATGATCGCTTCTCGGTACATGAAAGACCACACGAAGGAAAATATTGAAAAACTTGTAAGAGTACGCGATCAACTACTTGAGTTCCAAGGCACAGGCATGCAGTTTCAAGATCCTTCAGAATTAATGGACATTGGTGCATTAAACACAGTTATTCACCAAGGAATGGACGCCACGGCTGCTTGTATTGGATTGCAAGGGCGTTGGAATGCTTCACTCATTCAGCGCGATTTGATGATATCAGCAGGGGTCTTCACAGGAGGAATTCTTATGATGTGGTGTCTTTTCACAAAATGGAGTAAAACAGAAGTGTCACACCAAGGAAAGAACAAGCGTAGTCGGCAAAAATTACGATTCAAAGAGGCTCGTGATAACAAATACGCCTATGACGTAACAGGATCAAAGGAAGCTATCGAAGAAAATTTTGGATCCGCATATACTAAGAAAGGAAAAGGTAAGGGGACAAAAGTTGGTTTTGGAGTCAAGCAACACAAATTCCACATGATGTATGGGTTTGATCCTCAAGAGTACAACCTCATTCGTTTTGTCGATCCACTTACAGGAGCCACATTGGATGAACAGATCCATGCCGATATACGTCTAGTGCAAGAACATTTCGATGTTCTTCGGGAGGAAGCAGTAGCAAACGACACGATTGAGAGACAGCATATATATAGCAGTCCCGGTTTGCAAGCATTTTTCATACAGAATGGATCAGCAAATGCATTAAGAGTTGATCTAACGCCGCACTCACCCTTACGTGTTGTGACAAACAACAATATAGCAGGCTTCCCAGAATACGAAGGTACTCTTCGTCAAACAGGAACTGCCATCACTATACCTGTAAACCAAGTTCCAGTAGCGAATGAAACAGGAGTGGCACACGAATCGAAATCAATGATGATTGGACTAGGTGATTACACACCAATTTCACAACAATTGTGCTTAGTTCAAAATGACTCTGACGGGGTGAAAAGAAACGTATTTTCAATTGGATATGGATCGTACCTTATATCACCAGCGCATTTATTCAAGTATAATAATGGTGAAATTACGATTAAGTCATCGAGGGGTTTGTATAAGATAAGAAATTCAGTAGAACTCAAGTTACATCCTATTGCACACAGAGATATGGTTATAATTCAACTCCCTAAAGATTTCCCACCATTTCCAATGCGCCTTAAATTTTCAAAACCAACACGGGAATCACGGGTGTGCTTAGTTGGAGTAAATTTTCAACAAAATTACAGTACCTGCATTGTATCAGAGAGCAGCGTGACAGCACCAAAAGGAAATGGAGATTTTTGGAAACACTGGATATCTACAGTGGATGGACAATGCGGTCTTCCATTAGTAGATGTTAAAAGCAAGCATATAGTCGGAATACATAGTCTTGCATCAACGAGTGGAAACACAAACTTCTTTGTCGCCATACCGGAAAACTTCAATGAGTACATCAGCAATCTTGTGCAAACAAACAAGTGGGAAAAAGGATGGCACTACAATCCAAATCTCATTTCATGGTGTGGTTTAAATTTAGTTGATTCAGCACCTAAAGGCTTATTCAAGACATCAAAACTAGTTGAAGACTTAGACATGAGTGTTGAAGAACAATGCAAGGTGACAGAAACATGGCTCACAGAATGTATCCAGGACAATCTACAAGTTGTGGCAAAATGTCCAGGCCAACTCGTAACTAAACACGTTGTCAAAGGCCCGTGTCCACACTTTCAATTGTATTTATCAACACATGACGAAGCCAAAACATACTTTGCCCCACTACTTGGAAAGTATGATAAGAGCAGATTGAACAGAGCAGCATTCATCAAGGATATATCAAAGTATGCGAAACCGATTTATGTTGGTGAAATTAACTACGATATCTTTGAAAAGGCAATAGAGCGAGTTATTAAGATTCTTAAAAACGTCGGCATGCAGCAATGCGTTTATGTCACGGATGAAGAAGAAATTTTCAACTCACTCAATCTTAATGCAGCCGTCGGCGCCTTATACACAGGCAAGAAGAAGGATTATTTCAAGGATTATTCAAATGAGGATAAAGCCGAAATCATCATGCGATCCTGTGAGCGGATCTACAATGGACAACTTGGCATCTGGAATGGGTCACTCAAAGCTGAAATACGCCCAATTGAGAAAACCATGTTGAATAAAACACGCACTTTCACAGCAGCACCATTGGAAACTCTACTTGGTGGAAAGGTTTGCGTGGACGATTTTAATAATCAGTTTTATTCGCATCACCTTGAAGGCCCATGGACAGTTGGAATCACAAAATTTTATGGAGGATGGAATCGTCTACTCGAGAAGTTACCAGAAGGCTGGATTTATTGCGACGCCGATGGTTCACAATTTGATAGCTCATTAACGCCATACCTTATCAACGCCGTTTTACATATTCGGCTGCAATTTATGGAAGAATGGGCATTAGGGGCACAAATGTTGCAAAATCTGTACACCGAAATTGTTTACACACCAATTGCAACGCCAGATGGATCAGTCATTAAGAAATTCAAAGGAAACAACAGTGGCCAGCCCTCTACAGTTGTTGATAACACACTCATGGTCATATTAGCATTCAATTATGCAATGTTATCGAGTGGTATCAAAGAAGATGAAATAGACAACTGCTGCCGAATGTTCGCCAATGGAGACGATCTATTGCTGGCAGTGCATCCGGACTTTGAACATATACTGGATGGATTTCAAAATCACTTTGGAAATTTAGGCCTCAATTTTGAGTTTACATCACGAACAAAAGACAAATCAGAACTGTGGTTCATGTCCACACGAGGTATCAAATGTGAAGGCGTCTATATACCAAAGCTTGAGAAAGAAAGAATAGTTGCCATACTCGAGTGGGATCGGTCAAACTTACCTGAGCACCGTCTCGAAGCTATATGCGCAGCCATGGTAGAAGCATGGGGATATCCAGACCTTGTTCAAGAAATACGAAAATTCTATGCGTGGCTTCTGGAAATGGAACCATTTGCAAATCTAGCGAAAGAGGGCTTAGCACCATATATAGCAGAAACCGCACTCAGAAATTTGTACTTGGGCACAGGAATCAAGGAAGAGGAGATTGAAAAATACTATAAACAGTTTGCTAAAGATCTCTCTGGCTATATAGAAGACTATAATGAAGATGTTTTTTATCAATCCGGTTCTGTGGATGCAGGAGCTCAAGGAGGAAGAAGTGGCTCAGGAACAACCCCACCTGCAGCG---------------------------------------------------GGTAGTGGAAC----AGGAACTAGACCACCTTCAACTGGATCAGCAGCACAAGGAAACACACCTCCAG---CATCAGGTGGATCATCAGGAAACAATGGAAGC---AATCAATCAGGTTC---AAATGGCACCGG------------AAACCA---AGCAGGCTCAAGCGGA------------ACAGGGGGCCAAAGAGACAAAGATGTTGACGCTGGTTCAACAGGAAAGATATCAGTGCCAAAACTTAAGGCCATGTCGAAGAAAATGCGCCTGCCAAAGGCAAAAGGAAAAGATGTCTTACACTTAGATTTTCTGTTGACATATAAACCCCAACAACAAGACATATCAAACACTAGAGCAACCAAGGAAGAGTTTGATAGATGGTATGACGCCATAAAGAAGGAGTATGAAATTGATGACACACAAATGACAGTTGTCATGAGTGGTCTCATGGTATGGTGCATCGAAAATGGTTGCTCACCAAACATAAACGGAAATTGGACGATGATGGATGGAGATGAACAAAGGGTTTTTCCATTGAAACCAGTTATTGAAAATGCATCTCCAACTTTCCGACAAATTATGCATCACTTCAGTGATGCAGCTGAAGCGTATATAGAGTACCGAAACTCTACAGAGCGATATATGCCAAGATACGGACTTCAGCGAAATCTCACCGACTATAGCTTAGCACGGTATGCTTTTGATTTCTATGAAATGACTTCACGCACACCAGCTAGAGCTAAGGAAGCCCACATGCAG---ATGAAGGCCGCAGCAGTTCGTGGTTCAAACACACGCCTGTTCGGTCTGGACGGAAATGTCGGCGAGACTCAGGAGAATACAGAGAGACACACAGCTGGCGACGTTAGTCGCAACATGCACTCTCTGTTGGGAGTGCAGCAGCACCAC--------------------------------------------------------------------------------------------------------------------------------------------------------------------------------------------------------------------------------------------------------------------------------------------

>KR611109

-----------------------------------------------------------------------------------------------------------------------------------------------------------------------------------ATGGCGGGCTCTTGGACTCACGTGACATACAAGTGGCAACCAGATGTCAACAACGCACGTGATGTGAAAAGAGTGATGGAGATGTTTGCAGCAAAACATCAACGTTACACTGAGGAGCAGAGGCTTGCTCACAACAGCAAGCTATTAAGGAAGGCTTGTGTCACTAGTGCTGAGTTTGTTGAACCAGCACAGAAACCAAAATGTCACCAGACATGGGTTGAAAAGTGCGACCACAACCCCACAGAGCACTTTGTTTATCAACGCTTC---ACACCTGAGAAGAAAGTGCTAAGCATCAAACCTGAAACAACTTCTGTCACGAAGTTAATCAGGGATGTCCTAGAAATTTCGAAGGGTAGTGGGATAAAAATTGAGTTAATTGACAAGCGTGTCAAACGTAAGACTCAATTATCCATAAGGCAACACAATGGCAAAGATTTCTTGCACTGCAAAACCAGGCATGAAAATGGCCTGTTTAAACGCAAGGACATTGACATTAGTGTTAAGTGGTTGCCCACCATTGAAGCCATTGCAAAATGCTACAGCACGGTGAATGCAGAAGAACTGCAAAGTCTCAATAGAGGCAGTAGTGGTCTTACATTCATGCAAAACGATGAATTGTTCATCGTGCGTGGAAGGATGCATGGTGAGATTGTTAATAGTTTACACGAGAATAAGCACGTTATGGAAATTGAACACTATGCTGATCCACAAGCAAACAGTTTCTGGAAAGGTTATACAGATGCGTATGTCGAGAACAGAAACATATCTACCACTCACACAGAGCACACACCAACTATTAATTTAGAGGAGTGTGGCAAGAGAATGGCACTCTTAGAAATCTTATTCCATTCAACTTTTAAAATAACATGCAAAACGTGTAATATTGATGACCTGGAATTATCAGATGATGAATTTGGGGCCAAGTTATACAGTAATCTGCAGCGCATTGAAGAAAGGCAACGCGAATATCTTGCTAAAGATCAAAAACTTTTACGCATGATTCACTTTGTGAAGGACCGGTGTAATCGAAAATTTTCACATTTGCCTTTGCTATGGCAAGTGGCAGAAACAGTAGGACATTACACTGATAATCAATCGAAGCAGATAATTGATATCAGTGAGGCGCTCATCAAAGTTAATACCTTAACTCCTGATGATGCAGTGAAGGCCAGTGTAGCATTATTGGAAGTAGCACGATGGTATAAAAATCGGAAGGAATCACTTAAAACAGACACATTGGACTCATTCCGAAACAAAATTTCACCAAAGAGCACGATCAACGCAGCATTGATGTGTGACAACCAGTTAGATAAGAATGCTAATTTCGTATGGGGAAACAGAGAATACCATGCAAAGCGATTCTTCGCTAATTATTTTGAAGCTGTAGACCCAACTGATGCATATGAAAAACACGTCACACGCTTTAATCCCAATGGACAGCGGAAATTATCAATTGGCAAATTAGTAATTCCACTAGATTTCCAGAAAATTAGAGACTCGTTTGTTGGCCTATCGATAAATAAACAACCACTGAGCAAAGCTTGTGTAAGCAAAATTGATGGAGGCTACGTATATCCATGTTGCTGCGTCACAACGGAATTCGGAAAACCAGCATATTCTGAGATAATACCTCCAACAAAAGGACATATCACGATTGGAAATTCAGTGGACCCAAAAATAGTGGATTTACCGAACACAACACCACCAAGCATGTACATTGCAAAAGATGGATATTGTTATATTAATATATTCTTAGCAGCAATGATAAATGTCAATGAGGAATCCGCAAAAGACTACACGAAGTTCCTTAGAGATGAGTTGGTGGAACGGCTTGGCAAATGGCCTAAACTGAAAGATGTAGCAACAGCATGTTACGCTTTATCAGTAATGTTCCCAGAAATCAAGAATGCTGAATTACCACCAATATTAGTTGATCATGAGAGCAAGTCAATGCATGTCATCGATTCATATGGCTCACTCAGTGTTGGATTTCACATTCTAAAGGCAAGTACTGTGGGACAACTGATAAAATTCCAGTATGAATCATTAGAAAGCGAGATGCGTGAATACATAGTGGGAGGCACTTTGACACAACAAACTTTCAGCACACTTCTCAAGACTCTCACAAAGAACATGTTTAAGCCAGATAAGATTAAACAGATAATAGAGGAAGAGCCTTTCCTACTAATGATGGCGATTGCATCCCCAACCGTACTTATCTCACTGTATAACAACTGCTACATCGAACAGGCAATGACATATTGGATTGTCAAGAACCAAGGAGTTGCAGCAATTTTTGCACAGTTGGAGGCTTTGGCAAAGAAAACCTCTCAAGCAGAACTATTAGTTCTGCAAATGCAAATACTTGAAAAAGCTTCTTACCAACTAAGACTTGCAGTCACCGGACTTAATCATGTTGATCCAGCGAAACGACTTTTGTGGTCTCACCTAGAAGCCATGACAACACGATCGGAAATGAACAAGGAACTCATAGCGGAAGGTTATGCACTGTATGACGAGCGTCTATATACTCTAATGGAAAAAAGTTATGTAGATCAATTAAACCAATCATGGGCAGAATTATCATACTGTGGAAAATTTTCAGCAATATGGCGTGTGTTCAGAGTCAGGAAATATTACAAGCCATCTTTAACCGTGAGAAAAAGCGTAGATTTAGGCGCTGTTTACAATATATCAGCTACGCATCTAATATCAGATTTAGTGCAGAGAAGTCGCGATCAAGTCAGCTCTACTTTAACCAAACTCCGCAACGGTTTCTATGACAAAATGGAGAAAGCGAGGGTTAGTGCAGTGAGGACAGTATATTGGTTTATACCTGATATATTTAGATTAGTCCATATCTTTATAGTTCTAAGTTTTTTAACTACTATAGCTAATACAATAGTCACAACTATGAATGACTACAAAAAGTTAAAGAAACAACAAAGAGAGGATGAGTATGAAGCTGAAATTAATGAGGTGCGGAAAATACACGCCAATTTGATGAAGGAGCACAATGATAATCTGACATGTGATCAATTTATTGAATACATACGCCAAACACACCCACGCCTCATCGAAGCAACTTTGGATTTAACACATACAGGTGTTATTCACGAAGGTAAATCCAACCTGGAAACAAATCTCGAACAGGCGATGGCAGTGGGAACTTTACTCACTATGATACTCGACCCACAAAAGAGTGATGCAGTTTACAAGGTTCTCAATAAGATGCGAACAGTGATTAGCACAATTGAGCAGAATGTGCCATTTCCAACAGTTAATTTTACAAGCATCTTAACACCTCCTGTAACACAGCAAAGTGTTGATGTTGATGAACCATTGACATTGAGTACTGACAAGAATCTGACTATAGATTTTGATACAAACCAAGATTTGCCAGCAGATACATTTAGCAATGACGTTACATTCGAGAACTGGTGGGCTAACCAGATAAACAACAACAGAACAGTGCCACACTATCGACTTGGGGGAAAGTTTGTAGAATTCACAAGAGAAAATGCAGCAATGGTTAGCATTGAGCTCGCTCACTCGAACATCGAAAAAGAATTTCTACTTAGAGGAGCTGTTGGATCAGGAAAATCCACAGGTTTGCCATATCATCTCAGTATGCGTGGAAAAGTGCTATTGATAGAACCCACTCGACCGTTAGCTGAGAACGTTTGCAGGCAACTGCAAGGTCCCCCATTCAACGTGAGTCCCACTTTACAAATGAGAGGTTTGAGCACATTTGGCTGCACTCCTATCACGATAATGACGTCTGGTTTCGCATTACACATGTATGCTAATAACCCTGACAAGATTTCTGAATACGATTTCATTATCTTTGATGAATGTCACATCATGGAAGCACCTGCAATGGCATTTTATTGTTTGCTTAAGGAGTATGAATACCGAGGCAAGATAATAAAAGTTTCAGCTACACCACCAGGACGAGAATGCGAATTTTCAACCCAACATCCAGTAGATATACATGTATGTGAGAGCTTGACACAACAGCAATTCGTTATGGAACTAGGAACAGGATCAACTGCTGATGCAACCAAATATGGCAATAACATATTAGTGTACGTTGCAAGTTACAATGACGTAGATTCTTTATCCCATGCTCTAACTGAACTTAAGTATTCAGTAATTAAAGTCGACGGAAGAACAATGAAGCAGAACACCACAGGGATCGTAACAAATGGAACATCCAGTAAGAAATGCTTCGTTGTGGCCACGAATATTATTGAAAACGGTGTAACGCTAGATGTCGATGTCGTCGTCGACTTTGGACTTAAAGTAACAGCTGAATTAGATGTTGATAACAGGGCAATAATGTATAAACGTGTGAGCATATCTTATGGCGAGCGCATTCAGAGACTTGGAAGAGTTGGAAGGAATAAGCCTGGAACAGTAATCCGCATCGGGAAAACAATGAAAGGTTTACAAGAAATTCCAGCGATGATTGCAACTGAAGCGGCTTTCATGTGTTTCGCATATGGACTGAAAGTTATAACACATAATGTATCAACAACACATCTGGCAAAATGCACTGTCAAACAAGCTAGAACCATGATGCAATTTGAACTTTCACCATTTGTAATGGCTGATTTAGTTAAATTTGACGGTTCTATGCACCCACAGATTCATGAAGCATTAACCAAGTACAAATTGAGAGATTCTGTGATCATGTTAAGACCAAATGCAATACCAAAGGTAAATCTTCACAACTGGCTGACGGCTCGTGATTACAACAGGATAGGCTGCTCATTGGAACTCGAAGACCACGTTAAAATACCATATTATATACGGGGAGTTCCTGACAAGCTGTACGGGAAGTTGTATGATATCATCCTTCAATATAGCCCTACAAGTTGCTATGGAAGACTATCAAGTGCTTGTGTAGGCAAGGTTGCATATACATTGCGCACTGATCCTTGTTCGTTACCAAGAACAATAGCTATAATCAACGCACTGATTACTGAAGAATACGCAAAGAGGGATCATTACAGAAACATGATAGCGAACCCTTCATCATCACACGCCTTCTCACTTAATGGGCTAGTATCCATGATCGCTTCTCGGTACATGAAAGACCACACGAAGGAAAATATTGAAAAACTTGTAAGAGTACGCGATCAACTACTTGAGTTCCAAGGCACAGGCATGCAGTTTCAAGATCCTTCAGAATTAATGGACATTGGTGCATTAAACACAGTTATTCACCAAGGAATGGACGCCACGGCTGCTTGTATTGGATTGCAAGGGCGTTGGAATGCTTCACTTATTCAGCGCGATTTGATGATATCAGCAGGGGTCTTCACAGGAGGAATTCTTATGATGTGGTGTCTTTTCACAAAATGGAGCAAAACAGAAGTGTCACACCAAGGAAAGAACAAGCGTAGTCGGCAAAAACTACGATTTAAAGAGGCTCGTGATAACAAATACGCCTACGACGTAACAGGATCAAAGGAAGCGATCGAAGAAAATTTTGGATCCGCATATACTAAGAAAGGAAAAGGTAAAGGGACAAAAGTTGGTTTTGGAGTCAAGCAACACAAATTCCACATGATGTATGGGTTTGATCCTCAAGAATACAACCTCATTCGTTTTGTCGATCCACTCACAGGAGCCACATTGGATGAACAGATCCATGCCGATATACGTTTAGTGCAAGAACATTTCGATGTTCTTCGGGAGGAAGCAGTAGCAAACGACACGATTGAGAGACAACATATATATAGCAGTCCCGGTTTGCAAGCATTTTTCATACAGAATGGATCAGCAAATGCATTAAGAGTTGATCTAACGCCGCACTCACCCTTACGTGTTGTGACAAACAACAATATAGCAGGTTTCCCAGAATACGAAGGCACTCTTCGTCAAACAGGAACTGCCATCACTGTACCTGTAAACCAAGTTCCAGTAGCGAATGAAACAGGAGTGGCACACGAATCGAAATCAATGATGATTGGACTAGGTGATTACACACCAATTTCACAGCAATTGTGCTTAGTTCAAAATGATTCTGACGGGGTGAAAAGAAACGTATTTTCAATTGGATATGGATCGTACCTTATATCACCAGCGCATTTATTCAAGTATAATAATGGTGAAATTACGATTAAGTCATCGAGGGGTTTGTATAAGATAAGAAATTCAGTAGAACTCAAATTACACCCTATTGCACACAGAGATATGGTTATAATTCAACTCCCTAAAGATTTCCCACCATTTCCAATGCGCCTTAAATTTTCAAAACCAACACGGGAATCACGGGTGTGCTTGGTTGGAGTAAATTTTCAGCAAAATTACAGTACCTGCATTGTATCAGAGAGCAGCGTGACAGCACCAAAAGGAAATGGAGATTTTTGGAAACACTGGATATCTACAGTGGATGGACAATGTGGTCTTCCATTAGTAGATGTTAAAAGCAAGCACATAGTCGGAATACATAGTCTTGCATCAACGAGTGGAAACACAAATTTCTTCGTCGCCATACCGGAAAACTTTAATGAGTACATCAGCAATCTTGTGCAAACAAACAAGTGGGAAAAAGGATGGCACTACAATCCAAATCTCATTTCATGGTGTGGTTTAAATTTAGTTGACTCAGCACCTAAAGGCTTATTCAAGACATCAAAACTAGTTGAAGACTTAGACACGAGTGTTGAAGAACAATGCAAGGTGACAGAAACATGGCTCACAGAATGTATCCAGGACAATCTACAAGTTGTGGCAAAATGCCCAGGTCAACTCGTAACTAAACACGTTGTCAAAGGTCCGTGTCCACACTTTCAATTATATTTATCAACACATGACGAAGCCAAAACATACTTTGCCCCACTACTTGGAAAGTATGATAAGAGCAGATTGAACAGAGCAGCATTCATCAAGGATATATCAAAGTATGCGAAGCCGATTTACGTAGGTGAAATTAACTACGATATCTTTGAAAAGGCAATAGAGCGAGTGATCAAGATTCTTAAAAATGTCGGCATGCAGCAATGCGTTTATGTCACGGATGAAGAAGAAATTTTCAATTCACTCAATCTTAATGCAGCCGTCGGTGCCTTATACACAGGCAAGAAGAAGGATTATTTCAAGGATTATTCAAATGAGGATAAAGCTGAAATCATCATGCGATCCTGTGAGCGGATCTTCAATGGACAACTTGGCATCTGGAATGGGTCACTCAAAGCCGAAATACGCCCAATTGAGAAAACCATGTTGAATAAAACACGCACTTTCACAGCAGCACCATTGGAAACTCTACTTGGTGGAAAGGTTTGCGTGGACGATTTTAATAATCAGTTTTACTCGCATCACCTTGAAGGCCCATGGACAGTTGGAATCACAAAATTTTATGGAGGATGGAATCGTCTACTCGAGAAGTTACCAGAAGGCTGGATTTATTGCGACGCCGATGGTTCACAATTTGATAGCTCATTAACGCCATACCTTATCAACGCCGTTTTACATATTCGGCTGCAATTTATGGAAGAATGGGCACTAGGGGCACAAATGTTGCAAAATCTGTACACCGAAATCGTATACACACCAATTGCAACGCCAGACGGATCAGTCATTAAGAAATTCAAAGGAAACAACAGTGGCCAGCCCTCTACAGTTGTTGATAACACACTCATGGTCATATTAGCATTCAATTACGCAATGTTATCGAGTGGTATCAAAGAAGATGAAATAGACAACTGCTGCCGAATGTTCGCCAATGGAGACGATCTATTGTTGGCAGTGCACCCGGACTTTGAACATATATTGGATGGATTTCAAAATCACTTTGGAAATTTAGGTCTCAATTTTGAGTTCACATCACGAACAAAAGACAAATCAGAACTGTGGTTCATGTCCACACGAGGTATCAAATGTGAAGGCATCTATATACCAAAACTTGAGAAAGAAAGAATAGTTGCCATACTCGAATGGGATCGATCAAACCTACCTGAGCACCGTCTCGAAGCTATATGCGCAGCCATGGTAGAAGCATGGGGATATCCAGACCTTGTTCAAGAAATACGAAAATTCTATGCGTGGCTTCTGGAAATGGAACCATTTGCAAATCTAGCGAAAGAGGGCTTAGCACCATATATAGCAGAAACCGCACTCAGAAATTTGTACTTGGGCACAGGAATCAAGGAAGAGGAGATTGAAAAATACTTTAAACAGTTTGCAAAAGATCTCTCTGGCTATATAGAAGATTATAATGAAGATGTTTTTTATCAATCCGGTTCTGTGGATGCAGGAGCTCAAGGAGGAAGAAGTGGCTCAGGAACAACCCCACCTGCAGCG---------------------------------------------------GGTAGTGGAAC----AGGAACTAGACCACCTTCAACTGGATCAGCAACACAAGGAAACACACCTCCAG---CATCAGGTGGATCATCGGGAAATAATGGAGGT---AACCAATCAGGTTC---GAACGGCACTGG------------AAATCA---AGCAGGCTCAAACGGA------------ACAGGGGGCCAAAGAGACAAAGATGTTGATGCTGGTTCAACAGGAAAAATATCAGTGCCAAAGCTTAAGGCAATGTCAAAGAAAATGCGCCTGCCAAAAGCAAAAGGAAAAGATGTTTTACACTTAGATTTCCTTTTAACATATAAACCCCAACAACAAGACATATCAAACACTAGAGCAACCAAGGAAGAGTTTGATAGATGGTATGACGCCATAAAGAAGGAGTATGAAATTGATGATACACAAATGACAGTTGTCATGAGTGGTCTCATGGTATGGTGCATTGAAAATGGTTGCTCACCAAACATAAACGGAAATTGGACGATGATGGACGGAGATGAACAAAGAGTGTTTCCATTGAAACCAGTTATTGAAAATGCATCTCCAACTTTCCGACAAATTATGCATCACTTCAGTGATGCAGCTGAAGCGTATATAGAATACCGAAACTCTACAGAGCGATATATGCCAAGATACGGACTTCAGCGAAATCTCACCGACTATAGCTTAGCACGGTATGCTTTTGATTTCTATGAAATGACTTCACGCACACCAGCTAGAGCTAAGGAAGCCCACATGCAG---ATGAAGGCCGCAGCAGTTCGTGGTTCAAACACACGCCTGTTCGGTCTGGACGGAAATGTCGGCGAGACTCAGGAGAATACAGAGAGACACACAGCTGGCGACGTTAGTCGCAACATGCACTCTCTGTTGGGAGTGCAGCAGCACCAC--------------------------------------------------------------------------------------------------------------------------------------------------------------------------------------------------------------------------------------------------------------------------------------------

>KR611107

-----------------------------------------------------------------------------------------------------------------------------------------------------------------------------------ATGGCGGGCTCTTGGACTCACGTGACATACAAGTGGCAACCAGATGTCAACAACGCACGTGATGTCAAAAGAGTGATGGAGATGTTTGCAGCAAAACATCAACGTTACACTGAGGAGCAAAGGCTTGCTCACAACAGCAAGCTATTAAGGAAGGCTTGTGTCATTAGTGCTGAGTTTGTTGAACCAGCACAGAAACCAAAATGTCACCAGACATGGGTTGAAAAGTGCGACCACAACCCCACAGAGCACTTCGTTTATCAACGCTTC---ACACCTGAAAAGAAAGTGCTAAACACCAAACCTGAAACAACTTCTGTCACGAAGTTAATCAGGGATGTCCTTGAAATTTCGAAGGGTAGTGGAATAAAAATTGAGTTAATTGACAAGCGTATCAAACGTAAGACTCAATTATCCATAAGGCAACACAATGGCAAAGATTTCTTGCATTGCAAAACCAGGCATGAAAATGGCTTGTTCAAACGCAAGGACATTGACATTAGTGTCAAGTGGTTACCCACCATTGAAGCCATTGCAAAATGCTACAGCACGGTGAATGCAGAAGAACTACAAAGTCTCAATAGAGGCAGTAGTGGTCTTACATTCATGCAAAACGATGAATTGTTCATCGTGCGTGGAAGGATGCATGGTGAGATTGTTAATAGTTTGCACGAGAATAAGCATGTTATGGAAATTGAACACTATGCTGATCCACAAGCAAACAGTTTCTGGAAAGGTTATACAGATGCGTATGTCGAGAACAGAAACATATCTACCACTCACACAGAGCACACACCAACTATTAATTTAGAGGAGTGTGGCAAGAGAATGGCACTCTTAGAAATCTTATTCCATTCAACTTTTAAAATTACATGCAAAACGTGCAATATTGATGATCTGGAATTATCAGATGATGAATTTGGGGCCAAGTTATACAGTAATCTGCAGCGTATTGAAGAAAGGCAACGCGAATATCTTGCTAAAGATCAAAAACTTTTACGCATGATTCACTTTGTGAAGGACCGGTGTAATCGAAAATTTTCACATTTGCCTTTACTATGGCAAGTGGCAGAAACAGTAGGACATTACACTGATAACCAATCGAAGCAGATAATTGATATCAGTGAGGCGCTCATCAAAGTTAATACCTTAACTCCTGATGATGCAGTGAAGGCCAGTGTAGCATTATTGGAAGTAGCACGATGGTATAAAAATCGGAAGGAATCACTTAAAACAGACACATTGGACTCATTCCGAAACAAAATTTCACCAAAGAGCACGATCAACGCAGCATTGATGTGTGATAACCAGTTAGATAAGAATGCTAATTTCGTATGGGGAAACAGAGAATACCATGCAAAGCGATTCTTCGCTAATTATTTTGAAGCTGTAGACCCAACTGATGCATATGAAAAACACGTCACACGCTTTAACCCCAATGGACAGCGGAAATTATCAATTGGCAAATTAGTAATTCCACTAGATTTCCAGAAGATTAGAGACTCGTTCGTTGGCTTATCGATAAATAAACAACCACTGAGCAAAGCTTGCGTAAGCAAAATTGATGGAGGCTACGTATATCCATGTTGCTGCGTTACAACGGAATTTGGAAAACCAGCATATTCTGAGATAATACCTCCAACAAAAGGACATATCACGATTGGAAATTCAGTGGACCCAAAAATAGTGGATTTACCGAACACAACACCACCAAGTATGTACATTGCAAAAGATGGATATTGTTATATTAATATATTCTTAGCAGCAATGATAAACGTCAATGAGGAATCCGCAAAAGATTACACGAAGTTCCTTAGAGACGAGTTGGTGGAACGGCTTGGCAAATGGCCAAAATTGAAAGATGTAGCAACAGCATGTTATGCTTTATCAGTAATGTTCCCAGAAATCAAGAATGCCGAATTACCACCAATATTAGTTGATCATGAGAGTAAGTCAATGCACGTCATTGATTCATATGGATCACTCAGTGTTGGATTTCACATTCTAAAGGCAAGTACTGTTGGACAACTGATAAAATTTCAGTATGAATCATTGGAAAGTGAGATGCGTGAATACATAGTGGGAGGTACTTTGACACAACAAACTTTCAGCACACTTCTTAAGACTCTCACAAAGAACATGTTTAAGCCAGATAAAATAAAGCAGATAATAGAGGAAGAGCCTTTCCTACTAATGATGGCAATTGCATCCCCAACTGTACTTATCTCGCTGTACAACAACTGCTACATCGAGCAGGCGATGACATATTGGATTGTCAAGAACCAAGGAATCGCAGCAATTTTTGCACAGTTGGAGGCATTAGCAAAGAAAACTTCTCAAGCAGAACTACTAGTTCTTCAAATGCAAATACTTGAAAAAGCTTCTTACCAACTGAGACTTGCAGTCACGGGACTTAATCATGTTGATCCAGCGAAACGACTTCTATGGTCTCACCTAGAAGCCATGACAACACGATCGGAGATGAATAAGGAACTCATAGCGGAAGGTTATGCACTATATGACGAGCGCTTATATACTTTAATGGAAAAAAGTTATGTAGATCAATTAAACCAATCATGGGCAGAATTATCATACTGTGGAAAATTTTCAGCAATATGGCGTGTGTTCAGAGTCAGGAAATACTACAAACCATCTTTAACCGTGAGAAAAAGCGTAGATTTAGGCGCTGTTTACAATATATCAGCTACGCATCTAATATCAGATTTAGTGCAGAGAAGTCGAGATCAAGTCAGCTCTACTTTAACCAAACTCCGCAACGGTTTCTATGACAAAATGGAAAGAGCGAGAGTTAGTGCAGTAAGGACAATATATTGGTTCATACCTGACATATTTAGACTAATCCATATTTTCATAGTTTTAAGTTTTTTAACAACTATAGCTAATACAATAGTCACAACTATGAATGATTACAAAAAGTTGAAAAAGCAACAAAGAGAAGATGAATATGAAGCCGAGATTAATGAGGTGCGAAAAATACACGCCAACCTGATGAAGGAGCATAATGATAATCTGACATGTGATCAATTTATTGAACACATACGCCAGACACATCCACGCCTCATTGAGGCAACATTGGATTTAACACATACAGGTGTCATTCACGAGGGTAAATCCAATTTGGAAACAAACCTCGAACAGGCGATGGCAGTGGGAACTTTACTCACTATGATACTCGATCCACAGAAGAGTGATGCAGTTTATAAGGTTCTCAATAAAATGCGAACAGTGATTAGCACAATCGAACAGAATGTGCCATTTCCATCAGTCAACTTCACAAGCATCTTGACACCTCCTGTAACACAGCAAAGTGTTGATGTTGACGAACCATTAACACTGAGTACCGACAAGAATTTGACTATAGATTTTGACACAAATCAAGACTTGCCAGCGGATACATTTAGCAATGACGTTACATTTGAGAACTGGTGGGCTAATCAGATAAACAACAACAGAACAGTGCCACACTATCGACTTGGGGGAAAGTTTGTAGAATTCACAAGAGAAAATGCAGCAATGGTTAGCATTGAGCTTGCTCACTCGAACATCGAAAAAGAATTTCTACTCAGAGGAGCTGTTGGATCAGGAAAATCCACAGGTTTGCCATATCATCTCAGTATGCGTGGAAAAGTGCTATTGATAGAACCCACTCGACCATTAGCTGAGAACGTTTGTAGGCAACTGCAAGGTCCTCCATTTAATGTGAGCCCCACTCTACAAATGAGAGGTTTGAGCACATTTGGCTGCACTCCTATCACGATAATGACGTCTGGTTTCGCATTGCACATGTATGCTAACAACCCTGATAAGATTTCTGAATATGACTTCATCATCTTTGATGAATGTCACATTATGGAAGCACCTGCAATGGCATTCTATTGTTTGCTTAAGGAGTATGAATACCGAGGCAAGATAATAAAAGTTTCAGCTACACCACCAGGACGAGAATGCGAGTTTTCAACCCAACATCCAGTAGATATACATGTATGTGAAAGCTTGACACAACAGCAATTCGTTATGGAACTAGGAACAGGATCAACTGCTGATGCAACCAAATATGGCAATAACATATTAGTGTATGTTGCAAGTTATAATGACGTAGATTCTTTATCCCATGCTCTAACTGAACTTAAGTATTCAGTAATTAAAGTTGACGGAAGAACAATGAAGCAGAACACCACAGGAATCGTAACAAATGGAACATCCAGTAAGAAATGCTTCGTTGTGGCCACAAATATCATTGAAAACGGTGTAACGCTAGACGTCGATGTCGTCGTCGACTTTGGACTTAAAGTAACAGCTGAATTAGATGTTGATAACAGGGCGATAATGTATAAACGTGTGAGCATATCTTATGGCGAGCGCATTCAGAGACTCGGAAGAGTTGGAAGGAATAAGCCTGGAACAGTTATCCGCATCGGGAAAACAATGAAAGGTTTACAAGAAATTCCAGCGATGATTGCCACTGAAGCAGCTTTCATGTGTTTCGCATATGGACTGAAGGTTATAACACATAATGTATCAACAACACATCTGGCAAAATGCACTGTCAAACAAGCTAGAACCATGATGCAATTTGAACTTTCACCATTTGTAATGGCTGATCTAGTTAAATTTGACGGTTCTATGCACCCACAGATTCATGAAGCATTAACCAAGTATAAATTGAGAGATTCTGTGATCATGTTAAGACCAAATGCAATACCAAAGGTAAATCTTCACAACTGGCTGACGGCTCGTGATTACAACAGGATAGGCTGCTCATTGGAACTCGAAGACCACGTTAAAATACCATATTATATACGGGGAGTTCCTGACAAGCTGTATGGGAAGTTATATGATATTATCCTTCAATACAGCCCTACAAGTTGCTATGGAAGACTATCAAGTGCTTGTGTAGGCAAGGTTGCATATACATTGCGCACTGATCCTTGTTCGTTACCAAGAACAATAGCTATAATCAACGCACTGATTACTGAAGAATACGCAAAGAGGGATCATTACAGAAACATGATAGCGAACCCTTCATCATCGCACGCCTTCTCACTTAATGGGCTAGTATCCATGATCGCTTCTCGGTACATGAAAGACCACACGAAGGAAAATATTGAAAAACTTGTAAGAGTACGCGATCAACTACTTGAGTTCCAAGGCACAGGCATGCAGTTTCAAGATCCTTCAGAATTAATGGACATTGGTGCATTAAACACAGTTATTCACCAAGGAATGGACGCCACGGCTGCTTGTATTGGATTGCAAGGGCGTTGGAATGCTTCACTTATTCAGCGCGATTTGATGATATCAGCAGGGGTCTTCACAGGAGGAATTCTTATGATGTGGTGTCTTTTCACAAAATGGAGTAAAACAGAAGTGTCACACCAAGGAAAGAACAAGCGTAGTCGGCAAAAACTACGATTCAAAGAGGCTCGTGATAACAAATACGCCTACGACGTAATAGGATCAAAGGAAGCGATTGAAGAAAATTTTGGATCCGCATATACTAAGAAAGGAAAAGGTAAAGGGACAAAAGTTGGTTTTGGAGTCAAGCAACACAAATTCCACATGATGTATGGGTTTGATCCTCAAGAGTACAACCTCATTCGTTTTGTCGATCCACTCACAGGAGCCACACTGGATGAACAGATCCATGCCGATATACGTCTAGTGCAAGAACATTTCGATGTTCTTCGGGAGGAAGCAGTAGCAAACGACACGATTGAGAGACAACATATATATAGCAGTCCCGGTTTGCAAGCATTTTTCATACAGAATGGATCAGCAAATGCATTAAGAGTTGATCTAACGCCGCACTCACCCTTACGTGTTGTGACAAACAACAATATAGCAGGTTTCCCAGAGTATGAAGGCACTCTTCGTCAAACAGGAACTGCCATCACTGTACCTGTAAACCAAGTTCCAGTAGCGAATGAAACAGGAGTGGCACACGAATCGAAATCAATGATGATTGGACTAGGTGATTACACACCAATTTCACAACAATTGTGCTTAGTTCAAAATGATTCTGACGGGGTGAAAAGAAACGTATTTTCAATTGGATATGGATCGTACCTTATATCACCAGCGCATTTATTCAAATATAATAATGGTGAAATTACGATTAAGTCATCGAGGGGCTTGTATAAGATAAGAAATTCAGTAGAACTCAAATTACACCCTATTGCACACAGAGATATGGTTATAATTCAACTCCCTAAAGATTTCCCACCATTTCCAATGCGCCTTAAATTTTCAAAACCAACACGGGAATCACGGGTGTGCTTGGTTGGAGTAAATTTTCAGCAAAATTACAGTACCTGCATTGTATCGGAGAGCAGCGTGACAGCACCAAAAGGAAATGGAGATTTTTGGAAACACTGGATATCTACAGTGGATGGACAATGTGGTCTTCCATTAGTAGATGTTAAAAGCAAGCATATAGTCGGAATACATAGTCTTGCATCAACGAGTGGAAATACAAATTTCTTCGTCGCCATACCGGAAAACTTCAATGAGTACATCAGCAATCTTGTGCAAACAAACAAGTGGGAAAAAGGATGGCACTACAATCCAAATCTCATTTCATGGTGTGGTTTAAATTTAGTTGACTCAGCACCTAAAGGCTTATTCAAGACATCAAAACTAGTTGAAGACTTAGACATGAGTGTTGAAGAACAATGCAAGGTAACAGAAACATGGCTCACAGAATGTATCCAGGATAATCTACAAGTTGTGGCAAAATGCCCAGGTCAACTCGTAACTAAACACGTTGTCAAAGGTCCGTGTCCACACTTTCAATTGTACTTATCAACACATGACGAAGCCAAAACATACTTTGCCCCACTACTCGGAAAGTATGATAAGAGCAGATTAAACAGAGCAGCATTCATCAAGGATATATCAAAGTATGCGAAGCCGATTTACGTAGGTGAAATTAACTACGATATCTTTGAAAAGGCAATAGAGCGAGTGATCAAGATTCTTAAAAATGTCGGCATGCAGCAATGCGTTTATGTCACGGATGAAGAAGAAATTTTCAACTCACTCAATCTTAATGCAGCCGTCGGTGCCTTATACACAGGCAAGAAGAAGGATTATTTCAAGGATTATTCAAATGAGGATAAAGCTGAAATCATCATGCGATCCTGTGAGCGGATCTTCAATGGACAACTTGGCATCTGGAATGGGTCACTCAAAGCTGAAATACGCCCAATTGAGAAAACCATGTTGAATAAAACACGCACTTTCACAGCAGCACCATTGGAAACTCTACTTGGTGGAAAGGTTTGCGTGGACGATTTTAATAATCAGTTTTACTCGCATCACCTTGAAGGTCCATGGACAGTTGGAATCACAAAATTTTATGGAGGATGGAATCGTTTACTCGAGAAGTTACCAGAAGGCTGGATTTATTGCGACGCCGATGGTTCACAATTTGATAGCTCATTAACGCCATACCTTATCAACGCCGTTTTACATATTCGACTGCAATTTATGGAAGAATGGGCATTAGGGGCACAAATGTTGCAAAATCTGTACACCGAAATCGTCTACACACCAATTGCAACGCCAGACGGATCAGTCATTAAGAAATTCAAAGGAAACAACAGTGGCCAACCCTCTACAGTTGTTGATAACACACTCATGGTCATATTAGCATTCAATTACGCAATGTTATCGAGTGGTATCAAAGAAGATGAAATAGACAATTGCTGCCGAATGTTCGCCAATGGAGACGATTTATTGCTGGCAGTGCACCCGGACTTTGAACATATACTGGATGGATTTCAAAATCACTTTGGAAATTTAGGCCTCAATTTTGAGTTTACATCACGAACAAAAGACAAATCAGAACTGTGGTTCATGTCCACACGAGGTATCAAATGTGAAGGCATCTATATACCAAAACTTGAGAAAGAAAGAATAGTTGCCATACTCGAATGGGATCGGTCAAACCTACCTGAGCACCGTCTCGAAGCTATATGCGCAGCCATGGTAGAAGCATGGGGATATCCAGACCTTGTTCAAGAAATACGAAAATTCTATGCGTGGCTTCTGGAAATGGAACCATTTGCAAATCTAGCGAAAGAGGGCTTAGCACCATATATAGCAGAAACCGCACTCAGAAATTTGTACTTGGGCACAGGGATCAAGGAAGAGGAGATTGAAAAATACTTTAAACAGTTTGCTAAAGATCTCTCTGGCTATATAGAAGACTATAATGAAGATGTTTTTTATCAATCCGGTTCTGTGGATGCAGGAGCCCAAGGAGGAAGAAGTGGCTCAGGAACAACCCCACCTGCAGCG---------------------------------------------------GGTAGTGGAAC----AGGAACTAGACCACCTTCAACTGGATCAGCAGCACAAGGAAACACACCTCCAG---CATCAGGTGGATCATCAGGAAACAATGGAGGC---GGTCAATCAGGTTC---AAACGGCACTGG------------AGGTCA---AGCAGGCTCAAGCGGA------------ACAGGGGGCCAAAGAGACAAAGACGTTGATGCTGGTTCAACAGGAAAAATATCAGTGCCAAAGCTTAAGGCCATGTCGAAGAAAATGCGCCTGCCAAAGGCAAAAGGAAAAGACGTTTTACACTTAGATTTCCTGTTAACATATAAACCCCAACAGCAAGACATATCAAACACTAGAGCAACTAAGGAAGAGTTTGATAGATGGTACGATGCCATAAAGAAGGAGTACGAAATTGATGATACACAAATGACAGTTGTCATGAGTGGTCTCATGGTATGGTGCATCGAAAACGGTTGCTCACCAAACATAAACGGAAATTGGACGATGATGGACGGAGACGAACAAAGGGTTTTTCCATTAAAGCCAGTCATTGAAAACGCATCTCCAACTTTCCGACAAATAATGCATCATTTTAGTGATGCAGCTGAAGCGTATATAGAGTACCGAAACTCTACAGAGCGATACATGCCAAGATACGGTCTTCAGCGAAATCTCACCGACTATAGCTTAGCGCGGTATGCTTTTGATTTCTATGAAATGACTTCGCGCACACCAGCTAGAGCTAAGGAAGCCCACATGCAG---ATGAAAGCCGCAGCAGTTCGTGGTTCAAACACACGTCTGTTCGGTCTGGACGGAAATGTCGGCGAGACTCAGGAGAATACAGAGAGACACACAGCTGGCGACGTTAGTCGCAACATGCACTCTCTGTTGGGAGTGCAGCAGCACCAC--------------------------------------------------------------------------------------------------------------------------------------------------------------------------------------------------------------------------------------------------------------------------------------------

>KT895081

--------------------AAAAACAACAAGACTCAACACAACACAACAAAACACAATCAAGCAAATCCAATTTACTTGCGCTCAGATTGTAGTGAACGGCTCGAACGAAACGGTTCTTCGAGATCACTCTCTGATTTTCTCTCA----------TCTTTCAACTTCTTTCGAAAGAAATGGCGGGAATGTGGACTCATGTGACGTACAAGTGGCAACCAGATGTCAACAACGATCGTCACATTAAGAGAGTAATGGAAATGTTTGCAGCAAAACATCAACATTACTCAGAAGAACAGCGACTTGCTCATAATATGAAATTATTAAGGAAGACAAGTGTCGTAGGTGTTGAGCCTGCGAAATTAAAGCAGAAACAAACAACTCAACAAATGTGGGTTGAGAAGTGTGATCATAATCCTGTTGATCACTTAGTATATCCACGACTTGAAAGACCTTTGAACAAAGTG---GATATGAGCATTAAAAGTGCATCTGTCAGCAAGCTAACCAGAGAGATTTTAGAGATCTCAAAGGTAAGCGGTCTCAAAATTGAACTGATTGATAAACGGAAAAGATTTAAAACACAGTTATCAATTAAAAAGTTCAATGGTAAAAACTTCCTCCACTGCAAAACGAATCATGAAAACAATCTATTCAAGAGGAGGGACATAGCCATTGGATACAAATGGCTTCCGACAATTGAAGCTATTGCTCGATGCTATAGCACAATGAGTCAAGAGGAATTGCAAAGCCTTTACAGAGGAAGCAGTGGCCTCACATTCATCCAGAATGATGAATTGTTCATTGTCAGAGGGAGAATGAATGGTGAACTGATCAATAGCTTGCATGAAACAAATCGGGTTATGGACATTGAACACTATGCAGATCCCCAAGCTAATGATTTCTGGAGGGGATACACAGATGCTTACGTAGAAAATCGCAACATATCGACCACTCATACAGAGCACACCCCTACAATCAATCTAGAAGAATGTGGAAAACGAATGGCTCTACTTGAAATACTATTCCACTCTACATTCAAGATCACATGCAAAACATGTAACATTGATGATCTTGAATTGTCGGATGATGAATTTGGAGCCAAACTCTACAAGAATTTGCAACGCATTGAAGGAAAACAACGAGAGTACCTTGCCAAGGATCAAAAACTATCTAGGATGATACAATTTATCAAAGAAAGGTGTAATCCAAAATTCTCACATTTACCTATGTTATGGCAAGTTGCAGGAACAATAGGACACTACACTGATAATCAGTCAAAGCAAATAATGGATATTAGTGAAGCGCTCATTAAAGTCAATACTTTGACCCCTGATGACGCTATGAAAGCAAGTGCAGCACTACTTGAAGTGTCGCGATGGTATAAGAATCGTAAGGAGTCACTCAAAACTGACTCGTTGGAGTCTTTTAGAAATAAAATATCACCAAAGAGCACAATCAACACAGCACTGATGTGCGATAATCAATTGGACAAGAATGCCAATTTCGTCTGGGGAAACAGGGAGTATCATGCGAAGCGTTTCTTTGCCAACTATTTTGAGGCTGTAGACCCCACCGATGCTTACGAGAAGCACGTAACACGTTTTAATCCAAATGGTCAACGCAAGTTATCGATTGGGAAACTAGTGATCCCGCTAGATTTCCAGAAGATAAGGAATTCATTTGTGGGCCTACCGATTAACAAACAACCGTTGAGCAAAGCGTGCATTAGCAAAATCGACGGAGGGTATGTGTATCCATGCTGCTGTGTCACTACCGAATTTGGCAAACCAGCGTATTCTGAAATAATACCTCCAACTAAAGGACATATAACAATCGGAAATTCTGTTGACCCAAAAATAGTAGACTTGCCCAATACAACGCCACCAAGCATGTACATTGCACGAGACGGATTTTGTTACATCAATATTTTCCTCGCAGCAATGATCAACGTAAACGAAGATTCTGCAAAGGATTATACAAAATTTTTAAGGGATGAGTTAGTGGAACGTCTTGGAAAGTGGCCAAAACTCAAGGATGTTGCAACAGCTTGTTATGCATTGTCCGTGATGTTTCCGGAAATCAAAAATGCTGAATTACCGCCAATCTTAGTGGACCATGAGAACAAATCAATGCACGTAATTGATTCATATGGCTCACTGAGTGTTGGCTTTCACATCCTAAAAGCAAGTACAATAGGTCAGCTTATCAAATTTCAATATGAATCAATGGAAAGCGAAATGAGGGACTACTTAGTAGGAGGCACCCTGACACAACAAACGTTCAACACCCTTCTTAAGTCTCTCACAAAGAACATGTTCAAGCCAGACCGCATTAAGCAAATAATTGAAGAGGAACCTTTCCTACTAATGATGGCAGTTGCATCGCCAACGGTTTTGATATCATTGTACAACAATTGCTACATTGAGCAAGCCATGACGTATTGGATTGTTAAGAACCAAGGCATTGCAGCGATATTTGCTCAATTGGAAGCACTAGCTAAGAAGACATCTCAAGCGGAGTTATTAGTTCTACAAATGCAAATCCTCGAAAAAGCTTCCAATCAGCTGAGACTTGCTGTCACTGGACTTAATCATGTAGATCCAGCAAAGCGACTTCTCTGGTCCCACCTTGAAGCCATGACAACACGTTCTGAAATGAACAAAGAGCTGATAGCTGAGGGATATGCTTTATATGACGAACGCTTATATACTCTAATGGAAAAAAGTTATGTAGATCAATTAAACCAGTCATGGGCAGAATTATCATATTATGGAAAATTTTCAGCAATATGGCGTGTGTACAAAGTCAAGAAATACTACAAACCGTTCTTAACCGTGAGAAAAAGCGTAGATTTAGGCGCTGTTTACAATATATCAGCTACGCATCTAATATCAGATTTAGTGCAGAAAAGTCGCGATCGAGCCAGTTATACTTTAACCAAACTCCGCCACAGTTTTTGCGATAATTTAGAACGAGCACGCGTCAAGACAATTAAAACAATCTACTGGTTTATACCCGACGTATTTAGACTAATGCATATATTTATAATTCTTAGTTTGTTAACCACAATAGCAAACACTATAATCGTCACTATGAATGATTACAAAAAGTTGAAGAAACAACAAAGAGAGGATGAATATGAATCTGAAATTAATGAAGTTCGGAAAATTCACGCTAGTTTAATGAAGGAACACAATGATAATCTTACGTGCGAGCAATTTATTGAATATATACGCCAAACACACCCAAGATTGGAAGAGGCCACATTGGACTTAACACACACAGGTGTGATACATGAAGGCAAGTCCAATCTGGAAACTAATCTTGAACAAGCTATGGCAGTCGGAACTTTGATTACTATGATATTCGATCCACATAAGAGCGATGCTGTGTACAAAGTCTTAAACAAGATGAGAACAGTAATAAGCACAATAGAGCAGAACGTGCCATTCCCTTCAGTTAATTTCACAAATCTACTAACACCACCTGTAGTGCAGCAAAGCGTAGATGTTGATGAGCCATTAACTCTAAGCACTGAGAAGAATCTAACAATAGATTTTGACACAAATCAAGATTTACCTGCAGATACATTCAGCAACGATGTGACATTTGAGGACTGGTGGACAAACCAAATAAGCAATAATAGAACAGTCCCCCACTATCGACTTGGTGGCAAATTTGTAGAATTCACGAGAGAGAATGCAGCACGCGTTAGCATAGAACTTGCACATGAGAATATTGAGAAAGAATTCTTACTTAGAGGAGCTGTTGGTTCAGGAAAATCTACTGGACTACCATATCATTTAAGCATGCGAGGCAAAGTTCTGCTACTCGAACCAACACGACCACTAGCTGAGAACGTGTGTAGGCAATTGCAAGGGCCACCATTCAATGTTAGTCCAACACTGCAAATGCGAGGACTAAGCTCATTTGGTTGCACTCCGATCACAATAATGACCTCTGGTTTCGCACTGCATATGTATGCAAACAACCCCGACAAGATTTCTGATTATGACTTTGTCATCTTTGACGAATGTCACATAATGGAAGCACCAGCCATGGCATTTTATTGTTTACTCAAGGAGTACGAATATCGAGGAAAGATCATAAAAGTTTCAGCAACGCCTCCTGGTAGAGAGTGTGAATTCTCAACACAACACCCAGTGGACATTCACGTTTGCGAAAGTTTGACCCAACAACAATTTGTGCTGGAGTTGGGCACCGGGTCAAATGCTGATGCAACCAAATATGGGAACAACATACTAGTATATGTTGCAAGCTACAATGACGTCGACTCACTTTCACATGCACTCACTGAGTTGCACTATTCTGTCATCAAAGTTGATGGGAGAACAATGAAGCAAAACACCACAGGGATTGTGACAAACGGCACATCACAAAAGAAGTGCTTTATTGTTGCAACGAACATTATTGAAAATGGTGTCACATTGGACGTTGACGTCGTCGTTGATTTTGGACTCAAAGTCACAGCTGAACTTGACGTAGATAATAGAGCAATTCTTTACAAACGTTCAAGTATCTCGTATGGTGAGCGGATTCAACGATTGGGCCGTGTTGGTAGAAATAAACCTGGAACAGTTATCAGAATTGGTAAAACAATGAAAGGTCTACAGGAGATTCCGGCTATGATTGCAACTGAGGCAGCATTTATGTGTTTCGCATACGGTTTAAAGGTTATAACACATAATGTCTCCACGACACACTTAGCGAAGTGCACAGTGAAGCAGGCAAGAACAATGATGCAGTTTGAACTATCACCATTCGTTATGTCAGAACTAGTCAAATTTGATGGTTCAATGCATCCGCAGATTCATGAAGCTCTCGTTCGGTACAAGCTTCGGGATTCCGTAATCATGCTAAGACCAAATGCAATTCCAAAAGTGAACTTTCACAATTGGTTGACAGCTCGTGATTATAACAGAATGGGTTGTTCGTTGGAGCTTGAAGATCATGTCAAAATACCATATTATATACGAGGAGTACCTGACAAGCTCTACGGCAAATTGTATGACATTATCTTGCAATATAGCCCCACAAACTGTTATGGAAGACTATCGAGCGCGTGCGCTGGGAAGGTTGCATACACATTGCGAACCGATCCTTGTTCACTTCCGCGAACAATAGCAATCATCAACACCTTGATCACAGAAGAATATGCCAAAAGAGATCATTATCGTAATATGATTGCTAATCCTTCGTCGTCGCATGCATTCTCTTTAAATGGAATAGTCTCTATGATTGCTTCAAGGTACATGAAAGATCACACGAAGGAAAACATAGAGAAATTGGTGAAAGTGCGCGATCAGTTATTGGAATTCCAAAGTACAGGTATGCAATTTCAGGACCCATCTGATCTTATGGAAATAGGAGCGTTGAATACAGTCATTCACCAAGGAGTGGACGCAACAGCTGCGTGCATTGGACTACAAGGAAGATGGAATGCTCCTTTAATACGACGCGATCTAATGATTGCAGGAGGTGTGTTCGTTGGAGGAATTCTCATGATGTGGAGTTTGTTTACTCGATGGAGTGGCACAAATGTGTCGCACCAAGGAAAGAACAAGCGCAGCAGACAGAAGTTACGATTCAAAGCAGCCCGAGACACGAAATACGCATACGATGTGACAGGGTCGGAAGAAGCGCTTGGTGAGAATTTTGGGACTGCATACACCAAGAAAGGCAAAGGTAAAGGCACAAAAGTCGGAATGGGGGTAAAGCAACATAAATTTCATATGATGTACGGGTTTGATCCTCAAGAATACAATTTGATCCGTTTTGTGGATCCGCTTACAGGTGCAACTTTAGATGAACAAATTCACGCAGACATACGCTTGATTCAAGAGCACTTTGATGATATCCGGGAGGAAGCGATATTAAATGATGTGATCGAACGACAACACATTTACGGGAACCCTGGATTACAAGCGTTTTTCATACAGAATGGGTCATCAAATGCTCAGAGAGTTGATCTAACACCGCATTCACCCACACGTGTTGTTGGAAATAATAATATAGCAGGTTTCCCAGAATATGAAGGCACACTTCGTCAAACAGGAACGGCTATAACTGTGCCAATTAATCAGGTTCCAATGGCAAACGAGACAGGTGTCGCGCACGAATCTAAATCAATGATGACAGGGTTAGGTGACTATACACCCATTTCTCAGCAATTGTGTTTAGTCCAGAACGATTCCGATGGAGTGAAAAGAAACGTTTATTCAATCGGATACGGATCATATCTTATTTCACCAGCCCATCTGTTCAAATATAACAATGGTGAAATAACGATTAAGTCCTCAAGAGGCCTCTACAAAATCAGGAATTCAGTTGATCTCAAGTTACATCCCATTGCACACAGAGATATGGTCATAATTCAATTACCCAAGGACTTTCCACCATTTCCAATGCGCTTGAAATTTGCAACTCCATCACGAGATATGCGGGTTTGTTTAGTTGGAATAAATTTTCAGCAAAATCATAGTTCGGGTATAAATTCAAAGAGCAGTGTGACAGCACCAAAAGGAAATGGTGATTTTTGGAAACATTGGATCTCAACAGTTGATGGACAATGTGGACTACCGTTAGTGGATGTAAAGAATAAGCATATAGTTGGAATACATAGTCTTGCATCAACAAACGGGAACACCAATTTCTTCGTTGCAATGCCTGATAATTTCAATGAATACATCCATGGACTCGTGCAAACGAATAAATGGGAAAGAGGCTGGCACTATAATCCTAATCTTATTTCTTGGTGTGGACTAAATTTGGTCGATTCAGCTCCTAAAGGCTTGCTTAAGACCTCAAAATTAGTGGAAGATTTAGACATGGCTGTTGAGGAGCAATGTAAAATCACTGAGACTTGGCTCACAAAATGTATACAAGATAATCTCCAGGTCGTAGCAAAATGTCCTGGCCAGCTTGTGACCAAGCATGTCGTTAAAGGTCCATGTCCGCATTTCCAACTGTACCTGTCAACACATGATGAAGCTAAAGCATATTTTACTCCTTTACTTGGGAAATATGATAAGAGCAGACTAAACAGAGCAGCCTTCATTAAAGATATCTCGAAATATGCAAAACCAATTTATATTGGCGAAATAAATTATGATGTCTTCGAAAAAGCCATTGAACGGGTGATTCGCATTCTCGGAGACGTAGGAATGCAACAATGCACGTACGTCACAGATGAGGAGGAAATTTTCAAATCACTCAATCTTAATGCAGCAGTTGGAGCACTATACACAGGGAAGAAAAAGGATTACTTCGCAGATTTTTCGGAAGAAGACAAAGCAGAAATAGTTATGAGATCCTGTGAACGCTTATATAATGGACAACTGGGCATTTGGAATGGATCGCTCAAAGCCGAGATACGCCCTATAGAGAAGACCATGCTGAACAAAACTCGCACATTTACAGCAGCGCCATTGGAAACTTTACTTGGTGGAAAAGTGTGTGTTGACGATTTCAACAACCAATTCTATTCACATCATCTAGAGGGTCCCTGGACGGTTGGAATTACGAAATTCTATGGGGGATGGAACCGATTGCTCGAGAAGTTGCCAGAAGGATGGATTTACTGTGATGCTGATGGCTCACAATTTGACAGCTCCCTAACACCATATCTTATAAATGCCGTGCTACACATTCGACTACAATTTATGGAGGAGTGGGAACTAGGCGCACAAATGTTACGAAATCTGTACACTGAAATCGTCTACACACCAATTGCAACACCAGACGGTTCCATAATCAAGAAATTTAAAGGAAATAACAGTGGTCAGCCATCAACTGTAGTTGATAACACGCTAATGGTCATCTTAGCATTCAATTACGCAATGTTGTCAAGTGGAATACAAGACAATGAAATCGACAACTGTTGCAGAATGTTTGCTAATGGAGATGATCTATTATTGGCAGTACATCCCGATTATGAACATATACTGGACGGATTTCAAGATCACTTCGGAAATCTCGGATTAAATTTTGAGTTTACATCACGAACACGAGATAAGTCCGAACTGTGGTTCATGTCTACAAGAGGAATCAAATGTGAAGGAATCTACATACCTAAACTTGAGAAAGAAAGAATAGTCGCAATACTCGAATGGGATCGATCAAATCTACCTGAGCATAGATTGGAAGCCATTTGTGCAGCTATGGTTGAAGCATGGGGCTACTCAGATCTTGTTTACGAAATTCGAAAATTTTATGCGTGGCTTCTGGAAATGCAACCATTTGCAAATCTAGCCAAAGAGGGTTTAGCTCCATATATTGCAGAAACAGCGCTTCGTAACTTGTATCTGGGTTCAGGCATCAAGGAAGAGGAAATCGAGAAATACTTCAAGCAGTTTGCAAAGGACCTCCCTGGGTATTTAGAGGATTACAACGAAGAAGTTTTTCACCAAGCTGGAACAGTCGATGCAGGCGCACAAGGAGGAGATGGGAAAGCCGGGACTCAGCCGCCAGCCACAGG-------------------------------------------------AGCAGCAGCTCAAGGAGGAGCTCAACCACCAGCAACTGGAGCAGCCGCGCAA---------CCACCCG---CGACTCAAGGTTCACAACCGCCCACAGGGGG----AGCTACTAG-----TGGAGGTGGTGCACA---------------------AACGGGAGCTGGTGAAACTGGCTCAGTTACAGGAGGCCAAAAAGACAAGGATGTAGATGCTGGTACGACAGGCAAAATCACAGTGCCAAAACTTAAAGCCATGTCGAAGAAGATGCGCTTGCCAAAAGCAAAAGGAAAAGATGTTTTGCATCTGGACTTTCTGTTAACATACAAACCGCAACAACAAGACATATCAAACACAAGAGCAACCAGAGAGGAGTTTGATAGGTGGTATGAAGCCATAAAGAAGGAATATGAAATAGATGACACACAAATGACAGTTGTCATGAGTGGTCTAATGGTTTGGTGTATTGAGAATGGTTGCTCACCAAACATAAACGGAAATTGGACGATGATGGATGGAGATGAACAAAGGGTTTTCCCATTAAAGCCAGTCATTGAAAACGCATCTCCAACATTCCGACAAATAATGCATCATTTCAGTGATGCAGCTGAAGCATATATCGAGTATAGAAACTCTACAGAGCGATACATGCCACGATATGGACTTCAGCGGAATCTCACCGACTATAGCTTAGCGCGGTATGCCTTCGACTTCTACGAAATGAATTCAAGGACACCAGCTAGAGCTAAGGAAGCCCACATGCAG---ATGAAGGCCGCAGCAGTTCGTGGTTCAAACACACGATTGTTCGGTCTGGACGGAAATGTCGGCGAGACCCAGGAGAATACAGAGAGACACACAGCTGGCGATGTCAGTCGCAACATGCACTCTCTGTTGGGAGTGCAGCAGCACCACTAGTCTCCTGGAAACCCTGTTTGCAGTACCTATAGTATGTACTA--------ATAATGTATGTCAGTGAGGTTTTACCTC-----------------------GTTTTTACTA---TATTACGTATATACTTAAAGCGTGAACCAGTCTGCAGGACACAGGGTTGGACCCAGTGTCTTCTGGTGTAGCGTGTACTAGCGTCGAGCCACGTGACGGACAGCACTGGGTGTGGCTTTGCCATTGGTGCTGCGAGTCTCTTGGTGAGAGAC----------------

>KT895080

--------------------AAAAACAACAAGACTCAACACAACACAACAAAACACAACCAAGCAAATCCAATTTACTTGCGCTCAGATTGTAGTGAACGGCTCGAACGAAACGGTTCTTCGAGATCACTCTCTGATTCTTCCTCA----------TCTTTCAACTTCTTTCGAAAGAGATGGCGGGAATGTGGACTCATGTAACGTACAAGTGGCAACCAGATGTCAACAACGATCGTCACATTAAGAGAGTAATGGAAATGTTTGCAGCAAAACATCAACATTACTCAGAAGAACAGCGACTTGCTCATAACATGAAATTATTAAGGAAGACAAGTGTCGTATGCGTTGAGCCTGCGAAATCAAAGCAGAAGCAAACAACTCAACAAATGTGGGTTGAGAAGTGTGATCATAATCCTGTTGATCACTTAGTATATCCACGGTTTGAAAGACCTTTGAACAAAGTG---GATATGAGCATTAAAAGTGCATCTGTCAGCAAGCTAACCAGAGAGATTTTAGAAATCTCAAAGGTAAGCGGTCTCAAAATTGAACTAATTGATAAACGGAGAAGATTTAAAACACAGTTATCAATTAAAAAGTTCAATGGTAAAAACTTCCTCCACTGCAAAACGAATCATGAAAACAATCTATTTAAGAGGAGGGACATAGCCATTGGACACAAATGGCTTCCGACAATTGAAGCTATTGCTCGATGCTATAGCACAATGAGTCAAGAGGAATTGCAAAACCTTTACAGAGGAAGCAGTGGCCTCACATTCATCCAGAATGATGAATTGTTCATTGTCAGAGGGAGAATGAATGGTGAACTGATCAATAGCTTGCATGAAACAAATCGGGTTATGGACATTGAACACTATGCAGATCCCCAAGCTAATGATTTCTGGAGGGGATACACAGATGCTTACGTAGAAAATCGCAACATATCGACCACTCATACAGAGCATACCCCTACAATCAATCTAGAAGAATGTGGAAAACGAATGGCTCTACTTGAGATACTATTTCACTCTACATTCAAGATCACATGCAAAACATGTAATATTGATGATCTTGAATTGTCGGATGATGAATTTGGAGCCAAACTCTACAAGAATTTGCAACGCATTGAAGAAAAACAACGAGAGTACCTTGCCAAGGATCAAAAACTATCTAGGATGATACAATTTATCAAAGAAAGGTGTAATCCAAAATTCTCACATTTACCTATGTTATGGCAAGTTGCAGAAACAATAGGACACTACACTGATAATCAGTCAAAGCAAATAATGGATATTAGTGAAGCGCTCATTAAAGTCAATACTTTGACCCCTGATGACGCTATGAAAGCAAGTGCAGCACTACTTGAAGTGTCGCGATGGTATAAGAATCGTAAGGAGTCACTCAAAACTGACTCGTTGGAGTCTTTTAGAAATAAAATATCACCAAAGAGCACAATCAACACAGCACTGATGTGCGATAATCAATTGGACAAGAATGCCAATTTCGTCTGGGGAAACAGGGAGTATCATGCGAAGCGTTTCTTTGCCAACTATTTTGAGGCTGTAGACCCCACCGATGCTTACGAGAAGCACGTAACACGTTTTAATCCAAATGGTCAACGCAAGTTATCGATTGGGAAACTAGTGATACCGCTAGATTTCCAGAAGATAAGAGACTCATTTGTGGGCCTACCGATTAACAAACAACCGTTGGGCAAAGCGTGCATTAGCAAAATCGACGGAGGGTATGTGTATCCATGCTGCTGTGTCACTACTGAATTTGGCAAACCAGCGTATTCTGAAATAATACCTCCAACTAAGGGACATATAACAATCGGAAATTCTGTTGACCCAAAAATAGTAGACTTGCCCAACACAACGCCACCAAGCATGTACATTGCACGAAACGGATTTTGTTACATCAATATTTTCCTCGCAGCAATGATCAACGTAAACGAAGATTCTGCAAAGGATTATACAAAATTTTTAAGGGATGAGTTAGTAGAACGTCTTGGAAAGTGGCCAAAACTCAAGGATGTTGCAACAGCTTGTTATGCATTGTCCGTGATGTTTCCGGAAATCAAAAATGCTGAATTACCGCCAATCTTAGTGGACCATGAGAACAAATCAATGCACGTAATTGATTCATATGGCTCGCTGAGTGTTGGCTTTCACATCCTAAAAGCAAGTACAATAGGTCAGCTTATCAAATTTCAATATGAATCAATGGAAAGCGAAATGAGAGACTACTTAGTAGGAGGCACCCTGACACAACAAACGTTCAACACCCTTCTTAAGTCTCTCACAAAGAACATGTTCAAGCCAGACCGCATTAAGCAAATAATTGAAGAGGAACCTTTCCTACTAATGATGGCAGTTGTATCGCCAACGGTTTTGATATCATTGTACAACAATTGTTACATTGAGCAAGCCATGACGTATTGGATTGTTAAGAACCAAGGCATTGCAGCGATATTTGCTCAATTGGAAGCATTAGCTAAGAAGACATCTCAAGCGGAGTTATTAGTTCTACAGATGCAAATCCTCGAAAAGGCTTCCAATCAGCTGAGACTTGCTGTCACAGGACTTAATCATGTAGATCCAGCAAAACGACTTCTCTGGTCTCATCTTGAAGCCATGACGACACGTTCTGAAATGAACAAAGAGCTGATAGCTGAGGGATATGCTTTATATGACGAACGCTTATATACTCTAATGGAAAAAAGTTATGTAGATCAATTAAACCAGTCATGGGCAGAATTATCATATTATGGAAAATTTTCAGCAATATGGCGTGTGTACAAAGTCAAGAAATACTACAAACCGTTCTTAACCGTGAGAAAAAGCGTAGATTTAGGCGCTGTTTACAATATATCAGCTACGCATCTAATATCAGATTTAGTGCAGAAAAGTCGCGATCGAGCCAGTTATACTTTAACCAAACTCCGCCACAGTTTTTGCGATAATTTAGAACGAGCACGCGTCAAGACAATTAAAACAATCTACTGGTTTATACCCGACGTATTTAGACTAATGCATATATTTATAGTTCTTAGTTTGTTAACCACAATAGCAAACACTATAATCGTCACTATGAATGATTACAAAAAGTTGAAGAAACAACAAAGAGAGGATGAATATGAATCTGAAATTAATGAAGTTCGAAAAATTCACGCTAGTTTAATGAAGGAACACAATGACAATCTTACGTGCGAGCAATTTATTGAATATATACGCCAAACACACCCAAGATTGGAAGAGGCCACATTGGACTTAACACACACAGGTGTGATACATGAAGGCAAGTCCAATCTGGAAACTAATCTTGAACAAGCTATGGCAGTCGGAACTTTGATTACTATGATATTCGATCCACATAAGAGTGACGCTGTGTATAAAGTCTTGAACAAGATGAGAACAGTAATAAGCACAATAGAGCAGAACGTGCCATTCCCTTCAGTTAATTTCACAAATCTACTAACACCACCTGTAGTGCAGCAAAGCGTAGATGTTGACGAGCCATTAACTCTAAGCACTGAGAAGAATCTAACAATAGATTTTGACACAAATCAAGATTTACCTGCAGATACATTCAGCAACGATGTGACATTTGAGGACTGGTGGACAAACCAAATAAGCAATAATAGAACAGTTCCCCACTATCGACTTGGTGGCAAGTTTGTAGAATTCACGAGAGAAAATGCAGCACGCGTTAGCATAGAACTTGCACATGAGAATATTGAGAAAGAATTCTTACTTAGAGGAGCTGTTGGTTCAGGAAAATCTACTGGACTACCATATCATTTAAGCATGCGAGGCAAAGTCCTGCTACTCGAACCAACACGACCACTAGCTGAAAACGTGTGTAGGCAATTGCAAGGGCCACCATTCAATGTTAGTCCAACATTGCAGATGCGAGGACTAAGTTCATTTGGTTGTACTCCGATTACAATAATGACCTCCGGTTTCGCACTGCATATGTATGCAAACAACCCTGACAAGATCTCTGATTATGACTTTGTCATCTTTGATGAATGTCACATAATGGAAGCACCAGCCATGGCATTCTATTGTTTACTCAAGGAATATGAGTATCGGGGAAAGATCATAAAAGTTTCAGCAACGCCTCCTGGTAGAGAGTGTGAATTCTCAACACAACATCCAGTGGACATTCACGTTTGCGAAAGCTTAACCCAACAACAATTTGTGCTGGAGTTAGGCACTGGGTCCAATGCTGATGCAACCAAATATGGGAACAACATACTAGTATATGTTGCAAGCTACAATGATGTCGACTCGCTTTCACATGCACTCACTGAGTTGCACTATTCTGTCATCAAAGTTGACGGAAGAACAATGAAGCAAAACACCACAGGGATTGTGACAAACGGCACATCACAGAAGAAGTGCTTTATTGTTGCAACGAACATTATTGAAAATGGTGTTACATTGGACGTTGACGTCGTCGTTGATTTTGGACTCAAAGTCACAGCTGAACTTGACGTTGATAATAGAGCAATTCTTTACAAACGTCCAAGTATTTCGTATGGTGAGCGGATTCAACGATTGGGCCGTGTTGGTAGAAATAAACCTGGAACAGTTATCAGAATTGGTAAAACAATGAAAGGTCTACAGGAGATTCCGGCTATGATTGCAACTGAGGCAGCATTTATGTGTTTCGCATACGGTTTAAAGGTTATAACACATAATGTCTCTACGACACACTTAGCGAAGTGCACAGTGAAGCAGGCAAGGACAATGATGCAGTTTGAACTATCACCATTCGTTATGTCAGAACTAGTCAAATTTGATGGTTCAATGCATCCGCAGATTCATGAAGCTCTCGTTCGATACAAGCTTCGGGATTCCGTAATCATGCTAAGACCAAATGCAATTCCAAAAGTGAACTTTCACAATTGGTTGACAGCTCGTGATTATAACAGAATGGGTTGTTCGTTGGAGCTTGAAGATCATGTCAAAATACCATATTATATACGAGGAGTACCCGACAAGCTCTACGGCAAATTGTATGACATTATCTTGCAATATAGCCCCACAAACTGTTATGGAAGACTATCGAGCGCGTGCGCTGGGAAAGTTGCATACACATTGCGAACCGATCCTTGTTCACTTCCGCGAACAATAGCAATCATCAACACCTTGATCACAGAAGAATATGCCAAAAGAGATCATTATCGGAATATGATTGCTAATCCTTCGTCGTCGCATGCATTCTCTTTAAATGGTATAGTCTCTATGATTGCTTCAAGGTACATGAAAGACCACACAAAGGAAAACATAGAGAAATTGGTCAAAGTGCGCGATCAGTTATTGGAATTCCAAGGTACAGGTATGCAATTTCAGGACCCATCCGATCTTATGGATATAGGAGCGTTAAATACAGTCATTCACCAAGGAATGGATGCAACAGCTGCGTGTATTGGACTGCAAGGAAGATGGAACGCTCCTTTAATACAACGTGATTTAATGATTGCAGGAGGTGTGTTCATCGGAGGTATTCTCATGATGTGGAGTTTGTTCACTCGATGGAGTAGCACAAACGTGTCGCACCAGGGAAAGAACAAGCGCAGCAGACAGAAGTTACGATTCAAAGCAGCCCGAGACACGAAATACGCATACGATGTGACAGGGTCGGAAGAAGCGCTTGGTGAGAATTTTGGAACTGCATACACCAAGAAAGGCAAAGGTAAGGGCACAAAAGTTGGAATGGGGGTAAAGCAACACAAATTTCATATGATGTACGGGTTTGATCCTCAAGAGTACAATTTGATTCGTTTTGTGGACCCACTTACAGGTGCAACTTTGGATGAACAAATTCACGCAGACATACGCTTGATTCAAGAGCACTTTGATGATATCCGGGAAGAAGCAGTGTTAAATGATGTGATCGAACGACAACACATCTACGGAAACCCTGGATTACAAGCATTTTTCATACAGAATGGGTCATCAAATGCTCTAAGGGTTGATCTAACACCGCATTCACCCACACGTGTTGTTGGAAATAATAATATAGCAGGTTTCCCTGAATATGAAGGCACACTTCGTCAAACAGGAACAGCTATAACTGTGCCTATTAATCAGGTTCCAGTGGCAAATGAGACAGGTGTCGCGCATGAATCTAAATCAATGATGACAGGATTAGGTGACTACACACCCATTTCTCAGCAATTGTGTTTAGTCCAGAATGATTCTGATGGAGTGAAAAGGAACGTTTATTCAATTGGATATGGATCATATCTTATTTCACCAGCCCATCTATTCAAATACAACAATGGTGAAATTACAATTAAATCCTCAAGAGGTCTCTACAAAATTAGAAATTCAGTTGATCTCAAGTTACATCCCATTGCACACAGAGATATGGTCATAATCCAATTCCCCAAGGACTTCCCACCCTTTCCAATGCGTTTGAAATTTGCAACTCCATCCCGAGATATGGGAGTTTGTTTAGTTGGAATAAATTTTCAGCAAAATCATAGTTCGTGTATAATATCAGAGAGCAGTGTGACAGCACCAAAAGGAAATGGCGATTTTTGGAAACATTGGATTTCAACAGTTGATGGGCAATGTGGACTACCATTAGTGGATGTGAAGAATAAGCATATAGTTGGAATACACAGTCTCGCATCGACAAATGGGAACACCAATTTCTTTGTTGCAATGCCTGACAATTTCAATGAATACATCCATGAACTCGTACAAACGAATAAATGGGAAAGGGGCTGGCACTACAATCCCAATCTTATTTCTTGGTGTGGATTGAATTTGGTCGATTCAGCTCCTAAAGGTTTGTTCAAGACCTCAAAATTAGTGGAAGATTTAGACATGGCTGTTGAGGAACAATGTAAAATCACTGAGACTTGGCTTACAGAATGTATACAAGATAATCTCCAAGTCGTCGCAAAATGTCCTGGCCAGCTTGTGACTAAGCATGTCGTCAAAGGTCCATGTCCACATTTTCAACTATACCTGTCAACACATGAAGACGCTAAAGCATATTTTACTCCTTTACTTGGGAAATATGATAAGAGCAGACTAAACAGGGCGGCCTTCATTAAAGATATTTCGAAATATGCAAAACCAATTTATATTGGTGAAATAAATTACGATGTCTTCGAAAAAGCTATTGAACGAGTGATTCGTACTCTCAGAGACGTGGGAATGCAACAATGCACGTATGTCACAGATGAGGAGGAGATTTTCAAATCACTCAATCTTAATGCAGCAGTTGGAGCACTATACACAGGGAAGAAAAAGGACTATTTCGCAGATTTTTCAGAAGAAGACAAAGCAGAAATAGTTATGAGATCCTGTGAACGCTTATATAATGGACAACTGGGCATTTGGAATGGATCGCTCAAAGCCGAGATACGGCCCATAGAGAAGACTATGCTGAACAAGACTCGTACTTTTACAGCAGCGCCATTGGAAACTTTACTTGGTGGAAAAGTGTGCGTCGACGATTTCAACAACCAATTCTATTCACATCATCTAGAGGGTCCCTGGACGGTTGGAATCACAAAATTCTATGGAGGATGGAACCGATTGCTCGAGAAGTTGCCAGAAGGATGGATTTACTGTGATGCTGATGGCTCACAATTTGACAGCTCCCTAACACCATATCTTATAAATGCCGTGCTACACATTCGACTACAATTTATGGAGGAGTGGGAACTAGGCGCACAAATGTTACGAAATCTGTACACTGAAATCGTCTACACACCAATTGCAACACCAGACGGTTCCATAATCAAGAAATTTAAAGGAAATAACAGTGGTCAGCCATCAACTGTAGTTGATAATACGCTAATGGTCATCTTAGCATTCAATTACGCAATGTTGTCAAGTGGAATACAAGACAATGAAATCGACAACTGTTGCAGGATGTTTGCTAATGGAGATGATCTATTATTGGCAGTACATCCCGATTATGAACATATACTGGATGGATTTCAAGATCACTTCGGAAATCTTGGATTAAATTTTGAGTTTACATCACGAACACGAGATAAGTCCGAACTGTGGTTCATGTCTACAAGAGGAATCAAATGTGAAGGAATCTACATACCTAAACTTGAGAAAGAAAGAATAGTCGCAATACTCGAATGGGATCGATCAAATCTACCTGAGCATAGATTGGAAGCCATTTGTGCAGCTATGGTTGAAGCATGGGGCTACCCAGATCTTGTTTACGAAATTCGGAAATTTTATGCGTGGCTTCTGGAAATGCAACCATTTGCAAATCTAGCCAAAGAAGGTTTAGCTCCATATATTGCAGAAACAGCGCTTCGTAACTTGTATCTAGGTTCAGGCATCAAGGAAGAGGAAATCGAGAAATACTTCAAGCAGTTTGCAAAGGACCTCCCTGGGTATTTAGAGGATTATAACGAAGAAGTTTTCCACCAAGCTGGAACAGTCGATGCAGGCGCGCAAGGAGGAGATGGGAAAGCCGGGACTCAGCCGCCAGCCACAGG-------------------------------------------------AGCAGCAGCTCAAGGAGGAGCTCAACCACCAGCAACTGGAGCAGCCGCGCAA---------CCACCCG---CGACTCAAGGTTCACAACCGCCCACAGGGGG----AGCCACTGG-----TGGAGGTGGTGCACA---------------------AACAGGAGCTGGCGAAACTGGTTCAGTTACAGGAGGTCAAAAAGACAAGGATGTAGATGCTGGTACGACAGGCAAAATCACAGTGCCAAAACTTAAAGCCATGTCGAAGAAGATGCGCTTGCCAAAAGCAAAAGGAAAAGATGTTTTGCATCTGGACTTTCTGTTAACATACAAACCGCAACAACAAGACATATCAAACACAAGAGCAACCAGAGAGGAGTTTGATAGATGGTATGAAGCCATAAAGAAGGAATATGAAATAGATGACACACAAATGACAGTTGTCATGAGTGGTCTAATGGTATGGTGTATTGAGAATGGTTGCTCACCAAACATAAACGGAAATTGGACAATGATGGATGGAGATGAACAAAGAGTCTTTCCATTAAAACCAGTCATTGAAAACGCATCTCCAACATTCCGGCAAATAATGCATCATTTCAGTGATGCAGCTGAAGCATACATCGAGTATAGAAACTCTACAGAGCGATACATGCCACGATATGGACTTCAGCGGAATCTCACCGACTATAGCTTAGCGCGGTATGCCTTCGACTTTTACGAAATGAATTCAAGGACACCAGCTAGAGCTAAGGAAGCCCACATGCAG---ATGAAGGCCGCAGCAGTCCGTGGTTCAAACACACGATTGTTCGGTCTGGACGGAAATGTCGGCGAGACTCAGGAGAATACAGAGAGACACACAGCTGGCGATGTCAGTCGCAACATGCACTCTCTGTTGGGAGTGCAGCAGCACCACTAGTCTCCTGGAAACCCTGTTTGCAGTACCTATAGTATGTACTA--------ATAATGTATGTCAGTGAGGTTTTACCTC-----------------------G-TTTTACTA---TATTACGTATGTACTTAAAGCGTGAACCAGTCTGCAGGACACAGGGTTGGACCCAGTGTCTTCTGGTGTAGCGTGTACTAGCGTCGAGCCACGTGACGGACAGCACTGGGTGTGGCTTTGCCATTGGTGCTGCGAGTCTCTTGGTGAGAGAC----------------

>JX188385

--------------------TAAAACAACAAAACTCAACACAACACAACAAAACACAACCAAGCAAATCCAATTTACTTGCGCTCAGATTGTAGTGAACGGCTCGAACGAAACGGTTCTTCGAGATAACTCTCTGATTCTTCCTCA----------TCTTTCAATTTCTTTCGAAGGAAATGGCGGGAACGTGGACCTACGTGACACGTAAGTGGCAGCCAGATGTTAACAATGATCGTCACATTAAAAGAGTGATGGAAATGTTTGCAGCAAAACATCAACATTACTCAGAAGAACAGCGACTTGCTCATAATATGAAACTATTGAGGAAGGCAAGTGTTGTAAGCGCTGAACCTGCGAAACCAAAGCAGAAGCAGGCAACTCAACAGATGTGGGTTGAGAAATGTGATCACAATCCTGTTGATCACTTAGTATATCCACGACTTGGAAGATCCGCAAACAAGGTG---GAAATGAATATTAAAAGAACATCTGTGAGCAAATTAACCAGGGAGGTTTTAGAGATCTCAAAGTCAAGCGGTCTTAAAGTTGAACTAATTGATAAACGGAAAAGATCCAAAACACAGTTATCAATCAAAAGGTTCAATGGTAAAAATTTCCTCCATTGCAAAACGAATCACGAGAACAATTTATTCAAAAGGAGGGACATAGCCATTGGACATAAATGGTTTCCAACAATTGAAGCCATTGCTCGATGCTATAGCACGATGAATCGAGAGGAACTACAAAGCCTTTGTAGAGGAAGCAGTGGTCTCACATTCATCCAAAACGAAGAGTTATTCATTGTCAGAGGGAGAATGAATGGTGAACTCATTAATAGCTTGCACGAAACAAATCGGGTTTTGGATATTGAGCACTACGCAGATCCCCAGGCTAATGATTTTTGGAGGGGATACACAAATGCTTACGTAGAGAATCGCAGCATTCCGACCACTCATACAGAGCACACCCCTACAGTCAATCTAGAGGAATGTGGAAAACGAATGGCTTTACTTGAAATACTATTTCACTCTACATTCAAAATTACATGCAAGGCGTGCAACATTGATGATCTTGAATTATCAGATGATGAATTTGGAGCCAAACTCTATAAGAATTTGCAACGTATCGAAGAAAAACAAAGAGAGTACCTTGCAAAGGATCAAAAACTATCCAAAATGATACAATTTATCAAAGAAAGGTGTAATCCAAAATTTTCGCATCTACCAACGTTATGGCAAGTTGCAGAAACAATAGGACACTATACTGATAATCAGTCAAAGCAAATAATGGACATTAGCGAAGCGCTCTTTAAAGTTAATACTTTAACTCCTGACGATGCTATGAAAGCAAGCGCAGCATTACTTGAAGTATCGCGATGGTATAAGAACCGTAAGGAGTCGCTCAAAACTGACTCATTGGAATCTTTTAGAAATAAGATATCACCAAAGAGTACAATAAATGCAGCTTTAATGTGCGATAATCAATTGGATAAAAATGCAAATTTTGTATGGGGTAATAGGGAATATCACGCCAAACGATTTTTCGCGAACTATTTTGAAGCAGTGGATCCCACAGATGCATATGAAAAGCACGTCACACGGTTTAACCCTAATGGTCAACGAAAGTTATCAATAGGAAAGTTAGTTATCCCACTGGACTTTCAAAAGATTAGAGAATCATTCGTTGGACTTCCGATAAATAGACAACCGCTGGGTAAATGTTGCGTTAGCAAGATCGAAGGAGGGTATATATACCCATGTTGCTGCGTCACAACAGAATTTGGTAAACCAGCATACTCTGAGATAATACCTCCAACGAAAGGCCACATAACAATAGGCAATTCTGTTGATCCAAAGATTGTGGACCTGCCAAACACAACACCACCTAGCATGTACATCGCTAAGGATGGGTATTGCTACATCAACATCTTTTTAGCAGCCATGATCAACGTTAACGAAGAATCTGCCAAGGATTACACGAAATTTTTGAGGGACGAACTAGTCGAGCGTCTCGGAAAGTGGCCAAAGCTTAAAGACGTAGCAACAGCGTGTTATGCATTATCCGTAATGTTTCCAGAAATTAAGAATGCTGAGTTACCTCCAATTTTAGTTGACCATGAAAATAAATCAATGCATGTAATCGATTCATATGGTTCACTAAGTGTTGGATTTCACATATTAAAAGCAAGCACGATTGGTCAATTAATCAAATTCCAATATGAATCTATGGATAGTGAAATGCGCGAATACATAGTAGGAGGAACTCTCACGCAACAGACATTCAACACACTTCTTAAGATGCTTACGAAAAACATGTTCAAACCAGAGCGCATCAAGCAGATAATTGAAGAGGAACCCTTTTTACTTATGATGGCGATTGCATCTCCAACGGTATTAATAGCACTATATAATAATTGTTATATTGAGCAAGCTATGACATACTGGATCGTGAAGAATCAAGGAGTTGCAGCCATATTCGCACAACTCGAAGCATTAGCCAAGAAAACGTCCCAGGCTGAGTTATTAGTTCTACAAATGCAAATACTTGAAAAGGCATCTAACCAACTAAGATTAGCAGTTTCAGGACTTAGCCATATCGACCCAGCGAAGCGACTTCTGTGGTCACACCTTGAGGCAATGACAACACGTTCAGAAATGAACAAGGAGTTAATAGCTGAGGGGTATGCACTATATGACGAGCGTCTATATACCCTGATGGAAAAAAGTTACGTAGATCAATTAAACCAATCATGGGCAGAATTATCATACTGTGGAAAATTTTCAGCAATATGGCGTGTGTTCAGAGTCAGGAAGTATTACAAACCGTCTTTAACCGTGAGAAAAAGCGTAGATTTAGGCGCTGTATACAATATATCAGCTACGCATCTAATATCAGATTTAGCGCGGAAAAGTCAAGATCAAGTCAGCTCTATTTTAACCAAACTCCGCAACGGTTTCTATGATAAATTAGAGAAAGCTAGAATACGTACTATAAAAACGGTTTATTGGTTTATACCTGATGTATTTAGACTCATGCACATATTCATAGTTTTGAGTTTATTAACTACCATAGCTAATACTATCATAGTAACTATGAGTGACTACAAGAAATTGAAGAAGCAACAAAGAGAAGACGAATATGAAGCAGAGATTAACGAAGTTCGCAAAATCCATTCTACCTTGATGGAGGAGCGGAAGGATAATTTGACGTGTGAACAATTTGTTGAATATATGCGCCAAAATCATCCGCGGTTAGTTGAAGCGACACTGGACTTAACTCACACAGGCGTCATACACGAAGGAAAATCTAATCTCGAAACCAATTTGGAACAGGCAATGGCAGTTGGAACCTTGATAACAATGATACTTGATCCACAGAAAAGCGATGCTGTCTATAAGGTGTTGAACAAAATGCGGACAGTAATTAGTACAATTGAACAAAATGTCCCATTCCCTTCAGTGAACTTCTCCAACATCTTGACACCTCCAGTGACACAACAGAGTGTAGATGTTGATGAGCCATTGACACTTAGCACTGATAAAAACCTAACAATAGACTTTGACACGAATCAAGATTTACCTGCCGATACATTCAGTAATGATGTGACATTTGAAGATTGGTGGTCAAATCAGTTAAGTAACAACAGAACAGTGCCACACTACCGACTTGGAGGAAAGTTCATCGAGTTTACACGAGAAAATGCAGCCCACACGAGCATCGAACTTGCACACTCAAACATCGAAAGGGAATTCTTGCTTAGAGGAGCGGTCGGCTCGGGAAAATCCACCGGGTTACCATACCATCTTAGCATGCGTGGAAAAGTGCTTCTACTAGAGCCTACAAGACCGCTAGCTGAGAACGTGTGTAGGCAATTACAAGGACCGCCATTTAACGTAAGTCCAACTCTTCAAATGCGTGGACTAAGTTCTTTTGGATGCACTCCAATCACAATCATGACATCCGGTTTTGCATTGCACATGTACGCAAACAATCCAGATAAAATATCCGAATACGATTTTATTATCTTCGATGAATGTCATATAATGGAAGCACCAGCGATGGCCTTTTATTGCTTACTCAAAGAATATGAGTACCGAGGGAAAATTATCAAGGTATCAGCTACGCCTCCAGGAAGAGAGTGTGAATTCACAACACAACATCCAGTAGACATCCATGTTTGTGAGAATCTAACTCAGCAACAGTTTGTTATGGAACTCGGGACTGGTTCAACCGCAGATGCTACGAAGTACGGAAATAATATCTTAGTTTATGTAGCAAGCTATAATGACGTCGATTCATTGTCACACGCATTAGCCGAACTTAAATTTTCCGTAATCAAAGTGGATGGCCGAACAATGAAACAAAACACAACAGGAATCATTACAAACGGTACCTCACAAAAGAAGTGTTTTGTTGTCGCAACAAATATAATTGAAAATGGTGTTACACTAGATATTGATGTTGTTGTCGACTTCGGACTCAAGGTCTCAGCTGACTTGGACGTTGACAACAGGGCGGTATTGTATAAACGCGTAAGTATATCATATGGTGAACGCATACAACGATTGGGTCGTGTTGGCAGAAATAAACCTGGTACAGTTATTCGTATCGGAAAAACAATGAAGGGTTTGCAGGAAATTCCAGCAATGATCGCAACAGAAGCAGCCTTCATGTGTTTCGCATATGGTCTTAAAGTCATCACTCATAATGTTTCAACGACTCACCTTGCAAAGTGCACAGTTAAACAAGCGAGAACCATGATGCAATTCGAATTATCACCATTTGTCATGGCTGAGCTCGTTAAGTTTGATGGGTCAATGCATCCACAAATACATGAGGCACTAGTAAAGTACAAACTTAGAGATTCTGTCATAATGCTCAGACCGAATGCAATTCCAAGGGTCAATTTACACAATTGGCTTACAGCCCGGGATTATAATAGAATAGGATGCTCATTGGAACTCGAGGACCACGTCAAAATTCCGTACTACATTAGGGGAGTTCCTGACAAGTTGTATGGAAAGCTATATGATATTATTTTACAGTATAGTCCAACTAGTTGCTACGGTAGATTGTCAAGTGCGTGTGCAGGTAAAGTAGCATATACTTTGCGGACTGATCCATGTTCACTTCCAAGAACAATAGCAATAATTAATGCCTTAATCACGGAGGAGTATGCGAAGAGAGATCACTATCGTAACATGATTTCAAACCCATCTTCATCACACGCATTCTCACTCAATGGATTGGTGTCTATGATCGCCACTAGATACATGAAAGACCATACAAAAGAGAATATTGACAAACTTATTAGAGTGCGTGATCAATTACTTGAGTTTCAAGGTACTGGAATGCAATTTCAAGATCCGTCAGAACTTATGGAAATTGGGGCTCTTAACACAGTTATTCACCAAGGAATGGACGCAACTGCAGCTTGTATCGGGTTACAAGGACGATGGAATGCTTCACTTATACAACGCGATCTCCTAATTGCAGGTGGAGTTTTTATCGGAGGCATTTTGATGATGTGGAGCCTATTTACTAAATGGAGTAACACAAATGTCTCACATCAGGGGAAAAACAAACGCAGTAGACAAAAACTTCGATTCAAAGAAGCAAGAGACAACAAATATGCATATGACGTCACAGGATCGGAAGAATGCCTCGGTGAAAATTTTGGAACAGCCTATACAAAGAAAGGTAAAGGAAAAGGAACTAAGGTTGGACTCGGTGTGAAGCAGCATAAATTCCATATGATGTATGGTTTCGATCCTCAAGAGTACAACCTAATTCGGTTTGTCGATCCACTCACGGGAGCAACTCTGGATGAACAAATTCATGCCGATATGCGTTTAATTCAAGAGCACTTCGCTGAAATTCGTGAGGAGGCAGTGGCTAATGACACAATTGAAAGGCAGCAGATTTACGGCAATTCTGGACTACAAGCATTTTTCATACAAAATGGGTCAGCAAACGCTCTGAGAGTTGATTTAACACCACATTCACCTACACGAGTTGTCACAGGTAATAACATAGCAGGGTTCCCAGAATATGAAGGGACACTTCGTCAAACTGGAACAGCTATAACCATACCCATTGGTCAAGTCCCAATCGCAAATGAAGCAGGGGTTGCACACGAGTCAAAATCCATGATGAATGGGTTGGGTGATTACACGCCAATATCGCAACAATTGTGTTTAGTACAAAATGACTCCGATGGGGTAAAGCGGAATGTATTTTCAATTGGATATGGCTCATATCTTATTTCACCAGCGCACTTATTCAAATACAACAATGGTGAAATAACAATTAGATCATCAAGGGGATTATACAAAATTCGCAATTCTGTGGATTTAAAATTACATCCGATTGCACACAGGGACATGGTCATAATTCAACTCCCAAAAGATTTCCCACCGTTCCCAATGCGCTTGAAATTCACACAACCATCACGAGATATGCGAGTCTGCTTAGTAGGAGTCAACTTCCAACAGAACTATAGCACTTGCATCGTATCAGAAAGTAGTGTGACAGCACCAAAAGGAAATGGAGATTTTTGGAAACATTGGATATCAACAGTTGACGGTCAATGCGGACTACCATTGGTAGATACTAAGAGTAAACATATTGTTGGAATTCATAGTCTTGCATCAACAAGTGGAAACACTAATTTCTTTGTCGCTGTGCCTGAGAACTTTAATGAATACATCAATGGACTTGTGCAAGCAAATAAATGGGAAAAAGGATGGCACTATAATCCGAACCTCATATCCTGGTGTGGAYTAAATTTAGTCGATTCTGCCCCAAAGGGTTTGTTTAAAACGTCAAAACTGGTAGAAGATTTGGACGCGAGCGTTGAGGAGCAATGCAAGATCACTGAAACATGGCTCACAGAACAATTACACGATAATTTGCAAGTGGTTGCGAAATGTCCAGGCCAACTCGTTACCAAGCATGTTGTTAAGGGTCAATGCCCACACTTTCAATTGTACTTATCAACACATGACGATGCCAAAGAATATTTCACACCCATGCTTGGAAAATACGACAAGAGTAGGCTCAACAGAGCAGCTTTTATTAAAGACATATCGAAATATGCAAAGCCAATTTATATTGGGGAAATTAAGTATGATGTCTTTGATAGAGCTGTACAGCGAGTTGTCAACATCCTCAAAAATGTTGGAATGCAACAATGTGTTTATGTCACAGATGAAGAGGAAATTTTCAAATCGCTTAACCTAAACGCAGCTGTCGGAGCATTGTATACAGGAAAGAAGAAAAATTACTTTGAAAATTTTTCAAGCGAAGACAGAGAAGAAATCGTGATGAGATCCTGTGAACGTATTTACAATGGGCAACTTGGCGTATGGAATGGATCGCTCAAAGCTGAGATCAGACCAATAGAGAAAACCATGCTGAATAAGACTCGAACCTTCACAGCAGCCCCATTAGAAACTTTGCTCGGAGGAAAAGTGTGCGTGGATGATTTTAATAATCAATTCTATTCACATCATTTAGAAGGTCCATGGACTGTTGGGATAACAAAATTCTATGGAGGTTGGAATCGCTTACTCGAGAAGTTACCAGAAGGATGGGTTTACTGCGATGCTGACGGGTCTCAATTTGATAGTTCATTAACACCATATCTCATCAATGCAGTATTAAATATTCGATTGCAATTTATGGAAAATTGGGATATAGGAGCACAAATGCTAAAGAACCTTTATACTGAGATTGTTTACACACCAATCGCAACGCCAGATGGATCAATCGTAAAGAAATTCAAAGGAAATAATAGCGGACAACCTTCTACAGTAGTGGACAACACATTGATGGTTATAATAGCTTTCAACTATGCTATGCTATCAAGTGGAATCAAAGAAGAAGAGATCGATAATTGCTGTAGAATGTTTGCGAATGGTGATGACTTACTCCTAGCAGTGCATCCTGATTTTGAGTTCATTTTGGATGAATTTCAGAATCACTTTGGGAATCTTGGGCTGAACTTCGAATTCACATCACGAACACGAGACAAAACCGAACTGTGGTTCATGTCCACAAGAGGCATCAAGTATGAAGGAATTTACATACCAAAGCTTGAGAAAGAAAGAATAGTCGCCATACTTGAATGGGATCGATCAAACTTGCCTGAACACAGATTGGAAGCTATATGTGCAGCGATGGTTGAGGCCTGGGGATATCCTGATCTTGTTCATGAGATACGAAAGTTCTATGCGTGGCTTTTGGAGATGCAACCTTTTGCAAACCTCGCAAAAGAAGGGTTGGCCCCATATATTGCCGAGACAGCACTCCGCAATCTCTATCTTGGAACAGGCATCAAAGAGGAAGAAATTGAAAAATATTTTAAACAATTCATTAAGGATCTTCCTGGATACATAGAAGATTACAACGAGGATGTATTCCATCAGTCGGGAACTGTTGATGCAGGTACACAAGGAGGCAGTGGAAGCCAAGGAACAACACCACCAGCAACAGGCAGTGGAGCAAAACCAGCCACCTCAGGGG------------CAGGATCTGGTAGTAGCAC----AGGAGCTGGAACTGGTGTAACTGGAAGTCAAGCAGGG---------GCT---G---GCGGTAGCGCTGGGACGGGATCCGGAGCAACCGGAGGCCAAYCAGGATCTGGAAGTGGCACTGGACAGATTAACACGGGTTC---AGCAGGAACTAGTGCA------------ACAGGAGGCCAAAGAGATAGGGATGTGGATGCAGGTACAACAGGAAAAATTTCTGTACCAAAGCTCAAGGCCATGTCAAAGAAAATGCGCTTACCTAAAGCAAAAGGAAAAGATGTGCTACATTTGGATTTTCTATTGACATATAAACCACAACAACAAGACATATCAAACACTAGAGCAACCAAAGAAGAGTTTGATAGATGGTATGATGCCATAAAGAAGGAATATGAAATTGATGACACACAAATGACAGTTGTCATGAGTGGTCTTATGGTATGGTGCATCGAAAATGGTTGCTCACCAAACATAAACGGAAATTGGACAATGATGGATGGAGATGAACAAAGGGTTTTTCCACTCAAACCGGTCATTGAGAATGCATCTCCAACTTTCCGACAAATTATGCATCATTTTAGTGATGCAGCTGAAGCGTATATAGAGTACAGAAACTCTACTGAGCGATATATGCCAAGATACGGACTTCAGCGCAATCTCACCGACTACAGCTTAGCACGGTATGCATTTGACTTCTACGAAATGACTTCACGCACACCTGCTAGAGCTAAAGAAGCCCACATGCAG---ATGAAAGCTGCAGCAGTTCGTGGTTCAAACACACGACTGTTCGGCTTGGACGGAAATGTCGGCGAGACCCAGGAGAATACAGAGAGACACACAGCTGGCGATGTTAGTCGCAATATGCACTCTCTGTTGGGAGTGCAGCAGCACCACTAGTCTCCTGGAAACCCTGTTTGCAGTACCAATAATATATACTA------ATATATAGTACTTTAGTGAGGTTTTACCTC-----------------------GTCTTTACTATTTTATTACGTATGTATTTAAAGCGTGAACCAGTCTGCAGCATACAGGGTTGGACCCAGTGTGTTCTGGTGTAGCGTGTACTAGCGTCGAGCCATGAGATGGACTGCACTGGGTGTGGCTTTGCCACTTGTGCTGCGAGTCTCTTG-------------------------

>JX185303

---------------------------------------------CAACAAAACACAACCAAACAAAACCAAGTTACCTTCGCTCAGATTGTAGTGAACGGCTCGGTGGAAAAGGTTCCTCGAGATCACTCTCTGATTCTTCTCTC----------TCAACCAACTTCATTCAAGCGAGATGGCGGGCTCTTGGACTCACGTGACATACAAGTGGCAACCAGATGTCAACAACACACGCGATGTCAAAAGAGTGATGGAGATGTTTGTAGCAAAACATCAACGTTACACTGAGGAGCAAAGGCTTGCTCATAACAGCAAGCTATTGWGAAAGACTCGTGTGATTAGTGCTGAGTTTATTGAACCAGCACAGAAACCAAAATGTCATCAGACATGGGTTGAAAAGTGCGATCACAACCCCACAGAGCACTTCATTTATCAACGTTTC---ACACCTAAAAAGAAAGTGCTTAGTACTAAGCCTGAGACAACTTCTGTAACGAAGTTAATCAGGGACGTCCTTGAAATCTCGAAGGGTAGTGGAATCAAAATTGAGTTAATTGACAAGCGCATAAAACGCAAGACTCAACTATCTATAAGGAAACACAATGGCAAAGATTTCCTGCATTGCAAAACCAGGCATGAAAATGGCTTGTTCAAACGCAAGGACATTGACATTAGTGTCAAGTGGTTACCAACCATTGAATCTATTGCAAAATGCTACAGCACGGTGAATGCAAAAGAACTACAAAGTCTCAATAGAGGCAGTAGTGGTCTCACATTCATGCAAAATGGTGAATTGTTCATCGTGCGTGGAAGGATGCATGGTGAAATTGTTAATAGTCTGCGCGAAAGTAAGCATGTGATGGAAATTGAACACTATGCTGATCCACAAGCAAATAGTTTCTGGAAAGGCTATACAGACGCGTATGTCGAAAACAGAAACATATCCACCACTCACACAGAGCACACACCAACTATTAATTTAGAGGAGTGTGGCAAGAGAATGGCATTGTTAGAAATACTATTTCACTCAACTTTTAAAATCACATGCAAAACGTGTAATATTGACGATCTGGAATTATCAGATGATGAATTTGGGGCCAAGTTATATAGCAATCTGCAGCGTATTGAAGAAAAGCAACGTGAATATCTTGCTAAAGATCAAAAACTTCTACGCATGATACACTTCGTAAAGGATCGGTGTAACCCAAAATTTTCACATTTGCCTCTACTATGGCAAGTGGCAGAAACAGTAGGACATTACACTGACAATCAATCAAAGCAGATAATTGATATCAGTGAGGCGCTTATCAAAGTTAATACTTTAACTCCTGATGATGCAGTGAAGGCCAGTGTGGCATTATTGGAAGTGGCACGATGGTATAAAAATCGGAAGGAGTCGCTTAAAACGGACACATTGGATTCATTTCGAAACAAGATCTCACCAAAGAGCACGATCAACGCAGCATTAATGTGTGATAACCAGCTAGATAAGAATGCGAATTTCGTATGGGGAAACAGAGAATACCATGCGAAGCGATTCTTCGCTAATTATTTTGAAGCTGTGGACCCAACTGATGCATATGAAAAACACGTAACACGCTTTAACCCCAATGGACAGCGGAAACTATCAATTGGCAAGCTAGTAATTCCACTAGATTTCCAGAAGATTAGAGACTCGTTCGTTGGCCTATCAATAAATAGACAACCACTGAGTAAAGCTTGCGTAAGCAAGATTGATGGAGGCTACGTGTATCCATGCTGCTGCGTTACAACGGAGTTTGGAAAACCAGCATATTCTGAGATAATACCTCCAACGAAAGGGCATATCACGATTGGAAATTCAGTGGACCCAAAAATAGTGGATTTACCGAATACAACACCACCGAGTATGTATATTGCAAAAGATGGATATTGTTACATTAACATATTCCTGGCAGCAATGATAAACGTCAATGAGGAATCTGCAAAAGATTACACTAAATTCCTTAGGGACGAATTGGTGGAACGGCTTGGTAAATGGCCAAAATTGAAGGATGTAGCCACAGCCTGTTATGCTTTATCAGTGATGTTCCCGGAAATAAAGAAYGCTGAATTACCACCAATACTAGTAGATCATGAGAGTAAGTCTATGCACGTCATCGATTCATACGGATCGCTCAGTGTTGGATTTCACATTCTAAAGGCGAGCACCGTTGGACAACTGATAAAATTTCAGTATGAGTCACTGGAAAGTGAGATGCGTGAGTACATAGTAGGAGGTACTTTGACACAGCAAACTTTCAGTACACTTCTTAAGACTCTCACAAAGAACATGTTTAAGCCGAACAAGATAAGGCAGATAATTGAGGAAGAGCCCTTCTTACTAATGATGGCAATTGCATCTCCAACTGTACTCATCTCGCTATACAACAACTGCTACATCGAACAGGCAATGACGTATTGGATTGTCAAGAACCAAGGCATCGCAGCAATTTTTGCGCAGTTGGAGGCATTAGCAAAGAAAACTTCTCAAGCGGAACTACTAGTTCTTCAAATGCAAATACTTGAGAAAGCTTCAAACCAACTGAGACTCGCAGTTACAGGACTGAATCATGTTGATCCAGCAAAACGACTTTTATGGTCTCACTTGGAAGCTATGACAACACGAACGGAGATGAATAAGGAACTCATAGCAGAAGGTTATGCACTATATGACGAACGCTTATACACTTTAATGGAAAAAAGTTACGTAGATCAATTAAACCAATCATGGGCAGAGTTATCATATTGTGGAAAATTTTCAGCAATATGGCGTGTGTTCAGAGTCAGGAAATACTACAAACCATCCTTAACCGTGAGAAAAAGCGTAGATTTAGGCGCTGTGTACAATATATCAGCTACGCATCTAATATCAAATTTAGTGCAGAAAAGTCGAGATCAAGTCAGCTCTACTTTAACCAAACTCCGCAACGGTTTCTATGATAAAATGGAGAGAGCGAGAGTTAGTGCAGTAAGGACAATATACTGGTTTGTACCTGATATATTTAGATTAATTCATATTTTCTTAGTTTTAAGTTTGTTAACAACTATAGCTAATACAATAGTCACAACTATGAATGATTATAAAAAGTTGAAAAAGCAACAAAGAGAAGACGAATATGAAGCCGAGATTAATGAGGTACGGAGGATACACGCCAATCTGATGAAGGAGCATAATGATAATCTGACATGCGATCAATTTATTGAACACATACGACAGACACATCCACGCCTCATCGAAGCAACATTGGATTTAACACATACAGGTGTCATCCATGAGGGTAAGTCCAATTTGGAAACCAACCTCGAACAGGCAATGGCCGTGGGAACTTTACTCACTATGATACTCGATCCACAGAAGAGCGATGCAGTTTATAAGATTCTCAATAAAATGCGAACGGTGATTAGCACAATAGAACAAAATGTGCCATTCCCATCAGTGAACTTCACGAGCATCTTGGCACCTCCTGTAGCTCAGCAGAGTGTAGATGTTGACGAACCGTTAACACTGAGTACCGACAAGAATTTAACTATAGATTTCGACACAAATCAAGATTTGCCAGCAGATACATTTAGCAATGACGTTACATTCGAGAACTGGTGGGCTAATCAGATAAACAACAACAGAACAGTGCCACACTATCGACTTGGGGGAAAGTTTGTAGAATTCACAAGAGATAATGCAGCAATGGTTAGCATTGAACTTGCCCACTCGAACATCGAAAGAGAATTTCTACTCAGAGGAGCTGTTGGGTCAGGAAAATCCACAGGTTTGCCATATCATCTCAGTATGCGTGGAAAAGTGCTGTTGATAGAACCTACTCGACCGTTAGCTGAGAACGTCTGCAGGCAACTGCAAGGTCCTCCATTTAATGTGAGTCCCACTCTACAAATGAGAGGTTTGAGCACGTTTGGCTGCACTCCTATCACGATAATGACATCTGGTTTCGCATTGCACATGTATGCTAATAACCCCGATAAGATTTCTGAATACGACTTCATCATCTTTGATGAATGTCACATTATGGAAGCACCTGCGATGGCATTTTATTGTTTGCTTAAGGAGTATGAATACCGAGGCAAGATAATAAAAGTTTCAGCTACACCACCAGGACGAGAATGCGAATTTTCAACCCAACATCCAGTAGATATACATGTATGTGAAAGCTTGACACAACAGCAATTCGTCATGGAACTAGGAACAGGATCAACTGCTGACGCAACCAAATATGGCAATAACATATTAGTGTACGTTGCGAGTTATAATGACGTAGATTCTTTATCCCATGCGCTAACTGAACTTAAGTATTCAGTAATCAAAGTCGACGGAAGAACAATGAAGCAAAACACCACGGGAATCGTAACAAATGGAACATCCAGTAGGAAATGCTTCATTGTGGCCACGAACATTATTGAAAACGGAGTAACGCTAGATGTCGATGTCGTCGTCGACTTTGGGCTTAAAGTAACAGCCGAATTAGATGTTGATAACAGGGCGATAATGTATAAACGCGTGAGCATATCTTATGGCGAGCGCATTCAGAGACTCGGGAGAGTTGGAAGGAATAAGCCTGGGACAGTAATCCGCATCGGAAAAACTATGAAAGGTTTACAAGAAATTCCAGCGATGATTGCCACTGAAGCAGCTTTCATGTGTTTTGCATACGGACTGAAGGTCATAACACATAATGTATCAACAACACATCTGGCAAAATGCACTGTCAAACAAGCCAGAACCATGATGCAATTTGAGCTATCACCATTTGTAATGGCTGAGTTAGTCAAATTTGACGGTTCTATGCACCCACAGATTCATGAAGCATTAACCAAATATAAGTTGAGAGATTCTGTAATCATGTTAAGACCAAATGCAATACCAAAGGTTAATCTTCACAACTGGCTGACGGCTCGTGATTACAACAGAATAGGCTGCTCATTGGAACTCGAAGACCACGTCAAAATACCATATTATATACGGGGAGTTCCTGACAAGTTGTATGGGAAGTTATATGATATTATCCTTCAATATAGTCCTACAAGTTGCTATGGAAGACTATCAAGTGCTTGCGCGGGCAAAGTCGCATATACATTGCGCACTGATCCTTGTTCATTACCAAGAACAATAGCTATAATCAACGCACTGATTACTGAAGAATACGCAAAGAGGGATCATTATAGAAATATGATAGCGAACCCCTCATCATCGCATGCCTTTTCACTCAATGGGCTAGTATCCATGATCGCTTCTCGGTACATGAAAGACCACACGAAGGAAAATATTGAAAAACTCGTAAGAGTACGCGATCAACTAATCGAGTTCCAAAGCACAGGCATGCAGTTTCAAGATCCTTCAGAAATAATGGACATTGGTGCATTGAACACAGTTATCCACCAAGGAATGGATGCCACGGCTGCCTGTATTGGATTACAAGGGCGTTGGAATGCTTCACTCATTCAGCGCGATTTGATGATATCAGCAGGGGTTTTCACAGGAGGAATTCTTATGATGTGGTGTCTTTTCACAAAATGGAGTAAAACAGAAGTGTCACATCAAGGAAAGAACAAGCGTAGTCGGCAAAAATTACGATTCAAAGAGGCTCGTGATAACAAATACGCCTATGACGTAACAGGATCAAAGGACGCAATCGAAGAAAATTTTGGATCCGCGTATACTAAGAAAGGCAAAGGTAAGGGGACAAAAGTCGGTTTAGGAGTCAAGCAACACAAATTCCACATGATGTATGGCTTCGATCCTCAAGAGTACAACCTCATTCGTTTTGTCGATCCACTTACAGGAGCCACATTGGATGAACAGATCCATGCCGATATACGTCTAGTTCAAGAACATTTCGATGTTCTTCGGGAGGAAGCAGTAGCAAACGACACGATTGAGAGACAGCATATATATAGCAGTCCCGGTTTGCAAGCATTTTTCATACAGAATGGATCAGCTAATGCATTAAGAGTCGATCTAACGCCACACACACCCTTACGTGTTGTCACAAACAACAATATAGCAGGCTTTCCAGAATACGAAGGTACTCTTCGTCAAACCGGAACTGCCATCACTTTACCTGTAAACCAAGTTCCAGTAGCTAATGAAACAGGAGTGGCACACGAATCTAAATCAATGATGATTGGACTAGGTGATTACACACCAATTTCACAACAATTGTGCTTAGTCCAAAATGACTCTGACGGGGTGAAAAGAAACGTGTTTTCAATTGGCTATGGATCGTACCTTATATCACCAGCGCATTTATTCAAGTATAATAATGGTGAAATTACGATTAGGTCATCGAGGGGTTTGTATAAGATAAGGAATTCAGTAGAACTCAAGTTACATCCTATTGCACACAGAGATATGGTTGTAATTCAACTCCCTAAAGATTTCCCACCATTTCCGATGCGCCTTAAATTTTCAACACCAACACGGGAATCACGGGTGTGCTTAGTTGGAGTAAATTTTCAACAAAATTACAGTACCTGCATTGTATCAGAGAGCAGCGTGACAGCGCCAAAAGGAAATGGGGATTTTTGGAAACACTGGATCTCTACAGTGGATGGACAATGCGGTCTTCCATTAGTAGATGTTAAAAGCAAACATATAGTCGGAATTCATAGTCTTGCATCGACGAGTGGAAACACAAACTTCTTTGTCGCCGTACCGGATAACTTCAATGAGTACATCAGCAATCTTGTACAAACAAACAAGTGGGAAAAAGGATGGCACTACAATCCAAATCTAATTTCTTGGTGTGGTTTAAATTTAGTTGATTCAGCACCTAAAGGCTTATTCAAGACATCTAAACTAGTTGAAGACTTAGACATGAGTGTTGAAGAACAATGCAAGGTGACAGAAACATGGCTCACAGAATGTATCCAGGACAATCTACAAGTTGTGGCAAAATGTCCAGGCCAACTCGTAACTAAACACGTTGTCAAAGGCCCGTGTCCACACTTTCAATTGTATTTATCAACACATGACGAAGCCAAAACATACTTTGCCCCTCTACTTGGAAAGTATGATAAGAGCAGATTGAACAGAGCAGCATTCATCAAGGATATATCAAAGTATGCGAAACCGATTTATGTTGGTGAAATTAACTACGATATCTTTGAAAAGGCAATCGAGCGAGTTATTAAGATTCTCAAAAACGTCGGCATGCAGCAATGCGTTTATGTCACGGATGAAGAAGAAATTTTCAACTCACTCAATCTTAATGCAGCCGTCGGCGCCCTATACACAGGCAAGAAGAAGGATTATTTCAAGGATTACTCAAATGAGGATAAAGCCGAAATCATCATGCGATCTTGTGAGCGGATCTACAATGGACAACTTGGCATCTGGAATGGGTCACTCAAAGCTGAAATACGCCCAATTGAGAAAACCATGTTGAATAAAACACGCACTTTCACAGCAGCACCATTGGAAACTCTACTTGGCGGAAAGGTTTGCGTGGACGATTTTAATAATCAGTTTTATTCGCATCACCTTGAAGGCCCATGGACAGTTGGAATCACAAAATTTTATGGAGGATGGAATCGTCTACTCGAGAAGTTACCAGAAGGCTGGATTTATTGCGACGCCGATGGTTCACAATTTGATAGCTCATTAACGCCATACCTTATCAACGCCGTTTTACATATTCGGCTGCAATTTATGGAAGAATGGGCATTAGGGGCACAAATGTTGCAAAATCTGTACACCGAAATTGTTTACACACCAATTGCAACGCCAGATGGATCAGTCATTAAGAAATTCAAAGGAAACAACAGTGGCCAGCCCTCTACAGTTGTTGATAACACACTCATGGTCATATTAGCATTCAATTATGCAATGTTATCGAGTGGTATCAAAGAAGATGAAATAGACAACTGCTGCCGAATGTTCGCTAATGGAGACGATCTATTGCTGGCAGTGCATCCGAACTTTGAACATATACTGGATGGATTTCAAAATCACTTTGGAAATTTAGGCCTCAATTTTGAGTTTACATCACGAACAAAGGACAAATCAGAACTGTGGTTTATGTCCACACGAGGTATCAAATGTGAGGGCGTCTATATACCAAAGCTTGAGAAAGAAAGAATAGTTGCTATACTCGAGTGGGATCGGTCAAACTTACCTGAGCACCGTCTCGAAGCTATATGCGCAGCCATGGTAGAAGCATGGGGATATCCAGACCTTGTTCAAGAAATACGAAAGTTCTATGCGTGGCTTCTCGAAATGCAACCATTCGCAAATCTAGCGAAGGAGGGCTTAGCACCATATATAGCAGAAACCGCACTCAGAAATTTATACTTGGGCACAGGAATCAAGGAAGAGGAGATTGAAAAATACTATAAACAGTTTGCTAAAGATCTCCCTGGCTATATAGAAGACTATAATGAGGAAGTTTTCCATCAATCCGGTTCTGTGGATGCGGGAGCTCAAGGAGGAAATAGTGGCTCAGGAGCATCCACTCCTGCAGCG---------------------------------------------------GGTAGTGGGTC----AGGAACTAGACCACCTTCAACTGGATCAGCAGCACAAGGAAATACACCTACAG---CATCAGGTGGATCATCAGGAAACAATGGAAGC---AATCAATCAGGTTC---AAATGGCACCGG------------AAACCA---AGCAGGCGCAAGCGGA------------ACAGGGGACCAAAGAGACAAAGATGTCGACGTTGGTTCAACAGGAAAGATATCAGTGCCAAAACTTAAGGCGATGTCGAAGAAAATGCGTCTGCCAAAGGCAAAAGGAAAAGATGTCTTACACTTAGATTTTCTGTTGACATATAAACCACAACAACAAGACATATCAAACACTAGAGCAACCAAGGAAGAGTTTGATAGATGGTATGACGCCATAAAGAAGGAGTATGAAATTGATGACACACAAATGACAGTTGTCATGAGTGGTCTCATGGTATGGTGCATCGAAAATGGTTGCTCACCAAACATAAACGGGAATTGGACGATGATGGATGGAAATGAACAAAGGGTTTTTCCATTGAAACCAGTTATTGAAAATGCATCTCCAACTTTCCGACAAATTATGCATCACTTCAGTGATGCAGCTGAAGCGTATATAGAGTACCGAAACTCTACAGAGCGATATATGCCAAGATACGGACTTCAGCGAAATCTCACCGACTATAGCTTAGCACGGTATGCTTTTGATTTCTATGAAATGACTTCACGCACACCAGCTAGAGCTAAGGAAGCCCACATGCAG---ATGAAGGCCGCAGCAGTTCGTGGTTCTAACACACGCCTGTTCGGTCTGGACGGAAATGTCGGCGAGACTCAGGAGAATACAGAGAGACACACAGCTGGCGACGTCAGTCGCAACATGCACTCTCTGTTGGGAGTGCAGCAGCACCACTAGTCTCCTGGAAACCCTGTTTGCAGTACCTATAGTACGTACTTTATAATTGATATGGTATGTATGTGAGGCTTTGCCTC-----------------------GGGTTTACTATTTAATTACGTATGTACTTTAAGTGTGAACCAGTCTGCAGGATACAGGGTTGGACTCAGTGTCTTCTGGTGTAGCACGTACTAGCGTCGAGCCACGTCACGGACGGCATTANGGATGGCTTTGCCATGGGTGCTGCGAGTCTCTTG-------------------------

>JN021933

---------------------AAAACAACAAAACTCAACAACACACAACAAAACACAACCAAACAAAACCAAGTTTTCTTTGCTCAGATTGTAGTGAACGGCTCGCAAGAAACGGTCCTTCGAGATCACTCTCTGATTTCTTTCTC----------TCTCACACTTGCATTCAAGCGGAATGGCGGGATCGTGGACTCACGTGTCTTACAAGTGGCAGCCAAATGTCAACAATGACCGTGATGTAAGAAAGGTAATGGAAATGTTTGCAGCAAAACATCAACATTACACAGAGGAGCAGCGACTTGCACATAACAGCAAGTTGCTAAGGAAGGCGTATGTTGTGGACGTTGAACCAGTGAAGCCAGCACCGGAGCCTATAAGGCGTAAAGTGTGGGTGGAAAAATTCGATCACAACCCAACCGAAGACCTGGTGTATCCGCGCCTT---GTCACGGTTAAAAAGGCAGCAGAAATGAAGCCCGTGAACACCTCTATAAATAAACTTATAAGGGATGTTCTAGACATATCAAAAGGGAGCAGCCTTAAACTTGAGCTGATTGGCAAACGCCAGAAGTGCAAAACACAACTAGCTATTAAGAAATACAACAATAAGGACTACCTCCATTGCAGGACACGTCATGAAGACAACATGTTCAAGAGGAAGGACGTGGCAATCGGCATTGAATGGATCCCAACCATTGAAGCGATTGCTCGATGCTACAGCACAGTAAACAAACAGGAGATGCAAAGTCTCTACAAAGGCAGTAGTGGCTTAACATTCATGCAAAATGATGAATTATTCATTGTTAGAGGCAGAATGAATGGCGAGTTAGTTAATAGCCTGGAAGAGAACAGGAACGTGCTCGACATCGATCACTACGCTGATCCACAAGCGAACGACTTTTGGAAAGGGTACACCGATGCATACGTTGCGAATCGCAGTATCTCCACTACACATACCGTGCATACTCCAACCATCAACTTGGTAGAGTGTGGAAAGAGAATGGCATTACTTGAAATTTTATTCCACTCAACTTTTAAGATTACATGTAAGACATGCAATATTGATGACTTGGAGTTATCAGACGATGAATTTGGTGCAAAACTTTTCAAGAATCTACAACGTATCGAGGAACAGCAACGCGAATATCTCGCTAAAGATCAAAAGCTGCGTCGCATGATTCAATTCATCAAAGAAAGGTGTAATCCAAAGTTTTCTCACCTACCATTGCTTTGGCAAGTTGCAGAAACAATAGGACATTATACGGACAACCAGTCAAAGCAAATAATTGATATCAGTGAAGCACTCATCAAAGTTAACACATTAACACCTGATGATGCAGTCAAGGCGAGTGTAGCACTGCTTGAGGTGGCACGATGGTATAAAAATAGGAAAGAATCACTTAAAACAGACACATTAGATTCGTTCCGAAACAAGATATCCCCAAAGAGCACGATAAACACAGCATTGATGTGTGACAATCAACTAGACAAGAATGCAAATTTTGTCTGGGGAAACAGGGAATATCATGCAAAGCGCTTCTTCTCAAATTACTTTGAAGCAGTAGATCCTACTGACGCATACGAGAAGCACGTAACCCGTTTCAATCCAAATGGTCAACGGAAATTATCAATTGGTAAACTAGTAATTCCGTTAGATTTCCAGAAAATAAGAGACTCGTTCGTTGGTTTGGCAATAAACAAACAGCCACTTACAAAGGCTTGTGTGAGCAAAATCGATGGTGGTTATGTATACCCATGCTGTTGTGTCACAACAGAATTTGGAAAACCAGCATACTCTGAGATAATACCACCAACAAAGGGCCATATAACAATTGGAAATTCTGTAGACCCAAAGATCGTAGACCTGCCAAACACAACACCACCAAGCATGTACATTGCCAAAGACGGATATTGTTACATCAACATTTTCTTAGCAGCAATGATAAATGTCAATGAAGACTCAGCAAAGGACTACACAAAATTCCTCAGGGATGAATTAGTCGAGCGACTTGGAAAGTGGCCGAAACTTAAAGATGTTGCAACAGCGTGCTATGCGTTATCAGTTATGTTTCCAGAGATTAAGAACGCTGAATTGCCTCCAATACTTGTTGACCACGAAAGCAAGTCAATGCACGTGATCGATTCATACGGCTCATTGAGCGTCGGATTTCACATCCTTAAAGCAAGCACTGTTGGCCAGCTCATAAAATTTCAATACGAGTCAATGGATAGTGAGATGCGCGAGTATATAGTGGGAGGTACTTTAACGACCCAAACATTTAACAAACTCCTTACATCTTTAGCTAAAAATATGTTCAAACCAGATCAAATTAAGCAGATGATTGAGGAGGAACCCTTCCTACTAATGATGGCAATCGCGTCACCAACTATGCTCATAGCGCTATACAATAATTGCTATATAGAGCAGGCTATGACGTATTGGATCGTTAAAAGCCAAGGAGTGGCAGCGATATTCGCACAACTGGAAGCACTAGCAAAGAAGACCTCACAAGCTGAATTGCTAGTCCAACAAATGCAAATACTTGAAAAGGCGTCTAATCAATTGAGGCTTGCAGTCACGGGTTTGAATCATGTCGATCCAGCCAAGCGTCTGCTATGGTCGCATCTAGAAGCAATGACAACACGATCAGAGATGAACAAGGAATTAATTGCTGAAGGCTATGCCCTGTATGATGAGCGCCTGTATGCACTTATGGAAAAAAGTTACGTAGATCAATTAAACCAATCATGGGCAGAGTTATCATTCTGTGGAAAATTTTCAGCAATATGGCGTGTGTTCAAAGTCAAGAAATATTACAAGCCGTCTTTAACCGTGAGAAAAAGCGTAGATTTAGGCGCTGTGTACAATATATCAGCTACGCATCTAATATCAGATTTAGCGCAGAGAAGTCGCGATCGAGCCAGCTCTATTTTAACCAAACTCCGCAACGGTTTTTATGATAAGTTAGAGAAGGCTAGAACTCGAGCAATTAAAACTGTTTATTGGTTCATACCTGACATATTTAGACTTATGCATATTTTCATAGTTCTTAGTTTATTAACAACTGTGGCAAACACTATTATTGTGACTATGAATGATTACAAAAAGCTTAAAAAGCAACAAAGAGAGGATGAGTATGAAGCTGAAATTAACGAGGTTCGAAAAATTCACGCTAATCTGATGAAAGAGCACAATGACAATCTAACATGTGAACAGTTTATCGAACATATGCGCCAGACGCATCCACGATTAATTGAAGCCACATTGGAGTTGACACACACAGGCGTCATTCATGAAGGAAAGAGCAACCTAGAAACCAATCTCGAGCAAGCTATGGCAGTGGGCACTTTACTCACAATGATGTTAGACCCACAGAAGAGTGATGCAGTATACAAAGTGTTGAATAAGATGAGAACTGTAATTAGCACATTTGAGCAGAATGTCCCATTTCCTTCAATTAACTTTACAAATATTTTAACACCATCAGTGGCGCAACAAAGTGTGGATGTTGATGAACCTCTAACTTTAAGTACTGACAAGAATCTAACAATAGATTTTGATACTAATCAAGATTTACCAGCGGACACGTTTAGCAATGATGTTACTTTCGAAGATTGGTGGGCAAATCAAATAAGCAACAACAGAACAGTTCCACATTATCGGCTTGGTGGCAAATTTGTAGAATTCACAAGAGAAAATGCAGCACATGTTAGCATTGAGCTCGCACACTCAAACCTTGAGAAGGAGTTTCTACTCAGAGGAGCTGTTGGCTCAGGAAAATCCACTGGTCTTCCATATCATCTTAGTATGCGTGGTAAGGTGCTTTTAATAGAACCAACAAGACCACTAGCCGAGAATGTCTGCAGACAGCTTCAAGGACCACCATTCAATGTCAGCCCGACACTACAAATGCGTGGTTTAAGCTCTTTTGGTTGTACGCCAATTACGATAATGACATCAGGTTTCGCACTGCACATGTATGCAAATAATCCGGATAAGATCTCTGAATACGATTTTATAATATTTGATGAGTGTCATATCATGGAAGCGCCAGCCATGGCGTTCTACTGTTTGTTGAAAGAATATGAATATAGAGGCAAAATCATTAAGGTGTCCGCAACACCACCAGGACGAGAGTGTGAGTTCTCTACTCAGCATCCAGTTGATATCCATGTGTGCGAAAATCTCACTCAGCAACAATTCGTTAGAGAGCTTGGTTCCGGATCAAACGTCGACGCAACAAAGTATGGAAACAACATACTTGTGTATGTTGCAAGTTACAACGACGTCGACTCATTAGCGCATGCTTTAACTGAATTACACTATTCAGTCATAAAGGTTGATGGGAGAACAATGAAACAGAACACTACAGGGATAGTGACAAATGGCACCTCTCAAAAGAAGTGTTTTGTCGTTGCTACGAATATCATCGAGAATGGTGTCACATTAGATGTCGATGTTGTGGTCGACTTCGGACTCAAAGTAACAGCTGAATTAGATGTTGACAACAGAGCTATTCTATACAAGCGTGTTAGCATTTCATATGGTGAGCGAATTCAAAGGCTAGGGCGTGTTGGAAGAAACAAACCCGGAACAGTCGTTCGAATTGGAAAAACCATGAAGGGTTTGCAAGAAATCCCAGCCATGATAGCGACAGAAGCAGCATTCATGTGCTTTGCATACGGACTAAAGGTCATAACACACAATGTTTCAACAACACACTTAGCAAAGTGCACAGTCAAGCAAGCACGAACAATGATGCAATTTGAGCTTTCACCATTTGTAATGGCAGAACTAGTCAAATTTGATGGCTCAATGCACCCACAAATCCATGAAGCACTTGTGAAATATAAGCTTAGAGATTCTGTAATAATGCTCAGACCTAACGCAATTCCCAAAGTTAATTTTCACAACTGGCTGACAGCACGTGATTACAACAGGATGGGTTGTTCACTAGAGCTAGAGGATCATGTCAAAATTCCATATTACATACGAGGCGTCCCTGACAAGTTATACGGGAAATTGTATGATATCATTTTACAATATAGTCCAACCAGTTGTTACGGAAGACTATCAAGTGCTTGTGCTGGGAAGGTAGCATACACCTTGCGAACGGACCCTTGTTCGCTACCACGAACAATAGCAATAATCAATGCATTGATCACAGAGGAGTATGCTAAGAGAGATCATTACCGTAATATGGTAGCAAATCCTTCGTCTTCGCATGCATTCTCACTAAATGGATTAGTTTCCATGATTGCTTCAAGATACATGAAAGACCACACAAAAGAGAATATAGACAAACTTATTAAGGTGCGAGATCAACTACTCGAATTTCAAGGCATGGGTATGCAATTTCAAGATCCATCAGAACTAATGGACATTGGCGCCTTGAACACAGTTATACATCAAGGAATGGATGCAACAGCTGCATGCATTGGTCTCCAAGGACGATGGAATGCATCACTCATACAACGTGATCTCATGATTGCAGGAGGAGTATTCATTGGAGGAATATTAATGATGTGGAGCTTGTTCACTAAGTGGGGAACGATCAATGTATCACACCAAGGAAAGAACAAGCGTAGCCGACAAAAATTGAAGTTCAAGCAAGCTAGAGACACTAAATACGCATATGATGTGACAGGATCGGAAGAAACTCTTGGTGAAAACTTTGGAACAGCTTATACAAAGAAGGGCAAAGGAAAAGGAACCAAAGTTGGTCTTGGAGTGAAGCAGCATAAATTTCACATGATGTATGGTTTTGATCCTCAAGAGTACAACCTAATTCGTTTTGTTGACCCTCTTACAGGTGCAACATTAGATGAACAAATCCATGCCGATATTCGCTTAGTGCAAGAACACTTTGACATAATCCGAGAAGAGGCAGTCGCAAACGACACAATTGAGCGACAACACATATACGGAAATCCTGGTCTCCAAGCATTCTTCATACAGAATGGATCAGCTAATGCATTAAGAGTTGATTTAACACCACATTCACCTTTGCGTGTTATGACGAACAACAACATAGCAGGATTTCCAGAATATGAGGGCACATTACGACAAACTGGCACAGCTCTCACTGTACCTGTGAATCAAGTACCGGCAGCAAATGAGACAGGAGTTGCCCACGAATCCAAATCTATGATGGCTGGATTGGGCGATTACACACCTATTTCTCAGCAGCTCTGTCTCGTACAAAACGATTCTGAAGGAGTCAAACGTAATGTGTATGCAATTGGATATGGATCATATTTAATATCACCGGCGCATCTTTTCAAGTATAACAATGGTGAAATCACAATTAAATCCTCAAGAGGGCTGTATAAAATTAGAAATTCAGTTGATGTCAAATTGCACCCGATTGCACAGAGGGACATGGTCATAATTCAACTTCCAAAAGACTTCCCACCATTCCCAATGCGGCTTAAGTTCTCAACTCCGTCAAGAGATGTGCGTGTGTGCTTAGTTGGAATCAACTTTCAACAGAATCATACCACGTGCATAATATCCGAAAGCAGTGTGACAGCACCCAAAGGAAATGGTGACTTCTGGAAACATTGGATTTCAACTGTTGATGGGCAATGTGGGCTACCGTTAGTTGACGTTAAGAATAAACACATTGTCGGAATTCACAGCCTGGCTTCAACAAGTGGAAATACGAACTTTTTCGTTGCAATGCCTGAGAACTTCAATGAATATATATCTAATCTCGTGCAAACGAATAAGTGGGAAAAGGGATGGCATTACAACCCAAATCTTATTTCATGGTGTGGTCTAAACCTAGTTGATTCAGCACCTAAAGGATTGTTTAAAACATCAAAACTTGTTGAAGATTTGGATATGAGCGTTGAAGAACAATGCAAGGTGACAGAGACATGGTTGACGGAACACATCCAGGATAATCTACAGGTCGTTGCAAAGTGTCCAGGCCAACTTGTAACAAAGCATGTCGTTAAAGGCCCATGTCCGCATTTTCAACTGTATTTATCCACACATGATGAGGCAAAGTTGTACTTTTCACCTTTGCTTGGAAAGTATGACAAGAGTAGGTTGAATAGAGCAGCATTTATCAAAGATCTTTCAAAGTACGCAAAACCGATTTATATTGGAGAGATCAATTATGAAATCTTTGATAAGGCAGTTGATCGAGTTATAAGCATCCTCAGAAGTGTAGGAATGCTACAGTGTACATACGTGACGGACGAAGAAGAAATTTTCAATTCATTAAATATGAACGCAGCCGTAGGTGCACTCTATACAGGAAAGAAGAAAGACTATTTCAAAGATTTCTCGAATGATGACAAAGCCGAAATCATCATGCGTTCATGTGAGCGCATCTACAATGGACAATTGGGTGTATGGAACGGTTCACTCAAAGCTGAAATACGACCAATAGAGAAAACCATACTAAACAAGACACGCACTTTCACAGCAGCGCCATTAGAAACTCTACTTGGTGGGAAAGTATGTGTGGACGATTTCAACAATCAATTTTATTCACATCACCTTGAAGGCCCATGGACCGTAGGAATCACAAAGTTTTATGGAGGATGGAACCGACTTTTGGAGAAATTGCCAGAAGGATGGATTTATTGCGATGCAGATGGATCCCAGTTTGACAGCTCACTAACTCCATATCTTATTAATGCTGTATTGCACATTCGCTTACATTTCATGGAAGAATGGGAGTTGGGAGCTCAGATGTTGCGAAATTTATACACAGAGATTGTTTATACGCCAATCGCAACGCCTGATGGGTCTGTCATCAAGAAATTCAAAGGAAATAATAGTGGGCAACCATCTACAGTCGTTGACAACACGCTTATGGTTATTATAGCATTTAATTACGCAATGTTGTCAAGTGGCATTCCTGAAGACAAAATTGACGACTGCTGTAGAATGTTTGCAAACGGTGACGACTTACTCTTGGCAGTGCATCCGGATTACGAATATATATTGGACGGATTTCAAAATCATTTTGGAAACCTTGGCCTTAACTTTGAGTTCACATCGAGGACAAAGGACAAATCAGAGTTATGGTTCATGTCAACACAAGGAGTCAAGTGTGAAGGTATCTACATACCAAAACTCGAAAGGGAAAGGATAGTTGCAATCCTTGAATGGGATCGATCGAACTTGCCTGAGCATCGACTTGAAGCTATCTGTGCAGCCATGGTTGAAGCATGGGGATACCCCGATTTAGTTCATGAAATTCGAAAGTTTTACGCGTGGCTTCTTGAAATGCAACCCTTCGCGAACCTGGCAAAGGAAGGCATGGCGCCATACATAGCAGAAACAGCACTCCGCAACCTCTACCTTGGAACAGGCATCAAAGAAGAAGAAATTGAAAAATACTTTAGACAGTTTGTCAAGGATCTTCCTGGATACGTAGAAGATTACAATGAAGAAGTTATTCATCAATCTGGTCAAGTTGACGCAGGGAGACAGGGCGGTAGCGGCGCTCAAGGAGGCACACCGCCAGCAGGA---------------------------------------------------AGTGGAGGCAC----TGGATCTGGCACTCAAGGCAATGGGGGTCAGACGGGA---------TCCCAAG---GAAGTGGCGGTCAACAAGGGTCCGGTGGGGGCACTGGTCAAGGAGCAGCTGGAAACAACGGCGG------------AGGTCAGACAGGAGGCTCTAGTGGG------------ACAGCTGGTCAGAGAGATAAGGACGTTGACGCAGGCTCGGCTGGAAAGATATCCGTACCAAAGCTTAAAGCCATGTCAAAGAAAATGCGCTTGCCAAAGGCAAAAGGAAAAGACGTCTTGCATTTGGACTTTTTGTTGACATACAAGCCACAACAGCAGGACATATCGAACACAAGAGCAACTAAGGAAGAGTTCGATAGATGGTACGACGCCATAAAGAAGGAGTACGAGATCGATGATACACAAATGACAGTCGTCATGAGTGGTCTGATGGTCTGGTGCATTGAAAATGGTTGCTCACCAAACATAAACGGAAATTGGACGATGATGGATGGGGATGAACAAAGAGTTTTCCCACTAAAACCAGTTATTGAAAACGCATCTCCAACTTTTCGACAAGTTATGCATCATTTCAGTGATGCAGCTGAAGCGTATATAGAATACAGAAATTCTACTGAGCGATACATGCCAAGATATGGACTTCAGCGAAATCTCACCGACTATAGCTTAGCGCGGTATGCTTTTGATTTCTATGAAATGACTTCACGCACACCAGCTAGAGCTAAGGAAGCCCACATGCAG---ATGAAAGCCGCAGCAGTTCGTGGCTCAAACACACGACTGTTCGGCTTGGACGGAAATGTCGGCGAGACTCAGGAGAATACAGAGAGACACACAGCTGGCGACGTTAGTCGCAATATGCACTCTCTGTTGGGAGTGCAGCAACATCACTAGTCTCCTGGAAACCCTGTTTGCAGTACCTATAATATATACTA------ATATATAGTACGTTGGTGAGGCTTTGCCTC-----------------------GGTCTTACTATCTTATTATGTATGTATTTACAGCGTGAACCAGTCTGCAGCATGCAGGGTTGGACCCAGCGTGTTCTGGTGTAGCGTGTACTAGCGTCGAGCCATGAGACGGACTGCACTGGGTGTGGCTATGCCACTTGTGTTGCGAGTTTCCTGGTAAGAGAC----------------

>AY569692

--------------------AAAAACAACAAGACTCAACACAACACAACAAAACACAACCAAGCAAATCCAAGTTTCCTTTACTCAGATTGTAGTGAACGGCTCGGTAGGAAAGGTTCCTCGAGATCACTCTCTGATTCTTCTCTC----------TCAACCAACTTCATTCAAGCGAGATGGCGGGCTCTTGGACTCACGTGACATACAAGTGGCAACCAGATGTCAACAACGCACGTGATGTGAAAAGAGTGATGGAGATGTTTGCAGCAAAACATCAACATTACACTGAGGAGCAAAGGCTTGCTCACAACAGCAAGTTATTAAGGAAGGCTTGTGTCACTAGTGCTGAGTTTATTGAACCAGCACAGAAACCAAAATGTCACCAGACATGGGTTGAAAAGTGCGACCACAACCCCACAGAGCACTTTGTTTATCAACGCTCT---ACACCTGAGAAGAAAGTGCTTAGCACCAAACCTAAGACAACCTCTGTTACGAAGTTAATCAGGGATGTCCTTGAGATTTCGAAGGGCAGTGGGATAAAAATTGAGTTAATTGGCAAGCGTATCAAACGTAAGACTCAATTGTCCATAAGGCAACACAATGGCAAAGACTTCTTGCACTGCAAAACCAGGCATGAGAATGGCCTGTTTAAACGCAAGGACATTGACATTAATGTCAAGTGGTTGCCCACCATTGAAGCCATTGCAAAATGCTACAGCACGGTGAATGCGGAAGAACTGCAAAGTCTCAATAGAGGCAGTAGTGGTCTTACATTCATGCAAAACGATGAATTATTCATCGTGCGTGGAAGGATGCATGGTGAGATTGTCAATAGTTTACACGAAAATAAGCATGTTATGGAAATTGAACACTATGCTGATCCACAAGCAAACAGTTTCTGGAAAGGTTATACAGATGCATATGTCGGGAACAGAAACATATCTACCACTCACACAGAGCACACACCAACTATTAATTTAGAAGAGTGTGGCAAGAGAATGGCACTCTTAGAAATCTTATTCCATTCAACTTTCAAAATAACATGCAAAACGTGCAATATTGATGACCTCGAATTATCAGATGATGAATTTGGGGCCAAGTTATACAGTAATCTGCAACGCATTGAAGAAAGGCAACGTGAATATCTTGCTAAAGACCAAAAACTTTTACGCATGATTCACTTTGTGAAGGACCGGTGCAATCCAAAATTTTCGCACTTGCCCTTGCTATGGCAAGTAGCAGAAACAGTAGGACATTATACTGATAACCAATCGAAGCAGATAATTGATATCAGTGAGGCGCTCATAAAAGTTAATACCTTAACTCCTGATGATGCAGTAAAGGCCAGTGTAGCACTATTGGAAGTTGCACGATGGTATAAAAATCGGAAGGAATCACTCAAAACAGACACACTAGACTCATTCCGAAACAAAATTTCACCAAAGAGCACGATCAACGCAGCATTGATGTGCGACAATCAGTTAGATAAGAATGCTAATTTCGTATGGGGAAACAGAGAATATCATGCAAAGCGATTCTTTGCCAATTATTTTGAAGCTGTAGACCCAACTGATGCGTATGAAAAACACGTCACACGCTTCAACCCCAATGGACAGCGGAAATTATCAATTGGCAAGTTAGTAATTCCACTAGATTTCCAGAAGATTAGAGATTCGTTTGTTGGCTTATCGATAAATAAACAACCATTGAGCAAAGCTTGTGTGAGCAAAATTGATGGAGGCTACGTATATCCATGTTGCTGCGTTACAACGGAATTCGGAAAACCAGCATATTCTGAAATAATACCTCCAACAAAAGGACATATCACGATTGGAAATTCAGTGGACCCAAAAATAGTAGATTTGCCAAACACAACTCCACCGAGTATGTACATCGCAAAAGATGGATATTGTTATATTAACATATTCTTAGCAGCAATGATAAACGTCAATGAGGAATCCGCGAAAGACTACACGAAGTTCCTTAGAGACGAATTGGTGGAACGGCTCGGCAAGTGGCCTAAATTGAAAGATGTAGCAACAGCATGTTACGCTTTATCAGTAATGTTCCCAGAGATCAAGAATGCCGAATTACCACCAATATTGGTTGATCATGAGAGCAAGTCAATGCATGTCATTGATTCATATGGATCACTCAGTGTTGGATTCCACATTCTAAAGGCAAGTACCGTGGGACAACTGATAAAATTCCAGTACGAATCATTAGAAAGCGAGATGCGTGAATACATAGTGGGAGGTACTTTGACACAACAAACTTTTAACACACTTCTCAAGACTCTCACAAAGAACATGTTCAAGCCAAATAAAATAAAACAGATAATAGAGGAAGAACCTTTCCTACTAATGATGGCGATTGCATCCCCTACCGTGCTTATCTCACTGTATAACAATTGCTACATCGAGCAGGCGATGACATACTGGATTGTCAAGAACCAAGGAGTCGCGGCAATTTTTACACAGTTGGAGGCTTTGGCAAAGAAAACCTCCCAAGCAGAACTATTAGTTCTGCAAATGCAAATACTCGAAAAAGCTTCAAACCAGCTAAGACTTGCAGTCACCGGACTTAATCATGTCGATCCAGCGAAACGACTCCTGTGGTCTCACCTAGAAGCCATGACAACACGATCGGAAATGAATAAGGAACTCATAGCGGAAGGTTATGCACTATATGATGAGCGTCTATATACCCTAATGGAAAAAAGTTATGTAGATCAATTAAACCAGTCATGGGCAGAATTATCATACTGTGGAAAATTTTCAGCAATATGGCGTGTGTTCAGAGTCAAGAAATACTACAAGCCATCTTTAACCGTGAGAAAAAGCGTAGATTTAGGCGCTGTTTACAATATATCAGCTACGCATCTAATATCAGATTTAGTGCAGAGAAGTCGAGATCGAGTCAGCTCTACTTTAACCAAACTCCGCAACGGTTTCTATGACAAAATGGAAAAAGCGAGGGTTAGCGCAGTGAGGACTGTATATTGGTTCATACCCGACATATTTAGACTAGTCCACATCTTTATAATTCTAAGTTTATTAACTACTATAGCAAATACGATAGTTACAACCATGAATGACTACAAAAAGTTAAAGAAGCAACAAAGAGAGGACGAGTATGAGGCCGAAATTAATGAGGTGCGGAAAATACATGCCAATTTGATGAAGGAGCACAATGACAATCTGACATGCGATCAGTTTATTGAATACATACGCCAAACACACCCACGCCTCATTGAAGCAACTTTGGATTTGACACATACAGGCGTTATTCACGAGGGTAAATCCAGTCTAGAAACCAACCTTGAGCAAGCTATGGCTGTGGGAACACTACTCACGATGATACTTGATCCTCAGAAAAGTGATGCTGTGTATAAAGTTCTAAACAAAATGCGAACAGTGATTAGCACATTTGAACAGAATGTCTCATTCCCTTCAATTAACTTCACTAACATCTTGACACCACCCGTAACACAGCAGAGTGTGGACGTTGATGAGCCTCTTACTTTGAGCACTGACAAGAACTTAACGATAGATTTTGATACAAATCAAGATTTACCTGCAGACACTTTCAGTAACGATGTTACATTCGAGGATTGGTGGGCAAATCAGATAAGCAATAATAGAACAGTCCCACACTATCGTCTCGGTGGTAAGTTTGTAGAATTTACAAGGGAAAACGCAGCACTTGTTAGCATTGAATTAGCTCACTCGAACATTGAAAAGGAATTTCTTTTAAGGGGTGCGGTTGGCTCAGGAAAGTCCACAGGGCTGCCATACCATCTTAGTGCGCGTGGAAAAGTACTTCTAATTGAACCAACAAGACCACTTGCCGAGAATGTATGCAGACAGCTACAAGGGCCACCATTCAATGTTAGCCCGACACTTACAATGCGTGGGTTAAGCTCTTTTGGCTGCACGCCAATCACGATCATGACCTCTGGTTTTGCATTACATATGTACGCAAACAATCCGGACAAGATATCTGATTATGACTTCATCATATTTGATGAATGTCATATCATGGAAGCACCAGCTATGGCATTTTATTGTTTGCTGAAGGAATATGAATATCGAGGCAAAATCATTAAAGTCTCCGCGACACCACCTGGAAGAGAATGTGAATTTACCGCACAACATCCAGTTGACATTCACGTCTGCGAGAACCTCACACAACAGCAATTTGTAAGGGAACTTGGCACGGGTTCAAATGTCGACGCGACGAAATATGGAAACAACATACTTGTTTACGTTGCAAGCTACAATGACGTCGATTCGCTATCACACGCGCTAATTGAATTACATTATTCAGTTATCAAAGTTGACGGTAGAACCATGAAGCAGAACACCACAGGCATTGTAACTAATGGCACGTCACAAAAGAAGTGTTTTGTTGTTGCAACGAACATAATCGAGAATGGTGTTACATTGGATGTTGATGTCGTAGTGGACTTTGGACTAAAGGTCACAGCTGAGCTGGATGTTGACAATAGAGCAATTCTATACAAACGTGTCAGCATTTCGTATGGTGAGCGTATTCAAAGACTCGGTCGTGTTGGAAGGAATAAACCCGGAACAGTGGTCCGCATTGGAAAAACTATGAAAGGTCTGCAGGAAATCCCAGCGATGATAGCAACGGAAGCTGCTTTTATGTGTTTTGCATACGGACTCAAAGTTATAACACACAATGTATCTACAACACACTTAGCTAAATGCACGGTTAAACAAGCAAGAACAATGATGCAATTTGAACTATCACCATTCGTAATGGCAGAATTAGTTAAATTCGATGGTTCAATGCATCCACAAATTCATGAAGCACTCGTAAAATACAAACTTAGAGATTCTGTGATAATGCTAAGACCAAACGCTATCCCGAAGGTCAACTTCCACAACTGGCTGACTGCACGTGATTACAACAGGATGGGCTGTACAGTAGAACTCGAAGATCATGTGAAAATACCGTACTATATACGAGGGATTCCTGATAAACTATATGGAAAATTGTACGATATCATTTTACAATACAGCCCAACTAGTTGTTATGGAAGACTATCAAGTGCCTGTGCGGGAAAAGTAGCATATACCCTGCGCACTGATCCTTGTTCGTTACCACGCACGATAGCTATCATTAACGCATTAATTACTGAAGAGTATGCAAAGAGGGACCATTACAGAAACATGATAGCAAACCCCTCATCATCGCACGCCTTTTCACTCAATGGGCTAGTATCCATGATCGCTTCTCGGTATATGAAAGACCACACGAAGGAAAACATTGATAAGCTTGTAAGAGTGCGCGACCAACTACTTGAGTTCCAAGGCACAGGTATGCAATTTCAAGATCCTTCAGAATTGATGGACATTGGTGCATTAAACACAGTTATTCACCAAGGAATGGACGCCACGGCTGCTTGTATTGGATTGCAAGGGCGCTGGAATGCTTCGCTCATTCAACGCGACTTGATGATATCAGCAGGAGTCTTCACAGGAGGAATTTTCATGATGTGGTATCTTTTCATAAAATGGAGCAAGACAGAAGTGTCACACCAAGGAAAGAACAAACGCAGCCGGCAGAAACTACGATTCAAAGAGGCTCGTGATAACAAATATGCCTATGATGTGACAGGGTCAGAAGAAGTTCTTGGTGAGAATTTTGGAACTGCTTATGTTAAGAAAGGAAAAGGAAAAGGAACAAAAGTTGGTCTTGGAATCAAACAACACAAATTCCACATGATGTATGGTTTTGACCCACAAGAATACAACTTAATCCGTTTCGTGGATCCTCTAACAGGTGCAACACTAGACGAGCAAATTCATGCAGATATACGTCTAGTGCAAGAGCACTTTAGCGTCATTAGAGATGAAGCAGTGGCAAATGACACAATTGAAAGACAACATATTTATAGCAATCCTGGACTACAAGCGTTCTTCATACAAAATGGATCGGCGAATGCACTGAGAGTTGACTTAACACCGCATACACCACTGCGTGTCGTGACCAATAACAATATTGCAGGTTTCCCAGAATATGAAGGCACACTCCGACAAACAGGAACAGCTTTACAAATACCTGTTAATCAAGTTCCAGCTGCGAATGAAACAGGAGTAGCACATGAGTCAAAATCAATGATGGCAGGACTAGGTGATTACACACCCATATCACAGCAATTATGTTTAATCCAGAATGACTCTGATGGAATTAAAAGGAATGTCTATTCAATCGGATATGGGTCATATCTCATTGCGCCAGCACATTTATTTAAATATAACAATGGTGAGATAACGATCAAATCATCACGAGGCTTGTACAAGATCAGAAACTCAGTTGAAATCAAGTTACATCCCATTGCACATAGAGATATGGTTATAATTCAACTTCCAAAGGATTTTCCACCATTCCCAATGCGCCTTAAGTTTTCCAAACCATCCAGAGAGTCAAGAGTGTGCTTAGTTGGAGTTAACTTCCAACAAAACTACAGCACATGCATTGTTTCGGAGAGTAGCGTCACAGCACCAAAAGGCAACGGAGATTTCTGGAAACACTGGATATCCACAGTGGACGGACAATGCGGCCTCCCATTAGTAGACGTCAAGAGCAAGCACATAGTTGGAATACACAGCCTTGCATCAACTAGTGGAAACACTAACTTTTTCGTCGCCATGCCTGAAGATTTCAATGACTACATTCACAATCTTGTGCAAACCAACAAGTGGGAAAAAGGATGGCATTACAACCCAAATCTCATTTCATGGTGTGGTCTCAATCTAGTTGACTCAGCTCCAAAGGGTCTCTTCAAAACTTCGAAATTAGTGGAAGACCTTGACATGAGTGTTGAAGAGCAGTGCAAGGTTACAGAAACATGGCTCACAGAATGCATTCAGGATAATTTACAGGTTGTCGCAAAATGCCCAGGCCAACTTGTCACCAAGCACGTTGTCAAAGGCCCATGCCCACACTTTCAGCTATATCTGTCAACACATGATGAAGCCAAAGCATACTTTGCACCACTACTCGGAAAATACGATAAGAGCAGATTAAACAGAGCAGCTTTTATCAAAGACATTTCAAAATATGCAAAACCAATCTATATTGGAGAAATCAATTACGATGTCTTTGAAAAAGCTATAGAACGTGTGATTAAAATCCTTAAAGATGTGGGAATGCAACAATGCACGTACGTCACGGATGAGGATGAAATATTCCAGTCACTCAACCTCAATGCCGCAGTTGGTGCCTTATACACAGGAAAGAAGAAAGACTATTTCAAGGATTTCTCAAATGAGGATAAATCAGAAATCATTATGAGATCCTGTGAGCGTATCTACAACGGACAACTCGGTGTGTGGAATGGTTCTCTCAAAGCTGAAATAAGGCCTATAGAGAAAACAATATTAAATAAGACTCGGACTTTTACAGCAGCACCATTAGAAACTTTACTTGGTGGCAAGGTCTGTGTCGATGATTTCAACAACCAATTCTACTCGCACCATTTAGAAGGTCCTTGGACAGTTGGAATAACAAAGTTTTATGGTGGGTGGAACCGTTTGTTGGAAAAATTGCCAGATGGTTGGATATACTGCGACGCCGATGGATCACAGTTCGATAGCTCTTTGACACCATATCTCATCAATGCCGTATTACACATTCGATTACAATTCATGGAAAAATGGAACTTAGGAGAACAAATGTTGCGAAATTTGTACACTGAAATCGTGTACACACCAATTGCAACACCAGATGGATCTGTAATCAAGAAATTTAAAGGGAATAACAGCGGGCAGCCGTCAACAGTTGTAGACAACACACTCATGGTGATATTAGCATTTAATTATGCAATGTTATCAAGTGGTGTTAAAGAGGAAGAAATAGACAATTGCTGCCGAATGTTCGCCTATGGTGATGATCTGCTACTTGCAGTGCATCCAAAGTTCGAACACATATTGGACGGATTTCAAAATCACTTTGGAAACCTAGGTCTCAACTTTGAGTTTACATCACGAATAAGAGACAAGTCAGAGTTATGGTTCATGTCCACACGAGGTATCAAATGCGAAGGCATCTATATACCAAAGCTTGGGAAAGAAAGAATAGTTGCCATACTCGAGTGGGATCGGTCAAACTTACCTGAGCACCGTCTCGAAGCCATTTGTGCAGCCATGGTAGAGGCATGGGGATACCCAGACCTTGTCCAAGAGATACGGAAATTTTATGCGTGGCTTCTCGAAATGCAACCATTTGCAAATTTAGCTAAAGAGGGCCTAGCACCATATATCGCTGAAACTGCACTCAGAAATCTGTATTTGGGCACAGGGATCAAGGAAGAAGAGATTGGGAAATATTTCAAACAGTTTGCCAAGGATCTCCCTGGTTATATAGAAGATTACAATGAAGACGTTTTCCATCAATCCGGAACTGTGGATGCAGGAGCCCAAGGAGGGGGAAGTGGATCTGGAACAACGCCACCAGCAACA---------------------------------------------------GGTAGTGGAAC----AGGAACAAGAACACCATCTACTGGAACTCCAGCACAAGGCAACACACCTCCAG---CATCAGGTGGATCATCAGGAAACAATGGAGGC---GGCCAATCAGGTTC---AAACGGCACTGG------------AGGCCA---AGCAGGCTCAAGCGGA------------GCAGGGGGTCAAAGAGACAAAGATGTTGACGCTGGCTCAACAGGGAAGATATCAGTGCCAAAGCTTAAGGCAATGTCGAAGAAAATGCGCCTGCCAAAGGCGAAAGGAAAAGACGTTTTACACTTAGATTTCCTGTTAACATATAAACCACAACAGCAAGATATATCAAACACTAGAGCAACTAAGGAAGAGTTTGATAGATGGTACGATGCCATAAAGAAGGAGTACGAAATTGATGACACACAAATGACAGTTGTCATGAGTGGTCTCATGGTATGGTGCATCGAAAATGGTTGCTCACCAAACATAAACGGAAATTGGACGATGATGGACGGAGACGAACAAAGGGTTTTTCCATTAAAGCCAGTCATTGAGAACGCATCTCCAACTTTCCGACAAATAATGCATCATTTTAGTGATGCAGCTGAAGCGTATATAGAGTACCGAAACTCTACAGAGCGATACATGCCAAGATACGGTCTTCAGCGAAATCTCACCGACTATAGCTTAGCGCGGTATGCTTTCGATTTCTATGAAATGACTTCGCGCACACCAGCTAGAGCTAAGGAAGCCCACATGCAG---ATGAAAGCCGCAGCAGTTCGTGGTTCAAACACACGTCTGTTCGGTCTGGACGGAAATGTCGGCGAGACTCAGGAGAATACAGAGAGACACACAGCTGGCGACGTTAGTCGCAACATGCACTCTCTGTTGGGAGTGCAGCAGCACCACTAGTCTCCTGGAAACCCTGTTTGCAGTACCTATAGTA--TATTA------CTAAATAGTACGTTCGTGAGGCCTTGCCTCGTGTGTATGTGAGGTTCTACCTCGTATTTACTATTTCAGTA--TATGTACTTTTAGCGTGAACCAGTCTGCAGGACACAGGGTTGGACCCAGTGTCTTCTGGTGTAACGTGTACTAGCGTCGAGCCAATGGACGGACGGCACTGGGAGTGGTTTTACCATTGGTGCTGCGAGTCTCTTGGTGAGAGAC----------------

>GU474635
[truncated: 175,705 more chars]
